# A comparative, correlate analysis and projection of global and regional life expectancy, healthy life expectancy, and their GAP: 1995-2025

**DOI:** 10.7189/jogh.10.020407

**Published:** 2020-12

**Authors:** Xinxi Cao, Yabing Hou, Xinyu Zhang, Chenjie Xu, Peng Jia, Xiaomin Sun, Li Sun, Ying Gao, Hongxi Yang, Zhuang Cui, Youfa Wang, Yaogang Wang

**Affiliations:** 1 School of Public Health, Tianjin Medical University, Tianjin, China; 2 Department of Earth Observation Science, Faculty of Geo-information Science and Earth Observation (ITC), University of Twente, Enschede, Netherlands; 3 Global Health Institute, Xi’an Jiaotong University Health Science Center, Xi’an, Shanxi, China; 4 Nursing Laboratory Center, Tianjin Medical University, Tianjin, China; 5 Health Management Centre, Tianjin Medical University General Hospital, Tianjin, China; 6 Department of Biostatistics, Yale School of Public Health, Yale University, New Haven, Connecticut, USA

## Abstract

**Background:**

Life expectancy (LE) and healthy life expectancy (HALE) are indicators measuring the national health level. GAP is the difference between them. This study systematically analyzed and projected LE, HALE, and GAP across global regions from 1995 to 2025.

**Methods:**

We obtained the data of 195 countries/regions on their LE, HALE, and influencing factors from 1995 to 2017. We compared the overall changes of LE, HALE, and GAP. Multiple linear regression analysis examined relationships among LE, HALE, GAP, and the associated factors. Using the Autoregressive Integrated Moving Average (ARIMA) model, we projected trends in LE, HALE, and GAP for 2017-2025.

**Results:**

During 1995-2017, LE, HALE, and their GAP in 195 countries/regions in the world showed overall increasing trends. Global average LE increased from 66.20 to 72.98 years, HALE from 57.59 to 63.25 years, and GAP from 8.62 to 9.72 years. LE and HALE in North America, Europe, and Australia were generally higher, while Africa had the lowest rates. Females' LE, HALE, and GAP were all higher than males’, but females' growth rates of LE and HALE were lower. Different factors were included to project LE, HALE, and GAP, respectively, and prediction results showed that approximately 18% of the 195 countries/regions might achieve improved LE and HALE and lower GAP.

**Conclusions:**

LE, HALE will likely continue to increase in most of countries and regions worldwide in the future and GAP will further expand. While striving to improve LE and HALE, more attention needs be made to reduce GAP and improve quality of life.

With the socioeconomic development in many countries and regions worldwide, health policies and health care technologies are constantly improving, and life expectancy (LE) is increasing in most countries and regions [1]. The World Health Organization (WHO) has called for more attention to healthy life expectancy (HALE), but the focus was only on LE as early as 1997 [2]. Both LE and HALE are indicators that reflect the health status of a population. The GAP between LE and HALE can directly reflect the unhealthy survival time of a population, but there is scant research discussing GAP. Improving quality of life and decreasing unhealthy survival time while prolonging life is a goal for all countries [3].

LE is the most commonly used indicator to assess population health [4], and socioeconomic development and health care services are important factors affecting LE [5]. However, LE cannot fully reflect the quality of life. HALE, a better indicator of comprehensive population health and the quality of life [6-8], is defined by the Global Burden of Disease (GBD) studies as “the number of years that a person at a given age can expect to live in good health, taking into account mortality and disability” [9].

Many studies have referred to the changing trends and influencing factors in either LE or HALE [10]. Some studies have mentioned the gap between LE and HALE and called the indicator LE-HALE [11], but there are few in-depth analyses of the GAP, GAP focuses on revealing the unhealthy survival time of the population and is interpreted as the average number of years of healthy life lost to poor health, which is non-fatal disability. Raising LE and HALE and narrowing GAP are the goals of the global health field [3]. It is important not only to focus on the value of the changes in GAP, but also to combine LE and HALE to explore the health changes within each country.

Thus this study systematically studied LE, HALE, and GAP in 195 countries and regions from 1995 to 2017. We also projected the future trends in LE, HALE, and GAP for 2017-2025. Our finds may help design future national health policies.

## METHODS

### Data sources

We obtained the data for 195 countries and regions on their LE, HALE, and influencing factors for 1995-2017 from Global Burden of Disease (GBD), World Health Organization (WHO), and World Bank, and some from the official statistics websites. We computed GAP from LE and HALE (GAP = LE-HALE) and calculated the health loss rate (RATE, RATE = GAP/LE). Detailed data were shown in Table S1 in the **Online Supplementary Document**).

### Statistical analysis

**Descriptive analysis:** We described the overall changes in LE, HALE, and GAP from 1995 to 2017, and by geographical distribution, economic level, and gender. We explored special outliers in different countries and years and explained the possible reasons for outliers.

**Multiple linear regression analysis:** Using multiple linear regression analysis, we explored the relationships among the factors with LE, HALE, and GAP from 1995-2012. Considering the social, economic, environmental, and disease factors, we included 15 influencing factors in our models.

**Time series forecast analysis:** We used the autoregressive integrated moving average (ARIMA) model to project LE, HALE, and GAP. The ARIMA model regarded it as a random sequence and used a mathematical model to approximately describe the sequence. Once this model was identified, future values could be predicted from the past and present values of the time series.

**Statistical analyses:** All statistical analyses were performed using IBM SPSS Statistics (V22.0) (IBM, Armonk, NY, USA). Statistical significance was evaluated at the 0.05 level.

## RESULTS

### Overall changes of LE in global regions, 1995-2017

**Figure 1:**
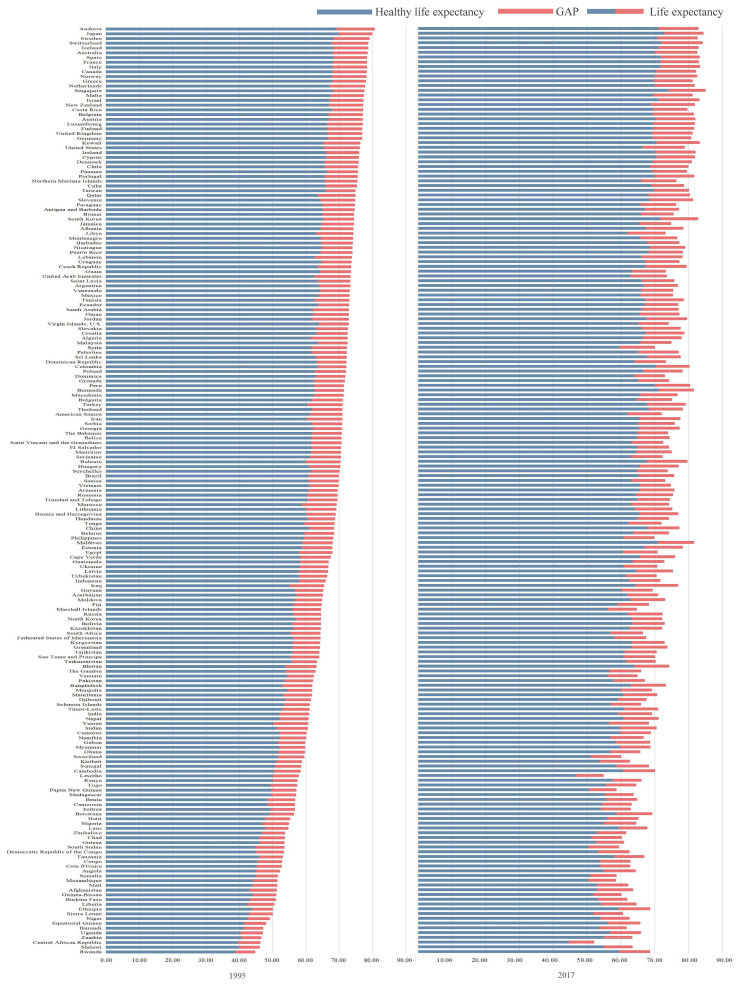
Trends of global and regional LE, HALE, and GAP, 1995 and 2017.

From 1995 to 2017, approximately 95%, 96%, and 97% of the 195 countries and regions had LE, HALE, and GAP showing an increasing trend (**Figure 1**). Global average LE increased from 66.20 in 1995 to 72.98 years in 2017, HALE increased from 58.12 to 62.96 years, and GAP increased from 8.52 to 9.33 years.

In 1995, the five countries with the highest LE were Andorra (80.45 years), Japan, Sweden, Switzerland, and Iceland; the five countries with the lowest LE were Rwanda (44.58 years), Malawi, the Central African Republic, Zambia, and Uganda. The five countries with the highest HALE were Japan (70.03 years), Andorra, Spain, Sweden, and Singapore. The five lowest countries were Rwanda (39.16 years), the Central African Republic, Malawi, Uganda, and Zambia, the difference between maximum and minimum LE is 30.87 years.

In 2017, the five countries with the highest LE were Singapore (84.79 years), Japan, Switzerland, Italy, and Kuwait, while the five with the lowest LE were the Central African Republic (51.87 years), Lesotho, Papua New Guinea, Mozambique, and Somalia. the difference between maximum and minimum LE is 32.92 years.

The five countries with the highest HALE were Singapore (74.22 years), Japan, Spain, Switzerland, and Italy; the five countries with the lowest HALE were the Central African Republic (44.75 years), Lesotho, South Sudan, Mozambique, and Papua New Guinea, the difference between maximum and minimum HALE is 29.47 years.

In 1995, China, North Korea, Seychelles, and Mongolia had the lowest RATE values. In 2017, the five countries/regions with the lowest were US Virgin Islands, China, Malaysia, the Bahamas, and Vietnam. From 1995 to 2017, the compound annual growth rates of GAP in Rwanda, Georgia, Botswana, Equatorial Guinea, and Ethiopia were the highest, while the United States, France, Canada, the United Kingdom, and Cyprus maintained relatively lower growth rates. Besides, the RATE in 68% of the countries and regions increased.

### Geographical and economic distribution characteristics of LE, HALE, and GAP

**Figure 2:**
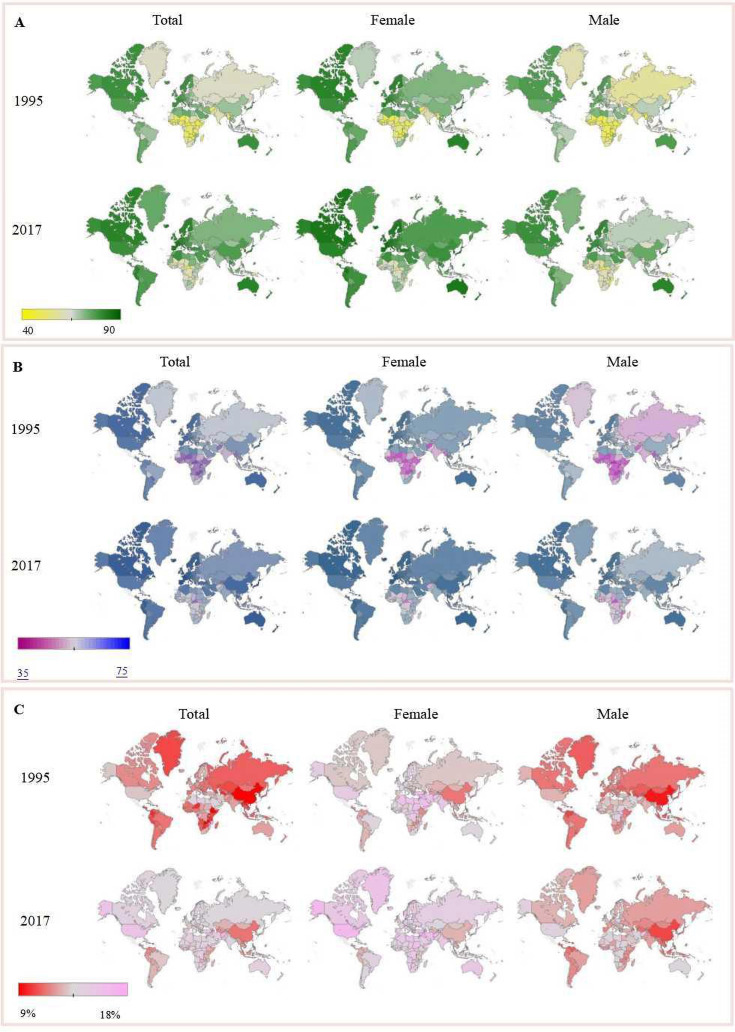
Changes in life expectancy (LE), healthy life expectancy (HALE) and RATE (GAP/LE) between 1995 and 2017 in different countries and regions and gender differences. **Panel A**. Changes in life expectancy between 1995 and 2017 in different countries and regions and gender differences. **Panel B**. Changes in healthy life expectancy between 1995 and 2017 in different countries and regions and gender differences. **Panel C**. Changes in RATE between 1995 and 2017 in different countries and regions and gender differences. GAP – difference between LE and HALE.

Geographically, LE in North America, Europe, and Australia was generally higher than that of other regions, but in Africa, it was relatively low. The top five countries with the highest compound annual growth rate of LE were Rwanda, Uganda, Ethiopia, Equatorial Guinea, and Malawi (**Figure 2****,** Panel A). HALE in North America, Europe, and Australia was generally higher than in other regions, Africa and India in particular had lower HALE (**Figure 2****,** Panel B). RATE in the United States, Australia, India, and Africa was large but relatively small in China and Central Asia, especially the Chinese males’ RATE is smaller (**Figure 2****,** panel C).

Nationally, developed countries such as Japan, Singapore, and Australia maintained high LE and showed an increasing trend, while the average growth rates of LE were lower in most developing countries. Developed countries had higher HALE than most developing regions, while HALE in China and Brazil showed a large increase from 1995 to 2017. In high- and low-income countries, RATE was large, while in the middle-income countries, RATE was smaller.

### Gender difference of LE, HALE, and GAP

There were some gender differences. Males’ LE was lower than females, but their annual growth rate was faster than females in 121 countries and regions. The gender difference in LE in most countries and regions showed a decreasing trend, but it increased in Lesotho, Syria, Swaziland, and Guam. In terms of gender, female’ HALE was higher than males’ while the female growth rates in more than half of the countries and regions were lower than the male. In most countries, the gender differences in HALE decreased, but widening gender differences persisted in Macedonia, Finland, Jordan, Mauritius, and Lesotho. In terms of gender, female GAP and RATE were both higher than male, especially in North America, Europe, and Africa. Overall, 59 countries and regions showed an increasing trend in female RATE and 67 countries and regions in male, while the growth rates of female GAP were lower than male in most countries and regions.

### Countries with special LE, HALE, and GAP

From 1995 to 2017, LE and HALE in most countries increased rapidly. In particular, Rwanda’s LE showed a sustained rapid rise, increasing from 44.58 years in 1995 to 68.46 years in 2017. HALE rising from 39.16 to 59.65 years.

In general, LE had a pronounced stratification around the age of 65 years. In most high-income and middle-high-income countries and regions, LE was above 65 years, while LE in low-income and middle-low-income countries was generally under 65 years. In general, HALE had a pronounced stratification around the age of 55 years. In most high-income and middle-high-income countries and regions, LE was above 55 years, while LE in low-income and middle-low-income countries was generally under 55 years.

**Figure 3:**
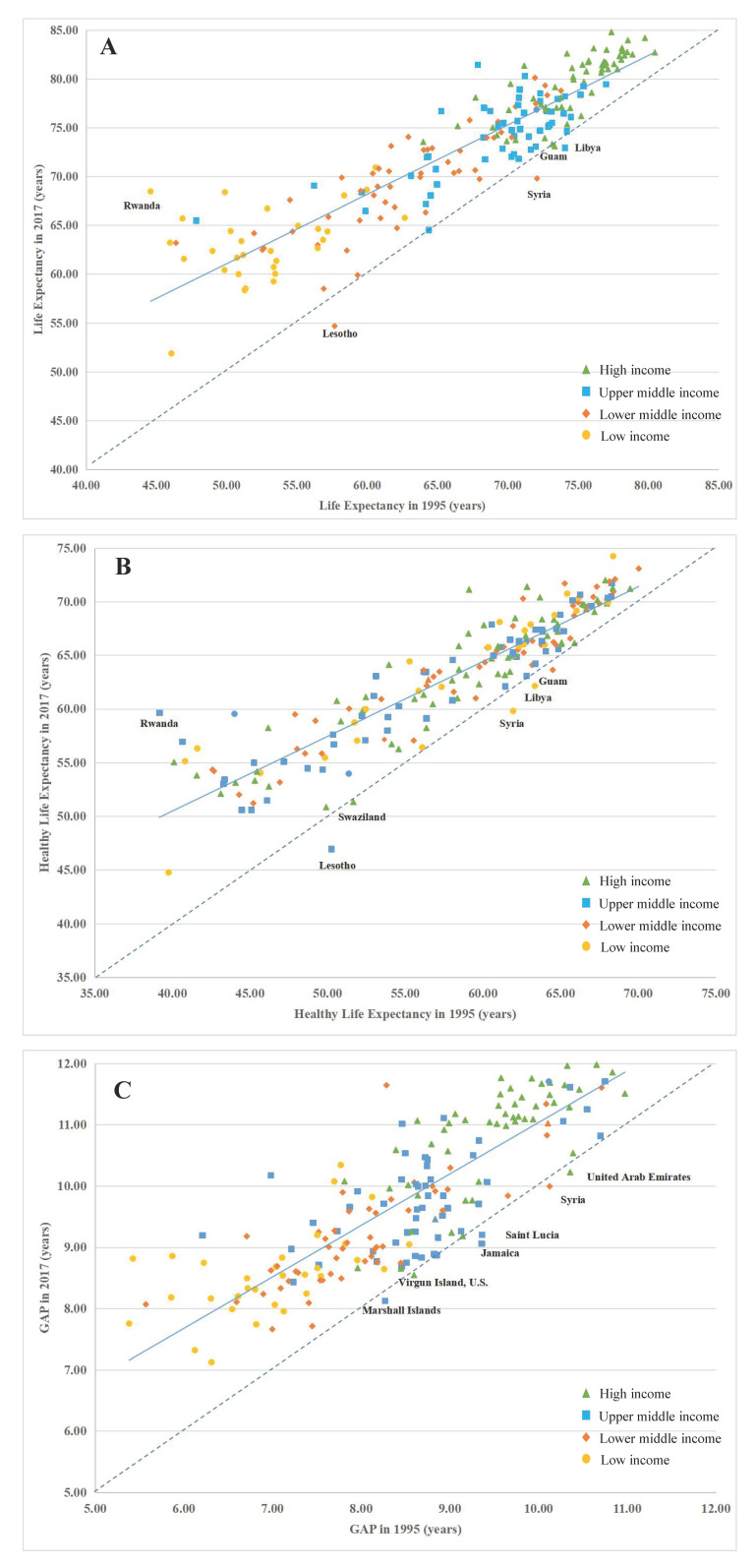
Comparison of global and regional life expectancy (LE), healthy life expectancy (HALE) and their difference (GAP) between 1995 and 2017. **Panel A**. Comparison of life expectancy, 1995 and 2017. **Panel B**. Comparison of healthy life expectancy, 1995 and 2017. **Panel C**. Comparison of GAP, 1995 and 2017.

Compared to 1995, LE in 2017 showed a decreasing trend in Guam, Syria, Libya, and Lesotho. Lesotho had the greatest decline in LE, decreasing from 57.68 years in 1995 to 54.66 years in 2017 (**Figure 3****,** Panel A). However, in some countries, HALE showed a decreasing trend (**Figure 3****,** Panel B). In 2017, HALE in Guam, Swaziland, Syria, Lesotho, and Libya were all lower than in 1995. GAP decreased in some countries and regions, for example GAP of Jamaica, Marshall Islands, Saint Lucia, Syria, United Arab Emirates, and US Virgin Islands in 2017 were lower than that in 1995 (**Figure 3****,** Panel C), GAP decreased in Syria by 9.59%, in Lesotho by 12.88%, and in Jamaica by 3.11%.

**Figure 4:**
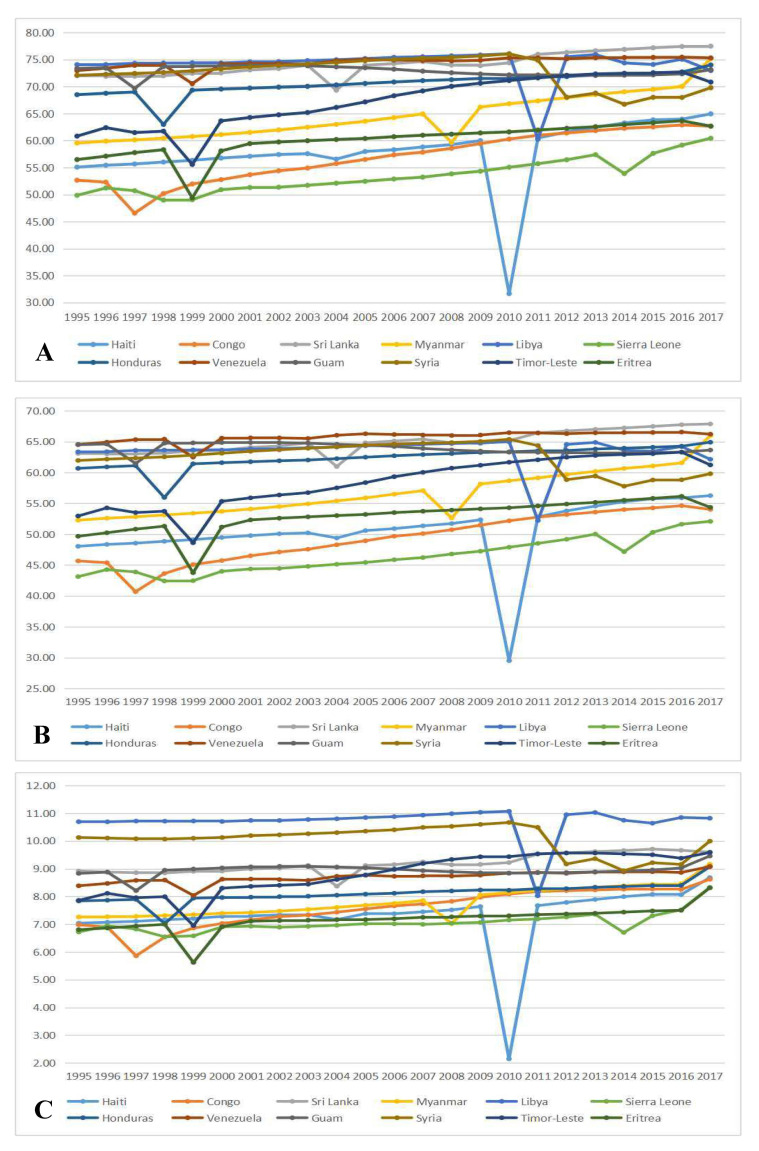
Countries/regions with special turning points of life expectancy (LE), healthy life expectancy (HALE) and their difference (GAP). **Panel A**. Countries/regions with turning points of life expectancy. **Panel B**. Countries/ regions with turning points of healthy life expectancy. **Panel C**. Countries/regions with turning points of GAP.

Some countries and regions showed significant turning points during 1995-2017 (**Figure 4****,** Panel A). Congo and Guam in 1997, Honduras in 1998, Indonesia and Eritrea in 1999, Myanmar in 2008, Haiti in 2010, and Libya in 2011 all had sharp declines in LE, while LE in North Korea increased from 63.59 years in 2002 to 69.97 years in 2003. The development of HALE had similar turning points with LE (**Figure 4****,** Panel B). For example, in 2009, Haiti’s HALE was 52.35 years, while it was 29.50 years in 2010. HALE had a decline of 43.64%. The increase in the mortality rate caused by emergencies resulted in a decrease in both LE and HALE during this year. GAP showed some turning points in some countries and regions (**Figure 4****,** Panel C). For example, in 2009, Haiti’s GAP was 7.64 years, while it was 2.15 years in 2010, a decline of 71.86%.

### Multiple linear regression analysis of factors associated with LE, HALE, and GAP

Fifteen factors were included in multiple linear regression analysis for these outcomes using the 1995-2012 data, respectively. Among them, three were statistically significant for LE (*P* < 0.05), Urbanization, TFR (the total fertility rate), and HFPerc (percentage of population with improved facilities). Four factors were statistically significant for HALE (*P* < 0.05), which were Urban, TFR, HFPerc, and incidence of chronic diseases. Only urbanization was statistically significant for GAP (*P* < 0.05).

### Projections of LE, HALE, and GAP in global regions, 2017-2025

Based on multiple linear regression analysis, we projected LE, HALE, and GAP for 2017 to 2025 in 149 countries and regions, which had the needed data (**Table 1**; see Table S2, S3, S4 in the **Online Supplementary Document** for the full projected results).

**Table 1:** Projected global and regional LE, HALE, and GAP, 2017 and 2025 (95% confidence interval)

	Life expectancy (LE) (years)		Healthy life expectancy (HALE) (years)		GAP (LE/HALE difference in years)	
	**2017**	**2025**	**2017**	**2025**	**2017**	**2025**
Afghanistan	58.30 (57.22-59.40)	61.21 (57.85-64.72)	49.79 (49.01-50.57)	52.16 (49.82-54.49)	8.49 (8.30-8.68)	8.75 (8.19-9.31)
Albania	77.59 (76.60-78.59)	79.20 (78.21-80.20)	67.77 (67.02-68.52)	69.11 (68.36-69.86)	9.93 (9.71-10.15)	10.30 (9.58-11.02)
Algeria	77.53 (77.31-77.74)	78.67 (77.33-80.01)	66.14 (65.95-66.33)	67.22 (66.23-68.20)	11.36 (11.30-11.42)	11.38 (10.91-11.84)
Andorra	82.52 (82.37-82.67)	82.51 (80.05-84.97)	71.20 (71.09-71.30)	71.21 (69.43-73.00)	11.32 (11.28-11.37)	11.28 (10.63-11.94)
Angola	65.30 (64.90-65.71)	69.79 (65.17-74.42)	56.92 (56.61-57.23)	61.94 (58.25-65.63)	8.42 (8.35-8.50)	9.01 (8.32-9.69)
Antigua and Barbuda	77.45 (77.11-77.79)	78.07 (77.05-79.08)	67.61 (67.35-67.86)	68.12 (67.36-68.88)	9.84 (9.75-9.93)	9.91 (9.65-10.17)
Argentina	76.88 (76.75-77.01)	78.26 (77.86-78.66)	67.37 (67.25-67.49)	68.54 (68.18-68.90)	9.54 (9.51-9.57)	9.77 (9.73-9.80)
Australia	82.37 (82.60-82.48)	82.20 (80.22-84.18)	71.53 (71.44-71.62)	71.47 (69.98-72.96)	10.96 (10.91-11.00)	10.73 (10.03-11.43)
Austria	81.66 (81.53-81.80)	82.66 (81.54-83.78)	71.04 (70.93-71.14)	71.84 (70.95-72.73)	10.63 (10.60-10.67)	10.83 (10.73-10.93)
Azerbaijan	72.45 (71.96-72.94)	76.03 (74.56-77.50)	63.72 (63.36-64.09)	66.40 (65.30-67.50)	8.70 (8.58-8.82)	9.15 (8.09-10.21)
Bahamas	73.94 (73.64-74.24)	74.68 (70.99-78.38)	64.96 (64.71-65.21)	65.69 (62.74-68.64)	8.97* (8.90-9.04)	8.91* (8.11-9.70)
Bahrain	77.00 (76.56-77.44)	78.66 (77.34-79.98)	65.55 (65.24-65.86)	66.86 (65.94-67.78)	11.43* (11.31-11.54)	11.42* (11.07-11.77)
Bangladesh	73.02 (72.66-73.38)	77.52 (73.32-81.73)	63.14 (62.90-63.37)	66.12 (63.30-68.94)	9.90 (9.81-9.99)	10.06 (9.66-10.47)
Barbados	76.55 (76.31-76.80)	76.01 (73.93-78.09)	66.87 (66.68-67.06)	66.50 (64.96-68.04)	9.67 (9.61-9.74)	9.43 (8.62-10.23)
Belarus	73.93 (73.25-74.61)	76.38 (69.65-83.11)	64.42 (63.90-64.94)	66.88 (62.33-71.44)	9.49* (9.31-9.67)	9.32* (7.01-11.63)
Belgium	81.05 (80.86-81.25)	82.02 (81.42-82.61)	70.25 (70.10-70.39)	71.25 (70.81-71.69)	10.79* (10.73-10.86)	10.73* (9.68-11.79)
Benin	64.91 (64.65-65.17)	68.85 (66.23-71.48)	56.34 (56.15-56.54)	59.74 (58.28-61.20)	8.52 (8.44-8.59)	8.87 (8.43-9.31)
Bhutan	74.06 (73.43-74.68)	76.10 (73.95-78.31)	63.40 (62.75-64.05)	64.48 (62.52-66.43)	10.77 (10.63-10.91)	11.01 (10.78-11.24)
Bolivia	73.47 (73.31-73.63)	75.32 (72.60-78.03)	64.27 (64.13-64.42)	67.01 (64.51-69.52)	9.26* (9.21-9.31)	9.19* (8.66-9.72)
Bosnia and Herzegovina	77.75 (76.07-79.43)	79.35 (51.02-107.7)	67.56 (66.14-68.97)	69.64 (45.74-93.53)	10.34 (10.03-10.65)	11.02 (10.08-11.96)
Botswana	65.97 (65.37-66.56)	64.51 (54.62-74.39)	57.14 (56.71-57.57)	55.61 (49.55-61.68)	8.81 (8.58-9.04)	10.23 (6.31-14.15)
Brazil	75.35 (75.26-75.44)	76.17 (74.68-77.66)	65.61 (65.51-65.70)	65.74 (64.18-67.31)	9.74* (9.68-9.80)	9.53* (8.78-10.28)
Bulgaria	75.16 (74.79-75.53)	76.94 (74.99-78.89)	65.76 (65.45-66.07)	67.01 (66.08-67.94)	9.39* (9.29-9.49)	9.07* (7.63-10.51)
Burundi	60.71 (60.13-61.30)	63.64 (56.71-70.56)	53.88 (53.36-54.40)	56.83 (50.64-63.03)	6.86 (6.76-6.96)	6.96 (5.97-7.94)
Cameroon	60.65 (60.37-60.93)	64.74 (60.34-69.15)	52.97 (52.73-53.21)	56.79 (53.08-60.51)	7.68 (7.63-7.74)	7.94 (7.03-8.86)
Canada	81.93 (81.84-82.02)	82.54 (81.03-84.05)	71.25 (71.18-71.32)	71.68 (70.56-72.81)	10.67 (10.62-10.72)	10.81 (10.13-11.50)
Chad	60.37 (59.96-60.78)	65.02 (60.87-69.17)	52.18 (51.85-52.52)	56.14 (52.74-59.54)	8.19 (8.10-8.28)	8.87 (8.10-9.63)
Chile	80.47 (80.21-80.72)	81.67 (79.91-83.43)	70.10 (69.90-70.29)	71.70 (71.11-72.29)	10.37* (10.27-10.47)	9.96* (8.67-11.25)
Colombia	78.55 (78.13-78.96)	80.81 (79.57-82.05)	69.34 (69.02-69.65)	71.20 (70.25-72.16)	9.26 (9.17-9.35)	9.64 (9.54-9.73)
China	76.71 (76.46-76.95)	79.29 (78.56-80.02)	68.13 (67.94-68.33)	70.21 (69.63-70.79)	8.55 (8.46-8.64)	8.82 (8.11-9.53)
Comoros	68.00 (67.29-68.71)	71.06 (69.70-72.42)	59.68 (59.09-60.26)	62.43 (61.30-63.55)	8.30 (8.16-8.45)	8.61 (8.32-8.90)
Congo gold	64.34 (61.37-67.32)	69.70 (66.63-72.77)	55.75 (53.31-58.19)	60.36 (57.84-62.88)	8.59 (8.04-9.14)	9.34 (8.77-9.91)
Costa Rica	81.08 (80.98-81.18)	81.84 (80.18-83.50)	71.28 (71.22-71.35)	72.26 (71.22-73.30)	9.81* (9.76-9.87)	9.75* (9.19-10.31)
Cote d’I voire	60.59 (60.34-60.84)	66.40 (63.31-69.49)	52.77 (52.56-52.98)	57.71 (55.14-60.28)	7.81 (7.69-7.93)	8.59 (7.50-9.68)
Croatia	77.37 (77.14-77.60)	77.45 (73.50-81.39)	67.48 (67.28-67.69)	68.03 (64.61-71.44)	9.97 (9.90-10.04)	10.10 (9.89-10.32)
Cuba	79.00 (78.93-79.06)	79.66 (78.54-80.79)	69.29 (69.23-69.34)	70.07 (69.12-71.03)	9.76 (9.72-9.79)	9.99 (9.87-10.10)
Cyprus	80.46 (80.10-80.82)	80.31 (76.15-84.48)	69.77 (69.52-70.03)	64.49 (63.72-65.25)	10.25 (10.16-10.34)	10.01 (8.95-11.07)
Czech Republic	79.25 (79.11-79.39)	80.42 (78.02-82.81)	68.3 (68.16-68.44)	69.84 (67.7-71.99)	11.07 (10.97-11.16)	11.61 (11.33-11.90)
Denmark	81.08 (80.91-81.26)	83.18 (82.64-83.73)	69.93 (69.80-70.07)	70.36 (68.32-72.40)	10.97 (10.92-11.02)	11.16 (10.61-11.70)
Djibouti	67.25 (66.88-80.91)	74.10 (70.43-82.64)	59.19 (58.88-59.50)	67.79 (64.64-70.95)	8.17 (8.10-8.23)	8.74 (8.32-9.16)
Timor-Leste	74.95 (71.04-78.86)	80.49 (76.44-84.54)	65.03 (61.82-68.24)	69.76 (66.43-73.08)	9.92 (9.21-10.63)	10.73 (9.99-11.47)
El Salvador	75.61 (75.28-75.95)	77.16 (75.46-78.85)	66.60 (66.22-66.98)	67.29 (66.14-68.43)	9.01 (8.93-9.09)	9.01 (8.60-9.41)
Equatorial Guinea	65.89 (64.84-66.94)	69.14 (55.85-82.44)	57.23 (56.39-58.07)	61.78 (55.88-67.69)	8.82 (8.64-9.00)	8.84 (6.51-11.17)
Eritrea	64.29 (60.08-68.50)	67.32 (62.96-71.68)	56.70 (53.13-60.28)	59.41 (55.71-63.11)	7.57 (6.95-8.19)	7.90 (7.27-8.52)
Estonia	77.79 (76.64-78.94)	78.62 (75.18-82.07)	67.92 (67.09-68.76)	70.89 (70.02-71.76)	10.46 (10.19-10.72)	10.68 (9.88-11.47)
Ethiopia	66.82 (65.54-68.11)	87.11 (82.28-91.93)	58.95 (57.84-60.06)	76.51 (72.96-80.66)	7.87 (7.69-8.06)	10.60 (9.91-11.29)
Fiji	65.38 (65.14-65.63)	65.44 (62.12-68.75)	57.21 (57.02-57.40)	57.28 (54.64-59.92)	8.17 (8.11-8.23)	8.17 (8.00-8.34)
Finland	82.02 (81.92-82.13)	84.42 (84.10-84.74)	70.76 (70.68-70.84)	72.85 (72.51-73.19)	11.25 (11.19-11.30)	11.53 (11.37-11.68)
France	82.49 (82.35-82.64)	83.53 (83.09-83.98)	71.81 (71.70-71.93)	72.49 (72.16-72.83)	10.65 (10.59-10.70)	10.73 (10.18-11.28)
Gabon	67.19 (66.60-67.77)	72.41 (67.37-77.45)	58.14 (57.69-58.60)	62.60 (58.46-66.74)	9.05 (8.91-9.19)	9.83 (8.91-10.76)
Gambia	67.68 (67.36-68.01)	70.85 (69.87-71.83)	58.58 (58.31-58.85)	61.45 (60.64-62.26)	9.10 (9.04-9.17)	9.44 (9.24-9.63)
Ghana	66.58 (66.19-66.97)	71.18 (67.54-74.82)	58.44 (58.12-58.76)	62.64 (59.66-65.62)	8.13 (8.05-8.22)	8.52 (7.68-9.37)
Greece	81.07 (80.94-81.20)	81.70 (81.31-82.08)	70.71 (70.61-70.81)	71.25 (70.95-71.55)	10.33* (10.29-10.37)	10.21* (9.71-10.71)
Guatemala	72.92 (72.69-73.15)	74.24 (70.39-78.09)	64.05 (63.89-64.20)	65.26 (64.78-65.73)	8.94* (8.87-9.01)	8.93* (8.56-9.31)
Guinea	61.15 (60.72-61.57)	65.29 (64.02-66.56)	53.11 (52.77-53.46)	56.18 (55.10-57.25)	7.79 (7.69-7.88)	8.18 (7.77-8.59)
Guyana	68.54 (68.15-68.93)	74.93 (69.13-80.74)	59.79 (59.47-60.10)	64.77 (60.55-68.98)	8.71 (8.62-8.79)	9.83 (8.40-11.25)
Honduras	73.23 (70.39-76.06)	75.24 (72.31-78.17)	64.75 (62.32-67.18)	66.48 (63.96-68.99)	8.48 (8.07-8.89)	8.76 (8.34-9.18)
Hungary	75.74 (75.62-75.87)	75.73 (73.62-77.84)	65.91 (65.77-66.05)	66.64 (64.42-68.85)	9.97 (9.85-10.08)	10.32 (9.98-10.67)
Iceland	82.30 (82.12-82.47)	82.51 (79.68-85.34)	71.51 (71.36-71.66)	71.73 (69.27-74.20)	10.79* (10.76-10.82)	10.77* (10.36-11.19)
India	68.93 (68.88-68.99)	71.95 (71.41-72.48)	59.31 (59.21-59.40)	63.48 (61.85-65.11)	9.73 (9.69-9.77)	10.34 (10.21-10.47)
Indonesia	71.94 (70.74-73.14)	74.20 (70.60-77.79)	63.16 (62.02-64.30)	64.80 (61.37-68.22)	8.78 (8.59-8.97)	9.36 (8.78-9.95)
Iraq	67.60 (66.19-69.00)	67.60 (63.38-71.82)	57.43 (56.31-58.55)	57.43 (54.06-60.80)	10.17 (9.87-10.47)	10.17 (9.26-11.07)
Ireland	81.17 (80.92-81.41)	81.35 (77.87-84.82)	70.47 (70.29-70.65)	70.63 (67.95-73.31)	10.70 (10.63-10.76)	10.71 (9.89-11.53)
Iran	76.34 (75.13-77.56)	80.05 (76.40-83.71)	64.99 (64.15-65.82)	66.79 (65.15-68.43)	11.23 (10.99-11.47)	11.68 (10.48-12.88)
Israel	82.29 (81.69-82.90)	83.27 (81.45-85.09)	71.64 (71.18-72.11)	73.28 (71.86-74.71)	10.72 (10.61-10.82)	10.77 (10.45-11.10)
Italy	82.45 (82.27-82.63)	82.61 (81.39-83.84)	71.80 (71.66-71.94)	71.81 (70.67-72.96)	10.61 (10.56-10.66)	10.72 (10.58-10.87)
Jamaica	74.96 (74.68-75.23)	75.78 (74.48-77.09)	65.42 (65.22-65.63)	66.24 (65.65-66.83)	9.57 (9.50-9.64)	9.69 (9.48-9.90)
Japan	84.08 (83.76-84.40)	84.93 (83.98-85.89)	73.25 (72.98-73.53)	73.79 (72.96-74.63)	10.84 (10.79-10.89)	11.25 (11.17-11.33)
Jordan	75.88 (75.49-76.26)	72.55 (66.07-79.03)	64.84 (64.55-65.12)	62.70 (57.94-67.45)	11.04 (10.93-11.16)	9.85 (7.91-11.80)
Kazakhstan	71.37 (71.04-71.69)	41.19 (22.08-60.29)	62.62 (62.39-62.85)	56.46 (50.68-62.23)	9.03 (8.92-9.14)	8.57 (6.70-10.44)
Kenya	67.38 (67.00-67.76)	71.70 (65.27-78.13)	59.03 (58.71-59.36)	62.59 (57.04-68.14)	8.35 (8.27-8.42)	9.11 (7.86-10.36)
Kiribati	62.02 (61.91-62.13)	63.89 (62.05-65.74)	54.21 (54.11-54.31)	55.74 (54.10-57.39)	7.81 (7.79-7.83)	8.15 (7.78-8.52)
Kuwait	79.61 (79.14-80.09)	78.96 (70.97-86.95)	68.24 (67.87-68.61)	68.22 (61.97-74.46)	11.38 (11.27-11.49)	10.74 (8.90-12.59)
Kyrgyzstan	71.42 (71.06-79.14)	72.90 (67.02-70.97)	62.65 (62.37-62.93)	64.20 (59.74-68.66)	8.77* (8.68-8.86)	8.70* (7.20-10.21)
Laos	67.73 (67.53-67.93)	71.59 (68.49-74.68)	59.52 (59.36-59.68)	63.04 (60.48-65.59)	8.24 (8.19-8.30)	8.84 (8.67-9.01)
Lebanon	80.19 (79.41-80.97)	80.47 (76.87-84.08)	68.29 (67.67-68.90)	68.61 (65.99-71.23)	11.84 (11.60-12.09)	12.28 (11.55-13.01)
Lesotho	51.11 (50.34-51.89)	57.55 (44.48-70.62)	44.45 (43.76-45.15)	49.52 (37.82-61.22)	6.66 (6.49-6.83)	8.03 (5.17-10.88)
Liberia	64.88 (62.50-67.27)	70.07 (67.59-72.55)	55.67 (53.76-57.58)	60.33 (58.34-62.31)	9.21 (8.73-9.69)	9.74 (9.24-10.24)
Lithuania	75.46 (74.36-76.56)	77.83 (74.53-81.13)	65.41 (64.74-66.07)	67.05 (65.05-69.04)	9.93* (9.67-10.19)	9.40* (8.62-10.17)
Luxembourg	82.20 (81.97-82.44)	82.89 (79.51-86.26)	71.04 (70.87-71.21)	71.69 (69.18-74.2)	11.16 (11.09-11.23)	11.18 (10.34-12.02)
Macedonia	74.97 (74.79-75.16)	76.10 (75.55-76.65)	65.68 (65.49-65.87)	66.31 (65.74-66.87)	9.28 (9.21-9.35)	9.72 (9.50-9.93)
Malawi	60.98 (60.58-61.39)	67.44 (60.66-74.21)	53.58 (53.27-53.89)	67.37 (62.16-72.57)	7.57 (7.47-7.67)	8.41 (6.94-9.88)
Malaysia	75.80 (75.72-75.88)	77.74 (76.40-79.08)	66.64 (66.57-66.72)	68.42 (67.18-69.66)	9.18 (9.14-9.21)	9.48 (9.19-9.77)
Malta	81.51 (81.32-81.71)	82.18 (78.86-85.50)	71.00 (70.85-71.16)	71.61 (68.94-74.27)	10.51 (10.46-10.56)	10.57 (9.75-11.39)
Mauritania	70.55 (70.30-70.81)	73.62 (72.87-74.38)	61.35 (61.15-61.54)	64.22 (63.64-64.81)	9.20 (9.16-9.25)	9.32 (9.19-9.46)
Mexico	76.41 (76.05-76.76)	76.16 (75.09-77.22)	67.24 (66.95-67.53)	68.67 (64.31-73.02)	9.33 (9.24-9.43)	9.48 (9.20-9.75)
Mongolia	68.24 (67.93-68.55)	69.21 (64.01-74.40)	60.10 (59.87-60.33)	61.64 (57.78-65.49)	8.14* (8.03-8.25)	7.57* (5.72-9.42)
Montenegro	77.14 (76.99-77.29)	77.98 (75.48-80.47)	67.24 (67.13-67.35)	68.16 (66.28-70.04)	9.91* (9.84-9.97)	9.88* (8.9-10.85)
Morocco	75.21 (75.11-75.30)	76.63 (75.85-77.41)	64.17 (64.08-64.26)	65.84 (65.10-66.58)	11.02* (10.98-11.07)	10.43* (9.93-10.93)
Mozambique	61.06 (60.58-61.55)	69.41 (63.78-75.04)	52.96 (52.47-53.45)	60.56 (52.30-68.82)	8.09 (7.98-8.20)	9.30 (7.73-10.86)
Namibia	65.41 (64.69-66.13)	73.46 (61.32-85.61)	57.02 (56.46-57.57)	63.60 (54.23-72.97)	8.37 (8.23-8.50)	8.86 (6.59-11.13)
New Zealand	81.98 (81.66-82.30)	83.20 (82.24-84.17)	70.82 (70.65-70.99)	70.98 (69.51-72.45)	10.93* (10.89-10.98)	10.88* (10.23-11.53)
Nicaragua	78.53 (77.30-79.76)	80.26 (78.40-82.13)	69.13 (68.09-70.17)	70.64 (69.07-72.21)	10.70 (10.65-10.75)	10.75 (10.59-10.91)
Niger	62.13 (61.93-62.34)	65.79 (63.35-68.22)	54.44 (54.27-54.60)	57.78 (55.67-59.88)	9.43 (9.24-9.62)	9.66 (9.46-9.85)
Nigeria	65.99 (65.57-66.42)	73.79 (68.53-79.04)	57.16 (56.81-57.51)	64.19 (59.82-68.55)	8.84 (8.75-8.92)	9.61 (8.55-10.66)
Norway	82.11 (81.95-82.27)	82.17 (79.47-84.87)	71.51 (71.39-71.64)	71.70 (69.66-73.73)	10.64 (10.59-10.69)	10.89 (10.74-11.05)
Pakistan	67.97 (67.21-68.73)	70.98 (67.77-74.19)	58.93 (58.28-59.59)	61.53 (58.74-64.31)	9.04 (8.92-9.15)	9.45 (8.93-9.97)
Panama	79.19 (79.06-79.32)	81.39 (79.25-83.53)	69.64 (69.52-69.76)	72.28 (70.28-74.29)	9.58 (9.53-9.62)	9.87 (9.31-10.43)
Papua New Guinea	61.13 (60.57-61.69)	63.91 (61.26-66.57)	53.40 (52.93-53.88)	55.69 (53.50-57.88)	7.73 (7.64-7.82)	8.22 (7.75-8.70)
Paraguay	74.59 (74.41-74.78)	75.62 (74.23-77.01)	65.06 (64.90-65.21)	65.92 (64.72-67.12)	9.52 (9.49-9.56)	9.56 (9.46-9.67)
Peru	79.92 (79.66-80.17)	81.34 (77.06-85.61)	70.10 (69.86-70.34)	72.14 (68.39-75.89)	9.96 (9.84-10.08)	10.47 (10.12-10.83)
Philippines	70.34 (70.12-70.56)	72.18 (70.22-74.13)	61.86 (61.66-62.05)	63.51 (61.68-65.34)	8.46 (8.40-8.52)	8.46 (8.29-8.63)
Poland	78.14 (77.81-78.47)	79.53 (78.55-80.51)	67.78 (67.48-68.08)	69.12 (68.23-70.01)	10.37 (10.24-10.50)	10.60 (10.21-11.00)
Portugal	81.12 (80.95-81.29)	81.74 (80.31-83.16)	70.63 (70.50-70.76)	71.40 (70.27-72.52)	10.49* (10.43-10.55)	10.41* (9.90-10.92)
Qatar	79.78 (79.52-80.03)	79.07 (74.77-83.37)	68.14 (67.95-68.33)	68.05 (64.90-71.19)	11.64 (11.56-11.71)	11.02 (9.74-12.31)
Republic of Congo	61.88 (60.79-62.98)	67.88 (65.10-70.65)	53.50 (52.61-54.38)	59.00 (56.54-61.46)	8.37 (8.17-8.57)	8.66 (8.45-8.86)
Republic of Guinea-Bissau	59.39 (58.72-60.06)	65.37 (63.37-67.36)	51.94 (51.35-52.53)	57.13 (55.35-58.90)	7.44 (7.34-7.55)	8.19 (7.87-8.50)
Republic of Korea	80.93 (80.78-81.07)	78.29 (75.83-80.75)	70.84 (70.69-71.00)	71.50 (68.94-74.06)	10.17 (10.05-10.29)	9.61 (8.24-10.97)
Romania	75.49 (74.87-76.11)	77.50 (75.63-79.37)	65.91 (65.64-66.17)	67.83 (67.16-68.50)	9.90 (9.73-10.07)	10.35 (10.18-10.53)
Rwanda	68.11 (67.02-69.19)	70.66 (59.63-81.68)	59.79 (58.86-60.72)	62.60 (53.19-72.01)	8.39 (8.14-8.64)	9.38 (8.63-10.13)
Saint Lucia	75.28 (74.84-75.73)	75.29 (73.95-76.64)	66.45 (66.17-66.73)	66.78 (65.94-67.62)	9.73* (9.63-9.83)	9.57* (8.48-10.65)
Saint Vincent	71.73 (71.34-72.11)	72.53 (71.38-73.69)	62.76 (62.44-63.08)	63.28 (62.33-64.24)	8.95 (8.88-9.02)	9.05 (8.84-9.25)
Sao Tome and Principe	70.89 (70.45-71.32)	73.20 (69.04-77.35)	61.91 (61.56-62.27)	64.03 (60.77-67.29)	8.97 (8.88-9.05)	9.13 (8.07-10.19)
Saudi Arabia	77.47 (77.22-77.72)	80.72 (78.67-82.76)	66.68 (66.47-66.88)	69.33 (67.77-70.88)	10.78 (10.71-10.85)	11.31 (10.45-12.17)
Senegal	66.64 (66.38-66.90)	69.94 (69.15-70.72)	58.30 (58.09-58.52)	61.86 (61.21-62.51)	8.38 (8.31-8.44)	8.60 (8.02-9.17)
Seychelles	73.80 (73.66-73.94)	74.77 (72.47-77.08)	65.08 (64.95-65.20)	66.10 (64.20-68.00)	8.77 (8.72-8.81)	9.07 (8.93-9.20)
Sierra Leone	57.87 (55.53-60.21)	61.10 (58.67-63.53)	50.55 (48.54-52.57)	53.56 (51.47-55.65)	7.32 (6.96-7.68)	7.54 (7.17-7.92)
Singapore	83.84 (83.62-84.05)	84.64 (81.44-87.84)	73.74 (73.59-73.90)	74.69 (73.02-76.36)	10.16 (10.08-10.25)	10.60 (10.33-10.86)
Slovak Republic	77.20 (76.94-77.46)	79.14 (78.36-79.91)	67.07 (66.88-67.27)	68.66 (68.07-69.25)	10.13 (10.03-10.22)	10.48 (10.19-10.76)
Slovenia	81.14 (80.87-81.41)	83.53 (82.71-84.34)	69.56 (69.36-69.75)	71.44 (70.85-72.04)	11.50* (11.40-11.59)	11.32* (10.29-12.35)
Solomon Islands	63.33 (63.05-63.60)	65.87 (62.66-69.07)	55.54 (55.30-55.77)	57.63 (54.87-60.38)	7.79 (7.74-7.84)	8.19 (7.70-8.68)
South Africa	63.04 (62.22-63.86)	67.89 (54.03-81.75)	54.31 (53.57-55.04)	58.11 (45.65-70.58)	8.74 (8.59-8.90)	9.84 (7.37-12.30)
Spain	83.16 (82.99-83.32)	84.51 (84.00-85.01)	83.16 (82.99-83.32)	84.51 (84.00-85.01)	83.16 (82.99-83.32)	84.51 (84.00-85.01)
Sri Lanka	77.40 (75.05-79.75)	79.61 (77.26-81.96)	67.56 (65.60-69.52)	69.42 (67.38-71.46)	9.66 (9.26-10.06)	10.01 (9.61-10.41)
Sudan	68.60 (68.19-69.01)	70.76 (69.52-72.02)	58.43 (58.07-58.78)	60.20 (59.13-61.28)	10.17 (10.10-10.24)	10.53 (10.31-10.75)
Suriname	71.56 (71.35-71.76)	73.32 (69.94-76.70)	62.43 (62.27-62.60)	63.94 (61.18-66.71)	9.13 (9.08-9.18)	9.41 (8.73-10.09)
Swaziland	58.77 (57.73-59.82)	69.44 (51.81-87.07)	50.60 (49.83-51.38)	54.76 (41.63-67.89)	8.15 (7.92-8.38)	10.82 (7.21-14.44)
Sweden	82.02 (81.88-82.17)	81.82 (79.51-84.14)	70.92 (70.83-71.01)	70.84 (69.81-71.87)	11.20 (11.16-11.24)	11.49 (11.36-11.61)
Switzerland	83.30 (83.11-83.49)	84.29 (82.96-85.61)	72.10 (71.95-72.25)	73.36 (72.92-73.8)	11.19* (11.12-11.26)	10.75* (9.67-11.83)
Tajikistan	72.41 (71.81-73.01)	75.79 (75.16-76.41)	63.70 (63.21-64.20)	66.61 (66.09-67.13)	8.76 (8.61-8.91)	9.26 (8.61-9.91)
Tanzania	65.13 (64.79-65.47)	71.83 (66.06-77.60)	57.26 (56.95-57.56)	61.82 (56.65-66.99)	7.86 (7.78-7.94)	8.64 (7.35-9.94)
Thailand	77.84 (77.51-78.18)	78.94 (75.49-82.38)	68.22 (67.98-68.46)	70.55 (69.84-71.25)	9.75* (9.67-9.83)	9.56* (8.61-10.51)
The Russian Federation	70.89 (69.73-72.06)	71.13 (52.52-89.74)	62.06 (61.11-63.01)	63.25 (47.87-78.63)	8.85* (8.59-9.10)	7.97* (4.31-11.63)
Togo	63.15 (62.61-63.68)	67.58 (62.03-73.13)	55.37 (54.92-55.82)	59.34 (54.6-64.08)	7.77 (7.66-7.88)	8.17 (7.40-8.93)
Tonga	70.39 (69.53-71.25)	71.12 (70.25-71.98)	61.22 (60.51-61.92)	61.74 (61.04-62.44)	9.17 (9.01-9.33)	9.38 (9.21-9.54)
Trinidad and Tobago	73.10 (72.79-73.41)	73.74 (70.01-77.47)	63.59 (63.35-63.82)	64.19 (61.28-67.1)	9.51 (9.44-9.59)	9.56 (8.71-10.41)
Tunisia	77.62 (77.51-77.73)	80.53 (78.69-82.36)	66.83 (66.70-66.96)	70.59 (68.44-72.75)	10.80* (10.76-10.84)	10.19* (9.47-10.91)
Turkey	79.22 (78.00-80.45)	78.66 (74.98-82.35)	67.67 (66.75-68.59)	67.39 (64.62-70.16)	11.54 (11.24-11.84)	11.15 (10.25-12.04)
Turkmenistan	70.58 (70.43-70.73)	73.25 (70.75-75.76)	62.37 (62.25-62.49)	64.88 (62.83-66.93)	8.20 (8.16-8.25)	8.37 (7.68-9.06)
Uganda	62.86 (62.51-63.22)	68.13 (62.89-73.37)	54.93 (54.64-55.23)	59.60 (55.29-63.92)	7.93 (7.84-8.03)	8.55 (8.27-8.84)
Ukraine	72.42 (71.42-73.43)	75.05 (72.04-78.06)	63.32 (62.52-64.13)	65.80 (63.39-68.22)	9.14 (8.88-9.40)	9.74 (8.97-10.52)
United Arab Emirates	75.59 (75.39-75.78)	76.16 (72.86-79.46)	65.21 (65.03-65.39)	66.21 (63.17-69.26)	10.39* (10.32-10.47)	10.09* (9.15-11.02)
United Kingdom	80.94 (80.80-81.08)	81.28 (79.23-83.32)	70.08 (70.00-70.17)	70.37 (69.05-71.69)	10.43 (10.36-10.50)	10.43 (10.23-10.64)
United States	78.84 (78.68-79.00)	78.70 (76.09-81.32)	67.68 (67.56-67.80)	67.55 (65.52-69.59)	11.16 (11.12-11.21)	11.14 (10.49-11.80)
Uruguay	77.46 (77.21-77.71)	78.31 (77.56-79.07)	68.03 (67.81-68.25)	68.64 (67.98-69.29)	9.43 (9.38-9.48)	9.71 (9.55-9.87)
US Virgin Islands	74.68 (74.54-74.81)	75.04 (74.63-75.44)	65.58 (65.47-65.70)	65.78 (65.43-66.13)	9.09 (9.06-9.13)	9.22 (9.12-9.31)
Uzbekistan	70.54 (70.34-70.73)	72.50 (69.20-75.80)	62.05 (61.90-62.20)	64.03 (61.49-66.57)	8.49* (8.43-8.54)	8.47* (7.52-9.42)
Vanuatu	64.04 (63.39-64.70)	65.56 (64.22-66.90)	56.05 (55.52-56.58)	57.23 (56.15-58.31)	7.93 (7.79-8.07)	8.15 (7.73-8.58)
Venezuela	75.87 (74.2-77.54)	76.88 (75.16-78.61)	66.90 (65.49-68.31)	67.71 (66.25-69.16)	8.97 (8.70-9.24)	9.17 (8.90-9.45)
Vietnam	74.79 (74.71-74.87)	76.67 (76.44-76.91)	66.13 (66.06-66.20)	67.87 (67.66-68.08)	8.66 (8.63-8.69)	8.83 (8.73-8.94)
Zambia	59.36 (58.76-59.96)	65.21 (56.08-74.34)	51.96 (51.46-52.45)	51.64 (43.25-60.03)	7.29 (7.15-7.43)	8.32 (6.58-10.07)

*These countries or regions will achieve the ideal state in the future, that is, LE increases, HALE increases and GAP decreases. For detailed results see Table S2, S3, S4 in the **Online Supplementary Document**.

**Figure 5:**
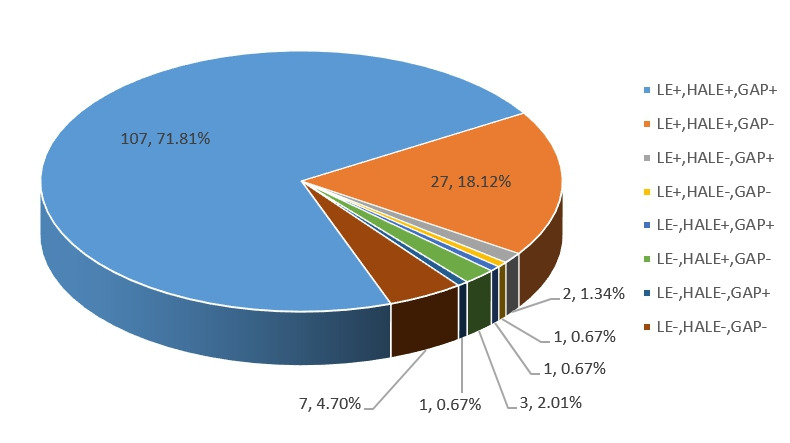
Eight scenarios of prediction of life expectancy (LE), healthy life expectancy (HALE) and their difference (GAP).

According to the projected trends, we divided the results into eight scenarios (**Figure 5**). The results showed that the largest proportion is 71.8%, indicating that the countries and regions with LE, HALE and GAP increase in the future still account for the majority. An ideal state of population health meant that the countries and regions should have higher LE, higher HALE, and lower GAP. The projected results showed that 27 (18.12%) countries and regions might reach this ideal state in the future. Some developed countries such as Switzerland would have slightly lower GAP along with higher LE and HALE, so did developing countries such as Belarus.

The most unsatisfactory state meant decreasing LE and HALE with increasing GAP. The projection suggested that 1 country might remain in this unsatisfactory state: Botswana. In Botswana, the projected LE would decrease by 1.46 years from 65.97 (95% confidence interval (CI) = 65.37-66.56) years in 2017 to 64.51 (95% CI = 54.62-74.39) years in 2025. Its HALE would decrease from 57.14 (95% CI = 56.71-57.57) to 55.61 (95% CI = 49.55-61.68) years and GAP increase from 8.81 (95% CI = 8.58-9.04) to 10.23 (95% CI = 6.31-14.15) years.

The projected results suggested that in Zambia, LE would continue to increase, while HALE would decrease, causing the continuous expansion of GAP in the future. LE in Zambia would increase from 59.36 (95% CI = 58.76-59.96) years in 2017 to 65.21 (95% CI = 56.08-74.34) years in 2025, HALE would decrease from 51.36 (95% CI = 51.46-52.45) to 51.64 (95% CI = 43.25-60.03) years, and GAP would increase by 1.03 (from 7.29 to 8.32) years. However, countries such as the United States, Australia, Cyprus, and Turkey would have lower LE, HALE, and GAP in the future. In United States, LE would decrease from 78.84 (95% CI = 78.68-79.00) years in 2017 to 78.80 (95% CI = 76.09-81.32) years in 2025, HALE would decrease from 67.68 (95% CI = 67.56-67.80) to 67.55 (95% CI = 65.52-69.59) years, and GAP would decrease by 0.02 years.

In general, in most countries/regions (about 72%), LE, HALE and GAP would maintain an increasing trend, while in some regions, the growth rate of HALE was greater than that of LE, ie, GAP would decrease.

## DISCUSSION

Our analysis showed that from 1995 to 2017, LE (95%), HALE (96%), and GAP (97%) in most of the 195 countries and regions showed an overall increasing trend. LE, HALE, and GAP would continue to increase in the future. Some countries and regions might achieve an ideal state. The increasing LE and HALE indicated that the health status had improved worldwide in the past and would continue to improve in the future, but there were some considerable disparities among countries, some countries/regions seem to be left behind these improvements, and more attention is needed for these countries in the future.

LE and HALE showed an increasing trend in 186 out of the 195 (95%) countries and regions, this was attributed to economic development that promotes improvements in medical services and the social environment. In most high-income and middle-high-income countries/regions, LE was above 65 years and HALE was above 55 years, the driving forces of higher LE and HALE in these countries/regions were mainly from national health policy reforms, welfare, and policies [12-16]. In low-income and low-middle-income countries, LE and HALE increased, but the growth was lower, especially the slower growth of HALE, which had led to further expansion of GAP. This might be due to improved coverage of infrastructure, security, and basic health services, and the promotion of basic health care for people, but the lack of further coverage of health facilities and technologies [17].

Both LE and HALE decreased in 4 countries and regions, Lesotho had the greatest decline. The main reasons for the reduction in these countries/regions were the outbreak of war and AIDS in these countries and regions. According to the United Nations’ estimates, one-quarter of people in Lesotho carry HIV. In addition, HALE in Paraguay and Northern Islands also decreased. The decrease in HALE means that the quality of life of the population has not improved, and medical claims, medical insurance systems, and welfare policies in the health field are inadequate. The increase in GAP meant that the growth rate of LE was greater than that of HALE, this was largely due to changes in the spectrum of diseases, the prolonged survival of the sick population, and the growing aging of society, which intensified the global burden of disease. GAP in 4 countries and regions decreased. These regions’ GAP decreased accompanied with LE and HALE increased during the last decade as medical advancements and breakthroughs in preventing HIV pediatric infections and mother-to-child transmission were widely implemented [18]. The absolute narrowing of the GAP did not mean that the health level of a country had reached the expected level. Only in the context of the common growth of LE and HALE, it could be meaningful to achieve the gap reduction.

Females’ LE, HALE, GAP, and RATE were all higher than males’ overall. Previous studies had shown that females tended to live longer and they had longer unhealthy survival time than males [19]. It is noteworthy that females paid more attention to their health than males; once females have symptoms of illness, they seek medical treatment more actively [20]. Compared with males, females gained a longer life expectancy free of major chronic diseases through adherence to a healthier lifestyle,such as no drinking and no smoking. While males bear more social stress and undertake more dangerous work. They tend to have unhealthier lifestyles, such as smoking and alcoholism.

Our analysis projected that 102 countries and regions (71%), LE, HALE and GAP would increase in the future. For example, Canada will achieve a higher LE and HALE along with a little higher GAP. Canada has enacted a series of funds to explore, prevent, and control morbidity and mortality and established an area-based social-economic measure that is confirmed to strongly correlate with improving potential life-years lost and life expectancy [21]. Japan is another example with such good outcomes, thank to its good medical service factors and other non-medical factors such as its education and legislation systems, culture, community-based activities, and diet [22]. Japan's medical affordability, medical quality, and community participation were satisfactory, while human resources and the easing of the medical environment were unsatisfactory, so there was an urgent need to increase the number of health workers and improve the medical environment to reduce the further expansion of the GAP [10].

The increase in Singapore's GAP contributed to the transition of disease burden from high-mortality diseases such as infections to chronic conditions. Studies have shown that factors such as diabetes and obesity make Singaporeans more susceptible to chronic diseases and affect their quality of life [23,24]. In the United States, LE, HALE, and GAP will decrease further. This may be attributable to a high incidence of gun violence, automobile accidents, drug abuse, and a high average BMI, while the incidence of chronic diseases such as diabetes and cardiovascular disease is increasing [25]. The health care needs of people suffering from chronic diseases have increased [26]. Insufficient investment in health care costs and unreasonable utilization of the infrastructure remain important challenges to improving the health of the worldwide population in the future [5,15].

Our study has some limitations. First, our analysis could not incorporate factors such as education and specific chronic diseases such as cardiovascular disease, hypertension, tobacco and alcohol use due to lack of data. The factors could affect LE, HALE, and GAP as well. Second, our projection model is an exploratory simulation of the trends of LE, HALE, and GAP. It is better suited for short-term predictions. The long-term predictions for the future would be affected by the uncertainties of society, medical technology development, health care system, and other unexpected events.

Prolonging LE and HALE can be achieved by improving economic development and investment in medical services. However, the prolongation of life does not mean improved quality of life. While striving to improve LE and HALE, we should pay more attention to reducing GAP to improve quality of life. Whether it is the current state or the future forecast trend, most countries’ LE, HALE, and GAP are increasing. Governments should provide universal free access to high-quality health care services. They should also provide accessible public health services. Underdeveloped countries and regions should improve the health infrastructure construction and increase health workforce input. As more developed regions continue to increase and improve sanitation facilities, they should formulate more effecting disease prevention and control systems and post-illness care protection systems for communities and families [27]. Some findings suggest that promotion of a healthy lifestyle would help to reduce the health care burdens through lowering the risk of developing multiple chronic diseases, including cancer, cardiovascular disease, and diabetes, and extending disease-free life expectancy [28,29].

People should pay more attention to changing for healthy lifestyle, reasonable diet, moderate exercise, quiting smoking, limiting alcohol intake, and good mental health. The impact of aging and increasing chronic diseases on the extension of the life span is increasing. Countries with the highest life expectancy need to assist their elderly people to spend years in later life with good health. In some countries, LE and HALE might show a decreasing trend in the future. They need to take actions to prevent this. In the future, while striving to improve LE and HALE, more efforts should be made to reduce GAP to improve quality of life.

## CONCLUSIONS

Public health as indicated by LE, HALE, and GAP has improved in most countries and regions in the world since 1995, and it likely will continue to improve in the future. Whether the years of life gained are spent in good or poor health and how to narrow the GAP is an important global health issue [30,31]. Research shows that considerable amount of years of life lost are due to tobacco use, unhealthy diet, alcohol consumption, and lack of physical activity. Large gains in HALE and decreasing of GAP will only be achieved by changing such risk factors. Public policies and programs are needed to empower people’ to have healthy lifestyles and take other actions needed to maintain good health.

## Additional material

Online Supplementary Document
