# Supplementary material for: A comparative, correlate analysis and projection of global and regional life expectancy, healthy life expectancy, and their GAP: 1995-2025
Source: J Glob Health. 2020 Sep 11;10(2):020407. doi: 10.7189/jogh.10.020407 (PMC7568920; doi:10.7189/jogh.10.020407)
Supplement: Online Supplementary Document [file jogh-10-020407-s001.pdf]

Table S1 Global and regional Life Expectancy, Healthy Life Expectancy, GAP and influencing factors

Country	Year	LE	HALE	GAP	GDPPC	HEPC	HE /GDP	OOP /PHE	OOP /HE	PHE /HE	PHE /GDP	PriHE /GDP	Urban	ODR	TFR	HFPerc	Wperc	GGE	ICD
Afghanistan	1995	51.05	43.28	7.77	-	-	-	-	-	-	-	-	19.76	4.67	7.61	20.60	21.50	11635.66	90877.27
	1996	51.38	43.60	7.78	-	-	-	-	-	-	-	-	20.06	4.68	7.63	21.10	23.30	12505.93	90857.47
	1997	51.11	43.41	7.69	-	-	-	-	-	-	-	-	20.36	4.67	7.64	21.70	25.00	13343.25	90842.43
	1998	50.08	42.60	7.48	-	-	-	-	-	-	-	-	20.66	4.65	7.62	22.20	26.80	13793.37	90825.92
	1999	51.52	43.79	7.73	-	-	-	-	-	-	-	-	20.97	4.61	7.57	22.80	28.50	14812.37	90838.68
	2000	51.61	43.87	7.74	-	-	-	-	-	-	-	-	21.28	4.57	7.49	23.40	30.30	13530.95	90873.48
	2001	51.66	43.99	7.68	-	-	-	-	-	-	-	-	21.60	4.52	7.39	23.90	32.00	12585.40	90864.12
	2002	52.41	44.64	7.77	364.57	15.48	7.76	99.53	92.47	7.09	0.55	7.21	21.92	4.47	7.27	24.50	33.80	13842.74	90873.14
	2003	53.01	45.16	7.85	376.76	19.33	8.82	99.55	92.69	6.90	0.61	8.21	22.24	4.43	7.14	25.10	35.50	14709.88	90871.68
	2004	53.48	45.58	7.90	364.10	20.89	8.79	99.54	88.56	11.03	0.97	7.82	22.56	4.39	6.99	25.70	37.30	14654.37	90866.08
	2005	53.85	45.91	7.94	389.42	21.90	8.07	99.52	89.63	9.94	0.80	7.27	22.90	4.36	6.83	26.30	39.10	14770.26	90857.52
	2006	54.12	46.17	7.95	397.99	22.18	7.43	99.45	85.23	14.29	1.06	6.37	23.24	4.42	6.65	26.90	40.80	14849.16	90803.99
	2007	54.54	46.53	8.01	440.37	26.99	6.73	99.49	7.76	7.76	0.52	6.21	23.59	4.47	6.46	27.40	42.60	15272.63	90764.35
	2008	55.18	47.07	8.11	444.95	33.81	8.33	99.71	83.12	16.64	1.39	6.94	23.95	4.52	6.25	28.00	44.40	15288.78	90751.46
	2009	55.67	47.47	8.19	524.82	43.53	9.42	99.68	72.31	27.46	2.59	6.83	24.31	4.57	6.04	28.70	46.20	15554.43	90753.39
	2010	56.16	47.91	8.25	553.30	52.89	9.20	99.63	68.70	31.04	2.86	6.34	24.69	4.62	5.82	29.30	48.00	17791.33	90766.92
	2011	56.67	48.33	8.34	569.23	52.38	7.87	99.56	73.26	26.42	2.08	5.79	25.07	4.65	5.60	29.90	49.80	17981.31	90803.94
	2012	57.19	48.78	8.42	630.43	58.17	8.52	99.56	65.43	34.28	2.92	5.60	25.47	4.67	5.38	30.50	51.60	-	90836.29
	2013	57.67	49.18	8.49	621.82	53.37	8.13	99.56	67.13	32.58	2.65	5.48	25.87	4.70	5.17	31.10	53.40	-	90855.94
	2014	57.78	49.30	8.49	610.24	56.57	8.18	99.56	63.88	35.84	2.93	5.25	26.28	4.71	4.98	31.80	55.20	-	90873.96
	2015	57.84	49.36	8.48	599.14	-	-	-	-	-	-	-	26.70	4.71	4.80	31.90	55.30	-	90872.57
	2016	57.95	49.49	8.46	596.26	-	-	-	-	-	-	-	27.13	4.74	-	-	-	-	90859.25
	2017	63.38	53.04	10.34	-	-	-	-	-	-	-	-	-	-	-	-	-	-	-
Albania	1995	74.05	64.73	9.32	1764.70	51.48	6.60	100.00	74.03	25.97	1.71	4.88	38.91	10.58	2.59	79.40	95.70	6421.57	90002.40

	1996	74.21	64.87	9.34	1937.29	72.43	7.43	99.93	76.45	23.50	1.75	5.69	39.47	10.73	2.51	80.20	95.70	6187.38	90109.07
	1997	72.33	63.33	9.00	1738.17	52.94	7.57	99.91	73.18	26.75	2.02	5.54	40.04	10.87	2.42	81.00	95.80	5500.61	90231.93
	1998	74.20	64.90	9.29	1906.72	61.25	6.94	99.91	69.76	30.17	2.10	4.85	40.60	11.00	2.33	81.80	95.80	5246.13	90371.76
	1999	74.23	64.95	9.29	2177.90	75.95	6.87	99.89	63.38	36.55	2.51	4.36	41.17	11.14	2.25	82.60	95.90	6493.49	90530.44
	2000	74.26	65.02	9.24	2337.94	74.03	6.26	99.87	63.82	36.10	2.26	4.00	41.74	11.29	2.16	83.40	96.10	8389.28	90691.08
	2001	74.31	65.06	9.24	2547.38	78.67	6.00	99.82	61.69	38.20	2.29	3.71	42.44	11.63	2.07	84.20	96.20	6820.62	90806.46
	2002	74.35	65.12	9.23	2663.14	89.92	6.30	99.83	64.07	35.81	2.26	4.04	43.50	11.98	1.98	85.00	96.10	7503.06	90942.93
	2003	74.43	65.20	9.22	2827.46	112.63	6.27	94.34	60.44	35.94	2.25	4.01	44.57	12.35	1.90	85.80	96.10	7834.02	91074.94
	2004	74.57	65.33	9.23	3001.41	153.26	6.38	92.78	53.81	42.00	2.68	3.70	45.65	12.73	1.82	86.60	96.10	7401.10	91207.09
	2005	74.77	65.55	9.21	3189.40	166.23	6.12	93.81	52.30	44.25	2.71	3.41	46.73	13.11	1.75	87.40	96.00	8308.39	91319.66
	2006	75.02	65.76	9.26	3383.89	175.46	5.86	92.43	51.82	43.93	2.58	3.29	47.82	13.62	1.70	88.10	96.00	8027.15	91399.16
	2007	75.27	65.89	9.38	3610.73	216.94	6.10	92.21	43.07	43.07	2.63	3.47	48.90	14.17	1.67	88.90	95.90	9879.39	91469.24
	2008	75.44	66.03	9.42	3775.38	254.74	5.87	95.50	51.67	45.90	2.69	3.18	49.99	14.74	1.65	89.60	95.80	8136.79	91546.70
	2009	75.75	66.26	9.49	3928.34	238.07	5.79	99.69	50.91	48.93	2.83	2.96	51.08	15.34	1.65	90.20	95.70	8108.81	91629.83
	2010	76.18	66.67	9.51	4094.36	219.27	5.34	99.68	53.41	46.42	2.48	2.86	52.16	15.96	1.65	90.90	95.60	8507.00	91718.06
	2011	76.56	66.87	9.68	4210.08	255.26	5.71	99.69	49.99	49.86	2.85	2.87	53.25	16.37	1.67	91.50	95.50	8712.30	91774.51
	2012	76.85	67.09	9.75	4276.92	239.51	5.59	99.69	50.89	48.95	2.74	2.85	54.33	16.76	1.69	92.10	95.40	8898.64	91815.14
	2013	77.07	67.27	9.80	4327.64	253.34	5.66	99.69	49.70	50.15	2.84	2.82	55.38	17.15	1.70	92.70	95.20	-	91847.27
	2014	77.27	67.43	9.84	4413.56	272.20	5.88	99.69	49.93	49.91	2.94	2.95	56.41	17.56	1.71	93.20	95.10	-	91881.87
	2015	77.44	67.55	9.89	4524.99	-	-	-	-	-	-	-	57.41	18.05	1.71	93.20	95.10	-	91908.52
	2016	77.59	67.77	9.83	4684.97	-	-	-	-	-	-	-	58.38	18.48	-	-	-	-	91912.23
	2017	78.23	67.49	10.74	-	-	-	-	-	-	-	-	-	-	-	-	-	-	-
Algeria	1995	72.28	61.74	10.54	3223.56	53.87	3.70	97.04	26.95	72.23	2.67	1.03	56.00	6.45	3.45	81.70	91.20	123799.18	87788.58
	1996	72.77	62.17	10.60	3297.86	53.45	3.35	96.49	24.40	74.71	2.50	0.85	56.77	6.55	3.21	82.10	90.90	123728.79	87821.24
	1997	72.93	62.33	10.59	3281.10	57.94	3.59	96.72	28.02	71.03	2.55	1.04	57.54	6.67	2.99	82.50	90.50	123971.39	87868.29
	1998	73.09	62.49	10.60	3397.41	57.09	3.59	97.12	28.81	70.34	2.53	1.07	58.31	6.79	2.80	82.90	90.20	126561.25	87918.71
	1999	73.52	62.86	10.66	3457.14	54.29	3.43	97.00	29.68	69.41	2.38	1.05	59.12	6.93	2.64	83.30	89.90	132794.42	87980.34
	2000	73.85	63.15	10.70	3541.07	61.30	3.49	96.72	25.79	73.33	2.56	0.93	59.92	7.06	2.51	83.60	89.50	133778.20	88059.50
	2001	74.10	63.36	10.74	3600.44	66.51	3.84	96.01	21.67	77.43	2.97	0.87	60.71	7.15	2.44	84.00	89.20	129334.83	88128.42

	2002	74.42	63.63	10.79	3754.52	66.18	3.73	95.72	22.52	76.47	2.85	0.88	61.50	7.23	2.40	84.30	88.80	131222.65	88191.02
	2003	74.53	63.72	10.81	3974.18	75.44	3.60	95.34	21.15	77.82	2.80	0.80	62.28	7.31	2.41	84.70	88.50	139592.33	88301.96
	2004	75.04	64.13	10.91	4091.14	92.15	3.54	94.58	26.09	72.41	2.57	0.98	63.06	7.40	2.44	85.00	88.10	139846.68	88424.68
	2005	75.35	64.41	10.94	4273.31	100.36	3.24	95.18	28.09	70.48	2.28	0.95	63.83	7.49	2.50	85.30	87.70	146241.50	88564.48
	2006	75.62	64.63	10.99	4282.33	116.34	3.36	95.76	29.12	69.59	2.33	1.02	64.59	7.59	2.58	85.60	87.40	151655.37	88668.38
	2007	75.86	64.77	11.09	4359.38	150.55	3.82	96.19	70.80	70.80	2.71	1.12	65.35	7.70	2.66	85.80	87.00	157333.43	88786.05
	2008	76.11	64.96	11.16	4390.50	206.41	4.20	96.45	26.15	72.89	3.06	1.14	66.10	7.82	2.75	86.10	86.60	160415.28	88916.01
	2009	76.31	65.10	11.21	4386.04	207.72	5.36	96.69	27.45	71.61	3.84	1.52	66.82	7.96	2.83	86.40	86.20	160849.93	89036.78
	2010	76.54	65.32	11.23	4463.39	228.92	5.12	96.98	28.88	70.22	3.59	1.52	67.53	8.12	2.89	86.60	85.70	167674.63	89181.14
	2011	76.70	65.41	11.29	4504.92	286.97	5.29	97.23	27.87	71.34	3.78	1.52	68.21	8.23	2.93	86.80	85.30	172303.51	89278.94
	2012	76.84	65.54	11.30	4564.44	335.27	6.14	97.28	25.70	73.58	4.52	1.62	68.87	8.34	2.94	87.00	84.90	176471.23	89391.81
	2013	76.97	65.66	11.32	4596.22	345.65	7.12	97.28	26.44	72.83	5.19	1.94	69.51	8.49	2.92	87.20	84.50	-	89491.59
	2014	77.09	65.75	11.34	4675.89	361.73	7.21	97.28	26.50	72.76	5.24	1.96	70.13	8.69	2.89	87.40	84.00	-	89603.76
	2015	77.27	65.90	11.37	4759.60	-	-	-	-	-	-	-	70.73	8.96	2.84	87.60	83.60	-	89712.75
	2016	77.41	66.07	11.34	4827.72	-	-	-	-	-	-	-	71.30	9.27	-	-	-	-	89826.16
	2017	77.74	66.48	11.25	-	-	-	-	-	-	-	-	-	-	-	-	-	-	-
American	1995	70.75	61.42	9.32	-	-	-	-	-	-	-	-	85.28	-	-	61.10	95.70	22.88	83944.90
	1996	70.79	61.45	9.34	-	-	-	-	-	-	-	-	86.04	-	-	61.20	96.10	23.26	84029.44
	1997	70.86	61.49	9.37	-	-	-	-	-	-	-	-	86.77	-	-	61.30	96.50	23.62	84124.08
	1998	71.04	61.61	9.43	-	-	-	-	-	-	-	-	87.47	-	-	61.40	96.90	23.93	84212.12
	1999	71.29	61.77	9.52	-	-	-	-	-	-	-	-	88.13	-	-	61.50	97.40	31.14	84300.27
	2000	71.53	61.94	9.59	-	-	-	-	-	-	-	-	88.59	-	-	61.50	97.80	47.66	84419.78
	2001	71.61	62.01	9.61	-	-	-	-	-	-	-	-	88.49	-	-	61.60	98.20	50.88	84519.33
	2002	71.61	62.01	9.59	10613.91	-	-	-	-	-	-	-	88.39	-	-	61.70	98.60	51.35	84627.16
	2003	71.34	61.80	9.53	10630.45	-	-	-	-	-	-	-	88.30	-	-	61.80	99.00	51.84	84749.89
	2004	71.52	61.96	9.56	10661.17	-	-	-	-	-	-	-	88.20	-	-	61.80	99.40	52.32	84885.73
	2005	71.63	62.05	9.57	10644.58	-	-	-	-	-	-	-	88.10	-	-	61.90	99.90	52.80	85041.40
	2006	71.73	62.15	9.58	10282.46	-	-	-	-	-	-	-	88.00	-	-	62.00	100.00	53.30	85144.85
	2007	71.90	62.27	9.64	10619.61	-	-	-	-	-	-	-	87.90	-	-	62.10	100.00	53.91	85249.63

	2008	72.19	62.50	9.70	10500.39	-	-	-	-	-	-	-	87.80	-	-	62.10	100.00	54.62	85376.09
	2009	70.89	61.43	9.46	10199.06	-	-	-	-	-	-	-	87.70	-	-	62.20	100.00	54.99	85498.04
	2010	72.36	62.66	9.71	10352.82	-	-	-	-	-	-	-	87.59	-	-	62.30	100.00	56.64	85615.61
	2011	72.19	62.51	9.68	10442.73	-	-	-	-	-	-	-	87.50	-	-	62.40	100.00	57.74	85773.99
	2012	72.21	62.52	9.69	10000.31	-	-	-	-	-	-	-	87.41	-	-	62.50	100.00	58.77	85941.26
	2013	72.15	62.46	9.69	9711.11	-	-	-	-	-	-	-	87.33	-	-	62.50	100.00	-	86098.58
	2014	72.21	62.50	9.71	9779.88	-	-	-	-	-	-	-	87.26	-	-	62.50	100.00	-	86255.90
	2015	72.25	62.52	9.73	9884.11	-	-	-	-	-	-	-	87.20	-	-	62.50	100.00	-	86419.14
	2016	72.39	62.64	9.75	9614.47	-	-	-	-	-	-	-	87.15	-	-	-	-	-	86586.95
	2017	71.83	62.12	9.71	-	-	-	-	-	-	-	-	-	-	-	-	-	-	-
Andorra	1995	80.45	69.48	10.97	32917.65	1282.52	5.81	71.41	22.44	68.57	3.98	1.83	93.66	-	-	100.00	100.00	-	92570.86
	1996	80.78	69.75	11.03	34175.26	1386.66	6.09	69.58	21.22	69.50	4.24	1.86	93.43	-	-	100.00	100.00	-	92546.96
	1997	81.11	70.02	11.09	37293.28	1395.52	6.14	69.71	20.96	69.93	4.30	1.85	93.18	-	-	100.00	100.00	-	92558.30
	1998	81.41	70.26	11.15	38595.72	1831.21	7.76	63.33	16.48	73.98	5.74	2.02	92.93	-	-	100.00	100.00	-	92602.84
	1999	81.69	70.48	11.21	40035.48	1369.16	5.73	71.11	22.28	68.67	3.93	1.79	92.67	-	-	100.00	100.00	-	92648.10
	2000	81.94	70.67	11.27	40801.54	1242.21	5.78	70.74	22.08	68.78	3.98	1.80	92.40	-	-	100.00	100.00	-	92700.31
	2001	82.19	70.87	11.32	41420.85	1268.63	5.79	70.00	21.88	68.75	3.98	1.81	92.06	-	-	100.00	100.00	-	92739.82
	2002	82.43	71.06	11.37	42396.30	1450.80	5.97	70.59	22.02	68.81	4.11	1.86	91.64	-	-	100.00	100.00	-	92794.84
	2003	82.51	71.12	11.38	45519.49	1827.84	5.75	71.36	22.34	68.69	3.95	1.80	91.21	-	-	100.00	100.00	-	92897.97
	2004	82.43	71.08	11.35	47032.87	2127.74	5.70	72.20	22.63	68.66	3.92	1.79	90.75	-	-	100.00	100.00	-	93008.42
	2005	82.54	71.16	11.38	48831.93	2089.67	5.22	72.84	24.59	66.24	3.46	1.76	90.30	-	-	100.00	100.00	-	93076.79
	2006	82.63	71.23	11.40	49708.40	2256.10	5.31	72.98	24.00	67.11	3.57	1.75	89.84	-	1.24	100.00	100.00	-	93125.90
	2007	82.71	71.29	11.42	48710.66	2997.57	6.34	72.56	72.39	72.39	4.59	1.75	89.36	-	1.18	100.00	100.00	-	93168.05
	2008	82.74	71.31	11.42	43900.95	3081.87	6.57	72.56	19.25	73.47	4.83	1.74	88.87	-	1.25	100.00	100.00	-	93201.55
	2009	82.69	71.28	11.41	41979.87	2897.93	6.76	72.56	18.49	74.52	5.04	1.72	88.35	-	1.19	100.00	100.00	-	93221.81
	2010	82.69	71.27	11.43	39736.35	3173.99	8.00	72.56	15.94	78.03	6.24	1.76	87.82	-	1.27	100.00	100.00	-	93244.73
	2011	82.64	71.24	11.39	38205.77	2794.50	6.70	72.56	19.24	73.49	4.93	1.78	87.26	-	-	100.00	100.00	-	93265.38
	2012	82.59	71.22	11.37	38190.59	2993.73	7.55	72.56	17.31	76.15	5.75	1.80	86.71	-	-	100.00	100.00	-	93301.35
	2013	82.58	71.22	11.36	39104.30	4914.39	11.48	72.56	11.29	84.44	9.69	1.79	86.17	-	-	100.00	100.00	-	93344.08

	2014	82.55	71.21	11.34	40785.05	3746.33	8.13	72.56	15.94	78.03	6.34	1.79	85.63	-	-	100.00	100.00	-	93391.66
	2015	82.52	71.19	11.33	41765.92	-	-	-	-	-	-	-	85.12	-	-	100.00	100.00	-	93445.46
	2016	82.52	71.20	11.33	42681.60	-	-	-	-	-	-	-	84.61	-	-	-	-	-	93491.76
	2017	82.70	71.20	11.51	-	-	-	-	-	-	-	-	-	-	-	-	-	-	-
Angola	1995	51.95	45.24	6.71	1775.90	18.01	6.46	72.69	20.26	72.14	4.66	1.80	28.88	5.01	6.92	25.50	46.10	48230.30	89975.49
	1996	52.29	45.56	6.74	1919.21	13.76	2.81	55.17	21.07	61.81	1.74	1.07	29.57	5.01	6.85	26.80	45.90	47136.22	89942.36
	1997	52.67	45.88	6.79	2015.01	19.28	3.48	66.72	28.04	57.98	2.02	1.46	30.27	4.99	6.79	28.10	45.80	33091.22	89915.66
	1998	52.80	46.00	6.80	2094.48	15.14	3.34	74.33	42.81	42.41	1.41	1.92	30.98	4.96	6.73	29.40	45.70	35275.13	89885.72
	1999	52.89	46.07	6.81	2101.95	14.23	3.38	61.15	22.56	63.11	2.13	1.25	31.69	4.91	6.68	30.80	45.70	36119.43	89875.41
	2000	53.32	46.45	6.87	2100.57	16.90	2.79	56.55	22.52	60.17	1.68	1.11	32.42	4.85	6.64	32.10	45.70	34780.68	89869.02
	2001	53.60	46.71	6.89	2119.33	30.92	5.38	80.50	38.45	52.23	2.81	2.57	33.15	4.84	6.60	33.50	45.70	34371.84	89835.24
	2002	54.43	47.43	7.00	1906.09	28.19	3.63	74.68	40.05	46.37	1.69	1.95	33.90	4.83	6.57	34.80	45.70	34490.37	89801.96
	2003	55.03	47.97	7.06	1936.60	37.45	4.41	78.87	42.27	46.41	2.04	2.36	34.65	4.80	6.54	36.20	45.80	35858.02	89771.93
	2004	55.66	48.52	7.14	2071.91	53.46	4.71	78.34	38.27	51.15	2.41	2.30	35.41	4.77	6.50	37.60	45.90	36860.23	89758.30
	2005	56.40	49.19	7.21	2364.19	64.69	4.10	76.19	38.60	49.33	2.02	2.08	36.17	4.72	6.47	39.00	46.00	35855.74	89733.88
	2006	56.89	49.63	7.27	2754.41	102.38	4.54	67.91	22.72	66.54	3.02	1.52	36.94	4.72	6.42	40.40	46.20	36375.82	89664.32
	2007	57.79	50.36	7.43	3258.48	106.60	3.38	77.95	65.44	65.44	2.21	1.17	37.72	4.71	6.37	41.80	46.40	36690.53	89590.79
	2008	58.53	50.99	7.54	3578.87	163.12	3.84	67.06	19.68	70.65	2.72	1.13	38.51	4.69	6.31	43.30	46.60	37396.29	89527.00
	2009	59.24	51.59	7.65	3536.80	160.84	4.37	75.38	29.45	60.93	2.66	1.71	39.30	4.66	6.24	44.70	46.90	38309.47	89470.52
	2010	60.04	52.30	7.74	3529.05	131.80	3.39	70.77	27.75	60.78	2.06	1.33	40.10	4.61	6.16	46.20	47.10	40029.39	89420.91
	2011	60.87	52.97	7.89	3538.72	160.36	3.38	68.98	24.99	63.77	2.16	1.22	40.90	4.59	6.08	47.60	47.50	40878.65	89348.10
	2012	61.68	53.69	7.99	3591.03	168.03	3.30	67.87	23.98	64.67	2.14	1.17	41.70	4.55	6.00	49.10	47.80	41657.16	89283.39
	2013	62.47	54.37	8.10	3702.60	225.74	4.26	67.00	18.59	72.26	3.08	1.18	42.49	4.52	5.92	50.60	48.20	-	89219.73
	2014	63.27	55.06	8.21	3747.57	179.36	3.31	67.04	23.96	64.26	2.12	1.18	43.27	4.52	5.84	51.10	48.60	-	89176.01
	2015	63.98	55.67	8.31	3730.17	-	-	-	-	-	-	-	44.05	4.56	5.77	51.60	49.00	-	89149.01
	2016	64.64	56.29	8.34	3582.65	-	-	-	-	-	-	-	44.82	4.63	-	-	-	-	89109.65
	2017	64.19	55.01	9.18	-	-	-	-	-	-	-	-	-	-	-	-	-	-	-
Antigua	1995	74.44	65.19	9.25	10440.71	353.19	4.14	86.45	29.15	66.28	2.75	1.40	33.96	12.58	2.21	79.60	97.60	391.94	91344.82
	1996	74.44	65.20	9.24	10834.63	356.41	3.92	86.07	31.07	63.91	2.51	1.42	33.59	12.13	2.25	80.60	97.60	390.01	91383.53

	1997	74.82	65.50	9.33	11117.09	373.27	3.94	86.70	29.53	65.94	2.60	1.34	33.22	11.64	2.28	81.60	97.60	362.38	91418.31
	1998	75.03	65.66	9.37	11335.13	386.63	3.92	87.98	28.01	68.16	2.67	1.25	32.86	11.16	2.30	82.60	97.60	379.42	91437.16
	1999	74.97	65.62	9.35	11470.99	400.74	3.96	87.04	26.74	69.28	2.74	1.22	32.49	10.71	2.32	83.60	97.70	404.10	91454.96
	2000	74.83	65.51	9.32	11981.92	416.41	4.13	81.27	26.35	67.58	2.79	1.34	32.13	10.30	2.32	84.60	97.70	398.69	91475.06
	2001	75.17	65.76	9.40	11191.10	439.38	4.48	81.10	27.75	65.78	2.95	1.53	31.74	10.17	2.31	85.50	97.70	399.27	91488.11
	2002	75.40	65.94	9.46	11147.26	442.61	4.41	81.10	26.89	66.84	2.95	1.46	31.10	10.08	2.29	86.50	97.70	406.43	91506.26
	2003	75.60	66.09	9.50	11683.46	470.78	4.53	81.10	28.34	65.06	2.95	1.58	30.47	10.08	2.27	87.50	97.80	418.84	91519.08
	2004	75.69	66.16	9.53	12218.74	462.49	4.21	81.10	29.44	63.70	2.68	1.53	29.84	10.18	2.25	88.50	97.80	449.00	91537.94
	2005	75.96	66.38	9.58	12857.16	532.73	4.41	78.48	25.98	66.89	2.95	1.46	29.22	10.37	2.22	89.50	97.80	454.07	91558.80
	2006	76.07	66.48	9.59	14325.48	590.24	4.34	76.64	24.55	67.97	2.95	1.39	28.61	10.33	2.20	90.40	97.80	463.57	91600.53
	2007	76.46	66.77	9.69	15466.95	652.25	4.27	74.71	69.09	69.09	2.95	1.32	28.00	10.30	2.18	91.40	97.90	478.08	91655.55
	2008	76.66	66.93	9.73	15278.87	740.69	4.69	77.10	22.17	71.25	3.34	1.35	27.41	10.24	2.16	91.40	97.90	491.90	91717.42
	2009	76.91	67.13	9.78	13270.70	679.68	4.86	77.07	24.06	68.78	3.34	1.52	26.82	10.14	2.15	91.40	97.90	-	91788.48
	2010	76.91	67.17	9.74	12174.70	732.68	5.63	74.93	23.10	69.18	3.89	1.73	26.24	9.99	2.13	91.40	97.90	-	91862.44
	2011	76.99	67.21	9.78	11789.80	724.02	5.65	73.97	23.01	68.89	3.89	1.76	25.67	9.88	2.12	91.40	97.90	537.81	91913.54
	2012	77.10	67.30	9.80	12069.81	728.48	5.39	74.99	24.14	67.81	3.65	1.73	25.14	9.74	2.10	-	97.90	552.75	91972.53
	2013	77.34	67.48	9.86	11928.22	711.43	5.33	74.73	24.38	67.38	3.59	1.74	24.64	9.59	2.09	-	97.90	-	92031.39
	2014	77.17	67.36	9.81	12403.53	773.71	5.54	74.73	23.69	68.29	3.78	1.76	24.19	9.52	2.08	-	97.90	-	92086.16
	2015	77.29	67.44	9.84	12771.77	-	-	-	-	-	-	-	23.77	9.56	2.06	-	97.90	-	92138.66
	2016	77.36	67.53	9.83	13315.51	-	-	-	-	-	-	-	23.39	9.74	-	-	-	-	92196.02
	2017	77.02	67.26	9.76	-	-	-	-	-	-	-	-	-	-	-	-	-	-	-
Egypt	1995	67.68	58.02	9.65	1661.33	37.25	3.54	89.64	47.92	46.54	1.65	1.89	42.81	8.52	3.71	78.80	94.60	155524.88	89825.22
	1996	68.07	58.41	9.67	1711.47	45.32	3.90	92.85	54.23	41.59	1.62	2.28	42.68	8.54	3.57	79.80	94.90	159241.03	89805.15
	1997	68.43	58.73	9.70	1772.21	58.07	4.61	95.20	58.33	38.73	1.79	2.82	42.66	8.53	3.46	81.00	95.10	165320.70	89789.54
	1998	69.00	59.20	9.80	1810.17	65.13	4.93	96.17	57.72	39.98	1.97	2.96	42.70	8.51	3.37	82.10	95.40	170721.57	89781.16
	1999	69.35	59.49	9.86	1885.74	72.00	5.26	96.96	57.80	40.39	2.12	3.13	42.75	8.46	3.29	83.20	95.60	177970.05	89773.32
	2000	69.85	59.91	9.94	1950.61	77.72	5.55	97.57	58.07	40.48	2.25	3.30	42.80	8.38	3.23	84.30	95.90	184459.74	89770.50
	2001	69.70	59.81	9.89	1982.11	74.96	5.75	98.03	58.35	40.48	2.33	3.42	42.84	8.32	3.18	85.50	96.10	187292.45	89820.91
	2002	69.79	59.89	9.90	1990.99	72.84	5.97	98.40	58.78	40.27	2.40	3.57	42.89	8.23	3.13	86.60	96.40	202779.01	89870.47

	2003	69.88	59.96	9.92	2015.90	55.49	5.41	98.41	60.79	38.22	2.07	3.34	42.94	8.13	3.09	87.70	96.60	211890.80	89918.11
	2004	70.06	60.10	9.96	2059.38	56.07	5.21	98.37	60.88	38.11	1.99	3.22	42.98	8.02	3.05	88.80	96.90	223361.42	89963.42
	2005	70.23	60.24	9.98	2112.49	63.81	5.06	98.24	59.72	39.21	1.99	3.08	43.03	7.89	3.02	90.00	97.10	243789.53	90012.27
	2006	70.22	60.29	9.94	2217.39	74.01	5.24	98.02	55.23	43.66	2.29	2.95	43.07	7.80	3.00	91.10	97.30	256320.60	90040.98
	2007	70.49	60.49	10.00	2333.47	84.31	4.95	97.79	41.34	41.34	2.05	2.90	43.08	7.70	3.02	92.20	97.60	266252.27	90059.86
	2008	70.49	60.51	9.97	2456.73	100.60	4.82	97.37	56.37	42.11	2.03	2.79	43.06	7.60	3.06	93.30	97.80	269736.63	90068.93
	2009	70.38	60.45	9.93	2524.43	117.05	5.01	96.87	57.07	41.09	2.06	2.95	43.04	7.55	3.11	94.40	98.10	266325.43	90079.06
	2010	70.46	60.55	9.91	2602.48	125.50	4.80	95.76	58.38	39.03	1.87	2.92	43.02	7.56	3.19	94.70	98.30	280255.58	90088.04
	2011	70.80	60.85	9.95	2593.60	139.00	5.04	93.67	56.67	39.50	1.99	3.05	43.00	7.66	3.25	94.70	98.60	288287.83	90052.75
	2012	70.94	61.00	9.94	2593.23	160.76	5.29	90.07	57.59	36.07	1.91	3.38	43.00	7.80	3.31	94.70	98.80	295499.75	90026.95
	2013	71.45	61.41	10.04	2591.06	159.11	5.46	90.07	56.15	37.67	2.06	3.41	43.03	7.95	3.34	94.70	99.10	-	90007.20
	2014	71.68	61.62	10.07	2608.38	177.77	5.64	90.07	55.66	38.20	2.16	3.49	43.07	8.09	3.34	94.70	99.20	-	90032.18
	2015	71.84	61.76	10.08	2665.35	-	-	-	-	-	-	-	43.14	8.19	3.31	94.70	99.40	-	90086.40
	2016	72.13	62.04	10.09	2724.40	-	-	-	-	-	-	-	43.22	8.32	-	-	-	-	90133.10
	2017	70.64	60.81	9.84	-	-	-	-	-	-	-	-	-	-	-	-	-	-	-
Argentina	1995	73.07	64.15	8.92	7630.02	612.96	8.31	69.52	27.98	59.76	4.97	3.34	88.16	15.57	2.76	89.50	95.10	275096.41	88435.42
	1996	73.25	64.31	8.94	7955.13	616.81	8.02	70.38	29.76	57.72	4.63	3.39	88.37	15.69	2.71	89.90	95.40	288246.27	88446.63
	1997	73.47	64.50	8.97	8500.94	683.69	8.36	63.21	28.79	54.45	4.55	3.81	88.56	15.79	2.66	90.20	95.60	299708.76	88405.59
	1998	73.68	64.69	8.99	8728.95	704.46	8.54	63.42	29.06	54.17	4.62	3.91	88.76	15.87	2.62	90.60	95.80	311514.70	88342.14
	1999	73.91	64.88	9.03	8339.89	724.11	9.36	63.65	28.60	55.07	5.15	4.20	88.95	15.93	2.59	91.00	96.10	333886.59	88272.95
	2000	74.19	65.12	9.07	8182.69	706.90	9.21	62.98	29.03	53.90	4.97	4.25	89.14	15.96	2.56	91.40	96.30	331450.87	88195.62
	2001	74.37	65.27	9.10	7735.49	672.65	9.38	64.01	29.43	54.03	5.07	4.31	89.33	16.01	2.54	91.70	96.50	326483.81	88191.14
	2002	74.47	65.37	9.10	6816.73	223.91	8.31	64.22	29.82	53.56	4.45	3.86	89.52	16.04	2.52	92.10	96.70	311051.93	88218.84
	2003	74.62	65.51	9.12	7337.79	277.94	8.22	64.41	31.08	51.74	4.25	3.97	89.71	16.05	2.50	92.50	96.90	344797.06	88274.96
	2004	74.92	65.75	9.17	7913.74	323.75	6.84	64.06	31.07	51.49	3.52	3.32	89.90	16.05	2.47	92.80	97.10	369944.11	88346.67
	2005	75.17	65.98	9.19	8522.52	389.82	6.85	64.30	29.88	53.53	3.66	3.18	90.08	16.06	2.45	93.20	97.30	358385.64	88437.86
	2006	75.31	66.10	9.21	9112.11	447.17	6.68	64.14	29.06	54.70	3.65	3.03	90.27	16.10	2.43	93.50	97.50	373600.56	88455.79
	2007	75.34	66.11	9.23	9830.68	539.24	6.49	61.51	58.25	58.25	3.78	2.71	90.45	16.14	2.41	93.90	97.70	384547.75	88465.16
	2008	75.55	66.28	9.27	10125.13	673.84	6.66	59.24	22.61	61.84	4.12	2.54	90.62	16.19	2.40	94.20	97.90	411503.63	88479.16

	2009	75.70	66.39	9.31	9428.50	711.95	7.63	59.24	20.13	66.02	5.04	2.59	90.80	16.25	2.38	94.50	98.10	373409.07	88501.98
	2010	75.87	66.55	9.32	10276.26	738.48	6.55	60.38	22.00	63.56	4.16	2.39	90.97	16.33	2.37	94.90	98.20	364684.25	88518.85
	2011	76.04	66.66	9.38	10780.02	795.75	5.89	63.19	23.03	63.56	3.75	2.15	91.13	16.46	2.36	95.20	98.40	372873.14	88517.87
	2012	76.16	66.75	9.41	10557.89	726.79	5.02	65.46	26.87	58.95	2.96	2.06	91.30	16.61	2.35	95.50	98.60	380295.32	88549.54
	2013	76.27	66.84	9.44	10699.20	731.45	4.99	66.85	30.19	54.84	2.74	2.25	91.45	16.77	2.34	95.80	98.80	-	88561.00
	2014	76.48	67.01	9.48	10323.21	605.19	4.79	68.94	30.73	55.43	2.65	2.13	91.60	16.93	2.32	96.10	98.90	-	88607.08
	2015	76.60	67.10	9.50	10490.02	-	-	-	-	-	-	-	91.75	17.11	2.31	96.40	99.10	-	88657.84
	2016	76.71	67.22	9.48	10154.00	-	-	-	-	-	-	-	91.89	17.31	-	-	-	-	88692.31
	2017	76.66	66.82	9.84	-	-	-	-	-	-	-	-	-	-	-	-	-	-	-
Armenia	1995	69.30	60.96	8.34	1043.54	25.62	6.42	96.13	66.23	31.10	2.00	4.42	66.06	13.44	2.06	89.40	90.50	6710.37	90717.61
	1996	69.62	61.29	8.33	1121.88	29.14	5.79	96.34	69.37	28.00	1.62	4.17	65.78	14.05	1.94	89.40	90.50	5992.24	90936.22
	1997	70.34	61.89	8.45	1172.14	30.12	5.77	94.97	71.79	24.40	1.41	4.36	65.50	14.47	1.83	89.40	91.00	6719.59	91144.76
	1998	70.84	62.32	8.52	1267.58	34.99	5.76	94.89	67.77	28.58	1.64	4.11	65.23	14.78	1.75	89.40	91.50	6737.04	91339.05
	1999	71.28	62.69	8.59	1317.74	33.89	5.68	93.94	67.28	28.38	1.61	4.07	64.95	15.13	1.69	89.40	92.10	6389.01	91522.86
	2000	71.96	63.23	8.73	1404.32	38.83	6.25	94.55	77.35	18.19	1.14	5.12	64.67	15.61	1.65	89.30	92.60	6867.71	91679.39
	2001	72.18	63.42	8.76	1548.08	41.13	5.94	95.31	69.78	26.79	1.59	4.35	64.39	16.11	1.64	89.30	93.10	6896.45	91699.12
	2002	72.56	63.72	8.84	1761.89	41.99	5.40	91.92	68.75	25.21	1.36	4.04	64.28	16.74	1.64	89.30	93.70	6460.24	91715.54
	2003	72.63	63.79	8.84	2019.98	51.51	5.56	95.04	69.13	27.26	1.52	4.05	64.25	17.37	1.65	89.40	94.30	6973.88	91718.93
	2004	72.71	63.86	8.85	2244.22	65.49	5.50	95.81	71.35	25.53	1.40	4.10	64.22	17.79	1.67	89.40	94.90	7633.47	91690.56
	2005	72.52	63.75	8.78	2571.99	86.10	5.25	96.73	61.65	36.27	1.91	3.35	64.18	17.86	1.68	89.40	95.50	8401.82	91667.11
	2006	72.43	63.71	8.72	2933.83	98.22	4.58	95.47	52.65	44.84	2.05	2.52	64.15	17.72	1.69	89.50	96.10	8743.51	91527.00
	2007	72.84	64.03	8.81	3366.16	134.06	4.31	96.44	48.72	48.72	2.10	2.21	64.12	17.29	1.70	89.50	96.70	9323.72	91415.82
	2008	72.73	63.97	8.77	3629.16	149.38	3.80	95.98	51.40	46.45	1.76	2.03	64.00	16.70	1.71	89.50	97.30	12469.98	91295.67
	2009	72.92	64.11	8.81	3136.81	132.52	4.55	95.16	53.08	44.22	2.01	2.54	63.79	16.16	1.70	89.50	97.90	11235.09	91211.93
	2010	73.27	64.41	8.86	3218.38	143.06	4.56	96.57	55.87	42.14	1.92	2.64	63.58	15.81	1.69	89.50	98.40	11393.04	91171.84
	2011	73.75	64.76	8.99	3371.67	127.01	3.71	98.45	47.05	52.21	1.94	1.77	63.37	15.59	1.68	89.50	99.00	12417.74	91233.49
	2012	74.29	65.19	9.11	3606.48	149.93	4.48	93.84	54.57	41.85	1.88	2.61	63.16	15.51	1.66	89.50	99.70	12319.39	91307.02
	2013	75.06	65.79	9.28	3710.58	158.98	4.55	93.84	53.69	42.78	1.95	2.60	62.98	15.55	1.65	89.50	99.90	-	91412.04
	2014	75.49	66.12	9.38	3827.34	161.55	4.48	93.85	53.51	42.98	1.93	2.55	62.81	15.64	1.63	89.50	100.00	-	91500.04

	2015	75.55	66.16	9.39	3935.29	-	-	-	-	-	-	-	62.67	15.75	1.62	89.50	100.00	-	91588.90
	2016	75.85	66.47	9.38	3932.55	-	-	-	-	-	-	-	62.56	16.03	-	-	-	-	91615.77
	2017	75.61	65.83	9.78	-	-	-	-	-	-	-	-	-	-	-	-	-	-	-
Australia	1995	78.30	68.04	10.26	38078.90	1591.18	7.26	47.01	16.09	65.78	4.78	2.48	86.11	17.84	1.82	100.00	100.00	489294.15	88098.28
	1996	78.50	68.24	10.26	39065.92	1789.35	7.44	48.77	16.95	65.25	4.86	2.59	86.28	18.01	1.80	100.00	100.00	508882.83	88126.93
	1997	78.80	68.50	10.29	40156.34	1787.63	7.50	50.97	16.87	66.89	5.02	2.48	86.50	18.14	1.78	100.00	100.00	921012.05	88172.51
	1998	79.11	68.78	10.33	41504.50	1613.11	7.66	59.16	19.90	66.36	5.08	2.58	86.73	18.24	1.76	100.00	100.00	920773.48	88244.75
	1999	79.40	69.04	10.36	43087.45	1775.35	7.79	58.87	18.61	68.38	5.33	2.46	86.95	18.35	1.76	100.00	100.00	1190138.38	88300.76
	2000	79.71	69.30	10.41	44223.59	1745.93	8.08	59.67	19.78	66.84	5.40	2.68	87.17	18.48	1.76	100.00	100.00	1081244.41	88493.44
	2001	80.02	69.56	10.46	44473.41	1665.20	8.18	57.10	19.25	66.28	5.42	2.76	87.38	18.58	1.74	100.00	100.00	1241515.99	88421.61
	2002	80.24	69.75	10.50	45626.62	1883.32	8.39	56.77	18.74	66.99	5.62	2.77	87.54	18.70	1.76	100.00	100.00	1117450.02	88454.49
	2003	80.54	69.99	10.55	46449.85	2370.88	8.32	53.78	18.18	66.19	5.51	2.81	87.70	18.84	1.75	100.00	100.00	1023667.58	88494.84
	2004	80.81	70.20	10.61	47817.90	2933.23	8.57	54.70	18.18	66.77	5.72	2.85	87.85	18.99	1.77	100.00	100.00	1144343.87	88522.25
	2005	81.09	70.41	10.68	48702.73	3214.03	8.45	56.12	18.54	66.97	5.66	2.79	88.00	19.16	1.81	100.00	100.00	909262.03	88546.89
	2006	81.27	70.54	10.73	49418.69	3421.91	8.49	55.73	18.57	66.67	5.66	2.83	88.15	19.21	1.91	100.00	100.00	1119632.10	88530.06
	2007	81.43	70.65	10.78	50952.47	4077.85	8.53	55.40	67.59	67.59	5.77	2.76	88.30	19.27	1.96	100.00	100.00	1065623.87	88504.98
	2008	81.58	70.74	10.83	51788.44	4410.44	8.78	55.14	17.93	67.49	5.93	2.86	88.45	19.38	1.98	100.00	100.00	892629.27	88478.42
	2009	81.79	70.88	10.91	51651.22	4256.64	9.05	57.30	18.11	68.39	6.19	2.86	88.59	19.59	1.97	100.00	100.00	1105167.24	88464.56
	2010	81.99	71.00	10.99	51874.08	5324.52	9.02	58.01	18.58	67.97	6.13	2.89	88.73	19.91	1.93	100.00	100.00	782102.69	88465.66
	2011	82.15	71.16	11.00	52372.15	6368.42	9.20	57.22	17.94	68.65	6.32	2.88	88.88	20.38	1.93	100.00	100.00	785795.15	88475.31
	2012	82.33	71.32	11.01	53348.39	6543.52	9.36	57.08	18.74	67.17	6.29	3.07	89.02	20.91	1.92	100.00	100.00	761686.27	88535.48
	2013	82.53	71.49	11.05	53798.36	6258.47	9.36	57.08	18.83	67.01	6.27	3.09	89.15	21.49	1.86	100.00	100.00	-	88614.90
	2014	82.58	71.54	11.04	54394.33	6031.11	9.42	57.08	18.82	67.04	6.32	3.11	89.29	22.07	1.83	100.00	100.00	-	88733.50
	2015	82.55	71.54	11.01	54941.91	-	-	-	-	-	-	-	89.42	22.63	1.83	100.00	100.00	-	88862.31
	2016	82.52	71.53	10.99	55670.92	-	-	-	-	-	-	-	89.55	23.16	-	-	-	-	89013.40
	2017	82.39	70.37	12.02	-	-	-	-	-	-	-	-	-	-	-	-	-	-	-
Austria	1995	76.84	67.11	9.73	36584.78	2867.56	9.55	60.04	15.15	74.76	7.14	2.41	65.80	22.64	1.42	100.00	100.00	82480.63	91909.64
	1996	77.10	67.36	9.75	37411.65	2811.46	9.53	60.87	15.53	74.48	7.10	2.43	65.80	22.73	1.45	100.00	100.00	85648.27	91941.35
	1997	77.40	67.61	9.78	38193.28	2563.92	9.83	61.21	15.38	74.88	7.36	2.47	65.80	22.74	1.39	100.00	100.00	84731.33	91995.13

	1998	77.68	67.86	9.82	39509.57	2675.56	10.00	60.89	15.34	74.81	7.48	2.52	65.80	22.70	1.37	100.00	100.00	85728.61	92073.46
	1999	77.94	68.08	9.86	40848.41	2709.57	10.19	59.98	14.95	75.07	7.65	2.54	65.80	22.67	1.34	100.00	100.00	83880.53	92153.64
	2000	78.26	68.33	9.93	42122.94	2415.78	10.06	59.97	15.07	74.88	7.54	2.53	65.80	22.70	1.36	100.00	100.00	82686.75	92246.11
	2001	78.61	68.62	10.00	42528.70	2418.44	10.12	61.40	15.87	74.15	7.50	2.62	65.80	22.87	1.33	100.00	100.00	85851.61	92317.82
	2002	78.84	68.81	10.03	43020.74	2643.58	10.27	61.05	15.83	74.07	7.61	2.66	65.81	23.05	1.39	100.00	100.00	87386.69	92391.20
	2003	79.04	68.97	10.07	43135.40	3285.60	10.48	61.24	16.03	73.82	7.74	2.74	65.81	23.27	1.38	100.00	100.00	92659.84	92439.80
	2004	79.35	69.22	10.13	44028.53	3779.75	10.56	66.67	17.48	73.78	7.79	2.77	65.82	23.54	1.42	100.00	100.00	96330.91	92493.44
	2005	79.59	69.40	10.19	44665.71	3916.48	10.53	67.96	17.42	74.36	7.83	2.70	65.82	23.88	1.41	100.00	100.00	97552.85	92556.31
	2006	79.87	69.63	10.24	45934.54	4075.33	10.35	68.09	17.19	74.75	7.74	2.61	65.83	24.34	1.41	100.00	100.00	95636.73	92595.23
	2007	80.10	69.81	10.29	47444.02	4710.11	10.40	67.71	74.76	74.76	7.78	2.63	65.84	24.86	1.38	100.00	100.00	93276.76	92648.09
	2008	80.29	69.95	10.34	48027.52	5286.92	10.60	66.66	16.39	75.40	8.00	2.61	65.84	25.40	1.42	100.00	100.00	94608.82	92707.51
	2009	80.38	70.02	10.37	46082.05	5153.79	11.19	67.02	16.33	75.63	8.47	2.73	65.85	25.91	1.39	100.00	100.00	89046.76	92762.94
	2010	80.58	70.15	10.43	46858.04	5049.56	11.17	66.95	16.58	75.23	8.40	2.77	65.85	26.37	1.44	100.00	100.00	94172.35	92824.28
	2011	80.80	70.33	10.46	48011.71	5431.67	10.94	67.02	16.68	75.11	8.21	2.72	65.86	26.77	1.43	100.00	100.00	92315.05	92851.85
	2012	80.94	70.45	10.48	48149.72	5239.52	11.17	66.35	16.34	75.37	8.42	2.75	65.86	27.13	1.44	100.00	100.00	90460.21	92876.73
	2013	81.13	70.61	10.52	47928.76	5478.24	11.14	65.36	16.24	75.16	8.37	2.77	65.88	27.46	1.44	100.00	100.00	-	92903.91
	2014	81.38	70.80	10.58	47886.56	5580.49	11.21	72.95	16.15	77.86	8.73	2.48	65.92	27.78	1.47	100.00	100.00	-	92923.36
	2015	81.46	70.88	10.59	47834.79	-	-	-	-	-	-	-	65.97	28.10	1.47	100.00	100.00	-	92942.28
	2016	81.54	70.94	10.60	47909.40	-	-	-	-	-	-	-	66.03	28.45	-	-	-	-	92979.07
	2017	81.77	70.44	11.33	-	-	-	-	-	-	-	-	-	-	-	-	-	-	-
Azerbaijan	1995	64.86	57.34	7.52	1216.08	18.02	5.79	87.35	66.36	24.03	1.39	4.40	52.21	8.70	2.29	62.70	70.20	42526.79	89449.89
	1996	65.31	57.76	7.55	1219.51	24.78	6.12	86.00	65.14	24.26	1.49	4.64	51.90	8.90	2.06	62.60	70.90	38971.06	89676.85
	1997	65.63	58.04	7.59	1277.85	27.27	5.45	80.26	62.17	22.54	1.23	4.22	51.59	9.03	2.07	62.50	71.70	37665.04	89905.16
	1998	65.80	58.22	7.59	1392.36	30.27	5.43	80.65	67.24	16.63	0.90	4.53	51.28	9.11	2.00	62.40	72.40	39953.33	90151.23
	1999	66.09	58.49	7.60	1482.33	30.50	5.36	79.37	64.77	18.39	0.99	4.37	51.19	9.20	2.07	64.00	73.20	40457.65	90394.94
	2000	66.54	58.88	7.66	1633.39	30.30	4.67	77.68	63.27	18.56	0.87	3.80	51.39	9.29	2.00	65.60	74.10	42646.56	90614.03
	2001	67.20	59.45	7.75	1781.25	31.17	4.48	77.94	63.22	18.88	0.85	3.64	51.59	9.51	1.80	67.20	75.00	41456.11	90704.36
	2002	67.48	59.70	7.78	1955.41	33.62	4.47	79.33	65.27	17.73	0.79	3.68	51.79	9.73	1.80	68.80	75.80	40298.55	90754.26
	2003	67.30	59.58	7.72	2158.01	57.04	6.56	88.55	77.35	12.64	0.83	5.73	51.99	9.90	1.90	70.50	76.70	44116.81	90793.29

	2004	67.41	59.67	7.74	2357.40	81.26	7.92	90.78	79.00	12.98	1.03	6.90	52.19	9.93	2.05	72.10	77.60	45005.73	90796.05
	2005	67.60	59.83	7.78	2949.44	121.89	7.86	92.75	82.36	11.20	0.88	6.98	52.39	9.80	2.00	73.70	78.50	47617.17	90776.26
	2006	67.96	60.13	7.83	3923.66	149.73	6.17	91.21	78.43	14.01	0.86	5.30	52.59	9.60	1.97	75.30	79.30	48492.28	90618.62
	2007	68.30	60.43	7.87	4851.18	192.38	5.10	89.85	19.24	19.24	0.98	4.11	52.79	9.30	1.97	76.80	80.20	45144.11	90452.06
	2008	68.35	60.48	7.87	5262.10	241.56	4.37	88.42	71.75	18.85	0.82	3.55	52.99	8.93	1.90	78.40	81.00	53832.82	90286.55
	2009	68.77	60.82	7.95	5639.00	288.54	5.85	88.83	68.50	22.89	1.34	4.51	53.19	8.58	1.82	80.00	81.90	49623.18	90145.76
	2010	69.09	61.09	8.00	5842.81	310.15	5.33	88.58	69.18	21.90	1.17	4.17	53.40	8.28	1.92	81.60	82.80	50724.57	90013.36
	2011	69.90	61.69	8.20	5770.97	358.26	5.01	89.04	69.83	21.58	1.08	3.93	53.62	8.13	1.96	83.20	83.60	53982.83	90013.14
	2012	70.21	61.94	8.27	5820.08	399.50	5.37	89.13	69.02	22.56	1.21	4.16	53.86	8.02	2.00	84.70	84.50	56537.08	90030.00
	2013	70.95	62.51	8.44	6078.32	432.42	5.54	89.69	71.06	20.77	1.15	4.39	54.10	7.95	1.98	86.30	85.30	-	90081.93
	2014	71.41	62.86	8.55	6122.98	471.41	6.04	90.55	72.08	20.39	1.23	4.81	54.36	7.96	1.97	87.90	86.20	-	90158.17
	2015	71.71	63.10	8.62	6117.03	-	-	-	-	-	-	-	54.62	8.04	1.97	89.30	87.00	-	90247.02
	2016	72.03	63.40	8.63	5858.83	-	-	-	-	-	-	-	54.90	8.24	-	-	-	-	90286.02
	2017	70.76	62.05	8.71	-	-	-	-	-	-	-	-	-	-	-	-	-	-	-
Bahamas	1995	70.55	62.10	8.45	21170.95	838.48	6.85	40.13	24.14	39.85	2.73	4.12	80.96	7.26	2.51	88.00	96.30	3532.13	90560.21
	1996	70.68	62.23	8.45	21782.10	879.52	6.92	40.20	24.08	40.10	2.77	4.14	81.18	7.45	2.44	88.00	96.30	3856.65	90604.52
	1997	70.81	62.34	8.47	21984.95	959.51	5.55	40.21	23.13	42.47	2.36	3.19	81.40	7.62	2.36	88.00	96.30	3535.75	90691.88
	1998	70.95	62.47	8.48	22776.61	1031.03	5.59	40.22	21.94	45.46	2.54	3.05	81.61	7.79	2.26	88.00	96.30	3683.26	90784.96
	1999	71.11	62.60	8.51	24111.86	1072.52	5.23	40.23	21.25	47.18	2.47	2.76	81.82	7.97	2.17	88.30	96.40	3580.50	90900.56
	2000	71.30	62.76	8.54	24748.31	1106.28	5.21	40.25	20.91	48.05	2.50	2.71	82.01	8.19	2.07	88.60	96.60	3185.22	91020.53
	2001	71.51	62.94	8.57	24958.65	1106.29	5.15	40.26	20.96	47.94	2.47	2.68	82.06	8.38	1.99	88.90	96.70	3151.87	91138.09
	2002	71.68	63.08	8.61	25134.38	1182.81	5.26	43.77	22.86	47.77	2.51	2.75	82.12	8.58	1.93	89.20	96.90	3255.00	91273.28
	2003	72.21	63.51	8.71	24298.62	1236.43	5.62	46.71	24.69	47.13	2.65	2.97	82.17	8.76	1.89	89.40	97.00	3556.08	91383.99
	2004	72.80	63.97	8.83	23997.85	1325.13	6.02	49.74	25.96	47.81	2.88	3.14	82.23	8.93	1.86	89.70	97.20	3731.97	91495.33
	2005	73.04	64.16	8.88	24305.99	1393.62	5.95	52.98	29.47	44.38	2.64	3.31	82.28	9.12	1.86	90.00	97.30	3854.11	91601.52
	2006	73.39	64.46	8.93	24429.45	1644.47	6.93	51.70	27.30	47.21	3.27	3.66	82.33	9.29	1.87	90.30	97.50	4014.02	91646.57
	2007	73.70	64.69	9.01	24312.40	1719.80	7.08	53.98	44.96	44.96	3.18	3.89	82.39	9.46	1.87	90.60	97.60	4128.89	91700.61
	2008	73.78	64.76	9.02	23314.99	1727.69	7.30	53.98	29.18	45.94	3.35	3.95	82.44	9.61	1.88	90.90	97.80	4339.66	91767.58
	2009	73.86	64.82	9.04	21951.15	1638.22	7.43	53.98	28.37	47.45	3.53	3.91	82.50	9.73	1.88	91.20	97.90	4335.93	91844.21

	2010	73.87	64.87	9.00	21921.56	1630.14	7.44	53.98	28.86	46.54	3.46	3.98	82.55	9.86	1.87	91.40	98.10	4570.36	91939.92
	2011	73.83	64.83	9.00	21710.59	1642.39	7.63	53.98	28.70	46.83	3.58	4.06	82.61	10.15	1.85	91.70	98.20	4726.38	91977.42
	2012	73.75	64.77	8.99	22050.20	1642.99	7.43	53.98	30.02	44.39	3.30	4.13	82.67	10.48	1.83	92.00	98.40	4865.21	92023.67
	2013	73.76	64.77	8.99	21622.87	1572.26	7.05	53.98	30.41	43.67	3.08	3.97	82.73	10.84	1.81	92.00	98.40	-	92072.50
	2014	73.82	64.82	9.00	21078.68	1720.16	7.74	53.98	29.23	45.86	3.55	4.19	82.80	11.25	1.79	92.00	98.40	-	92099.93
	2015	73.68	64.69	8.99	20184.74	-	-	-	-	-	-	-	82.87	11.70	1.78	92.00	98.40	-	92148.35
	2016	73.86	64.88	8.98	19991.09	-	-	-	-	-	-	-	82.95	12.20	-	-	-	-	92201.74
	2017	73.73	65.08	8.65	-	-	-	-	-	-	-	-	-	-	-	-	-	-	-
Bahrain	1995	70.19	60.06	10.13	21910.24	495.17	4.11	71.88	22.62	68.53	2.82	1.29	88.39	3.56	3.15	99.10	96.00	15330.07	92425.27
	1996	70.63	60.42	10.21	22220.68	491.52	4.04	71.62	22.81	68.15	2.75	1.29	88.39	3.62	3.06	99.10	96.60	15986.52	92497.25
	1997	71.02	60.71	10.31	22281.80	532.05	4.35	71.88	21.50	70.10	3.05	1.30	88.38	3.66	2.99	99.10	97.10	-	92564.79
	1998	71.32	60.94	10.39	22634.89	488.70	4.32	73.63	24.48	66.76	2.88	1.43	88.38	3.67	2.92	99.10	97.70	-	92584.90
	1999	71.67	61.18	10.49	22760.99	495.58	4.19	72.20	23.27	67.77	2.84	1.35	88.38	3.66	2.86	99.10	98.30	18047.95	92593.22
	2000	71.41	60.97	10.44	22955.09	476.89	3.51	67.98	22.21	67.33	2.36	1.15	88.37	3.64	2.80	99.10	98.90	-	92632.46
	2001	72.07	61.48	10.59	22416.08	490.26	3.80	68.13	22.66	66.74	2.53	1.26	88.37	3.49	2.73	99.20	99.40	19051.28	92744.70
	2002	72.41	61.76	10.65	22038.33	515.03	3.89	67.15	22.59	66.36	2.58	1.31	88.37	3.36	2.66	99.20	100.00	19967.01	92888.46
	2003	72.72	62.02	10.70	22058.76	543.82	3.74	67.23	22.27	66.88	2.50	1.24	88.38	3.27	2.59	99.20	100.00	20539.29	93006.97
	2004	73.00	62.25	10.75	22144.47	547.82	3.37	69.80	22.77	67.37	2.27	1.10	88.39	3.19	2.51	99.20	100.00	21451.12	93118.63
	2005	73.47	62.65	10.81	22066.07	581.65	3.16	68.37	21.68	68.29	2.16	1.00	88.40	3.11	2.44	99.20	100.00	23432.38	93197.87
	2006	73.66	62.84	10.83	21796.02	605.17	3.08	67.37	20.83	69.07	2.13	0.95	88.42	2.96	2.36	99.20	100.00	25118.22	93313.68
	2007	74.44	63.39	11.04	21838.07	651.47	3.08	66.30	70.98	70.98	2.18	0.89	88.44	2.84	2.30	99.20	100.00	-	93415.38
	2008	75.03	63.86	11.17	21563.65	730.12	3.17	63.46	17.13	73.00	2.31	0.86	88.47	2.75	2.24	99.20	100.00	-	93504.91
	2009	75.54	64.26	11.28	20797.01	725.75	3.79	63.50	17.41	72.59	2.75	1.04	88.50	2.70	2.20	99.20	100.00	29249.58	93590.06
	2010	75.95	64.65	11.30	20722.10	741.57	3.64	61.41	17.63	71.29	2.59	1.04	88.54	2.68	2.16	99.20	100.00	30921.97	93658.41
	2011	76.11	64.73	11.39	20514.76	756.55	3.40	60.24	18.03	70.06	2.38	1.02	88.58	2.77	2.14	99.20	100.00	31941.04	93599.33
	2012	76.23	64.84	11.40	20921.20	1006.91	4.37	68.66	25.21	63.28	2.76	1.60	88.62	2.84	2.12	99.20	100.00	32849.11	93545.09
	2013	76.49	65.05	11.44	21799.65	1142.50	4.69	68.73	24.50	64.36	3.02	1.67	88.67	2.92	2.10	99.20	100.00	-	93494.30
	2014	76.58	65.14	11.44	22390.68	1242.84	4.98	63.51	23.34	63.25	3.15	1.83	88.72	2.98	2.08	99.20	100.00	-	93454.36
	2015	76.67	65.22	11.45	22436.21	-	-	-	-	-	-	-	88.78	3.03	2.06	99.20	100.00	-	93459.68

	2016	76.77	65.36	11.41	-	-	-	-	-	-	-	-	88.84	3.03	-	-	-	-	93508.12
	2017	79.49	67.81	11.68	-	-	-	-	-	-	-	-	-	-	-	-	-	-	-
Bangladesh	1995	61.70	53.10	8.60	445.92	10.30	3.22	95.86	60.16	37.24	1.20	2.02	21.69	5.94	3.71	40.00	72.10	136508.33	90981.00
	1996	62.58	53.85	8.73	456.24	11.13	2.96	96.16	62.70	34.80	1.03	1.93	22.06	6.06	3.59	41.10	72.90	139004.89	91049.98
	1997	63.45	54.58	8.87	466.77	8.55	2.25	98.15	55.92	43.02	0.97	1.28	22.44	6.18	3.48	42.20	73.60	140107.01	91106.07
	1998	64.28	55.28	9.00	480.87	8.63	2.25	97.47	56.99	41.54	0.94	1.32	22.82	6.28	3.37	43.30	74.40	129129.51	91179.51
	1999	65.09	55.95	9.14	493.26	8.95	2.29	97.58	57.78	40.78	0.94	1.36	23.20	6.38	3.27	44.30	75.20	138323.90	91244.35
	2000	65.41	56.27	9.15	509.29	9.13	2.33	97.41	57.78	40.69	0.95	1.38	23.59	6.48	3.17	45.40	76.00	136012.90	91321.95
	2001	65.95	56.72	9.23	525.07	9.64	2.47	97.01	56.76	41.50	1.02	1.45	24.10	6.59	3.07	46.50	76.70	142753.53	91377.55
	2002	66.45	57.15	9.30	535.25	10.33	2.59	95.99	56.46	41.18	1.07	1.53	24.76	6.69	2.97	47.60	77.50	143812.57	91419.51
	2003	66.98	57.60	9.38	550.86	10.85	2.51	96.36	58.91	38.87	0.98	1.53	25.43	6.78	2.88	48.60	78.30	146424.25	91467.68
	2004	67.54	58.08	9.47	570.34	11.96	2.62	96.32	57.94	39.84	1.04	1.57	26.11	6.86	2.78	49.70	79.10	147368.17	91511.01
	2005	67.93	58.43	9.50	598.62	12.44	2.68	93.79	59.92	36.11	0.97	1.71	26.81	6.95	2.69	50.70	79.80	151091.70	91568.56
	2006	68.37	58.87	9.50	630.05	13.51	2.80	93.44	58.72	37.15	1.04	1.76	27.52	7.08	2.60	51.80	80.60	155320.50	91610.88
	2007	68.77	59.21	9.56	666.40	15.24	2.80	94.53	35.29	35.29	0.99	1.81	28.24	7.20	2.52	52.80	81.30	161041.76	91666.14
	2008	69.41	59.79	9.63	698.56	17.60	2.85	90.71	60.31	33.51	0.95	1.89	28.97	7.30	2.45	53.80	82.00	164700.50	91722.21
	2009	69.93	60.25	9.68	725.77	19.82	2.91	92.71	61.13	34.06	0.99	1.92	29.71	7.37	2.38	54.80	82.70	169927.91	91802.85
	2010	70.26	60.62	9.64	757.67	23.14	3.06	92.78	60.97	34.28	1.05	2.01	30.46	7.42	2.33	55.80	83.50	177504.47	91876.38
	2011	70.85	61.09	9.75	797.41	25.41	3.16	91.51	61.32	32.99	1.04	2.11	31.23	7.52	2.28	56.80	84.20	180498.43	91929.37
	2012	71.09	61.35	9.74	839.51	25.58	3.08	93.03	63.30	31.95	0.98	2.10	31.99	7.61	2.24	57.70	84.80	183300.56	92004.38
	2013	71.47	61.69	9.78	879.58	28.16	2.88	92.98	66.84	28.11	0.81	2.07	32.75	7.67	2.20	58.70	85.50	-	92079.66
	2014	71.87	62.04	9.83	922.16	30.83	2.82	92.89	66.98	27.90	0.79	2.03	33.52	7.70	2.17	59.60	86.20	-	92156.21
	2015	72.25	62.38	9.87	971.64	-	-	-	-	-	-	-	34.28	7.69	2.13	60.60	86.90	-	92259.71
	2016	72.62	62.77	9.85	1029.58	-	-	-	-	-	-	-	35.04	7.69	-	-	-	-	92343.96
	2017	73.12	63.06	10.06	-	-	-	-	-	-	-	-	-	-	-	-	-	-	-
Barbados	1995	73.83	64.81	9.02	12919.22	447.48	5.21	75.79	24.29	67.95	3.54	1.67	33.26	16.06	1.73	83.40	96.90	1099.56	92218.28
	1996	74.13	65.04	9.09	13381.99	462.00	5.07	75.84	24.01	68.34	3.46	1.60	33.39	16.28	1.73	84.00	97.00	1083.64	92298.23
	1997	74.42	65.26	9.16	13963.65	490.44	5.11	75.85	26.39	65.21	3.33	1.78	33.51	16.49	1.74	84.70	97.20	1123.04	92372.19
	1998	74.71	65.48	9.23	14431.62	526.21	4.88	76.43	26.45	65.39	3.19	1.69	33.63	16.73	1.74	85.40	97.30	1208.66	92444.04

	1999	74.97	65.66	9.31	14426.32	564.43	5.01	77.14	26.68	65.41	3.28	1.73	33.76	17.00	1.74	86.10	97.50	1310.15	92505.72
	2000	74.96	65.65	9.31	15017.08	595.97	5.16	77.27	26.43	65.80	3.39	1.76	33.83	17.33	1.74	86.80	97.60	1313.95	92559.28
	2001	75.38	65.96	9.41	14616.18	629.15	5.47	76.92	24.96	67.56	3.70	1.78	33.65	17.32	1.75	87.40	97.80	1225.12	92612.01
	2002	75.59	66.13	9.46	14688.78	672.51	5.76	77.19	24.10	68.78	3.96	1.80	33.47	17.33	1.75	88.10	97.90	1263.74	92663.76
	2003	75.78	66.28	9.50	14964.72	698.67	5.82	79.07	26.81	66.09	3.84	1.97	33.29	17.34	1.75	88.80	98.10	-	92717.42
	2004	75.88	66.35	9.53	15128.95	734.16	5.71	80.00	27.93	65.09	3.72	1.99	33.12	17.35	1.76	89.50	98.20	-	92761.19
	2005	76.01	66.46	9.55	15676.11	763.74	5.38	80.54	28.67	64.40	3.46	1.91	32.94	17.35	1.76	90.10	98.40	-	92807.41
	2006	76.12	66.56	9.56	16502.69	825.34	5.27	81.25	28.63	64.76	3.42	1.86	32.76	17.47	1.76	90.80	98.50	-	92850.60
	2007	76.32	66.70	9.62	16726.28	928.29	5.64	80.70	66.65	66.65	3.76	1.88	32.59	17.61	1.77	91.50	98.70	-	92900.09
	2008	76.58	66.89	9.69	16721.73	996.66	6.01	80.56	26.23	67.44	4.06	1.96	32.41	17.79	1.77	92.20	98.80	-	92957.61
	2009	76.73	67.00	9.73	15979.68	1026.94	6.21	80.56	26.82	66.70	4.14	2.07	32.24	18.06	1.78	92.80	99.00	-	93009.20
	2010	76.77	67.05	9.73	15959.03	980.96	6.17	80.56	28.19	65.00	4.01	2.16	32.06	18.44	1.78	93.50	99.10	-	93068.58
	2011	76.79	67.04	9.75	15972.77	1035.87	6.67	81.88	30.86	62.31	4.16	2.51	31.91	18.84	1.79	94.20	99.30	-	93117.52
	2012	76.93	67.14	9.79	15961.70	1137.95	7.43	81.88	28.52	65.17	4.84	2.59	31.77	19.34	1.79	94.90	99.40	1540.62	93167.74
	2013	76.93	67.13	9.79	15910.93	1146.95	7.57	81.88	28.87	64.74	4.90	2.67	31.65	19.94	1.79	95.50	99.60	-	93217.33
	2014	76.80	67.04	9.76	15864.62	1146.04	7.47	81.88	29.87	63.52	4.74	2.72	31.55	20.60	1.79	96.20	99.70	-	93273.49
	2015	76.68	66.95	9.74	15961.20	-	-	-	-	-	-	-	31.48	21.31	1.80	96.20	99.70	-	93338.18
	2016	76.62	66.92	9.70	16242.77	-	-	-	-	-	-	-	31.42	21.96	-	-	-	-	93409.24
	2017	77.13	67.89	9.24	-	-	-	-	-	-	-	-	-	-	-	-	-	-	-
Belarus	1995	68.23	59.78	8.45	2024.69	69.91	6.74	64.51	18.63	71.12	4.79	1.95	67.93	18.89	1.41	95.00	99.50	130352.35	91726.01
	1996	68.25	59.82	8.43	2088.35	89.29	6.24	59.84	14.77	75.32	4.70	1.54	68.31	19.27	1.34	95.00	99.50	130526.99	91856.63
	1997	68.14	59.75	8.39	2336.31	92.10	6.59	49.78	8.80	82.32	5.43	1.17	68.69	19.48	1.25	95.00	99.50	131300.40	92003.84
	1998	68.06	59.69	8.37	2544.63	90.21	5.96	58.33	13.39	77.04	4.59	1.37	69.06	19.59	1.30	94.90	99.50	87300.09	92139.57
	1999	67.95	59.61	8.34	2642.24	74.77	6.16	56.22	13.20	76.51	4.72	1.45	69.48	19.69	1.31	94.90	99.50	87514.80	92287.29
	2000	68.34	59.91	8.43	2808.69	74.97	6.13	57.13	14.01	75.49	4.63	1.50	69.97	19.85	1.32	94.80	99.50	83387.55	92423.30
	2001	68.16	59.79	8.38	2956.54	82.23	6.62	64.26	18.28	71.55	4.74	1.88	70.46	20.23	1.29	94.80	99.50	80803.90	92472.46
	2002	67.96	59.64	8.32	3125.54	95.75	6.47	69.42	20.49	70.49	4.56	1.91	70.94	20.66	1.24	94.80	99.50	89727.11	92522.08
	2003	68.30	59.91	8.39	3369.17	119.76	6.59	69.64	18.22	73.84	4.87	1.72	71.42	21.03	1.23	94.70	99.50	82041.12	92574.49
	2004	68.57	60.12	8.45	3780.64	157.24	6.59	68.32	17.26	74.74	4.92	1.66	71.89	21.22	1.23	94.70	99.50	85627.16	92602.19

	2005	68.73	60.26	8.47	4164.36	215.87	6.89	73.38	19.86	72.94	5.02	1.86	72.35	21.15	1.25	94.60	99.50	89989.72	92632.73
	2006	69.23	60.65	8.58	4608.93	244.18	6.34	74.51	22.18	70.23	4.45	1.89	72.82	21.05	1.34	94.60	99.60	94475.09	92585.23
	2007	69.83	61.08	8.75	5028.32	304.99	6.44	76.44	69.15	69.15	4.45	1.99	73.27	20.73	1.43	94.60	99.60	95710.82	92531.54
	2008	70.09	61.27	8.82	5560.38	379.11	5.95	78.61	27.45	65.08	3.87	2.08	73.73	20.30	1.49	94.50	99.60	100860.18	92476.48
	2009	70.21	61.35	8.86	5583.94	313.29	6.09	74.76	26.90	64.02	3.90	2.19	74.17	19.91	1.51	94.50	99.60	101754.79	92427.05
	2010	70.34	61.49	8.85	6029.40	322.54	5.55	88.94	19.85	77.69	4.31	1.24	74.62	19.65	1.49	94.50	99.60	108204.55	92386.82
	2011	70.62	61.66	8.96	6375.35	293.12	4.92	90.40	26.64	70.53	3.47	1.45	75.05	19.68	1.52	94.40	99.60	108921.43	92310.41
	2012	71.86	62.63	9.23	6491.67	335.97	5.01	85.32	19.46	77.19	3.87	1.14	75.47	19.82	1.62	94.40	99.70	109647.24	92231.55
	2013	72.49	63.13	9.36	6557.11	465.17	6.07	90.92	30.69	66.24	4.02	2.05	75.88	20.05	1.67	94.40	99.70	-	92164.51
	2014	73.14	63.63	9.51	6664.10	450.21	5.69	93.62	32.03	65.79	3.74	1.95	76.28	20.33	1.70	94.30	99.70	-	92107.62
	2015	73.41	63.85	9.57	6398.69	-	-	-	-	-	-	-	76.67	20.62	1.72	94.30	99.70	-	92089.82
	2016	73.60	64.09	9.51	6217.72	-	-	-	-	-	-	-	77.05	21.11	-	-	-	-	92092.09
	2017	74.03	63.92	10.11	-	-	-	-	-	-	-	-	-	-	-	-	-	-	-
Belgium	1995	76.90	66.97	9.92	35228.34	2137.81	7.61	84.42	19.59	76.80	5.85	1.77	96.78	24.07	1.56	99.50	100.00	146712.33	89866.88
	1996	77.13	67.16	9.97	35719.81	2150.83	7.91	86.45	18.87	78.18	6.18	1.73	96.85	24.47	1.59	99.50	100.00	153314.45	89874.81
	1997	77.37	67.34	10.03	36955.64	1907.11	7.77	85.83	21.13	75.38	5.85	1.91	96.92	24.84	1.60	99.50	100.00	149509.67	89904.79
	1998	77.52	67.44	10.08	37605.23	1977.77	7.89	84.09	21.19	74.80	5.90	1.99	97.00	25.18	1.60	99.50	100.00	152174.81	89922.83
	1999	77.68	67.55	10.13	38856.06	2012.69	8.08	82.04	20.86	74.58	6.02	2.05	97.07	25.47	1.62	99.50	100.00	147499.80	89942.54
	2000	77.83	67.64	10.19	40170.42	1845.34	8.12	80.89	20.54	74.61	6.06	2.06	97.13	25.72	1.67	99.50	100.00	147051.20	89960.38
	2001	78.04	67.79	10.25	40357.37	1878.37	8.29	80.16	19.71	75.41	6.25	2.04	97.18	25.95	1.67	99.50	100.00	144535.00	89997.15
	2002	78.25	67.94	10.31	40892.22	2075.07	8.46	81.45	21.34	73.80	6.24	2.22	97.24	26.13	1.65	99.50	100.00	138048.51	90032.28
	2003	78.53	68.13	10.40	41036.80	2800.31	9.30	79.20	20.31	74.36	6.92	2.39	97.29	26.27	1.67	99.50	100.00	144031.57	90082.08
	2004	78.90	68.38	10.52	42344.69	3242.84	9.32	77.29	18.19	76.47	7.13	2.19	97.35	26.35	1.72	99.50	100.00	141187.99	90102.78
	2005	79.16	68.54	10.61	42994.36	3339.72	9.24	76.32	17.60	76.94	7.11	2.13	97.40	26.37	1.76	99.50	100.00	136696.04	90123.08
	2006	79.42	68.77	10.65	43782.14	3489.81	9.17	77.46	18.66	75.92	6.96	2.21	97.45	26.40	1.80	99.50	100.00	134802.75	90195.89
	2007	79.54	68.90	10.65	44960.80	4017.79	9.25	78.08	75.15	75.15	6.95	2.30	97.50	26.34	1.82	99.50	100.00	131823.17	90281.00
	2008	79.65	69.01	10.65	44956.37	4565.95	9.60	80.52	18.47	77.06	7.40	2.20	97.55	26.25	1.85	99.50	100.00	137213.87	90377.37
	2009	79.82	69.16	10.66	43591.27	4574.69	10.39	80.47	18.20	77.39	8.04	2.35	97.59	26.23	1.84	99.50	100.00	131110.29	90489.63
	2010	80.03	69.33	10.70	44380.18	4419.44	10.17	80.32	17.93	77.68	7.90	2.27	97.64	26.35	1.86	99.50	100.00	138029.41	90628.96

	2011	80.23	69.51	10.72	44556.04	4864.15	10.42	80.78	18.73	76.81	8.01	2.42	97.69	26.56	1.81	99.50	100.00	137517.87	90615.14
	2012	80.36	69.63	10.73	44337.58	4587.91	10.54	79.64	17.78	77.67	8.19	2.35	97.73	26.87	1.79	99.50	100.00	133373.68	90623.55
	2013	80.59	69.82	10.77	44209.74	4813.42	10.57	80.45	17.85	77.81	8.23	2.35	97.78	27.26	1.75	99.50	100.00	-	90632.08
	2014	80.91	70.07	10.84	44702.32	4884.07	10.59	80.45	17.81	77.87	8.25	2.34	97.82	27.64	1.74	99.50	100.00	-	90645.96
	2015	80.89	70.09	10.81	45068.25	-	-	-	-	-	-	-	97.86	27.97	1.74	99.50	100.00	-	90644.85
	2016	80.91	70.11	10.80	45430.91	-	-	-	-	-	-	-	97.90	28.43	-	-	-	-	90658.92
	2017	81.38	69.62	11.75	-	-	-	-	-	-	-	-	-	-	-	-	-	-	-
Belize	1995	70.43	61.92	8.52	3424.09	127.21	4.25	79.96	30.39	62.00	2.63	1.61	47.48	8.57	4.11	79.30	79.00	602.93	89394.27
	1996	70.29	61.80	8.49	3363.90	108.98	3.63	78.80	34.35	56.40	2.05	1.58	47.53	8.23	4.01	80.00	80.20	573.96	89489.20
	1997	69.51	61.17	8.34	3358.51	115.72	3.92	79.36	33.53	57.74	2.26	1.66	47.57	7.87	3.91	80.60	81.40	662.67	89528.97
	1998	69.31	61.00	8.31	3352.85	113.56	3.80	78.76	34.03	56.79	2.16	1.64	47.62	7.55	3.81	81.30	82.70	923.14	89555.97
	1999	69.00	60.74	8.27	3513.86	112.39	3.67	78.65	34.99	55.51	2.04	1.63	47.66	7.28	3.70	82.00	83.90	1143.68	89541.68
	2000	68.63	60.40	8.23	3838.89	133.72	3.98	82.30	39.01	52.61	2.09	1.88	47.66	7.06	3.60	82.70	85.10	1676.47	89511.62
	2001	68.45	60.27	8.18	3910.19	153.94	4.50	81.61	34.68	57.51	2.59	1.91	47.39	6.82	3.50	83.30	86.30	1013.48	89497.19
	2002	68.90	60.64	8.26	3997.12	155.77	4.38	81.62	35.83	56.09	2.46	1.92	47.12	6.59	3.39	84.00	87.50	1702.59	89484.26
	2003	69.20	60.88	8.32	4257.49	166.87	4.53	79.35	34.04	57.10	2.59	1.95	46.85	6.34	3.28	84.60	88.70	3604.31	89492.32
	2004	69.53	61.14	8.40	4343.18	168.36	4.39	79.37	34.08	57.06	2.51	1.89	46.58	6.08	3.18	85.30	89.90	1146.09	89524.59
	2005	69.84	61.36	8.47	4342.13	175.14	4.45	79.37	32.17	59.47	2.65	1.80	46.31	5.80	3.07	86.00	91.10	2022.18	89579.54
	2006	70.16	61.64	8.52	4424.44	184.27	4.40	75.03	29.52	60.65	2.67	1.73	46.04	5.86	2.98	86.60	92.30	1276.56	89659.67
	2007	70.46	61.88	8.58	4358.47	205.89	4.76	75.13	63.49	63.49	3.02	1.74	45.77	5.93	2.89	87.30	93.50	1802.16	89749.76
	2008	70.68	62.05	8.63	4385.18	227.66	5.09	73.16	25.89	64.61	3.29	1.80	45.50	6.02	2.82	87.90	94.70	1408.15	89841.18
	2009	70.86	62.18	8.69	4307.22	247.34	5.81	73.16	24.50	66.51	3.86	1.95	45.23	6.14	2.76	88.60	96.00	1534.36	89935.55
	2010	71.14	62.38	8.76	4344.15	253.95	5.85	69.55	23.65	66.00	3.86	1.99	44.96	6.32	2.72	89.20	97.20	1498.88	90036.10
	2011	71.28	62.51	8.77	4333.36	253.19	5.61	69.77	23.40	66.47	3.73	1.88	44.72	6.20	2.68	89.90	98.50	1536.24	90070.68
	2012	71.41	62.64	8.77	4398.76	254.89	5.45	69.76	23.99	65.61	3.58	1.88	44.50	6.10	2.64	90.20	99.30	1569.86	90101.95
	2013	71.50	62.73	8.77	4359.71	273.27	5.79	69.76	24.26	65.23	3.78	2.01	44.30	6.01	2.61	90.60	99.50	-	90140.78
	2014	71.61	62.83	8.78	4415.99	278.58	5.79	69.76	23.01	67.02	3.88	1.91	44.12	5.94	2.58	90.50	99.50	-	90185.98
	2015	71.73	62.94	8.79	4446.79	-	-	-	-	-	-	-	43.97	5.90	2.54	90.50	99.50	-	90238.23
	2016	71.84	63.05	8.79	4328.02	-	-	-	-	-	-	-	43.85	5.92	-	-	-	-	90287.78

	2017	74.16	64.92	9.24	-	-	-	-	-	-	-	-	-	-	-	-	-	-	-
Benin	1995	56.51	48.55	7.96	631.17	17.01	4.69	99.89	54.94	45.00	2.11	2.58	36.76	6.80	6.36	9.40	61.70	45322.67	90242.72
	1996	56.47	48.58	7.88	638.08	17.53	4.59	99.89	55.65	44.29	2.03	2.55	37.07	6.71	6.28	9.90	62.60	45079.96	90222.59
	1997	56.89	48.97	7.92	654.54	15.37	4.31	99.89	57.86	42.08	1.81	2.50	37.39	6.62	6.20	10.40	63.40	46264.14	90206.36
	1998	57.11	49.19	7.92	660.63	16.45	4.39	99.89	55.71	44.23	1.94	2.45	37.70	6.51	6.12	10.90	64.30	49501.74	90201.15
	1999	57.38	49.45	7.93	675.68	16.94	4.59	99.89	53.94	46.00	2.11	2.48	38.02	6.41	6.04	11.40	65.10	43491.03	90189.34
	2000	57.60	49.67	7.94	694.24	14.73	4.34	99.89	55.74	44.20	1.92	2.42	38.33	6.31	5.96	11.90	66.00	40254.01	90202.35
	2001	57.80	49.90	7.90	709.47	16.33	4.69	99.88	50.69	49.25	2.31	2.38	38.65	6.25	5.89	12.40	66.90	33015.41	90168.94
	2002	58.27	50.34	7.93	720.17	16.19	4.27	99.88	55.75	44.19	1.89	2.39	38.97	6.19	5.82	12.90	67.70	47757.34	90126.40
	2003	58.62	50.66	7.95	722.67	21.49	4.63	99.88	52.19	47.75	2.21	2.42	39.30	6.12	5.75	13.40	68.60	36885.54	90081.97
	2004	59.05	51.04	8.00	732.34	23.32	4.56	99.52	52.07	47.68	2.18	2.39	39.64	6.07	5.69	14.00	69.40	31729.03	90052.54
	2005	59.49	51.43	8.07	723.20	25.21	4.73	94.87	47.72	49.69	2.35	2.38	39.98	6.02	5.63	14.50	70.30	48572.51	90028.91
	2006	59.88	51.80	8.08	730.28	26.47	4.75	94.83	47.21	50.22	2.39	2.36	40.34	6.05	5.58	15.10	71.10	36385.67	90021.06
	2007	60.28	52.17	8.11	752.22	28.77	4.55	93.75	50.75	50.75	2.31	2.24	40.70	6.08	5.52	15.60	72.00	39886.08	90024.79
	2008	60.79	52.63	8.16	767.06	31.02	4.20	91.14	44.71	50.95	2.14	2.06	41.08	6.11	5.47	16.20	72.80	37639.93	90029.88
	2009	61.38	53.14	8.24	763.11	31.81	4.46	88.90	42.53	52.16	2.33	2.14	41.46	6.11	5.42	16.70	73.70	38068.37	90053.75
	2010	61.83	53.54	8.29	757.70	34.14	4.95	85.16	38.89	54.33	2.69	2.26	41.85	6.10	5.36	17.30	74.50	33029.82	90084.73
	2011	62.25	53.97	8.28	758.58	40.02	5.37	78.95	35.88	54.56	2.93	2.44	42.26	6.11	5.31	17.80	75.30	33289.55	90081.53
	2012	62.63	54.33	8.30	773.14	36.45	4.86	69.80	38.84	44.35	2.15	2.70	42.67	6.09	5.25	18.40	76.20	33533.10	90097.10
	2013	63.02	54.69	8.33	805.94	36.96	4.59	76.67	39.68	48.25	2.22	2.38	43.09	6.06	5.18	19.00	77.00	-	90090.77
	2014	63.41	55.05	8.36	833.66	37.89	4.59	76.67	39.10	49.00	2.25	2.34	43.51	6.03	5.12	19.60	77.80	-	90100.92
	2015	63.89	55.47	8.42	827.85	-	-	-	-	-	-	-	43.95	6.01	5.05	19.70	77.90	-	90123.37
	2016	64.42	55.95	8.48	837.34	-	-	-	-	-	-	-	44.40	6.00	-	-	-	-	90128.30
	2017	64.63	55.84	8.79	-	-	-	-	-	-	-	-	-	-	-	-	-	-	-
Bermuda	1995	71.16	62.84	8.32	65690.96	-	-	-	-	-	-	-	100.00	-	1.80	-	-	496.77	90420.13
	1996	71.21	62.92	8.29	66969.93	-	-	-	-	-	-	-	100.00	-	-	-	-	504.10	90430.59
	1997	72.16	63.70	8.46	69624.44	-	-	-	-	-	-	-	100.00	-	-	-	-	-	90438.36
	1998	72.56	64.05	8.51	71810.38	-	-	-	-	-	-	-	100.00	-	-	-	-	461.72	90448.51
	1999	72.69	64.17	8.52	73825.18	-	-	-	-	-	-	-	100.00	-	-	-	-	492.03	90474.88

	2000	72.51	64.07	8.45	79988.35	-	-	-	-	-	-	-	100.00	-	1.74	-	-	518.97	90511.48
	2001	73.51	64.84	8.67	84844.74	-	-	-	-	-	-	-	100.00	-	1.75	-	-	510.11	90542.07
	2002	73.82	65.11	8.72	83048.80	-	-	-	-	-	-	-	100.00	-	1.77	-	-	527.10	90577.95
	2003	74.11	65.32	8.79	85321.24	-	-	-	-	-	-	-	100.00	-	1.80	-	-	543.01	90614.39
	2004	74.71	65.81	8.90	86733.37	-	-	-	-	-	-	-	100.00	-	1.78	-	-	591.97	90655.02
	2005	75.18	66.22	8.97	87616.39	-	-	-	-	-	-	-	100.00	-	1.76	-	-	584.76	90717.76
	2006	75.33	66.35	8.99	91944.87	-	-	-	-	-	-	-	100.00	-	1.76	-	-	600.52	90730.66
	2007	75.79	66.67	9.12	94029.61	-	-	-	-	-	-	-	100.00	-	1.76	-	-	-	90766.34
	2008	76.21	67.01	9.21	94903.19	-	-	-	-	-	-	-	100.00	-	1.77	-	-	-	90815.07
	2009	76.59	67.31	9.29	89391.21	-	-	-	-	-	-	-	100.00	-	1.76	-	-	561.26	90866.58
	2010	76.97	67.65	9.32	88207.33	-	-	-	-	-	-	-	100.00	-	1.76	-	-	594.65	90932.67
	2011	77.42	67.97	9.45	86001.17	-	-	-	-	-	-	-	100.00	-	1.59	-	-	614.88	90939.01
	2012	77.78	68.27	9.51	81547.86	-	-	-	-	-	-	-	100.00	-	1.59	-	-	632.88	90967.23
	2013	78.12	68.54	9.58	79251.78	-	-	-	-	-	-	-	100.00	-	1.63	-	-	-	91002.58
	2014	78.37	68.75	9.62	-	-	-	-	-	-	-	-	100.00	-	1.63	-	-	-	91037.19
	2015	78.84	69.12	9.72	-	-	-	-	-	-	-	-	100.00	-	1.62	-	-	-	91094.29
	2016	78.93	69.25	9.67	-	-	-	-	-	-	-	-	100.00	-	-	-	-	-	91231.23
	2017	81.34	71.38	9.96	-	-	-	-	-	-	-	-	-	-	-	-	-	-	-
Bhutan	1995	62.92	53.95	8.98	983.26	23.69	3.98	100.00	33.23	66.77	2.66	1.32	20.54	5.96	4.60	23.60	76.90	1647.89	88452.47
	1996	63.94	54.78	9.16	1029.18	38.97	6.31	100.00	22.01	77.99	4.92	1.39	21.46	6.08	4.40	25.00	78.30	1663.93	88485.86
	1997	64.45	55.23	9.22	1065.06	51.64	7.35	100.00	23.96	76.04	5.59	1.76	22.40	6.18	4.20	26.40	79.80	1597.10	88543.45
	1998	65.13	55.79	9.34	1100.16	51.13	7.24	100.00	25.09	74.91	5.42	1.82	23.38	6.28	4.00	27.90	81.20	1943.09	88610.31
	1999	66.06	56.54	9.53	1155.21	53.03	6.94	100.00	27.08	72.92	5.06	1.88	24.38	6.34	3.80	29.40	82.60	3857.57	88682.24
	2000	65.61	56.22	9.40	1201.11	53.80	6.91	100.00	22.96	77.04	5.33	1.59	25.42	6.38	3.60	31.00	83.90	2309.76	88762.65
	2001	67.22	57.52	9.69	1263.97	48.51	5.91	100.00	17.99	82.01	4.85	1.06	26.48	6.35	3.42	32.50	85.30	1613.47	88845.47
	2002	67.94	58.13	9.81	1360.79	69.57	7.75	100.00	14.77	85.23	6.61	1.14	27.57	6.31	3.24	34.10	86.60	1707.60	88931.08
	2003	68.45	58.58	9.87	1425.06	49.48	4.90	100.00	23.55	76.45	3.75	1.16	28.69	6.26	3.09	35.70	87.90	1467.57	89040.96
	2004	69.01	59.06	9.94	1469.37	48.84	4.41	100.00	27.54	72.46	3.19	1.21	29.84	6.22	2.95	37.40	89.10	1623.24	89149.07
	2005	69.46	59.51	9.95	1534.82	66.41	5.28	97.85	20.65	78.89	4.17	1.11	30.97	6.18	2.84	39.00	90.40	1617.86	89277.02

	2006	70.04	59.99	10.05	1601.92	70.92	5.27	97.74	18.15	81.44	4.29	0.98	31.71	6.22	2.73	40.50	91.50	1966.35	89393.76
	2007	70.57	60.38	10.19	1848.57	103.18	5.88	96.97	87.08	87.08	5.12	0.76	32.47	6.25	2.64	42.10	92.70	2326.55	89509.73
	2008	71.11	60.81	10.30	1898.05	119.13	6.58	97.68	15.10	84.54	5.56	1.02	33.24	6.29	2.55	43.60	93.80	1908.29	89640.98
	2009	71.38	61.05	10.34	1986.14	107.71	6.03	97.74	18.43	81.15	4.89	1.14	34.01	6.34	2.46	45.20	94.90	2190.92	89788.32
	2010	71.90	61.54	10.36	2178.92	113.85	5.17	94.74	11.49	87.88	4.54	0.63	34.79	6.40	2.38	46.80	96.00	3220.40	89939.58
	2011	72.29	61.81	10.49	2310.00	117.61	4.73	94.74	11.79	87.56	4.14	0.59	35.59	6.49	2.31	48.30	97.00	3259.61	90064.55
	2012	72.61	62.09	10.53	2387.01	90.82	3.70	94.87	25.69	72.92	2.70	1.00	36.37	6.59	2.24	49.40	98.00	3296.96	90199.80
	2013	72.90	62.34	10.56	2399.92	90.30	3.83	94.54	25.23	73.31	2.80	1.02	37.14	6.69	2.18	49.70	99.10	-	90345.46
	2014	73.20	62.59	10.60	2500.26	88.79	3.57	94.49	25.33	73.19	2.62	0.96	37.90	6.79	2.13	50.10	100.00	-	90490.24
	2015	73.49	62.84	10.65	2628.19	-	-	-	-	-	-	-	38.64	6.89	2.09	50.40	100.00	-	90622.64
	2016	73.79	63.18	10.61	2801.28	-	-	-	-	-	-	-	39.38	7.01	-	-	-	-	90758.92
	2017	74.05	64.11	9.94	-	-	-	-	-	-	-	-	-	-	-	-	-	-	-
Bolivia	1995	64.29	56.19	8.10	1504.40	33.10	3.73	76.79	33.30	56.64	2.11	1.62	59.40	8.67	4.50	33.20	73.90	170371.97	90497.42
	1996	65.01	56.82	8.19	1539.34	44.36	4.63	77.93	27.78	64.35	2.98	1.65	59.89	8.74	4.42	34.10	74.90	176121.30	90470.72
	1997	65.61	57.34	8.27	1584.12	41.99	4.17	83.87	30.22	63.97	2.67	1.50	60.38	8.81	4.33	35.00	75.80	179070.82	90454.81
	1998	66.30	57.93	8.37	1631.57	53.99	5.10	80.18	31.09	61.22	3.12	1.98	60.87	8.86	4.24	35.90	76.70	136014.87	90431.53
	1999	66.99	58.52	8.47	1607.21	62.74	6.20	83.83	35.09	58.14	3.60	2.59	61.35	8.91	4.15	36.80	77.70	225873.92	90430.22
	2000	67.65	59.09	8.56	1616.54	57.09	5.67	81.57	34.88	57.24	3.25	2.42	61.83	8.94	4.06	37.70	78.60	91400.93	90444.18
	2001	68.30	59.63	8.68	1613.41	60.48	6.31	77.91	31.69	59.32	3.74	2.57	62.31	9.01	3.96	38.60	79.50	62224.11	90483.92
	2002	68.74	60.01	8.73	1623.52	59.65	6.53	78.85	29.32	62.82	4.10	2.43	62.79	9.06	3.87	39.50	80.30	110701.19	90540.01
	2003	69.25	60.43	8.82	1637.81	49.38	5.30	80.23	27.34	65.92	3.50	1.81	63.26	9.12	3.77	40.40	81.20	124597.50	90585.75
	2004	69.69	60.81	8.88	1676.23	51.77	5.22	81.25	26.85	66.96	3.49	1.72	63.73	9.19	3.68	41.30	82.10	252571.29	90636.05
	2005	70.07	61.16	8.91	1720.10	60.16	5.67	83.77	26.69	68.14	3.86	1.81	64.19	9.30	3.60	42.20	82.90	309458.39	90699.76
	2006	70.45	61.49	8.96	1771.95	65.05	5.19	81.46	26.24	67.79	3.52	1.67	64.65	9.43	3.51	43.10	83.70	164214.82	90745.93
	2007	70.71	61.70	9.01	1821.80	70.05	4.96	81.55	68.68	68.68	3.41	1.55	65.10	9.58	3.43	44.00	84.60	227561.90	90792.82
	2008	71.04	61.99	9.05	1901.90	88.31	5.00	82.98	26.46	68.11	3.41	1.60	65.55	9.74	3.35	44.90	85.40	120003.25	90843.96
	2009	71.34	62.26	9.09	1933.74	104.13	5.76	85.27	26.84	68.52	3.95	1.81	65.99	9.89	3.27	45.80	86.20	76425.97	90886.84
	2010	71.63	62.55	9.09	1981.16	109.76	5.44	85.31	27.90	67.30	3.66	1.78	66.43	10.03	3.20	46.70	86.90	102900.52	90940.82
	2011	71.91	62.75	9.15	2051.15	132.83	5.49	79.76	25.27	68.32	3.75	1.74	66.86	10.14	3.13	47.50	87.70	620981.96	90957.85

	2012	72.21	63.02	9.19	2122.38	149.34	5.56	80.64	25.90	67.89	3.77	1.78	67.28	10.23	3.07	48.40	88.50	621726.73	90972.30
	2013	72.48	63.27	9.21	2231.48	178.73	5.96	84.13	24.35	71.05	4.24	1.73	67.70	10.32	3.02	49.30	89.20	-	90994.79
	2014	72.73	63.49	9.24	2317.26	208.78	6.33	82.73	23.11	72.07	4.57	1.77	68.11	10.43	2.97	50.20	90.00	-	91003.76
	2015	73.00	63.73	9.28	2392.98	-	-	-	-	-	-	-	68.51	10.58	2.92	50.30	90.00	-	91017.99
	2016	73.24	64.00	9.24	2457.63	-	-	-	-	-	-	-	68.91	10.72	-	-	-	-	91033.48
	2017	72.75	63.59	9.16	-	-	-	-	-	-	-	-	-	-	-	-	-	-	-
Bosnia and	1995	68.74	60.28	8.46	866.94	46.55	9.03	100.00	62.90	37.10	3.35	5.68	39.41	13.37	1.71	95.00	97.10	6576.49	92979.21
Herzegovina	1996	72.90	63.76	9.14	1665.60	73.53	10.22	100.00	53.01	46.99	4.80	5.42	39.39	13.94	1.69	95.00	97.10	7102.69	92993.99
	1997	73.39	64.16	9.22	2255.06	81.40	8.13	100.00	56.95	43.05	3.50	4.63	39.37	14.45	1.66	95.00	97.10	11979.39	93042.05
	1998	73.85	64.54	9.31	2608.20	91.29	8.15	100.00	59.14	40.86	3.33	4.82	39.35	14.94	1.62	95.00	97.10	14843.31	93124.86
	1999	74.43	64.99	9.44	2852.02	110.33	8.91	100.00	46.90	53.10	4.73	4.18	39.33	15.46	1.56	94.90	97.30	15369.30	93220.92
	2000	74.87	65.38	9.49	3002.82	103.79	7.09	100.00	43.12	56.88	4.03	3.06	39.31	16.03	1.50	94.90	97.50	22492.91	93318.52
	2001	75.16	65.56	9.60	3131.14	109.29	7.16	100.00	39.77	60.23	4.31	2.85	39.29	16.83	1.43	94.90	97.70	18080.88	93371.15
	2002	75.30	65.66	9.63	3293.14	123.83	7.01	100.00	36.92	63.08	4.42	2.59	39.26	17.69	1.37	94.90	97.80	17112.30	93406.93
	2003	75.42	65.75	9.67	3421.75	176.80	7.94	100.00	31.01	68.99	5.48	2.46	39.24	18.53	1.33	94.90	98.00	18645.79	93421.92
	2004	75.61	65.88	9.73	3628.52	240.04	9.04	100.00	42.80	57.20	5.17	3.87	39.22	19.19	1.30	94.90	98.20	20000.84	93415.78
	2005	75.78	66.06	9.72	3946.06	248.79	8.50	100.00	42.73	57.27	4.87	3.63	39.20	19.60	1.29	94.90	98.30	20780.36	93407.87
	2006	75.95	66.19	9.76	4160.56	277.66	8.28	100.00	39.88	60.12	4.98	3.30	39.18	19.85	1.29	94.80	98.50	22772.23	93408.83
	2007	76.13	66.22	9.90	4405.29	343.85	8.37	100.00	63.73	63.73	5.33	3.04	39.16	19.86	1.29	94.80	98.70	24673.09	93410.83
	2008	76.35	66.37	9.98	4664.09	426.92	8.58	100.00	32.27	67.73	5.81	2.77	39.16	19.76	1.30	94.80	98.80	24236.61	93425.02
	2009	76.59	66.52	10.07	4545.23	442.14	9.64	97.30	28.63	70.57	6.80	2.84	39.18	19.71	1.30	94.80	99.00	24471.90	93465.71
	2010	76.77	66.75	10.02	4614.83	428.65	9.58	97.17	28.30	70.88	6.79	2.79	39.23	19.80	1.31	94.80	99.20	25619.33	93522.25
	2011	76.88	66.71	10.17	4700.89	472.11	9.71	96.85	27.81	71.28	6.92	2.79	39.29	20.16	1.31	94.80	99.40	26405.84	93550.23
	2012	77.05	66.83	10.21	4714.12	446.75	9.94	96.66	27.81	71.23	7.08	2.86	39.38	20.62	1.31	94.80	99.50	27108.40	93558.03
	2013	77.16	66.91	10.24	4882.76	449.38	9.46	96.96	29.04	70.05	6.63	2.83	39.49	21.17	1.32	94.80	99.70	-	93592.91
	2014	77.17	66.92	10.25	4992.91	463.64	9.57	96.90	27.93	71.18	6.81	2.76	39.62	21.81	1.33	94.80	99.90	-	93635.30
	2015	77.35	67.04	10.31	5190.10	-	-	-	-	-	-	-	39.77	22.52	1.35	94.80	99.90	-	93684.20
	2016	77.55	67.30	10.25	5377.84	-	-	-	-	-	-	-	39.94	23.17	-	-	-	-	93742.22
	2017	76.72	65.71	11.02	-	-	-	-	-	-	-	-	-	-	-	-	-	-	-

Botswana	1995	56.19	49.22	6.98	4237.57	125.97	4.08	37.73	18.04	52.18	2.13	1.95	48.98	4.85	3.95	46.10	93.70	12153.00	87685.80
	1996	53.87	47.29	6.58	4386.85	125.67	4.04	34.28	16.25	52.58	2.12	1.91	49.83	4.87	3.84	47.20	93.90	10207.06	87702.36
	1997	51.67	45.44	6.23	4641.81	138.61	4.30	34.50	14.98	56.57	2.43	1.87	50.68	4.90	3.72	48.30	94.10	10100.56	87740.31
	1998	49.63	43.70	5.94	4585.74	127.47	3.86	36.51	15.88	56.51	2.18	1.68	51.53	4.92	3.61	49.40	94.30	34293.87	87790.41
	1999	47.94	42.20	5.74	4939.60	132.11	3.74	36.16	15.26	57.79	2.16	1.58	52.37	4.94	3.49	50.50	94.50	34724.21	87817.32
	2000	46.56	40.92	5.64	4954.77	154.79	4.64	36.70	13.78	62.45	2.90	1.74	53.22	4.97	3.39	51.60	94.70	39682.50	87878.44
	2001	45.68	40.21	5.47	4891.91	178.45	5.73	37.83	10.45	72.37	4.15	1.58	54.06	5.05	3.29	52.70	94.90	54348.93	87899.24
	2002	45.15	39.79	5.35	5115.90	197.06	6.47	36.62	8.93	75.61	4.89	1.58	54.37	5.12	3.19	53.70	95.00	51450.30	87915.87
	2003	45.23	39.85	5.39	5280.22	192.84	4.65	30.80	11.12	63.90	2.97	1.68	54.61	5.18	3.11	54.50	95.10	17482.99	87936.79
	2004	46.06	40.47	5.58	5349.00	271.23	5.56	26.38	7.93	69.95	3.89	1.67	54.84	5.24	3.04	55.40	95.20	23886.71	87953.82
	2005	47.70	41.71	5.99	5512.81	299.63	5.62	22.93	6.25	72.74	4.09	1.53	55.07	5.28	2.99	56.30	95.30	24188.46	87980.31
	2006	49.46	43.26	6.20	5883.90	263.34	4.93	20.01	5.89	70.58	3.48	1.45	55.31	5.35	2.95	57.20	95.40	43484.66	88029.98
	2007	51.07	44.66	6.41	6270.47	267.07	4.71	17.55	70.35	70.35	3.32	1.40	55.54	5.40	2.92	58.00	95.50	26082.88	88080.32
	2008	52.71	46.05	6.66	6552.78	313.44	5.55	12.73	3.72	70.78	3.93	1.62	55.77	5.46	2.90	58.90	95.70	73051.13	88123.19
	2009	54.78	47.76	7.03	5948.85	321.66	6.39	13.48	4.00	70.36	4.49	1.89	56.00	5.50	2.89	59.80	95.80	22941.97	88171.64
	2010	56.84	49.43	7.41	6346.16	378.78	5.64	11.94	4.44	62.82	3.54	2.10	56.24	5.53	2.88	60.60	95.90	81652.26	88234.97
	2011	58.69	51.12	7.56	6610.33	384.13	5.25	12.66	4.85	61.65	3.24	2.01	56.47	5.56	2.88	61.50	96.00	81891.04	88262.96
	2012	60.24	52.50	7.73	6779.39	427.52	6.27	12.66	4.68	63.06	3.96	2.32	56.70	5.59	2.86	62.40	96.10	82110.28	88277.94
	2013	62.08	54.05	8.03	7409.42	396.60	5.84	12.66	5.06	60.07	3.51	2.33	56.94	5.62	2.84	63.30	96.20	-	88319.14
	2014	63.38	55.12	8.26	7574.28	385.31	5.41	12.66	5.19	59.01	3.19	2.22	57.19	5.68	2.81	63.30	96.20	-	88359.81
	2015	64.38	55.92	8.46	7308.61	-	-	-	-	-	-	-	57.44	5.78	2.77	63.40	96.20	-	88402.10
	2016	65.23	56.60	8.63	7483.17	-	-	-	-	-	-	-	57.71	5.92	-	-	-	-	88432.21
	2017	69.07	58.89	10.18	-	-	-	-	-	-	-	-	-	-	-	-	-	-	-
Brazil	1995	69.66	60.88	8.78	8547.94	314.30	6.51	67.98	38.74	43.01	2.80	3.71	77.61	7.19	2.59	70.80	91.10	1574445.63	90304.13
	1996	69.96	61.13	8.84	8598.01	346.30	6.71	68.64	41.00	40.26	2.70	4.01	78.30	7.33	2.54	71.50	91.60	1613760.44	90410.47
	1997	70.34	61.42	8.93	8750.27	353.30	6.69	66.89	38.16	42.95	2.87	3.82	79.05	7.44	2.48	72.40	92.10	1647413.79	90519.67
	1998	70.67	61.67	9.01	8644.42	333.47	6.56	66.89	38.37	42.64	2.80	3.76	79.78	7.55	2.43	73.10	92.50	2263725.63	90631.74
	1999	70.98	61.89	9.09	8554.83	240.30	6.91	67.09	38.42	42.73	2.95	3.96	80.50	7.67	2.37	73.90	93.00	1595863.26	90744.40
	2000	71.36	62.17	9.19	8778.19	262.76	7.03	63.58	37.95	40.30	2.83	4.20	81.19	7.81	2.30	74.70	93.50	1222630.43	90866.17

	2001	71.76	62.49	9.27	8776.86	225.78	7.19	62.59	36.12	42.29	3.04	4.15	81.55	8.01	2.23	75.30	93.80	1277451.88	90926.41
	2002	72.06	62.73	9.33	8924.33	201.09	7.13	62.52	34.61	44.64	3.18	3.95	81.88	8.23	2.16	75.90	94.20	1591827.05	90990.89
	2003	72.33	62.94	9.39	8910.86	211.33	6.94	62.61	34.83	44.37	3.08	3.86	82.20	8.45	2.10	76.50	94.50	1624554.70	91055.19
	2004	72.62	63.16	9.45	9309.01	254.29	7.07	62.64	33.19	47.02	3.32	3.74	82.52	8.66	2.03	77.10	94.90	1967427.39	91115.96
	2005	72.93	63.42	9.51	9495.10	391.29	8.27	62.77	36.71	41.51	3.43	4.84	82.83	8.86	1.98	77.70	95.20	2386439.68	91179.17
	2006	73.20	63.67	9.53	9761.88	485.39	8.36	61.82	35.98	41.80	3.49	4.86	83.14	9.06	1.93	78.20	95.60	1621079.23	91232.56
	2007	73.46	63.88	9.58	10245.23	599.60	8.28	58.46	41.73	41.73	3.46	4.83	83.45	9.24	1.88	78.80	95.90	2703498.56	91287.96
	2008	73.72	64.11	9.61	10658.23	716.76	8.24	56.04	31.47	43.84	3.61	4.63	83.75	9.42	1.85	79.40	96.20	1532859.83	91347.95
	2009	73.94	64.30	9.64	10540.11	731.92	8.65	57.19	31.79	44.41	3.84	4.81	84.04	9.62	1.82	79.90	96.50	1211969.41	91410.80
	2010	74.15	64.52	9.63	11224.15	919.67	8.27	50.40	27.32	45.80	3.79	4.48	84.34	9.85	1.81	80.50	96.90	2902242.62	91479.46
	2011	74.38	64.69	9.69	11559.21	1055.14	8.09	48.96	26.85	45.17	3.65	4.44	84.62	10.13	1.79	81.10	97.20	2953040.52	91584.35
	2012	74.64	64.90	9.73	11671.18	984.92	8.26	48.31	26.90	44.32	3.66	4.60	84.90	10.42	1.78	81.60	97.50	2989417.96	91697.40
	2013	74.84	65.08	9.76	11912.15	993.46	8.48	46.93	25.76	45.12	3.83	4.66	85.17	10.74	1.77	82.10	97.80	-	91814.44
	2014	75.01	65.22	9.79	11866.39	947.43	8.32	47.20	25.47	46.04	3.83	4.49	85.43	11.08	1.75	82.70	98.10	-	91944.66
	2015	75.14	65.34	9.80	11322.15	-	-	-	-	-	-	-	85.69	11.45	1.74	82.80	98.10	-	92087.51
	2016	75.24	65.48	9.76	10826.27	-	-	-	-	-	-	-	85.93	11.85	-	-	-	-	92229.31
	2017	75.51	65.40	10.11	-	-	-	-	-	-	-	-	-	-	-	-	-	-	-
Brunei	1995	74.21	65.07	9.14	37703.79	475.80	2.97	98.31	22.25	77.37	2.29	0.67	68.64	4.14	2.80	-	-	21264.68	83178.03
	1996	74.48	65.28	9.20	37833.36	629.83	3.72	98.39	18.69	81.00	3.02	0.71	69.16	4.02	2.69	-	-	18153.20	83268.90
	1997	74.92	65.64	9.28	36400.04	626.75	3.74	98.44	18.93	80.77	3.02	0.72	69.67	3.91	2.56	-	-	18632.40	83361.29
	1998	75.34	65.99	9.35	35382.14	569.42	4.46	98.33	16.27	83.45	3.72	0.74	70.17	3.80	2.44	-	-	17789.18	83497.89
	1999	75.68	66.27	9.41	35672.34	542.03	3.82	98.28	16.28	83.44	3.18	0.63	70.67	3.69	2.33	-	-	9479.82	83635.28
	2000	75.83	66.42	9.41	35923.40	554.45	3.05	98.16	14.64	85.09	2.60	0.46	71.16	3.58	2.22	-	-	9649.40	83799.65
	2001	76.10	66.63	9.47	36162.98	508.30	3.06	98.32	15.50	84.23	2.58	0.48	71.65	3.74	2.12	-	-	9865.14	83932.60
	2002	76.33	66.83	9.49	36832.27	516.68	3.04	98.30	15.18	84.56	2.57	0.47	72.14	3.88	2.03	-	-	9840.81	84102.78
	2003	76.50	66.99	9.51	37202.36	579.79	3.09	98.90	15.91	83.91	2.59	0.50	72.61	4.01	1.95	-	-	10762.04	84295.64
	2004	76.61	67.09	9.52	36752.05	665.22	3.01	98.90	15.91	83.92	2.52	0.48	73.06	4.14	1.89	-	-	10320.10	84490.76
	2005	76.64	67.16	9.48	36325.12	691.29	2.62	98.90	15.40	84.43	2.22	0.41	73.50	4.30	1.85	-	-	10968.96	84708.93
	2006	76.63	67.17	9.46	37401.05	699.00	2.24	98.74	13.84	85.99	1.93	0.31	73.93	4.46	1.82	-	-	13195.22	84851.38

	2007	76.62	67.14	9.48	36997.81	758.10	2.32	98.55	87.96	87.96	2.04	0.28	74.35	4.60	1.81	-	-	12998.23	84997.05
	2008	76.72	67.24	9.49	35860.39	842.72	2.23	98.34	9.43	90.42	2.02	0.21	74.75	4.70	1.81	-	-	13738.06	85138.30
	2009	76.79	67.30	9.49	34812.71	789.85	2.85	98.10	8.84	90.99	2.59	0.26	75.13	4.78	1.82	-	-	13549.80	85286.23
	2010	76.95	67.48	9.47	35268.10	859.66	2.73	97.83	7.63	92.20	2.52	0.21	75.51	4.82	1.84	-	-	14123.60	85414.96
	2011	76.84	67.36	9.47	36092.10	938.48	2.25	97.83	7.80	92.02	2.07	0.18	75.87	4.92	1.86	-	-	14494.49	85584.85
	2012	76.75	67.31	9.45	35899.04	961.89	2.30	97.83	8.00	91.82	2.11	0.19	76.22	5.04	1.87	-	-	14828.97	85763.36
	2013	76.79	67.34	9.45	34618.97	1022.87	2.61	97.83	7.68	92.15	2.41	0.21	76.56	5.20	1.88	-	-	-	85951.60
	2014	76.75	67.32	9.43	33313.83	957.61	2.65	97.83	6.01	93.86	2.49	0.16	76.89	5.40	1.89	-	-	-	86140.86
	2015	76.79	67.36	9.44	32661.86	-	-	-	-	-	-	-	77.20	5.67	1.88	-	-	-	86334.40
	2016	76.88	67.48	9.40	31430.96	-	-	-	-	-	-	-	77.51	5.98	-	-	-	-	86514.55
	2017	75.35	66.17	9.18	-	-	-	-	-	-	-	-	-	-	-	-	-	-	-
Bulgaria	1995	70.87	62.14	8.72	3781.90	81.38	4.75	100.00	26.04	73.96	3.52	1.24	67.78	22.55	1.23	85.50	99.80	81769.92	93532.67
	1996	70.74	62.08	8.66	3862.28	55.96	4.57	100.00	30.40	69.60	3.18	1.39	68.01	23.06	1.23	85.50	99.80	82852.67	93616.74
	1997	70.51	61.93	8.58	3843.12	64.20	4.74	100.00	28.34	71.66	3.40	1.34	68.23	23.55	1.09	85.50	99.80	78532.98	93685.60
	1998	71.01	62.38	8.64	4004.26	82.66	4.67	100.00	30.10	69.88	3.26	1.41	68.45	23.98	1.11	85.60	99.80	71902.28	93732.15
	1999	71.42	62.72	8.70	3800.87	96.16	5.86	100.00	33.67	66.33	3.88	1.97	68.68	24.31	1.23	85.60	99.80	65864.38	93753.27
	2000	71.67	62.96	8.71	4011.10	97.88	6.07	100.00	39.07	60.93	3.70	2.37	68.90	24.49	1.26	85.60	99.70	66195.09	93785.94
	2001	71.91	63.15	8.76	4265.56	126.03	7.23	99.94	41.58	58.39	4.22	3.01	69.17	24.79	1.21	85.70	99.70	68733.68	93804.41
	2002	72.17	63.37	8.81	4621.50	155.17	7.41	98.46	38.15	61.26	4.54	2.87	69.52	24.97	1.23	85.80	99.70	63711.02	93835.34
	2003	72.38	63.53	8.85	4894.78	201.88	7.43	97.61	36.98	62.11	4.62	2.82	69.88	25.06	1.26	85.80	99.70	70024.03	93857.41
	2004	72.47	63.61	8.87	5255.18	239.47	7.09	97.38	38.22	60.76	4.31	2.78	70.23	25.10	1.33	85.80	99.60	68865.66	93874.92
	2005	72.57	63.71	8.86	5678.05	274.97	7.08	96.95	37.89	60.92	4.31	2.77	70.58	25.13	1.37	85.90	99.60	68542.77	93888.81
	2006	72.71	63.82	8.88	6107.71	300.39	6.67	97.09	41.79	56.95	3.80	2.87	70.93	25.33	1.44	85.90	99.60	69900.82	93856.06
	2007	72.98	64.01	8.97	6625.03	379.48	6.41	97.13	58.20	58.20	3.73	2.68	71.28	25.50	1.49	85.90	99.60	72526.72	93819.48
	2008	73.27	64.23	9.05	6914.40	483.39	6.61	97.35	40.37	58.54	3.87	2.74	71.62	25.69	1.56	85.90	99.60	71720.34	93790.40
	2009	73.53	64.41	9.12	6709.53	471.57	6.78	97.14	43.37	55.35	3.75	3.03	71.96	25.98	1.66	85.90	99.50	65132.86	93764.26
	2010	73.80	64.63	9.17	6843.26	488.21	7.24	96.77	42.88	55.69	4.03	3.21	72.30	26.41	1.57	85.90	99.50	67945.31	93773.26
	2011	74.25	64.95	9.31	7019.18	532.50	6.88	96.77	43.91	54.62	3.76	3.12	72.64	27.10	1.51	85.90	99.50	72946.62	93768.96
	2012	74.63	65.24	9.40	7062.14	519.48	7.11	97.28	42.52	56.29	4.00	3.11	72.97	27.91	1.50	86.00	99.50	67942.70	93779.85

	2013	74.85	65.41	9.45	7162.99	606.03	7.93	97.47	46.80	51.99	4.12	3.81	73.30	28.80	1.48	86.00	99.50	-	93807.04
	2014	74.93	65.46	9.47	7299.55	661.85	8.44	97.28	44.19	54.57	4.61	3.84	73.63	29.67	1.53	86.00	99.40	-	93830.08
	2015	74.98	65.50	9.48	7612.02	-	-	-	-	-	-	-	73.95	30.46	1.53	86.00	99.40	-	93864.02
	2016	75.03	65.60	9.43	7967.71	-	-	-	-	-	-	-	74.27	31.29	-	-	-	-	93907.55
	2017	74.88	64.87	10.01	-	-	-	-	-	-	-	-	-	-	-	-	-	-	-
Burkina	1995	50.74	43.36	7.38	361.12	11.58	4.91	94.33	58.47	38.02	1.87	3.04	15.13	6.12	6.84	9.20	50.70	14072.24	92488.55
	1996	50.45	43.17	7.27	389.97	12.81	5.14	94.38	58.84	37.66	1.93	3.20	15.41	6.03	6.80	9.60	52.50	14494.96	92469.07
	1997	50.92	43.61	7.31	403.22	11.77	5.13	94.33	56.30	40.32	2.07	3.06	15.88	5.91	6.75	10.00	54.30	15472.87	92465.82
	1998	51.40	44.06	7.35	420.72	13.45	5.26	94.38	57.40	39.19	2.06	3.20	16.51	5.77	6.70	10.50	56.20	45357.72	92447.24
	1999	51.85	44.46	7.39	439.30	13.93	5.27	94.34	55.09	41.60	2.19	3.08	17.17	5.63	6.65	11.00	58.00	41428.10	92428.04
	2000	52.22	44.79	7.43	434.76	11.48	5.06	94.33	56.72	39.87	2.02	3.04	17.84	5.48	6.59	11.60	59.90	46470.10	92416.07
	2001	52.69	45.38	7.31	450.45	11.51	4.85	94.32	58.70	37.77	1.83	3.02	18.54	5.41	6.53	12.10	61.80	41227.96	92397.35
	2002	53.17	45.82	7.36	456.73	13.73	5.26	94.32	53.86	42.89	2.26	3.00	19.26	5.33	6.47	12.70	63.60	50062.48	92388.28
	2003	53.85	46.41	7.44	478.30	18.64	5.61	92.22	49.41	46.42	2.60	3.01	20.00	5.23	6.40	13.20	65.40	43713.29	92377.06
	2004	54.42	46.91	7.51	485.31	23.96	6.45	95.18	44.08	53.68	3.46	2.99	20.76	5.14	6.34	13.80	67.30	43284.41	92384.39
	2005	55.10	47.50	7.60	511.97	27.98	6.87	94.25	38.13	59.54	4.09	2.78	21.54	5.04	6.27	14.40	69.10	47809.70	92403.61
	2006	55.64	48.05	7.59	527.96	27.65	6.58	91.28	39.37	56.86	3.74	2.84	22.34	5.01	6.19	15.00	71.00	41364.17	92373.64
	2007	56.21	48.60	7.62	541.27	31.47	6.63	93.73	60.27	60.27	4.00	2.63	23.16	4.98	6.12	15.60	72.80	43631.84	92350.34
	2008	56.88	49.22	7.66	563.45	38.51	6.77	92.95	38.06	59.05	4.00	2.77	23.99	4.93	6.04	16.20	74.60	40706.30	92335.59
	2009	57.43	49.73	7.70	562.84	40.87	7.41	73.97	37.43	49.40	3.66	3.75	24.83	4.88	5.95	16.80	76.40	43473.10	92315.90
	2010	57.91	50.19	7.72	575.45	41.19	7.17	73.77	32.91	55.39	3.97	3.20	25.67	4.81	5.87	17.40	78.20	43405.91	92317.87
	2011	58.45	50.75	7.70	595.39	34.42	5.17	99.85	42.77	57.17	2.96	2.21	26.51	4.79	5.78	18.00	80.00	43660.15	92304.75
	2012	58.90	51.19	7.71	615.10	35.85	5.33	86.29	38.01	55.95	2.98	2.35	27.35	4.76	5.69	18.60	81.70	43910.40	92314.77
	2013	59.38	51.64	7.74	631.61	42.21	5.91	82.11	33.65	59.02	3.49	2.42	28.19	4.72	5.61	19.00	81.90	-	92323.87
	2014	59.85	52.06	7.78	639.71	35.20	4.96	81.94	39.09	52.30	2.59	2.37	29.02	4.68	5.52	19.40	82.10	-	92342.30
	2015	60.35	52.51	7.83	645.37	-	-	-	-	-	-	-	29.86	4.63	5.44	19.70	82.30	-	92356.15
	2016	60.79	52.93	7.86	663.91	-	-	-	-	-	-	-	30.69	4.62	-	-	-	-	92363.84
	2017	61.68	53.43	8.24	-	-	-	-	-	-	-	-	-	-	-	-	-	-	-
Burundi	1995	46.96	41.57	5.38	263.27	8.61	4.37	75.17	52.04	30.78	1.35	3.03	7.21	6.21	7.29	43.00	70.30	2998.94	88678.09

	1996	46.58	41.29	5.29	239.04	7.57	4.50	68.21	45.54	33.22	1.49	3.00	7.41	6.30	7.24	43.30	70.60	3046.74	88691.55
	1997	46.99	41.64	5.35	232.51	7.65	4.11	68.90	49.96	27.49	1.13	2.98	7.62	6.37	7.18	43.50	70.90	3059.80	88707.88
	1998	47.27	41.87	5.40	240.63	7.58	4.50	68.62	49.60	27.73	1.25	3.25	7.83	6.41	7.13	43.80	71.20	6229.07	88756.67
	1999	47.77	42.29	5.49	234.68	6.58	4.39	68.48	49.17	28.20	1.24	3.15	8.04	6.41	7.07	44.00	71.50	2767.73	88806.56
	2000	47.89	42.36	5.53	228.25	6.40	4.98	72.70	52.19	28.21	1.40	3.57	8.25	6.37	7.01	44.30	71.80	3398.87	88867.05
	2001	49.01	43.33	5.68	227.43	6.26	4.96	74.02	49.63	32.95	1.63	3.33	8.46	6.23	6.95	44.60	72.10	3390.84	88911.29
	2002	49.85	44.06	5.78	231.00	5.94	5.15	75.84	52.95	30.18	1.56	3.60	8.68	6.07	6.89	44.80	72.40	5029.51	88949.82
	2003	50.84	44.93	5.91	221.23	5.56	5.25	75.06	55.82	25.63	1.35	3.90	8.91	5.91	6.82	45.10	72.70	5258.75	88986.72
	2004	51.97	45.92	6.06	224.52	8.48	7.10	67.72	43.46	35.83	2.54	4.56	9.14	5.75	6.76	45.30	73.00	5370.44	88983.70
	2005	53.09	46.90	6.19	219.19	13.85	9.84	69.87	49.01	29.86	2.94	6.90	9.38	5.59	6.69	45.60	73.30	5302.31	88993.71
	2006	54.30	47.99	6.30	223.40	17.80	11.49	69.18	47.94	30.71	3.53	7.96	9.62	5.46	6.62	45.90	73.50	5553.35	88955.20
	2007	55.40	48.95	6.45	226.30	15.97	10.03	60.49	37.70	37.70	3.78	6.25	9.86	5.34	6.53	46.10	73.80	5477.68	88914.99
	2008	56.30	49.76	6.54	229.83	14.36	7.86	65.36	37.46	42.69	3.36	4.50	10.12	5.22	6.45	46.40	74.10	5724.96	88872.90
	2009	57.13	50.50	6.63	230.05	13.53	6.96	67.53	37.54	44.41	3.09	3.87	10.38	5.10	6.35	46.70	74.40	5925.88	88843.59
	2010	57.71	51.07	6.64	231.19	18.95	8.82	69.75	26.26	62.35	5.50	3.32	10.64	4.99	6.26	46.90	74.70	6040.98	88824.29
	2011	58.20	51.49	6.70	233.52	20.77	8.58	69.67	25.80	62.97	5.40	3.18	10.91	4.92	6.16	47.20	75.00	6152.00	88683.29
	2012	58.74	52.00	6.74	235.71	20.35	8.21	54.90	21.92	60.08	4.93	3.28	11.19	4.84	6.06	47.50	75.30	6254.37	88570.69
	2013	59.20	52.43	6.77	239.33	20.86	8.03	44.67	20.21	54.74	4.40	3.63	11.47	4.78	5.96	47.80	75.50	-	88453.40
	2014	59.58	52.78	6.80	243.10	21.55	7.54	44.48	21.02	52.73	3.97	3.56	11.76	4.74	5.87	48.00	75.80	-	88368.19
	2015	59.97	53.14	6.84	226.53	-	-	-	-	-	-	-	12.06	4.74	5.78	48.00	75.90	-	88268.33
	2016	60.34	53.51	6.83	218.28	-	-	-	-	-	-	-	12.36	4.81	-	-	-	-	88203.48
	2017	61.55	53.80	7.75	-	-	-	-	-	-	-	-	-	-	-	-	-	-	-
Cambodia	1995	58.19	50.59	7.60	343.47	17.33	5.39	99.30	78.58	20.86	1.12	4.26	17.31	6.01	4.69	7.70	30.30	20790.18	90454.76
	1996	58.35	50.74	7.61	351.29	23.63	7.43	99.20	63.90	35.59	2.64	4.78	17.68	5.99	4.50	9.40	32.60	19023.22	90507.94
	1997	58.61	50.96	7.66	360.66	19.62	6.46	99.30	67.70	31.82	2.06	4.40	18.06	5.93	4.33	11.20	34.80	19550.57	90601.02
	1998	58.88	51.18	7.70	368.87	16.19	6.02	99.23	76.87	22.53	1.36	4.67	18.36	5.82	4.15	12.90	37.10	125316.38	90702.84
	1999	59.22	51.46	7.76	402.87	16.49	5.60	99.33	78.31	21.16	1.18	4.41	18.47	5.69	3.97	14.60	39.40	54731.98	90807.42
	2000	59.53	51.69	7.84	428.51	17.63	5.87	99.36	76.65	22.85	1.34	4.53	18.59	5.57	3.81	16.30	41.60	42118.31	90910.62
	2001	60.14	52.26	7.88	454.08	18.28	5.70	99.39	76.66	22.86	1.30	4.40	18.70	5.58	3.65	18.00	43.90	63995.78	90914.40

	2002	60.75	52.81	7.94	475.05	20.11	5.95	99.34	73.94	25.57	1.52	4.43	18.82	5.60	3.52	19.80	46.10	108594.63	90902.68
	2003	61.41	53.40	8.01	506.70	24.38	6.74	99.29	63.71	35.83	2.42	4.33	18.94	5.63	3.41	21.50	48.40	105986.66	90880.13
	2004	62.12	54.02	8.10	550.10	26.18	6.43	99.23	62.33	37.19	2.39	4.04	19.06	5.66	3.31	23.20	50.60	178379.10	90845.91
	2005	62.96	54.75	8.20	613.28	27.60	5.84	99.26	71.76	27.70	1.62	4.22	19.17	5.69	3.23	24.90	52.90	101252.49	90813.09
	2006	63.83	55.59	8.24	669.04	24.09	4.48	99.08	71.76	27.58	1.24	3.24	19.29	5.74	3.16	26.60	55.10	102119.77	90747.89
	2007	64.58	56.26	8.32	726.46	23.57	3.75	98.81	35.29	35.29	1.32	2.42	19.41	5.78	3.09	28.40	57.40	197112.04	90680.79
	2008	65.26	56.87	8.38	763.69	41.27	5.55	75.89	60.70	20.01	1.11	4.44	19.53	5.82	3.02	30.10	59.70	120478.86	90640.85
	2009	65.78	57.34	8.43	752.98	46.74	6.36	75.95	60.26	20.65	1.31	5.04	19.67	5.87	2.95	31.90	61.90	87674.95	90604.13
	2010	66.20	57.77	8.43	785.69	46.59	5.95	79.52	60.27	24.20	1.44	4.51	19.81	5.93	2.88	33.60	64.20	126272.20	90588.45
	2011	66.69	58.20	8.50	827.98	49.60	5.64	80.28	61.21	23.75	1.34	4.30	19.97	6.00	2.81	35.40	66.50	126838.76	90570.38
	2012	67.25	58.69	8.56	874.16	59.12	6.24	93.48	70.96	24.10	1.50	4.74	20.14	6.07	2.74	37.20	68.80	127399.59	90563.71
	2013	67.66	59.06	8.60	923.73	60.44	5.93	95.63	73.10	23.56	1.40	4.54	20.32	6.15	2.68	39.00	71.10	-	90553.05
	2014	68.05	59.41	8.64	972.98	61.28	5.68	95.17	74.19	22.04	1.25	4.42	20.51	6.26	2.63	40.80	73.40	-	90533.33
	2015	68.41	59.74	8.67	1024.87	-	-	-	-	-	-	-	20.72	6.41	2.59	42.40	75.50	-	90524.56
	2016	68.75	60.12	8.63	1079.11	-	-	-	-	-	-	-	20.95	6.62	-	-	-	-	90519.10
	2017	69.88	60.74	9.14	-	-	-	-	-	-	-	-	-	-	-	-	-	-	-
Cameroon	1995	56.48	48.70	7.78	1065.12	25.15	3.86	93.76	71.90	23.32	0.90	2.96	42.57	6.86	5.98	41.00	56.50	90767.15	88257.49
	1996	56.05	48.37	7.68	1083.28	34.46	5.17	95.34	78.85	17.29	0.89	4.27	43.16	6.83	5.89	41.30	57.60	89198.54	88283.33
	1997	55.50	47.95	7.56	1111.55	28.81	4.64	94.54	74.62	21.08	0.98	3.67	43.76	6.77	5.80	41.50	58.60	90784.24	88330.72
	1998	54.99	47.55	7.44	1132.95	28.77	4.42	94.51	77.75	17.74	0.78	3.64	44.35	6.70	5.72	41.70	59.70	157727.29	88367.88
	1999	54.77	47.37	7.40	1152.35	28.64	4.43	94.47	76.70	18.80	0.83	3.60	44.94	6.62	5.65	42.00	60.70	134749.25	88430.12
	2000	54.45	47.10	7.34	1163.43	26.12	4.48	94.40	74.74	20.82	0.93	3.55	45.54	6.52	5.58	42.20	61.70	163101.04	88480.35
	2001	54.42	47.11	7.31	1183.44	27.70	4.70	94.43	71.75	24.02	1.13	3.57	46.14	6.50	5.52	42.40	62.80	118945.30	88463.64
	2002	54.44	47.15	7.30	1201.91	31.53	4.86	94.43	69.38	26.53	1.29	3.57	46.74	6.46	5.48	42.70	63.80	128242.37	88474.15
	2003	54.50	47.21	7.29	1224.16	39.34	4.97	94.52	69.21	26.78	1.33	3.64	47.34	6.41	5.43	42.90	64.90	167009.84	88468.26
	2004	54.63	47.33	7.30	1272.85	42.24	4.73	94.47	71.91	23.88	1.13	3.60	47.94	6.34	5.39	43.20	65.90	97585.68	88472.20
	2005	54.86	47.52	7.34	1264.15	43.48	4.75	94.50	72.22	23.57	1.12	3.63	48.54	6.27	5.35	43.40	66.90	160728.00	88472.14
	2006	55.27	47.93	7.34	1272.88	45.23	4.69	94.48	72.78	22.97	1.08	3.61	49.14	6.24	5.31	43.70	68.00	123887.44	88464.02
	2007	55.60	48.25	7.34	1299.29	50.94	4.76	94.57	22.41	22.41	1.07	3.69	49.74	6.20	5.27	43.90	69.00	120331.65	88454.18

	2008	56.17	48.77	7.40	1308.24	61.69	5.18	95.12	75.84	20.27	1.05	4.13	50.34	6.16	5.22	44.20	70.00	120917.19	88457.18
	2009	56.76	49.30	7.47	1300.84	59.05	5.07	94.68	70.72	25.31	1.28	3.79	50.93	6.10	5.17	44.40	71.00	116739.19	88461.23
	2010	57.24	49.73	7.50	1309.12	60.63	5.28	94.67	67.66	28.53	1.51	3.78	51.52	6.04	5.11	44.70	72.10	99823.28	88466.38
	2011	57.67	50.16	7.51	1326.65	49.87	3.96	73.35	48.70	33.61	1.33	2.63	52.10	6.02	5.05	44.90	73.10	100386.55	88463.21
	2012	58.24	50.70	7.54	1349.95	53.03	4.34	85.98	64.84	24.58	1.07	3.27	52.68	6.00	4.98	45.20	74.10	100922.14	88477.69
	2013	58.72	51.16	7.56	1385.24	57.15	4.29	85.98	65.70	23.58	1.01	3.28	53.25	5.96	4.92	45.40	75.10	-	88491.31
	2014	59.13	51.55	7.58	1428.22	58.65	4.10	85.98	66.31	22.87	0.94	3.17	53.82	5.92	4.85	45.60	75.40	-	88520.78
	2015	59.63	52.01	7.62	1469.64	-	-	-	-	-	-	-	54.38	5.88	4.78	45.80	75.60	-	88568.74
	2016	60.14	52.49	7.65	1495.44	-	-	-	-	-	-	-	54.94	5.87	-	-	-	-	88613.83
	2017	62.98	54.49	8.49	-	-	-	-	-	-	-	-	-	-	-	-	-	-	-
Canada	1995	78.07	68.26	9.81	37569.47	1830.54	8.86	55.61	15.99	71.25	6.31	2.55	77.68	17.68	1.64	99.80	99.80	836025.63	89170.56
	1996	78.31	68.46	9.85	37765.73	1838.85	8.64	55.63	16.26	70.77	6.11	2.52	77.95	17.86	1.59	99.80	99.80	698763.38	89251.20
	1997	78.57	68.67	9.90	38967.95	1882.63	8.60	56.54	16.90	70.12	6.03	2.57	78.34	18.02	1.55	99.80	99.80	690068.75	89346.02
	1998	78.78	68.84	9.94	40131.70	1856.23	8.83	55.31	16.28	70.56	6.23	2.60	78.72	18.17	1.53	99.80	99.80	1129087.11	89434.46
	1999	79.01	69.02	10.00	41856.05	1945.37	8.73	54.54	16.38	69.96	6.11	2.62	79.10	18.30	1.51	99.80	99.80	821594.26	89528.78
	2000	79.26	69.19	10.07	43638.28	2099.84	8.67	53.67	15.91	70.35	6.10	2.57	79.48	18.41	1.49	99.80	99.80	760643.00	89647.89
	2001	79.48	69.40	10.09	43964.95	2162.23	9.10	50.73	15.23	69.98	6.36	2.73	79.81	18.51	1.51	99.80	99.80	718534.46	89723.86
	2002	79.66	69.56	10.10	44883.83	2262.07	9.37	49.87	15.20	69.52	6.51	2.86	79.89	18.59	1.52	99.80	99.80	908686.15	89807.84
	2003	79.84	69.72	10.12	45239.81	2690.51	9.54	48.71	14.53	70.17	6.69	2.85	79.97	18.68	1.53	99.80	99.80	951373.04	89892.02
	2004	80.05	69.90	10.15	46170.92	3062.67	9.56	49.27	14.62	70.32	6.72	2.84	80.05	18.80	1.53	99.80	99.80	1040136.77	89966.05
	2005	80.23	70.06	10.18	47181.56	3474.44	9.57	49.11	14.62	70.24	6.72	2.85	80.12	18.96	1.54	99.80	99.80	849798.74	90042.56
	2006	80.48	70.23	10.24	48035.04	3945.46	9.75	49.42	15.01	69.62	6.79	2.96	80.21	19.15	1.59	99.80	99.80	894922.55	90162.74
	2007	80.65	70.36	10.29	48552.70	4382.43	9.83	49.39	70.09	70.09	6.89	2.94	80.40	19.37	1.66	99.80	99.80	892066.85	90275.20
	2008	80.87	70.51	10.36	48510.57	4682.04	10.03	49.33	14.60	70.41	7.06	2.97	80.58	19.65	1.68	99.80	99.80	908926.00	90380.99
	2009	81.12	70.68	10.44	46543.79	4582.08	11.17	48.78	14.23	70.83	7.91	3.26	80.76	20.00	1.67	99.80	99.80	749863.65	90505.97
	2010	81.34	70.81	10.53	47447.48	5347.69	11.20	49.22	14.58	70.39	7.88	3.32	80.94	20.46	1.63	99.80	99.80	764137.68	90643.60
	2011	81.53	70.96	10.57	48456.96	5665.42	10.82	48.17	14.00	70.94	7.68	3.14	81.12	20.98	1.61	99.80	99.80	1033481.98	90628.15
	2012	81.66	71.06	10.60	48724.25	5719.03	10.78	47.36	13.74	70.98	7.65	3.13	81.29	21.58	1.61	99.80	99.80	1027063.85	90637.18
	2013	81.75	71.12	10.63	49355.10	5619.38	10.67	47.09	13.64	71.03	7.58	3.09	81.47	22.27	1.59	99.80	99.80	-	90642.00

	2014	81.79	71.16	10.64	50067.04	5291.75	10.45	46.79	13.60	70.93	7.41	3.04	81.65	23.01	1.60	99.80	99.80	-	90653.24
	2015	81.77	71.14	10.63	50109.88	-	-	-	-	-	-	-	81.83	23.79	1.60	99.80	99.80	-	90652.67
	2016	81.85	71.20	10.66	50231.89	-	-	-	-	-	-	-	82.01	24.57	-	-	-	-	90655.38
	2017	81.96	70.51	11.45	-	-	-	-	-	-	-	-	-	-	-	-	-	-	-
Cape Verde	1995	67.28	58.45	8.83	1377.42	66.65	5.29	94.01	18.40	80.43	4.25	1.03	48.77	9.15	4.54	34.60	79.90	252.28	87350.31
	1996	68.77	59.74	9.02	1496.61	60.96	4.82	92.90	17.02	81.68	3.94	0.88	49.70	9.30	4.38	35.00	79.90	233.24	87337.77
	1997	68.99	59.94	9.04	1624.70	57.01	4.73	93.00	17.09	81.62	3.86	0.87	50.64	9.42	4.22	37.10	80.50	248.80	87350.49
	1998	69.23	60.16	9.07	1788.00	65.58	5.24	95.23	23.85	74.95	3.93	1.31	51.57	9.53	4.07	39.30	81.10	263.81	87370.97
	1999	69.40	60.32	9.08	1946.85	64.61	4.65	95.19	25.06	73.67	3.42	1.22	52.51	9.64	3.91	41.50	81.80	274.24	87400.57
	2000	69.81	60.66	9.15	2179.98	59.11	4.81	95.16	25.45	73.25	3.52	1.29	53.44	9.78	3.76	43.60	82.40	333.36	87437.90
	2001	70.06	60.90	9.16	2185.25	65.32	5.19	95.05	23.36	75.43	3.91	1.27	54.29	9.91	3.62	45.70	83.00	301.76	87495.04
	2002	70.30	61.12	9.18	2257.32	70.58	5.17	95.06	23.52	75.26	3.89	1.28	55.15	10.04	3.48	47.80	83.70	309.04	87560.29
	2003	70.54	61.34	9.20	2310.49	87.92	5.00	95.04	23.78	74.98	3.75	1.25	56.00	10.15	3.36	49.90	84.30	317.11	87605.64
	2004	70.77	61.55	9.22	2505.17	99.75	5.06	95.05	23.89	74.87	3.79	1.27	56.85	10.20	3.24	52.00	85.00	335.59	87672.43
	2005	71.00	61.76	9.24	2639.38	103.62	4.86	95.01	23.63	75.13	3.65	1.21	57.69	10.17	3.13	54.00	85.60	362.76	87723.68
	2006	71.26	62.02	9.24	2813.19	126.41	5.11	94.98	21.40	77.47	3.96	1.15	58.53	9.99	3.02	56.10	86.30	-	87752.03
	2007	71.21	61.98	9.23	3202.38	135.32	4.30	94.51	78.72	78.72	3.39	0.92	59.36	9.77	2.93	58.10	86.90	-	87765.68
	2008	71.83	62.52	9.31	3378.65	146.55	3.96	94.69	22.11	76.65	3.04	0.93	60.19	9.51	2.83	60.20	87.60	369.12	87824.56
	2009	72.05	62.72	9.34	3300.55	149.23	4.24	93.74	22.17	76.35	3.24	1.00	61.02	9.24	2.75	62.20	88.30	376.40	87866.44
	2010	72.26	62.93	9.33	3312.83	163.98	4.83	89.79	26.01	71.03	3.43	1.40	61.83	8.95	2.67	64.20	88.90	-	87952.25
	2011	72.51	63.13	9.37	3405.78	173.17	4.60	88.32	24.25	72.54	3.34	1.26	62.62	8.50	2.59	66.20	89.60	-	88021.32
	2012	72.68	63.30	9.38	3403.03	161.16	4.61	88.35	24.33	72.46	3.34	1.27	63.39	8.02	2.53	68.10	90.30	-	88099.05
	2013	72.93	63.53	9.40	3389.94	155.56	4.29	87.89	24.01	72.68	3.12	1.17	64.13	7.57	2.47	70.10	91.00	-	88186.02
	2014	73.17	63.75	9.42	3369.64	173.31	4.76	88.04	22.24	74.74	3.56	1.20	64.84	7.21	2.42	72.00	91.70	-	88271.41
	2015	73.31	63.89	9.42	3364.18	-	-	-	-	-	-	-	65.53	6.95	2.37	72.20	91.70	-	88372.85
	2016	73.59	64.18	9.41	3452.95	-	-	-	-	-	-	-	66.19	6.84	-	-	-	-	88466.37
	2017	75.77	65.85	9.91	-	-	-	-	-	-	-	-	-	-	-	-	-	-	-
Central	1995	46.07	39.76	6.31	431.50	12.05	3.60	93.45	58.08	37.85	1.36	2.24	37.23	7.37	5.62	15.60	60.20	133886.53	92010.86
	1996	45.50	39.30	6.20	404.22	13.25	4.49	93.46	51.36	45.04	2.02	2.47	37.31	7.38	5.59	15.90	60.60	129536.67	92001.73

African	1997	44.98	38.89	6.09	415.79	9.62	3.59	93.49	61.81	33.88	1.22	2.37	37.39	7.35	5.56	16.20	61.10	132321.70	91998.99
	1998	44.80	38.75	6.05	425.64	10.14	3.75	93.19	57.96	37.81	1.42	2.33	37.48	7.30	5.54	16.50	61.60	548910.53	92006.63
	1999	44.59	38.57	6.01	431.47	11.14	4.07	93.44	50.50	45.96	1.87	2.20	37.56	7.23	5.52	16.90	62.00	437192.12	92011.06
	2000	44.37	38.38	5.99	411.94	10.40	4.24	93.01	46.85	49.63	2.10	2.13	37.64	7.14	5.50	17.20	62.50	499622.06	92028.76
	2001	44.30	38.35	5.95	421.66	9.70	3.95	92.56	48.88	47.19	1.86	2.09	37.72	7.15	5.48	17.50	62.90	453849.63	92017.18
	2002	44.43	38.49	5.94	428.48	10.70	4.16	92.66	46.78	49.51	2.06	2.10	37.80	7.13	5.46	17.80	63.40	350970.59	92016.37
	2003	44.55	38.61	5.94	397.81	12.52	4.31	92.94	48.15	48.19	2.08	2.23	37.88	7.09	5.44	18.20	63.80	532900.06	92018.71
	2004	44.69	38.75	5.95	414.03	13.06	4.10	92.68	50.98	44.99	1.85	2.26	37.97	7.03	5.41	18.50	64.30	345419.44	92028.83
	2005	44.94	38.95	5.99	410.41	14.28	4.29	92.44	46.87	49.29	2.11	2.18	38.07	6.94	5.38	18.90	64.80	455529.68	92043.16
	2006	45.25	39.26	5.99	422.44	14.24	3.99	91.89	47.57	48.24	1.92	2.06	38.18	6.97	5.35	19.20	65.20	595439.93	92046.82
	2007	45.66	39.64	6.02	434.25	16.34	4.04	91.36	50.36	50.36	2.04	2.01	38.32	6.97	5.32	19.60	65.70	695449.72	92058.97
	2008	46.27	40.18	6.09	436.07	19.97	4.30	85.08	39.81	53.21	2.29	2.01	38.47	6.95	5.29	19.90	66.20	603473.10	92067.06
	2009	46.89	40.73	6.17	437.60	16.25	3.58	83.30	48.43	41.86	1.50	2.08	38.64	6.90	5.25	20.30	66.70	428273.61	92077.27
	2010	47.41	41.18	6.22	446.44	17.41	3.90	90.49	44.59	50.73	1.98	1.92	38.83	6.82	5.22	20.70	67.20	511442.69	92085.00
	2011	47.97	41.69	6.28	458.33	18.09	3.73	90.48	45.87	49.30	1.84	1.89	39.03	6.90	5.17	21.10	67.70	513289.52	92075.67
	2012	48.55	42.21	6.34	475.67	17.02	3.62	90.46	46.31	48.80	1.77	1.85	39.26	6.96	5.12	21.50	68.20	515134.08	92076.07
	2013	47.87	41.67	6.19	300.48	12.53	3.82	90.49	45.19	50.06	1.91	1.91	39.50	6.98	5.07	21.60	68.30	-	92080.16
	2014	48.25	42.02	6.23	302.55	15.59	4.20	90.59	46.22	48.97	2.06	2.14	39.76	6.98	5.01	21.70	68.40	-	92085.77
	2015	49.63	43.19	6.44	314.93	-	-	-	-	-	-	-	40.04	6.96	4.94	21.80	68.50	-	92102.74
	2016	50.16	43.65	6.51	325.72	-	-	-	-	-	-	-	40.33	6.92	-	-	-	-	92113.23
	2017	51.87	44.75	7.12	-	-	-	-	-	-	-	-	-	-	-	-	-	-	-
Chad	1995	53.45	46.08	7.37	484.76	11.95	5.79	96.48	62.94	34.77	2.01	3.77	21.47	6.25	7.43	8.80	42.30	17935.69	88173.82
	1996	52.99	45.72	7.27	479.04	12.01	5.41	96.18	61.32	36.24	1.96	3.45	21.51	6.21	7.43	9.00	42.80	17780.53	88164.11
	1997	52.72	45.50	7.22	489.10	11.45	5.56	96.18	62.19	35.34	1.96	3.59	21.54	6.16	7.42	9.20	43.30	18882.93	88175.28
	1998	52.43	45.26	7.17	505.16	12.17	5.41	96.21	66.71	30.66	1.66	3.75	21.57	6.09	7.41	9.40	43.80	110317.97	88190.91
	1999	52.36	45.20	7.16	484.08	11.19	5.87	96.22	63.58	33.92	1.99	3.88	21.60	6.01	7.38	9.50	44.30	123674.32	88208.18
	2000	52.15	44.98	7.17	462.53	10.42	6.28	96.20	55.32	42.50	2.67	3.61	21.64	5.93	7.35	9.70	44.70	129247.65	88232.80
	2001	52.43	45.23	7.20	497.35	12.01	6.09	94.59	57.34	39.38	2.40	3.69	21.67	5.89	7.32	9.90	45.20	104127.31	88185.25
	2002	52.59	45.37	7.21	519.28	18.40	8.33	94.39	63.90	32.31	2.69	5.64	21.70	5.83	7.27	10.10	45.70	127184.57	88146.61

	2003	52.76	45.52	7.24	573.33	16.06	5.49	91.73	53.83	41.31	2.27	3.22	21.74	5.77	7.21	10.30	46.20	132429.25	88105.56
	2004	53.11	45.82	7.29	737.99	26.01	5.72	92.83	55.51	40.20	2.30	3.42	21.77	5.69	7.15	10.50	46.70	125121.30	88074.08
	2005	53.85	46.41	7.44	835.20	25.81	3.91	90.98	54.73	39.84	1.56	2.35	21.80	5.61	7.07	10.70	47.20	123870.95	88040.45
	2006	54.05	46.64	7.41	812.01	23.65	3.32	89.32	58.96	33.99	1.13	2.19	21.83	5.56	6.99	10.80	47.70	127646.75	87974.14
	2007	54.88	47.37	7.51	811.02	26.82	3.35	88.73	37.36	37.36	1.25	2.10	21.87	5.51	6.90	11.00	48.20	128596.50	87916.02
	2008	55.18	47.66	7.52	808.89	27.10	2.92	87.61	57.67	34.17	1.00	1.92	21.90	5.44	6.80	11.20	48.70	119558.38	87848.99
	2009	55.91	48.30	7.61	815.97	26.58	3.31	87.07	54.64	37.24	1.23	2.08	21.93	5.35	6.70	11.40	49.20	102031.54	87812.43
	2010	56.48	48.80	7.68	896.57	26.42	2.95	85.86	51.35	40.19	1.19	1.76	21.98	5.26	6.59	11.60	49.70	109130.42	87776.77
	2011	56.96	49.24	7.72	868.00	31.32	3.17	86.33	44.51	48.44	1.53	1.63	22.05	5.20	6.48	11.80	50.20	109463.64	87754.12
	2012	57.53	49.73	7.79	914.12	29.18	3.00	86.33	45.42	47.39	1.42	1.58	22.13	5.12	6.37	12.00	50.70	109796.02	87736.26
	2013	58.16	50.28	7.88	934.70	33.67	3.42	86.33	41.50	51.93	1.77	1.64	22.23	5.05	6.26	12.00	50.80	-	87731.38
	2014	58.67	50.73	7.95	967.10	37.10	3.62	86.33	39.15	54.65	1.98	1.64	22.34	4.98	6.16	12.00	50.80	-	87736.47
	2015	59.18	51.16	8.02	953.59	-	-	-	-	-	-	-	22.47	4.93	6.05	12.10	50.80	-	87732.48
	2016	59.83	51.72	8.11	859.65	-	-	-	-	-	-	-	22.62	4.93	-	-	-	-	87729.91
	2017	60.03	51.48	8.55	-	-	-	-	-	-	-	-	-	-	-	-	-	-	-
Chile	1995	75.41	65.88	9.53	8051.49	266.97	5.15	62.31	48.49	48.18	2.48	4.01	84.55	10.56	2.31	88.30	92.70	66432.85	88718.66
	1996	75.71	66.10	9.61	8479.88	303.31	5.66	63.56	49.44	47.17	2.67	4.40	84.86	10.74	2.27	89.00	93.20	73626.89	88745.04
	1997	76.19	66.46	9.73	8987.62	333.68	5.78	66.54	53.53	47.14	2.72	4.65	85.17	10.92	2.23	89.70	93.60	82392.69	88782.49
	1998	76.56	66.74	9.83	9254.79	341.34	6.23	66.09	52.32	48.11	3.00	4.94	85.48	11.10	2.18	90.30	94.10	91627.89	88846.22
	1999	76.89	66.98	9.91	9101.00	318.93	6.40	66.26	51.07	49.86	3.19	4.93	85.78	11.27	2.14	91.00	94.50	97091.05	88931.19
	2000	77.27	67.26	10.01	9469.11	326.16	6.40	65.53	47.00	52.10	3.33	4.59	86.07	11.42	2.11	91.70	94.90	90384.58	89028.20
	2001	77.47	67.41	10.06	9666.48	299.81	6.51	65.90	46.59	53.54	3.48	4.60	86.36	11.57	2.07	92.30	95.30	90862.19	89201.19
	2002	77.70	67.61	10.09	9852.83	290.11	6.48	66.81	47.39	54.51	3.53	4.59	86.65	11.70	2.05	92.90	95.60	91214.15	89372.09
	2003	77.90	67.79	10.11	10141.73	352.96	7.34	66.97	42.45	36.62	2.69	4.65	86.92	11.84	2.02	93.60	96.00	94236.30	89535.28
	2004	78.11	67.97	10.14	10754.31	430.31	6.93	67.14	41.59	38.05	2.64	4.30	87.19	12.00	2.00	94.20	96.30	99878.51	89718.80
	2005	78.42	68.26	10.17	11249.87	508.88	6.69	67.30	41.79	37.90	2.54	4.15	87.44	12.20	1.98	94.80	96.70	101594.28	89894.05
	2006	78.74	68.51	10.23	11833.95	584.73	6.18	67.18	39.82	40.73	2.52	3.66	87.69	12.42	1.96	95.40	97.00	101965.01	89985.50
	2007	78.98	68.67	10.31	12285.05	664.98	6.35	65.79	41.85	41.85	2.66	3.69	87.93	12.67	1.95	96.00	97.30	113127.23	90057.17
	2008	79.26	68.88	10.38	12588.69	739.62	6.87	64.27	36.74	42.84	2.94	3.93	88.16	12.95	1.93	96.60	97.60	111021.04	90129.22

	2009	79.26	68.88	10.38	12268.44	754.59	7.39	63.70	33.82	46.91	3.47	3.92	88.38	13.23	1.90	97.10	97.90	110404.53	90199.49
	2010	79.19	68.87	10.32	12860.18	891.12	6.97	63.86	33.66	47.28	3.29	3.67	88.59	13.52	1.88	97.70	98.20	114285.00	90258.01
	2011	79.52	69.12	10.40	13518.77	1023.22	7.00	63.98	33.54	47.58	3.33	3.67	88.79	13.81	1.86	98.30	98.50	117661.68	90300.40
	2012	79.67	69.25	10.42	14109.14	1108.01	7.24	62.80	32.64	48.03	3.48	3.76	88.99	14.09	1.83	98.80	98.70	120687.89	90359.31
	2013	79.86	69.41	10.45	14551.04	1192.05	7.53	62.43	32.32	48.24	3.63	3.90	89.18	14.39	1.82	99.00	99.00	-	90425.04
	2014	80.10	69.61	10.49	14701.95	1137.36	7.79	62.37	31.52	49.47	3.85	3.93	89.36	14.74	1.80	99.00	99.00	-	90496.31
	2015	80.21	69.72	10.49	14907.12	-	-	-	-	-	-	-	89.53	15.16	1.79	99.10	99.00	-	90579.00
	2016	80.32	69.90	10.42	15019.63	-	-	-	-	-	-	-	89.70	15.63	-	-	-	-	90657.17
	2017	79.70	68.69	11.01	-	-	-	-	-	-	-	-	-	-	-	-	-	-	-
China	1995	68.29	61.08	7.21	1227.56	21.02	3.53	93.74	46.40	50.50	1.78	1.75	30.96	9.28	1.64	53.20	73.90	5042349.12	87074.06
	1996	68.62	61.38	7.24	1335.36	26.32	3.79	94.82	50.64	46.59	1.76	2.02	31.92	9.46	1.57	54.30	75.20	5181880.51	87013.86
	1997	68.92	61.65	7.27	1443.77	30.92	4.02	94.77	52.84	44.24	1.78	2.24	32.88	9.64	1.53	55.40	76.50	5113706.85	86985.88
	1998	69.25	61.94	7.31	1542.06	35.40	4.33	94.26	54.85	41.81	1.81	2.52	33.87	9.81	1.50	56.60	77.80	5141402.70	86991.70
	1999	69.61	62.25	7.36	1645.99	38.72	4.49	94.48	55.85	40.89	1.83	2.65	34.87	9.97	1.49	57.70	79.10	5079293.70	87042.92
	2000	69.94	62.55	7.40	1771.74	43.63	4.60	95.56	58.98	38.28	1.76	2.84	35.88	10.09	1.50	58.80	80.30	5082325.60	87154.72
	2001	70.22	62.80	7.42	1905.61	47.54	4.56	93.07	59.97	35.57	1.62	2.94	37.09	10.23	1.51	60.00	81.60	5145521.55	87271.05
	2002	70.52	63.08	7.44	2065.72	54.47	4.79	89.96	57.72	35.83	1.71	3.07	38.43	10.35	1.52	61.20	82.80	5474914.90	87422.80
	2003	71.22	63.65	7.57	2258.91	61.59	4.82	87.61	55.87	36.23	1.75	3.07	39.78	10.45	1.54	62.40	84.00	6226962.50	87593.94
	2004	71.78	64.12	7.66	2472.59	70.62	4.72	86.48	53.64	37.97	1.79	2.93	41.14	10.54	1.55	63.70	85.20	7116642.40	87768.14
	2005	72.26	64.55	7.71	2738.21	80.94	4.66	85.26	52.21	38.77	1.81	2.85	42.52	10.63	1.57	64.90	86.30	7803535.60	87951.56
	2006	72.88	65.05	7.84	3069.30	94.05	4.52	83.09	49.31	40.65	1.84	2.68	43.87	10.74	1.57	66.10	87.40	8573395.40	88118.91
	2007	73.38	65.40	7.98	3487.85	115.29	4.32	83.01	46.93	46.93	2.03	2.29	45.20	10.86	1.58	67.20	88.50	9124046.50	88281.41
	2008	73.60	65.56	8.04	3805.03	157.68	4.59	80.76	40.42	49.95	2.29	2.30	46.54	11.00	1.58	68.40	89.50	10022024.8	88442.79
	2009	74.12	65.97	8.14	4142.04	192.52	5.08	78.86	37.46	52.50	2.66	2.41	47.88	11.17	1.59	69.60	90.50	10617264.9	88602.77
	2010	74.42	66.25	8.17	4560.51	220.08	4.89	77.24	35.29	54.31	2.65	2.23	49.23	11.39	1.59	70.80	91.40	11183810.6	88777.50
	2011	74.87	66.57	8.30	4971.54	279.48	5.03	78.83	34.77	55.89	2.81	2.22	50.57	11.66	1.59	71.90	92.30	12064260.0	88855.21
	2012	75.25	66.88	8.36	5336.06	328.66	5.26	77.97	34.34	55.96	2.95	2.32	51.89	11.95	1.60	73.10	93.20	12454710.6	88948.62
	2013	75.52	67.11	8.41	5721.69	375.14	5.39	76.66	33.88	55.81	3.01	2.38	53.17	12.30	1.60	74.20	94.00	-	89051.05
	2014	75.68	67.25	8.43	6108.24	419.73	5.55	72.35	31.99	55.79	3.10	2.45	54.41	12.75	1.61	75.40	94.80	-	89164.68

	2015	76.04	67.53	8.51	6496.62	-	-	-	-	-	-	-	55.61	13.32	1.62	76.50	95.50	-	89293.00
	2016	76.36	67.85	8.51	6893.78	-	-	-	-	-	-	-	56.78	14.03	-	-	-	-	89442.77
	2017	77.07	68.09	8.98	-	-	-	-	-	-	-	-	-	-	-	-	-	-	-
Colombia	1995	71.93	63.68	8.25	4841.37	166.93	6.76	84.71	38.06	55.06	3.72	3.04	70.52	7.18	2.66	72.00	89.20	179102.11	87594.12
	1996	72.11	63.84	8.27	4862.07	208.36	8.16	85.38	32.36	62.10	5.07	3.09	70.83	7.23	2.60	72.50	89.40	183442.78	87662.89
	1997	72.64	64.28	8.36	4951.25	247.20	8.96	85.90	32.25	62.46	5.59	3.36	71.15	7.28	2.54	73.10	89.50	188061.88	87751.06
	1998	73.00	64.57	8.42	4904.75	219.11	8.73	80.34	24.15	69.94	6.10	2.62	71.46	7.33	2.48	73.60	89.60	165331.70	87842.52
	1999	73.00	64.59	8.42	4629.50	187.95	8.67	72.36	18.46	74.48	6.46	2.21	71.77	7.39	2.43	74.10	89.70	149560.77	87934.11
	2000	73.19	64.76	8.44	4764.17	146.01	5.91	59.04	12.22	79.30	4.68	1.22	72.08	7.44	2.39	74.60	89.90	164520.43	88039.16
	2001	73.33	64.87	8.46	4774.98	142.81	5.96	59.10	12.59	78.70	4.69	1.27	72.38	7.51	2.35	75.10	90.00	153808.68	88171.81
	2002	73.54	65.05	8.49	4825.83	133.59	5.67	55.86	11.03	80.26	4.55	1.12	72.68	7.59	2.31	75.60	90.10	148233.52	88312.52
	2003	74.32	65.70	8.61	4945.96	132.98	5.92	47.17	8.15	82.72	4.90	1.02	72.98	7.67	2.27	76.10	90.20	162980.74	88441.86
	2004	74.54	65.90	8.63	5139.98	147.18	5.37	60.50	14.51	76.02	4.08	1.29	73.28	7.76	2.24	76.60	90.30	174608.71	88586.84
	2005	74.99	66.31	8.67	5312.08	196.93	5.82	65.94	17.01	74.21	4.32	1.50	73.58	7.86	2.20	77.10	90.50	150617.80	88723.23
	2006	75.18	66.52	8.67	5596.73	226.69	6.11	71.63	18.48	74.20	4.54	1.58	73.88	7.98	2.16	77.50	90.60	151546.74	88788.47
	2007	75.79	66.98	8.81	5910.29	292.30	6.25	72.47	72.59	72.59	4.54	1.71	74.17	8.11	2.12	78.00	90.70	177034.10	88856.92
	2008	76.02	67.18	8.84	6048.09	359.71	6.62	73.69	21.39	70.97	4.70	1.92	74.46	8.26	2.08	78.40	90.80	167450.09	88935.15
	2009	76.15	67.29	8.85	6078.32	361.03	7.01	70.22	18.67	73.42	5.15	1.86	74.75	8.44	2.04	78.90	90.90	171912.03	89009.50
	2010	76.45	67.59	8.86	6250.65	422.39	6.76	67.37	17.76	73.63	4.98	1.78	75.04	8.66	2.01	79.30	91.00	166695.87	89117.16
	2011	77.03	68.03	9.01	6592.40	479.62	6.64	64.03	15.63	75.59	5.02	1.62	75.32	8.91	1.98	79.80	91.10	170199.58	89166.91
	2012	77.33	68.27	9.06	6789.53	546.71	6.93	60.94	14.55	76.12	5.28	1.66	75.60	9.19	1.95	80.20	91.20	173411.77	89214.74
	2013	77.70	68.56	9.13	7051.04	549.37	6.84	58.10	13.79	76.26	5.22	1.62	75.88	9.51	1.92	80.60	91.30	-	89290.65
	2014	77.90	68.73	9.17	7291.69	569.19	7.20	61.73	15.36	75.12	5.41	1.79	76.16	9.86	1.90	81.10	91.30	-	89384.09
	2015	78.07	68.88	9.19	7446.18	-	-	-	-	-	-	-	76.44	10.25	1.87	81.10	91.40	-	89493.62
	2016	78.26	69.09	9.17	7525.86	-	-	-	-	-	-	-	76.71	10.66	-	-	-	-	89593.79
	2017	80.11	70.40	9.71	-	-	-	-	-	-	-	-	-	-	-	-	-	-	-
Comoros	1995	59.96	52.45	7.51	782.97	22.31	4.61	100.00	37.58	62.42	2.88	1.73	28.30	6.00	5.84	21.30	90.10	317.95	89028.27
	1996	59.23	51.87	7.36	751.92	20.95	4.48	100.00	39.42	60.58	2.71	1.77	28.26	5.97	5.74	22.10	90.10	325.15	89042.12
	1997	60.15	52.65	7.50	761.46	18.81	4.49	100.00	42.46	57.54	2.58	1.91	28.21	5.92	5.64	22.80	90.10	331.39	89069.98

	1998	60.16	52.65	7.50	751.13	16.75	4.04	100.00	45.97	54.03	2.19	1.86	28.17	5.87	5.55	23.60	90.10	341.63	89100.64
	1999	61.23	53.54	7.69	746.09	16.13	3.87	100.00	49.21	50.79	1.96	1.90	28.12	5.80	5.47	24.30	90.10	355.47	89139.23
	2000	61.46	53.73	7.73	806.43	13.11	3.56	100.00	57.17	42.83	1.52	2.03	28.08	5.73	5.38	25.10	90.10	365.90	89181.46
	2001	62.36	54.52	7.84	805.16	11.21	2.86	100.00	61.72	38.28	1.09	1.76	28.04	5.70	5.31	25.80	90.10	423.63	89160.69
	2002	62.68	54.83	7.85	804.21	14.53	3.39	100.00	50.71	49.29	1.67	1.72	27.99	5.66	5.24	26.60	90.10	406.27	89162.93
	2003	63.50	55.55	7.95	801.80	19.66	3.65	100.00	50.26	49.74	1.82	1.83	27.95	5.61	5.17	27.40	90.10	387.33	89165.97
	2004	63.96	55.96	7.99	798.01	24.14	3.96	100.00	49.22	50.78	2.01	1.95	27.90	5.55	5.10	28.10	90.10	389.63	89187.31
	2005	64.28	56.29	7.98	801.33	26.54	4.32	100.00	47.32	52.68	2.27	2.04	27.87	5.48	5.04	28.90	90.10	480.84	89215.11
	2006	64.53	56.55	7.98	803.11	29.29	4.57	100.00	45.16	54.84	2.51	2.06	27.86	5.46	4.98	29.60	90.10	385.28	89215.15
	2007	64.69	56.68	8.01	790.37	35.11	4.93	100.00	56.85	56.85	2.80	2.13	27.85	5.43	4.93	30.40	90.10	513.65	89212.17
	2008	65.28	57.21	8.08	774.68	40.34	5.13	100.00	39.68	60.32	3.09	2.04	27.86	5.39	4.87	31.20	90.10	579.09	89246.03
	2009	64.90	56.91	7.99	770.99	29.33	3.82	100.00	54.89	45.11	1.72	2.09	27.88	5.34	4.81	31.90	90.10	531.67	89279.68
	2010	65.92	57.81	8.11	769.17	44.23	5.82	47.96	35.99	24.97	1.45	4.37	27.92	5.27	4.75	32.70	90.10	543.62	89343.76
	2011	65.61	57.56	8.05	770.32	49.93	6.10	67.12	48.88	27.17	1.66	4.44	27.97	5.23	4.69	33.50	90.10	554.34	89314.14
	2012	66.20	58.07	8.13	774.47	55.30	7.11	67.12	42.12	37.24	2.65	4.46	28.03	5.18	4.63	34.20	90.10	564.22	89335.42
	2013	66.51	58.36	8.16	782.52	53.57	6.51	67.12	46.46	30.77	2.00	4.51	28.10	5.13	4.56	35.00	90.10	-	89349.14
	2014	66.95	58.73	8.21	779.84	56.76	6.75	67.12	45.06	32.86	2.22	4.53	28.19	5.09	4.49	35.80	90.10	-	89371.57
	2015	67.25	59.00	8.25	769.48	-	-	-	-	-	-	-	28.30	5.08	4.42	35.80	90.10	-	89412.14
	2016	67.54	59.29	8.25	768.44	-	-	-	-	-	-	-	28.41	5.12	-	-	-	-	89431.28
	2017	68.63	59.97	8.66	-	-	-	-	-	-	-	-	-	-	-	-	-	-	-
Congo	1995	52.65	45.67	6.98	381.72	4.35	3.25	81.39	77.60	4.66	0.15	3.10	32.84	5.74	6.78	20.30	45.20	1232299.89	90279.79
	1996	52.28	45.39	6.90	367.43	4.03	3.03	82.74	79.66	3.73	0.11	2.92	33.29	5.77	6.78	20.80	45.60	1050676.58	90269.37
	1997	46.54	40.68	5.86	338.41	4.95	3.62	82.39	77.72	5.66	0.20	3.41	33.74	5.78	6.77	21.30	46.00	998227.15	90276.87
	1998	50.16	43.62	6.54	325.41	5.81	4.26	84.44	80.91	4.18	0.18	4.09	34.20	5.78	6.77	21.80	46.40	1014212.24	90288.83
	1999	51.94	45.08	6.86	304.34	10.48	3.80	83.24	77.82	6.51	0.25	3.56	34.66	5.76	6.76	22.20	46.80	824119.40	90294.57
	2000	52.75	45.72	7.03	276.22	18.16	1.45	56.51	54.76	3.09	0.04	1.40	35.12	5.73	6.75	22.70	47.10	861822.71	90294.45
	2001	53.66	46.50	7.16	263.05	5.82	2.67	75.26	71.19	5.41	0.14	2.52	35.59	5.76	6.74	23.10	47.50	678319.48	90257.79
	2002	54.39	47.11	7.27	262.97	4.43	2.59	75.49	70.82	6.19	0.16	2.43	36.06	5.78	6.73	23.50	47.90	763919.42	90240.55
	2003	54.90	47.57	7.33	269.24	5.44	3.20	74.88	58.52	21.85	0.70	2.50	36.53	5.79	6.72	24.00	48.20	748084.20	90223.51

	2004	55.72	48.29	7.43	278.48	6.33	3.34	77.17	60.58	21.51	0.72	2.62	37.00	5.79	6.71	24.40	48.60	816309.43	90215.13
	2005	56.49	48.94	7.55	286.29	6.71	3.14	75.18	57.19	23.93	0.75	2.39	37.48	5.78	6.69	24.80	49.00	794528.22	90223.04
	2006	57.34	49.68	7.66	291.97	8.36	3.39	76.28	55.12	27.73	0.94	2.45	37.96	5.82	6.67	25.20	49.30	922488.68	90214.62
	2007	57.84	50.10	7.73	300.29	9.87	3.61	75.39	33.50	33.50	1.21	2.40	38.45	5.84	6.65	25.60	49.70	895387.99	90195.88
	2008	58.56	50.73	7.83	308.65	13.85	4.46	74.05	39.17	47.11	2.10	2.36	38.94	5.86	6.62	26.00	50.00	754085.86	90176.27
	2009	59.44	51.47	7.97	307.11	16.06	5.61	64.82	37.53	42.10	2.36	3.25	39.44	5.87	6.59	26.40	50.40	731884.63	90159.98
	2010	60.27	52.18	8.09	318.08	12.58	4.04	50.34	37.11	26.28	1.06	2.98	39.94	5.88	6.54	26.80	50.70	799228.67	90151.12
	2011	60.96	52.78	8.18	328.75	12.38	3.53	60.20	38.82	35.51	1.26	2.28	40.44	5.92	6.49	27.20	51.10	800756.94	90089.99
	2012	61.40	53.19	8.21	340.71	14.71	3.77	68.29	37.69	44.81	1.69	2.08	40.95	5.94	6.43	27.60	51.40	802271.30	90060.09
	2013	61.84	53.60	8.24	357.57	16.08	3.89	59.73	39.39	34.05	1.32	2.56	41.46	5.96	6.36	27.90	51.80	-	90019.74
	2014	62.27	54.00	8.27	378.79	19.05	4.33	61.47	38.77	36.93	1.60	2.73	41.98	5.97	6.29	28.30	52.10	-	90017.91
	2015	62.53	54.26	8.27	391.72	-	-	-	-	-	-	-	42.49	5.96	6.20	28.70	52.40	-	89994.77
	2016	62.89	54.63	8.25	388.27	-	-	-	-	-	-	-	43.02	5.97	-	-	-	-	89987.85
	2017	62.65	54.03	8.62	-	-	-	-	-	-	-	-	-	-	-	-	-	-	-
Costa Rica	1995	76.96	67.99	8.97	5651.68	217.31	6.51	87.68	20.60	76.50	4.98	1.53	54.56	8.42	2.82	90.00	94.00	10054.85	87313.52
	1996	77.05	68.07	8.98	5585.14	219.02	6.65	87.50	20.86	76.16	5.07	1.59	55.47	8.53	2.74	90.30	94.20	9907.29	87423.03
	1997	77.23	68.21	9.02	5758.28	221.23	6.35	87.12	20.74	76.20	4.84	1.51	56.37	8.63	2.65	90.60	94.40	9924.61	87534.20
	1998	77.48	68.41	9.07	6020.76	266.92	7.13	87.58	18.36	79.04	5.64	1.49	57.26	8.74	2.56	90.90	94.70	10575.34	87637.39
	1999	77.72	68.59	9.13	6125.90	280.36	6.83	88.23	18.96	78.51	5.36	1.47	58.15	8.85	2.46	91.20	94.90	10338.55	87776.10
	2000	77.96	68.77	9.19	6230.48	289.41	7.12	88.17	18.71	78.78	5.61	1.51	59.05	8.96	2.37	91.40	95.10	9851.27	87899.33
	2001	78.22	68.97	9.24	6332.84	297.42	7.25	89.88	21.84	75.69	5.49	1.76	60.41	9.11	2.29	91.70	95.30	10917.34	88010.86
	2002	78.47	69.18	9.30	6434.39	339.93	8.23	90.90	21.46	76.39	6.28	1.94	61.75	9.26	2.22	92.00	95.50	9998.17	88141.93
	2003	78.74	69.38	9.36	6606.16	358.12	8.45	89.69	21.22	76.34	6.45	2.00	63.08	9.43	2.16	92.30	95.80	10415.84	88264.04
	2004	79.04	69.61	9.43	6792.11	346.18	7.80	90.50	24.89	72.49	5.65	2.15	64.39	9.60	2.11	92.60	96.00	10571.67	88399.95
	2005	79.31	69.84	9.47	6954.04	364.01	7.74	84.52	24.83	70.62	5.47	2.27	65.67	9.79	2.07	92.80	96.20	10095.24	88525.96
	2006	79.59	70.05	9.54	7351.86	409.93	7.82	86.68	26.88	68.99	5.40	2.43	66.93	9.99	2.03	93.00	96.40	10715.12	88610.29
	2007	79.91	70.28	9.64	7841.93	524.55	8.37	87.73	67.24	67.24	5.63	2.74	68.17	10.19	2.01	93.30	96.60	11298.03	88699.94
	2008	80.12	70.43	9.69	8095.31	606.60	9.01	88.95	27.10	69.53	6.26	2.74	69.39	10.41	1.98	93.50	96.80	10806.54	88784.85
	2009	80.27	70.54	9.74	7911.80	634.14	9.69	89.65	24.66	72.49	7.02	2.66	70.57	10.67	1.95	93.70	97.00	11067.75	88886.83

	2010	80.37	70.63	9.74	8199.41	771.24	9.66	90.47	24.03	73.44	7.09	2.56	71.73	10.97	1.92	93.90	97.20	11684.57	88998.16
	2011	80.50	70.71	9.79	8449.96	872.11	9.73	90.99	24.13	73.49	7.15	2.58	72.87	11.31	1.89	94.10	97.30	11994.69	89090.21
	2012	80.57	70.77	9.80	8753.23	930.48	9.56	90.98	24.36	73.23	7.00	2.56	73.94	11.69	1.87	94.30	97.50	12274.13	89179.40
	2013	80.67	70.86	9.81	8852.40	990.35	9.47	90.98	24.53	73.04	6.91	2.55	74.96	12.10	1.84	94.50	97.60	-	89287.98
	2014	80.77	70.94	9.83	9077.41	970.00	9.31	90.98	24.87	72.67	6.77	2.55	75.92	12.52	1.82	94.50	97.70	-	89404.05
	2015	80.89	71.04	9.85	9406.76	-	-	-	-	-	-	-	76.82	12.94	1.80	94.50	97.80	-	89530.01
	2016	80.98	71.16	9.82	9714.10	-	-	-	-	-	-	-	77.68	13.34	-	-	-	-	89654.52
	2017	79.47	69.83	9.64	-	-	-	-	-	-	-	-	-	-	-	-	-	-	-
Cote d'Ivoire	1995	52.54	45.45	7.09	1314.12	48.64	6.37	81.63	60.54	25.84	1.65	4.72	41.21	5.15	6.22	16.20	76.90	147447.84	89351.85
	1996	52.46	45.44	7.01	1372.79	55.09	6.74	81.64	59.18	27.51	1.85	4.89	41.59	5.17	6.15	16.40	77.10	145020.63	89374.77
	1997	52.53	45.54	7.00	1382.62	54.15	7.07	83.69	59.96	28.35	2.00	5.06	41.96	5.17	6.08	16.70	77.30	148926.94	89388.96
	1998	52.49	45.53	6.95	1410.74	58.52	7.30	84.29	62.47	25.88	1.89	5.41	42.34	5.15	6.01	17.00	77.50	24962.96	89401.69
	1999	52.44	45.53	6.92	1397.00	50.02	6.52	81.45	58.98	27.60	1.80	4.72	42.89	5.13	5.93	17.30	77.70	24317.16	89403.15
	2000	52.42	45.52	6.90	1336.43	38.94	6.00	78.77	55.30	29.79	1.79	4.21	43.54	5.11	5.86	17.60	77.90	29442.74	89404.21
	2001	52.47	45.75	6.72	1310.29	32.16	4.85	74.98	55.02	26.62	1.29	3.56	44.20	5.16	5.79	17.90	78.20	28584.19	89364.43
	2002	52.72	45.96	6.76	1264.22	32.09	4.47	71.82	49.17	31.54	1.41	3.06	44.85	5.20	5.72	18.20	78.50	28670.94	89333.67
	2003	52.92	46.13	6.79	1224.97	40.71	4.65	74.05	52.75	28.77	1.34	3.31	45.51	5.22	5.65	18.60	78.70	22407.15	89317.60
	2004	53.13	46.30	6.83	1218.12	48.70	5.24	76.57	55.05	28.10	1.47	3.77	46.18	5.22	5.59	18.90	79.00	22931.74	89302.14
	2005	53.51	46.61	6.90	1216.21	50.78	5.39	78.45	59.97	23.56	1.27	4.12	46.84	5.20	5.53	19.20	79.20	26282.74	89289.78
	2006	53.92	46.98	6.94	1210.67	56.53	5.87	80.10	61.57	23.14	1.36	4.51	47.50	5.27	5.48	19.50	79.50	27248.77	89291.50
	2007	54.49	47.46	7.03	1207.09	68.44	6.35	81.05	22.51	22.51	1.43	4.92	48.27	5.32	5.42	19.90	79.80	27859.55	89294.34
	2008	55.04	47.93	7.11	1211.62	78.05	6.21	80.18	60.43	24.63	1.53	4.68	49.03	5.35	5.37	20.20	80.10	29682.24	89300.48
	2009	55.62	48.42	7.20	1223.51	79.10	6.41	78.09	57.43	26.46	1.70	4.72	49.80	5.36	5.32	20.60	80.40	30685.23	89315.18
	2010	56.20	48.92	7.28	1219.75	78.17	6.32	76.85	56.48	26.51	1.68	4.65	50.56	5.34	5.27	20.90	80.70	32525.25	89330.95
	2011	56.79	49.45	7.33	1138.66	79.09	6.42	77.13	57.48	25.48	1.64	4.78	51.31	5.37	5.22	21.30	80.90	33027.48	89444.62
	2012	57.22	49.86	7.36	1229.78	78.72	6.14	75.51	52.34	30.68	1.89	4.26	52.04	5.37	5.16	21.60	81.20	33502.16	89557.85
	2013	57.95	50.50	7.45	1305.71	84.08	5.81	71.85	49.63	30.92	1.80	4.01	52.77	5.35	5.10	22.00	81.50	-	89685.16
	2014	58.48	50.97	7.51	1384.91	88.37	5.72	71.93	50.81	29.36	1.68	4.04	53.48	5.33	5.04	22.30	81.80	-	89825.59
	2015	59.08	51.49	7.60	1469.73	-	-	-	-	-	-	-	54.18	5.30	4.98	22.50	81.90	-	89963.15

	2016	59.87	52.15	7.72	1552.77	-	-	-	-	-	-	-	54.87	5.34	-	-	-	-	90120.55
	2017	62.48	54.15	8.33	-	-	-	-	-	-	-	-	-	-	-	-	-	-	-
Croatia	1995	72.29	63.36	8.93	8475.14	324.13	6.74	100.00	13.54	86.46	5.83	0.91	54.89	20.16	1.58	97.30	98.20	24621.25	92769.09
	1996	72.98	63.96	9.02	9322.35	362.08	7.01	100.00	12.42	87.58	6.14	0.87	55.03	20.83	1.67	97.30	98.20	24101.40	92766.98
	1997	73.19	64.15	9.05	9772.26	304.44	5.79	100.00	15.52	84.48	4.89	0.90	55.17	21.47	1.69	97.30	98.30	26444.91	92792.87
	1998	73.42	64.34	9.08	10111.22	369.31	6.59	100.00	12.96	87.04	5.73	0.85	55.31	22.08	1.45	97.30	98.30	27432.06	92863.28
	1999	73.64	64.52	9.11	9900.06	374.15	7.24	100.00	12.66	87.34	6.33	0.92	55.45	22.65	1.38	97.30	98.30	27203.21	92942.69
	2000	73.91	64.77	9.14	10570.14	371.22	7.66	100.00	13.86	86.14	6.60	1.06	55.59	23.19	1.39	97.30	98.30	27312.05	93014.15
	2001	74.18	64.95	9.22	10898.69	387.77	7.16	86.05	14.39	83.28	5.96	1.20	55.73	23.88	1.46	97.30	98.30	27165.05	93037.29
	2002	74.44	65.15	9.29	11470.48	388.08	6.19	93.38	18.63	80.05	4.96	1.24	55.88	24.53	1.42	97.30	98.40	28301.73	93087.79
	2003	74.70	65.35	9.35	12108.00	512.02	6.35	95.92	16.68	82.61	5.24	1.10	56.04	25.11	1.41	97.30	98.50	30011.88	93123.68
	2004	74.93	65.52	9.41	12605.19	633.90	6.56	95.92	18.17	81.06	5.31	1.24	56.22	25.52	1.43	97.20	98.60	29691.51	93141.86
	2005	75.14	65.70	9.44	13121.18	726.52	6.89	95.92	13.41	86.02	5.93	0.96	56.41	25.73	1.50	97.20	98.70	29863.54	93171.05
	2006	75.36	65.87	9.49	13755.24	813.87	6.95	95.92	13.36	86.07	5.98	0.97	56.61	25.93	1.47	97.20	98.80	30290.07	93157.18
	2007	75.58	66.00	9.58	14476.68	1038.92	7.44	95.92	87.03	87.03	6.48	0.97	56.82	25.97	1.48	97.20	98.90	33301.10	93152.78
	2008	75.84	66.19	9.65	14778.91	1265.14	7.70	95.92	14.51	84.87	6.54	1.17	57.05	25.94	1.55	97.10	99.00	32938.06	93147.76
	2009	76.09	66.37	9.72	13704.44	1193.49	8.18	95.92	13.73	85.69	7.01	1.17	57.29	25.99	1.58	97.10	99.10	31461.04	93161.81
	2010	76.35	66.61	9.75	13505.75	1143.85	8.25	95.92	13.76	85.66	7.06	1.18	57.54	26.21	1.55	97.10	99.20	31172.65	93209.31
	2011	76.63	66.77	9.85	13899.29	1131.67	7.80	60.95	12.06	80.21	6.26	1.54	57.80	26.51	1.48	97.10	99.30	30453.51	93214.04
	2012	76.88	66.98	9.90	13636.87	1029.52	7.80	62.38	11.47	81.61	6.36	1.43	58.07	26.90	1.51	97.10	99.40	30421.05	93228.09
	2013	77.12	67.16	9.96	13529.41	1060.83	7.83	61.80	11.25	81.79	6.40	1.43	58.36	27.39	1.46	97.10	99.50	-	93249.26
	2014	77.30	67.30	10.00	13517.83	1050.33	7.80	61.80	11.21	81.87	6.39	1.41	58.66	27.92	1.46	97.10	99.60	-	93280.18
	2015	77.35	67.35	10.00	13935.95	-	-	-	-	-	-	-	58.96	28.49	1.46	97.00	99.60	-	93315.07
	2016	77.36	67.42	9.94	14465.13	-	-	-	-	-	-	-	59.28	29.26	-	-	-	-	93360.16
	2017	78.53	67.42	11.11	-	-	-	-	-	-	-	-	-	-	-	-	-	-	-
Cuba	1995	75.15	66.03	9.12	2844.12	111.84	5.15	100.00	9.77	90.23	4.65	0.50	74.28	13.51	1.64	84.10	89.60	46584.98	91464.31
	1996	75.40	66.27	9.14	3051.68	119.36	5.26	100.00	9.51	90.49	4.76	0.50	74.43	13.68	1.64	84.60	89.70	48578.11	91532.60
	1997	75.64	66.48	9.16	3121.54	126.06	5.50	100.00	9.32	90.68	4.99	0.51	74.62	13.85	1.63	85.20	89.80	49638.53	91574.79
	1998	75.94	66.74	9.20	3112.35	133.59	5.77	100.00	9.43	90.57	5.23	0.54	74.86	14.01	1.63	85.70	89.90	46514.68	91619.83

	1999	76.22	66.99	9.23	3291.30	152.60	6.00	100.00	8.81	91.19	5.47	0.53	75.09	14.19	1.63	86.20	90.20	45832.24	91688.68
	2000	76.52	67.26	9.27	3473.24	166.33	6.07	100.00	9.17	90.83	5.51	0.56	75.32	14.39	1.62	86.70	90.60	46257.54	91777.01
	2001	76.81	67.49	9.32	3572.40	177.88	6.27	100.00	9.56	90.44	5.67	0.60	75.56	14.63	1.61	87.20	90.90	44507.96	91847.91
	2002	77.06	67.70	9.36	3613.14	194.80	6.50	100.00	11.86	88.14	5.72	0.77	75.79	14.89	1.59	87.70	91.20	48831.04	91946.57
	2003	77.26	67.86	9.39	3741.21	201.88	6.31	100.00	10.55	89.45	5.65	0.67	75.95	15.15	1.58	88.30	91.50	48493.24	92063.31
	2004	77.41	67.99	9.41	3949.35	208.58	6.14	100.00	10.91	89.09	5.47	0.67	76.04	15.43	1.58	88.70	91.80	48579.22	92191.23
	2005	77.57	68.14	9.42	4385.00	356.29	9.39	100.00	6.92	93.08	8.74	0.65	76.13	15.73	1.57	89.20	92.10	45763.45	92338.24
	2006	77.76	68.31	9.45	4908.88	359.99	7.67	100.00	7.69	92.31	7.08	0.59	76.23	16.12	1.57	89.70	92.40	45505.48	92455.41
	2007	77.93	68.42	9.51	5261.90	586.91	10.42	100.00	94.85	94.85	9.88	0.54	76.32	16.53	1.58	90.20	92.70	44012.99	92537.41
	2008	78.08	68.53	9.55	5475.58	584.10	10.79	100.00	4.63	95.37	10.29	0.50	76.41	16.95	1.60	90.70	92.90	48557.44	92583.18
	2009	78.22	68.63	9.59	5550.71	650.55	11.78	100.00	4.22	95.78	11.28	0.50	76.51	17.38	1.62	91.20	93.20	50545.40	92610.60
	2010	78.34	68.75	9.59	5676.14	583.37	10.19	100.00	4.81	95.19	9.70	0.49	76.60	17.80	1.64	91.70	93.50	49782.53	92660.33
	2011	78.49	68.83	9.66	5824.10	645.63	10.60	100.00	4.96	95.04	10.07	0.53	76.69	18.18	1.66	92.20	93.80	51170.75	92671.55
	2012	78.56	68.89	9.67	5985.20	554.72	8.59	100.00	5.85	94.15	8.09	0.50	76.78	18.55	1.69	92.70	94.10	52418.46	92708.33
	2013	78.64	68.95	9.69	6133.47	656.70	9.48	100.00	5.48	94.52	8.96	0.52	76.87	18.93	1.70	93.10	94.30	-	92757.94
	2014	78.72	69.01	9.71	6182.77	816.62	11.06	100.00	4.39	95.61	10.57	0.49	76.97	19.38	1.71	93.20	94.60	-	92821.59
	2015	78.83	69.09	9.74	6444.98	-	-	-	-	-	-	-	77.07	19.92	1.72	93.20	94.90	-	92920.53
	2016	78.91	69.19	9.73	-	-	-	-	-	-	-	-	77.18	20.55	-	-	-	-	93022.67
	2017	78.42	69.15	9.27	-	-	-	-	-	-	-	-	-	-	-	-	-	-	-
Cyprus	1995	75.70	66.25	9.45	24030.24	680.67	4.74	98.61	63.27	35.84	1.70	3.04	68.04	15.45	2.12	100.00	100.00	6594.25	91036.63
	1996	76.10	66.56	9.54	23965.53	744.09	5.21	98.42	64.86	34.10	1.77	3.43	68.16	15.40	2.04	100.00	100.00	6931.65	91162.74
	1997	76.22	66.66	9.56	24234.42	728.27	5.53	98.20	63.60	35.23	1.95	3.58	68.28	15.33	1.95	100.00	100.00	7004.33	91317.48
	1998	76.37	66.78	9.58	25192.43	775.91	5.58	98.16	57.05	41.88	2.34	3.25	68.41	15.27	1.86	100.00	100.00	7355.14	91475.11
	1999	76.62	66.98	9.64	26115.00	787.91	5.60	95.16	54.56	42.66	2.39	3.21	68.53	15.22	1.78	100.00	100.00	7572.95	91620.88
	2000	76.82	67.15	9.67	27317.80	753.35	5.77	95.74	55.86	41.65	2.40	3.37	68.65	15.18	1.72	100.00	100.00	7952.75	91802.28
	2001	77.25	67.48	9.76	27998.02	783.71	5.78	95.73	55.19	42.36	2.45	3.33	68.77	15.18	1.66	100.00	100.00	7790.87	91942.32
	2002	77.46	67.67	9.79	28619.83	894.66	6.12	95.91	52.77	44.98	2.75	3.37	68.69	15.20	1.61	100.00	100.00	8040.81	92076.12
	2003	77.74	67.90	9.84	28974.13	1260.41	6.83	79.14	43.43	45.13	3.08	3.75	68.55	15.28	1.58	100.00	100.00	8896.65	92176.42
	2004	77.91	68.04	9.86	29895.75	1384.67	6.42	82.78	46.49	43.84	2.81	3.60	68.41	15.41	1.55	100.00	100.00	8725.30	92271.50

	2005	78.03	68.16	9.87	30565.78	1448.75	6.37	80.83	47.04	41.81	2.66	3.71	68.27	15.55	1.52	100.00	100.00	8911.67	92363.09
	2006	78.66	68.66	10.00	31415.82	1529.21	6.28	80.99	46.63	42.43	2.66	3.61	68.13	15.65	1.50	100.00	100.00	8761.86	92411.95
	2007	78.93	68.86	10.07	32236.59	1734.48	6.05	83.24	42.59	42.59	2.58	3.47	67.98	15.73	1.48	100.00	100.00	9077.28	92486.53
	2008	79.24	69.11	10.13	32651.91	2241.27	6.89	84.75	49.70	41.36	2.85	4.04	67.84	15.82	1.46	100.00	100.00	9165.95	92545.22
	2009	79.55	69.35	10.20	31223.62	2185.84	7.40	87.43	48.32	44.74	3.31	4.09	67.70	15.95	1.44	100.00	100.00	-	92585.68
	2010	79.87	69.62	10.26	30818.46	2038.20	7.23	86.89	45.37	47.35	3.43	3.78	67.55	16.14	1.42	100.00	100.00	-	92645.09
	2011	80.23	69.89	10.33	30138.59	2235.67	7.54	84.57	44.84	46.54	3.51	4.00	67.41	16.50	1.41	100.00	100.00	7652.33	92690.19
	2012	80.58	70.17	10.40	28745.54	1964.82	7.44	87.67	47.04	45.86	3.41	3.99	67.26	16.90	1.39	100.00	100.00	7431.19	92692.61
	2013	80.64	70.25	10.39	27097.31	1889.87	7.46	88.86	46.97	46.53	3.47	3.94	67.13	17.33	1.37	100.00	100.00	-	92722.66
	2014	80.51	70.18	10.34	26977.63	1819.11	7.37	88.93	48.71	45.23	3.33	4.04	67.02	17.80	1.36	100.00	100.00	-	92757.92
	2015	80.48	70.17	10.31	27587.28	-	-	-	-	-	-	-	66.92	18.26	1.35	100.00	100.00	-	92794.67
	2016	80.48	70.20	10.28	28325.44	-	-	-	-	-	-	-	66.84	18.75	-	-	-	-	92827.26
	2017	81.72	70.68	11.04	-	-	-	-	-	-	-	-	-	-	-	-	-	-	-
Czech Republic	1995	73.36	63.78	9.58	13462.99	374.35	6.69	100.00	9.11	90.89	6.08	0.61	74.64	19.40	1.28	99.10	99.80	150508.56	91612.32
	1996	73.78	64.10	9.67	14047.84	403.12	6.41	100.00	9.33	90.67	5.81	0.60	74.51	19.55	1.18	99.10	99.80	154942.06	91693.35
	1997	74.13	64.39	9.73	13979.36	369.23	6.40	100.00	9.71	90.29	5.78	0.62	74.38	19.69	1.17	99.10	99.80	149454.44	91767.33
	1998	74.47	64.69	9.79	13946.62	397.98	6.42	100.00	9.57	90.43	5.80	0.61	74.25	19.80	1.16	99.10	99.80	146940.02	91831.93
	1999	74.74	64.92	9.82	14160.91	383.60	6.35	100.00	9.52	90.48	5.74	0.60	74.12	19.84	1.13	99.10	99.80	142384.43	91890.21
	2000	75.03	65.22	9.81	14806.58	361.23	6.31	100.00	9.68	90.32	5.70	0.61	73.99	19.78	1.15	99.10	99.80	162021.19	91954.37
	2001	75.27	65.33	9.95	15294.63	404.06	6.42	100.00	10.23	89.77	5.76	0.66	73.88	19.80	1.15	99.10	99.80	157672.17	92006.84
	2002	75.42	65.41	10.01	15577.39	522.58	6.80	100.00	9.53	90.47	6.15	0.65	73.81	19.74	1.17	99.10	99.80	152372.42	92062.89
	2003	75.64	65.54	10.09	16143.26	666.81	7.13	97.67	9.96	89.80	6.40	0.73	73.74	19.66	1.18	99.10	99.80	157836.12	92118.89
	2004	75.92	65.71	10.20	16930.19	771.78	6.90	95.42	10.35	89.15	6.15	0.75	73.67	19.64	1.23	99.10	99.80	152079.89	92166.54
	2005	76.23	65.96	10.27	18011.39	884.36	6.93	84.29	10.70	87.31	6.05	0.88	73.60	19.72	1.29	99.10	99.90	147551.44	92208.45
	2006	76.55	66.19	10.36	19193.75	971.51	6.69	85.11	11.29	86.73	5.81	0.89	73.53	19.99	1.34	99.10	99.90	147768.30	92191.43
	2007	76.85	66.34	10.52	20151.18	1147.98	6.52	89.03	85.19	85.19	5.56	0.97	73.46	20.34	1.45	99.10	99.90	149838.95	92166.03
	2008	77.13	66.52	10.60	20520.78	1486.18	6.82	90.15	15.73	82.55	5.63	1.19	73.39	20.78	1.51	99.10	99.90	144072.58	92150.24
	2009	77.27	66.62	10.65	19424.27	1483.94	7.85	89.96	14.63	83.74	6.57	1.28	73.32	21.31	1.51	99.10	99.90	137877.29	92170.92
	2010	77.50	66.84	10.66	19808.07	1410.04	7.43	91.50	14.86	83.76	6.22	1.21	73.26	21.95	1.51	99.10	100.00	145706.92	92207.20

	2011	77.77	67.00	10.78	20118.59	1545.57	7.50	93.20	14.74	84.18	6.31	1.19	73.19	22.82	1.43	99.10	100.00	144613.25	92215.11
	2012	78.03	67.20	10.82	19929.76	1411.48	7.55	94.06	15.03	84.02	6.34	1.21	73.12	23.78	1.45	99.10	100.00	138957.42	92224.96
	2013	78.30	67.42	10.88	19826.79	1412.79	7.49	92.70	14.53	84.33	6.31	1.17	73.06	24.82	1.46	99.10	100.00	-	92219.18
	2014	78.64	67.67	10.96	20343.68	1378.52	7.41	92.65	14.33	84.54	6.26	1.15	73.02	25.87	1.53	99.10	100.00	-	92219.58
	2015	78.96	67.92	11.04	21381.70	-	-	-	-	-	-	-	72.99	26.91	1.53	99.10	100.00	-	92235.18
	2016	79.10	68.10	11.00	21903.85	-	-	-	-	-	-	-	72.98	27.98	-	-	-	-	92262.67
	2017	79.17	67.40	11.76	-	-	-	-	-	-	-	-	-	-	-	-	-	-	-
Korea	1995	64.32	57.20	7.12	-	-	-	-	-	-	-	-	59.02	7.50	2.12	53.30	100.00	111920.31	84165.22
	1996	64.27	57.17	7.10	-	-	-	-	-	-	-	-	59.10	7.71	2.08	53.30	100.00	100618.40	84285.04
	1997	64.19	57.10	7.09	-	-	-	-	-	-	-	-	59.18	7.91	2.04	55.20	99.90	98661.43	84442.91
	1998	64.07	57.00	7.08	-	-	-	-	-	-	-	-	59.26	8.11	2.01	57.10	99.90	84375.45	84622.97
	1999	63.95	56.89	7.06	-	-	-	-	-	-	-	-	59.33	8.35	2.00	59.00	99.90	89468.29	84837.56
	2000	63.83	56.76	7.06	-	-	-	-	-	-	-	-	59.41	8.65	1.99	60.90	99.90	97813.67	85082.40
	2001	63.69	56.70	6.99	-	-	-	-	-	-	-	-	59.49	9.08	1.99	62.80	99.90	101904.13	85241.96
	2002	63.59	56.64	6.95	-	-	-	-	-	-	-	-	59.57	9.54	1.99	64.70	99.90	96022.37	85436.34
	2003	69.97	62.03	7.93	-	-	-	-	-	-	-	-	59.65	10.02	2.00	66.60	99.80	98123.03	85651.49
	2004	69.94	62.05	7.89	-	-	-	-	-	-	-	-	59.73	10.48	2.00	68.50	99.80	101210.57	85870.69
	2005	69.96	62.07	7.88	-	-	-	-	-	-	-	-	59.81	10.91	2.00	70.40	99.80	106557.19	86128.66
	2006	69.99	62.12	7.87	-	-	-	-	-	-	-	-	59.88	11.37	2.00	72.30	99.80	106807.17	86327.07
	2007	70.00	62.13	7.87	-	-	-	-	-	-	-	-	59.96	11.78	2.00	74.20	99.80	92054.15	86522.67
	2008	70.13	62.25	7.88	-	-	-	-	-	-	-	-	60.04	12.14	1.99	76.10	99.70	99381.17	86707.47
	2009	70.18	62.31	7.87	-	-	-	-	-	-	-	-	60.12	12.47	1.99	78.00	99.70	99920.38	86876.25
	2010	70.26	62.39	7.87	-	-	-	-	-	-	-	-	60.21	12.76	1.98	79.90	99.70	104116.60	87042.91
	2011	70.34	62.48	7.86	-	-	-	-	-	-	-	-	60.32	13.15	1.97	81.80	99.70	107165.32	87153.24
	2012	70.43	62.58	7.85	-	-	-	-	-	-	-	-	60.44	13.54	1.96	81.80	99.70	109894.96	87291.53
	2013	70.54	62.69	7.85	-	-	-	-	-	-	-	-	60.57	13.86	1.95	81.90	99.70	-	87413.71
	2014	70.65	62.80	7.85	-	-	-	-	-	-	-	-	60.72	14.03	1.93	81.90	99.70	-	87549.32
	2015	70.76	62.91	7.85	-	-	-	-	-	-	-	-	60.88	14.01	1.92	81.90	99.70	-	87676.10
	2016	70.85	63.01	7.84	-	-	-	-	-	-	-	-	61.05	13.88	-	-	-	-	87806.79

	2017				-	-	-	-	-	-	-	-	-	-	-	-	-	-	-
Denmark	1995	75.58	65.81	9.77	49122.88	2835.43	8.13	93.26	16.30	82.52	6.71	1.42	84.98	22.61	1.80	99.60	100.00	79490.35	92416.50
	1996	75.90	66.09	9.81	50262.23	2884.22	8.21	92.31	16.23	82.42	6.77	1.44	85.01	22.57	1.75	99.60	100.00	92887.16	92381.61
	1997	76.22	66.38	9.84	51685.98	2633.58	8.15	92.13	16.33	82.28	6.71	1.44	85.03	22.51	1.75	99.60	100.00	83193.33	92360.46
	1998	76.48	66.61	9.86	52640.94	2675.35	8.16	91.26	16.35	82.08	6.69	1.46	85.06	22.44	1.72	99.60	100.00	76245.00	92346.12
	1999	76.60	66.73	9.86	54013.78	2931.36	8.95	90.97	14.60	83.95	7.52	1.44	85.09	22.36	1.73	99.60	100.00	72949.81	92344.20
	2000	76.95	67.02	9.93	55850.64	2612.69	8.70	90.91	14.67	83.86	7.30	1.40	85.10	22.28	1.77	99.60	100.00	68553.55	92347.49
	2001	77.15	67.18	9.97	56108.96	2730.15	9.10	91.09	14.41	84.18	7.66	1.44	85.15	22.36	1.74	99.60	100.00	70080.08	92353.96
	2002	77.31	67.29	10.02	56190.81	3023.12	9.33	90.95	14.11	84.49	7.89	1.45	85.25	22.43	1.72	99.60	100.00	69387.75	92384.64
	2003	77.55	67.46	10.09	56256.77	3756.11	9.51	90.47	13.98	84.55	8.04	1.47	85.36	22.53	1.76	99.60	100.00	75002.53	92415.51
	2004	77.81	67.65	10.16	57608.73	4387.09	9.67	90.40	14.22	84.27	8.15	1.52	85.57	22.68	1.78	99.60	100.00	68854.76	92445.19
	2005	78.11	67.85	10.26	58792.67	4652.52	9.77	90.49	14.05	84.48	8.25	1.52	85.86	22.92	1.80	99.60	100.00	64816.69	92497.66
	2006	78.36	68.04	10.32	60892.77	5017.51	9.92	90.06	13.84	84.64	8.40	1.52	86.10	23.32	1.85	99.60	100.00	72755.34	92508.13
	2007	78.64	68.24	10.40	61174.55	5709.97	9.99	89.38	84.40	84.40	8.43	1.56	86.29	23.78	1.84	99.60	100.00	67993.20	92518.74
	2008	78.94	68.46	10.48	60504.78	6395.90	10.18	88.35	13.55	84.66	8.62	1.56	86.49	24.30	1.89	99.60	100.00	65167.03	92532.79
	2009	79.20	68.65	10.55	57229.05	6464.58	11.47	88.05	13.17	85.04	9.76	1.72	86.65	24.87	1.84	99.60	100.00	63012.02	92557.45
	2010	79.51	68.86	10.65	58041.41	6266.81	11.08	88.72	13.19	85.13	9.43	1.65	86.80	25.49	1.87	99.60	100.00	63679.09	92609.46
	2011	79.86	69.14	10.72	58575.62	6522.13	10.87	87.23	12.82	85.30	9.27	1.60	86.96	26.14	1.75	99.60	100.00	58416.04	92668.00
	2012	80.16	69.37	10.79	58487.79	6203.77	10.98	87.35	12.44	85.76	9.42	1.56	87.14	26.93	1.73	99.60	100.00	53703.22	92748.00
	2013	80.41	69.56	10.85	58788.08	6637.66	11.25	87.67	12.89	85.30	9.59	1.65	87.32	27.87	1.67	99.60	100.00	-	92815.82
	2014	80.68	69.76	10.91	59437.93	6463.24	10.80	87.69	13.36	84.76	9.16	1.65	87.50	28.84	1.69	99.60	100.00	-	92883.46
	2015	80.75	69.83	10.93	59967.74	-	-	-	-	-	-	-	87.68	29.71	1.69	99.60	100.00	-	92941.51
	2016	80.82	69.88	10.95	60637.34	-	-	-	-	-	-	-	87.85	30.37	-	-	-	-	92992.69
	2017	80.76	69.63	11.13	-	-	-	-	-	-	-	-	-	-	-	-	-	-	-
Djibouti	1995	61.28	53.88	7.41	1249.15	30.46	3.95	98.66	39.12	60.34	2.38	1.57	76.30	5.19	5.35	65.10	78.90	1914.42	88254.10
	1996	61.31	53.91	7.40	1173.01	29.29	3.97	98.69	41.09	58.37	2.32	1.65	76.35	5.23	5.15	64.00	79.60	1915.84	88271.73
	1997	61.17	53.79	7.38	1133.83	32.94	4.60	98.87	37.96	61.61	2.83	1.77	76.39	5.27	4.96	62.90	80.20	2018.25	88294.15
	1998	61.29	53.89	7.40	1102.18	38.88	5.45	98.77	33.40	66.18	3.61	1.84	76.44	5.31	4.78	61.70	80.90	1994.59	88330.23
	1999	61.25	53.84	7.41	1094.85	46.91	6.22	98.82	28.78	70.87	4.41	1.81	76.49	5.35	4.62	60.60	81.60	1971.94	88383.62

	2000	61.30	53.84	7.45	1072.62	44.27	5.75	98.53	31.74	67.79	3.90	1.85	76.53	5.37	4.48	59.50	82.30	1969.17	88454.30
	2001	61.46	54.00	7.45	1071.98	42.26	5.37	98.59	34.61	64.89	3.49	1.89	76.58	5.43	4.36	58.40	83.00	2273.90	88478.81
	2002	61.51	54.07	7.44	1080.14	43.57	5.45	98.63	34.98	64.54	3.52	1.93	76.62	5.48	4.24	57.30	83.70	2019.01	88514.94
	2003	61.61	54.17	7.45	1096.49	51.60	6.22	98.70	32.58	66.99	4.17	2.05	76.67	5.52	4.12	56.10	84.40	2094.82	88550.92
	2004	61.62	54.18	7.44	1120.52	58.70	6.76	98.81	31.60	68.02	4.60	2.16	76.72	5.56	3.99	55.00	85.10	2115.90	88587.71
	2005	61.97	54.46	7.51	1137.58	65.68	7.21	98.85	31.17	68.46	4.94	2.28	76.76	5.61	3.87	53.90	85.70	2160.04	88653.87
	2006	61.85	54.39	7.46	1172.79	68.70	7.05	98.84	32.92	66.69	4.70	2.35	76.81	5.67	3.74	52.80	86.40	2149.83	88681.62
	2007	62.49	54.94	7.55	1212.51	81.90	7.72	98.84	69.13	69.13	5.34	2.38	76.85	5.73	3.62	51.70	87.10	2432.43	88709.45
	2008	62.84	55.25	7.59	1261.74	97.69	8.05	98.77	28.27	71.38	5.75	2.30	76.90	5.80	3.50	50.60	87.80	2518.57	88753.33
	2009	63.27	55.62	7.65	1303.22	107.10	8.37	98.82	28.50	71.16	5.96	2.41	76.94	5.87	3.40	49.50	88.50	2523.22	88793.48
	2010	63.63	55.92	7.71	1325.99	120.11	8.84	99.22	41.43	58.25	5.15	3.69	77.00	5.95	3.30	48.40	89.20	2629.83	88846.30
	2011	64.09	56.33	7.76	1361.57	128.23	8.71	99.22	42.05	57.62	5.02	3.69	77.05	6.05	3.21	47.30	89.90	2701.62	88801.23
	2012	64.56	56.74	7.81	1402.80	141.97	8.95	99.22	40.90	58.78	5.26	3.69	77.12	6.16	3.13	47.30	89.90	2766.30	88773.05
	2013	65.04	57.17	7.87	1447.47	153.96	9.14	99.22	41.44	58.23	5.32	3.82	77.19	6.27	3.06	47.30	89.90	-	88746.80
	2014	65.56	57.62	7.94	1508.29	190.76	10.57	99.22	35.84	63.88	6.75	3.82	77.26	6.35	2.98	47.40	90.00	-	88740.28
	2015	66.01	58.00	8.01	1579.92	-	-	-	-	-	-	-	77.34	6.42	2.91	47.40	90.00	-	88736.32
	2016	66.68	58.58	8.10	-	-	-	-	-	-	-	-	77.43	6.48	-	-	-	-	88743.97
	2017	67.35	59.26	8.09	-	-	-	-	-	-	-	-	-	-	-	-	-	-	-
Dominican Republic	1995	71.97	63.17	8.80	3032.79	102.46	5.21	69.94	54.43	22.17	1.16	4.06	57.60	7.73	3.13	75.20	87.00	22043.96	90083.57
	1996	72.06	63.24	8.82	3193.76	106.02	5.01	75.69	57.00	24.69	1.24	3.78	58.44	7.91	3.07	75.60	87.00	23333.57	90067.71
	1997	72.39	63.39	9.00	3392.62	124.17	5.27	68.76	49.34	28.24	1.49	3.78	59.27	8.08	3.01	76.10	87.00	25012.87	90083.84
	1998	72.51	63.19	9.31	3572.31	133.71	5.44	68.14	45.61	33.07	1.80	3.64	60.10	8.25	2.97	76.60	87.00	26833.05	90115.62
	1999	72.86	63.76	9.09	3752.17	144.07	5.66	68.40	44.22	35.34	2.00	3.66	60.93	8.41	2.93	77.00	86.90	27056.23	90147.01
	2000	73.05	64.12	8.93	3902.60	163.05	5.90	68.55	43.09	37.15	2.19	3.71	61.75	8.58	2.89	77.50	86.80	28492.20	90180.31
	2001	73.23	64.24	8.99	3911.76	157.67	5.59	68.20	44.72	34.43	1.93	3.67	62.56	8.74	2.86	77.90	86.80	28416.24	90228.97
	2002	73.33	64.26	9.07	4074.85	163.28	5.79	66.60	43.49	34.70	2.01	3.78	63.36	8.90	2.84	78.40	86.70	29253.32	90290.54
	2003	73.37	64.26	9.11	4003.13	123.70	5.53	67.98	45.61	32.90	1.82	3.71	64.60	9.05	2.81	78.90	86.60	28367.13	90351.32
	2004	73.12	64.44	8.68	3995.40	101.54	4.28	77.19	52.15	32.44	1.39	2.89	66.00	9.19	2.78	79.40	86.60	28494.00	90421.74
	2005	73.32	64.50	8.82	4301.88	154.23	4.26	77.87	50.72	34.87	1.49	2.78	67.36	9.30	2.75	79.90	86.50	29247.79	90491.17

	2006	73.49	64.52	8.98	4692.99	165.87	4.34	77.41	45.66	41.01	1.78	2.56	68.71	9.39	2.72	80.40	86.40	29635.72	90526.91
	2007	73.77	64.49	9.28	5030.36	199.65	4.34	75.84	45.87	45.87	1.99	2.35	70.02	9.47	2.69	80.80	86.30	30393.15	90571.91
	2008	74.08	64.53	9.55	5120.61	206.95	4.20	75.13	38.41	48.88	2.05	2.15	71.30	9.54	2.66	81.30	86.20	30646.08	90615.86
	2009	74.31	64.49	9.82	5099.60	216.25	4.41	71.02	34.86	50.91	2.25	2.17	72.54	9.62	2.63	81.70	86.00	30499.85	90658.76
	2010	74.47	64.13	10.34	5451.07	217.71	4.06	75.09	33.24	55.73	2.26	1.80	73.75	9.72	2.60	82.10	85.90	31668.71	90718.28
	2011	74.60	64.10	10.50	5547.79	244.15	4.22	74.80	28.63	61.73	2.60	1.61	74.93	9.84	2.57	82.50	85.70	32578.16	90753.97
	2012	74.82	64.03	10.79	5630.99	253.80	4.26	71.19	26.08	63.36	2.70	1.56	76.04	9.97	2.54	82.90	85.40	33395.08	90781.65
	2013	75.01	63.86	11.14	5825.53	245.18	4.12	68.90	25.02	63.69	2.62	1.50	77.08	10.12	2.51	83.30	85.20	-	90822.43
	2014	75.22	65.99	9.23	6193.76	268.99	4.38	63.87	21.14	66.90	2.93	1.45	78.06	10.30	2.48	83.60	85.00	-	90862.87
	2015	75.43	62.95	12.48	6552.69	-	-	-	-	-	-	-	78.98	10.50	2.45	84.00	84.70	-	90944.07
	2016	75.64	64.07	11.57	6909.13	-	-	-	-	-	-	-	79.84	10.72	-	-	-	-	91002.60
	2017	73.09	64.23	8.86	-	-	-	-	-	-	-	-	-	-	-	-	-	-	-
Timor-Leste	1995	60.82	52.96	7.86	-	-	-	-	-	-	-	-	22.53	3.58	6.38	37.10	52.80	781.22	88098.21
	1996	62.39	54.27	8.11	-	-	-	-	-	-	-	-	22.87	3.84	6.63	37.10	52.90	637.44	87968.11
	1997	61.48	53.52	7.96	-	-	-	-	-	-	-	-	23.22	4.08	6.83	37.20	52.90	617.30	87889.46
	1998	61.73	53.74	7.99	-	-	-	-	-	-	-	-	23.56	4.30	6.99	37.30	53.00	643.15	87826.78
	1999	55.57	48.62	6.95	385.10	-	-	-	-	-	-	-	23.91	4.51	7.08	37.30	53.10	560.33	87828.35
	2000	63.64	55.34	8.30	602.95	16.99	3.26	48.89	30.56	37.48	1.22	2.04	24.26	4.72	7.11	37.40	54.30	615.33	87865.40
	2001	64.27	55.91	8.37	684.86	21.09	3.75	49.59	33.41	32.64	1.22	2.53	24.62	4.84	7.09	37.40	55.60	790.91	87991.19
	2002	64.77	56.37	8.41	617.55	20.86	3.89	47.79	32.15	32.73	1.27	2.62	24.98	4.97	7.04	37.50	56.90	804.05	88070.42
	2003	65.20	56.75	8.44	580.59	13.38	2.48	100.00	45.82	54.18	1.35	1.14	25.34	5.10	6.97	37.60	58.20	860.41	88121.70
	2004	66.14	57.53	8.62	565.35	14.82	1.31	100.00	45.55	54.45	0.71	0.60	25.71	5.21	6.88	37.70	59.60	878.93	88090.14
	2005	67.16	58.38	8.78	584.53	19.20	1.05	100.00	42.63	57.37	0.60	0.45	26.31	5.29	6.79	37.80	60.90	885.58	87971.26
	2006	68.30	59.33	8.98	538.69	18.30	0.65	100.00	32.52	67.48	0.44	0.21	26.93	5.41	6.69	38.00	62.40	898.10	87821.15
	2007	69.20	60.02	9.19	591.12	10.69	0.37	99.34	48.36	48.36	0.18	0.19	27.56	5.53	6.59	38.30	63.80	901.88	87629.21
	2008	70.03	60.70	9.33	666.68	31.83	0.74	99.35	15.85	84.05	0.62	0.12	28.20	5.67	6.48	38.50	65.20	912.73	87453.76
	2009	70.60	61.17	9.43	743.76	32.24	1.02	99.36	13.99	85.92	0.88	0.14	28.85	5.82	6.36	38.80	66.70	925.42	87295.68
	2010	71.09	61.66	9.43	806.06	36.78	0.92	99.38	11.32	88.61	0.82	0.11	29.51	6.00	6.23	39.10	68.20	940.46	87169.84
	2011	71.59	62.05	9.54	853.15	40.25	0.76	99.48	10.61	89.34	0.68	0.08	30.17	6.15	6.11	39.50	69.70	949.91	87020.72

	2012	72.04	62.47	9.56	876.45	51.02	1.01	99.42	9.09	90.86	0.92	0.09	30.83	6.29	5.98	39.80	71.20	959.35	86907.53
	2013	72.32	62.76	9.56	878.56	56.60	1.29	99.58	8.89	91.08	1.17	0.11	31.49	6.40	5.86	40.10	71.40	-	86858.46
	2014	72.45	62.91	9.53	893.87	57.08	1.48	99.62	9.57	90.40	1.33	0.14	32.13	6.50	5.74	40.40	71.70	-	86859.01
	2015	72.54	63.04	9.50	909.25	-	-	-	-	-	-	-	32.77	6.58	5.62	40.60	71.90	-	86889.48
	2016	72.67	63.29	9.38	939.80	-	-	-	-	-	-	-	33.40	6.66	-	-	-	-	86888.97
	2017	70.81	61.22	9.58	-	-	-	-	-	-	-	-	-	-	-	-	-	-	-
Ecuador	1995	72.76	63.84	8.91	3847.51	72.55	3.40	72.92	32.55	55.36	1.88	1.52	57.77	7.69	3.36	63.50	76.80	38984.73	90697.71
	1996	73.08	64.12	8.96	3832.76	80.73	3.74	72.58	29.82	58.92	2.20	1.54	58.28	7.81	3.29	64.80	77.30	41250.82	90717.11
	1997	73.41	64.42	9.00	3917.66	87.74	3.72	74.29	34.57	53.47	1.99	1.73	58.79	7.93	3.23	66.00	77.90	41388.37	90749.31
	1998	73.64	64.61	9.03	3966.17	82.28	3.58	78.93	48.04	39.14	1.40	2.18	59.29	8.05	3.16	67.20	78.50	40663.02	90807.01
	1999	73.98	64.89	9.09	3706.65	60.32	3.81	84.20	53.80	36.10	1.38	2.43	59.80	8.17	3.09	68.50	79.10	37373.91	90878.41
	2000	74.21	65.10	9.11	3678.90	49.01	3.38	85.29	62.67	26.52	0.90	2.48	60.30	8.29	3.03	69.70	79.70	39805.12	90994.11
	2001	74.52	65.34	9.18	3759.89	73.45	3.86	87.04	59.32	31.84	1.23	2.63	60.80	8.42	2.97	70.80	80.30	41166.86	90923.00
	2002	74.77	65.55	9.22	3848.27	100.80	4.62	87.94	60.72	30.96	1.43	3.19	61.12	8.55	2.91	72.00	80.80	42616.27	90883.70
	2003	75.02	65.77	9.25	3888.34	157.75	6.46	72.19	56.27	22.06	1.43	5.04	61.32	8.68	2.87	73.10	81.30	44122.40	90839.58
	2004	75.29	66.00	9.30	4139.08	168.40	6.22	70.57	54.34	22.99	1.43	4.79	61.51	8.82	2.83	74.20	81.80	45931.82	90804.99
	2005	75.55	66.24	9.31	4286.52	177.48	5.87	71.09	55.24	22.30	1.31	4.56	61.71	8.97	2.79	75.30	82.30	47049.33	90788.76
	2006	75.78	66.45	9.32	4400.86	196.26	5.86	67.89	51.39	24.31	1.42	4.43	61.91	9.09	2.76	76.40	82.80	48495.59	90813.75
	2007	75.99	66.60	9.39	4421.90	213.18	5.94	66.20	25.37	25.37	1.51	4.43	62.10	9.23	2.74	77.40	83.30	49801.89	90838.09
	2008	76.23	66.81	9.42	4624.20	241.97	5.66	67.58	48.11	28.80	1.63	4.03	62.30	9.37	2.71	78.50	83.90	47603.31	90876.53
	2009	76.46	67.01	9.45	4573.25	237.25	5.58	81.33	49.43	39.22	2.19	3.39	62.50	9.52	2.68	79.60	84.40	49448.33	90908.25
	2010	76.68	67.24	9.44	4657.30	274.88	5.90	81.37	47.47	41.67	2.46	3.44	62.69	9.66	2.66	80.70	84.90	50320.70	90948.36
	2011	76.95	67.42	9.53	4943.42	308.97	5.92	81.90	45.94	43.91	2.60	3.32	62.89	9.78	2.63	81.70	85.40	51593.66	90968.21
	2012	77.21	67.64	9.58	5140.26	368.20	6.48	93.16	50.64	45.64	2.96	3.52	63.09	9.89	2.60	82.80	85.90	52746.57	90989.05
	2013	77.47	67.85	9.62	5311.21	439.56	7.29	94.20	44.32	52.95	3.86	3.43	63.30	10.02	2.57	83.80	86.40	-	91022.59
	2014	77.73	68.06	9.67	5428.71	579.19	9.16	95.35	48.43	49.21	4.51	4.65	63.52	10.20	2.54	84.70	86.90	-	91052.16
	2015	77.91	68.21	9.70	5352.88	-	-	-	-	-	-	-	63.74	10.43	2.51	84.70	86.90	-	91082.15
	2016	77.83	68.20	9.63	5191.10	-	-	-	-	-	-	-	63.98	10.70	-	-	-	-	91121.49
	2017	76.73	67.21	9.52	-	-	-	-	-	-	-	-	-	-	-	-	-	-	-

El Salvador	1995	70.28	62.04	8.24	2724.76	108.43	6.38	98.77	60.73	38.52	2.46	3.92	53.97	8.37	3.58	56.90	76.20	9244.05	87793.50
	1996	70.64	62.35	8.29	2741.53	138.52	7.58	97.46	57.51	40.99	3.11	4.47	55.25	8.57	3.48	58.10	77.40	9079.12	87785.08
	1997	71.11	62.74	8.37	2830.08	160.55	8.21	97.32	59.32	39.05	3.20	5.00	56.53	8.78	3.38	59.30	78.60	10076.61	87785.98
	1998	71.35	62.93	8.42	2910.29	176.39	8.43	96.67	54.41	43.72	3.68	4.74	57.80	9.01	3.26	60.50	79.80	10166.68	87790.70
	1999	71.92	63.41	8.51	2986.64	176.19	8.16	94.60	52.29	44.73	3.65	4.51	58.36	9.25	3.14	61.50	80.70	10405.90	87810.55
	2000	72.25	63.71	8.53	3028.87	184.64	8.17	94.62	50.66	46.46	3.80	4.37	58.91	9.49	3.02	62.40	81.60	10344.83	87856.54
	2001	71.98	63.47	8.51	3060.63	189.50	8.02	93.11	49.53	46.81	3.75	4.27	59.46	9.77	2.90	63.30	82.50	11361.00	87927.85
	2002	72.73	64.10	8.63	3114.17	192.39	7.90	93.38	48.39	48.18	3.81	4.09	60.01	10.04	2.79	64.20	83.40	11242.81	88020.63
	2003	72.86	64.21	8.65	3169.13	193.95	7.61	93.30	47.30	49.30	3.75	3.86	60.56	10.30	2.69	65.10	84.30	11251.28	88121.23
	2004	73.03	64.34	8.69	3212.05	194.57	7.30	92.55	46.44	49.82	3.64	3.66	61.11	10.54	2.60	65.90	85.20	11578.90	88235.23
	2005	73.20	64.51	8.69	3310.94	206.66	7.19	91.68	43.07	53.02	3.81	3.38	61.65	10.77	2.53	66.80	86.00	11528.25	88350.82
	2006	73.48	64.76	8.72	3424.83	207.54	6.68	88.93	33.61	62.21	4.15	2.52	62.19	11.00	2.47	67.70	86.90	11690.55	88439.32
	2007	73.67	64.87	8.80	3540.55	212.09	6.32	89.00	59.30	59.30	3.74	2.57	62.72	11.20	2.41	68.50	87.70	12085.96	88529.57
	2008	73.92	65.07	8.85	3569.92	221.77	6.21	88.77	35.81	59.66	3.71	2.51	63.25	11.39	2.36	69.40	88.50	11979.67	88643.82
	2009	73.98	65.11	8.88	3442.87	234.40	6.83	87.94	34.73	60.51	4.13	2.70	63.77	11.57	2.31	70.20	89.30	11911.56	88756.30
	2010	74.27	65.41	8.87	3474.39	245.19	6.91	88.58	33.61	62.06	4.29	2.62	64.29	11.72	2.27	71.00	90.10	11978.85	88876.78
	2011	74.53	65.56	8.97	3535.39	260.16	6.81	88.04	32.07	63.57	4.33	2.48	64.79	11.86	2.22	71.80	90.90	12293.61	88969.09
	2012	74.70	65.73	8.97	3585.28	262.93	6.70	87.08	32.39	62.80	4.21	2.49	65.29	11.99	2.19	72.60	91.60	12577.79	89058.54
	2013	74.80	65.86	8.95	3634.24	276.70	6.95	85.38	28.44	66.69	4.63	2.31	65.78	12.11	2.16	73.40	92.40	-	89152.31
	2014	75.02	66.04	8.98	3668.20	279.65	6.77	84.81	28.85	66.00	4.47	2.30	66.26	12.26	2.13	74.20	93.10	-	89236.93
	2015	75.23	66.21	9.02	3733.92	-	-	-	-	-	-	-	66.73	12.44	2.10	75.00	93.80	-	89310.65
	2016	75.47	66.48	8.99	3802.86	-	-	-	-	-	-	-	67.19	12.63	-	-	-	-	89405.98
	2017	74.01	65.00	9.01	-	-	-	-	-	-	-	-	-	-	-	-	-	-	-
Equatorial Guinea	1995	47.81	41.61	6.21	871.59	17.03	5.38	100.00	27.83	72.17	3.88	1.50	38.83	7.44	5.97	81.10	47.30	1991.99	92037.86
	1996	48.41	42.15	6.26	1398.89	22.26	4.44	100.00	38.46	61.54	2.73	1.71	38.83	7.30	5.96	81.10	47.30	2146.82	91847.44
	1997	50.60	44.00	6.60	3364.35	29.35	3.18	87.25	25.57	70.70	2.25	0.93	38.82	7.15	5.94	81.10	47.30	2937.21	91693.01
	1998	51.93	45.12	6.81	4002.27	31.95	4.28	90.26	25.78	71.44	3.06	1.22	38.82	6.99	5.91	81.10	47.30	3525.00	91541.18
	1999	53.05	46.07	6.98	4831.07	33.37	2.76	85.79	26.00	69.69	1.92	0.84	38.81	6.83	5.88	80.60	47.30	3782.64	91410.76
	2000	54.31	47.12	7.19	5484.30	53.84	2.73	80.13	16.61	79.27	2.17	0.57	38.81	6.68	5.83	80.20	47.30	4048.33	91293.52

	2001	55.77	48.35	7.42	8603.96	47.46	1.78	74.08	18.59	74.90	1.34	0.45	38.80	6.55	5.79	79.70	47.30	5278.00	91196.00
	2002	56.93	49.32	7.61	9867.56	65.28	2.05	87.71	40.17	54.20	1.11	0.94	38.79	6.41	5.73	79.30	47.30	-	91114.70
	2003	57.44	49.75	7.69	10788.03	94.42	2.23	84.22	26.52	68.51	1.53	0.70	38.80	6.27	5.68	78.90	47.30	-	91035.82
	2004	57.98	50.20	7.79	14266.93	174.45	2.40	89.64	39.08	56.41	1.35	1.05	38.82	6.15	5.62	78.40	47.30	5553.98	90959.85
	2005	58.06	50.28	7.78	15941.65	214.10	1.63	87.76	38.57	56.05	0.91	0.72	38.86	6.03	5.56	78.00	47.30	5675.89	90896.16
	2006	58.96	51.08	7.88	16413.54	266.45	2.13	81.65	25.37	68.93	1.47	0.66	38.90	5.90	5.50	77.50	47.40	5693.14	90870.04
	2007	59.36	51.40	7.96	18075.18	344.18	2.25	82.71	72.99	72.99	1.64	0.61	38.96	5.77	5.43	77.10	47.40	5927.14	90837.73
	2008	59.77	51.76	8.01	20333.94	519.90	2.31	79.66	17.79	77.67	1.80	0.52	39.04	5.65	5.36	76.70	47.40	5766.37	90820.78
	2009	60.25	52.16	8.09	19684.70	702.63	5.32	87.78	14.18	83.85	4.46	0.86	39.12	5.53	5.29	76.20	47.50	5840.52	90805.81
	2010	60.93	52.78	8.15	17136.45	609.27	3.84	86.57	17.63	79.63	3.06	0.78	39.22	5.41	5.21	75.80	47.50	6099.11	90783.38
	2011	61.41	53.15	8.26	17461.55	688.82	3.29	84.90	17.94	78.87	2.60	0.70	39.34	5.29	5.13	75.40	47.60	-	90745.28
	2012	62.26	53.86	8.40	18106.34	852.46	4.00	84.44	14.25	83.13	3.33	0.67	39.46	5.16	5.04	74.90	47.70	6374.17	90730.67
	2013	63.66	55.03	8.62	16634.77	729.75	3.73	86.39	17.85	79.33	2.96	0.77	39.60	5.03	4.95	74.50	47.70	-	90711.29
	2014	64.65	55.88	8.78	16028.25	663.05	3.80	87.95	20.15	77.09	2.93	0.87	39.76	4.92	4.86	74.50	47.80	-	90683.09
	2015	65.16	56.32	8.85	14010.94	-	-	-	-	-	-	-	39.92	4.84	4.78	74.50	47.90	-	90663.36
	2016	65.47	56.66	8.81	12278.13	-	-	-	-	-	-	-	40.10	4.79	-	-	-	-	90640.68
	2017	65.52	56.32	9.20	-	-	-	-	-	-	-	-	-	-	-	-	-	-	-
Eritrea	1995	56.47	49.67	6.80	539.93	8.58	4.52	100.00	51.99	48.01	2.17	2.35	16.54	6.31	5.91	9.80	48.40	3751.32	88797.23
	1996	57.10	50.24	6.86	584.95	7.82	3.61	100.00	63.43	36.57	1.32	2.29	16.72	6.38	5.79	10.10	48.90	4186.31	88816.87
	1997	57.76	50.83	6.93	622.24	9.31	4.20	100.00	57.82	42.18	1.77	2.43	16.91	6.43	5.67	10.40	49.40	4543.02	88840.35
	1998	58.30	51.31	6.99	620.92	10.02	4.48	100.00	58.71	41.29	1.85	2.63	17.11	6.45	5.55	10.70	49.90	4670.67	88877.85
	1999	49.38	43.75	5.62	606.33	10.05	5.01	100.00	60.11	39.89	2.00	3.01	17.33	6.45	5.43	11.10	50.50	4939.41	88897.24
	2000	58.08	51.17	6.91	571.60	8.86	4.43	100.00	64.18	35.82	1.59	2.85	17.56	6.45	5.33	11.40	51.00	4981.26	88927.90
	2001	59.43	52.32	7.11	603.11	8.13	3.95	100.00	64.88	35.12	1.39	2.56	17.80	6.40	5.23	11.70	51.50	4725.51	88958.56
	2002	59.73	52.60	7.13	601.03	7.74	4.02	100.00	59.22	40.78	1.64	2.38	18.05	6.34	5.15	12.00	52.10	4456.55	88980.11
	2003	59.97	52.82	7.14	565.73	7.76	3.50	100.00	63.12	36.88	1.29	2.21	18.32	6.28	5.07	12.40	52.60	4481.71	88997.59
	2004	60.19	53.03	7.16	556.04	8.57	3.14	100.00	60.67	39.33	1.24	1.91	18.60	6.21	5.01	12.70	53.10	4532.74	88985.64
	2005	60.38	53.22	7.17	554.49	7.78	2.97	100.00	63.07	36.93	1.10	1.87	18.89	6.16	4.94	13.00	53.70	4476.29	88969.04
	2006	60.71	53.51	7.20	535.93	9.28	3.30	100.00	55.43	44.57	1.47	1.83	19.20	6.25	4.88	13.30	54.20	4483.42	88934.67

	2007	60.98	53.73	7.25	532.23	9.85	3.29	100.00	45.28	45.28	1.49	1.80	19.52	6.33	4.82	13.70	54.80	4868.86	88910.65
	2008	61.18	53.91	7.27	471.17	11.31	3.69	100.00	47.64	52.36	1.93	1.76	19.85	6.40	4.75	14.00	55.30	4954.76	88903.07
	2009	61.41	54.12	7.30	480.61	13.35	3.30	100.00	57.66	42.34	1.40	1.90	20.21	6.46	4.68	14.30	55.90	4751.09	88904.79
	2010	61.60	54.30	7.31	482.15	14.63	3.24	100.00	56.03	43.97	1.42	1.82	20.57	6.51	4.61	14.60	56.40	4844.79	88941.28
	2011	61.94	54.59	7.35	514.18	16.67	3.06	100.00	54.01	45.99	1.41	1.65	20.95	6.61	4.53	14.90	57.00	4912.88	88892.08
	2012	62.27	54.90	7.37	-	18.81	2.98	100.00	53.50	46.50	1.38	1.59	-	-	4.44	15.30	57.50	4977.89	88866.96
	2013	62.57	55.17	7.40	-	20.75	3.01	100.00	54.24	45.76	1.38	1.63	-	-	4.36	15.40	57.60	-	88844.12
	2014	62.95	55.52	7.44	-	25.20	3.34	100.00	54.24	45.76	1.53	1.81	-	-	4.29	15.60	57.70	-	88860.01
	2015	63.30	55.82	7.48	-	-	-	-	-	-	-	-	-	-	4.21	15.70	57.80	-	88874.25
	2016	63.66	56.16	7.50	-	-	-	-	-	-	-	-	-	-	-	-	-	-	88918.86
	2017	62.67	54.36	8.31	-	-	-	-	-	-	-	-	-	-	-	-	-	-	-
Estonia	1995	67.70	59.07	8.63	7313.74	164.80	6.32	100.00	10.23	89.77	5.67	0.65	70.30	20.68	1.38	96.90	98.90	31933.33	90739.19
	1996	69.89	60.86	9.03	7817.60	218.12	6.57	98.85	11.49	88.37	5.81	0.76	70.11	21.11	1.37	96.90	98.90	32604.28	90800.01
	1997	70.13	61.06	9.07	8840.26	222.44	6.19	97.87	10.60	89.17	5.52	0.67	69.92	21.45	1.32	96.90	98.90	32114.21	90874.62
	1998	69.73	60.79	8.94	9293.08	223.10	5.56	96.62	13.19	86.35	4.80	0.76	69.73	21.71	1.28	96.90	98.90	19347.23	90944.08
	1999	70.54	61.43	9.12	9186.39	242.57	5.86	74.41	14.11	77.47	4.54	1.11	69.54	21.98	1.30	96.90	99.00	18098.98	91006.15
	2000	70.84	61.69	9.15	10108.21	214.38	5.28	88.49	19.93	77.16	4.08	1.19	69.37	22.28	1.36	97.00	99.00	17768.81	91091.14
	2001	70.61	61.54	9.08	10816.60	217.71	4.85	88.94	18.99	78.65	3.82	1.04	69.24	22.82	1.32	97.00	99.00	17940.16	91102.89
	2002	71.38	62.17	9.21	11546.77	256.66	4.84	87.72	20.07	77.12	3.74	1.11	69.12	23.37	1.36	97.00	99.10	22955.36	91117.28
	2003	71.94	62.62	9.32	12481.19	352.40	4.92	88.01	20.41	76.75	3.78	1.14	68.99	23.90	1.37	97.00	99.10	19449.74	91120.89
	2004	72.43	63.02	9.41	13346.40	452.33	5.13	88.82	21.32	75.55	3.88	1.23	68.86	24.32	1.47	97.00	99.20	20024.43	91116.04
	2005	72.89	63.44	9.45	14681.23	514.22	5.02	88.74	20.42	76.70	3.85	1.15	68.74	24.61	1.52	97.10	99.20	20053.58	91118.20
	2006	73.20	63.69	9.50	16285.01	624.62	5.01	95.97	25.09	73.25	3.67	1.31	68.61	24.99	1.58	97.10	99.30	19628.39	91098.85
	2007	73.25	63.67	9.59	17627.03	845.47	5.16	94.08	75.58	75.58	3.90	1.20	68.48	25.26	1.69	97.10	99.30	23503.71	91072.41
	2008	74.38	64.51	9.86	16716.50	1075.41	6.06	95.20	19.66	77.81	4.71	1.25	68.35	25.47	1.72	97.10	99.40	21871.91	91051.92
	2009	75.14	65.09	10.05	14282.60	1008.52	6.93	97.38	20.32	75.25	5.22	1.45	68.22	25.71	1.70	97.10	99.40	18815.61	91047.22
	2010	76.23	65.97	10.26	14638.60	902.67	6.25	91.69	18.57	78.85	4.93	1.27	68.09	26.02	1.72	97.20	99.50	22767.13	91076.47
	2011	76.50	66.13	10.37	15798.63	988.10	5.83	91.28	17.56	79.32	4.63	1.12	67.97	26.50	1.61	97.20	99.50	24378.85	91069.34
	2012	76.53	66.17	10.36	16538.21	1077.55	6.36	97.57	19.15	80.38	5.12	1.25	67.84	27.05	1.56	97.20	99.50	23292.78	91091.49

	2013	77.38	66.85	10.53	16918.59	1213.56	6.48	97.84	20.26	79.28	5.14	1.34	67.72	27.65	1.52	97.20	99.60	-	91117.57
	2014	77.29	66.81	10.48	17453.37	1248.28	6.38	97.84	20.72	78.82	5.03	1.35	67.62	28.29	1.54	97.20	99.60	-	91159.64
	2015	77.45	66.95	10.49	17733.96	-	-	-	-	-	-	-	67.54	28.94	1.54	97.20	99.60	-	91193.92
	2016	77.58	67.16	10.41	18085.09	-	-	-	-	-	-	-	67.47	29.62	-	-	-	-	91231.18
	2017	78.09	67.03	11.06	-	-	-	-	-	-	-	-	-	-	-	-	-	-	-
Ethiopia	1995	49.87	44.00	5.87	182.71	4.02	2.96	78.33	46.51	40.62	1.20	1.76	13.83	6.13	7.00	3.00	19.50	69202.10	88793.80
	1996	50.52	44.57	5.95	199.01	4.20	2.92	78.57	48.60	38.15	1.11	1.81	14.01	6.14	6.94	3.00	21.40	71509.73	88778.67
	1997	51.02	45.01	6.00	199.11	4.37	3.20	78.53	46.43	40.87	1.31	1.89	14.19	6.17	6.86	4.60	23.30	73810.38	88783.42
	1998	51.46	45.40	6.05	186.66	4.50	3.73	79.49	44.71	43.76	1.63	2.10	14.37	6.18	6.77	5.90	25.20	190909.59	88786.00
	1999	51.07	45.09	5.98	190.69	4.46	3.96	79.60	42.52	46.58	1.84	2.11	14.55	6.17	6.66	7.30	27.00	217793.99	88785.50
	2000	51.10	45.13	5.97	196.50	5.37	4.36	79.17	35.95	54.60	2.38	1.98	14.74	6.14	6.53	8.60	28.90	183617.67	88802.27
	2001	53.06	46.82	6.25	206.74	5.05	4.26	79.84	38.06	52.33	2.23	2.03	14.93	6.16	6.38	9.90	30.80	144657.84	88781.21
	2002	53.84	47.50	6.34	203.91	5.26	4.73	80.30	37.00	53.92	2.55	2.18	15.12	6.15	6.22	11.20	32.70	186749.54	88764.84
	2003	54.51	48.09	6.42	193.87	5.51	4.64	80.52	36.11	55.15	2.56	2.08	15.31	6.14	6.04	12.60	34.50	171731.66	88752.90
	2004	55.28	48.76	6.52	214.04	5.75	4.23	80.44	35.35	56.05	2.37	1.86	15.50	6.12	5.87	13.90	36.40	175248.63	88753.58
	2005	56.06	49.46	6.60	232.78	6.79	4.20	79.31	30.61	61.41	2.58	1.62	15.70	6.11	5.69	15.20	38.30	200010.18	88759.78
	2006	57.02	50.33	6.69	251.06	8.65	4.47	77.31	33.53	56.63	2.53	1.94	15.90	6.16	5.52	16.50	40.10	175184.51	88728.65
	2007	58.17	51.31	6.86	272.39	11.21	4.69	77.91	54.83	54.83	2.57	2.12	16.12	6.21	5.36	17.80	42.00	189006.75	88701.70
	2008	59.36	52.34	7.02	293.85	13.43	4.28	79.96	38.47	51.89	2.22	2.06	16.51	6.25	5.20	19.10	43.90	175266.23	88692.94
	2009	60.38	53.22	7.16	311.37	14.83	4.41	85.14	38.38	54.92	2.42	1.99	16.91	6.28	5.06	20.40	45.90	182032.58	88698.18
	2010	61.28	54.03	7.25	341.31	20.98	6.86	73.53	33.70	54.17	3.72	3.14	17.32	6.32	4.92	21.70	47.80	182771.20	88709.06
	2011	62.22	54.83	7.39	369.58	22.21	6.55	73.95	31.76	57.05	3.74	2.81	17.74	6.33	4.80	23.00	49.70	184045.88	88604.18
	2012	63.06	55.58	7.48	391.13	26.42	5.77	77.85	33.18	57.38	3.31	2.46	18.16	6.34	4.68	24.30	51.60	185292.17	88501.12
	2013	63.85	56.28	7.57	421.38	25.04	5.19	77.39	33.37	56.89	2.95	2.24	18.59	6.35	4.56	25.50	53.50	-	88396.95
	2014	64.62	56.97	7.66	452.78	26.65	4.88	78.14	32.26	58.71	2.87	2.02	19.03	6.35	4.44	26.80	55.40	-	88313.87
	2015	65.14	57.42	7.72	487.29	-	-	-	-	-	-	-	19.47	6.34	4.32	28.00	57.30	-	88234.10
	2016	65.63	57.91	7.72	511.19	-	-	-	-	-	-	-	19.92	6.33	-	-	-	-	88137.69
	2017	68.39	59.53	8.86	-	-	-	-	-	-	-	-	-	-	-	-	-	-	-
Federated	1995	64.15	56.36	7.79	2721.86	189.00	9.16	100.00	5.03	94.97	8.70	0.46	25.07	6.67	4.66	25.00	90.70	57.83	85288.32

States of	1996	64.35	56.54	7.81	2617.99	174.34	8.62	100.00	5.68	94.32	8.14	0.49	24.47	6.72	4.61	26.70	90.60	58.09	85428.24
	1997	64.52	56.68	7.84	2455.61	148.43	7.78	100.00	6.47	93.53	7.28	0.50	23.89	6.80	4.54	28.40	90.50	-	85553.34
	1998	64.74	56.85	7.89	2534.36	141.22	6.95	100.00	6.99	93.01	6.46	0.49	23.31	6.85	4.47	30.10	90.40	-	85711.61
	1999	64.95	57.02	7.93	2579.48	155.35	7.58	100.00	6.78	93.22	7.06	0.51	22.75	6.81	4.39	31.90	90.30	-	85882.72
	2000	64.71	56.79	7.92	2706.83	170.94	7.88	100.00	6.70	93.30	7.35	0.53	22.33	6.65	4.30	33.60	90.10	-	86068.37
	2001	65.39	57.37	8.02	2760.98	192.22	8.58	100.00	6.72	93.28	8.01	0.58	22.33	6.79	4.20	35.60	90.00	-	86229.28
	2002	64.44	56.57	7.86	2782.27	180.66	8.02	100.00	7.55	92.45	7.41	0.61	22.33	6.90	4.11	37.50	90.00	-	86406.98
	2003	65.62	57.56	8.06	2840.87	222.27	9.69	100.00	6.82	93.18	9.03	0.66	22.33	6.95	4.01	39.50	89.90	-	86599.77
	2004	65.68	57.61	8.08	2758.10	238.52	10.64	100.00	6.92	93.08	9.90	0.74	22.33	6.99	3.91	41.40	89.80	-	86778.08
	2005	65.58	57.53	8.05	2824.93	285.13	12.11	96.50	6.38	93.39	11.31	0.80	22.33	7.00	3.82	43.40	89.70	-	86980.58
	2006	65.58	57.59	8.00	2834.91	285.47	11.92	96.89	7.23	92.54	11.03	0.89	22.32	6.89	3.74	45.30	89.60	-	87144.77
	2007	65.57	57.61	7.97	2795.30	292.11	11.99	94.06	92.87	92.87	11.14	0.85	22.32	6.76	3.66	47.30	89.50	-	87297.93
	2008	65.59	57.64	7.94	2749.01	323.33	12.94	97.54	9.40	90.37	11.70	1.25	22.31	6.65	3.59	49.20	89.40	-	87431.98
	2009	65.50	57.59	7.91	2795.23	358.12	13.44	97.54	9.20	90.57	12.17	1.27	22.31	6.55	3.52	51.20	89.30	-	87554.32
	2010	65.32	57.47	7.85	2861.77	392.41	13.83	97.54	8.60	91.18	12.61	1.22	22.30	6.49	3.46	53.10	89.20	-	87667.79
	2011	65.38	57.51	7.88	2961.79	410.81	13.76	97.54	8.64	91.14	12.54	1.22	22.30	6.53	3.40	55.10	89.10	-	87840.59
	2012	65.50	57.59	7.91	2901.88	402.08	12.77	97.54	9.01	90.76	11.59	1.18	22.32	6.59	3.35	57.00	89.00	-	87997.67
	2013	65.56	57.62	7.94	2784.56	406.49	13.38	97.54	9.43	90.33	12.09	1.29	22.34	6.70	3.29	57.00	89.00	-	88161.19
	2014	65.40	57.47	7.92	2716.32	414.99	13.71	97.54	9.06	90.71	12.43	1.27	22.38	6.86	3.24	57.10	89.00	-	88304.97
	2015	65.26	57.35	7.92	2838.87	-	-	-	-	-	-	-	22.42	7.07	3.19	57.10	89.00	-	88471.03
	2016	65.50	57.51	7.99	2823.43	-	-	-	-	-	-	-	22.48	7.39	-	-	-	-	88622.70
	2017	67.21	58.23	8.98	-	-	-	-	-	-	-	-	-	-	-	-	-	-	-
Fiji	1995	64.49	56.36	8.13	3122.91	79.11	3.11	63.49	13.25	79.13	2.46	0.65	45.50	5.00	3.28	66.30	88.30	2182.20	86676.31
	1996	64.32	56.21	8.11	3235.36	88.82	3.27	63.49	12.20	80.79	2.64	0.63	46.28	5.13	3.24	68.10	88.80	2222.89	86922.39
	1997	64.08	56.00	8.08	3130.72	93.28	3.53	63.49	11.49	81.90	2.89	0.64	46.73	5.25	3.21	69.80	89.30	2217.20	87177.87
	1998	64.09	56.01	8.09	3141.88	65.93	3.18	63.49	11.57	81.78	2.60	0.58	47.12	5.36	3.17	71.40	89.70	2322.71	87426.48
	1999	64.01	55.92	8.09	3392.17	75.73	3.14	63.49	12.91	79.67	2.51	0.64	47.52	5.47	3.13	73.00	90.20	2090.80	87675.41
	2000	64.18	56.02	8.16	3315.07	80.31	3.87	63.49	10.39	83.63	3.24	0.63	47.91	5.58	3.09	74.60	90.70	1574.68	87923.84
	2001	64.37	56.20	8.17	3368.93	66.91	3.28	63.49	12.64	80.09	2.63	0.65	48.30	5.69	3.05	76.20	91.10	1639.36	88115.83

	2002	64.54	56.35	8.19	3470.46	78.66	3.48	63.49	11.43	81.99	2.85	0.63	48.69	5.81	3.00	77.70	91.60	1807.00	88291.78
	2003	64.61	56.41	8.20	3501.14	95.08	3.35	63.49	12.35	80.55	2.70	0.65	49.09	5.95	2.95	79.30	92.00	1931.69	88488.75
	2004	64.70	56.49	8.21	3678.93	120.25	3.61	63.49	12.67	80.04	2.89	0.72	49.48	6.11	2.91	80.80	92.50	1810.48	88686.66
	2005	64.76	56.54	8.21	3689.07	132.00	3.61	63.49	11.82	81.38	2.94	0.67	49.87	6.29	2.86	82.30	92.90	1836.07	88889.36
	2006	64.74	56.55	8.18	3732.00	143.85	3.84	63.17	13.56	78.53	3.01	0.82	50.26	6.51	2.82	83.80	93.40	1945.97	88869.25
	2007	64.74	56.58	8.16	3667.45	152.57	3.74	60.82	74.65	74.65	2.79	0.95	50.66	6.71	2.77	85.30	93.80	2097.70	88864.36
	2008	64.78	56.62	8.15	3667.86	153.71	3.68	63.38	15.48	75.58	2.78	0.90	51.05	6.92	2.74	86.80	94.30	2123.96	88872.96
	2009	64.87	56.71	8.17	3580.40	143.49	4.26	72.47	22.20	69.37	2.95	1.30	51.44	7.11	2.70	88.20	94.70	2336.07	88898.62
	2010	65.17	56.93	8.24	3651.97	154.40	4.23	65.82	19.66	70.13	2.97	1.26	51.83	7.30	2.67	89.60	95.10	2163.92	88919.57
	2011	65.36	57.12	8.24	3719.89	170.51	4.06	64.72	22.00	66.01	2.68	1.38	52.21	7.63	2.64	91.00	95.60	2213.24	88990.47
	2012	65.30	57.10	8.20	3744.28	183.38	4.16	64.55	21.73	66.34	2.76	1.40	52.60	7.95	2.62	91.10	95.60	2258.17	89065.54
	2013	65.36	57.18	8.18	3894.26	186.64	4.26	64.55	20.60	68.08	2.90	1.36	52.98	8.28	2.59	91.10	95.60	-	89140.99
	2014	65.37	57.19	8.17	4084.20	204.01	4.49	67.26	23.00	65.81	2.95	1.53	53.35	8.60	2.57	91.10	95.70	-	89222.23
	2015	65.37	57.20	8.17	4211.06	-	-	-	-	-	-	-	53.73	8.91	2.54	91.10	95.70	-	89314.17
	2016	65.38	57.21	8.17	4195.97	-	-	-	-	-	-	-	54.10	9.23	-	-	-	-	89405.00
	2017	68.07	59.14	8.94	-	-	-	-	-	-	-	-	-	-	-	-	-	-	-
Finland	1995	76.70	66.41	10.29	31997.00	2014.08	7.85	79.99	22.65	71.69	5.63	2.22	80.96	21.41	1.81	97.30	100.00	77426.36	91558.28
	1996	76.86	66.55	10.31	33059.10	2014.75	8.03	79.85	22.66	71.62	5.75	2.28	81.22	21.67	1.76	97.30	100.00	83954.85	91584.25
	1997	77.09	66.73	10.36	35021.59	1848.77	7.71	79.42	22.15	72.11	5.56	2.15	81.47	21.88	1.75	97.40	100.00	82093.03	91624.80
	1998	77.29	66.89	10.40	36824.85	1853.03	7.35	78.53	21.62	72.47	5.33	2.02	81.71	22.06	1.70	97.40	100.00	77932.97	91671.40
	1999	77.49	67.05	10.44	38372.22	1862.53	7.37	78.38	22.37	71.46	5.27	2.10	81.95	22.23	1.73	97.40	100.00	76491.33	91731.82
	2000	77.81	67.28	10.53	40450.37	1700.32	7.22	77.65	22.32	71.26	5.14	2.07	82.18	22.42	1.73	97.40	100.00	75117.48	91792.17
	2001	78.14	67.54	10.60	41399.94	1786.26	7.43	77.73	21.79	71.97	5.34	2.08	82.37	22.73	1.73	97.40	100.00	80288.02	91838.88
	2002	78.39	67.73	10.67	41993.69	2032.23	7.81	77.22	21.27	72.45	5.66	2.15	82.50	23.06	1.72	97.50	100.00	83951.85	91884.05
	2003	78.56	67.84	10.72	42729.02	2571.71	8.15	76.76	20.90	72.78	5.93	2.22	82.64	23.40	1.76	97.50	100.00	95013.03	91922.90
	2004	78.74	67.95	10.79	44277.84	2974.00	8.21	76.78	20.50	73.30	6.02	2.19	82.77	23.74	1.80	97.50	100.00	88571.16	91949.02
	2005	78.98	68.11	10.87	45353.26	3151.84	8.43	76.63	20.07	73.81	6.22	2.21	82.91	24.05	1.80	97.50	100.00	76199.79	91996.09
	2006	79.18	68.30	10.87	47011.66	3316.63	8.38	75.72	18.99	74.92	6.28	2.10	83.04	24.32	1.84	97.50	100.00	88152.98	92012.09
	2007	79.38	68.50	10.89	49239.19	3775.58	8.09	75.31	74.53	74.53	6.03	2.06	83.17	24.55	1.83	97.50	100.00	85742.23	92023.68

	2008	79.62	68.71	10.91	49363.70	4287.02	8.35	75.29	18.93	74.86	6.25	2.10	83.30	24.83	1.85	97.50	100.00	78384.73	92033.84
	2009	79.80	68.89	10.92	45065.75	4136.66	9.20	74.70	18.72	74.94	6.90	2.31	83.43	25.29	1.86	97.60	100.00	74993.89	92038.63
	2010	80.03	69.09	10.93	46202.42	4001.33	9.05	75.74	19.31	74.50	6.74	2.31	83.56	26.01	1.87	97.60	100.00	84371.54	92045.53
	2011	80.32	69.34	10.97	47171.02	4398.94	9.01	75.08	18.81	74.94	6.75	2.26	83.69	26.97	1.83	97.60	100.00	76124.63	92058.87
	2012	80.57	69.56	11.01	46277.56	4254.76	9.30	74.33	18.04	75.72	7.04	2.26	83.82	28.15	1.80	97.60	100.00	69072.94	92088.02
	2013	80.93	69.86	11.07	45715.65	4518.97	9.55	74.13	18.19	75.46	7.20	2.34	83.95	29.48	1.75	97.60	100.00	-	92115.46
	2014	81.27	70.13	11.13	45239.37	4612.29	9.68	73.84	18.23	75.31	7.29	2.39	84.09	30.79	1.71	97.60	100.00	-	92151.43
	2015	81.53	70.36	11.17	45086.74	-	-	-	-	-	-	-	84.22	31.99	1.71	97.60	100.00	-	92180.50
	2016	81.74	70.53	11.21	45825.49	-	-	-	-	-	-	-	84.36	33.07	-	-	-	-	92214.29
	2017	81.42	69.78	11.64	-	-	-	-	-	-	-	-	-	-	-	-	-	-	-
France	1995	78.12	68.28	9.84	34145.70	2744.52	10.11	37.43	7.60	79.69	8.06	2.05	74.91	23.08	1.74	98.70	100.00	543013.14	90411.35
	1996	78.33	68.48	9.86	34497.29	2741.66	10.11	35.83	7.32	79.56	8.04	2.07	75.08	23.43	1.75	98.70	100.00	572529.08	90437.62
	1997	78.65	68.75	9.90	35178.94	2439.59	9.98	35.33	7.19	79.64	7.94	2.03	75.25	23.76	1.77	98.70	100.00	565220.99	90464.51
	1998	78.77	68.86	9.91	36295.93	2486.68	9.86	34.74	7.12	79.52	7.84	2.02	75.42	24.07	1.78	98.70	100.00	577851.54	90486.41
	1999	78.92	69.00	9.92	37339.98	2458.13	9.86	34.86	7.16	79.45	7.83	2.03	75.61	24.33	1.81	98.70	100.00	563699.03	90497.91
	2000	79.12	69.16	9.96	38522.21	2209.24	9.77	34.44	7.10	79.38	7.76	2.02	75.87	24.55	1.89	98.70	100.00	559922.20	90520.78
	2001	79.35	69.37	9.99	38990.31	2241.06	9.89	34.79	7.17	79.38	7.85	2.04	76.13	24.77	1.90	98.70	100.00	565204.33	90519.21
	2002	79.59	69.58	10.01	39140.71	2496.52	10.22	34.28	6.97	79.66	8.14	2.08	76.38	24.95	1.88	98.70	100.00	557850.13	90531.18
	2003	79.77	69.74	10.03	39182.78	3115.24	10.43	32.20	7.10	77.94	8.13	2.30	76.63	25.10	1.89	98.70	100.00	564607.18	90543.81
	2004	80.15	70.05	10.10	39979.12	3592.42	10.53	31.51	6.96	77.90	8.21	2.33	76.88	25.21	1.92	98.70	100.00	560648.84	90550.45
	2005	80.40	70.26	10.14	40316.81	3721.11	10.60	32.15	7.08	77.99	8.27	2.33	77.13	25.27	1.94	98.70	100.00	557130.13	90562.45
	2006	80.67	70.45	10.22	40987.55	3871.53	10.53	33.16	7.42	77.62	8.17	2.36	77.38	25.34	2.00	98.70	100.00	547361.38	90615.05
	2007	80.91	70.61	10.29	41696.69	4371.97	10.45	32.86	77.69	77.69	8.12	2.33	77.62	25.35	1.98	98.70	100.00	540947.14	90684.41
	2008	81.08	70.72	10.36	41545.29	4827.88	10.57	33.55	7.62	77.29	8.17	2.40	77.86	25.38	2.01	98.70	100.00	544424.06	90760.38
	2009	81.29	70.85	10.44	40116.38	4722.01	11.28	33.38	7.50	77.54	8.75	2.53	78.11	25.56	2.00	98.70	100.00	527525.78	90843.52
	2010	81.48	70.97	10.52	40703.34	4583.69	11.20	33.14	7.45	77.51	8.68	2.52	78.35	25.96	2.03	98.70	100.00	532133.04	90952.33
	2011	81.71	71.16	10.55	41349.19	4993.72	11.33	30.83	6.69	77.03	8.73	2.46	78.58	26.55	2.01	98.70	100.00	502656.65	91030.60
	2012	81.84	71.28	10.56	41224.73	4698.92	11.44	29.98	6.51	77.16	8.83	2.48	78.82	27.34	2.01	98.70	100.00	499146.63	91125.87
	2013	82.01	71.42	10.59	41249.45	4955.35	11.56	29.32	6.35	77.08	8.91	2.50	79.06	28.28	1.99	98.70	100.00	-	91224.44

	2014	82.22	71.59	10.63	41431.04	4958.99	11.54	29.08	6.34	78.21	9.02	2.52	79.29	29.25	2.01	98.70	100.00	-	91342.56
	2015	82.27	71.64	10.63	41689.71	-	-	-	-	-	-	-	79.52	30.16	2.01	98.70	100.00	-	91468.33
	2016	82.34	71.71	10.64	42013.29	-	-	-	-	-	-	-	79.75	30.96	-	-	-	-	91598.52
	2017	82.84	71.75	11.09	-	-	-	-	-	-	-	-	-	-	-	-	-	-	-
Gabon	1995	59.59	51.73	7.87	11342.21	158.98	3.48	83.48	53.34	36.10	1.26	2.23	75.36	11.38	5.00	38.30	80.00	13230.37	90064.50
	1996	59.25	51.47	7.79	11459.46	173.48	3.40	83.48	51.87	37.87	1.29	2.11	76.43	11.35	4.90	38.30	80.60	13569.32	90025.54
	1997	59.63	51.77	7.86	11816.54	140.00	3.01	83.48	50.38	39.66	1.19	1.81	77.44	11.28	4.81	38.30	81.20	12977.43	89996.42
	1998	59.61	51.75	7.86	11925.78	162.18	4.24	83.48	49.42	40.80	1.73	2.51	78.38	11.19	4.72	38.40	81.80	38130.03	89971.80
	1999	59.37	51.55	7.82	10593.24	136.52	3.52	83.48	54.69	34.49	1.21	2.31	79.26	11.08	4.63	38.60	82.80	35739.95	89938.86
	2000	59.11	51.32	7.78	10137.55	118.89	2.89	83.48	50.11	39.97	1.15	1.73	80.08	10.94	4.54	38.80	83.80	32923.10	89927.99
	2001	58.82	51.12	7.70	10098.60	128.61	3.23	83.48	50.25	39.81	1.29	1.94	80.85	10.77	4.46	39.00	84.70	13751.40	89898.50
	2002	59.17	51.40	7.77	9823.25	132.58	3.22	83.48	52.17	37.51	1.21	2.01	81.57	10.57	4.39	39.20	85.60	12656.01	89886.43
	2003	59.21	51.45	7.76	9788.88	171.49	3.48	83.48	48.27	42.18	1.47	2.01	82.23	10.35	4.33	39.40	86.40	14728.79	89871.57
	2004	59.24	51.51	7.73	9595.85	187.36	3.26	83.48	48.59	41.80	1.36	1.89	82.85	10.12	4.27	39.60	87.20	15137.54	89868.59
	2005	59.92	52.01	7.91	9579.36	189.15	2.76	83.48	49.70	40.47	1.11	1.64	83.42	9.89	4.23	39.80	87.90	14185.92	89883.76
	2006	60.44	52.46	7.98	9041.67	207.71	2.88	83.48	47.71	42.85	1.24	1.65	83.95	9.63	4.19	40.00	88.60	13966.24	89892.11
	2007	61.14	53.01	8.12	9299.46	246.75	2.86	83.48	44.33	44.33	1.27	1.59	84.44	9.36	4.16	40.30	89.30	13080.54	89902.84
	2008	61.41	53.26	8.14	8715.46	266.86	2.54	83.48	48.05	42.44	1.08	1.46	84.90	9.10	4.14	40.50	89.90	61809.41	89918.88
	2009	61.96	53.72	8.24	8449.94	276.35	3.43	83.48	45.78	45.16	1.55	1.88	85.31	8.84	4.11	40.70	90.50	14325.64	89948.12
	2010	62.80	54.41	8.39	8754.11	319.74	3.41	67.36	18.39	71.67	2.44	0.93	85.70	8.59	4.08	40.90	91.10	34053.55	89981.40
	2011	63.22	54.77	8.45	9060.66	353.00	3.12	70.98	23.17	67.36	2.10	1.02	86.05	8.37	4.05	41.20	91.60	34324.76	89970.43
	2012	63.84	55.30	8.53	9212.29	343.24	3.13	71.45	21.02	70.58	2.21	0.92	86.37	8.16	4.01	41.40	92.20	34570.63	89957.09
	2013	64.48	55.85	8.63	9408.01	380.26	3.98	78.98	20.85	73.60	2.93	1.05	86.66	7.95	3.96	41.60	92.70	-	89954.44
	2014	65.24	56.49	8.76	9508.15	321.33	3.44	69.17	21.87	68.38	2.35	1.09	86.92	7.75	3.91	41.80	93.20	-	89952.01
	2015	65.92	57.04	8.87	9598.30	-	-	-	-	-	-	-	87.16	7.56	3.85	41.90	93.20	-	89951.22
	2016	66.53	57.58	8.94	9569.45	-	-	-	-	-	-	-	87.37	7.51	-	-	-	-	89959.82
	2017	68.39	58.74	9.66	-	-	-	-	-	-	-	-	-	-	-	-	-	-	-
Gambia	1995	62.66	54.12	8.54	491.59	24.03	3.26	53.64	36.50	31.96	1.04	2.22	43.36	5.45	6.04	60.30	79.30	1244.17	87832.44
	1996	62.85	54.30	8.55	488.56	26.06	3.37	53.79	39.47	26.62	0.90	2.47	44.26	5.44	6.02	60.30	80.00	1240.74	87831.37

	1997	62.84	54.30	8.54	498.20	22.21	3.11	53.63	37.76	29.58	0.92	2.19	45.16	5.41	6.00	60.10	80.70	1251.17	87847.28
	1998	63.19	54.60	8.59	501.08	25.64	3.54	53.72	35.57	33.79	1.19	2.34	46.06	5.38	5.98	60.00	81.30	2088.65	87867.87
	1999	63.17	54.60	8.57	517.78	24.34	3.56	53.70	34.83	35.15	1.25	2.31	46.96	5.33	5.97	59.80	82.00	2714.85	87900.52
	2000	63.30	54.70	8.60	530.10	23.02	3.61	53.74	35.37	34.17	1.23	2.38	47.87	5.28	5.95	59.70	82.70	2864.99	87933.85
	2001	63.56	54.94	8.62	543.78	20.12	3.71	53.60	31.14	41.90	1.55	2.15	48.78	5.21	5.93	59.60	83.30	3730.61	87920.38
	2002	63.49	54.90	8.59	509.72	16.59	3.75	53.72	33.56	37.52	1.41	2.34	49.68	5.12	5.91	59.50	84.00	3407.84	87937.35
	2003	63.95	55.26	8.69	527.50	15.22	4.22	51.18	25.42	50.34	2.12	2.10	50.59	5.04	5.88	59.40	84.60	2438.47	87946.87
	2004	64.21	55.47	8.74	546.77	18.06	4.35	48.23	20.36	57.79	2.51	1.84	51.48	4.97	5.86	59.30	85.20	3115.45	87977.16
	2005	64.25	55.51	8.75	524.51	21.52	4.97	49.27	20.53	58.34	2.90	2.07	52.34	4.93	5.84	59.20	85.90	3001.60	88004.75
	2006	64.48	55.72	8.76	513.75	24.32	5.52	50.06	20.65	58.75	3.24	2.28	53.18	4.93	5.81	59.10	86.50	3751.61	88031.14
	2007	64.48	55.74	8.75	515.77	29.96	5.76	50.69	59.07	59.07	3.40	2.36	54.00	4.95	5.79	59.00	87.10	4029.42	88065.00
	2008	64.89	56.08	8.81	528.37	36.19	5.95	51.20	20.83	59.32	3.53	2.42	54.79	4.96	5.76	58.90	87.70	3350.44	88102.45
	2009	65.10	56.26	8.84	544.96	32.15	5.85	51.61	20.89	59.53	3.48	2.37	55.56	4.95	5.74	58.90	88.30	3012.98	88158.59
	2010	65.52	56.62	8.90	562.48	32.33	5.75	51.96	20.94	59.70	3.43	2.32	56.30	4.89	5.71	58.80	88.90	3481.57	88210.02
	2011	65.96	57.00	8.96	521.61	32.27	6.24	52.26	20.98	59.85	3.74	2.51	57.01	4.83	5.68	58.80	89.50	3505.92	88296.12
	2012	66.27	57.28	8.99	533.78	30.92	6.12	52.52	21.02	59.97	3.67	2.45	57.71	4.73	5.64	58.70	90.10	3529.23	88376.68
	2013	66.51	57.51	9.00	542.13	30.99	6.49	54.67	19.45	64.42	4.18	2.31	58.37	4.62	5.60	58.80	90.10	-	88464.24
	2014	66.73	57.71	9.02	530.32	30.72	7.34	54.53	17.05	68.74	5.05	2.29	59.02	4.54	5.54	58.80	90.20	-	88575.08
	2015	67.04	57.99	9.05	536.41	-	-	-	-	-	-	-	59.63	4.48	5.49	58.90	90.20	-	88666.83
	2016	67.34	58.27	9.07	531.92	-	-	-	-	-	-	-	60.22	4.47	-	-	-	-	88787.83
	2017	65.77	56.72	9.05	-	-	-	-	-	-	-	-	-	-	-	-	-	-	-
Georgia	1995	70.55	62.27	8.28	1010.25	29.58	5.12	100.00	94.78	5.22	0.27	4.86	53.84	17.74	1.88	97.80	85.80	13616.27	91443.64
	1996	71.27	62.88	8.39	1152.09	45.98	7.49	100.00	88.33	11.67	0.87	6.62	53.60	18.24	1.81	97.70	86.50	12931.43	91592.78
	1997	71.42	63.03	8.38	1297.02	57.00	7.99	99.93	81.28	18.66	1.49	6.50	53.36	18.51	1.75	97.60	87.20	12076.96	91752.61
	1998	71.47	63.11	8.36	1350.50	48.68	6.55	99.89	81.65	18.26	1.20	5.35	53.12	18.64	1.69	96.90	87.90	11244.97	91889.10
	1999	71.62	63.27	8.35	1400.10	36.70	6.29	99.83	81.74	18.12	1.14	5.15	52.88	18.81	1.64	96.30	88.60	10982.37	92026.92
	2000	71.81	63.46	8.36	1436.88	44.99	6.94	99.43	82.51	17.01	1.18	5.76	52.64	19.10	1.61	95.70	89.30	11554.31	92133.30
	2001	72.09	63.69	8.41	1516.88	54.02	7.82	87.88	72.27	17.76	1.39	6.43	52.40	19.67	1.59	95.00	90.00	9788.14	92141.87
	2002	71.95	63.58	8.37	1610.71	64.33	8.72	84.80	71.19	16.05	1.40	7.32	52.29	20.35	1.59	94.40	90.80	20647.47	92144.38

	2003	71.82	63.51	8.31	1812.11	74.42	8.49	90.47	77.06	14.82	1.26	7.23	52.35	21.03	1.60	93.70	91.50	9653.44	92150.72
	2004	71.69	63.40	8.29	1943.56	96.98	8.53	91.40	77.40	15.32	1.31	7.22	52.41	21.50	1.63	93.10	92.30	10168.48	92167.66
	2005	72.08	63.67	8.41	2158.09	123.40	8.60	95.05	76.83	19.17	1.65	6.95	52.47	21.68	1.66	92.50	93.00	11190.17	92156.15
	2006	72.45	63.95	8.51	2391.41	147.35	8.39	91.54	72.22	21.11	1.77	6.62	52.53	21.82	1.71	91.80	93.80	11840.24	92104.07
	2007	72.70	64.13	8.57	2722.15	189.93	8.17	86.07	17.75	17.75	1.45	6.72	52.58	21.73	1.77	91.20	94.60	12884.99	92035.96
	2008	72.31	63.78	8.53	2821.08	264.76	8.99	80.09	64.24	19.79	1.78	7.21	52.63	21.50	1.82	90.50	95.30	12282.14	91959.62
	2009	72.66	64.06	8.60	2753.64	255.28	10.19	85.62	66.55	22.27	2.27	7.92	52.72	21.28	1.87	89.90	96.10	13250.12	91883.24
	2010	72.78	64.14	8.64	2964.48	274.86	10.05	89.50	69.11	22.78	2.29	7.76	52.87	21.15	1.92	89.30	96.80	13108.62	91812.30
	2011	72.90	64.23	8.66	3220.39	323.43	9.38	79.25	64.89	18.13	1.70	7.68	53.02	21.14	1.95	88.70	97.60	14665.80	91731.75
	2012	73.07	64.37	8.70	3469.67	327.69	8.57	72.67	62.41	14.12	1.21	7.36	53.16	21.22	1.98	88.10	98.30	14627.75	91655.72
	2013	73.22	64.48	8.74	3633.74	286.07	7.25	74.09	61.05	17.61	1.28	5.97	53.31	21.38	2.00	87.50	99.00	-	91579.27
	2014	73.42	64.63	8.79	3851.72	302.60	7.42	74.09	58.58	20.94	1.55	5.86	53.47	21.62	2.00	86.90	99.60	-	91521.84
	2015	73.64	64.79	8.85	3973.22	-	-	-	-	-	-	-	53.64	21.92	2.00	86.30	100.00	-	91482.77
	2016	73.92	65.01	8.90	4084.00	-	-	-	-	-	-	-	53.83	22.21	-	-	-	-	91467.93
	2017	77.17	65.53	11.64	-	-	-	-	-	-	-	-	-	-	-	-	-	-	-
Germany	1995	76.64	66.70	9.94	34782.57	3128.74	9.43	53.62	9.96	81.42	7.68	1.75	73.29	22.65	1.25	99.20	100.00	1131718.77	92014.00
	1996	76.89	66.92	9.97	34965.69	3107.37	9.91	54.36	9.85	81.88	8.12	1.80	73.23	22.97	1.30	99.20	100.00	1153137.92	91976.31
	1997	77.18	67.17	10.01	35560.21	2702.66	10.03	54.74	10.63	80.58	8.09	1.95	73.17	23.22	1.35	99.20	100.00	1122917.37	91950.40
	1998	77.45	67.41	10.05	36258.67	2728.66	10.05	55.94	11.18	80.01	8.04	2.01	73.10	23.46	1.36	99.20	100.00	1060398.26	91924.76
	1999	77.73	67.64	10.09	36955.29	2694.64	10.05	55.79	11.31	79.74	8.01	2.04	73.06	23.79	1.36	99.20	100.00	1020391.26	91908.09
	2000	78.02	67.88	10.14	37998.43	2397.75	10.10	56.79	11.83	79.17	8.00	2.10	73.07	24.28	1.38	99.20	100.00	1016295.55	91924.02
	2001	78.30	68.10	10.20	38577.73	2407.87	10.15	56.53	11.80	79.12	8.03	2.12	73.11	24.97	1.35	99.20	100.00	1025983.50	92009.86
	2002	78.52	68.28	10.23	38512.92	2622.98	10.40	56.82	12.12	78.67	8.18	2.22	73.17	25.80	1.34	99.20	100.00	1007404.93	92095.76
	2003	78.72	68.44	10.27	38218.35	3222.75	10.62	57.05	12.50	78.09	8.29	2.33	73.23	26.69	1.34	99.20	100.00	1017485.98	92183.55
	2004	79.02	68.68	10.34	38673.89	3542.82	10.37	58.81	13.90	76.37	7.92	2.45	73.29	27.53	1.36	99.20	100.00	1016628.79	92274.03
	2005	79.25	68.87	10.38	38969.32	3647.79	10.52	58.62	13.99	76.13	8.01	2.51	73.36	28.26	1.34	99.20	100.00	981287.68	92369.17
	2006	79.49	69.06	10.42	40456.86	3766.11	10.34	58.82	14.12	75.99	7.86	2.48	73.49	29.03	1.33	99.20	100.00	994686.05	92389.79
	2007	79.67	69.20	10.46	41831.87	4254.23	10.18	58.46	75.95	75.95	7.73	2.45	73.70	29.69	1.37	99.20	100.00	972896.23	92407.40
	2008	79.83	69.35	10.48	42365.10	4743.27	10.39	57.50	13.78	76.03	7.90	2.49	73.90	30.25	1.38	99.20	100.00	992116.30	92423.67

	2009	79.97	69.46	10.51	40086.10	4752.82	11.40	57.41	13.56	76.38	8.71	2.69	74.09	30.74	1.36	99.20	100.00	950463.41	92441.07
	2010	80.14	69.60	10.54	41785.56	4700.09	11.25	57.84	13.75	76.22	8.58	2.68	74.29	31.17	1.39	99.20	100.00	948006.75	92457.95
	2011	80.32	69.74	10.59	44125.33	5025.25	10.93	57.39	13.78	75.99	8.31	2.63	74.49	31.40	1.39	99.20	100.00	929540.26	92554.11
	2012	80.48	69.87	10.61	44259.26	4753.88	10.99	57.80	13.83	76.07	8.36	2.63	74.69	31.56	1.41	99.20	100.00	951716.71	92642.73
	2013	80.57	69.95	10.63	44354.74	5098.24	11.16	56.77	13.20	76.75	8.57	2.60	74.89	31.69	1.42	99.20	100.00	-	92723.44
	2014	80.79	70.11	10.68	45022.57	5410.63	11.30	57.35	13.20	76.99	8.70	2.60	75.09	31.86	1.47	99.20	100.00	-	92807.57
	2015	80.87	70.17	10.69	45412.56	-	-	-	-	-	-	-	75.30	32.11	1.50	99.20	100.00	-	92893.87
	2016	80.93	70.23	10.70	45745.79	-	-	-	-	-	-	-	75.51	32.40	-	-	-	-	92998.43
	2017	80.63	69.55	11.09	-	-	-	-	-	-	-	-	-	-	-	-	-	-	-
Ghana	1995	59.46	51.90	7.56	886.48	19.07	3.09	64.24	30.46	52.58	1.62	1.46	40.14	5.48	5.17	8.60	63.20	30510.82	87741.10
	1996	59.48	51.96	7.52	904.34	22.29	3.45	63.88	26.45	58.60	2.02	1.43	40.90	5.49	5.10	9.00	64.70	30600.45	87771.45
	1997	59.06	51.65	7.41	919.64	18.83	3.00	64.79	33.74	47.93	1.44	1.56	41.65	5.49	5.04	9.30	66.20	31402.41	87821.99
	1998	59.36	51.92	7.44	940.04	20.55	3.23	63.40	28.62	54.86	1.77	1.46	42.42	5.48	4.97	9.60	67.60	130472.49	87871.18
	1999	59.30	51.89	7.40	957.97	19.87	3.08	63.15	30.49	51.72	1.59	1.49	43.18	5.46	4.90	10.00	69.10	94526.40	87908.19
	2000	59.31	51.92	7.39	969.22	12.27	3.00	63.56	31.80	49.97	1.50	1.50	43.93	5.43	4.83	10.30	70.50	107699.83	87943.88
	2001	59.22	51.90	7.32	982.93	15.25	3.56	63.92	27.19	57.46	2.05	1.51	44.60	5.56	4.75	10.60	71.80	104386.21	87936.03
	2002	59.31	52.00	7.31	1001.24	14.98	3.07	62.80	31.97	49.10	1.51	1.56	45.28	5.69	4.68	11.00	73.10	92865.73	87936.23
	2003	59.53	52.19	7.34	1026.40	18.48	3.12	64.62	31.92	50.60	1.58	1.54	45.95	5.80	4.61	11.30	74.50	108030.31	87915.34
	2004	59.84	52.44	7.40	1056.00	26.70	3.96	64.97	25.58	60.62	2.40	1.56	46.63	5.91	4.55	11.60	75.80	71082.31	87905.90
	2005	60.13	52.70	7.43	1089.47	36.03	4.51	64.89	22.80	64.86	2.92	1.58	47.31	6.00	4.49	12.00	77.00	113159.71	87893.76
	2006	60.46	53.00	7.45	1129.24	43.11	4.64	65.53	22.50	65.66	3.04	1.59	47.99	6.08	4.44	12.30	78.30	104044.73	87877.09
	2007	60.79	53.28	7.51	1147.87	58.25	5.30	65.81	70.37	70.37	3.73	1.57	48.67	6.12	4.39	12.60	79.50	107333.77	87866.33
	2008	61.27	53.70	7.57	1220.71	59.86	4.85	66.16	22.20	66.45	3.22	1.63	49.35	6.15	4.35	13.00	80.70	124072.65	87853.45
	2009	61.72	54.08	7.64	1247.46	56.67	5.17	65.31	18.91	71.04	3.67	1.50	50.03	6.16	4.31	13.30	81.90	104912.64	87827.04
	2010	62.16	54.48	7.68	1312.61	70.58	5.33	65.36	18.44	71.78	3.83	1.51	50.71	6.17	4.27	13.70	83.10	106432.69	87796.14
	2011	62.91	55.14	7.77	1460.65	76.30	4.81	63.01	16.15	74.38	3.58	1.23	51.39	6.10	4.23	14.00	84.30	107126.22	87703.18
	2012	63.71	55.85	7.85	1558.46	78.64	4.79	77.24	26.60	65.57	3.14	1.65	52.07	6.04	4.19	14.40	85.40	107784.29	87650.62
	2013	64.34	56.42	7.92	1633.49	84.53	4.63	66.59	19.92	70.08	3.24	1.38	52.74	5.99	4.15	14.70	86.50	-	87617.54
	2014	64.87	56.89	7.98	1659.78	57.89	3.56	66.85	26.84	59.85	2.13	1.43	53.39	5.93	4.10	14.80	87.60	-	87595.77

	2015	65.43	57.37	8.05	1685.99	-	-	-	-	-	-	-	54.04	5.86	4.04	14.90	88.70	-	87608.16
	2016	66.01	57.92	8.08	1707.66	-	-	-	-	-	-	-	54.68	5.85	-	-	-	-	87608.88
	2017	65.50	57.04	8.46	-	-	-	-	-	-	-	-	-	-	-	-	-	-	-
Greece	1995	77.79	68.05	9.74	19909.53	1067.87	8.27	90.59	43.45	52.04	4.30	3.96	72.12	21.65	1.28	94.00	98.20	99302.95	92257.36
	1996	77.94	68.19	9.75	20389.32	1116.37	8.17	89.70	42.20	52.95	4.33	3.84	72.24	22.12	1.26	94.30	98.30	102354.44	92337.20
	1997	78.10	68.34	9.76	21198.79	1067.18	8.01	89.61	42.26	52.84	4.23	3.78	72.36	22.60	1.27	94.60	98.50	106166.94	92430.69
	1998	78.23	68.45	9.78	21902.74	1057.22	7.91	89.16	42.74	52.06	4.12	3.79	72.48	23.09	1.24	94.90	98.60	113865.03	92523.59
	1999	78.36	68.57	9.80	22489.32	1115.87	8.13	88.57	41.26	53.42	4.34	3.79	72.60	23.57	1.23	95.20	98.70	109857.75	92619.39
	2000	78.62	68.77	9.85	23275.44	918.43	7.60	85.93	34.36	60.01	4.56	3.04	72.72	24.04	1.25	95.50	98.80	115225.46	92737.31
	2001	78.89	68.99	9.90	24111.42	1055.01	8.47	82.57	32.40	60.76	5.15	3.32	72.91	24.64	1.25	95.80	98.90	114019.90	92677.56
	2002	79.09	69.14	9.95	24965.59	1222.75	8.72	83.36	35.02	57.99	5.06	3.66	73.30	25.23	1.28	96.20	99.10	115164.42	92622.88
	2003	79.27	69.27	9.99	26349.28	1580.46	8.61	85.18	34.25	59.79	5.15	3.46	73.69	25.77	1.29	96.50	99.20	118731.24	92588.74
	2004	79.44	69.39	10.05	27614.41	1809.35	8.30	86.16	35.22	59.12	4.91	3.39	74.07	26.22	1.31	96.80	99.30	118117.94	92555.75
	2005	79.60	69.50	10.10	27698.51	2094.78	9.36	87.31	34.83	60.11	5.63	3.73	74.45	26.56	1.34	97.10	99.40	120036.21	92534.34
	2006	79.85	69.68	10.17	29176.39	2296.05	9.34	86.57	32.87	62.03	5.79	3.54	74.83	26.84	1.40	97.40	99.60	117313.29	92484.72
	2007	79.93	69.73	10.19	30054.89	2689.06	9.41	87.16	60.35	60.35	5.68	3.73	75.20	26.99	1.41	97.70	99.70	127392.26	92427.72
	2008	80.16	69.90	10.26	29874.74	3093.58	9.76	94.53	37.86	59.94	5.85	3.91	75.57	27.08	1.50	98.00	99.80	117310.66	92372.98
	2009	80.27	69.97	10.30	28514.81	2879.08	9.76	93.16	28.43	69.46	6.78	2.98	75.93	27.22	1.50	98.20	99.90	113365.80	92319.50
	2010	80.39	70.05	10.35	26917.76	2458.73	9.18	90.96	29.36	67.68	6.22	2.96	76.29	27.50	1.48	98.50	100.00	107505.98	92301.68
	2011	80.48	70.14	10.34	24495.71	2528.39	9.77	91.32	28.71	68.39	6.68	3.07	76.65	27.94	1.40	98.80	100.00	105013.93	92397.27
	2012	80.58	70.24	10.34	22830.53	2048.93	9.24	90.18	28.84	67.89	6.27	2.96	77.00	28.51	1.34	99.00	100.00	100571.17	92538.51
	2013	80.82	70.44	10.37	22251.26	2017.22	9.26	90.95	30.67	65.53	6.07	3.12	77.34	29.16	1.29	99.00	100.00	-	92725.01
	2014	80.93	70.55	10.38	22565.68	1743.04	8.08	90.94	34.86	61.66	4.99	3.10	77.68	29.83	1.30	99.00	100.00	-	92927.55
	2015	80.96	70.60	10.36	22648.77	-	-	-	-	-	-	-	78.01	30.45	1.30	99.00	100.00	-	93133.32
	2016	80.97	70.63	10.34	22749.31	-	-	-	-	-	-	-	78.33	30.84	-	-	-	-	93322.67
	2017	80.99	69.93	11.06	-	-	-	-	-	-	-	-	-	-	-	-	-	-	-
Greenland	1995	63.98	56.17	7.81	26899.80	-	-	-	-	-	-	-	80.90	-	2.51	100.00	100.00	-	86070.61
	1996	64.09	56.28	7.81	27262.41	-	-	-	-	-	-	-	81.10	-	2.49	100.00	100.00	-	86171.95
	1997	64.20	56.39	7.82	27613.48	-	-	-	-	-	-	-	81.23	-	2.69	100.00	100.00	-	86290.24

	1998	64.47	56.62	7.84	29702.69	-	-	-	-	-	-	-	81.35	-	2.46	100.00	100.00	-	86429.39
	1999	64.70	56.81	7.88	30111.96	-	-	-	-	-	-	-	81.48	-	2.42	100.00	100.00	-	86589.68
	2000	65.05	57.11	7.94	32193.00	-	-	-	-	-	-	-	81.60	-	2.33	100.00	100.00	-	86758.58
	2001	65.22	57.24	7.98	32514.76	-	-	-	-	-	-	-	81.85	-	2.51	100.00	100.00	-	86877.15
	2002	65.46	57.44	8.02	32051.67	-	-	-	-	-	-	-	82.10	-	2.57	100.00	100.00	-	87022.30
	2003	65.57	57.53	8.04	31842.24	-	-	-	-	-	-	-	82.34	-	2.37	100.00	100.00	-	87126.45
	2004	65.71	57.67	8.05	33899.13	-	-	-	-	-	-	-	82.58	-	2.42	100.00	100.00	-	87310.43
	2005	66.05	57.97	8.08	34814.54	-	-	-	-	-	-	-	82.86	-	2.38	100.00	100.00	-	87445.24
	2006	66.76	58.54	8.22	37394.62	-	-	-	-	-	-	-	83.17	-	2.26	100.00	100.00	-	87576.48
	2007	67.45	59.07	8.38	37763.90	-	-	-	-	-	-	-	83.48	-	2.30	100.00	100.00	-	87683.38
	2008	67.86	59.41	8.46	39281.01	-	-	-	-	-	-	-	83.79	-	2.25	100.00	100.00	-	87802.14
	2009	68.10	59.59	8.51	39870.42	-	-	-	-	-	-	-	84.09	-	2.36	100.00	100.00	-	87928.17
	2010	68.30	59.78	8.52	40532.51	-	-	-	-	-	-	-	84.38	-	2.20	100.00	100.00	-	88014.28
	2011	68.95	60.26	8.69	41147.97	-	-	-	-	-	-	-	84.76	-	2.12	100.00	100.00	-	88064.87
	2012	69.72	60.89	8.84	41831.88	-	-	-	-	-	-	-	85.20	-	2.02	100.00	100.00	-	88141.05
	2013	70.09	61.19	8.89	40827.78	-	-	-	-	-	-	-	85.63	-	2.08	100.00	100.00	-	88227.20
	2014	70.19	61.30	8.88	40629.96	-	-	-	-	-	-	-	86.05	-	2.04	100.00	100.00	-	88275.15
	2015	70.07	61.23	8.84	41435.63	-	-	-	-	-	-	-	86.44	-	2.00	100.00	100.00	-	88290.41
	2016	70.00	61.26	8.74	-	-	-	-	-	-	-	-	86.81	-	-	-	-	-	88310.02
	2017	73.55	63.47	10.08	-	-	-	-	-	-	-	-	-	-	-	-	-	-	-
Grenada	1995	71.48	62.63	8.85	4690.73	190.35	6.91	100.00	56.47	43.53	3.01	3.90	34.55	14.75	3.12	98.00	96.60	177.64	90927.95
	1996	71.48	62.65	8.83	4872.62	203.25	6.95	100.00	56.61	43.39	3.02	3.94	34.81	14.64	2.99	98.00	96.60	197.29	90953.18
	1997	71.39	62.58	8.81	5100.51	218.75	7.25	100.00	56.47	43.53	3.16	4.10	35.07	14.41	2.87	98.00	96.60	208.01	90989.22
	1998	71.31	62.52	8.79	5688.94	247.11	7.35	100.00	57.09	42.91	3.15	4.19	35.34	14.11	2.76	98.00	96.60	205.60	91035.03
	1999	71.30	62.50	8.80	6073.99	291.28	7.78	100.00	52.37	47.63	3.71	4.08	35.60	13.82	2.67	98.00	96.60	221.83	91102.78
	2000	71.25	62.46	8.79	6359.78	339.02	6.62	100.00	47.97	52.03	3.45	3.18	35.87	13.58	2.58	98.00	96.60	474.97	91180.92
	2001	71.15	62.38	8.77	6217.00	391.89	7.67	100.00	41.15	58.85	4.51	3.16	36.11	13.30	2.51	98.00	96.60	444.69	91266.88
	2002	71.02	62.27	8.75	6414.89	353.18	6.67	100.00	47.19	52.81	3.52	3.15	36.06	13.04	2.46	98.00	96.60	479.73	91355.70
	2003	71.01	62.26	8.75	7003.13	340.27	5.89	100.00	48.17	51.83	3.06	2.84	36.01	12.81	2.41	98.00	96.60	473.34	91439.33

	2004	70.57	61.89	8.69	6938.74	305.53	5.24	100.00	48.41	51.59	2.70	2.53	35.96	12.56	2.37	98.00	96.60	501.04	91511.16
	2005	71.05	62.29	8.77	7837.35	371.77	5.50	96.70	45.42	53.03	2.92	2.59	35.92	12.29	2.34	98.00	96.60	562.25	91583.09
	2006	71.14	62.37	8.77	7501.84	405.41	5.99	96.95	45.05	53.53	3.21	2.78	35.87	12.06	2.32	98.00	96.60	628.85	91627.31
	2007	71.30	62.49	8.81	7936.04	430.27	5.88	97.38	49.32	49.32	2.90	2.98	35.82	11.81	2.30	98.00	96.60	625.76	91671.57
	2008	71.35	62.53	8.82	7984.84	457.99	5.76	97.66	52.05	46.70	2.69	3.07	35.78	11.57	2.28	98.00	96.60	643.73	91697.36
	2009	71.41	62.57	8.85	7430.56	461.69	6.24	97.75	49.64	49.22	3.07	3.17	35.73	11.33	2.26	98.00	96.60	645.08	91733.86
	2010	71.32	62.46	8.85	7365.67	471.95	6.41	96.79	49.95	48.40	3.10	3.31	35.68	11.11	2.24	98.00	96.60	682.66	91774.58
	2011	71.32	62.46	8.86	7393.89	470.47	6.35	96.70	50.40	47.88	3.04	3.31	35.63	11.01	2.22	98.00	96.60	705.68	91805.05
	2012	71.27	62.42	8.85	7280.34	482.12	6.36	96.66	50.34	47.92	3.05	3.31	35.60	10.91	2.19	98.00	96.60	726.19	91847.70
	2013	71.28	62.43	8.85	7421.48	485.42	6.15	94.96	52.01	45.23	2.78	3.37	35.58	10.83	2.17	98.00	96.60	-	91876.13
	2014	71.29	62.44	8.85	7932.67	505.83	6.10	94.96	50.93	46.37	2.83	3.27	35.58	10.79	2.15	98.00	96.60	-	91916.59
	2015	71.33	62.48	8.85	8406.77	-	-	-	-	-	-	-	35.59	10.78	2.13	98.00	96.60	-	91965.88
	2016	71.56	62.69	8.87	8676.34	-	-	-	-	-	-	-	35.62	10.88	-	-	-	-	92010.18
	2017	74.11	65.24	8.87	-	-	-	-	-	-	-	-	-	-	-	-	-	-	-
Guam	1995	73.33	64.50	8.83	-	-	-	-	-	-	-	-	92.06	7.22	2.87	88.90	99.60	67.20	85061.19
	1996	73.49	64.61	8.88	-	-	-	-	-	-	-	-	92.29	7.47	2.87	88.90	99.60	68.49	85116.59
	1997	69.67	61.46	8.21	-	-	-	-	-	-	-	-	92.51	7.69	2.87	89.00	99.60	69.80	85192.43
	1998	73.69	64.74	8.95	-	-	-	-	-	-	-	-	92.73	7.90	2.86	89.10	99.60	66.97	85293.63
	1999	73.79	64.80	8.99	-	-	-	-	-	-	-	-	92.95	8.11	2.85	89.10	99.60	68.24	85378.50
	2000	73.89	64.86	9.03	-	-	-	-	-	-	-	-	93.13	8.34	2.82	89.20	99.60	69.55	85488.68
	2001	73.93	64.86	9.06	-	-	-	-	-	-	-	-	93.23	8.71	2.79	89.20	99.60	70.86	85584.43
	2002	73.93	64.85	9.08	27723.64	-	-	-	-	-	-	-	93.33	9.08	2.76	89.30	99.60	72.25	85682.53
	2003	73.84	64.75	9.08	27883.24	-	-	-	-	-	-	-	93.44	9.44	2.72	89.30	99.60	73.51	85796.38
	2004	73.64	64.58	9.06	29604.15	-	-	-	-	-	-	-	93.53	9.75	2.68	89.40	99.60	74.84	85901.44
	2005	73.47	64.43	9.03	30592.16	-	-	-	-	-	-	-	93.63	10.00	2.64	89.40	99.60	76.08	86031.69
	2006	73.20	64.22	8.99	29375.28	-	-	-	-	-	-	-	93.73	10.25	2.60	89.50	99.60	77.38	86149.96
	2007	72.83	63.90	8.94	29576.90	-	-	-	-	-	-	-	93.82	10.43	2.56	89.60	99.60	78.75	86271.86
	2008	72.54	63.65	8.89	30054.20	-	-	-	-	-	-	-	93.92	10.61	2.53	89.60	99.50	80.21	86405.87
	2009	72.31	63.46	8.86	30111.68	-	-	-	-	-	-	-	94.01	10.84	2.50	89.70	99.50	81.48	86534.47

	2010	72.14	63.29	8.85	30700.43	-	-	-	-	-	-	-	94.10	11.14	2.47	89.70	99.50	83.62	86675.40
	2011	72.13	63.27	8.86	30693.13	-	-	-	-	-	-	-	94.19	11.54	2.45	89.80	99.50	84.77	86832.68
	2012	72.06	63.19	8.87	31257.16	-	-	-	-	-	-	-	94.27	12.01	2.43	89.80	99.50	85.87	86983.83
	2013	72.05	63.16	8.89	31754.11	-	-	-	-	-	-	-	94.36	12.52	2.41	89.80	99.50	-	87137.05
	2014	72.10	63.18	8.92	32129.52	-	-	-	-	-	-	-	94.44	13.05	2.39	89.80	99.50	-	87300.18
	2015	72.15	63.19	8.96	32119.65	-	-	-	-	-	-	-	94.52	13.56	2.37	89.80	99.50	-	87448.13
	2016	72.34	63.31	9.03	32013.77	-	-	-	-	-	-	-	94.59	14.05	-	-	-	-	87593.56
	2017	73.09	63.63	9.46	-	-	-	-	-	-	-	-	-	-	-	-	-	-	-
Guatemala	1995	66.60	58.46	8.15	2355.89	55.46	3.92	92.42	55.43	40.02	1.57	2.35	43.11	7.18	5.09	50.90	80.30	19535.94	85790.17
	1996	67.20	58.92	8.28	2371.31	57.34	3.86	92.36	57.05	38.23	1.47	2.38	43.51	7.30	4.99	51.70	81.00	19900.95	85759.55
	1997	67.57	59.24	8.33	2420.03	65.83	4.02	92.25	54.37	41.06	1.65	2.37	43.91	7.41	4.90	52.40	81.70	19295.85	85753.02
	1998	67.80	59.42	8.38	2484.76	67.23	3.86	92.42	52.01	43.72	1.69	2.17	44.32	7.49	4.80	53.10	82.40	87558.71	85750.48
	1999	68.26	59.80	8.47	2522.86	68.83	4.29	84.96	48.84	42.51	1.82	2.46	44.72	7.56	4.70	53.80	83.10	23117.74	85746.62
	2000	68.64	60.10	8.54	2554.78	86.67	5.25	89.41	56.96	36.30	1.91	3.35	45.13	7.62	4.60	54.60	83.90	70005.69	85771.18
	2001	68.94	60.41	8.54	2554.26	105.54	6.76	86.84	59.02	32.04	2.17	4.59	45.53	7.68	4.49	55.30	84.60	21772.12	85795.42
	2002	69.31	60.69	8.62	2591.33	113.54	6.71	86.04	58.75	31.71	2.13	4.58	45.94	7.72	4.37	56.00	85.20	31424.57	85817.64
	2003	69.58	60.91	8.67	2594.93	120.00	6.88	86.18	58.00	32.70	2.25	4.63	46.34	7.75	4.25	56.70	85.90	55215.32	85874.97
	2004	69.73	61.04	8.68	2614.71	126.05	6.77	85.50	57.87	32.31	2.19	4.58	46.76	7.77	4.11	57.40	86.60	24367.66	85940.58
	2005	69.82	61.14	8.69	2638.29	140.14	6.79	85.80	58.61	31.70	2.15	4.64	47.17	7.77	3.97	58.10	87.30	41739.13	86023.00
	2006	70.19	61.48	8.71	2717.76	158.48	7.07	86.57	57.32	33.79	2.39	4.68	47.59	7.75	3.83	58.80	88.00	27001.20	86088.16
	2007	70.55	61.77	8.77	2825.14	169.51	6.86	85.41	33.08	33.08	2.27	4.59	48.02	7.72	3.70	59.50	88.70	34084.60	86153.77
	2008	70.84	62.04	8.80	2854.07	184.46	6.65	85.32	55.55	34.89	2.32	4.33	48.45	7.69	3.58	60.20	89.40	32857.91	86255.95
	2009	71.08	62.26	8.82	2806.98	177.64	6.79	85.05	53.51	37.08	2.52	4.27	48.88	7.65	3.47	60.90	90.00	37118.66	86363.91
	2010	71.29	62.50	8.80	2825.52	186.27	6.64	85.04	54.61	35.78	2.38	4.26	49.32	7.61	3.38	61.60	90.70	30327.55	86467.02
	2011	71.55	62.71	8.85	2880.41	198.98	6.28	84.62	55.62	34.26	2.15	4.13	49.77	7.59	3.29	62.20	91.40	30950.25	86534.05
	2012	71.87	63.00	8.88	2903.40	207.52	6.33	83.89	55.42	33.93	2.15	4.18	50.21	7.56	3.22	62.90	92.00	31515.45	86608.94
	2013	72.15	63.24	8.91	2947.97	216.09	6.30	83.32	52.60	36.88	2.32	3.97	50.66	7.54	3.15	63.60	92.70	-	86683.92
	2014	72.37	63.45	8.92	3007.90	232.63	6.20	83.74	52.22	37.64	2.33	3.87	51.12	7.56	3.09	63.70	92.70	-	86756.08
	2015	72.59	63.66	8.93	3069.04	-	-	-	-	-	-	-	51.57	7.61	3.03	63.90	92.80	-	86814.37

	2016	72.75	63.86	8.89	3100.21	-	-	-	-	-	-	-	52.03	7.68	-	-	-	-	86855.64
	2017	72.63	63.67	8.96	-	-	-	-	-	-	-	-	-	-	-	-	-	-	-
Guinea	1995	53.34	46.22	7.13	529.37	16.59	3.53	87.94	56.95	35.24	1.24	2.29	29.48	6.74	6.39	10.60	57.60	62537.28	89929.42
	1996	53.83	46.67	7.16	535.22	14.90	3.13	87.96	66.34	24.58	0.77	2.36	29.77	6.74	6.33	11.00	58.60	61747.24	89875.93
	1997	54.12	46.94	7.18	549.08	13.87	3.05	87.94	66.23	24.70	0.75	2.30	30.07	6.72	6.27	11.50	59.60	62164.74	89824.18
	1998	54.22	47.06	7.15	558.03	14.33	3.39	87.95	63.36	27.96	0.95	2.44	30.37	6.67	6.21	11.90	60.70	116336.80	89802.70
	1999	54.30	47.17	7.14	569.23	13.67	3.42	87.94	61.32	30.27	1.03	2.38	30.69	6.60	6.15	12.40	61.70	89859.98	89768.78
	2000	54.66	47.49	7.18	573.22	11.77	3.46	87.94	60.06	31.71	1.10	2.36	31.02	6.52	6.08	12.90	62.70	84360.14	89763.55
	2001	54.82	47.70	7.12	583.42	11.54	3.65	87.94	57.36	34.77	1.27	2.38	31.36	6.48	6.02	13.40	63.70	80294.73	89744.99
	2002	55.10	47.96	7.14	602.40	12.18	3.76	87.93	57.24	34.90	1.31	2.45	31.70	6.42	5.95	13.80	64.80	102233.51	89738.95
	2003	55.50	48.31	7.19	598.62	12.80	3.45	87.90	54.40	38.11	1.31	2.13	32.06	6.33	5.88	14.30	65.80	75067.41	89742.96
	2004	55.81	48.58	7.23	600.98	12.05	3.14	87.91	63.26	28.04	0.88	2.26	32.43	6.22	5.80	14.80	66.80	76276.00	89758.88
	2005	56.17	48.89	7.28	606.87	8.57	2.82	87.91	70.62	19.67	0.55	2.27	32.81	6.09	5.73	15.30	67.80	81922.61	89768.65
	2006	56.41	49.14	7.27	609.33	8.95	3.02	87.92	68.55	22.03	0.67	2.35	33.20	6.08	5.65	15.80	68.80	101496.41	89770.20
	2007	56.70	49.40	7.30	635.17	12.53	3.08	87.95	13.59	13.59	0.42	2.66	33.60	6.06	5.58	16.30	69.80	91126.98	89777.00
	2008	57.16	49.80	7.36	646.97	13.89	3.21	87.93	66.42	24.47	0.78	2.42	34.01	6.01	5.50	16.80	70.80	135227.87	89805.81
	2009	57.52	50.11	7.40	622.94	16.56	3.85	87.94	57.17	34.99	1.35	2.50	34.43	5.94	5.42	17.30	71.80	160212.12	89821.44
	2010	57.98	50.52	7.46	635.67	19.56	4.55	87.94	49.03	44.24	2.01	2.54	34.86	5.85	5.34	17.90	72.80	100274.04	89846.41
	2011	58.48	50.96	7.53	655.92	21.18	4.73	87.96	51.20	41.79	1.98	2.75	35.30	5.82	5.26	18.40	73.80	100814.39	89830.26
	2012	58.92	51.35	7.57	679.56	26.27	5.39	88.00	53.74	38.93	2.10	3.29	35.75	5.77	5.18	18.90	74.80	101348.99	89829.11
	2013	59.38	51.76	7.62	690.67	28.65	5.49	87.99	49.89	43.29	2.38	3.11	36.21	5.70	5.09	19.40	75.70	-	89826.72
	2014	59.14	51.59	7.55	699.96	30.46	5.64	87.97	45.33	48.47	2.74	2.91	36.68	5.65	5.01	20.00	76.70	-	89834.78
	2015	60.02	52.34	7.68	707.37	-	-	-	-	-	-	-	37.16	5.63	4.93	20.10	76.80	-	89846.77
	2016	60.68	52.92	7.76	735.72	-	-	-	-	-	-	-	37.65	5.68	-	-	-	-	89862.19
	2017	60.72	52.77	7.95	-	-	-	-	-	-	-	-	-	-	-	-	-	-	-
Guyana	1995	64.95	56.77	8.17	2036.70	43.34	5.07	87.51	16.28	81.40	4.12	0.94	29.14	6.45	3.07	77.30	82.30	16107.04	90965.05
	1996	64.83	56.70	8.13	2195.62	47.79	4.95	87.94	16.07	81.73	4.04	0.90	29.05	6.53	3.07	77.70	83.10	16425.01	90947.61
	1997	64.96	56.79	8.18	2336.48	52.52	5.15	87.08	14.86	82.94	4.27	0.88	28.96	6.59	3.06	78.10	83.90	16419.27	90933.56
	1998	65.13	56.89	8.23	2304.31	51.29	5.27	87.31	15.85	81.85	4.31	0.96	28.87	6.65	3.05	78.50	84.70	12863.53	90921.22

	1999	65.36	57.05	8.31	2381.15	43.93	4.68	86.08	15.90	81.53	3.82	0.86	28.78	6.69	3.04	78.90	85.50	4579.59	90908.60
	2000	65.43	57.07	8.36	2354.53	56.15	5.85	86.08	13.14	84.74	4.95	0.89	28.69	6.74	3.02	79.20	86.20	4405.23	90899.00
	2001	65.33	57.01	8.32	2410.83	54.57	5.82	86.08	13.70	84.09	4.90	0.93	28.61	6.82	2.99	79.60	87.00	4876.15	91010.03
	2002	65.15	56.88	8.26	2437.40	56.73	5.84	86.08	13.11	84.77	4.95	0.89	28.52	6.91	2.96	80.00	87.80	5086.47	91120.90
	2003	64.90	56.69	8.20	2412.98	59.63	5.97	90.61	18.50	79.58	4.75	1.22	28.43	6.99	2.92	80.40	88.60	5790.98	91239.06
	2004	64.70	56.55	8.16	2493.14	60.66	5.73	90.61	18.67	79.40	4.55	1.18	28.36	7.02	2.88	80.80	89.40	4964.89	91355.22
	2005	64.65	56.49	8.16	2446.65	64.74	5.83	90.61	22.82	74.81	4.36	1.47	28.30	6.97	2.84	81.20	90.20	4917.33	91478.88
	2006	64.90	56.72	8.18	2576.78	91.57	4.67	90.61	31.52	65.21	3.04	1.62	28.26	7.05	2.80	81.60	91.00	4648.22	91580.41
	2007	65.01	56.82	8.19	2764.06	150.63	6.45	90.61	72.61	72.61	4.69	1.77	28.23	7.08	2.76	82.00	91.80	4939.33	91690.33
	2008	65.05	56.87	8.18	2824.57	191.83	7.46	90.61	22.66	74.99	5.60	1.87	28.22	7.10	2.72	82.40	92.60	5112.84	91797.86
	2009	65.30	57.07	8.23	2927.33	197.50	7.32	92.34	25.47	72.41	5.30	2.02	28.22	7.14	2.68	82.80	93.40	25763.69	91904.74
	2010	65.36	57.13	8.24	3044.95	198.02	6.60	92.34	29.96	67.55	4.46	2.14	28.24	7.26	2.66	83.20	94.20	5937.29	92016.07
	2011	65.58	57.32	8.26	3192.30	231.69	6.80	92.34	30.24	67.26	4.57	2.23	28.27	7.29	2.63	83.60	95.10	6042.58	92092.93
	2012	65.83	57.55	8.28	3342.92	246.81	6.57	92.34	31.31	66.10	4.34	2.23	28.32	7.37	2.61	83.60	95.90	6140.69	92148.41
	2013	66.21	57.88	8.33	3487.55	200.22	5.10	92.32	39.85	56.83	2.90	2.20	28.38	7.49	2.58	83.60	96.70	-	92203.63
	2014	66.57	58.18	8.38	3598.29	221.78	5.25	92.32	37.44	59.45	3.12	2.13	28.46	7.61	2.56	83.60	97.50	-	92260.08
	2015	66.86	58.44	8.43	3683.50	-	-	-	-	-	-	-	28.55	7.73	2.53	83.70	98.30	-	92291.20
	2016	67.74	59.17	8.57	3783.54	-	-	-	-	-	-	-	28.66	7.90	-	-	-	-	92304.19
	2017	69.21	60.44	8.77	-	-	-	-	-	-	-	-	-	-	-	-	-	-	-
Haiti	1995	55.07	48.04	7.04	-	22.77	6.60	77.04	45.54	40.89	2.70	3.90	32.59	7.47	4.89	19.20	62.20	5951.29	91400.73
	1996	55.42	48.35	7.07	757.06	24.04	6.45	83.29	51.55	38.11	2.46	3.99	33.20	7.46	4.78	19.60	61.90	6270.29	91401.53
	1997	55.66	48.55	7.10	763.50	24.79	6.20	86.25	52.25	39.41	2.44	3.76	33.79	7.44	4.67	20.00	61.70	6632.31	91387.33
	1998	56.01	48.85	7.16	766.32	27.26	5.99	73.55	48.61	33.91	2.03	3.96	34.39	7.41	4.55	20.30	61.40	6749.95	91396.12
	1999	56.33	49.12	7.21	773.42	27.50	5.65	72.84	50.97	30.02	1.70	3.96	34.99	7.37	4.43	20.70	61.10	7000.62	91400.96
	2000	56.75	49.47	7.28	766.94	25.97	6.06	69.71	50.38	27.72	1.68	4.38	35.60	7.33	4.30	21.00	60.80	7179.10	91402.92
	2001	57.08	49.78	7.30	746.41	22.73	5.63	71.77	53.00	26.15	1.47	4.16	37.25	7.35	4.18	21.60	60.80	7498.51	91400.76
	2002	57.41	50.07	7.34	732.55	19.89	5.47	71.15	52.78	25.81	1.41	4.06	38.94	7.36	4.07	22.10	60.80	7616.28	91397.87
	2003	57.56	50.22	7.34	723.60	16.74	5.32	74.27	52.12	29.82	1.59	3.73	40.65	7.36	3.96	22.60	60.80	7598.89	91403.25
	2004	56.56	49.39	7.17	687.19	22.52	5.61	75.94	48.48	36.16	2.03	3.58	42.38	7.35	3.85	23.20	60.70	7871.31	91405.73

	2005	57.97	50.59	7.38	688.70	19.77	4.41	73.08	57.52	21.29	0.94	3.47	44.08	7.34	3.76	23.70	60.60	8059.94	91422.02
	2006	58.29	50.91	7.38	693.26	29.56	5.70	54.52	41.55	23.78	1.36	4.34	45.76	7.39	3.67	24.20	60.40	8169.69	91420.48
	2007	58.81	51.36	7.45	705.39	34.72	5.56	55.97	23.87	23.87	1.33	4.23	47.39	7.44	3.58	24.60	60.20	8537.21	91427.16
	2008	59.25	51.73	7.52	700.48	39.10	5.92	50.82	39.19	22.90	1.36	4.57	48.98	7.48	3.49	25.10	59.90	8462.39	91444.81
	2009	59.99	52.35	7.64	711.25	44.07	6.68	45.66	36.28	20.55	1.37	5.31	50.52	7.49	3.41	25.50	59.60	8453.15	91470.28
	2010	31.65	29.50	2.15	662.28	54.30	8.09	40.59	33.52	17.43	1.41	6.68	52.02	7.47	3.33	25.90	59.30	8545.17	91525.96
	2011	60.49	52.82	7.67	688.84	76.73	10.41	31.00	25.63	17.34	1.81	8.61	53.46	7.49	3.25	26.30	58.90	8695.43	91553.62
	2012	61.58	53.79	7.79	698.79	75.12	9.88	30.88	27.82	9.92	0.98	8.90	54.84	7.48	3.17	26.70	58.50	8835.47	91582.86
	2013	62.46	54.57	7.89	718.42	65.15	8.10	41.13	32.37	21.28	1.72	6.38	56.17	7.47	3.10	27.10	58.00	-	91604.26
	2014	63.26	55.26	8.00	728.78	61.46	7.56	43.87	34.82	20.64	1.56	6.00	57.44	7.47	3.03	27.40	57.50	-	91624.40
	2015	63.84	55.76	8.08	728.06	-	-	-	-	-	-	-	58.65	7.51	2.97	27.60	57.70	-	91652.35
	2016	63.97	55.90	8.07	729.27	-	-	-	-	-	-	-	59.79	7.60	-	-	-	-	91667.94
	2017	64.95	56.26	8.68	-	-	-	-	-	-	-	-	-	-	-	-	-	-	-
Honduras	1995	68.50	60.66	7.84	1594.36	37.81	5.34	89.53	41.45	53.70	2.87	2.47	42.94	6.74	4.64	55.90	77.00	16822.76	86599.79
	1996	68.77	60.90	7.87	1606.98	35.37	4.96	89.55	45.14	49.59	2.46	2.50	43.44	6.77	4.53	57.40	77.80	17186.99	86677.27
	1997	68.99	61.10	7.89	1642.16	39.33	4.88	89.61	46.08	48.58	2.37	2.51	43.94	6.80	4.41	58.90	78.60	16118.76	86732.53
	1998	62.99	55.97	7.02	1645.29	49.51	5.63	85.50	41.11	51.92	2.92	2.71	44.45	6.83	4.29	60.30	79.30	22838.19	86814.35
	1999	69.34	61.40	7.94	1572.25	55.12	6.21	88.15	46.97	46.72	2.90	3.31	44.95	6.86	4.17	61.80	80.10	13997.56	86891.09
	2000	69.55	61.59	7.96	1620.06	76.28	6.63	94.96	43.47	54.22	3.59	3.03	45.46	6.88	4.06	63.30	80.80	21929.62	86972.59
	2001	69.71	61.74	7.98	1622.21	82.60	6.88	95.52	45.29	52.59	3.62	3.26	45.96	6.88	3.94	64.70	81.50	12536.91	87065.03
	2002	69.89	61.89	8.00	1641.40	88.08	7.28	95.88	44.46	53.64	3.91	3.38	46.59	6.89	3.82	66.20	82.30	14868.59	87167.45
	2003	70.03	62.02	8.01	1674.40	103.07	8.29	93.49	40.14	57.06	4.73	3.56	47.23	6.88	3.69	67.60	83.00	22792.46	87263.52
	2004	70.29	62.24	8.05	1736.69	103.63	7.89	93.20	43.50	53.33	4.21	3.68	47.88	6.88	3.57	69.10	83.80	15006.24	87363.92
	2005	70.56	62.48	8.09	1799.49	110.82	7.81	94.15	47.09	49.98	3.91	3.91	48.52	6.86	3.45	70.50	84.50	23206.91	87460.02
	2006	70.83	62.71	8.12	1874.95	118.92	7.63	94.62	51.37	45.71	3.49	4.14	49.16	6.85	3.33	71.90	85.20	16686.65	87503.16
	2007	71.08	62.90	8.17	1947.96	136.79	7.89	94.63	46.10	46.10	3.64	4.25	49.80	6.83	3.20	73.30	85.90	19178.53	87545.10
	2008	71.26	63.06	8.20	1987.91	160.65	8.40	94.60	52.65	44.34	3.73	4.68	50.44	6.81	3.09	74.70	86.60	19785.86	87584.05
	2009	71.48	63.25	8.23	1900.38	174.67	8.84	94.25	45.70	51.51	4.55	4.29	51.07	6.81	2.98	76.00	87.30	19877.56	87613.24
	2010	71.57	63.34	8.23	1932.86	178.39	8.45	94.25	47.24	49.88	4.22	4.24	51.70	6.82	2.87	77.40	88.00	19663.72	87655.35

	2011	71.80	63.52	8.28	1969.31	199.10	8.57	92.54	50.24	45.71	3.92	4.65	52.32	6.83	2.78	78.70	88.70	20084.14	87670.71
	2012	71.83	63.55	8.28	2013.48	234.23	9.78	90.44	49.25	45.54	4.45	5.33	52.93	6.85	2.69	80.00	89.30	20467.16	87707.90
	2013	72.14	63.80	8.33	2033.32	214.19	9.15	88.97	45.63	48.71	4.46	4.69	53.54	6.89	2.62	81.30	90.00	-	87753.71
	2014	72.31	63.95	8.36	2059.47	212.32	8.72	88.07	43.46	50.65	4.42	4.30	54.14	6.96	2.56	82.60	90.60	-	87800.67
	2015	72.48	64.09	8.39	2098.34	-	-	-	-	-	-	-	54.73	7.05	2.51	82.60	91.20	-	87854.06
	2016	72.67	64.27	8.40	2137.81	-	-	-	-	-	-	-	55.32	7.17	-	-	-	-	87919.08
	2017	73.98	64.91	9.07	-	-	-	-	-	-	-	-	-	-	-	-	-	-	-
Hungary	1995	70.07	61.08	8.99	8952.04	323.01	7.22	100.00	16.04	83.96	6.07	1.16	65.21	21.08	1.57	98.00	97.10	77421.42	91641.87
	1996	70.38	61.36	9.03	8968.70	315.02	6.99	100.00	18.40	81.60	5.71	1.29	65.08	21.35	1.46	98.00	97.30	80713.52	91729.71
	1997	70.77	61.68	9.08	9286.96	308.51	6.74	100.00	18.72	81.28	5.48	1.26	64.96	21.63	1.37	98.00	97.50	78760.59	91830.00
	1998	71.10	61.98	9.13	9702.89	334.76	7.08	88.44	22.30	74.78	5.29	1.78	64.83	21.88	1.32	98.00	97.70	77888.81	91926.80
	1999	71.44	62.27	9.17	10040.51	344.64	7.21	89.96	24.85	72.37	5.22	1.99	64.70	22.07	1.28	98.00	98.00	78178.00	92027.24
	2000	71.80	62.61	9.19	10490.35	325.71	7.06	89.77	26.27	70.74	4.99	2.07	64.58	22.18	1.32	98.00	98.20	75482.06	92122.16
	2001	72.22	62.91	9.30	10918.25	374.28	7.11	89.27	27.69	68.98	4.91	2.21	64.67	22.36	1.31	98.00	98.40	75969.17	92161.76
	2002	72.53	63.17	9.36	11445.19	495.38	7.47	88.22	26.28	70.21	5.24	2.22	65.08	22.46	1.30	98.00	98.60	74621.99	92214.93
	2003	72.72	63.34	9.39	11919.77	705.83	8.42	88.19	25.50	71.09	5.99	2.44	65.49	22.52	1.27	98.00	98.80	76989.23	92266.05
	2004	72.92	63.51	9.42	12544.09	828.14	8.08	81.93	24.91	69.60	5.63	2.46	65.90	22.58	1.28	98.00	99.00	75935.95	92320.66
	2005	73.13	63.74	9.39	13120.62	922.78	8.28	83.40	25.00	70.03	5.80	2.48	66.35	22.68	1.31	98.00	99.20	75543.76	92371.88
	2006	73.37	63.93	9.43	13647.15	922.33	8.10	80.12	24.22	69.77	5.65	2.45	66.86	22.84	1.34	98.00	99.40	75140.64	92347.84
	2007	73.62	64.05	9.56	13727.73	1037.58	7.51	77.56	67.26	67.26	5.05	2.46	67.37	23.01	1.32	98.00	99.70	72331.25	92328.69
	2008	73.93	64.29	9.64	13869.44	1145.80	7.33	77.98	25.69	67.05	4.91	2.41	67.87	23.16	1.35	98.00	99.90	71578.85	92320.27
	2009	74.27	64.55	9.71	12974.14	976.78	7.55	73.86	25.35	65.68	4.96	2.59	68.37	23.25	1.32	98.00	100.00	65812.14	92329.82
	2010	74.61	64.91	9.70	13092.23	1019.85	7.85	75.92	26.43	65.19	5.12	2.73	68.86	23.31	1.25	98.00	100.00	66250.77	92368.09
	2011	74.99	65.12	9.87	13347.58	1098.71	7.84	76.20	27.31	64.16	5.03	2.81	69.35	23.73	1.23	98.00	100.00	64385.00	92413.14
	2012	75.30	65.37	9.93	13196.15	991.27	7.74	77.89	28.48	63.43	4.91	2.83	69.83	24.12	1.34	98.00	100.00	62988.40	92458.18
	2013	75.56	65.58	9.99	13509.92	1021.07	7.53	78.48	27.33	65.17	4.91	2.62	70.31	24.52	1.35	98.00	100.00	-	92462.55
	2014	75.70	65.69	10.01	14119.07	1036.62	7.40	78.16	26.59	65.98	4.88	2.52	70.77	25.04	1.44	98.00	100.00	-	92463.04
	2015	75.75	65.73	10.02	14629.24	-	-	-	-	-	-	-	71.23	25.71	1.44	98.00	100.00	-	92475.56
	2016	75.75	65.82	9.92	14991.19	-	-	-	-	-	-	-	71.67	26.63	-	-	-	-	92507.93

	2017	76.81	65.78	11.03	-	-	-	-	-	-	-	-	-	-	-	-	-	-	-
Iceland	1995	78.49	68.36	10.13	30178.05	2156.82	8.21	100.01	16.07	83.93	6.89	1.32	91.63	17.48	2.08	98.80	100.00	20878.68	91560.02
	1996	79.00	68.81	10.19	31451.82	2225.12	8.16	100.01	16.75	83.25	6.80	1.37	91.80	17.58	2.12	98.80	100.00	21181.61	91621.32
	1997	79.17	68.96	10.21	32727.90	2217.90	7.91	100.00	17.94	82.06	6.49	1.42	91.96	17.66	2.04	98.80	100.00	21089.45	91705.81
	1998	79.42	69.17	10.26	34505.69	2718.16	8.73	93.22	18.11	80.58	7.04	1.70	92.12	17.74	2.05	98.80	100.00	3585.22	91820.03
	1999	79.63	69.34	10.30	35455.89	3055.93	9.44	93.24	16.42	82.39	7.78	1.66	92.27	17.80	1.99	98.80	100.00	3657.94	91919.36
	2000	79.89	69.54	10.35	36610.69	2966.40	9.28	93.72	17.48	81.35	7.54	1.73	92.40	17.81	2.08	98.80	100.00	4122.34	92011.01
	2001	80.20	69.79	10.41	37512.27	2616.10	9.12	93.41	17.58	81.18	7.41	1.72	92.53	17.84	1.95	98.80	100.00	3830.92	92074.76
	2002	80.47	70.01	10.47	37295.93	3150.77	9.86	92.93	16.79	81.93	8.07	1.78	92.66	17.84	1.93	98.80	100.00	3963.39	92147.49
	2003	80.73	70.21	10.52	37942.34	3945.30	10.07	92.86	16.99	81.71	8.23	1.84	92.79	17.83	1.99	98.80	100.00	3941.86	92195.01
	2004	80.97	70.40	10.57	40657.07	4521.68	9.59	92.93	17.38	81.30	7.79	1.79	92.91	17.84	2.04	98.80	100.00	4023.27	92240.38
	2005	81.19	70.58	10.62	42701.59	5254.40	9.21	92.58	17.19	81.43	7.50	1.71	93.04	17.89	2.05	98.80	100.00	3980.63	92312.25
	2006	81.35	70.71	10.64	43796.69	5094.19	8.96	92.01	16.58	81.98	7.35	1.62	93.16	17.78	2.08	98.80	100.00	4230.93	92349.21
	2007	81.58	70.90	10.68	46695.21	6057.86	8.75	91.59	82.55	82.55	7.23	1.53	93.28	17.71	2.09	98.80	100.00	4449.11	92359.35
	2008	81.78	71.06	10.72	46531.31	4892.20	8.80	91.54	15.90	82.63	7.27	1.53	93.40	17.68	2.15	98.80	100.00	5043.32	92322.37
	2009	81.90	71.15	10.75	43152.69	3671.31	9.12	91.97	16.55	82.01	7.48	1.64	93.51	17.73	2.23	98.80	100.00	5144.29	92262.66
	2010	82.03	71.23	10.80	41676.45	3690.42	8.86	92.71	18.21	80.36	7.12	1.74	93.62	17.87	2.20	98.80	100.00	5390.98	92253.80
	2011	82.15	71.34	10.81	42374.97	3985.00	8.65	92.53	17.98	80.57	6.97	1.68	93.73	18.31	2.02	98.80	100.00	5530.26	92274.20
	2012	82.23	71.41	10.82	42663.28	3856.83	8.68	92.48	17.99	80.55	6.99	1.69	93.84	18.83	2.04	98.80	100.00	5514.85	92306.16
	2013	82.25	71.44	10.81	44126.03	4182.44	8.75	92.38	17.80	80.73	7.07	1.69	93.94	19.44	1.93	98.80	100.00	-	92372.18
	2014	82.23	71.43	10.80	44477.94	4661.62	8.86	92.18	17.48	81.04	7.18	1.68	94.04	20.10	1.93	98.80	100.00	-	92465.85
	2015	82.24	71.45	10.79	45820.06	-	-	-	-	-	-	-	94.14	20.79	1.93	98.80	100.00	-	92541.28
	2016	82.27	71.48	10.79	48613.89	-	-	-	-	-	-	-	94.23	21.40	-	-	-	-	92598.66
	2017	82.75	71.26	11.49	-	-	-	-	-	-	-	-	-	-	-	-	-	-	-
India	1995	60.73	52.20	8.53	622.30	15.82	4.02	91.36	67.45	26.17	1.05	2.97	26.61	6.85	3.65	20.80	75.60	1651503.01	91063.61
	1996	61.03	52.46	8.56	656.70	15.94	3.90	91.03	67.51	25.83	1.01	2.89	26.82	6.95	3.58	21.70	76.60	1708836.97	91092.15
	1997	61.28	52.69	8.59	670.61	18.43	4.25	91.92	68.71	25.25	1.07	3.18	27.03	7.03	3.51	22.70	77.60	1761537.39	91127.30
	1998	61.61	52.97	8.64	699.07	18.48	4.30	91.84	68.23	25.70	1.10	3.19	27.24	7.10	3.45	23.70	78.60	1768965.00	91170.10
	1999	61.94	53.24	8.70	747.25	18.28	4.03	91.02	65.46	28.08	1.13	2.90	27.45	7.17	3.38	24.60	79.60	1956039.00	91214.14

	2000	62.29	53.54	8.75	762.31	19.61	4.26	91.81	67.86	26.09	1.11	3.15	27.67	7.23	3.31	25.60	80.60	1885189.00	91265.53
	2001	62.62	53.81	8.80	785.34	20.94	4.50	92.41	70.26	23.97	1.08	3.42	27.92	7.33	3.24	26.60	81.50	1875600.85	91286.94
	2002	63.00	54.14	8.86	801.51	21.06	4.40	91.92	70.50	23.31	1.03	3.38	28.24	7.43	3.18	27.60	82.50	1917294.66	91313.99
	2003	63.40	54.48	8.92	850.29	23.65	4.30	91.61	70.61	22.92	0.98	3.31	28.57	7.51	3.11	28.60	83.50	1976449.15	91345.73
	2004	63.77	54.79	8.98	902.91	26.80	4.22	89.55	67.85	24.23	1.02	3.20	28.90	7.58	3.04	29.60	84.50	2090334.85	91376.90
	2005	64.16	55.13	9.03	971.23	31.34	4.28	89.65	65.90	26.49	1.13	3.15	29.24	7.64	2.97	30.60	85.50	2117460.59	91415.84
	2006	64.55	55.50	9.05	1044.89	34.64	4.25	89.03	65.75	26.15	1.11	3.14	29.57	7.73	2.90	31.60	86.50	2232760.11	91406.07
	2007	64.93	55.83	9.11	1130.09	43.21	4.23	88.20	26.02	26.02	1.10	3.13	29.91	7.80	2.82	32.60	87.40	2382011.76	91400.94
	2008	65.32	56.18	9.14	1156.93	46.91	4.34	87.96	64.39	26.79	1.16	3.18	30.25	7.87	2.75	33.60	88.40	2438714.28	91407.93
	2009	65.73	56.55	9.19	1237.34	48.23	4.38	87.84	63.33	27.90	1.22	3.15	30.59	7.93	2.67	34.60	89.40	2594639.92	91420.71
	2010	66.12	56.92	9.20	1345.77	59.18	4.28	86.96	63.37	27.13	1.16	3.12	30.93	7.99	2.60	35.50	90.30	2771456.76	91446.37
	2011	66.51	57.21	9.30	1416.40	65.71	4.33	88.43	64.43	27.14	1.18	3.16	31.28	8.10	2.53	36.50	91.30	2828845.83	91472.45
	2012	66.91	57.54	9.37	1474.97	64.93	4.39	88.85	64.88	26.97	1.18	3.21	31.63	8.19	2.48	37.50	92.20	3002894.93	91510.88
	2013	67.28	57.84	9.44	1550.14	68.53	4.53	89.14	63.81	28.41	1.29	3.24	31.99	8.29	2.43	38.50	93.10	-	91562.27
	2014	67.72	58.19	9.53	1646.78	74.99	4.69	89.21	62.42	30.04	1.41	3.28	32.37	8.42	2.38	39.50	94.10	-	91621.37
	2015	68.10	58.48	9.61	1758.04	-	-	-	-	-	-	-	32.75	8.58	2.35	39.60	94.10	-	91695.05
	2016	68.55	58.89	9.66	1861.49	-	-	-	-	-	-	-	33.14	8.80	-	-	-	-	91768.37
	2017	68.97	59.37	9.60	-	-	-	-	-	-	-	-	-	-	-	-	-	-	-
Indonesia	1995	65.74	58.02	7.72	2219.81	20.08	1.96	72.94	46.51	36.24	0.71	1.25	36.08	6.68	2.69	41.00	73.70	1315570.67	88315.16
	1996	66.15	58.39	7.76	2357.96	22.59	1.99	73.13	46.37	36.58	0.73	1.26	37.24	6.81	2.63	42.30	74.50	1314060.75	88353.72
	1997	66.48	58.67	7.81	2433.34	20.48	1.93	72.99	47.33	35.16	0.68	1.25	38.41	6.93	2.58	43.50	75.40	5040841.30	88402.05
	1998	66.77	58.91	7.85	2084.23	9.53	2.06	74.33	48.72	34.45	0.71	1.35	39.59	7.05	2.54	44.70	76.30	1191187.16	88456.07
	1999	66.98	59.10	7.88	2071.55	14.89	2.22	75.56	49.24	34.83	0.77	1.45	40.79	7.17	2.52	45.90	77.10	668887.60	88513.45
	2000	67.24	59.31	7.94	2143.39	15.43	1.98	72.66	46.04	36.63	0.72	1.25	42.00	7.29	2.51	47.10	77.90	622464.65	88578.88
	2001	67.54	59.58	7.96	2190.77	16.70	2.23	73.56	41.80	43.17	0.96	1.27	42.78	7.36	2.51	48.10	78.60	849945.65	88614.10
	2002	67.79	59.81	7.98	2257.75	20.42	2.27	72.17	44.04	38.98	0.88	1.38	43.57	7.41	2.51	49.20	79.30	1720445.17	88652.90
	2003	68.04	60.03	8.02	2333.10	27.00	2.53	74.11	44.41	40.07	1.02	1.52	44.35	7.42	2.51	50.20	80.00	888646.15	88698.43
	2004	66.48	58.71	7.77	2416.84	27.24	2.37	73.88	44.71	39.48	0.93	1.43	45.15	7.40	2.52	51.20	80.60	1060213.55	88751.04
	2005	68.54	60.44	8.10	2519.51	35.25	2.79	76.70	54.62	28.79	0.80	1.99	45.94	7.35	2.51	52.10	81.30	1171042.59	88814.66

	2006	68.79	60.65	8.14	2621.96	46.20	2.91	76.32	52.34	31.41	0.91	1.99	46.73	7.40	2.51	53.10	81.90	2381293.72	88883.57
	2007	69.06	60.86	8.20	2750.62	57.65	3.10	77.16	36.35	36.35	1.13	1.97	47.53	7.42	2.51	54.10	82.60	772946.22	88946.92
	2008	69.32	61.06	8.26	2876.89	60.83	2.81	76.47	49.06	35.85	1.01	1.80	48.33	7.42	2.50	55.10	83.20	796581.92	89007.50
	2009	69.58	61.26	8.31	2970.04	63.93	2.83	76.67	49.00	36.08	1.02	1.81	49.12	7.39	2.49	56.00	83.80	1148285.68	89074.12
	2010	69.87	61.51	8.36	3113.48	85.65	2.74	75.76	47.21	37.69	1.03	1.71	49.92	7.33	2.48	57.00	84.50	744790.02	89153.27
	2011	70.19	61.77	8.42	3262.75	98.97	2.71	76.35	47.39	37.92	1.03	1.68	50.71	7.38	2.47	57.90	85.10	763553.16	89142.22
	2012	70.51	62.03	8.48	3415.35	107.24	2.90	75.09	45.35	39.61	1.15	1.75	51.49	7.42	2.46	58.80	85.70	780550.76	89167.35
	2013	70.81	62.27	8.54	3560.11	106.02	2.93	75.33	45.63	39.43	1.15	1.77	52.25	7.47	2.44	59.70	86.20	-	89216.23
	2014	71.09	62.49	8.60	3692.94	99.41	2.85	75.32	46.87	37.78	1.08	1.77	53.00	7.52	2.41	60.60	86.80	-	89287.52
	2015	71.38	62.71	8.67	3827.55	-	-	-	-	-	-	-	53.74	7.60	2.39	60.80	87.40	-	89374.43
	2016	71.66	62.95	8.71	3974.06	-	-	-	-	-	-	-	54.47	7.75	-	-	-	-	89460.94
	2017	71.48	62.65	8.82	-	-	-	-	-	-	-	-	-	-	-	-	-	-	-
Iraq	1995	65.24	55.28	9.97	2322.87	-	-	-	-	-	-	-	68.78	6.89	5.42	71.50	78.20	107285.11	89420.99
	1996	65.23	55.35	9.88	2500.01	-	-	-	-	-	-	-	68.59	6.79	5.33	72.20	78.60	98175.48	89391.76
	1997	64.95	55.18	9.77	2937.47	-	-	-	-	-	-	-	68.41	6.71	5.22	73.00	78.90	103253.91	89378.25
	1998	65.70	55.80	9.90	3839.83	-	-	-	-	-	-	-	68.39	6.63	5.11	73.70	79.40	106026.11	89359.32
	1999	65.98	56.05	9.93	4379.20	-	-	-	-	-	-	-	68.44	6.55	5.00	74.50	79.90	110878.99	89343.79
	2000	65.96	56.06	9.90	4311.28	-	-	-	-	-	-	-	68.50	6.47	4.89	75.30	80.40	124402.47	89350.75
	2001	66.54	56.55	9.99	4285.86	-	-	-	-	-	-	-	68.55	6.42	4.80	76.20	80.80	132843.68	89351.09
	2002	66.35	56.44	9.91	3880.11	-	-	-	-	-	-	-	68.60	6.36	4.71	76.90	81.30	128686.16	89367.63
	2003	64.96	55.35	9.61	2526.04	16.41	4.36	100.00	54.64	45.36	1.98	2.38	68.66	6.29	4.64	77.70	81.80	107790.88	89381.87
	2004	65.19	55.55	9.64	3792.14	55.83	5.61	100.00	27.83	72.17	4.05	1.56	68.71	6.23	4.59	78.50	82.30	125423.58	89372.73
	2005	64.74	55.28	9.46	3857.61	55.49	4.13	100.00	32.65	67.35	2.78	1.35	68.77	6.18	4.56	79.30	82.80	124611.70	89336.21
	2006	63.77	54.41	9.36	4143.69	59.33	3.00	100.00	36.19	63.81	1.91	1.09	68.82	6.16	4.55	80.10	83.20	129502.48	89264.90
	2007	64.22	54.74	9.48	4098.31	97.30	3.69	100.00	69.49	69.49	2.57	1.13	68.87	6.15	4.55	80.90	83.70	130078.49	89165.49
	2008	66.12	56.24	9.89	4325.67	146.59	3.93	100.00	25.16	74.84	2.94	0.99	68.93	6.13	4.55	81.70	84.20	137871.12	89025.27
	2009	66.83	56.78	10.05	4354.68	146.76	4.65	100.00	25.74	74.26	3.45	1.20	68.98	6.08	4.56	82.50	84.70	138423.77	88882.29
	2010	67.20	57.08	10.12	4502.75	144.98	3.82	100.00	26.10	73.90	2.82	1.00	69.03	5.98	4.56	83.30	85.10	146903.73	88765.21
	2011	67.54	57.33	10.21	4695.36	164.05	3.32	100.00	24.93	75.07	2.49	0.83	69.10	5.86	4.56	84.00	85.60	151457.06	88611.94

	2012	67.82	57.56	10.27	5178.42	294.16	5.26	100.00	37.69	62.31	3.28	1.98	69.18	5.71	4.54	84.80	86.10	155529.51	88494.01
	2013	67.68	57.44	10.24	5338.52	339.35	5.92	100.00	37.17	62.83	3.72	2.20	69.26	5.57	4.52	85.60	86.50	-	88422.71
	2014	67.01	56.90	10.11	5203.44	292.00	5.54	100.00	39.73	60.27	3.34	2.20	69.36	5.48	4.48	85.60	86.60	-	88398.31
	2015	67.52	57.34	10.18	5285.67	-	-	-	-	-	-	-	69.47	5.46	4.43	85.60	86.60	-	88391.28
	2016	67.60	57.43	10.17	5695.68	-	-	-	-	-	-	-	69.59	5.53	-	-	-	-	88417.53
	2017	76.73	64.42	12.31	-	-	-	-	-	-	-	-	-	-	-	-	-	-	-
Ireland	1995	75.75	66.18	9.57	28961.60	1239.14	6.44	55.83	15.34	72.52	4.67	1.77	57.92	16.71	1.84	89.30	96.40	68374.02	90149.71
	1996	75.95	66.35	9.59	30977.21	1330.36	6.35	54.56	15.53	71.53	4.54	1.81	58.14	16.41	1.88	89.30	96.40	70151.95	90284.30
	1997	76.02	66.43	9.60	33823.85	1404.67	6.20	60.93	15.27	74.95	4.64	1.55	58.40	16.13	1.93	89.30	96.30	71594.31	90376.84
	1998	76.17	66.55	9.62	36318.14	1467.94	6.02	56.88	14.40	74.68	4.49	1.52	58.65	15.89	1.94	89.30	96.30	65387.42	90451.23
	1999	76.32	66.68	9.64	39724.03	1567.82	5.93	60.63	15.00	75.25	4.46	1.47	58.90	15.70	1.90	89.20	96.30	66633.59	90525.10
	2000	76.62	66.90	9.72	42945.26	1593.34	6.03	60.76	15.74	74.10	4.47	1.56	59.15	15.56	1.89	89.20	96.30	68143.29	90618.80
	2001	77.12	67.30	9.82	44719.92	1878.20	6.60	58.59	14.97	74.44	4.91	1.69	59.40	15.44	1.94	89.30	96.40	70154.00	90623.48
	2002	77.63	67.70	9.93	46746.99	2248.68	6.85	58.15	14.27	75.46	5.17	1.68	59.65	15.37	1.97	89.40	96.60	69094.69	90613.66
	2003	78.12	68.08	10.04	47426.39	2925.20	7.05	65.58	15.31	76.66	5.40	1.65	59.93	15.34	1.96	89.50	96.70	68872.54	90604.77
	2004	78.52	68.39	10.13	49678.42	3532.97	7.34	63.26	15.02	76.26	5.60	1.74	60.20	15.35	1.93	89.60	96.80	68770.04	90615.49
	2005	78.93	68.70	10.23	51527.34	3736.53	7.27	66.76	16.04	75.97	5.52	1.75	60.48	15.39	1.86	89.70	96.90	70080.71	90620.37
	2006	79.27	68.97	10.30	52926.19	3982.27	7.22	65.19	16.06	75.36	5.44	1.78	60.75	15.53	1.91	89.80	97.00	71503.35	90582.17
	2007	79.59	69.22	10.36	54096.27	4704.24	7.57	60.98	75.69	75.69	5.73	1.84	61.03	15.68	2.01	89.90	97.10	70033.03	90508.14
	2008	79.77	69.36	10.40	50918.35	5326.22	8.64	62.25	15.33	75.37	6.51	2.13	61.30	15.86	2.06	90.00	97.20	70132.72	90436.52
	2009	79.95	69.51	10.44	48071.73	4961.58	9.53	58.79	16.12	72.58	6.92	2.61	61.57	16.11	2.06	90.00	97.30	65613.07	90353.10
	2010	80.40	69.84	10.56	48671.89	4238.22	8.76	60.04	18.22	69.64	6.10	2.66	61.84	16.43	2.05	90.10	97.40	65591.25	90294.98
	2011	80.55	69.97	10.58	49942.51	4309.72	8.15	54.90	17.65	67.84	5.53	2.62	62.11	17.10	2.03	90.20	97.50	61268.10	90261.33
	2012	80.78	70.15	10.63	49851.02	4079.48	8.32	52.09	16.89	67.57	5.62	2.70	62.39	17.85	2.00	90.30	97.70	62433.01	90294.52
	2013	80.98	70.30	10.68	50542.41	4156.59	8.01	52.06	17.40	66.59	5.33	2.68	62.67	18.65	1.96	90.40	97.80	-	90372.49
	2014	81.10	70.40	10.70	54527.29	4239.15	7.78	52.04	17.66	66.06	5.14	2.64	62.95	19.49	1.94	90.50	97.90	-	90464.16
	2015	81.12	70.43	10.70	67590.37	-	-	-	-	-	-	-	63.24	20.34	1.94	90.50	97.90	-	90580.58
	2016	81.14	70.45	10.70	69632.27	-	-	-	-	-	-	-	63.54	20.98	-	-	-	-	90701.69
	2017	81.85	70.35	11.49	-	-	-	-	-	-	-	-	-	-	-	-	-	-	-

Iran	1995	70.72	60.36	10.35	4298.12	67.59	3.74	97.01	53.59	44.76	1.68	2.07	60.24	6.97	3.22	73.80	93.00	370387.94	88753.33
	1996	70.76	60.43	10.33	4496.29	85.52	3.64	97.22	51.13	47.41	1.73	1.91	61.09	6.98	2.96	74.80	93.30	385888.41	89090.06
	1997	70.93	60.58	10.34	4475.08	109.23	4.00	96.52	49.67	48.54	1.94	2.06	61.85	6.98	2.73	75.90	93.50	402922.92	89416.94
	1998	71.15	60.79	10.36	4483.09	133.21	4.40	96.93	49.88	48.54	2.14	2.27	62.59	6.96	2.53	76.90	93.70	410530.57	89731.80
	1999	71.39	61.00	10.39	4491.20	176.52	4.39	96.22	55.42	42.40	1.86	2.53	63.32	6.94	2.36	77.90	93.90	425640.49	90008.88
	2000	71.44	61.10	10.34	4676.87	229.50	4.47	96.23	56.16	41.64	1.86	2.61	64.04	6.91	2.21	78.90	94.10	448089.45	90251.04
	2001	71.47	61.14	10.33	4719.91	291.68	5.02	95.38	53.71	43.69	2.19	2.83	64.76	6.96	2.09	79.90	94.30	460783.88	90359.94
	2002	71.56	61.26	10.30	5034.67	107.59	5.39	90.09	53.49	40.62	2.19	3.20	65.47	7.01	2.00	80.80	94.50	483378.51	90457.49
	2003	70.11	60.13	9.98	5403.71	110.83	5.42	87.83	49.75	43.36	2.35	3.07	66.17	7.05	1.93	81.80	94.70	516232.69	90529.30
	2004	71.94	61.64	10.30	5572.91	144.96	5.89	89.19	55.27	38.03	2.24	3.65	66.87	7.10	1.87	82.80	94.90	552318.80	90597.63
	2005	72.38	62.04	10.34	5684.96	177.77	6.06	89.22	54.89	38.48	2.33	3.73	67.56	7.13	1.83	83.80	95.10	576650.65	90656.59
	2006	72.85	62.41	10.44	5901.64	204.26	5.95	88.54	50.30	43.20	2.57	3.38	68.24	7.07	1.81	84.80	95.20	619914.47	90668.99
	2007	73.29	62.70	10.59	6311.79	254.35	5.84	88.32	41.48	41.48	2.42	3.42	68.86	7.01	1.79	85.70	95.40	650956.81	90679.46
	2008	73.68	62.99	10.69	6256.88	314.55	6.28	86.67	53.04	38.80	2.44	3.85	69.46	6.96	1.78	86.70	95.50	-	90681.60
	2009	74.03	63.25	10.78	6247.69	380.76	7.63	87.23	54.32	37.73	2.88	4.75	70.05	6.91	1.77	87.60	95.60	-	90680.29
	2010	74.36	63.55	10.81	6531.93	455.45	8.02	86.79	57.74	33.48	2.68	5.33	70.63	6.89	1.77	88.60	95.80	-	90715.26
	2011	74.70	63.76	10.95	6622.67	545.23	7.12	85.24	55.58	34.80	2.48	4.65	71.20	6.86	1.76	89.30	95.90	-	90691.53
	2012	74.81	63.86	10.95	6052.52	510.59	6.98	81.30	50.01	38.48	2.69	4.30	71.77	6.84	1.74	89.60	96.00	-	90707.86
	2013	74.96	63.98	10.98	5964.18	414.70	6.49	81.30	47.06	42.11	2.73	3.76	72.32	6.86	1.73	89.90	96.10	-	90738.42
	2014	75.22	64.18	11.04	6161.10	350.74	6.89	81.30	47.80	41.20	2.84	4.05	72.86	6.92	1.71	89.90	96.20	-	90806.52
	2015	75.69	64.52	11.18	6007.00	-	-	-	-	-	-	-	73.38	7.06	1.69	90.00	96.20	-	90889.13
	2016	75.94	64.79	11.15	6733.91	-	-	-	-	-	-	-	73.88	7.33	-	-	-	-	90994.53
	2017	77.35	65.74	11.61	-	-	-	-	-	-	-	-	-	-	-	-	-	-	-
Israel	1995	77.02	67.34	9.68	24592.25	1315.72	7.35	85.85	25.73	67.39	4.95	2.20	90.87	15.91	2.90	100.00	100.00	53255.20	88368.86
	1996	77.36	67.60	9.76	25230.31	1421.06	7.40	84.23	23.89	69.02	5.11	2.10	90.94	15.94	2.90	100.00	100.00	56520.49	88472.00
	1997	77.67	67.84	9.82	25508.75	1470.44	7.56	83.23	24.76	68.05	5.14	2.25	91.01	15.98	2.94	100.00	100.00	58641.05	88561.79
	1998	78.00	68.11	9.89	25970.83	1429.04	7.42	83.45	28.01	64.18	4.76	2.49	91.07	16.03	2.98	100.00	100.00	59555.16	88646.55
	1999	78.29	68.33	9.96	26233.31	1359.28	7.16	80.36	26.57	64.25	4.60	2.37	91.14	16.10	2.97	100.00	100.00	60998.69	88723.56
	2000	78.52	68.52	10.00	27636.34	1490.05	7.13	80.48	28.33	62.56	4.46	2.51	91.20	16.18	2.95	100.00	100.00	65619.39	88826.61

	2001	78.64	68.60	10.04	26999.37	1531.73	7.61	80.56	29.06	62.01	4.72	2.74	91.27	16.17	2.92	100.00	100.00	65879.06	88820.59
	2002	78.58	68.55	10.03	26503.84	1366.91	7.49	77.54	26.84	63.24	4.74	2.59	91.33	16.16	2.89	100.00	100.00	69487.80	88797.63
	2003	79.07	68.96	10.11	26229.40	1394.67	7.44	75.95	27.62	61.69	4.59	2.70	91.39	16.15	2.95	100.00	100.00	71533.78	88787.05
	2004	79.38	69.20	10.18	26947.44	1445.93	7.35	75.23	27.77	60.90	4.47	2.71	91.46	16.14	2.90	100.00	100.00	70576.27	88795.87
	2005	79.75	69.51	10.24	27570.95	1515.13	7.44	75.51	29.25	59.29	4.41	2.88	91.52	16.13	2.84	100.00	100.00	71353.03	88813.38
	2006	79.85	69.58	10.27	28499.33	1592.65	7.38	71.76	26.03	63.72	4.70	2.68	91.58	16.15	2.88	100.00	100.00	73591.54	88848.03
	2007	80.27	69.90	10.37	29614.08	1813.20	7.37	70.89	61.89	61.89	4.56	2.81	91.64	16.18	2.90	100.00	100.00	77128.57	88881.50
	2008	80.59	70.13	10.46	29962.01	2145.39	7.33	68.96	26.03	61.00	4.47	2.77	91.70	16.26	2.96	100.00	100.00	75238.55	88929.12
	2009	80.93	70.38	10.54	29658.52	2057.35	7.46	68.94	26.13	61.15	4.56	2.83	91.76	16.45	2.96	100.00	100.00	75026.04	88975.41
	2010	81.49	70.84	10.65	30642.94	2247.65	7.36	67.31	24.39	62.69	4.61	2.67	91.82	16.77	3.03	100.00	100.00	79072.44	89007.36
	2011	81.70	71.01	10.70	31483.00	2460.50	7.39	67.03	24.52	62.46	4.62	2.71	91.89	16.98	2.98	100.00	100.00	81703.91	89000.12
	2012	81.96	71.22	10.74	31507.48	2514.57	7.73	67.56	25.06	62.91	4.87	2.87	91.95	17.26	3.05	100.00	100.00	84044.43	88983.52
	2013	82.07	71.32	10.75	32196.36	2853.69	7.89	68.92	26.51	61.54	4.86	3.04	92.01	17.62	3.03	100.00	100.00	-	88979.50
	2014	81.40	70.78	10.62	32661.29	2910.29	7.81	68.93	26.98	60.85	4.75	3.06	92.08	18.01	3.08	100.00	100.00	-	88994.93
	2015	82.08	71.36	10.72	32993.31	-	-	-	-	-	-	-	92.14	18.42	3.09	100.00	100.00	-	89004.29
	2016	82.14	71.44	10.70	33673.13	-	-	-	-	-	-	-	92.21	18.92	-	-	-	-	89031.40
	2017	82.97	71.38	11.59	-	-	-	-	-	-	-	-	-	-	-	-	-	-	-
Italy	1995	78.12	68.15	9.97	32829.88	1461.94	7.10	91.87	26.86	70.76	5.02	2.07	66.92	24.20	1.19	99.50	100.00	518470.76	92558.81
	1996	78.33	68.37	9.97	33242.85	1657.17	7.19	91.33	26.78	70.68	5.08	2.11	66.98	24.73	1.20	99.50	100.00	507338.86	92546.47
	1997	78.69	68.69	10.01	33835.07	1628.35	7.47	91.69	26.82	70.75	5.29	2.19	67.04	25.24	1.21	99.50	100.00	508373.86	92539.74
	1998	78.97	68.94	10.03	34371.98	1681.17	7.55	91.87	27.39	70.19	5.30	2.25	67.10	25.75	1.21	99.50	100.00	519046.02	92541.12
	1999	79.27	69.21	10.06	34902.26	1674.54	7.63	91.74	27.00	70.58	5.38	2.25	67.16	26.29	1.23	99.50	100.00	521472.01	92535.13
	2000	79.60	69.48	10.13	36180.78	1587.96	7.91	90.78	25.34	72.09	5.70	2.21	67.22	26.88	1.26	99.50	100.00	547349.86	92558.76
	2001	79.93	69.75	10.18	36801.29	1656.22	8.11	88.82	23.43	73.62	5.97	2.14	67.28	27.40	1.25	99.50	100.00	543360.17	92571.38
	2002	80.17	69.96	10.21	36837.86	1829.93	8.23	88.41	23.13	73.84	6.08	2.15	67.38	27.95	1.27	99.50	100.00	550782.07	92592.64
	2003	80.27	70.07	10.21	36729.98	2246.11	8.17	88.63	22.79	74.28	6.07	2.10	67.50	28.51	1.29	99.50	100.00	564951.18	92613.15
	2004	80.72	70.42	10.30	37070.33	2658.15	8.49	89.86	22.00	75.52	6.41	2.08	67.62	29.01	1.34	99.50	100.00	562794.85	92625.26
	2005	80.91	70.58	10.34	37238.94	2788.52	8.71	87.53	20.74	76.31	6.64	2.06	67.74	29.45	1.34	99.50	100.00	564114.95	92649.30
	2006	81.09	70.73	10.36	37872.17	2951.59	8.82	86.87	20.44	76.47	6.74	2.07	67.86	29.86	1.37	99.50	100.00	563324.94	92668.46

	2007	81.27	70.88	10.39	38236.80	3210.65	8.48	88.05	76.49	76.49	6.49	1.99	67.97	30.18	1.40	99.50	100.00	547383.14	92692.09
	2008	81.46	71.04	10.42	37585.34	3623.85	8.89	89.12	20.55	76.94	6.84	2.05	68.09	30.47	1.45	99.50	100.00	529283.80	92718.07
	2009	81.57	71.13	10.44	35363.40	3488.47	9.41	85.58	19.69	76.99	7.25	2.17	68.21	30.82	1.45	99.50	100.00	480698.22	92755.71
	2010	81.78	71.30	10.48	35849.37	3384.45	9.42	84.99	19.52	77.04	7.26	2.16	68.33	31.30	1.46	99.50	100.00	489460.13	92816.71
	2011	81.92	71.42	10.50	35994.13	3559.50	9.27	84.32	20.96	75.14	6.97	2.31	68.44	31.91	1.44	99.50	100.00	482619.04	92880.64
	2012	82.02	71.51	10.51	34885.30	3242.22	9.28	84.80	20.91	75.34	6.99	2.29	68.56	32.63	1.43	99.50	100.00	482634.00	92975.81
	2013	82.24	71.67	10.56	33887.30	3294.85	9.22	84.58	20.64	75.59	6.97	2.25	68.69	33.43	1.39	99.50	100.00	-	93091.82
	2014	82.29	71.72	10.57	33615.97	3257.75	9.25	86.88	21.19	75.61	6.99	2.26	68.82	34.24	1.37	99.50	100.00	-	93220.84
	2015	82.33	71.75	10.58	33984.09	-	-	-	-	-	-	-	68.96	34.99	1.37	99.50	100.00	-	93351.89
	2016	82.34	71.75	10.59	34377.93	-	-	-	-	-	-	-	69.12	35.66	-	-	-	-	93483.21
	2017	83.17	71.87	11.30	-	-	-	-	-	-	-	-	-	-	-	-	-	-	-
Jamaica	1995	74.19	64.84	9.36	4946.87	97.24	4.16	61.92	28.85	53.42	2.22	1.94	50.62	12.71	2.77	80.30	93.30	10886.33	90616.48
	1996	74.07	64.74	9.33	4893.89	122.89	4.73	60.58	23.96	60.45	2.86	1.87	50.86	12.76	2.74	80.30	93.30	11247.79	90663.83
	1997	73.94	64.63	9.31	4791.63	175.98	5.97	69.38	30.40	56.19	3.35	2.62	51.10	12.82	2.70	80.40	93.40	11457.93	90722.20
	1998	73.91	64.59	9.32	4635.79	187.37	5.48	66.59	25.50	61.71	3.38	2.10	51.34	12.91	2.67	80.50	93.40	11874.58	90773.98
	1999	73.89	64.54	9.34	4642.55	172.57	5.04	69.47	32.11	53.78	2.71	2.33	51.57	13.02	2.62	80.60	93.40	12168.26	90836.49
	2000	73.82	64.48	9.34	4644.60	199.94	5.81	65.01	28.97	55.44	3.22	2.59	51.81	13.13	2.58	80.70	93.50	12364.43	90905.18
	2001	73.64	64.33	9.30	4671.64	185.61	5.34	69.27	37.48	45.89	2.45	2.89	52.05	13.11	2.53	80.80	93.50	12249.86	90990.16
	2002	73.92	64.56	9.36	4732.36	179.12	4.86	61.81	26.33	57.40	2.79	2.07	52.25	13.07	2.48	80.90	93.50	12392.12	91078.97
	2003	74.12	64.72	9.40	4875.00	162.22	4.56	64.73	32.01	50.55	2.30	2.25	52.44	13.05	2.44	81.00	93.60	12959.04	91172.66
	2004	74.33	64.87	9.46	4910.08	183.47	4.80	63.64	27.56	56.69	2.72	2.08	52.63	13.04	2.40	81.00	93.60	13419.47	91276.30
	2005	74.66	65.12	9.55	4925.28	170.75	4.07	63.64	32.58	48.81	1.99	2.08	52.81	13.06	2.36	81.10	93.60	13314.89	91366.37
	2006	74.83	65.29	9.53	5039.41	187.07	4.22	63.73	28.85	54.73	2.31	1.91	53.00	13.05	2.33	81.20	93.60	14549.96	91449.10
	2007	74.73	65.22	9.50	5083.60	230.49	4.87	70.95	52.03	52.03	2.53	2.34	53.18	13.05	2.29	81.30	93.70	15423.55	91524.91
	2008	74.65	65.17	9.48	5015.85	272.62	5.40	70.95	32.96	53.54	2.89	2.51	53.37	13.05	2.25	81.40	93.70	13912.56	91590.92
	2009	74.59	65.13	9.46	4774.45	229.13	5.16	70.90	31.12	56.10	2.89	2.26	53.56	13.05	2.21	81.50	93.70	13884.10	91677.66
	2010	74.65	65.17	9.48	4682.52	255.66	5.30	70.95	30.90	56.45	2.99	2.31	53.74	13.09	2.17	81.60	93.70	14581.68	91744.36
	2011	74.75	65.24	9.51	4742.92	273.31	5.21	70.98	32.91	53.64	2.80	2.42	53.93	13.18	2.14	81.60	93.80	15053.51	91815.80
	2012	74.80	65.27	9.53	4694.25	302.92	5.66	59.49	25.06	57.87	3.27	2.38	54.13	13.30	2.10	81.70	93.80	15474.32	91890.06

	2013	74.84	65.30	9.54	4699.77	305.85	5.91	58.41	24.98	57.23	3.38	2.53	54.34	13.45	2.07	81.80	93.80	-	91956.45
	2014	74.87	65.32	9.55	4714.86	266.19	5.36	58.41	27.81	52.38	2.81	2.55	54.56	13.62	2.05	81.80	93.80	-	92028.81
	2015	74.89	65.33	9.56	4740.64	-	-	-	-	-	-	-	54.79	13.84	2.03	81.80	93.80	-	92100.26
	2016	74.90	65.34	9.55	4790.04	-	-	-	-	-	-	-	55.03	14.06	-	-	-	-	92176.20
	2017	74.66	65.60	9.06	-	-	-	-	-	-	-	-	-	-	-	-	-	-	-
Japan	1995	79.75	70.03	9.72	40368.71	2844.85	6.62	79.32	14.03	82.32	5.45	1.17	78.02	20.58	1.42	100.00	100.00	1421640.91	90189.27
	1996	80.17	70.38	9.79	41514.86	2455.15	6.49	79.32	14.03	82.32	5.34	1.15	78.15	21.41	1.43	100.00	100.00	1424112.70	90289.81
	1997	80.39	70.55	9.83	41861.91	2333.37	6.73	81.23	15.17	81.33	5.47	1.26	78.27	22.26	1.39	100.00	100.00	1414397.05	90397.96
	1998	80.55	70.68	9.87	41277.08	2195.84	7.01	80.84	15.64	80.65	5.65	1.36	78.40	23.13	1.38	100.00	100.00	1371363.01	90514.91
	1999	80.72	70.81	9.90	41097.96	2597.76	7.34	81.19	15.84	80.49	5.91	1.43	78.52	24.00	1.34	100.00	100.00	1399008.94	90631.57
	2000	81.03	71.04	9.99	42169.70	2838.60	7.53	81.33	15.61	80.81	6.09	1.45	78.65	24.89	1.36	100.00	100.00	1406194.59	90754.25
	2001	81.31	71.26	10.06	42239.11	2531.11	7.75	82.93	15.51	81.29	6.30	1.45	79.99	25.78	1.33	100.00	100.00	1387952.21	90866.50
	2002	81.56	71.44	10.12	42190.78	2450.73	7.85	83.02	15.73	81.05	6.36	1.49	81.65	26.67	1.32	100.00	100.00	1422453.19	90980.48
	2003	81.75	71.57	10.17	42743.99	2694.43	8.00	84.28	16.61	80.30	6.42	1.58	83.20	27.58	1.29	100.00	100.00	1428102.13	91099.81
	2004	81.93	71.71	10.22	43671.68	2927.43	8.03	84.10	16.35	80.56	6.47	1.56	84.64	28.54	1.29	100.00	100.00	1427399.20	91225.30
	2005	82.05	71.79	10.26	44393.66	2927.57	8.18	83.23	15.50	81.37	6.66	1.52	85.98	29.55	1.26	100.00	100.00	1439905.16	91350.08
	2006	82.24	71.93	10.32	44995.52	2791.22	8.19	83.45	16.27	80.50	6.60	1.60	87.06	30.51	1.32	100.00	100.00	1425898.63	91418.07
	2007	82.41	72.05	10.37	45687.35	2806.57	8.25	82.74	81.41	81.41	6.72	1.53	88.01	31.50	1.34	100.00	100.00	1461356.12	91482.70
	2008	82.57	72.15	10.42	45165.89	3255.56	8.60	80.93	15.10	81.35	7.00	1.60	88.91	32.55	1.37	100.00	100.00	1385164.84	91555.68
	2009	82.77	72.29	10.48	42724.53	3741.59	9.51	81.04	15.00	81.50	7.75	1.76	89.74	33.72	1.37	100.00	100.00	1290243.65	91631.11
	2010	82.91	72.37	10.54	44507.68	4110.46	9.58	80.62	14.45	82.08	7.86	1.72	90.52	35.07	1.39	100.00	100.00	1350427.91	91715.55
	2011	82.66	72.16	10.50	44538.71	4654.23	10.07	80.19	13.96	82.59	8.32	1.75	91.25	36.45	1.39	100.00	100.00	1396767.26	91781.13
	2012	83.23	72.63	10.59	45276.83	4748.92	10.17	80.22	13.88	82.70	8.41	1.76	91.90	37.97	1.41	100.00	100.00	1478858.88	91853.62
	2013	83.44	72.80	10.64	46249.26	3960.20	10.25	82.77	13.89	83.22	8.53	1.72	92.49	39.58	1.43	100.00	100.00	-	91934.97
	2014	83.69	72.98	10.71	46466.12	3702.95	10.23	84.77	13.91	83.59	8.55	1.68	93.02	41.17	1.42	100.00	100.00	-	92020.33
	2015	83.83	73.08	10.74	47082.69	-	-	-	-	-	-	-	93.50	42.65	1.46	100.00	100.00	-	92120.09
	2016	83.94	73.16	10.78	47623.27	-	-	-	-	-	-	-	93.93	43.91	-	-	-	-	92230.46
	2017	84.19	73.07	11.12	-	-	-	-	-	-	-	-	-	-	-	-	-	-	-
Jordan	1995	72.65	61.94	10.71	2678.07	131.96	8.47	64.26	23.82	62.93	5.33	3.14	78.37	5.19	4.61	97.50	96.70	15834.06	88511.43

	1996	72.64	61.98	10.66	2650.81	138.76	8.91	66.86	26.03	61.07	5.44	3.47	78.66	5.22	4.47	97.60	96.70	16405.74	88478.55
	1997	72.76	62.10	10.67	2672.82	142.99	8.97	68.55	29.45	57.04	5.12	3.85	78.95	5.25	4.35	97.60	96.70	16830.79	88490.89
	1998	72.87	62.21	10.66	2699.41	159.37	9.31	69.13	29.76	56.95	5.30	4.01	79.24	5.27	4.23	97.70	96.70	17159.42	88528.56
	1999	72.97	62.31	10.66	2743.03	162.58	9.36	72.42	33.80	53.33	4.99	4.37	79.53	5.30	4.13	97.80	96.80	17135.08	88587.29
	2000	73.05	62.41	10.64	2810.04	171.34	9.65	74.94	38.96	48.01	4.64	5.02	79.81	5.33	4.03	97.80	96.80	17799.02	88685.45
	2001	73.13	62.49	10.64	2906.62	183.25	9.90	74.31	38.39	48.33	4.79	5.12	80.09	5.41	3.95	97.90	96.80	17657.35	88752.89
	2002	73.16	62.54	10.61	3020.09	187.84	9.68	78.25	39.37	49.69	4.81	4.87	80.36	5.49	3.88	98.00	96.80	18834.63	88821.51
	2003	73.06	62.51	10.55	3082.05	185.10	9.15	81.50	41.87	48.62	4.45	4.70	80.64	5.57	3.83	98.00	96.80	18728.01	88882.79
	2004	72.97	62.48	10.49	3262.19	199.75	9.05	84.06	40.49	51.83	4.69	4.36	80.91	5.65	3.78	98.10	96.80	20882.01	88952.01
	2005	73.11	62.60	10.50	3417.73	209.68	8.88	86.27	40.03	53.60	4.76	4.12	81.18	5.73	3.75	98.20	96.80	22339.73	89011.31
	2006	73.77	63.12	10.65	3557.30	220.13	8.08	88.50	39.35	55.53	4.49	3.60	81.44	5.82	3.73	98.20	96.90	22823.08	89104.27
	2007	74.39	63.54	10.84	3687.23	247.94	8.35	89.40	59.84	59.84	4.99	3.35	81.70	5.91	3.71	98.30	96.90	23900.58	89192.97
	2008	74.82	63.87	10.95	3773.19	320.87	8.78	83.58	31.86	61.88	5.43	3.35	81.96	6.00	3.69	98.30	96.90	24469.12	89277.14
	2009	75.29	64.21	11.08	3786.53	362.73	9.54	75.85	22.63	70.17	6.70	2.85	82.22	6.09	3.68	98.40	96.90	24315.65	89346.80
	2010	75.75	64.57	11.17	3679.19	341.27	8.42	73.49	21.79	70.34	5.92	2.50	82.47	6.18	3.66	98.50	96.90	25590.05	89430.98
	2011	76.05	64.78	11.27	3578.77	357.76	8.39	70.20	20.76	70.42	5.91	2.48	82.72	6.18	3.64	98.50	96.90	26440.77	89461.76
	2012	76.30	64.98	11.32	3481.69	354.06	8.00	69.15	21.56	68.83	5.51	2.50	82.97	6.19	3.60	98.60	96.90	27198.59	89489.87
	2013	76.46	65.13	11.33	3401.08	336.79	7.23	68.97	22.50	67.38	4.87	2.36	83.21	6.20	3.56	98.60	96.90	-	89530.54
	2014	76.60	65.26	11.34	3348.83	358.91	7.45	68.83	20.87	69.68	5.19	2.26	83.45	6.22	3.51	98.60	96.90	-	89588.66
	2015	76.71	65.37	11.34	3297.89	-	-	-	-	-	-	-	83.68	6.25	3.45	98.60	96.90	-	89659.48
	2016	76.29	65.10	11.19	3258.49	-	-	-	-	-	-	-	83.91	6.26	-	-	-	-	89715.45
	2017	79.33	67.73	11.60	-	-	-	-	-	-	-	-	-	-	-	-	-	-	-
Kazakhstan	1995	64.21	56.49	7.73	3738.47	48.25	4.62	98.57	35.53	63.96	2.95	1.66	55.92	11.36	2.26	96.50	94.00	247266.95	89254.32
	1996	63.92	56.30	7.62	3814.50	58.17	4.34	98.53	35.94	63.53	2.76	1.58	55.94	11.35	2.13	96.60	94.00	272328.50	89373.80
	1997	63.95	56.32	7.62	3941.12	66.97	4.67	98.60	35.24	64.26	3.00	1.67	55.96	11.11	2.00	96.60	93.90	207305.66	89500.58
	1998	64.11	56.46	7.65	3933.56	70.18	4.83	98.65	35.49	64.02	3.09	1.74	55.99	10.78	1.80	96.70	93.90	218453.24	89619.53
	1999	64.29	56.61	7.69	4078.43	49.02	4.38	98.87	46.99	52.47	2.30	2.08	55.93	10.52	1.70	96.70	93.80	182819.10	89737.41
	2000	64.42	56.70	7.73	4491.59	50.81	4.16	98.88	48.51	50.94	2.12	2.04	55.73	10.40	1.80	96.80	93.80	207395.42	89838.38
	2001	64.58	56.85	7.72	5106.63	51.41	3.47	98.50	42.71	56.64	1.97	1.51	55.53	10.66	1.90	96.80	93.70	223606.92	89875.35

	2002	64.71	56.96	7.75	5606.85	59.31	3.61	98.65	45.89	53.48	1.93	1.68	55.33	11.03	2.00	96.90	93.70	285884.29	89891.95
	2003	64.76	57.01	7.75	6107.71	75.36	3.70	98.65	44.88	54.51	2.01	1.68	55.12	11.35	2.03	97.00	93.60	256799.01	89887.53
	2004	64.73	56.99	7.73	6647.69	111.71	3.95	98.64	41.36	58.07	2.29	1.66	54.92	11.47	2.21	97.00	93.60	285197.38	89850.45
	2005	64.75	57.00	7.75	7227.98	149.99	4.07	98.57	37.49	61.96	2.52	1.55	54.72	11.32	2.22	97.10	93.50	291641.05	89798.32
	2006	65.05	57.25	7.80	7917.20	194.02	3.73	98.46	37.54	61.87	2.31	1.42	54.52	11.16	2.36	97.10	93.40	324812.47	89686.24
	2007	65.75	57.83	7.92	8523.77	212.10	3.19	98.45	56.01	56.01	1.78	1.40	54.31	10.85	2.50	97.20	93.40	315840.14	89559.46
	2008	66.70	58.57	8.13	8698.42	305.82	3.65	98.45	37.40	62.01	2.26	1.39	54.11	10.47	2.70	97.30	93.30	330512.05	89431.51
	2009	67.61	59.28	8.33	8573.77	295.32	4.13	98.53	35.36	64.12	2.65	1.48	53.91	10.13	2.55	97.30	93.20	287801.13	89304.99
	2010	68.34	59.86	8.48	9070.65	401.21	4.42	98.86	42.34	57.17	2.53	1.89	53.73	9.87	2.60	97.40	93.20	347356.08	89206.10
	2011	69.01	60.40	8.61	9603.59	460.55	4.06	98.80	43.46	56.02	2.27	1.78	53.58	9.92	2.59	97.40	93.10	350475.73	89111.74
	2012	69.71	60.97	8.74	9923.81	521.64	4.32	98.86	43.69	55.81	2.41	1.91	53.46	9.98	2.62	97.50	93.10	366502.20	89026.16
	2013	70.50	61.61	8.89	10368.67	582.10	4.30	98.95	48.56	50.92	2.19	2.11	53.36	10.02	2.64	97.50	93.00	-	88937.83
	2014	71.26	62.21	9.05	10645.46	538.78	4.36	98.90	45.14	54.36	2.37	1.99	53.29	10.06	2.74	97.50	92.90	-	88869.55
	2015	71.72	62.58	9.14	10616.68	-	-	-	-	-	-	-	53.25	10.16	2.73	97.50	92.90	-	88810.71
	2016	71.75	62.67	9.08	10580.93	-	-	-	-	-	-	-	53.23	10.40	-	-	-	-	88776.34
	2017	71.98	62.71	9.27	-	-	-	-	-	-	-	-	-	-	-	-	-	-	-
Kenya	1995	57.24	50.34	6.90	861.25	14.06	4.25	78.31	42.06	46.29	1.97	2.29	18.26	5.30	5.46	25.70	47.40	41798.48	89072.72
	1996	56.53	49.75	6.78	871.43	17.77	4.15	79.09	47.38	40.09	1.66	2.49	18.58	5.29	5.40	26.00	48.30	41134.09	89123.53
	1997	55.83	49.16	6.68	851.18	18.72	4.12	79.20	48.43	38.85	1.60	2.52	18.90	5.28	5.35	26.20	49.10	41235.59	89177.80
	1998	55.27	48.67	6.60	855.10	21.42	4.49	80.38	44.69	44.40	1.99	2.50	19.22	5.25	5.30	26.40	50.00	66595.01	89235.12
	1999	55.25	48.63	6.63	851.07	17.86	4.20	80.43	48.38	39.85	1.67	2.52	19.55	5.21	5.24	26.70	50.90	46998.10	89295.35
	2000	55.20	48.55	6.65	833.03	19.15	4.68	80.40	43.20	46.27	2.17	2.52	19.89	5.15	5.18	26.90	51.80	46237.25	89355.93
	2001	55.45	48.80	6.65	841.22	18.84	4.62	80.47	44.48	44.73	2.07	2.55	20.24	5.12	5.11	27.10	52.60	44415.73	89407.77
	2002	55.67	49.02	6.64	823.09	18.25	4.54	78.27	44.76	42.82	1.94	2.60	20.59	5.06	5.04	27.40	53.50	43784.87	89465.01
	2003	56.00	49.33	6.67	824.47	19.76	4.45	79.10	44.81	43.35	1.93	2.52	20.95	5.00	4.98	27.60	54.30	46308.66	89517.37
	2004	56.56	49.82	6.74	843.23	20.05	4.29	78.25	45.49	41.86	1.80	2.49	21.31	4.92	4.91	27.80	55.20	46544.16	89574.30
	2005	57.43	50.56	6.87	868.92	23.13	4.36	77.37	44.65	42.29	1.85	2.52	21.68	4.84	4.84	28.00	56.00	48661.63	89631.47
	2006	58.47	51.50	6.98	900.10	30.20	4.24	75.20	46.42	38.28	1.62	2.62	22.05	4.77	4.76	28.30	56.80	50017.92	89661.12
	2007	59.61	52.49	7.13	935.66	34.99	4.08	77.96	39.16	39.16	1.60	2.48	22.42	4.71	4.68	28.50	57.70	53054.24	89692.72

	2008	60.65	53.38	7.26	912.38	36.60	3.90	77.97	49.91	35.99	1.40	2.50	22.80	4.64	4.58	28.70	58.50	53277.03	89730.42
	2009	61.68	54.26	7.42	917.04	39.29	4.17	78.00	47.28	39.39	1.64	2.53	23.18	4.58	4.48	29.00	59.30	52547.68	89775.97
	2010	62.76	55.15	7.60	967.34	39.41	3.97	78.03	50.17	35.70	1.42	2.55	23.57	4.54	4.37	29.20	60.00	52675.10	89823.37
	2011	63.75	56.00	7.75	999.00	52.99	5.23	78.08	38.92	50.15	2.62	2.61	23.97	4.53	4.27	29.40	60.80	53513.99	89763.22
	2012	64.55	56.69	7.86	1016.83	65.06	5.49	67.28	26.56	60.52	3.32	2.17	24.37	4.53	4.17	29.60	61.60	54302.10	89714.36
	2013	65.16	57.20	7.95	1048.27	70.00	5.57	67.38	26.89	60.10	3.35	2.22	24.78	4.54	4.07	29.90	62.30	-	89677.75
	2014	65.74	57.69	8.05	1075.64	77.70	5.72	67.37	26.11	61.25	3.50	2.22	25.20	4.57	3.99	30.10	63.10	-	89648.92
	2015	66.30	58.15	8.16	1107.92	-	-	-	-	-	-	-	25.62	4.60	3.92	30.10	63.20	-	89632.38
	2016	66.84	58.59	8.25	1143.07	-	-	-	-	-	-	-	26.06	4.66	-	-	-	-	89613.87
	2017	65.86	57.62	8.23	-	-	-	-	-	-	-	-	-	-	-	-	-	-	-
Kiribati	1995	58.54	51.36	7.18	1630.71	69.60	9.39	2.57	0.12	95.50	8.97	0.42	36.41	6.27	4.37	30.50	53.30	40.16	85795.29
	1996	58.87	51.64	7.23	1633.62	79.16	9.20	2.57	0.11	95.59	8.80	0.41	37.39	6.25	4.30	31.20	54.30	42.23	85905.42
	1997	59.05	51.78	7.27	1634.39	80.69	9.36	2.57	0.10	96.00	8.98	0.37	38.75	6.19	4.24	32.00	55.40	43.72	86024.85
	1998	59.16	51.87	7.29	1712.39	57.55	7.00	2.57	0.12	95.17	6.66	0.34	40.14	6.11	4.17	32.70	56.60	39.11	86143.29
	1999	59.24	51.93	7.32	1657.27	75.03	8.82	2.57	0.11	95.77	8.44	0.37	41.54	5.99	4.11	33.50	57.70	40.42	86280.37
	2000	59.37	52.01	7.36	1730.81	64.96	8.12	2.57	0.17	93.36	7.58	0.54	42.96	5.85	4.06	34.20	58.90	42.70	86428.00
	2001	59.59	52.19	7.40	1676.52	66.17	9.00	2.57	0.15	94.32	8.49	0.51	43.47	5.94	4.02	34.80	59.70	43.93	86546.56
	2002	59.71	52.28	7.43	1710.43	75.31	9.10	2.57	0.15	94.08	8.56	0.54	43.49	6.02	3.98	35.20	60.30	44.79	86677.57
	2003	59.84	52.38	7.46	1714.29	101.20	9.98	2.57	0.13	94.87	9.47	0.51	43.51	6.04	3.95	35.70	61.00	44.64	86804.58
	2004	59.96	52.46	7.49	1655.68	117.32	10.39	2.57	0.12	95.19	9.89	0.50	43.53	6.01	3.93	36.10	61.60	46.23	86932.48
	2005	60.09	52.56	7.53	1704.11	115.64	10.06	2.57	0.15	94.05	9.46	0.60	43.55	5.96	3.91	36.50	62.20	-	87021.34
	2006	60.18	52.65	7.53	1642.73	121.34	10.93	0.85	0.12	85.66	9.36	1.57	43.57	5.93	3.90	37.00	62.90	-	87177.88
	2007	60.31	52.77	7.54	1643.16	174.44	13.66	0.55	83.47	83.47	11.40	2.26	43.60	5.93	3.88	37.40	63.50	51.38	87297.84
	2008	60.44	52.88	7.55	1577.92	167.77	12.23	0.61	0.10	84.32	10.31	1.92	43.65	5.92	3.87	37.90	64.10	-	87441.05
	2009	60.55	52.99	7.57	1549.00	154.75	12.24	0.64	0.10	84.67	10.36	1.88	43.70	5.91	3.86	38.30	64.80	52.70	87588.89
	2010	60.66	53.08	7.57	1493.16	154.16	10.52	0.55	0.09	82.95	8.73	1.79	43.77	5.88	3.84	38.80	65.40	-	87741.60
	2011	60.75	53.16	7.59	1471.55	171.54	10.42	0.55	0.10	82.22	8.57	1.85	43.86	5.91	3.82	39.20	66.10	-	87864.13
	2012	60.92	53.31	7.61	1519.48	170.27	10.37	0.55	0.11	80.13	8.31	2.06	43.95	5.90	3.80	39.70	66.70	-	88022.81
	2013	61.12	53.47	7.65	1578.88	158.06	10.15	0.55	0.10	81.07	8.23	1.92	44.06	5.92	3.77	39.70	66.80	-	88159.66

	2014	61.32	53.64	7.68	1565.24	154.17	10.21	0.55	0.10	81.18	8.29	1.92	44.17	5.96	3.73	39.70	66.80	-	88268.98
	2015	61.55	53.83	7.72	1696.47	-	-	-	-	-	-	-	44.30	6.04	3.69	39.70	66.90	-	88388.52
	2016	61.79	54.02	7.77	1685.99	-	-	-	-	-	-	-	44.45	6.19	-	-	-	-	88523.32
	2017	62.41	53.97	8.45	-	-	-	-	-	-	-	-	-	-	-	-	-	-	-
Kuwait	1995	76.10	65.43	10.67	41473.33	618.51	3.72	93.80	16.24	82.68	3.08	0.64	98.04	2.00	2.72	100.00	99.00	57167.73	86580.24
	1996	75.76	65.19	10.57	41185.04	666.27	3.47	93.80	19.28	79.45	2.75	0.71	98.06	1.98	2.80	100.00	99.00	56837.80	86562.15
	1997	75.57	65.04	10.53	40147.42	664.98	3.69	93.80	18.87	79.88	2.95	0.74	98.07	2.01	2.86	100.00	99.00	59684.91	86737.91
	1998	75.67	65.11	10.56	38874.51	651.79	4.44	93.80	19.70	79.00	3.51	0.93	98.09	2.07	2.90	100.00	99.00	63941.67	87021.22
	1999	76.15	65.48	10.67	35824.14	596.04	3.67	93.16	19.18	79.41	2.91	0.75	98.10	2.15	2.89	100.00	99.00	65097.15	87326.33
	2000	76.44	65.68	10.75	35792.71	490.55	2.51	93.16	22.35	76.01	1.91	0.60	98.11	2.25	2.85	100.00	99.00	65239.57	87613.67
	2001	76.47	65.68	10.79	35051.80	632.85	3.61	93.16	17.32	81.41	2.94	0.67	98.13	2.37	2.77	100.00	99.00	63455.72	87750.05
	2002	76.91	66.02	10.89	35522.73	665.50	3.56	93.16	18.79	79.83	2.85	0.72	98.14	2.52	2.69	100.00	99.00	62755.27	87846.77
	2003	77.18	66.23	10.95	41189.46	737.54	3.23	90.54	17.87	80.26	2.59	0.64	98.16	2.68	2.62	100.00	99.00	74340.53	87897.31
	2004	77.11	66.16	10.94	44820.20	756.08	2.76	90.54	18.34	79.75	2.20	0.56	98.17	2.85	2.55	100.00	99.00	82089.05	87944.80
	2005	77.07	66.13	10.94	47847.82	848.79	2.38	90.54	18.26	79.84	1.90	0.48	98.19	3.00	2.50	100.00	99.00	91416.66	88022.50
	2006	77.09	66.15	10.94	49268.26	957.25	2.25	90.54	17.13	81.08	1.83	0.43	98.20	2.95	2.45	100.00	99.00	87101.27	88191.81
	2007	77.19	66.22	10.98	49588.76	963.14	2.13	90.54	78.72	78.72	1.68	0.45	98.22	2.88	2.40	100.00	99.00	86228.46	88378.37
	2008	77.25	66.25	11.00	47965.01	1052.24	1.93	90.54	19.67	78.27	1.51	0.42	98.23	2.80	2.34	100.00	99.00	89301.44	88600.07
	2009	77.51	66.42	11.09	41936.82	1423.52	3.87	90.54	11.69	87.09	3.37	0.50	98.25	2.73	2.28	100.00	99.00	88824.88	88822.92
	2010	78.22	66.95	11.27	38497.62	1041.03	2.76	90.54	13.95	84.59	2.33	0.43	98.26	2.67	2.22	100.00	99.00	93785.57	89053.51
	2011	78.90	67.47	11.43	39652.17	1244.30	2.62	90.54	12.49	86.21	2.26	0.36	98.28	2.64	2.15	100.00	99.00	96788.19	89172.54
	2012	79.34	67.82	11.52	39733.29	1309.07	2.57	90.54	13.55	85.04	2.19	0.38	98.29	2.62	2.09	100.00	99.00	-	89310.89
	2013	79.74	68.13	11.61	37924.47	1252.53	2.56	90.54	13.95	84.59	2.17	0.39	98.31	2.61	2.05	100.00	99.00	-	89457.26
	2014	79.86	68.26	11.61	36259.39	1385.78	3.04	90.54	12.74	85.93	2.61	0.43	98.33	2.63	2.01	100.00	99.00	-	89614.29
	2015	79.78	68.24	11.54	35053.59	-	-	-	-	-	-	-	98.34	2.68	1.99	100.00	99.00	-	89783.12
	2016	79.70	68.24	11.46	35250.91	-	-	-	-	-	-	-	98.36	2.86	-	-	-	-	89878.11
	2017	83.12	70.75	12.38	-	-	-	-	-	-	-	-	-	-	-	-	-	-	-
Kyrgyzstan	1995	64.01	56.31	7.70	535.04	19.40	5.97	92.58	45.15	51.23	3.06	2.91	36.35	9.62	3.10	91.30	75.00	10488.68	88902.23
	1996	64.60	56.82	7.78	564.53	22.33	5.69	93.16	51.95	44.23	2.52	3.17	36.07	9.65	2.80	91.40	75.60	12527.74	89080.69

	1997	65.28	57.38	7.91	611.52	19.54	5.23	92.78	47.37	48.95	2.56	2.67	35.79	9.57	2.60	91.50	76.30	11417.86	89276.28
	1998	65.76	57.76	7.99	614.99	20.01	5.88	96.46	54.82	43.17	2.54	3.34	35.50	9.42	2.70	91.60	77.00	11119.91	89488.19
	1999	66.12	58.07	8.06	628.07	13.63	5.34	89.09	51.51	42.19	2.25	3.09	35.30	9.31	2.60	91.70	77.70	9840.12	89687.96
	2000	66.32	58.22	8.10	654.31	12.91	4.68	89.29	49.76	44.28	2.07	2.61	35.30	9.25	2.40	91.80	78.40	9973.96	89871.89
	2001	66.54	58.45	8.10	682.62	14.61	4.79	90.57	53.31	41.14	1.97	2.82	35.30	9.30	2.40	91.90	79.20	8996.75	89967.55
	2002	66.49	58.45	8.04	676.27	17.37	5.43	91.05	55.04	39.55	2.15	3.28	35.30	9.36	2.40	92.00	80.00	10365.59	90053.95
	2003	66.47	58.48	7.99	716.26	20.63	5.43	92.95	57.73	37.89	2.06	3.37	35.29	9.37	2.50	92.10	80.70	10992.91	90128.18
	2004	66.56	58.60	7.96	757.37	24.38	5.57	94.33	55.82	40.83	2.28	3.30	35.29	9.22	2.60	92.20	81.50	11243.60	90172.19
	2005	66.49	58.59	7.90	747.57	27.99	5.82	94.72	55.99	40.89	2.38	3.44	35.29	8.90	2.50	92.30	82.30	10852.47	90215.09
	2006	66.50	58.62	7.88	762.52	37.05	6.73	93.76	48.14	48.66	3.28	3.46	35.29	8.56	2.70	92.40	83.00	10680.91	90170.82
	2007	66.79	58.87	7.92	819.81	50.10	6.87	92.70	51.36	51.36	3.53	3.34	35.29	8.12	2.70	92.50	83.80	11464.26	90110.46
	2008	67.27	59.26	8.01	880.28	58.61	6.07	87.18	42.26	51.53	3.13	2.94	35.29	7.66	2.80	92.60	84.60	11873.26	90014.18
	2009	67.95	59.80	8.15	894.82	59.02	6.79	88.46	39.18	55.71	3.79	3.01	35.28	7.25	2.90	92.70	85.30	13218.81	89900.53
	2010	68.37	60.12	8.25	880.04	58.28	6.66	87.33	38.66	55.73	3.71	2.95	35.30	6.90	3.10	92.80	86.10	13008.54	89785.20
	2011	69.04	60.65	8.38	921.18	69.08	6.18	86.00	34.49	59.89	3.70	2.48	35.34	6.79	3.10	92.90	86.90	13853.67	89675.00
	2012	69.51	61.03	8.48	905.17	81.66	6.97	88.47	35.22	60.19	4.20	2.78	35.40	6.70	3.20	93.00	87.60	13794.74	89568.33
	2013	70.08	61.48	8.60	984.24	85.26	6.68	89.09	37.33	58.09	3.88	2.80	35.48	6.63	3.10	93.10	88.40	-	89482.05
	2014	70.64	61.92	8.71	1003.51	81.57	6.48	89.82	39.40	56.13	3.64	2.84	35.59	6.59	3.20	93.20	89.20	-	89431.22
	2015	71.07	62.27	8.79	1021.16	-	-	-	-	-	-	-	35.71	6.62	3.20	93.30	90.00	-	89396.52
	2016	71.24	62.45	8.78	1038.31	-	-	-	-	-	-	-	35.85	6.79	-	-	-	-	89390.10
	2017	72.71	63.45	9.27	-	-	-	-	-	-	-	-	-	-	-	-	-	-	-
Laos	1995	54.50	47.90	6.59	547.36	14.37	3.77	88.97	37.55	57.80	2.18	1.59	17.38	6.78	5.39	20.40	39.90	31879.68	87134.78
	1996	54.98	48.36	6.62	572.86	14.53	3.67	91.02	45.67	49.82	1.83	1.84	18.23	6.81	5.17	20.80	40.20	24691.47	87074.37
	1997	55.50	48.82	6.68	600.19	16.67	4.61	90.54	49.83	44.97	2.07	2.54	19.12	6.82	4.94	21.30	40.50	24955.41	87037.94
	1998	55.94	49.21	6.73	612.59	13.66	5.43	90.54	59.66	34.11	1.85	3.58	20.04	6.81	4.72	21.80	40.80	141736.46	87033.32
	1999	56.51	49.71	6.80	646.00	10.08	3.77	91.81	64.75	29.47	1.11	2.66	20.99	6.78	4.51	24.90	43.20	143278.75	87025.18
	2000	57.03	50.14	6.89	672.17	10.46	3.41	91.81	61.39	33.14	1.13	2.28	21.98	6.74	4.31	28.00	45.50	24667.95	87049.25
	2001	57.53	50.62	6.91	699.63	13.21	4.32	67.41	49.03	27.27	1.18	3.14	22.99	6.73	4.14	31.10	47.80	15958.36	87150.61
	2002	58.34	51.31	7.03	729.89	12.91	4.04	69.16	50.62	26.80	1.08	2.96	24.04	6.71	3.98	34.20	50.10	34783.70	87246.44

	2003	59.00	51.87	7.13	762.75	17.73	4.91	74.85	55.48	25.88	1.27	3.64	25.13	6.67	3.85	37.30	52.30	20412.98	87363.37
	2004	59.68	52.46	7.23	799.07	19.08	4.54	74.82	61.62	17.65	0.80	3.74	26.24	6.61	3.72	40.30	54.60	76333.64	87497.65
	2005	60.27	52.96	7.32	842.57	20.41	4.32	75.15	62.36	17.02	0.73	3.58	27.39	6.53	3.62	43.40	56.80	53444.81	87618.84
	2006	60.92	53.55	7.37	900.28	25.13	4.14	75.15	54.35	27.68	1.15	3.00	28.54	6.46	3.52	46.50	59.00	25073.37	87624.75
	2007	61.66	54.21	7.45	952.32	29.40	4.14	75.15	24.85	24.85	1.03	3.11	29.69	6.38	3.42	49.60	61.20	70977.96	87627.04
	2008	62.34	54.81	7.52	1009.46	24.22	2.77	55.19	35.24	36.15	1.00	1.77	30.84	6.30	3.33	52.60	63.30	21335.61	87601.95
	2009	63.05	55.43	7.62	1067.58	34.22	3.77	59.44	30.15	49.28	1.86	1.91	31.99	6.22	3.24	55.70	65.40	24469.64	87568.10
	2010	63.79	56.08	7.71	1141.13	30.02	2.75	78.25	41.82	46.55	1.28	1.47	33.12	6.15	3.15	58.70	67.50	38034.96	87541.41
	2011	64.55	56.72	7.83	1215.88	27.81	2.20	83.57	51.77	38.05	0.84	1.36	34.25	6.14	3.06	61.70	69.50	161456.98	87395.16
	2012	65.16	57.25	7.91	1296.75	30.76	2.12	83.82	52.48	37.39	0.79	1.33	35.37	6.14	2.97	64.60	71.50	161718.74	87246.34
	2013	65.70	57.72	7.97	1383.70	32.70	2.00	78.80	40.78	48.25	0.96	1.03	36.47	6.15	2.90	67.60	73.50	-	87098.64
	2014	66.27	58.21	8.06	1470.50	32.57	1.87	78.80	38.98	50.53	0.94	0.92	37.55	6.18	2.82	70.50	75.50	-	86955.68
	2015	66.77	58.64	8.13	1556.67	-	-	-	-	-	-	-	38.61	6.23	2.76	70.90	75.70	-	86834.43
	2016	67.25	59.08	8.17	1642.73	-	-	-	-	-	-	-	39.65	6.30	-	-	-	-	86712.15
	2017	67.58	59.48	8.11	-	-	-	-	-	-	-	-	-	-	-	-	-	-	-
Latvia	1995	66.43	58.04	8.39	5140.53	114.73	5.76	100.00	33.73	66.27	3.82	1.94	68.75	20.94	1.25	-	98.30	18783.00	91773.11
	1996	68.52	59.72	8.81	5321.40	139.97	6.10	98.23	41.49	57.77	3.53	2.58	68.65	21.38	1.16	-	98.30	18788.76	91990.87
	1997	69.25	60.31	8.94	5857.04	159.21	6.20	97.93	43.31	55.77	3.46	2.74	68.60	21.68	1.11	81.80	98.30	18051.45	92204.94
	1998	68.99	60.14	8.85	6298.13	175.30	6.12	97.22	39.63	59.24	3.63	2.50	68.45	21.89	1.09	81.80	98.30	12905.80	92390.96
	1999	69.96	60.92	9.04	6515.89	194.71	6.39	97.20	40.48	58.35	3.73	2.66	68.22	22.09	1.16	81.80	98.30	12076.29	92558.44
	2000	70.50	61.37	9.13	6934.72	196.41	6.00	96.78	44.14	54.39	3.27	2.74	68.07	22.32	1.25	81.70	98.30	11507.81	92711.27
	2001	69.92	60.96	8.96	7478.76	216.32	6.17	95.28	46.49	51.21	3.16	3.01	67.95	22.85	1.22	81.70	98.30	12089.66	92723.05
	2002	70.27	61.27	9.01	8103.59	251.62	6.29	94.37	45.17	52.14	3.28	3.01	67.85	23.38	1.26	82.10	98.30	13748.35	92745.78
	2003	70.68	61.63	9.06	8872.10	300.98	6.15	96.80	45.67	52.82	3.25	2.90	67.80	23.88	1.32	82.60	98.40	12783.26	92756.61
	2004	71.03	61.94	9.10	9717.11	396.98	6.51	93.68	40.67	56.58	3.68	2.83	67.90	24.30	1.29	83.00	98.50	13021.82	92757.14
	2005	70.83	61.84	8.99	10873.41	455.84	6.37	94.78	40.70	57.06	3.64	2.74	68.00	24.62	1.39	83.50	98.60	13288.20	92753.77
	2006	70.49	61.59	8.90	12278.31	612.62	6.80	92.72	33.29	64.09	4.36	2.44	67.97	25.18	1.46	83.90	98.60	14054.37	92707.09
	2007	70.93	61.87	9.06	13614.26	915.23	7.01	95.09	60.69	60.69	4.26	2.75	67.90	25.66	1.54	84.30	98.70	14416.14	92656.32
	2008	71.99	62.67	9.32	13270.00	1018.76	6.63	94.52	35.32	62.21	4.12	2.48	67.83	26.09	1.58	84.80	98.80	14204.67	92620.54

	2009	72.77	63.25	9.52	11547.99	817.20	6.84	97.32	36.06	59.51	4.07	2.53	67.76	26.48	1.46	85.20	98.90	13580.32	92623.42
	2010	73.23	63.65	9.58	11326.22	739.38	6.55	93.54	35.11	60.11	3.94	2.46	67.69	26.84	1.36	85.60	98.90	14670.00	92651.83
	2011	73.81	64.06	9.75	12270.34	827.44	6.10	94.10	32.10	63.50	3.87	2.08	67.62	27.37	1.33	86.10	99.00	14150.10	92674.82
	2012	74.22	64.39	9.82	12924.73	826.55	5.91	93.58	34.39	60.55	3.58	2.17	67.55	27.88	1.44	86.50	99.10	13943.92	92678.77
	2013	74.28	64.48	9.81	13400.91	868.85	5.67	95.40	35.83	62.44	3.54	2.13	67.48	28.38	1.52	86.90	99.20	-	92646.44
	2014	74.81	64.90	9.91	13786.43	920.70	5.88	95.41	35.13	63.18	3.72	2.17	67.42	28.88	1.64	87.40	99.30	-	92615.16
	2015	74.95	65.03	9.92	14294.04	-	-	-	-	-	-	-	67.38	29.40	1.64	87.80	99.30	-	92570.98
	2016	75.06	65.21	9.85	14718.03	-	-	-	-	-	-	-	67.36	29.93	-	-	-	-	92527.04
	2017	75.17	64.58	10.59	-	-	-	-	-	-	-	-	-	-	-	-	-	-	-
Lebanon	1995	73.54	62.79	10.75	6703.23	460.52	12.56	80.71	53.46	33.77	4.24	8.32	84.82	10.22	2.62	-	83.30	15558.42	90308.06
	1996	74.07	63.20	10.87	6961.33	568.51	13.44	80.73	50.74	37.15	4.99	8.45	85.14	10.39	2.54	-	83.30	16648.26	90366.19
	1997	74.72	63.75	10.97	6754.39	665.04	13.06	80.64	49.40	38.75	5.06	8.00	85.46	10.55	2.47	-	83.30	18223.05	90443.73
	1998	75.31	64.23	11.08	6949.23	634.16	11.42	82.26	59.61	27.53	3.14	8.28	85.77	10.71	2.39	82.70	83.30	18388.35	90536.45
	1999	76.03	64.79	11.24	6824.18	599.33	10.87	81.49	59.43	27.08	2.94	7.93	85.89	10.88	2.31	82.70	84.50	18215.94	90654.88
	2000	76.47	65.26	11.21	6747.63	579.03	10.86	80.67	56.85	29.52	3.21	7.66	86.00	11.06	2.23	82.70	85.70	16825.66	90790.38
	2001	76.98	65.57	11.41	6749.15	571.03	10.90	79.71	52.42	34.24	3.73	7.17	86.12	11.09	2.13	82.70	86.90	18142.41	90903.91
	2002	77.37	65.89	11.48	6656.74	544.44	10.05	78.54	49.33	37.18	3.74	6.31	86.23	11.16	2.04	82.70	88.10	17968.86	91024.73
	2003	77.70	66.17	11.53	6539.66	496.31	9.30	77.34	48.27	37.59	3.50	5.81	86.35	11.27	1.94	82.40	89.30	20293.95	91159.03
	2004	78.01	66.43	11.58	6636.30	483.33	8.91	75.95	44.31	41.66	3.71	5.20	86.47	11.40	1.84	82.20	90.50	18716.22	91287.59
	2005	78.26	66.80	11.46	6606.29	449.54	8.42	77.16	42.85	44.47	3.74	4.68	86.58	11.55	1.75	82.00	91.70	19431.45	91396.29
	2006	77.95	66.58	11.37	6592.54	474.40	8.83	75.23	44.29	41.13	3.63	5.20	86.70	11.72	1.68	81.80	92.90	16947.88	91477.52
	2007	78.63	66.95	11.69	7157.55	535.63	8.90	73.47	41.73	41.73	3.72	5.19	86.82	11.87	1.63	81.60	94.10	15102.07	91550.70
	2008	79.12	67.33	11.79	7859.94	566.25	8.07	71.90	43.89	38.97	3.14	4.93	86.94	12.02	1.60	81.30	95.30	18270.36	91631.89
	2009	79.45	67.58	11.87	8501.11	623.90	7.42	73.15	42.38	42.07	3.12	4.30	87.06	12.19	1.60	81.10	96.50	-	91732.79
	2010	79.81	68.05	11.76	8858.28	630.23	7.19	73.15	45.60	37.66	2.71	4.48	87.18	12.37	1.61	80.90	97.70	-	91862.14
	2011	78.99	67.20	11.79	8450.11	621.85	7.12	74.74	45.95	38.52	2.74	4.38	87.30	12.23	1.64	80.70	99.00	-	91921.04
	2012	80.15	68.13	12.02	8107.44	626.09	6.99	69.51	37.34	46.29	3.24	3.76	87.43	12.12	1.67	80.70	99.00	-	91980.94
	2013	79.92	67.96	11.96	7753.88	597.19	6.63	69.51	37.50	46.04	3.05	3.58	87.55	12.04	1.70	80.70	99.00	-	92045.48
	2014	80.00	68.03	11.97	7447.36	568.71	6.39	69.51	36.42	47.61	3.04	3.35	87.67	12.00	1.71	80.70	99.00	-	92091.34

	2015	80.04	68.07	11.97	7189.63	-	-	-	-	-	-	-	87.79	12.00	1.72	80.70	99.00	-	92124.10
	2016	80.07	68.28	11.79	7143.96	-	-	-	-	-	-	-	87.91	12.21	-	-	-	-	92212.98
	2017	77.97	66.26	11.71	-	-	-	-	-	-	-	-	-	-	-	-	-	-	-
Lesotho	1995	57.68	50.23	7.45	804.84	36.55	7.46	80.64	42.14	47.73	3.56	3.90	16.96	7.95	4.55	22.70	78.00	1938.44	88827.82
	1996	56.46	49.20	7.27	837.56	34.38	7.50	79.76	39.54	50.42	3.78	3.72	17.57	8.06	4.48	22.80	78.20	1839.49	88836.12
	1997	54.86	47.83	7.03	857.46	32.06	6.72	77.10	41.32	46.40	3.12	3.60	18.05	8.16	4.39	22.80	78.40	1810.89	88843.54
	1998	52.70	46.02	6.69	860.74	30.81	6.85	71.57	37.38	47.77	3.27	3.58	18.54	8.24	4.30	22.90	78.60	4735.80	88859.84
	1999	50.85	44.43	6.43	855.85	29.45	6.76	71.35	37.16	47.92	3.24	3.52	19.04	8.29	4.20	23.40	78.80	3817.01	88872.16
	2000	48.82	42.60	6.23	880.37	28.73	6.92	71.13	35.40	50.24	3.47	3.44	19.55	8.28	4.09	23.90	79.00	3230.58	88894.11
	2001	47.50	41.49	6.01	903.38	28.42	7.53	71.88	30.14	58.07	4.37	3.16	20.07	8.31	3.97	24.30	79.10	3844.31	88882.21
	2002	46.06	40.27	5.78	902.10	24.06	6.91	69.01	30.74	55.46	3.83	3.08	20.60	8.32	3.86	24.80	79.30	2941.82	88873.49
	2003	44.73	39.15	5.58	935.47	36.39	7.13	69.01	29.96	56.59	4.03	3.09	21.14	8.29	3.74	25.30	79.50	4650.11	88862.81
	2004	43.82	38.34	5.48	943.61	44.95	6.96	69.01	29.99	56.55	3.94	3.03	21.69	8.21	3.64	25.70	79.70	2341.00	88850.82
	2005	43.27	37.77	5.50	968.40	44.73	6.30	69.01	32.73	52.57	3.31	2.99	22.25	8.10	3.55	26.20	79.90	3703.90	88864.05
	2006	43.24	37.81	5.43	1001.09	52.40	7.12	69.01	28.51	58.68	4.18	2.94	22.82	8.00	3.47	26.70	80.10	2161.30	88863.73
	2007	43.67	38.23	5.44	1040.67	69.17	8.47	69.01	66.10	66.10	5.60	2.87	23.29	7.87	3.41	27.10	80.30	4439.43	88858.82
	2008	44.36	38.82	5.54	1101.00	73.18	8.85	69.01	21.76	68.46	6.06	2.79	23.77	7.73	3.37	27.60	80.50	2543.50	88858.78
	2009	45.44	39.71	5.73	1113.98	84.22	9.80	69.01	19.69	71.47	7.00	2.79	24.26	7.62	3.33	28.00	80.70	2727.98	88866.04
	2010	46.53	40.53	6.00	1169.27	118.26	10.87	69.01	17.71	74.33	8.08	2.79	24.75	7.55	3.30	28.50	80.80	3425.28	88879.84
	2011	47.56	41.46	6.10	1235.66	146.32	11.79	69.01	15.81	77.09	9.09	2.70	25.25	7.50	3.28	28.90	81.00	3449.41	88873.12
	2012	47.88	41.79	6.10	1293.63	129.15	11.14	69.01	16.70	75.80	8.45	2.70	25.76	7.47	3.25	29.30	81.20	3472.71	88871.01
	2013	48.09	41.99	6.10	1300.41	114.16	11.07	69.01	16.61	75.94	8.41	2.66	26.27	7.45	3.22	29.80	81.40	-	88863.35
	2014	48.57	42.41	6.16	1323.24	105.11	10.62	69.01	16.48	76.12	8.08	2.54	26.79	7.43	3.19	30.20	81.60	-	88864.21
	2015	49.50	43.18	6.32	1338.54	-	-	-	-	-	-	-	27.31	7.41	3.14	30.30	81.80	-	88885.17
	2016	50.31	43.82	6.49	1352.48	-	-	-	-	-	-	-	27.84	7.46	-	-	-	-	88884.47
	2017	54.66	46.95	7.71	-	-	-	-	-	-	-	-	-	-	-	-	-	-	-
Liberia	1995	50.28	42.59	7.70	115.79	-	-	-	-	-	-	-	45.96	5.71	6.16	13.10	61.30	1428.10	89774.93
	1996	49.21	41.80	7.41	122.86	-	-	-	-	-	-	-	42.96	5.68	6.11	12.50	60.50	1491.94	89894.75
	1997	52.33	44.38	7.95	235.45	-	-	-	-	-	-	-	43.30	5.67	6.06	12.60	60.60	1602.41	90019.99

	1998	52.60	44.67	7.93	283.43	8.33	5.92	50.34	37.93	24.65	1.46	4.46	43.65	5.68	6.01	12.60	60.70	4980.42	90100.18
	1999	53.60	45.54	8.06	321.90	9.73	6.04	50.34	37.23	26.05	1.57	4.46	43.99	5.71	5.95	12.90	61.60	3371.33	90154.69
	2000	54.44	46.28	8.16	392.49	10.82	5.91	50.34	38.01	24.50	1.45	4.46	44.33	5.77	5.88	13.10	62.40	2076.32	90169.86
	2001	55.26	46.98	8.28	389.55	11.00	6.41	50.61	37.60	25.71	1.65	4.76	44.67	5.77	5.80	13.40	63.30	2163.91	90054.75
	2002	54.85	46.70	8.14	394.74	9.47	5.43	50.21	40.12	20.10	1.09	4.34	45.02	5.74	5.72	13.60	64.20	2047.19	89953.03
	2003	54.85	46.74	8.11	271.02	4.50	3.44	46.06	30.12	34.60	1.19	2.25	45.36	5.71	5.64	13.80	65.00	2062.91	89859.66
	2004	57.54	48.95	8.60	272.86	12.87	8.77	51.64	37.43	27.51	2.41	6.36	45.71	5.67	5.55	14.10	65.90	2311.55	89808.37
	2005	58.14	49.47	8.67	279.80	13.32	8.04	51.69	41.70	19.33	1.55	6.48	46.05	5.62	5.46	14.30	66.80	2082.70	89792.23
	2006	58.65	49.96	8.69	292.04	18.42	10.90	52.51	42.86	18.38	2.00	8.90	46.40	5.66	5.37	14.60	67.60	2475.90	89834.84
	2007	59.15	50.42	8.73	307.40	21.47	10.24	52.09	27.04	27.04	2.77	7.47	46.74	5.70	5.28	14.80	68.50	2847.61	89875.92
	2008	59.73	50.96	8.78	315.88	27.37	11.83	52.24	35.00	33.00	3.90	7.92	47.09	5.73	5.19	15.10	69.40	2863.14	89929.89
	2009	60.24	51.42	8.82	319.66	43.50	14.39	30.05	23.09	23.18	3.34	11.05	47.44	5.75	5.11	15.30	70.30	2599.80	89974.25
	2010	60.75	51.90	8.85	327.42	38.76	11.87	35.17	27.04	23.10	2.74	9.13	47.80	5.73	5.02	15.60	71.10	2758.55	90016.08
	2011	61.37	52.46	8.91	343.65	42.34	11.23	44.83	29.16	34.96	3.93	7.31	48.17	5.73	4.94	15.80	72.00	2797.37	90028.37
	2012	62.09	53.11	8.97	361.23	42.15	10.19	44.85	29.94	33.24	3.39	6.80	48.54	5.69	4.87	16.10	72.90	2833.94	90040.90
	2013	62.72	53.70	9.02	383.08	41.93	9.25	44.85	31.12	30.60	2.83	6.42	48.92	5.63	4.79	16.40	73.80	-	90060.23
	2014	59.36	51.05	8.31	376.59	46.27	10.04	44.85	30.73	31.48	3.16	6.88	49.31	5.58	4.72	16.60	74.70	-	90094.91
	2015	63.80	54.71	9.09	367.48	-	-	-	-	-	-	-	49.70	5.54	4.65	16.90	75.60	-	90130.35
	2016	64.38	55.27	9.11	352.65	-	-	-	-	-	-	-	50.10	5.54	-	-	-	-	90158.16
	2017	64.41	54.33	10.07	-	-	-	-	-	-	-	-	-	-	-	-	-	-	-
Libya	1995	74.04	63.34	10.69	-	190.09	3.28	100.00	53.79	46.21	1.51	1.76	75.99	5.84	3.64	96.50	71.20	65215.47	89323.15
	1996	74.06	63.37	10.69	-	210.86	3.35	100.00	50.37	49.63	1.66	1.69	76.05	5.90	3.44	96.50	71.20	67250.67	89371.08
	1997	74.30	63.58	10.72	-	226.65	3.46	100.00	48.21	51.79	1.79	1.67	76.11	5.95	3.27	96.50	71.20	63411.21	89433.82
	1998	74.34	63.63	10.71	-	226.26	3.90	100.00	51.68	48.32	1.88	2.02	76.18	5.99	3.11	96.50	71.20	63333.03	89511.28
	1999	74.39	63.67	10.72	8782.83	233.89	3.65	100.00	54.75	45.25	1.65	2.00	76.26	6.02	2.98	96.50	71.20	60164.76	89606.43
	2000	74.34	63.63	10.70	8967.25	245.62	3.41	100.00	51.29	48.71	1.66	1.75	76.35	6.04	2.86	96.50	71.20	63666.89	89718.04
	2001	74.55	63.81	10.74	8671.84	254.61	4.06	100.00	45.53	54.47	2.21	1.85	76.44	6.06	2.75	96.50	71.20	62555.04	89758.19
	2002	74.61	63.87	10.74	8453.58	181.40	4.57	100.00	33.09	66.91	3.06	1.51	76.54	6.06	2.67	96.50	-	63328.59	89823.38
	2003	74.79	64.01	10.77	9403.45	190.07	4.06	100.00	33.76	66.24	2.69	1.37	76.66	6.06	2.60	96.50	-	68226.67	89921.74

	2004	74.94	64.14	10.80	9670.08	202.01	3.46	100.00	34.21	65.79	2.28	1.18	76.77	6.06	2.54	96.50	-	68566.55	90010.38
	2005	75.17	64.32	10.84	10653.78	211.95	2.71	100.00	35.35	64.65	1.75	0.96	76.90	6.05	2.49	96.50	-	69931.74	90111.22
	2006	75.38	64.51	10.88	11175.13	233.94	2.51	100.00	34.63	65.37	1.64	0.87	77.03	6.11	2.46	96.50	-	71318.67	90177.32
	2007	75.51	64.58	10.93	11707.99	273.51	2.63	100.00	66.56	66.56	1.75	0.88	77.18	6.16	2.44	96.50	-	71090.57	90257.12
	2008	75.67	64.69	10.98	11856.02	317.77	2.04	100.00	32.27	67.73	1.38	0.66	77.32	6.20	2.43	96.60	-	74238.82	90344.37
	2009	75.84	64.80	11.03	11631.72	350.64	3.16	100.00	31.12	68.88	2.18	0.98	77.48	6.23	2.42	96.60	-	73995.42	90446.52
	2010	76.05	64.98	11.07	12120.56	394.34	3.05	100.00	30.00	70.00	2.14	0.92	77.64	6.22	2.41	96.60	-	77780.09	90563.55
	2011	60.25	52.23	8.03	4578.53	307.86	4.77	100.00	36.69	63.31	3.02	1.75	77.81	6.28	2.40	96.60	-	80072.82	90682.90
	2012	75.50	64.55	10.95	-	655.43	4.30	100.00	29.75	70.25	3.02	1.28	77.99	6.32	2.39	96.60	-	82129.13	90833.90
	2013	75.90	64.88	11.03	-	516.07	4.33	100.00	30.33	69.67	3.02	1.31	78.17	6.35	2.37	96.60	-	-	90981.78
	2014	74.37	63.62	10.75	-	371.72	4.97	100.00	26.46	73.54	3.65	1.31	78.36	6.39	2.35	96.60	-	-	91159.87
	2015	74.08	63.44	10.64	-	-	-	-	-	-	-	-	78.55	6.45	2.31	96.60	-	-	91354.40
	2016	75.05	64.21	10.85	-	-	-	-	-	-	-	-	78.75	6.49	-	-	-	-	91581.98
	2017	72.96	62.13	10.82	-	-	-	-	-	-	-	-	-	-	-	-	-	-	-
Lithuania	1995	68.92	60.13	8.79	5322.42	99.33	5.37	86.63	22.39	74.15	3.99	1.39	67.29	18.63	1.55	84.70	89.10	29219.71	91322.75
	1996	70.17	61.14	9.02	5639.24	130.21	5.59	88.36	26.18	70.37	3.93	1.65	67.23	19.21	1.49	85.00	89.50	30160.76	91483.33
	1997	71.11	61.86	9.24	6152.14	162.01	5.74	88.97	24.46	72.51	4.16	1.58	67.17	19.74	1.47	85.40	89.90	29876.40	91643.51
	1998	71.38	62.08	9.29	6659.60	192.03	6.08	95.74	23.03	75.95	4.62	1.46	67.11	20.21	1.46	85.80	90.20	25363.91	91780.41
	1999	71.83	62.43	9.40	6630.91	192.45	6.20	99.56	24.98	74.91	4.65	1.56	67.05	20.65	1.46	86.20	90.60	22732.14	91911.54
	2000	72.10	62.64	9.46	6933.59	211.49	6.46	86.22	26.12	69.71	4.50	1.96	66.99	21.06	1.39	86.60	91.00	21257.13	92071.40
	2001	71.61	62.29	9.33	7447.08	219.45	6.26	97.01	26.54	72.65	4.55	1.71	66.92	21.62	1.29	86.90	91.40	21048.71	92215.69
	2002	72.00	62.60	9.40	8014.64	263.65	6.40	98.21	24.63	74.92	4.79	1.60	66.83	22.18	1.23	87.30	91.80	21963.75	92379.84
	2003	72.03	62.65	9.39	8931.52	352.04	6.46	98.22	23.26	76.32	4.93	1.53	66.74	22.73	1.26	87.70	92.20	21789.31	92514.09
	2004	72.01	62.62	9.39	9624.01	378.28	5.67	98.44	31.93	67.55	3.83	1.84	66.65	23.27	1.27	88.10	92.60	22521.04	92629.90
	2005	71.34	62.15	9.19	10537.91	453.88	5.83	98.55	31.75	67.77	3.95	1.88	66.64	23.77	1.29	88.50	93.00	23569.41	92738.40
	2006	71.07	61.94	9.13	11500.53	569.67	6.20	98.36	29.98	69.51	4.31	1.89	66.71	24.22	1.33	88.90	93.40	24082.21	92768.08
	2007	70.81	61.71	9.10	12928.26	753.19	6.22	98.33	72.98	72.98	4.54	1.68	66.78	24.60	1.36	89.30	93.80	25791.10	92779.09
	2008	71.55	62.27	9.27	13405.19	976.91	6.61	97.97	27.04	72.40	4.78	1.82	66.85	24.91	1.45	89.70	94.20	26732.65	92770.92
	2009	73.04	63.39	9.65	11546.82	876.78	7.53	97.40	26.46	72.82	5.49	2.05	66.84	25.18	1.50	90.10	94.60	24473.52	92758.76

	2010	73.21	63.57	9.64	11984.87	828.65	7.09	97.78	26.83	70.81	5.02	1.95	66.76	25.41	1.50	90.50	95.00	26786.48	92761.28
	2011	73.58	63.83	9.75	13000.19	967.81	6.86	97.59	27.02	69.12	4.74	1.90	66.67	25.91	1.55	90.80	95.40	29324.69	92736.08
	2012	73.96	64.15	9.82	13681.00	940.10	6.67	97.49	30.36	65.23	4.35	2.08	66.60	26.41	1.60	91.20	95.80	29442.21	92733.22
	2013	74.12	64.29	9.83	14304.80	1019.79	6.59	97.37	30.87	65.44	4.31	2.09	66.55	26.92	1.59	91.60	96.20	-	92733.50
	2014	74.83	64.83	9.99	14932.60	1063.42	6.55	97.31	31.27	67.87	4.45	2.11	66.52	27.44	1.63	92.00	96.60	-	92763.28
	2015	74.97	64.96	10.01	15341.84	-	-	-	-	-	-	-	66.51	28.01	1.63	92.40	96.60	-	92797.88
	2016	75.16	65.20	9.96	15872.72	-	-	-	-	-	-	-	66.51	28.35	-	-	-	-	92846.43
	2017	75.02	64.33	10.68	-	-	-	-	-	-	-	-	-	-	-	-	-	-	-
Luxembourg	1995	76.84	66.51	10.32	74776.81	2841.61	5.57	100.00	6.19	92.44	5.15	0.34	82.89	20.56	1.70	97.70	100.00	10728.91	92460.48
	1996	77.07	66.72	10.35	74790.07	2830.06	5.65	100.03	7.20	92.81	5.25	0.41	82.83	20.77	1.77	97.70	100.00	10528.99	92423.83
	1997	77.33	66.95	10.38	78075.53	2489.74	5.59	100.00	7.45	92.48	5.17	0.42	82.78	20.90	1.71	97.70	100.00	10135.52	92428.21
	1998	77.72	67.26	10.46	81771.44	2598.89	5.67	100.00	7.58	92.42	5.24	0.43	82.89	20.97	1.68	97.70	100.00	9328.09	92444.67
	1999	78.10	67.57	10.53	87516.45	2891.01	5.83	72.91	7.36	89.79	5.23	0.59	83.45	21.00	1.74	97.70	100.00	9683.29	92433.90
	2000	78.51	67.88	10.62	93462.92	3500.00	7.48	79.05	11.80	85.07	6.36	1.12	84.22	21.01	1.76	97.70	100.00	10268.36	92456.26
	2001	78.75	68.10	10.65	94695.34	3405.29	7.40	79.13	12.46	84.25	6.24	1.17	84.84	21.19	1.66	97.70	100.00	10701.18	92470.43
	2002	79.01	68.32	10.69	97287.60	4202.19	8.27	81.74	11.82	85.53	7.07	1.20	85.30	21.34	1.63	97.70	100.00	11557.26	92513.43
	2003	79.13	68.45	10.68	97678.46	4987.75	7.67	77.49	12.23	84.22	6.46	1.21	85.74	21.45	1.62	97.70	100.00	12047.22	92548.41
	2004	79.60	68.82	10.78	99778.47	6145.32	8.20	76.65	11.64	84.81	6.96	1.25	86.18	21.51	1.66	97.70	100.00	13115.92	92583.88
	2005	80.04	69.17	10.88	101380.7	6488.07	7.95	76.90	11.59	84.92	6.75	1.20	86.60	21.54	1.63	97.70	100.00	13445.82	92633.03
	2006	80.43	69.48	10.95	104943.4	7029.83	7.75	77.64	11.54	85.13	6.60	1.15	87.01	21.31	1.65	97.70	100.00	13493.66	92651.36
	2007	80.75	69.74	11.01	111968.3	7329.37	6.80	65.16	85.61	85.61	5.82	0.98	87.41	21.06	1.61	97.60	100.00	13072.44	92669.92
	2008	81.08	70.01	11.07	108577.3	8303.75	7.34	79.37	9.15	88.48	6.49	0.85	87.80	20.81	1.61	97.60	100.00	12702.34	92693.05
	2009	81.27	70.17	11.10	101939.6	8126.26	8.11	69.32	9.30	86.58	7.03	1.09	88.18	20.61	1.59	97.60	100.00	12343.32	92707.20
	2010	81.44	70.31	11.13	104965.3	7964.46	7.68	67.71	9.58	85.86	6.59	1.09	88.55	20.47	1.63	97.60	100.00	13034.82	92736.64
	2011	81.56	70.43	11.12	105264.7	8333.58	7.34	71.70	10.55	85.28	6.26	1.08	88.91	20.33	1.52	97.60	100.00	12929.54	92778.19
	2012	81.81	70.65	11.16	102404.6	7550.71	7.18	66.33	10.97	83.46	5.99	1.19	89.25	20.23	1.57	97.60	100.00	12611.00	92813.28
	2013	81.98	70.80	11.19	103721.7	7980.83	7.10	66.16	10.80	83.68	5.94	1.16	89.57	20.16	1.55	97.60	100.00	-	92829.96
	2014	81.95	70.79	11.16	107152.9	8137.52	6.94	65.97	10.60	83.93	5.82	1.12	89.87	20.12	1.50	97.60	100.00	-	92872.94
	2015	82.03	70.88	11.15	107648.6	-	-	-	-	-	-	-	90.16	20.10	1.50	97.60	100.00	-	92909.28

	2016	82.12	70.96	11.16	108422.4	-	-	-	-	-	-	-	90.43	20.38	-	-	-	-	92925.19
	2017	81.66	69.70	11.96	-	-	-	-	-	-	-	-	-	-	-	-	-	-	-
Macedonia	1995	71.13	62.64	8.49	3057.00	198.55	8.39	100.00	40.45	59.55	5.00	3.39	59.59	13.14	1.98	-	99.30	11579.55	90664.37
	1996	71.42	62.90	8.52	3083.61	200.36	8.61	100.00	40.32	59.68	5.14	3.47	59.38	13.49	1.92	-	99.30	12809.73	90737.77
	1997	71.58	63.05	8.53	3112.12	176.32	9.09	100.00	38.86	61.14	5.55	3.53	59.17	13.81	1.86	-	99.30	11705.19	90841.27
	1998	71.65	63.11	8.54	3197.35	185.20	9.99	100.00	35.05	64.95	6.49	3.50	58.97	14.12	1.81	-	99.30	-	90943.26
	1999	71.89	63.31	8.58	3315.76	161.74	8.80	100.00	39.30	60.70	5.34	3.46	58.76	14.40	1.77	-	99.30	-	91040.15
	2000	72.18	63.57	8.61	3448.83	160.15	8.52	100.00	43.81	56.19	4.79	3.73	58.55	14.67	1.72	89.80	99.30	-	91121.58
	2001	72.23	63.59	8.64	3329.92	150.22	8.18	100.00	43.41	56.59	4.63	3.55	58.34	14.99	1.68	89.80	99.30	-	91203.46
	2002	72.45	63.77	8.68	3369.63	178.16	9.01	100.00	42.40	57.60	5.19	3.82	58.13	15.29	1.64	89.80	99.30	-	91309.37
	2003	72.53	63.82	8.71	3436.97	223.70	9.17	100.00	43.01	56.99	5.23	3.94	57.92	15.57	1.61	89.80	99.30	-	91417.15
	2004	72.67	63.92	8.75	3591.29	241.47	8.64	100.00	41.70	58.30	5.04	3.60	57.71	15.80	1.57	89.80	99.30	11251.00	91519.23
	2005	72.86	64.10	8.75	3755.04	243.73	7.95	100.00	39.88	60.12	4.78	3.17	57.53	16.00	1.53	90.00	99.30	11591.60	91629.15
	2006	73.07	64.28	8.80	3942.44	255.50	7.61	100.00	35.94	64.06	4.88	2.74	57.37	16.13	1.50	90.10	99.30	11559.34	91685.07
	2007	73.29	64.38	8.91	4192.95	280.97	6.90	100.00	64.44	64.44	4.45	2.45	57.24	16.22	1.48	90.30	99.30	12465.44	91719.73
	2008	73.50	64.52	8.98	4418.27	330.65	6.85	100.00	30.71	69.29	4.75	2.10	57.13	16.29	1.47	90.50	99.30	11742.82	91756.05
	2009	73.64	64.61	9.03	4398.77	308.76	6.74	100.00	33.84	66.16	4.46	2.28	57.05	16.36	1.46	90.60	99.30	11639.37	91774.53
	2010	73.96	64.93	9.04	4542.90	312.22	6.83	100.00	37.18	62.82	4.29	2.54	56.99	16.45	1.47	90.80	99.40	12257.54	91813.66
	2011	74.16	65.01	9.15	4645.52	336.33	6.61	100.00	35.37	64.63	4.27	2.34	56.96	16.63	1.47	91.00	99.40	12645.71	91835.55
	2012	74.18	65.02	9.16	4620.64	318.52	6.76	100.00	34.56	65.44	4.43	2.34	56.96	16.81	1.49	91.00	99.40	12992.25	91869.24
	2013	74.24	65.06	9.18	4751.90	316.14	6.07	100.00	30.78	69.22	4.20	1.87	56.98	17.03	1.50	90.90	99.40	-	91900.13
	2014	74.39	65.17	9.22	4920.22	353.93	6.48	100.00	36.67	63.33	4.10	2.37	57.03	17.33	1.51	90.90	99.40	-	91955.77
	2015	74.59	65.32	9.27	5104.78	-	-	-	-	-	-	-	57.10	17.73	1.52	90.90	99.40	-	92018.40
	2016	74.82	65.59	9.23	5222.83	-	-	-	-	-	-	-	57.20	18.29	-	-	-	-	92080.11
	2017	76.55	66.01	10.54	-	-	-	-	-	-	-	-	-	-	-	-	-	-	-
Madagascar	1995	56.85	49.83	7.03	416.94	9.55	4.07	76.80	49.57	35.46	1.44	2.62	25.80	5.80	5.98	9.90	34.00	46520.60	92044.30
	1996	57.19	50.13	7.06	412.81	13.00	4.52	76.76	44.22	42.39	1.91	2.60	26.06	5.81	5.91	10.00	34.80	43115.83	91958.45
	1997	57.47	50.38	7.09	414.77	11.83	4.78	76.82	42.31	44.93	2.15	2.63	26.32	5.80	5.84	10.00	35.60	42210.39	91876.49
	1998	57.79	50.67	7.12	417.67	11.40	4.51	76.65	43.31	43.50	1.96	2.55	26.59	5.79	5.75	10.10	36.40	142475.82	91795.63

	1999	58.22	51.03	7.20	423.58	11.09	4.55	76.57	42.22	44.86	2.04	2.51	26.85	5.76	5.65	10.20	37.20	90964.37	91724.72
	2000	58.45	51.22	7.23	430.11	12.51	5.08	76.57	37.79	50.65	2.57	2.51	27.12	5.72	5.55	10.30	38.00	84187.24	91659.66
	2001	58.98	51.68	7.30	442.14	14.43	5.17	76.25	34.84	54.30	2.81	2.36	27.39	5.72	5.45	10.40	38.80	131802.60	91664.29
	2002	59.13	51.83	7.30	374.49	13.91	5.29	76.66	36.94	51.81	2.74	2.55	27.66	5.70	5.34	10.50	39.60	84561.66	91686.51
	2003	59.32	52.01	7.31	398.90	15.28	4.81	76.66	40.66	46.96	2.26	2.55	27.94	5.67	5.24	10.60	40.40	163798.10	91714.60
	2004	59.60	52.25	7.35	407.52	12.01	4.89	78.10	39.16	49.86	2.44	2.45	28.21	5.62	5.14	10.60	41.20	87784.42	91748.24
	2005	60.07	52.66	7.41	413.87	13.86	5.03	80.17	38.39	52.12	2.62	2.41	28.81	5.56	5.05	10.80	42.10	159818.56	91777.76
	2006	60.40	52.98	7.42	422.14	14.98	5.11	80.08	35.94	55.12	2.82	2.29	29.43	5.52	4.96	10.90	43.10	107263.38	91813.99
	2007	60.65	53.20	7.45	435.72	18.89	4.98	80.04	54.97	54.97	2.74	2.24	30.04	5.48	4.87	11.00	44.00	134284.87	91848.95
	2008	60.75	53.32	7.43	453.64	21.24	4.50	79.75	36.84	53.80	2.42	2.08	30.67	5.42	4.78	11.10	45.00	94468.14	91892.31
	2009	60.75	53.34	7.41	423.31	19.61	4.70	80.12	39.07	51.23	2.41	2.29	31.30	5.35	4.69	11.20	45.90	117984.46	91938.98
	2010	60.83	53.45	7.38	412.73	20.15	4.86	86.01	39.92	53.58	2.61	2.26	31.93	5.28	4.60	11.40	46.90	117231.04	91989.84
	2011	61.10	53.69	7.41	407.33	19.33	4.24	82.07	34.87	57.51	2.44	1.80	32.56	5.24	4.52	11.50	47.80	117586.22	91822.63
	2012	61.35	53.94	7.41	408.34	15.38	3.46	80.46	38.18	52.55	1.82	1.64	33.20	5.18	4.44	11.60	48.70	117932.60	91688.47
	2013	61.65	54.23	7.42	406.37	19.20	4.15	80.29	30.73	61.73	2.56	1.59	33.83	5.13	4.37	11.70	49.70	-	91571.78
	2014	61.95	54.51	7.43	408.66	13.67	3.04	80.23	41.36	48.45	1.47	1.57	34.47	5.10	4.30	11.90	50.60	-	91447.40
	2015	62.30	54.84	7.46	410.19	-	-	-	-	-	-	-	35.11	5.10	4.24	12.00	51.50	-	91338.71
	2016	62.68	55.21	7.47	416.00	-	-	-	-	-	-	-	35.74	5.15	-	-	-	-	91232.83
	2017	63.51	55.45	8.06	-	-	-	-	-	-	-	-	-	-	-	-	-	-	-
Malawi	1995	45.97	40.11	5.86	364.61	7.18	5.05	46.58	29.36	36.96	1.87	3.18	13.26	5.93	6.42	31.90	52.70	8132.45	87869.58
	1996	45.39	39.67	5.72	383.52	10.52	4.61	46.10	33.47	27.38	1.26	3.35	13.63	5.91	6.36	32.40	54.70	7698.45	87861.63
	1997	44.99	39.36	5.64	387.63	13.37	5.15	43.69	30.12	31.07	1.60	3.55	14.00	5.87	6.31	32.90	56.80	7584.73	87868.38
	1998	44.54	38.99	5.55	390.58	9.13	5.51	39.74	26.16	34.16	1.88	3.63	14.38	5.82	6.26	33.40	58.80	17453.98	87866.03
	1999	44.40	38.87	5.53	390.09	10.64	6.52	38.35	24.96	34.90	2.27	4.24	14.52	5.76	6.20	33.80	60.60	13768.20	87875.28
	2000	44.72	39.11	5.61	384.68	9.45	6.07	40.47	21.95	45.78	2.78	3.29	14.61	5.68	6.15	34.30	62.50	15450.39	87877.71
	2001	45.14	39.50	5.64	355.55	7.58	5.07	42.38	23.88	43.66	2.22	2.86	14.70	5.69	6.10	34.70	64.40	12365.35	87873.41
	2002	45.54	39.86	5.68	352.03	10.90	4.82	41.74	16.53	60.39	2.91	1.91	14.79	5.67	6.05	35.20	66.20	14272.76	87892.72
	2003	46.21	40.44	5.76	362.37	12.73	6.35	42.33	11.80	72.13	4.58	1.77	14.88	5.65	6.00	35.60	68.10	20340.81	87893.91
	2004	46.81	40.96	5.85	371.79	16.54	7.82	35.08	9.42	73.14	5.72	2.10	14.96	5.62	5.96	36.10	70.00	15083.16	87899.58

	2005	47.61	41.62	5.99	373.23	17.73	8.20	33.86	8.82	73.96	6.07	2.14	15.05	5.59	5.90	36.50	71.80	20503.21	87917.73
	2006	48.45	42.41	6.04	379.44	21.38	8.99	28.30	8.80	68.89	6.19	2.80	15.14	5.62	5.83	37.00	73.70	15055.37	87887.80
	2007	49.46	43.32	6.15	403.49	25.17	9.31	22.28	47.42	47.42	4.42	4.90	15.24	5.64	5.73	37.40	75.50	17520.18	87852.36
	2008	50.57	44.29	6.28	421.23	32.90	10.70	24.11	10.37	56.99	6.10	4.60	15.33	5.66	5.61	37.90	77.40	17735.30	87819.24
	2009	51.80	45.36	6.45	442.56	35.73	10.18	28.65	11.24	60.78	6.19	3.99	15.43	5.68	5.47	38.30	79.20	15595.83	87788.81
	2010	53.03	46.40	6.63	458.87	36.72	10.05	27.53	10.16	63.11	6.34	3.71	15.54	5.70	5.31	38.80	81.00	21415.54	87772.29
	2011	54.47	47.70	6.76	466.96	43.14	11.67	20.16	8.18	59.40	6.93	4.74	15.66	5.73	5.15	39.20	82.90	21525.76	87679.09
	2012	56.04	49.10	6.95	461.89	32.56	12.06	23.34	10.24	56.12	6.77	5.29	15.80	5.76	4.99	39.70	84.70	21632.13	87594.32
	2013	57.42	50.30	7.12	471.84	26.29	10.96	23.34	11.00	52.85	5.79	5.17	15.94	5.78	4.86	40.10	86.50	-	87512.11
	2014	58.44	51.21	7.24	484.37	29.02	11.38	22.49	10.64	52.72	6.00	5.38	16.10	5.77	4.74	40.60	88.40	-	87453.80
	2015	59.37	52.01	7.36	483.63	-	-	-	-	-	-	-	16.27	5.74	4.65	41.00	90.20	-	87413.75
	2016	60.18	52.71	7.46	481.45	-	-	-	-	-	-	-	16.45	5.69	-	-	-	-	87380.43
	2017	63.21	55.03	8.18	-	-	-	-	-	-	-	-	-	-	-	-	-	-	-
Malaysia	1995	72.27	63.76	8.51	6275.12	127.04	2.96	74.98	32.64	56.46	1.67	1.29	55.69	6.27	3.31	88.80	92.20	252184.59	85211.80
	1996	72.43	63.91	8.52	6729.52	139.11	2.93	74.98	31.69	57.73	1.69	1.24	56.97	6.27	3.24	89.30	92.60	240005.00	85258.58
	1997	72.61	64.06	8.55	7040.79	125.14	2.72	74.98	33.60	55.18	1.50	1.22	58.24	6.24	3.15	89.80	93.00	245919.39	85344.55
	1998	72.73	64.18	8.56	6360.95	94.49	2.93	74.07	32.99	55.46	1.62	1.30	59.50	6.22	3.04	90.20	93.30	259140.17	85447.55
	1999	72.84	64.27	8.56	6589.60	104.83	3.03	74.28	33.08	55.47	1.68	1.35	60.74	6.21	2.91	90.70	93.70	160190.90	85521.45
	2000	73.01	64.45	8.57	7009.60	121.62	3.04	76.29	33.75	55.76	1.69	1.34	61.98	6.23	2.78	91.20	94.10	171393.52	85629.03
	2001	73.18	64.58	8.60	6893.28	131.78	3.40	73.76	30.55	58.59	1.99	1.41	62.92	6.38	2.66	91.60	94.40	176214.37	85730.64
	2002	73.29	64.67	8.62	7114.82	140.65	3.40	73.83	31.34	57.56	1.96	1.44	63.86	6.51	2.54	92.10	94.80	199983.48	85883.22
	2003	73.45	64.79	8.66	7377.31	175.21	3.95	75.06	30.22	59.74	2.36	1.59	64.78	6.61	2.44	92.50	95.10	195386.72	86022.35
	2004	73.66	64.95	8.72	7725.85	184.42	3.74	77.34	33.60	56.56	2.12	1.63	65.69	6.68	2.36	92.90	95.40	216283.73	86160.50
	2005	73.84	65.09	8.74	7983.89	183.16	3.29	78.29	38.08	51.36	1.69	1.60	66.59	6.76	2.29	93.40	95.70	246665.16	86330.07
	2006	73.98	65.22	8.76	8273.66	226.37	3.65	79.25	35.62	55.05	2.01	1.64	67.48	6.94	2.25	93.80	96.10	240375.43	86418.98
	2007	74.08	65.27	8.81	8889.68	261.07	3.61	78.43	54.41	54.41	1.96	1.64	68.36	7.10	2.22	94.20	96.40	250680.68	86481.94
	2008	74.19	65.34	8.84	9020.40	294.59	3.47	78.54	34.72	55.79	1.94	1.53	69.23	7.22	2.19	94.60	96.70	256755.87	86536.45
	2009	74.29	65.40	8.88	8635.12	290.62	3.97	76.33	31.45	58.79	2.34	1.64	70.08	7.29	2.17	95.00	97.00	277979.42	86591.10
	2010	74.41	65.50	8.91	9071.36	351.28	3.99	76.80	32.80	57.29	2.29	1.70	70.91	7.35	2.15	95.40	97.30	263676.11	86658.06

	2011	74.53	65.59	8.95	9377.19	393.67	3.89	77.84	35.08	54.94	2.14	1.75	71.74	7.53	2.13	95.70	97.70	271818.84	86601.19
	2012	74.67	65.70	8.97	9708.94	421.51	4.01	77.97	34.91	55.22	2.22	1.80	72.53	7.74	2.11	95.90	97.90	279098.38	86569.02
	2013	74.85	65.84	9.01	9981.15	427.05	4.02	79.95	36.11	54.83	2.20	1.81	73.28	7.98	2.09	96.00	98.00	-	86566.61
	2014	75.05	66.00	9.05	10398.23	455.83	4.17	78.75	35.30	55.18	2.30	1.87	74.01	8.22	2.07	96.00	98.20	-	86586.90
	2015	75.32	66.20	9.12	10745.05	-	-	-	-	-	-	-	74.71	8.48	2.06	96.00	98.20	-	86618.92
	2016	75.56	66.42	9.14	11031.82	-	-	-	-	-	-	-	75.37	8.77	-	-	-	-	86665.38
	2017	74.72	65.97	8.75	-	-	-	-	-	-	-	-	-	-	-	-	-	-	-
Maldives	1995	67.85	59.11	8.74	-	94.59	6.02	36.08	14.14	60.80	3.66	2.36	25.64	6.00	4.30	69.70	93.50	343.53	89805.01
	1996	68.80	59.97	8.83	-	110.51	6.37	47.70	20.73	56.55	3.60	2.77	26.01	6.13	3.98	71.70	93.80	344.68	89800.03
	1997	69.78	60.82	8.96	-	122.46	6.39	47.04	20.94	55.49	3.54	2.84	26.38	6.25	3.67	73.70	94.20	337.75	89809.67
	1998	70.65	61.59	9.06	-	150.12	7.51	49.43	18.85	61.88	4.65	2.86	26.76	6.38	3.40	75.60	94.50	351.80	89826.72
	1999	71.34	62.20	9.14	-	157.79	7.37	44.55	15.86	64.40	4.75	2.63	27.13	6.50	3.16	77.50	94.80	-	89880.83
	2000	72.12	62.90	9.22	-	178.05	8.00	52.70	18.96	64.02	5.12	2.88	27.71	6.62	2.94	79.40	95.20	-	89946.79
	2001	72.79	63.49	9.30	2209.74	180.93	5.84	56.43	20.67	63.38	3.70	2.14	28.86	6.65	2.77	81.50	95.50	-	89991.80
	2002	73.46	64.08	9.38	2289.71	181.37	5.78	59.42	22.73	61.75	3.57	2.21	30.04	6.67	2.63	83.50	95.90	-	90041.69
	2003	74.24	64.75	9.49	6245.22	208.58	5.90	63.43	22.79	64.07	3.78	2.12	31.25	6.69	2.51	85.40	96.20	-	90099.27
	2004	73.70	64.56	9.14	6452.25	236.05	5.89	67.96	23.22	65.84	3.88	2.01	32.49	6.69	2.42	87.30	96.60	-	90132.44
	2005	75.66	65.99	9.66	5447.97	349.73	9.52	71.78	18.07	74.82	7.13	2.40	33.75	6.67	2.34	89.10	96.90	-	90159.43
	2006	76.26	66.50	9.77	6622.93	350.47	7.37	71.82	20.78	71.06	5.24	2.13	35.03	6.65	2.29	90.90	97.20	-	90157.68
	2007	77.00	67.04	9.97	6806.77	367.53	6.64	72.66	68.56	68.56	4.55	2.09	36.30	6.61	2.26	92.60	97.50	-	90141.91
	2008	77.51	67.45	10.06	7184.78	613.81	9.30	74.81	18.32	75.51	7.03	2.28	37.55	6.55	2.24	94.20	97.80	-	90118.27
	2009	77.70	67.64	10.06	6702.44	611.23	9.22	78.18	21.11	73.00	6.73	2.49	38.78	6.48	2.24	95.80	98.10	-	90100.91
	2010	77.80	67.81	9.99	7052.25	556.11	7.93	79.43	26.60	66.51	5.27	2.66	39.98	6.39	2.23	97.30	98.30	-	90108.40
	2011	78.15	68.04	10.10	7453.31	589.64	8.11	81.67	25.22	69.12	5.61	2.51	41.16	6.22	2.22	98.00	98.60	-	90101.20
	2012	78.42	68.28	10.14	7482.17	673.56	9.16	82.83	24.05	70.96	6.50	2.66	42.30	6.05	2.21	98.00	98.60	-	90104.54
	2013	78.64	68.47	10.17	7863.55	860.15	11.16	83.10	21.59	74.02	8.26	2.90	43.42	5.89	2.19	98.00	98.60	-	90148.50
	2014	78.94	68.72	10.22	8271.54	1165.13	13.73	84.26	18.26	78.33	10.76	2.98	44.49	5.76	2.17	98.00	98.60	-	90195.44
	2015	79.16	68.92	10.24	8288.59	-	-	-	-	-	-	-	45.54	5.66	2.13	97.90	98.60	-	90255.94
	2016	79.32	69.13	10.20	8623.87	-	-	-	-	-	-	-	46.54	5.66	-	-	-	-	90329.32

	2017	81.45	71.12	10.33	-	-	-	-	-	-	-	-	-	-	-	-	-	-	-
Mali	1995	51.18	44.07	7.11	495.70	14.69	5.03	99.96	47.72	52.26	2.63	2.40	25.52	7.34	7.06	16.00	36.80	31483.01	87293.27
	1996	51.46	44.33	7.13	517.09	22.00	7.56	99.98	60.41	39.58	2.99	4.57	25.98	7.23	7.02	16.30	38.70	31338.26	87235.87
	1997	52.01	44.81	7.19	528.27	18.60	6.86	99.98	63.87	36.12	2.48	4.38	26.43	7.09	6.98	16.70	40.60	32233.05	87163.87
	1998	50.95	43.95	7.00	553.67	17.34	6.20	99.98	65.53	34.45	2.14	4.06	26.95	6.94	6.95	17.10	42.60	86412.25	87104.32
	1999	53.32	45.92	7.40	569.70	16.55	6.05	99.68	69.69	30.08	1.82	4.23	27.65	6.76	6.92	17.50	44.60	78429.02	87073.97
	2000	53.27	45.91	7.36	553.58	15.13	6.29	99.10	66.50	32.89	2.07	4.22	28.36	6.57	6.90	18.00	46.60	70241.63	87030.88
	2001	53.83	46.43	7.40	620.28	16.96	6.39	99.95	55.04	44.93	2.87	3.52	29.08	6.44	6.88	18.40	48.60	84085.49	86990.51
	2002	54.35	46.89	7.46	620.56	17.18	6.32	99.63	59.59	40.19	2.54	3.78	29.81	6.29	6.86	18.90	50.60	79829.52	86932.02
	2003	55.12	47.53	7.59	656.49	21.83	6.25	98.60	55.10	44.11	2.76	3.49	30.55	6.13	6.84	19.30	52.70	67302.87	86907.44
	2004	55.80	48.11	7.69	645.92	25.08	6.28	99.46	50.49	49.23	3.09	3.19	31.30	5.97	6.82	19.70	54.70	88391.55	86883.48
	2005	56.50	48.72	7.78	666.26	26.98	6.34	99.53	51.80	47.96	3.04	3.30	32.06	5.83	6.79	20.20	56.70	87079.09	86875.66
	2006	57.05	49.25	7.80	674.74	30.16	6.56	99.55	51.49	48.27	3.16	3.39	32.83	5.78	6.76	20.60	58.70	83577.39	86861.38
	2007	57.67	49.80	7.87	675.41	36.21	6.97	99.58	48.28	48.28	3.37	3.61	33.61	5.74	6.72	21.10	60.80	74744.40	86855.65
	2008	58.34	50.40	7.94	684.49	41.39	6.74	99.30	53.08	46.54	3.14	3.60	34.40	5.69	6.67	21.50	62.80	86890.41	86855.00
	2009	58.94	50.95	8.00	693.55	41.78	6.85	99.29	53.64	45.97	3.15	3.70	35.20	5.62	6.61	22.00	64.80	74104.27	86852.53
	2010	59.44	51.42	8.01	708.37	39.46	6.35	97.93	54.65	44.20	2.81	3.54	36.00	5.51	6.55	22.40	66.90	76827.88	86878.25
	2011	59.95	51.88	8.07	709.40	45.61	6.59	92.42	50.13	45.76	3.02	3.57	36.79	5.47	6.47	22.90	68.90	77134.18	86849.49
	2012	60.31	52.24	8.08	683.00	43.17	6.47	75.90	49.48	34.81	2.25	4.22	37.58	5.39	6.40	23.30	70.90	77437.93	86817.70
	2013	60.49	52.42	8.06	678.76	43.87	6.58	61.50	50.90	17.24	1.13	5.44	38.36	5.29	6.32	23.80	73.00	-	86804.27
	2014	60.88	52.79	8.09	705.79	47.77	6.86	61.77	47.66	22.85	1.57	5.29	39.14	5.20	6.23	24.20	75.00	-	86781.90
	2015	61.34	53.21	8.13	726.25	-	-	-	-	-	-	-	39.92	5.12	6.15	24.70	77.00	-	86796.58
	2016	61.84	53.69	8.15	745.87	-	-	-	-	-	-	-	40.68	5.09	-	-	-	-	86789.50
	2017	61.95	53.12	8.83	-	-	-	-	-	-	-	-	-	-	-	-	-	-	-
Malta	1995	77.12	67.50	9.63	14736.88	609.64	5.66	87.68	28.47	67.53	3.82	1.84	90.95	17.14	1.81	100.00	99.90	2710.72	91082.10
	1996	77.32	67.67	9.65	15193.46	664.18	6.07	88.05	28.53	67.60	4.11	1.97	91.18	17.31	2.03	100.00	99.90	2659.97	91143.09
	1997	77.39	67.74	9.65	15871.61	644.73	6.25	88.06	26.25	70.19	4.38	1.86	91.49	17.49	1.98	100.00	99.90	2835.19	91230.20
	1998	77.41	67.77	9.64	16577.03	691.34	6.45	89.88	27.91	68.94	4.44	2.00	91.79	17.67	1.88	100.00	100.00	2742.62	91348.04
	1999	77.37	67.74	9.63	17256.85	689.69	6.43	92.94	29.44	68.32	4.39	2.04	92.09	17.85	1.77	100.00	100.00	2781.48	91465.48

	2000	77.51	67.84	9.67	18306.67	686.93	6.83	92.91	28.41	69.42	4.74	2.09	92.37	18.02	1.70	100.00	100.00	2553.05	91605.81
	2001	77.82	68.10	9.72	18280.21	694.68	7.11	91.69	29.39	67.95	4.83	2.28	92.64	18.31	1.48	100.00	100.00	3049.25	91781.96
	2002	78.17	68.37	9.81	18685.66	851.07	7.86	90.79	28.07	69.09	5.43	2.43	92.91	18.62	1.45	100.00	100.00	2551.86	91954.58
	2003	78.62	68.71	9.90	19035.36	1092.51	8.06	90.49	28.24	68.79	5.54	2.51	93.16	18.96	1.48	100.00	100.00	2955.31	92095.73
	2004	78.94	68.95	9.98	18991.78	1280.23	8.46	82.74	27.46	66.81	5.65	2.81	93.41	19.32	1.40	100.00	100.00	3020.01	92226.78
	2005	79.27	69.20	10.07	19585.12	1407.70	8.83	89.64	28.78	67.89	6.00	2.84	93.65	19.70	1.38	100.00	100.00	3166.97	92369.23
	2006	79.51	69.40	10.12	19870.75	1495.37	8.93	91.47	29.56	67.69	6.05	2.89	93.88	20.03	1.36	100.00	100.00	3056.58	92453.48
	2007	79.66	69.50	10.15	20590.95	1628.07	8.36	90.52	65.60	65.60	5.48	2.87	94.09	20.37	1.35	100.00	100.00	3227.64	92519.60
	2008	79.81	69.61	10.20	21142.02	1805.92	8.15	89.09	32.75	63.24	5.16	3.00	94.30	20.84	1.43	100.00	100.00	-	92578.38
	2009	80.10	69.83	10.26	20466.56	1739.74	8.33	86.33	31.93	63.01	5.25	3.08	94.49	21.52	1.42	100.00	100.00	-	92651.45
	2010	80.32	69.99	10.33	21087.79	1753.89	8.30	88.54	32.85	62.89	5.22	3.08	94.67	22.45	1.36	100.00	100.00	-	92721.87
	2011	80.48	70.14	10.34	21296.26	2222.48	9.60	92.32	30.17	67.32	6.46	3.14	94.83	23.18	1.45	100.00	100.00	-	92763.13
	2012	80.72	70.34	10.38	21685.29	2216.60	9.95	89.45	29.90	66.58	6.63	3.33	94.99	24.11	1.43	100.00	100.00	-	92825.54
	2013	81.02	70.58	10.44	22471.64	2389.08	9.89	89.80	30.30	66.26	6.55	3.34	95.14	25.19	1.38	100.00	100.00	-	92899.70
	2014	81.25	70.77	10.48	24080.23	2470.60	9.75	93.59	28.86	69.16	6.74	3.01	95.28	26.30	1.42	100.00	100.00	-	92962.49
	2015	81.35	70.85	10.49	25511.47	-	-	-	-	-	-	-	95.41	27.32	1.42	100.00	100.00	-	93017.64
	2016	81.43	70.93	10.50	26606.31	-	-	-	-	-	-	-	95.53	28.41	-	-	-	-	93087.81
	2017	80.99	69.82	11.18	-	-	-	-	-	-	-	-	-	-	-	-	-	-	-
Marshall Islands	1995	64.36	56.10	8.27	3175.88	712.67	30.83	75.20	6.78	90.98	28.05	2.78	66.73	-	-	67.40	92.50	-	85334.47
	1996	64.26	56.00	8.26	2827.28	411.94	19.47	75.20	10.78	85.67	16.68	2.79	67.06	-	-	67.90	92.70	-	85271.75
	1997	64.00	55.77	8.23	2630.15	407.61	19.88	75.20	10.26	86.36	17.17	2.71	67.39	-	-	68.50	92.80	-	85228.22
	1998	63.82	55.61	8.21	2543.88	384.74	18.33	75.20	11.00	85.37	15.65	2.68	67.71	-	-	69.00	92.90	-	85209.06
	1999	63.62	55.43	8.20	2475.56	374.43	18.05	75.20	10.94	85.45	15.43	2.63	68.04	-	5.71	69.50	93.00	-	85242.62
	2000	63.21	55.04	8.17	2617.37	465.62	21.88	75.20	9.07	87.93	19.24	2.64	68.36	-	-	70.10	93.10	-	85325.12
	2001	63.33	55.15	8.17	2756.48	411.94	18.69	75.20	10.54	85.98	16.07	2.62	68.67	-	-	70.60	93.20	-	85419.23
	2002	63.26	55.10	8.16	2829.94	418.01	17.44	75.20	11.24	85.05	14.84	2.61	68.99	-	-	71.10	93.40	-	85563.85
	2003	63.20	55.04	8.15	2837.04	411.08	16.87	75.20	11.85	84.25	14.21	2.66	69.30	-	-	71.60	93.50	-	85706.03
	2004	63.13	54.98	8.15	2842.69	448.80	17.84	75.20	11.59	84.59	15.09	2.75	69.60	-	-	72.10	93.60	-	85845.40
	2005	63.06	54.92	8.15	2926.64	461.92	17.43	75.20	11.85	84.24	14.68	2.75	69.90	-	4.50	72.70	93.70	-	85969.12

	2006	62.97	54.86	8.11	2967.37	547.46	19.80	75.20	10.42	86.14	17.06	2.74	70.20	-	4.50	73.20	93.80	-	86053.05
	2007	63.14	55.00	8.14	3072.29	587.55	20.42	75.20	86.14	86.14	17.59	2.83	70.49	-	4.50	73.70	93.90	-	86120.88
	2008	63.29	55.12	8.17	3017.07	569.32	19.43	75.20	11.42	84.82	16.48	2.95	70.78	-	-	74.20	94.10	-	86211.57
	2009	63.46	55.25	8.21	2961.93	551.37	18.98	75.20	11.69	84.46	16.03	2.95	71.06	-	-	74.70	94.20	-	86293.21
	2010	63.64	55.39	8.25	3146.76	542.26	17.34	75.20	12.74	83.06	14.40	2.94	71.34	-	-	75.20	94.30	-	86391.85
	2011	63.83	55.54	8.28	3177.64	566.00	17.19	75.20	12.77	83.02	14.27	2.92	71.62	-	4.05	75.70	94.40	-	86497.32
	2012	64.02	55.70	8.32	3279.96	582.62	16.68	75.20	12.49	83.40	13.91	2.77	71.89	-	-	76.20	94.50	-	86653.60
	2013	64.21	55.85	8.36	3365.47	623.75	17.24	75.20	11.72	84.42	14.55	2.69	72.16	-	-	76.70	94.60	-	86784.13
	2014	64.38	55.98	8.40	3333.36	625.20	17.14	75.20	11.79	84.32	14.45	2.69	72.42	-	-	76.80	94.60	-	86933.61
	2015	64.59	56.14	8.45	3315.19	-	-	-	-	-	-	-	72.68	-	-	76.90	94.60	-	87092.57
	2016	64.92	56.40	8.51	3373.95	-	-	-	-	-	-	-	72.94	-	-	-	-	-	87354.68
	2017	64.54	56.41	8.13	-	-	-	-	-	-	-	-	-	-	-	-	-	-	-
Mauritania	1995	61.55	53.46	8.09	1038.26	28.18	4.65	94.70	49.26	47.99	2.23	2.42	45.25	6.05	5.77	18.20	35.70	8039.71	88529.46
	1996	61.69	53.60	8.08	1066.52	22.45	3.74	94.70	61.05	35.53	1.33	2.41	46.05	6.03	5.70	19.30	37.00	8251.86	88546.10
	1997	62.25	54.08	8.17	992.60	22.15	3.91	94.70	58.58	38.14	1.49	2.42	46.85	6.01	5.64	20.40	38.20	8692.80	88580.53
	1998	62.61	54.39	8.22	989.10	24.91	4.62	94.73	51.99	45.11	2.08	2.54	47.64	5.97	5.58	21.50	39.50	9423.20	88604.03
	1999	63.13	54.81	8.31	1033.14	28.50	5.33	94.70	43.16	54.42	2.90	2.43	48.44	5.93	5.51	22.60	40.80	9840.51	88636.06
	2000	63.57	55.19	8.38	998.11	25.08	5.26	94.72	44.78	52.72	2.77	2.48	49.24	5.89	5.45	23.70	42.00	11950.56	88676.17
	2001	63.90	55.47	8.43	988.47	20.09	4.34	94.71	53.67	43.33	1.88	2.46	50.04	5.87	5.39	24.80	43.30	11586.06	88684.24
	2002	64.41	55.90	8.51	966.47	24.38	5.31	94.71	43.67	53.89	2.86	2.45	50.83	5.84	5.34	26.00	44.60	10019.58	88710.70
	2003	64.86	56.28	8.58	995.19	23.79	4.53	94.68	49.12	48.12	2.18	2.35	51.61	5.80	5.28	27.20	45.80	10307.28	88733.52
	2004	65.21	56.59	8.62	1022.75	28.11	4.70	94.65	45.07	52.38	2.46	2.24	52.38	5.76	5.23	28.40	47.00	11027.64	88758.19
	2005	65.56	56.90	8.66	1083.20	29.13	4.21	94.62	48.88	48.34	2.03	2.17	53.13	5.72	5.18	29.50	48.30	11475.54	88787.94
	2006	66.27	57.53	8.75	1251.63	30.09	3.21	94.45	51.82	45.13	1.45	1.76	53.87	5.70	5.14	30.80	49.50	11509.78	88760.98
	2007	66.78	57.94	8.83	1251.15	35.66	3.54	94.51	46.72	46.72	1.65	1.88	54.59	5.68	5.10	32.00	50.70	13429.12	88736.59
	2008	67.26	58.37	8.88	1229.45	38.09	3.23	94.50	54.83	41.98	1.35	1.87	55.30	5.64	5.06	33.20	51.90	11721.45	88719.31
	2009	67.66	58.74	8.93	1182.38	39.61	3.79	93.07	48.84	47.52	1.80	1.99	56.00	5.61	5.02	34.40	53.10	11967.58	88718.49
	2010	68.11	59.16	8.95	1203.38	39.61	3.28	91.96	49.30	46.38	1.52	1.76	56.68	5.58	4.98	35.70	54.30	12951.01	88727.43
	2011	68.49	59.47	9.02	1223.34	39.92	2.87	85.74	47.52	44.57	1.28	1.59	57.35	5.55	4.94	36.90	55.50	13153.86	88758.72

	2012	68.85	59.79	9.06	1256.20	43.12	3.36	85.31	44.45	40.76	1.37	1.75	58.00	5.53	4.90	38.20	56.70	13343.16	88809.96
	2013	69.18	60.08	9.10	1293.56	47.47	3.63	86.55	40.49	47.49	1.73	1.70	58.64	5.50	4.85	39.50	57.90	-	88868.58
	2014	69.54	60.39	9.14	1326.16	48.84	3.77	86.99	43.85	49.59	1.87	1.90	59.26	5.48	4.79	39.70	57.90	-	88916.94
	2015	69.86	60.68	9.18	1306.65	-	-	-	-	-	-	-	59.86	5.47	4.74	40.00	57.90	-	88988.11
	2016	70.16	60.99	9.17	1296.01	-	-	-	-	-	-	-	60.45	5.49	-	-	-	-	89045.52
	2017	70.53	60.90	9.62	-	-	-	-	-	-	-	-	-	-	-	-	-	-	-
Mauritius	1995	70.27	61.63	8.64	4439.86	129.72	3.62	74.03	33.60	54.62	1.98	1.64	43.28	7.73	2.14	91.10	99.20	1834.36	88800.55
	1996	70.49	61.80	8.68	4640.25	136.63	3.53	73.81	33.96	53.99	1.90	1.62	43.16	8.02	2.12	91.10	99.20	-	88893.94
	1997	70.71	61.98	8.73	4843.14	128.05	3.53	73.75	33.81	54.16	1.91	1.62	43.04	8.30	2.04	91.10	99.20	-	89001.37
	1998	70.99	62.20	8.79	5083.42	127.95	3.58	73.47	33.31	54.66	1.95	1.62	42.92	8.56	1.97	91.10	99.20	-	89125.30
	1999	71.23	62.38	8.84	5150.25	127.26	3.49	73.03	33.59	54.01	1.88	1.60	42.79	8.80	2.05	91.20	99.20	-	89215.26
	2000	71.39	62.52	8.87	5560.23	146.29	3.78	74.64	35.78	52.06	1.97	1.81	42.67	9.02	1.99	91.40	99.20	-	89312.79
	2001	71.63	62.69	8.94	5658.45	146.88	3.87	74.02	35.64	51.85	2.00	1.86	42.46	9.14	1.91	91.50	99.30	-	89410.07
	2002	71.74	62.77	8.97	5738.06	168.02	4.24	74.81	36.30	51.48	2.18	2.06	42.25	9.28	1.94	91.60	99.30	-	89504.61
	2003	71.89	62.87	9.02	5905.00	192.40	4.15	80.78	38.15	52.77	2.19	1.96	42.04	9.41	1.87	91.80	99.40	-	89602.72
	2004	72.13	63.03	9.09	6205.25	225.43	4.29	80.80	36.58	54.73	2.35	1.94	41.83	9.51	1.92	91.90	99.40	-	89736.87
	2005	72.33	63.18	9.15	6245.20	229.79	4.47	84.58	42.66	49.56	2.21	2.25	41.62	9.59	1.88	92.00	99.50	-	89892.20
	2006	72.47	63.28	9.20	6746.89	240.08	4.38	86.61	46.11	46.76	2.05	2.33	41.41	9.79	1.77	92.20	99.50	-	90045.10
	2007	72.68	63.40	9.28	7100.86	268.02	4.24	88.01	44.16	44.16	1.87	2.37	41.20	9.97	1.74	92.30	99.60	-	90210.94
	2008	72.88	63.53	9.35	7456.37	358.47	4.60	90.71	55.57	38.73	1.78	2.82	41.00	10.17	1.67	92.40	99.60	-	90373.65
	2009	73.12	63.70	9.42	7683.12	353.09	4.97	91.08	51.32	43.66	2.17	2.80	40.79	10.45	1.59	92.60	99.70	-	90539.89
	2010	73.34	63.86	9.48	8000.38	411.58	5.29	91.26	46.67	48.86	2.58	2.70	40.58	10.83	1.57	92.70	99.70	-	90700.09
	2011	73.70	64.12	9.58	8313.27	451.21	5.02	91.29	46.57	48.98	2.46	2.56	40.37	11.34	1.55	92.90	99.80	-	90800.45
	2012	74.00	64.35	9.65	8580.09	432.48	4.76	91.38	47.33	48.20	2.29	2.46	40.16	11.93	1.54	93.00	99.80	-	90906.06
	2013	74.14	64.47	9.68	8848.89	455.53	4.82	91.35	46.53	49.06	2.37	2.46	39.98	12.59	1.44	93.20	99.90	-	90989.09
	2014	74.26	64.57	9.69	9163.63	482.45	4.81	91.32	46.42	49.17	2.36	2.44	39.81	13.32	1.43	93.20	99.90	-	91076.07
	2015	74.46	64.72	9.73	9468.94	-	-	-	-	-	-	-	39.67	14.09	1.36	93.10	99.90	-	91165.51
	2016	74.58	64.86	9.72	9822.01	-	-	-	-	-	-	-	39.55	14.78	-	-	-	-	91246.34
	2017	74.77	64.78	10.00	-	-	-	-	-	-	-	-	-	-	-	-	-	-	-

Mexico	1995	72.85	63.98	8.86	7307.18	172.44	5.06	97.04	56.15	42.13	2.13	2.93	73.37	7.85	3.02	70.70	85.60	515961.21	85455.85
	1996	73.22	64.31	8.91	7603.71	179.25	4.59	96.63	56.62	41.41	1.90	2.69	73.67	7.94	2.95	71.50	86.20	532804.43	85476.64
	1997	73.46	64.54	8.92	7999.86	218.92	4.72	95.98	53.07	44.71	2.11	2.61	73.93	8.04	2.88	72.30	86.80	539136.34	85521.49
	1998	73.71	64.76	8.95	8245.49	230.92	4.80	95.94	51.79	46.02	2.21	2.59	74.19	8.14	2.82	73.10	87.40	692323.18	85582.12
	1999	74.13	65.12	9.01	8340.56	271.48	5.01	95.86	50.03	47.81	2.39	2.61	74.44	8.23	2.77	73.90	88.00	553847.34	85657.63
	2000	74.61	65.53	9.08	8659.80	322.09	4.98	95.33	50.94	46.56	2.32	2.66	74.72	8.31	2.72	74.70	88.60	576489.52	85766.67
	2001	74.87	65.75	9.12	8494.84	366.31	5.35	95.00	52.43	44.81	2.40	2.95	75.05	8.37	2.67	75.50	89.20	547960.58	85891.68
	2002	74.89	65.81	9.08	8401.02	389.26	5.52	94.83	53.23	43.87	2.42	3.10	75.37	8.43	2.62	76.30	89.70	567679.66	86036.01
	2003	74.93	65.87	9.05	8416.90	412.98	6.02	95.08	54.96	42.20	2.54	3.48	75.68	8.48	2.58	77.10	90.30	601685.72	86190.27
	2004	75.22	66.12	9.09	8667.29	446.92	6.11	94.89	53.15	43.99	2.69	3.42	76.00	8.54	2.54	77.80	90.90	567790.06	86356.02
	2005	75.21	66.16	9.05	8808.56	490.72	6.04	94.37	53.48	43.33	2.62	3.43	76.31	8.63	2.50	78.60	91.40	641308.82	86539.88
	2006	75.46	66.36	9.10	9108.07	522.31	5.83	93.90	52.68	43.90	2.56	3.27	76.62	8.72	2.46	79.40	92.00	642137.77	86691.11
	2007	75.66	66.49	9.17	9253.32	565.53	5.92	93.33	44.67	44.67	2.64	3.27	76.92	8.83	2.42	80.10	92.50	646318.66	86858.50
	2008	75.44	66.32	9.11	9232.20	603.92	6.06	93.21	50.75	45.55	2.76	3.30	77.23	8.95	2.39	80.90	93.00	644167.96	87035.80
	2009	75.35	66.25	9.10	8657.84	526.08	6.60	92.58	49.12	46.94	3.10	3.50	77.53	9.06	2.36	81.60	93.60	648860.08	87218.17
	2010	75.44	66.34	9.10	8959.58	591.71	6.39	92.28	47.50	48.53	3.10	3.29	77.83	9.17	2.34	82.30	94.10	643375.43	87408.41
	2011	75.72	66.53	9.19	9183.33	615.10	6.04	91.47	45.05	50.75	3.07	2.98	78.12	9.28	2.32	83.00	94.60	652861.29	87499.65
	2012	75.88	66.65	9.23	9414.91	633.09	6.21	91.57	45.00	50.86	3.16	3.05	78.41	9.37	2.30	83.70	95.10	663424.95	87606.43
	2013	75.79	66.59	9.20	9409.97	673.81	6.30	91.22	44.00	51.74	3.26	3.04	78.69	9.48	2.27	84.40	95.60	-	87719.30
	2014	75.90	66.68	9.23	9492.99	677.19	6.30	91.22	44.00	51.77	3.26	3.04	78.97	9.62	2.24	85.10	96.10	-	87836.95
	2015	76.19	66.89	9.30	9615.31	-	-	-	-	-	-	-	79.25	9.81	2.22	85.20	96.10	-	87969.26
	2016	76.38	67.06	9.32	9707.91	-	-	-	-	-	-	-	79.52	10.05	-	-	-	-	88103.38
	2017	75.14	65.97	9.16	-	-	-	-	-	-	-	-	-	-	-	-	-	-	-
Moldova	1995	64.61	56.82	7.79	1086.94	30.04	9.06	76.41	27.35	64.21	5.82	3.24	46.29	13.99	1.90	71.70	84.20	16651.89	91219.73
	1996	65.34	57.45	7.90	1025.11	40.16	10.27	79.10	27.43	65.33	6.71	3.56	46.20	14.17	1.81	71.60	84.20	16914.86	91422.93
	1997	67.10	58.84	8.26	1045.85	42.22	9.45	78.93	28.20	64.27	6.08	3.38	46.10	14.22	1.72	71.90	84.40	15739.98	91635.11
	1998	67.71	59.34	8.37	977.82	35.72	7.69	83.11	36.37	56.23	4.32	3.36	46.00	14.19	1.62	72.10	84.60	14206.27	91842.74
	1999	67.54	59.24	8.30	946.37	18.89	5.89	86.25	43.72	49.31	2.90	2.98	45.90	14.13	1.53	72.40	84.80	11858.89	92056.05
	2000	67.62	59.35	8.28	968.29	23.45	6.65	83.34	42.88	48.55	3.23	3.42	45.80	14.04	1.44	72.70	85.10	10796.14	92256.67

	2001	68.19	59.84	8.35	1029.65	28.47	7.00	85.47	46.29	45.85	3.21	3.79	45.70	14.09	1.35	72.90	85.30	11371.92	92346.65
	2002	68.54	60.17	8.37	1112.54	38.01	8.31	80.63	41.44	48.61	4.04	4.27	45.61	14.13	1.29	73.20	85.50	11279.53	92423.57
	2003	68.80	60.42	8.38	1189.31	45.21	8.24	79.48	41.19	48.17	3.97	4.27	45.51	14.12	1.25	73.40	85.70	11996.30	92491.46
	2004	69.17	60.74	8.44	1280.49	61.25	8.49	79.56	40.35	49.28	4.18	4.30	45.41	14.04	1.22	73.70	86.00	12302.82	92532.60
	2005	68.55	60.30	8.25	1379.88	75.85	9.15	82.12	44.65	45.62	4.18	4.98	45.31	13.87	1.22	73.90	86.20	12762.86	92557.53
	2006	68.94	60.62	8.33	1450.13	100.81	10.63	82.92	46.10	44.41	4.72	5.91	45.21	13.95	1.23	74.20	86.40	12243.80	92548.88
	2007	69.22	60.79	8.42	1497.10	134.27	10.88	83.33	45.20	45.20	4.92	5.96	45.11	13.97	1.24	74.50	86.70	12163.98	92542.15
	2008	69.60	61.08	8.51	1616.95	193.76	11.41	85.41	45.07	47.22	5.39	6.02	45.01	13.95	1.26	74.70	86.90	11170.17	92529.13
	2009	69.89	61.30	8.59	1521.86	189.05	12.49	84.82	43.67	48.52	6.06	6.43	44.94	13.90	1.27	75.00	87.10	11008.69	92534.03
	2010	69.72	61.22	8.50	1631.54	196.83	12.08	82.75	44.25	46.53	5.62	6.46	44.89	13.84	1.27	75.30	87.40	11338.71	92547.81
	2011	71.49	62.55	8.95	1743.49	209.53	10.61	80.98	40.44	50.06	5.31	5.30	44.86	13.78	1.27	75.50	87.60	11433.49	92554.05
	2012	71.64	62.68	8.96	1731.51	242.63	11.87	83.55	44.72	46.47	5.52	6.36	44.86	13.62	1.27	75.80	87.90	11350.63	92557.98
	2013	72.53	63.36	9.17	1894.78	224.74	10.05	80.61	40.27	50.04	5.03	5.02	44.88	13.42	1.26	76.10	88.10	-	92557.97
	2014	72.23	63.14	9.09	1986.94	228.85	10.32	78.95	38.39	51.38	5.30	5.02	44.93	13.29	1.26	76.40	88.40	-	92567.60
	2015	72.12	63.07	9.05	1980.27	-	-	-	-	-	-	-	45.00	13.37	1.25	76.40	88.40	-	92575.14
	2016	72.25	63.28	8.97	2062.68	-	-	-	-	-	-	-	45.09	13.96	-	-	-	-	92591.86
	2017	72.90	63.00	9.89	-	-	-	-	-	-	-	-	-	-	-	-	-	-	-
Mongolia	1995	61.63	54.58	7.05	1459.11	19.91	3.15	66.18	11.60	82.47	2.60	0.55	56.81	6.60	2.74	46.00	53.70	59119.42	88656.56
	1996	61.46	54.48	6.98	1479.79	20.61	3.55	77.53	11.25	85.49	3.03	0.51	56.76	6.53	2.57	45.90	54.20	60763.25	88803.04
	1997	61.64	54.65	6.99	1524.86	18.44	3.65	63.14	10.10	84.00	3.06	0.58	56.71	6.44	2.43	46.50	54.60	60760.15	88942.81
	1998	61.91	54.89	7.02	1562.48	19.28	4.04	71.59	9.89	86.19	3.48	0.56	56.67	6.33	2.31	47.00	55.10	50019.83	89089.32
	1999	62.33	55.24	7.09	1596.51	17.97	4.04	64.18	9.88	84.61	3.42	0.62	56.62	6.18	2.22	47.50	55.60	30732.31	89228.99
	2000	62.70	55.53	7.17	1600.48	23.35	4.92	74.75	16.62	77.76	3.83	1.09	57.13	6.00	2.14	48.20	56.30	51756.46	89398.58
	2001	62.99	55.78	7.21	1632.52	28.58	5.45	84.53	24.10	71.49	3.90	1.56	58.22	5.94	2.10	49.20	57.20	27501.30	89493.28
	2002	63.18	55.96	7.22	1693.08	33.22	5.81	90.85	36.84	59.45	3.46	2.36	59.30	5.85	2.08	50.10	58.00	50488.32	89601.71
	2003	63.30	56.05	7.25	1792.87	39.93	6.18	95.31	50.30	47.22	2.92	3.26	60.38	5.75	2.08	51.00	58.80	39185.71	89701.44
	2004	63.46	56.18	7.28	1961.49	47.83	5.99	93.70	47.95	48.82	2.93	3.07	61.44	5.64	2.11	51.90	59.60	25435.53	89770.71
	2005	63.72	56.41	7.31	2079.11	50.80	5.09	94.03	48.09	48.85	2.48	2.60	62.49	5.53	2.17	52.70	60.20	27669.27	89821.87
	2006	64.24	56.83	7.41	2229.15	62.40	4.68	92.01	44.84	51.26	2.40	2.28	63.54	5.53	2.24	53.50	60.90	37790.42	89793.18

	2007	64.86	57.27	7.59	2425.68	82.71	5.06	92.59	54.04	54.04	2.74	2.33	64.56	5.53	2.33	54.30	61.50	55403.60	89734.39
	2008	65.41	57.69	7.72	2604.93	119.26	5.58	94.04	40.36	57.08	3.18	2.39	65.58	5.54	2.43	55.10	62.00	46513.80	89653.89
	2009	65.73	57.93	7.80	2533.18	90.83	5.29	94.21	44.91	52.33	2.77	2.52	66.58	5.53	2.54	55.80	62.50	50237.02	89564.37
	2010	65.99	58.17	7.82	2650.35	124.49	4.70	93.13	40.50	56.52	2.65	2.04	67.57	5.51	2.64	56.50	62.90	24860.38	89486.92
	2011	66.38	58.44	7.94	3053.61	168.06	4.45	92.47	38.59	58.27	2.60	1.86	68.54	5.55	2.72	57.20	63.30	-	89351.45
	2012	66.85	58.81	8.04	3365.57	184.71	4.22	93.23	43.29	53.57	2.26	1.96	69.47	5.59	2.77	57.90	63.70	-	89221.92
	2013	67.37	59.22	8.15	3685.75	184.79	4.21	93.29	44.30	52.51	2.21	2.00	70.37	5.63	2.81	58.50	64.00	-	89100.60
	2014	67.85	59.59	8.27	3901.87	195.33	4.73	93.35	41.63	55.40	2.62	2.11	71.22	5.68	2.81	59.10	64.20	-	89009.87
	2015	68.00	59.72	8.28	3923.63	-	-	-	-	-	-	-	72.04	5.77	2.79	59.70	64.40	-	88927.92
	2016	68.12	59.91	8.21	3906.12	-	-	-	-	-	-	-	72.82	5.91	-	-	-	-	88898.36
	2017	68.94	60.25	8.69	-	-	-	-	-	-	-	-	-	-	-	-	-	-	-
Montenegro	1995	73.95	64.69	9.26		139.21	7.42	100.00	29.97	70.03	5.20	2.22	53.37	14.67	1.98	-	-	-	91114.90
	1996	73.82	64.62	9.20		102.39	7.43	100.00	30.10	69.90	5.20	2.24	54.41	15.19	1.95	-	-	-	91172.73
	1997	73.73	64.55	9.18	5014.11	101.86	7.40	100.00	29.82	70.18	5.20	2.21	55.45	15.71	1.93	-	-	-	91259.35
	1998	73.64	64.49	9.15	5275.88	104.13	7.42	100.00	29.97	70.03	5.20	2.22	56.49	16.23	1.91	89.10	97.10	-	91347.40
	1999	73.62	64.47	9.15	4793.05	101.78	7.48	100.00	30.50	69.50	5.20	2.28	57.52	16.74	1.89	89.20	97.10	-	91439.83
	2000	73.82	64.61	9.20	4950.22	117.38	7.32	100.00	28.98	71.02	5.20	2.12	58.54	17.27	1.88	89.20	97.10	-	91555.07
	2001	74.00	64.78	9.23	4984.58	155.63	8.23	100.00	27.60	72.40	5.96	2.27	59.56	17.82	1.87	89.30	97.20	-	91584.12
	2002	74.28	64.99	9.29	5058.97	174.68	8.33	100.00	29.42	70.58	5.88	2.45	60.57	18.37	1.86	89.30	97.20	-	91613.62
	2003	74.52	65.19	9.34	5164.79	247.80	8.91	100.00	25.23	74.77	6.66	2.25	61.57	18.89	1.85	89.80	97.50	-	91660.78
	2004	74.75	65.36	9.39	5382.49	285.18	8.45	100.00	26.23	73.77	6.23	2.22	62.02	19.31	1.85	90.40	97.70	-	91701.48
	2005	75.00	65.57	9.43	5599.89	310.00	8.46	100.00	28.83	71.17	6.02	2.44	62.20	19.57	1.84	90.90	97.90	-	91743.42
	2006	75.27	65.78	9.49	6072.03	350.14	8.01	100.00	29.94	70.06	5.61	2.40	62.38	19.60	1.83	91.40	98.10	-	91728.82
	2007	75.63	66.01	9.62	6709.91	400.37	6.74	100.00	67.45	67.45	4.55	2.19	62.56	19.50	1.82	91.90	98.30	-	91707.28
	2008	76.00	66.28	9.71	6932.07	449.18	6.13	100.00	34.92	65.08	3.99	2.14	62.74	19.33	1.81	92.50	98.50	-	91688.63
	2009	76.21	66.44	9.78	6516.36	406.79	6.07	100.00	40.51	59.49	3.61	2.46	62.92	19.19	1.79	93.00	98.70	-	91687.26
	2010	76.36	66.58	9.78	6682.28	456.63	6.90	100.00	39.53	60.47	4.17	2.73	63.10	19.13	1.77	93.50	98.90	-	91714.03
	2011	76.43	66.62	9.81	6890.77	500.42	6.92	100.00	39.54	60.46	4.19	2.74	63.27	19.26	1.75	94.00	99.10	-	91729.24
	2012	76.54	66.72	9.82	6697.45	470.00	7.25	100.00	38.29	61.71	4.47	2.78	63.46	19.44	1.73	94.60	99.30	-	91752.08

	2013	76.67	66.82	9.85	6928.37	454.49	6.43	100.00	42.12	57.88	3.72	2.71	63.64	19.69	1.71	95.10	99.40	-	91772.76
	2014	76.81	66.92	9.88	7045.12	457.66	6.42	100.00	42.84	57.16	3.67	2.75	63.83	20.05	1.69	95.60	99.60	-	91798.19
	2015	76.93	67.01	9.92	7279.89	-	-	-	-	-	-	-	64.03	20.54	1.68	95.90	99.70	-	91826.13
	2016	77.04	67.13	9.91	7487.11	-	-	-	-	-	-	-	64.22	21.20	-	-	-	-	91861.69
	2017	76.49	65.98	10.51	-	-	-	-	-	-	-	-	-	-	-	-	-	-	-
Morocco	1995	69.01	58.92	10.09	1722.19	48.23	3.55	77.81	51.89	33.31	1.18	2.37	51.69	8.06	3.30	58.80	75.80	41902.28	90150.87
	1996	69.52	59.35	10.17	1907.79	51.82	3.49	77.47	52.07	32.78	1.14	2.34	52.02	8.22	3.16	59.80	76.40	42078.66	90238.50
	1997	69.82	59.61	10.22	1853.01	49.59	3.71	77.13	54.82	28.93	1.07	2.64	52.35	8.34	3.04	60.90	76.90	43941.60	90355.16
	1998	70.23	59.94	10.30	1962.03	55.38	3.92	76.79	53.66	30.12	1.18	2.74	52.68	8.43	2.93	61.90	77.30	45637.61	90478.51
	1999	70.54	60.18	10.36	1958.95	55.31	3.99	76.68	54.57	28.84	1.15	2.84	53.01	8.51	2.85	62.90	77.80	48246.34	90599.84
	2000	70.90	60.46	10.43	1972.30	53.46	4.18	76.58	54.05	29.42	1.23	2.95	53.34	8.59	2.78	64.00	78.30	50974.38	90745.32
	2001	71.22	60.73	10.48	2091.23	57.24	4.44	76.41	51.83	32.17	1.43	3.01	53.66	8.68	2.72	65.00	78.80	53556.27	90870.53
	2002	71.47	60.96	10.51	2130.73	72.64	5.31	81.74	60.51	25.97	1.38	3.93	53.99	8.77	2.68	66.00	79.30	53925.24	90986.93
	2003	71.73	61.18	10.55	2230.90	87.77	5.25	81.66	59.97	26.56	1.39	3.86	54.32	8.87	2.64	66.90	79.80	54270.64	91108.21
	2004	71.95	61.37	10.58	2310.34	98.70	5.22	81.38	59.07	27.41	1.43	3.79	54.64	8.96	2.61	67.90	80.30	65993.20	91248.24
	2005	72.24	61.63	10.61	2358.44	99.08	5.06	83.48	59.73	28.45	1.44	3.62	55.13	9.06	2.59	68.90	80.80	70022.09	91397.36
	2006	72.50	61.85	10.65	2507.67	111.94	5.23	86.25	58.00	32.76	1.71	3.52	55.64	9.13	2.57	69.90	81.40	71423.41	91514.16
	2007	72.76	62.02	10.73	2566.22	132.81	5.48	87.39	34.67	34.67	1.90	3.58	56.15	9.21	2.56	70.90	81.90	73113.44	91635.87
	2008	73.03	62.24	10.79	2686.23	153.51	5.41	87.26	56.77	34.94	1.89	3.52	56.67	9.28	2.56	71.90	82.50	72650.85	91751.93
	2009	73.31	62.45	10.86	2765.86	162.40	5.67	87.81	56.10	36.12	2.05	3.62	57.18	9.34	2.57	72.90	83.00	72152.21	91876.99
	2010	73.57	62.67	10.90	2834.20	165.63	5.86	88.34	57.16	35.29	2.07	3.79	57.68	9.39	2.58	73.80	83.50	76087.38	91993.81
	2011	73.83	62.87	10.96	2942.04	182.64	5.99	88.34	57.54	34.86	2.09	3.90	58.19	9.40	2.58	74.70	84.00	78381.40	92066.39
	2012	74.10	63.12	10.98	2987.32	178.72	6.15	88.34	56.99	35.49	2.18	3.97	58.70	9.40	2.58	75.60	84.60	80436.72	92150.27
	2013	74.35	63.34	11.01	3077.32	184.23	5.94	88.34	59.20	32.98	1.96	3.98	59.20	9.43	2.57	76.50	85.10	-	92225.76
	2014	74.57	63.54	11.03	3110.21	190.05	5.91	88.34	58.41	33.88	2.00	3.91	59.70	9.52	2.56	76.60	85.30	-	92295.61
	2015	74.79	63.73	11.06	3204.75	-	-	-	-	-	-	-	60.20	9.70	2.53	76.70	85.40	-	92377.64
	2016	75.00	63.97	11.03	3196.04	-	-	-	-	-	-	-	60.69	9.95	-	-	-	-	92453.75
	2017	73.97	63.15	10.83	-	-	-	-	-	-	-	-	-	-	-	-	-	-	-
Mozambiqu	1995	51.27	44.46	6.82	170.58	7.63	5.30	36.59	13.14	64.09	3.40	1.90	27.50	5.74	5.96	11.50	37.00	36851.04	89374.51

	1996	51.28	44.49	6.79	209.86	8.46	4.28	41.52	16.33	60.67	2.60	1.68	28.01	5.80	5.92	12.10	37.90	31666.11	89298.21
	1997	51.23	44.47	6.76	226.30	10.46	4.63	39.77	14.39	63.83	2.96	1.68	28.54	5.84	5.88	12.60	38.90	30429.45	89231.12
	1998	51.36	44.58	6.78	246.71	11.29	4.52	43.73	15.01	65.66	2.97	1.55	28.74	5.86	5.85	13.10	39.60	301746.74	89168.87
	1999	51.45	44.65	6.80	259.23	13.49	5.29	41.61	13.54	67.47	3.57	1.72	28.92	5.87	5.83	13.60	40.40	202613.16	89103.32
	2000	51.36	44.56	6.80	256.54	14.54	6.16	40.63	12.20	69.98	4.31	1.85	29.10	5.87	5.82	14.10	41.10	215278.01	89056.58
	2001	51.81	44.94	6.87	281.07	12.24	5.64	41.51	12.32	70.31	3.97	1.68	29.28	5.92	5.81	14.60	41.90	293796.22	88994.71
	2002	51.87	45.01	6.86	296.98	14.00	6.45	35.80	10.88	69.61	4.49	1.96	29.46	5.96	5.80	15.00	42.60	248706.42	88946.21
	2003	52.13	45.22	6.91	307.03	14.97	6.39	32.75	11.70	64.27	4.11	2.28	29.64	5.98	5.78	15.50	43.30	354834.81	88891.86
	2004	52.30	45.36	6.94	321.30	16.42	5.91	32.10	12.98	59.56	3.52	2.39	29.82	5.99	5.76	16.00	44.10	301972.73	88835.40
	2005	52.38	45.42	6.96	339.13	21.39	6.87	27.32	10.19	62.70	4.31	2.56	30.00	6.00	5.74	16.50	44.80	349872.74	88794.63
	2006	52.63	45.66	6.97	361.75	21.26	6.51	26.31	10.35	60.67	3.95	2.56	30.18	6.04	5.71	16.90	45.60	301392.99	88732.86
	2007	52.95	45.94	7.01	377.38	21.41	5.25	19.58	59.02	59.02	3.10	2.15	30.36	6.08	5.67	17.40	46.30	317678.29	88675.18
	2008	53.65	46.52	7.13	391.71	23.61	4.91	13.10	6.15	53.06	2.61	2.31	30.55	6.10	5.64	17.90	47.00	363780.57	88625.58
	2009	54.11	46.91	7.21	404.60	24.63	5.43	13.29	7.14	46.28	2.52	2.92	30.75	6.11	5.60	18.40	47.80	307176.21	88599.30
	2010	54.51	47.24	7.27	419.23	22.39	5.38	17.54	7.70	56.10	3.02	2.36	30.96	6.10	5.56	18.80	48.50	379823.55	88579.42
	2011	55.09	47.82	7.27	436.14	32.89	6.23	11.46	6.56	42.74	2.66	3.57	31.18	6.12	5.52	19.30	49.30	380073.24	88507.76
	2012	55.67	48.38	7.29	454.11	32.38	5.58	14.27	7.17	49.76	2.78	2.80	31.42	6.11	5.47	19.80	50.10	380308.29	88445.16
	2013	56.56	49.16	7.40	472.59	34.44	5.90	14.26	6.72	52.88	3.12	2.78	31.67	6.10	5.42	20.30	50.80	-	88408.36
	2014	57.95	50.32	7.63	493.25	42.00	6.98	21.75	9.48	56.44	3.94	3.04	31.93	6.08	5.37	20.40	50.90	-	88376.81
	2015	59.01	51.22	7.79	510.79	-	-	-	-	-	-	-	32.21	6.06	5.31	20.50	51.10	-	88365.12
	2016	60.02	52.08	7.94	515.39	-	-	-	-	-	-	-	32.51	6.07	-	-	-	-	88336.22
	2017	58.35	50.60	7.74	-	-	-	-	-	-	-	-	-	-	-	-	-	-	-
Myanmar	1995	59.54	52.28	7.26	246.06	2.41	2.14	99.92	80.98	18.96	0.41	1.73	25.53	7.72	3.04	54.40	60.90	943040.18	88975.61
	1996	59.88	52.60	7.27	258.60	2.39	2.12	99.39	83.68	15.81	0.33	1.78	25.77	7.75	2.99	55.90	62.00	704660.58	89014.71
	1997	60.11	52.83	7.28	269.65	1.83	1.80	99.59	85.07	14.58	0.26	1.54	26.04	7.76	2.97	57.40	63.20	697087.16	89060.51
	1998	60.43	53.12	7.31	281.73	1.90	1.59	100.00	89.41	10.59	0.17	1.42	26.33	7.75	2.95	58.90	64.30	306622.79	89110.99
	1999	60.75	53.40	7.34	308.59	2.45	1.56	100.00	89.01	10.99	0.17	1.38	26.64	7.71	2.93	60.40	65.40	337749.19	89139.26
	2000	61.10	53.71	7.39	346.77	3.19	1.84	100.00	85.79	14.21	0.26	1.58	26.97	7.65	2.91	61.90	66.60	185137.09	89179.44
	2001	61.51	54.09	7.42	381.70	2.47	1.80	99.95	87.46	12.50	0.22	1.58	27.32	7.65	2.89	63.40	67.70	186669.70	89063.20

	2002	61.96	54.49	7.47	422.96	2.82	2.05	98.79	83.68	15.29	0.31	1.74	27.69	7.63	2.87	64.90	68.90	226294.68	88947.63
	2003	62.48	54.94	7.54	476.61	3.71	1.97	98.61	86.40	12.38	0.24	1.72	28.08	7.59	2.83	66.40	70.00	187353.37	88832.52
	2004	63.01	55.40	7.61	536.21	4.57	1.97	98.64	85.26	13.56	0.27	1.70	28.50	7.54	2.78	67.90	71.20	379912.77	88716.82
	2005	63.58	55.89	7.69	603.83	4.74	1.83	99.62	90.64	9.01	0.16	1.67	28.93	7.49	2.72	69.40	72.30	223012.72	88626.51
	2006	64.26	56.51	7.75	677.70	5.36	1.78	95.33	81.58	14.43	0.26	1.52	29.39	7.50	2.66	70.80	73.50	199289.35	88589.09
	2007	64.93	57.07	7.85	753.95	6.94	1.68	95.58	11.77	11.77	0.20	1.49	29.87	7.51	2.59	72.30	74.60	349940.75	88555.03
	2008	59.66	52.64	7.02	826.09	9.83	1.87	95.72	85.12	11.07	0.21	1.67	30.36	7.51	2.53	73.80	75.80	189004.37	88543.59
	2009	66.20	58.14	8.06	907.36	13.20	2.05	92.70	82.16	11.37	0.23	1.81	30.88	7.52	2.47	75.20	76.90	266006.03	88559.93
	2010	66.82	58.68	8.14	987.74	15.25	1.92	90.75	76.61	15.58	0.30	1.62	31.41	7.53	2.41	76.60	78.10	325367.14	88586.29
	2011	67.35	59.14	8.21	1034.77	19.07	1.87	93.72	78.79	15.93	0.30	1.57	31.94	7.58	2.36	78.00	79.20	526919.84	88681.55
	2012	67.94	59.67	8.27	1101.21	23.67	2.22	93.72	59.09	36.95	0.82	1.40	32.47	7.62	2.32	79.40	80.30	528416.22	88805.40
	2013	68.51	60.18	8.33	1183.28	17.93	2.16	93.72	57.81	38.31	0.83	1.33	33.01	7.69	2.29	79.50	80.40	-	88939.09
	2014	69.04	60.65	8.39	1266.12	20.29	2.28	93.72	50.69	45.91	1.04	1.23	33.55	7.80	2.26	79.50	80.50	-	89098.94
	2015	69.50	61.06	8.44	1342.26	-	-	-	-	-	-	-	34.10	7.96	2.23	79.60	80.60	-	89261.90
	2016	70.02	61.56	8.46	1408.14	-	-	-	-	-	-	-	34.65	8.21	-	-	-	-	89423.56
	2017	68.50	59.90	8.61	-	-	-	-	-	-	-	-	-	-	-	-	-	-	-
Namibia	1995	59.86	52.40	7.45	3617.29	131.46	6.21	21.81	6.30	71.10	4.41	1.79	29.81	6.17	4.59	25.70	74.10	7245.02	87743.66
	1996	58.78	51.52	7.26	3620.89	134.66	6.58	21.83	6.06	72.23	4.75	1.83	30.32	6.14	4.47	26.00	75.00	6716.93	87751.73
	1997	57.64	50.57	7.07	3661.05	141.49	6.84	21.87	6.13	71.96	4.92	1.92	30.82	6.09	4.35	26.40	75.90	7024.66	87783.67
	1998	56.29	49.43	6.86	3673.84	128.38	6.84	21.85	6.02	72.44	4.95	1.88	31.34	6.02	4.23	26.70	76.80	22239.12	87808.17
	1999	54.97	48.30	6.67	3700.59	127.07	6.97	21.25	5.67	73.33	5.11	1.86	31.85	5.95	4.12	27.10	77.60	28386.79	87829.22
	2000	53.58	47.05	6.53	3746.71	125.91	6.11	18.16	5.64	68.92	4.21	1.90	32.37	5.87	4.02	27.40	78.50	33985.47	87864.23
	2001	52.85	46.45	6.39	3723.53	113.49	6.18	6.80	2.98	56.23	3.47	2.70	32.90	5.89	3.93	27.80	79.30	31359.31	87877.44
	2002	51.95	45.71	6.25	3845.06	105.89	6.17	9.35	3.93	58.00	3.58	2.59	33.77	5.89	3.84	28.30	80.30	29497.37	87895.24
	2003	51.45	45.26	6.18	3958.87	162.44	6.52	11.85	5.09	57.06	3.72	2.80	34.71	5.88	3.77	28.80	81.20	28634.23	87927.72
	2004	51.25	45.06	6.19	4394.41	213.50	6.47	7.43	3.75	49.55	3.21	3.26	35.67	5.85	3.71	29.30	82.10	34751.22	87968.51
	2005	51.61	45.25	6.35	4454.63	262.17	7.32	7.28	3.72	48.91	3.58	3.74	36.63	5.81	3.67	29.80	83.00	30934.05	88018.81
	2006	52.50	46.08	6.42	4715.11	279.66	7.20	5.73	3.24	43.46	3.13	4.07	37.61	5.84	3.64	30.30	83.90	37085.69	88063.31
	2007	54.06	47.41	6.65	4968.66	293.43	6.99	18.67	54.35	54.35	3.80	3.19	38.60	5.85	3.62	30.80	84.80	36561.10	88097.33

	2008	55.62	48.71	6.91	5036.25	285.98	7.15	17.91	8.14	54.53	3.90	3.25	39.60	5.86	3.61	31.30	85.60	28595.47	88135.42
	2009	57.21	50.01	7.20	4978.68	332.05	8.05	17.92	8.09	54.86	4.42	3.64	40.60	5.85	3.61	31.70	86.50	39971.70	88148.74
	2010	58.61	51.14	7.46	5191.58	405.64	7.89	17.91	7.48	58.21	4.59	3.30	41.62	5.84	3.61	32.20	87.30	37647.57	88166.44
	2011	60.02	52.36	7.66	5351.37	486.47	8.78	17.91	7.07	60.54	5.32	3.46	42.64	5.84	3.60	32.70	88.00	37855.65	88148.36
	2012	61.63	53.73	7.90	5502.26	467.90	8.24	17.91	7.35	58.94	4.85	3.38	43.67	5.84	3.59	33.10	88.80	38049.27	88122.80
	2013	62.59	54.57	8.03	5679.28	470.04	8.53	17.91	7.35	58.97	5.03	3.50	44.68	5.84	3.56	33.60	89.60	-	88097.52
	2014	63.55	55.40	8.16	5901.24	499.02	8.93	17.92	7.17	60.00	5.36	3.57	45.68	5.84	3.52	34.00	90.30	-	88080.18
	2015	64.22	55.97	8.24	6114.06	-	-	-	-	-	-	-	46.66	5.85	3.47	34.40	91.00	-	88052.62
	2016	64.77	56.47	8.30	6045.22	-	-	-	-	-	-	-	47.63	5.90	-	-	-	-	88041.36
	2017	66.49	57.09	9.40	-	-	-	-	-	-	-	-	-	-	-	-	-	-	-
Nepal	1995	60.59	52.46	8.12	403.05	10.49	5.31	94.62	69.56	26.48	1.41	3.91	10.90	6.62	4.72	13.10	71.60	26905.31	88298.69
	1996	61.33	53.09	8.24	414.70	10.86	5.42	94.31	69.56	26.25	1.42	4.00	11.37	6.69	4.60	14.80	72.70	27238.25	88294.00
	1997	62.07	53.71	8.37	426.17	11.87	5.50	93.83	68.73	26.75	1.47	4.03	11.86	6.73	4.47	16.60	73.80	27566.65	88282.98
	1998	62.58	54.13	8.44	430.07	13.18	6.61	92.93	57.02	38.64	2.55	4.06	12.36	6.77	4.33	18.30	75.00	28206.92	88273.62
	1999	63.17	54.63	8.55	440.38	12.63	5.87	92.36	64.63	30.02	1.76	4.11	12.89	6.80	4.18	20.00	76.00	41332.58	88275.87
	2000	63.72	55.10	8.63	459.12	12.21	5.43	91.21	68.49	24.91	1.35	4.08	13.43	6.82	4.03	21.70	77.10	31234.02	88287.59
	2001	64.33	55.60	8.73	472.77	13.08	5.36	91.08	63.99	29.74	1.59	3.77	13.97	6.96	3.88	23.40	78.20	29843.76	88390.04
	2002	64.40	55.68	8.72	465.55	13.48	5.60	88.65	62.68	29.29	1.64	3.96	14.26	7.12	3.72	25.00	79.20	28915.42	88516.83
	2003	65.24	56.39	8.85	476.46	14.26	5.48	89.45	64.53	27.86	1.53	3.96	14.56	7.29	3.58	26.60	80.20	29847.02	88641.35
	2004	65.55	56.67	8.88	491.70	16.84	5.82	75.36	54.78	27.31	1.59	4.23	14.87	7.48	3.43	28.30	81.20	30427.86	88789.83
	2005	66.02	57.10	8.91	502.24	18.53	5.72	72.95	52.74	27.70	1.59	4.14	15.18	7.69	3.29	29.90	82.20	30862.52	88949.12
	2006	66.47	57.53	8.94	513.13	19.85	5.70	72.78	46.66	35.90	2.04	3.65	15.50	7.84	3.15	31.50	83.20	30946.10	89029.98
	2007	66.91	57.89	9.02	525.08	24.56	5.84	82.87	39.09	39.09	2.28	3.56	15.82	7.99	3.01	33.10	84.10	31263.61	89104.49
	2008	67.34	58.27	9.06	551.64	28.58	6.44	83.63	48.21	42.35	2.73	3.71	16.15	8.14	2.87	34.70	85.10	32026.57	89170.23
	2009	67.79	58.67	9.12	570.93	30.70	6.41	81.26	45.45	44.08	2.82	3.58	16.48	8.29	2.74	36.30	86.10	32809.26	89228.40
	2010	68.27	59.13	9.14	592.18	38.96	6.43	80.33	44.52	44.58	2.87	3.56	16.82	8.44	2.61	37.90	87.00	32738.69	89292.61
	2011	68.74	59.49	9.25	605.63	45.72	6.73	79.86	41.38	48.18	3.24	3.49	17.17	8.48	2.49	39.50	88.00	33160.97	89239.97
	2012	69.23	59.91	9.31	627.18	38.41	5.89	78.97	45.91	41.86	2.47	3.43	17.52	8.51	2.38	41.10	88.90	40762.72	89196.80
	2013	69.66	60.28	9.38	645.25	37.18	5.69	79.86	48.68	39.04	2.22	3.47	17.88	8.57	2.29	42.60	89.80	-	89157.62

	2014	70.05	60.62	9.43	675.74	39.87	5.80	79.86	47.65	40.33	2.34	3.46	18.24	8.67	2.22	44.20	90.70	-	89146.11
	2015	69.70	60.33	9.37	690.08	-	-	-	-	-	-	-	18.62	8.84	2.16	45.80	91.60	-	89166.75
	2016	70.86	61.37	9.49	685.12	-	-	-	-	-	-	-	19.00	9.00	-	-	-	-	89201.87
	2017	70.92	61.10	9.82	-	-	-	-	-	-	-	-	-	-	-	-	-	-	-
Netherlands	1995	77.55	67.37	10.17	38438.55	2261.89	7.44	33.29	9.64	71.04	5.29	2.15	72.81	19.17	1.53	98.20	100.00	235053.01	90411.65
	1996	77.69	67.49	10.20	39626.29	2215.27	7.48	24.02	8.12	66.18	4.95	2.53	73.64	19.35	1.53	98.10	100.00	244204.43	90442.76
	1997	77.91	67.66	10.25	41117.96	1973.73	7.48	23.84	7.67	67.82	5.07	2.41	74.45	19.53	1.56	98.10	100.00	235836.24	90466.95
	1998	78.00	67.74	10.27	42714.51	2073.68	7.56	23.57	8.45	64.15	4.85	2.71	75.25	19.70	1.63	98.10	100.00	227061.48	90478.32
	1999	78.03	67.76	10.28	44574.69	2111.57	7.53	24.10	8.99	62.71	4.72	2.81	76.03	19.85	1.65	98.10	100.00	218722.67	90482.58
	2000	78.14	67.82	10.32	46133.18	1931.79	7.42	24.32	8.98	63.08	4.68	2.74	76.80	19.99	1.72	98.10	100.00	220319.90	90492.74
	2001	78.29	67.97	10.32	46758.88	2079.41	7.79	23.39	8.70	62.81	4.89	2.90	77.83	20.18	1.71	98.00	100.00	220252.52	90489.58
	2002	78.47	68.14	10.33	46509.52	2411.36	8.34	21.37	8.02	62.46	5.21	3.13	79.11	20.36	1.73	98.00	100.00	218276.48	90512.37
	2003	78.75	68.37	10.38	46422.02	2986.76	8.46	23.80	7.97	66.54	5.63	2.83	80.34	20.54	1.75	98.00	100.00	221354.46	90542.54
	2004	79.16	68.70	10.46	47200.42	3409.74	8.52	22.85	7.86	65.61	5.59	2.93	81.51	20.72	1.72	97.90	100.00	221355.35	90592.25
	2005	79.50	68.97	10.53	48107.59	3994.00	9.60	25.08	7.65	69.48	6.67	2.93	82.63	20.92	1.71	97.90	100.00	217083.63	90670.58
	2006	79.86	69.26	10.61	49720.39	4162.44	9.36	41.86	6.19	85.22	7.97	1.38	83.64	21.25	1.72	97.90	100.00	211767.90	90723.95
	2007	80.19	69.52	10.68	51447.26	4801.73	9.36	41.42	85.29	85.29	7.98	1.38	84.54	21.58	1.72	97.90	100.00	208848.04	90784.33
	2008	80.41	69.68	10.73	52118.09	5462.49	9.57	43.45	6.18	85.77	8.21	1.36	85.40	21.94	1.77	97.80	100.00	205061.31	90845.96
	2009	80.61	69.84	10.77	49897.23	5355.07	10.29	39.01	5.26	86.50	8.90	1.39	86.24	22.41	1.79	97.80	100.00	201884.49	90902.53
	2010	80.80	69.98	10.82	50338.25	5286.47	10.48	39.61	5.27	86.69	9.08	1.39	87.06	23.03	1.79	97.80	100.00	212418.46	90977.63
	2011	80.98	70.14	10.85	50937.55	5649.20	10.53	39.57	5.42	86.31	9.09	1.44	87.84	23.79	1.76	97.80	100.00	200143.54	91041.69
	2012	81.12	70.25	10.87	50212.96	5456.45	11.01	40.23	5.42	86.52	9.53	1.48	88.58	24.69	1.72	97.80	100.00	195873.76	91123.87
	2013	81.34	70.43	10.91	49969.85	5688.44	11.04	40.43	5.23	87.07	9.62	1.43	89.27	25.65	1.68	97.80	100.00	-	91210.89
	2014	81.52	70.58	10.94	50497.24	5693.86	10.90	40.18	5.22	87.00	9.48	1.42	89.91	26.58	1.71	97.70	100.00	-	91297.51
	2015	81.65	70.70	10.95	51410.49	-	-	-	-	-	-	-	90.50	27.43	1.71	97.70	100.00	-	91375.39
	2016	81.64	70.70	10.94	52304.30	-	-	-	-	-	-	-	91.03	28.24	-	-	-	-	91445.22
	2017	81.52	70.16	11.36	-	-	-	-	-	-	-	-	-	-	-	-	-	-	-
new Zealand	1995	76.98	67.19	9.80	26804.83	1202.81	6.95	70.74	16.15	77.17	5.36	1.59	85.33	17.66	1.99	-	100.00	69576.05	87452.04
	1996	77.18	67.35	9.83	27341.08	1291.82	6.89	69.87	16.29	76.68	5.28	1.61	85.42	17.79	1.96	-	100.00	70563.23	87445.00

	1997	77.57	67.65	9.92	27533.79	1307.64	7.10	68.83	15.63	77.29	5.49	1.61	85.49	17.87	1.96	-	100.00	73548.22	87493.99
	1998	77.96	67.95	10.01	27512.47	1129.68	7.51	70.83	16.26	77.03	5.79	1.73	85.55	17.93	1.89	-	100.00	69545.60	87562.67
	1999	78.21	68.15	10.06	28861.12	1157.64	7.40	70.70	15.92	77.48	5.73	1.67	85.61	17.99	1.97	-	100.00	71814.67	87625.70
	2000	78.55	68.41	10.14	29525.12	1056.08	7.47	69.88	15.36	78.02	5.83	1.64	85.68	18.05	1.98	-	100.00	74422.83	87735.51
	2001	78.71	68.56	10.15	30363.32	1058.84	7.58	72.04	16.99	76.42	5.79	1.79	85.76	18.04	1.97	-	100.00	76276.94	87677.39
	2002	79.00	68.80	10.20	31233.91	1261.34	7.89	72.65	16.06	77.90	6.15	1.74	85.90	18.04	1.89	-	100.00	77305.47	87667.15
	2003	79.27	69.02	10.25	32019.15	1623.88	7.71	72.65	15.74	78.34	6.04	1.67	86.00	18.06	1.93	-	100.00	80252.28	87659.41
	2004	79.52	69.22	10.29	32792.17	1992.66	7.89	69.26	14.11	79.63	6.28	1.61	86.03	18.10	1.98	-	100.00	80229.16	87644.02
	2005	79.89	69.51	10.37	33494.58	2307.10	8.25	69.24	14.07	79.68	6.57	1.68	86.05	18.17	1.97	-	100.00	82066.57	87662.67
	2006	80.04	69.66	10.38	34016.62	2315.65	8.65	69.43	13.84	80.07	6.92	1.72	86.08	18.39	2.01	-	100.00	83539.49	87645.86
	2007	80.23	69.82	10.41	34674.01	2713.54	8.35	65.06	82.39	82.39	6.88	1.47	86.10	18.61	2.18	-	100.00	81888.39	87599.60
	2008	80.37	69.94	10.43	33996.08	3318.77	10.70	69.64	11.66	83.25	8.91	1.79	86.13	18.87	2.19	-	100.00	81442.90	87553.83
	2009	80.63	70.15	10.48	33555.47	3145.24	11.21	66.12	11.11	83.20	9.32	1.88	86.15	19.21	2.13	-	100.00	76062.15	87568.43
	2010	80.93	70.38	10.55	33691.35	3742.56	11.20	64.88	10.97	83.10	9.31	1.89	86.17	19.64	2.17	-	100.00	76141.66	87598.69
	2011	80.99	70.42	10.57	34194.45	4251.40	11.24	64.34	10.87	83.11	9.34	1.90	86.18	20.07	2.09	-	100.00	75851.12	87712.27
	2012	81.25	70.63	10.62	34771.29	4470.86	11.53	63.80	10.97	82.80	9.55	1.98	86.20	20.59	2.10	-	100.00	78130.98	87875.83
	2013	81.48	70.81	10.68	35350.44	4661.80	11.17	62.57	10.90	82.58	9.23	1.95	86.22	21.19	2.01	-	100.00	-	88057.42
	2014	81.54	70.85	10.70	36006.02	4896.35	11.03	62.57	11.04	82.35	9.08	1.95	86.25	21.80	1.92	-	100.00	-	88252.26
	2015	81.49	70.81	10.68	36507.17	-	-	-	-	-	-	-	86.28	22.40	1.99	-	100.00	-	88447.97
	2016	81.48	70.80	10.68	36844.45	-	-	-	-	-	-	-	86.32	23.01	-	-	-	-	88648.27
	2017	81.64	69.05	12.59	-	-	-	-	-	-	-	-	-	-	-	-	-	-	-
Nicaragua	1995	73.78	64.98	8.80	1117.05	58.08	6.48	98.51	35.07	64.40	4.18	2.31	53.53	6.57	3.78	49.20	75.80	10769.14	86888.34
	1996	74.07	65.25	8.82	1165.54	58.73	6.41	98.83	40.31	59.21	3.79	2.61	53.78	6.63	3.62	50.20	76.40	10980.73	86865.10
	1997	74.44	65.57	8.87	1190.03	48.33	5.27	97.62	49.87	48.91	2.58	2.69	54.02	6.67	3.47	51.30	77.00	11325.79	86845.24
	1998	72.67	64.09	8.58	1213.17	53.56	5.63	98.04	49.74	49.27	2.77	2.85	54.26	6.71	3.33	52.30	77.60	22494.95	86856.68
	1999	75.26	66.27	9.00	1277.53	52.15	5.32	95.65	51.85	45.79	2.43	2.88	54.50	6.75	3.20	53.40	78.30	12417.60	86883.58
	2000	75.67	66.61	9.05	1309.53	54.78	5.39	91.63	42.65	53.46	2.88	2.51	54.74	6.79	3.08	54.40	78.90	14043.29	86925.09
	2001	75.93	66.84	9.09	1328.75	55.18	5.26	91.42	42.12	53.92	2.84	2.42	54.98	6.86	2.98	55.50	79.50	14462.64	86995.25
	2002	76.10	67.00	9.09	1320.39	57.72	5.71	95.46	45.43	52.41	3.00	2.72	55.22	6.93	2.89	56.50	80.10	14719.83	87066.97

	2003	76.19	67.10	9.09	1335.82	62.11	6.12	88.68	38.48	56.61	3.46	2.65	55.46	7.00	2.81	57.50	80.70	16965.37	87156.50
	2004	76.35	67.24	9.10	1388.54	66.83	6.13	91.99	41.09	55.33	3.39	2.74	55.70	7.07	2.74	58.60	81.30	14793.13	87265.78
	2005	76.64	67.51	9.12	1429.26	71.82	6.11	91.63	39.89	56.46	3.45	2.66	55.93	7.15	2.68	59.60	81.90	16831.87	87375.59
	2006	76.92	67.77	9.15	1464.35	78.84	6.33	93.04	42.01	54.85	3.47	2.86	56.18	7.24	2.62	60.60	82.50	15048.35	87465.07
	2007	77.10	67.88	9.21	1518.65	93.38	6.91	93.04	54.85	54.85	3.79	3.12	56.44	7.34	2.57	61.60	83.10	15276.86	87551.12
	2008	77.35	68.09	9.25	1550.50	105.99	6.98	93.05	42.01	54.85	3.83	3.15	56.70	7.44	2.52	62.70	83.70	15791.95	87672.52
	2009	77.42	68.15	9.26	1480.38	100.83	6.82	90.63	33.65	62.88	4.29	2.53	56.97	7.52	2.48	63.70	84.30	15861.97	87778.40
	2010	77.43	68.20	9.23	1526.50	100.22	6.58	90.63	31.55	65.18	4.29	2.29	57.26	7.58	2.43	64.70	85.00	15794.35	87903.76
	2011	77.55	68.27	9.28	1603.33	107.40	6.39	90.34	32.67	63.84	4.08	2.31	57.54	7.60	2.38	65.70	85.60	16068.64	87984.67
	2012	77.60	68.32	9.28	1687.36	143.13	8.04	85.62	40.26	52.98	4.26	3.78	57.84	7.60	2.34	66.80	86.20	16323.04	88088.45
	2013	77.68	68.38	9.29	1750.06	153.83	8.43	86.51	41.14	52.44	4.42	4.01	58.15	7.62	2.30	67.80	86.80	-	88190.25
	2014	77.81	68.50	9.32	1812.99	177.46	9.04	86.04	37.54	56.37	5.10	3.94	58.46	7.69	2.26	67.80	86.90	-	88296.00
	2015	78.03	68.67	9.35	1879.71	-	-	-	-	-	-	-	58.78	7.84	2.23	67.90	87.00	-	88415.29
	2016	78.27	68.92	9.35	1946.37	-	-	-	-	-	-	-	59.11	8.04	-	-	-	-	88523.45
	2017	78.80	68.80	10.00	-	-	-	-	-	-	-	-	-	-	-	-	-	-	-
Niger	1995	48.99	42.68	6.30	335.48	12.47	6.65	88.30	68.80	22.09	1.47	5.18	15.77	4.89	7.73	5.20	38.70	8185.43	90547.06
	1996	49.84	43.41	6.43	334.85	12.21	6.30	88.20	70.22	20.39	1.28	5.02	15.86	4.90	7.72	5.50	39.70	8402.84	90540.81
	1997	50.43	43.94	6.50	331.90	11.58	6.73	88.22	66.12	25.05	1.68	5.04	15.94	4.91	7.71	5.80	40.70	8595.59	90536.75
	1998	51.03	44.46	6.57	353.42	12.88	6.79	88.25	66.23	24.95	1.69	5.10	16.02	4.92	7.70	6.00	41.70	10401.14	90548.82
	1999	51.64	44.99	6.65	338.85	12.05	6.82	88.26	66.27	24.92	1.70	5.12	16.10	4.92	7.69	6.30	42.70	11321.46	90555.29
	2000	52.17	45.44	6.73	322.15	8.91	6.01	87.83	65.57	25.35	1.52	4.48	16.19	4.93	7.68	6.60	43.70	11124.28	90573.97
	2001	52.68	45.93	6.76	332.76	10.92	7.01	88.28	64.77	26.63	1.87	5.14	16.27	4.95	7.67	6.80	44.70	10982.17	90566.62
	2002	53.48	46.62	6.86	330.55	11.20	6.55	87.95	62.32	29.15	1.91	4.64	16.37	4.96	7.66	7.10	45.70	9326.63	90579.11
	2003	54.14	47.20	6.93	335.67	13.13	6.23	87.88	64.19	26.96	1.68	4.55	16.47	4.97	7.64	7.40	46.70	9570.27	90592.08
	2004	54.89	47.86	7.03	323.97	14.73	6.61	87.08	62.76	27.93	1.85	4.76	16.59	4.99	7.63	7.70	47.70	9191.39	90609.34
	2005	55.58	48.48	7.11	326.33	17.86	7.15	92.42	53.92	41.66	2.98	4.17	16.72	5.01	7.61	7.90	48.70	10258.53	90622.71
	2006	56.19	49.06	7.14	332.71	19.27	7.39	90.53	51.65	42.94	3.18	4.22	16.86	5.05	7.59	8.20	49.80	10176.13	90600.97
	2007	56.86	49.65	7.21	330.63	20.46	6.94	90.58	38.37	38.37	2.66	4.28	17.02	5.10	7.57	8.50	50.80	10970.29	90588.54
	2008	57.55	50.27	7.28	349.00	24.21	6.76	91.45	54.97	39.89	2.70	4.06	17.19	5.13	7.54	8.80	51.90	10491.43	90576.42

	2009	58.11	50.78	7.33	333.69	24.03	6.98	90.61	56.20	37.98	2.65	4.33	17.37	5.17	7.52	9.10	52.90	10530.93	90571.70
	2010	58.75	51.36	7.39	348.15	22.33	6.36	90.49	59.73	33.99	2.16	4.20	17.56	5.19	7.49	9.50	54.00	11219.51	90575.78
	2011	59.26	51.82	7.43	342.86	25.20	6.66	91.21	57.61	36.83	2.45	4.21	17.77	5.25	7.46	9.80	55.10	11342.12	90579.33
	2012	59.76	52.28	7.48	368.94	23.16	6.11	94.84	60.50	36.20	2.21	3.90	17.99	5.29	7.42	10.10	56.20	11460.92	90578.36
	2013	60.27	52.75	7.52	373.74	20.78	5.09	76.67	39.68	48.25	2.46	2.63	18.22	5.33	7.38	10.50	57.30	-	90585.19
	2014	60.77	53.20	7.57	386.73	24.40	5.82	76.67	34.33	55.22	3.21	2.61	18.47	5.36	7.34	10.80	58.10	-	90585.91
	2015	61.20	53.58	7.61	386.90	-	-	-	-	-	-	-	18.73	5.38	7.29	10.90	58.20	-	90587.92
	2016	61.68	54.02	7.66	391.13	-	-	-	-	-	-	-	19.01	5.40	-	-	-	-	90593.48
	2017	62.37	54.20	8.16	-	-	-	-	-	-	-	-	-	-	-	-	-	-	-
Nigeria	1995	54.68	47.16	7.52	1242.74	37.68	2.77	94.55	72.09	23.76	0.66	2.11	32.21	5.43	6.26	36.00	46.00	184046.20	91382.98
	1996	54.45	46.98	7.47	1272.73	53.95	2.92	94.74	75.23	20.59	0.60	2.32	32.73	5.43	6.22	35.60	47.20	203140.48	91375.81
	1997	54.65	47.16	7.49	1276.24	54.64	2.92	94.59	71.31	24.61	0.72	2.20	33.25	5.41	6.19	35.20	48.30	196351.83	91364.78
	1998	54.61	47.12	7.48	1278.65	61.42	3.47	95.00	70.17	26.14	0.91	2.56	33.77	5.37	6.16	34.80	49.50	332361.06	91365.63
	1999	54.77	47.24	7.53	1253.05	16.26	3.38	94.76	67.16	29.12	0.98	2.39	34.30	5.31	6.13	34.40	50.60	353503.93	91362.96
	2000	54.89	47.32	7.57	1287.06	17.22	2.84	92.65	61.65	33.46	0.95	1.89	34.84	5.25	6.11	34.00	51.80	314978.54	91359.60
	2001	55.12	47.55	7.57	1310.51	18.28	3.25	91.39	62.74	31.35	1.02	2.23	35.67	5.26	6.08	33.70	53.00	319145.79	91311.25
	2002	55.13	47.59	7.54	1326.24	17.89	2.43	90.43	67.30	25.58	0.62	1.81	36.51	5.24	6.06	33.30	54.20	295068.36	91271.57
	2003	55.57	47.98	7.59	1426.90	38.50	4.05	96.22	74.66	22.40	0.91	3.14	37.36	5.21	6.04	32.90	55.40	314942.19	91235.53
	2004	56.11	48.44	7.67	1860.06	44.94	4.33	95.34	64.17	32.69	1.41	2.91	38.21	5.17	6.01	32.60	56.60	288145.57	91202.06
	2005	56.84	49.06	7.78	1875.03	53.09	4.11	95.80	67.86	29.17	1.20	2.91	39.07	5.11	5.99	32.20	57.80	374421.70	91171.72
	2006	57.47	49.62	7.85	1976.71	61.01	3.66	95.62	64.12	32.94	1.20	2.45	39.94	5.15	5.96	31.90	59.00	318579.09	91116.97
	2007	58.20	50.25	7.95	2056.84	81.37	4.47	95.83	32.92	32.92	1.47	3.00	40.82	5.18	5.93	31.50	60.10	335201.39	91058.15
	2008	59.00	50.92	8.08	2128.67	88.52	4.00	95.66	60.48	36.77	1.47	2.53	41.70	5.18	5.90	31.20	61.20	316058.63	91022.84
	2009	59.74	51.55	8.19	2216.50	74.36	4.24	95.80	65.83	31.28	1.32	2.91	42.59	5.17	5.87	30.80	62.30	273156.36	90970.45
	2010	60.52	52.22	8.29	2327.32	80.34	3.47	95.67	70.62	26.18	0.91	2.56	43.48	5.14	5.84	30.50	63.40	292211.74	90944.70
	2011	61.27	52.89	8.38	2376.64	93.23	3.69	95.66	65.78	31.23	1.15	2.54	44.36	5.18	5.80	30.20	64.50	296799.95	90896.74
	2012	61.93	53.49	8.44	2412.86	90.39	3.30	95.53	65.61	31.32	1.03	2.27	45.23	5.20	5.76	29.90	65.50	301010.13	90858.42
	2013	62.50	54.02	8.49	2475.95	110.37	3.70	95.77	72.95	23.83	0.88	2.82	46.09	5.20	5.71	29.60	66.60	-	90828.56
	2014	62.97	54.46	8.52	2563.09	117.52	3.67	95.74	71.67	25.15	0.92	2.75	46.94	5.18	5.65	29.30	67.60	-	90803.94

	2015	64.02	55.37	8.65	2562.52	-	-	-	-	-	-	-	47.78	5.15	5.59	29.00	68.50	-	90793.98
	2016	65.02	56.28	8.74	2455.92	-	-	-	-	-	-	-	48.60	5.17	-	-	-	-	90785.84
	2017	64.35	55.10	9.25	-	-	-	-	-	-	-	-	-	-	-	-	-	-	-
Northern Mariana Islands	1995	75.22	65.89	9.33	-	-	-	-	-	-	-	-	89.56	-	-	71.20	94.80	6.56	83754.75
	1996	75.42	66.04	9.38	-	-	-	-	-	-	-	-	89.66	-	-	71.70	94.90	6.93	83695.17
	1997	75.60	66.17	9.43	-	-	-	-	-	-	-	-	89.78	-	-	72.20	95.10	7.32	83679.67
	1998	75.74	66.27	9.47	-	-	-	-	-	-	-	-	89.91	-	-	72.70	95.30	7.70	83698.12
	1999	75.91	66.40	9.51	-	-	-	-	-	-	-	-	90.04	-	-	73.20	95.40	8.09	83732.79
	2000	76.14	66.58	9.56	-	-	-	-	-	-	-	-	90.12	-	-	73.70	95.60	8.47	83798.83
	2001	76.14	66.55	9.58	-	-	-	-	-	-	-	-	90.05	-	-	74.20	95.80	8.85	83895.84
	2002	76.17	66.57	9.60	21177.70	-	-	-	-	-	-	-	89.99	-	-	74.70	95.90	9.22	84000.37
	2003	76.25	66.62	9.63	21687.13	-	-	-	-	-	-	-	89.93	-	-	75.20	96.10	9.59	84115.41
	2004	76.26	66.61	9.65	21573.63	-	-	-	-	-	-	-	89.86	-	-	75.70	96.20	9.96	84220.70
	2005	76.15	66.54	9.60	19408.28	-	-	-	-	-	-	-	89.80	-	-	76.20	96.40	10.33	84356.99
	2006	76.18	66.58	9.60	18673.13	-	-	-	-	-	-	-	89.74	-	-	76.70	96.60	10.65	84540.00
	2007	76.12	66.49	9.63	18056.32	-	-	-	-	-	-	-	89.67	-	-	77.20	96.70	11.00	84745.84
	2008	76.06	66.43	9.63	16639.58	-	-	-	-	-	-	-	89.61	-	-	77.70	96.90	11.38	84947.41
	2009	75.91	66.31	9.60	14155.54	-	-	-	-	-	-	-	89.54	-	-	78.20	97.10	11.80	85144.59
	2010	75.90	66.32	9.58	14681.02	-	-	-	-	-	-	-	89.47	-	-	78.70	97.20	12.24	85377.37
	2011	75.75	66.16	9.59	13712.46	-	-	-	-	-	-	-	89.41	-	-	79.20	97.40	12.37	85586.11
	2012	75.63	66.05	9.58	13803.64	-	-	-	-	-	-	-	89.36	-	-	79.70	97.50	12.49	85813.45
	2013	75.50	65.93	9.57	14089.31	-	-	-	-	-	-	-	89.31	-	-	79.70	97.50	-	86032.73
	2014	75.40	65.83	9.57	14468.96	-	-	-	-	-	-	-	89.27	-	-	79.70	97.50	-	86251.58
	2015	75.36	65.77	9.59	14919.64	-	-	-	-	-	-	-	89.24	-	-	79.70	97.50	-	86475.20
	2016	75.47	65.86	9.61	19115.38	-	-	-	-	-	-	-	89.21	-	-	-	-	-	86693.87
	2017	76.20	66.13	10.07	-	-	-	-	-	-	-	-	-	-	-	-	-	-	-
Norway	1995	77.94	67.99	9.95	70457.69	2698.13	7.72	96.50	15.22	84.23	6.50	1.22	73.76	24.81	1.87	98.10	100.00	70569.10	89493.88
	1996	78.21	68.23	9.98	73626.16	2868.62	7.67	96.48	15.27	84.17	6.45	1.21	74.04	24.63	1.89	98.10	100.00	72016.75	89429.29
	1997	78.34	68.35	9.99	77097.89	3025.91	8.24	95.41	17.83	81.31	6.70	1.54	74.31	24.40	1.86	98.10	100.00	74552.49	89391.12

	1998	78.42	68.42	9.99	78651.55	3165.81	9.07	95.35	16.98	82.19	7.45	1.62	74.38	24.14	1.81	98.10	100.00	74639.79	89377.66
	1999	78.53	68.52	10.01	79687.51	3337.84	9.14	95.37	16.63	82.56	7.55	1.59	75.09	23.86	1.85	98.10	100.00	76295.63	89378.73
	2000	78.74	68.68	10.06	81709.66	3164.81	8.27	95.48	16.72	82.49	6.82	1.45	76.08	23.56	1.85	98.10	100.00	71217.90	89405.87
	2001	78.94	68.86	10.07	82992.53	3340.80	8.65	95.67	15.73	83.56	7.22	1.42	76.56	23.34	1.78	98.10	100.00	71491.23	89460.87
	2002	79.14	69.05	10.09	83732.94	4153.81	9.62	95.36	15.76	83.48	8.03	1.59	77.00	23.11	1.75	98.10	100.00	69689.53	89519.69
	2003	79.50	69.34	10.16	84008.96	4951.23	9.85	95.33	15.53	83.71	8.25	1.61	77.23	22.89	1.80	98.10	100.00	73172.63	89593.53
	2004	79.87	69.64	10.22	86820.34	5452.96	9.44	95.31	15.67	83.56	7.89	1.55	77.28	22.71	1.83	98.10	100.00	73534.99	89669.53
	2005	80.17	69.87	10.29	88494.36	5961.32	8.89	95.15	15.66	83.54	7.43	1.46	77.49	22.59	1.84	98.10	100.00	71578.06	89759.97
	2006	80.40	70.07	10.34	89887.02	6276.09	8.43	95.23	15.45	83.78	7.06	1.37	77.89	22.45	1.90	98.10	100.00	71355.50	89791.04
	2007	80.56	70.20	10.36	91617.28	7351.79	8.58	94.79	84.10	84.10	7.22	1.37	78.23	22.33	1.90	98.10	100.00	72404.47	89825.44
	2008	80.73	70.34	10.39	90917.54	8193.61	8.40	95.46	14.84	84.40	7.09	1.31	78.53	22.27	1.96	98.10	100.00	71002.76	89848.49
	2009	80.89	70.46	10.44	88259.97	7637.20	9.49	94.81	14.59	84.57	8.02	1.46	78.82	22.30	1.98	98.10	100.00	65840.66	89884.95
	2010	81.08	70.59	10.49	87770.27	8164.47	9.26	94.96	14.49	84.70	7.84	1.41	79.10	22.45	1.95	98.10	100.00	65710.06	89937.62
	2011	81.26	70.76	10.50	87481.15	9249.83	9.14	94.27	14.64	84.48	7.72	1.42	79.39	22.82	1.88	98.10	100.00	64181.15	89950.80
	2012	81.50	70.97	10.53	88689.49	9360.77	9.16	93.90	14.18	84.90	7.77	1.38	79.67	23.30	1.85	98.10	100.00	63536.73	89974.23
	2013	81.75	71.18	10.58	88538.70	9719.99	9.39	93.92	13.86	85.24	8.01	1.39	79.94	23.84	1.78	98.10	100.00	-	90010.39
	2014	82.03	71.40	10.63	89274.96	9522.22	9.72	93.80	13.61	85.49	8.31	1.41	80.21	24.36	1.75	98.10	100.00	-	90058.31
	2015	82.09	71.47	10.62	90132.39	-	-	-	-	-	-	-	80.47	24.80	1.75	98.10	100.00	-	90091.98
	2016	82.10	71.49	10.61	90344.41	-	-	-	-	-	-	-	80.73	25.30	-	-	-	-	90118.17
	2017	82.34	70.24	12.10	-	-	-	-	-	-	-	-	-	-	-	-	-	-	-
Oman	1995	72.67	62.32	10.35	16295.73	227.09	3.65	63.24	10.17	83.92	3.06	0.59	71.67	3.55	5.35	84.80	80.30	41448.64	89853.69
	1996	73.03	62.67	10.37	16548.97	242.12	3.56	63.00	10.15	83.90	2.98	0.57	71.65	3.64	4.98	85.70	81.10	43219.73	89757.94
	1997	73.42	63.02	10.40	17445.23	235.92	3.36	63.00	10.76	82.93	2.78	0.57	71.63	3.73	4.63	86.50	81.80	44902.25	89653.45
	1998	73.78	63.34	10.44	17889.47	231.72	3.70	64.44	11.89	81.56	3.02	0.68	71.61	3.82	4.30	87.30	82.50	27560.65	89533.03
	1999	74.13	63.65	10.48	17843.04	247.32	3.54	64.99	11.92	81.66	2.89	0.65	71.59	3.92	3.99	88.20	83.30	30791.43	89414.78
	2000	74.40	63.91	10.49	18698.40	266.88	3.07	64.39	11.75	81.75	2.51	0.56	71.57	4.02	3.72	89.00	84.00	35439.11	89310.32
	2001	74.62	64.10	10.52	19308.42	263.89	3.08	62.69	11.27	82.03	2.53	0.55	71.55	4.05	3.48	89.80	84.70	40680.09	89286.68
	2002	74.81	64.28	10.53	18772.80	275.76	3.18	61.40	10.15	83.47	2.65	0.53	71.53	4.06	3.29	90.70	85.50	43297.51	89307.71
	2003	75.03	64.47	10.55	17881.31	287.80	3.17	61.76	10.58	82.87	2.63	0.54	71.51	4.05	3.14	91.50	86.20	46464.79	89376.93

	2004	75.43	64.80	10.63	17671.58	301.00	2.98	61.65	11.15	81.91	2.44	0.54	71.83	4.02	3.03	92.40	87.00	46069.20	89454.65
	2005	75.78	65.12	10.66	17631.94	320.77	2.59	59.74	10.55	82.34	2.13	0.46	72.40	3.98	2.95	93.30	87.80	48099.66	89541.06
	2006	75.89	65.23	10.66	18063.25	336.81	2.31	59.44	11.48	80.69	1.86	0.45	72.97	3.92	2.91	94.20	88.60	50886.20	89680.10
	2007	75.90	65.21	10.69	18302.32	393.60	2.43	59.76	81.14	81.14	1.97	0.46	73.53	3.88	2.89	95.00	89.40	52515.09	89806.64
	2008	76.15	65.39	10.76	19112.20	462.07	2.01	58.82	12.45	78.83	1.59	0.43	74.08	3.85	2.89	95.90	90.10	55220.80	89958.50
	2009	75.96	65.27	10.69	19408.63	495.79	2.83	61.47	11.50	81.30	2.30	0.53	74.62	3.81	2.90	96.70	90.90	55510.21	90157.64
	2010	75.75	65.17	10.58	19280.75	546.64	2.74	60.20	10.41	82.71	2.27	0.47	75.16	3.75	2.90	96.70	91.70	59041.98	90405.78
	2011	75.94	65.31	10.63	17914.03	539.86	2.49	59.63	10.84	81.82	2.04	0.45	75.69	3.56	2.90	96.70	92.50	60705.59	90695.72
	2012	76.24	65.56	10.68	18300.33	551.58	2.52	59.35	10.43	82.43	2.08	0.44	76.20	3.37	2.88	96.70	93.30	62201.55	91004.25
	2013	76.40	65.70	10.70	17830.35	562.89	2.76	55.97	7.14	87.24	2.41	0.35	76.70	3.22	2.85	96.70	93.30	-	91278.14
	2014	76.58	65.86	10.72	17132.09	675.04	3.55	56.49	5.78	89.77	3.19	0.36	77.18	3.12	2.80	96.70	93.40	-	91492.58
	2015	76.78	66.02	10.76	17070.96	-	-	-	-	-	-	-	77.64	3.06	2.74	96.70	93.40	-	91668.41
	2016	76.97	66.20	10.76	-	-	-	-	-	-	-	-	78.09	3.08	-	-	-	-	91748.22
	2017	77.10	65.82	11.28	-	-	-	-	-	-	-	-	-	-	-	-	-	-	-
Pakistan	1995	61.95	53.84	8.12	815.34	15.78	2.50	97.65	72.09	26.18	0.66	1.85	31.84	7.47	5.34	27.80	87.40	209249.97	84679.01
	1996	62.10	54.00	8.10	833.75	15.77	2.57	97.66	70.53	27.78	0.72	1.86	32.09	7.49	5.20	29.60	87.60	220080.40	84705.67
	1997	62.07	54.00	8.07	821.66	15.41	2.57	97.71	72.74	25.56	0.66	1.91	32.35	7.49	5.05	31.40	87.80	225905.90	84789.87
	1998	62.28	54.19	8.09	822.44	16.22	2.75	82.45	65.31	20.78	0.57	2.18	32.61	7.49	4.89	33.20	88.10	234307.76	84889.09
	1999	62.44	54.33	8.11	832.70	16.30	2.83	82.82	66.60	19.59	0.55	2.28	32.87	7.48	4.73	35.00	88.30	246612.07	85010.14
	2000	62.61	54.48	8.13	848.63	15.50	2.79	81.49	63.70	21.83	0.61	2.18	33.16	7.46	4.58	36.90	88.50	247348.80	85133.82
	2001	62.76	54.60	8.16	846.64	13.53	2.61	79.12	62.39	21.15	0.55	2.06	33.45	7.45	4.44	38.70	88.70	250584.71	85115.00
	2002	62.93	54.73	8.20	855.50	15.39	2.76	79.39	56.14	29.29	0.81	1.95	33.75	7.44	4.31	40.50	88.90	256954.14	85121.25
	2003	63.09	54.84	8.25	878.44	16.12	2.61	81.27	61.08	24.85	0.65	1.96	34.07	7.41	4.21	42.30	89.10	271615.15	85105.40
	2004	63.35	55.04	8.32	923.92	17.76	2.56	82.37	60.56	26.48	0.68	1.88	34.39	7.39	4.13	44.10	89.30	291014.02	85138.88
	2005	62.52	54.32	8.21	974.54	22.36	2.91	86.34	66.01	23.54	0.69	2.23	34.73	7.38	4.07	45.90	89.50	299134.72	85151.36
	2006	64.01	55.56	8.45	1013.77	29.63	3.40	89.79	68.97	23.19	0.79	2.61	35.08	7.40	4.02	47.60	89.70	314154.54	85216.99
	2007	64.35	55.85	8.51	1041.29	31.93	3.35	88.98	23.82	23.82	0.80	2.55	35.44	7.42	3.98	49.40	89.90	336409.91	85275.33
	2008	64.65	56.09	8.56	1037.58	30.19	3.26	88.31	65.57	25.75	0.84	2.42	35.82	7.44	3.94	51.20	90.10	338551.92	85316.57
	2009	64.97	56.35	8.61	1045.21	28.51	2.94	88.50	64.32	27.32	0.80	2.14	36.20	7.44	3.90	53.00	90.30	343244.20	85352.13

	2010	65.29	56.62	8.67	1040.14	30.95	3.02	88.65	60.61	31.64	0.95	2.06	36.60	7.44	3.86	54.80	90.50	354528.62	85401.15
	2011	65.73	56.99	8.74	1046.49	36.66	3.01	88.59	61.49	30.59	0.92	2.09	37.01	7.45	3.80	56.50	90.70	362475.09	85417.27
	2012	66.08	57.30	8.78	1060.50	33.47	2.76	86.79	54.76	36.90	1.02	1.74	37.43	7.45	3.74	58.30	90.90	369734.58	85445.05
	2013	66.46	57.63	8.83	1083.97	33.64	2.70	86.79	54.87	36.77	0.99	1.71	37.86	7.45	3.68	60.00	91.10	-	85508.64
	2014	66.83	57.94	8.88	1111.20	36.15	2.61	86.79	56.28	35.15	0.92	1.70	38.30	7.43	3.62	61.80	91.30	-	85599.24
	2015	67.21	58.27	8.94	1140.21	-	-	-	-	-	-	-	38.76	7.41	3.55	63.50	91.40	-	85742.65
	2016	67.61	58.63	8.98	1178.80	-	-	-	-	-	-	-	39.22	7.41	-	-	-	-	85911.81
	2017	66.85	58.00	8.85	-	-	-	-	-	-	-	-	-	-	-	-	-	-	-
Panama	1995	75.37	66.62	8.75	4741.13	222.93	7.72	84.10	26.95	67.96	5.25	2.47	58.14	8.33	2.85	63.00	86.90	8817.59	88331.35
	1996	75.58	66.78	8.80	4834.65	239.58	7.18	86.22	30.66	64.44	4.63	2.55	58.98	8.41	2.83	63.80	87.40	8891.11	88321.72
	1997	75.79	66.95	8.84	5043.14	251.69	7.12	83.75	26.41	68.47	4.88	2.25	59.81	8.50	2.81	64.60	88.00	8870.50	88322.48
	1998	76.06	67.16	8.89	5304.85	266.09	7.09	83.33	27.74	66.71	4.73	2.36	60.63	8.60	2.79	65.30	88.50	10070.74	88338.01
	1999	76.41	67.44	8.98	5403.43	264.39	6.85	81.87	27.61	66.28	4.54	2.31	61.45	8.71	2.77	66.10	89.00	8736.71	88379.01
	2000	76.76	67.72	9.04	5441.82	297.88	7.76	81.26	25.95	68.07	5.28	2.48	62.20	8.83	2.74	66.80	89.50	9158.75	88449.62
	2001	76.96	67.87	9.09	5367.96	295.35	7.72	82.34	27.10	67.08	5.18	2.54	62.49	8.96	2.72	67.40	89.90	11337.66	88519.82
	2002	77.07	67.97	9.10	5383.80	313.35	8.03	81.64	25.27	69.05	5.54	2.49	62.79	9.10	2.70	67.90	90.30	10475.86	88614.27
	2003	77.16	68.04	9.12	5505.49	307.10	7.60	82.16	27.64	66.37	5.05	2.56	63.08	9.25	2.68	68.50	90.60	10850.46	88701.60
	2004	77.27	68.14	9.14	5810.32	351.65	8.09	81.47	24.16	70.35	5.69	2.40	63.38	9.41	2.66	69.00	91.00	10819.01	88774.05
	2005	77.39	68.25	9.14	6114.22	348.45	7.48	81.06	24.64	69.60	5.21	2.27	63.67	9.58	2.65	69.60	91.40	10588.62	88830.87
	2006	77.45	68.29	9.16	6522.92	354.88	7.00	80.77	26.27	67.47	4.72	2.28	63.96	9.75	2.64	70.10	91.70	11987.78	88851.00
	2007	77.50	68.30	9.21	7173.70	387.41	6.31	83.01	64.20	64.20	4.05	2.26	64.25	9.94	2.64	70.70	92.10	11689.15	88860.23
	2008	77.52	68.29	9.23	7653.07	480.09	6.75	83.61	25.81	69.13	4.67	2.08	64.54	10.13	2.63	71.20	92.40	15082.59	88875.95
	2009	77.61	68.35	9.26	7638.29	550.66	7.56	83.61	21.32	74.50	5.63	1.93	64.83	10.33	2.63	71.80	92.80	15489.51	88904.08
	2010	77.73	68.45	9.28	7937.26	640.44	8.05	83.61	24.91	70.21	5.65	2.40	65.12	10.55	2.62	72.30	93.10	15428.76	88929.39
	2011	77.82	68.50	9.32	8719.97	677.83	7.50	83.91	26.98	67.85	5.09	2.41	65.41	10.75	2.61	72.90	93.40	15860.46	88906.17
	2012	77.93	68.59	9.35	9360.34	734.56	7.25	79.03	24.80	68.62	4.97	2.27	65.70	10.97	2.60	73.40	93.70	16248.77	88892.54
	2013	78.13	68.74	9.39	9810.01	906.14	8.09	78.74	22.22	71.78	5.80	2.28	65.99	11.19	2.59	73.90	94.10	-	88903.64
	2014	78.33	68.89	9.44	10229.23	958.98	8.03	83.22	22.27	73.24	5.88	2.15	66.29	11.43	2.57	74.50	94.40	-	88912.62
	2015	78.64	69.13	9.51	10642.30	-	-	-	-	-	-	-	66.59	11.70	2.54	75.00	94.70	-	88953.60

	2016	78.92	69.38	9.54	10982.37	-	-	-	-	-	-	-	66.90	11.96	-	-	-	-	89018.59
	2017	79.29	69.44	79.29	-	-	-	-	-	-	-	-	-	-	-	-	-	-	-
Papua New Guinea	1995	56.90	49.90	7.00	1984.10	30.10	2.93	46.30	7.62	83.54	2.45	0.48	14.08	5.43	4.67	19.70	33.60	35410.12	86722.83
	1996	57.17	50.14	7.03	2083.00	32.95	3.10	46.50	7.66	83.52	2.59	0.51	13.90	5.47	4.65	19.60	33.90	34368.44	86707.51
	1997	57.34	50.28	7.06	1950.23	36.08	3.64	46.82	8.52	81.81	2.98	0.66	13.72	5.50	4.63	19.50	34.20	143275.03	86709.18
	1998	56.66	49.69	6.96	1828.39	28.10	3.81	46.88	7.91	83.14	3.17	0.64	13.55	5.52	4.60	19.40	34.50	8730.32	86703.76
	1999	57.53	50.43	7.10	1814.55	26.84	4.09	48.75	8.58	82.40	3.37	0.72	13.38	5.53	4.57	19.30	34.80	6685.11	86713.08
	2000	57.60	50.47	7.13	1724.27	25.94	3.98	56.05	10.24	81.74	3.26	0.73	13.20	5.53	4.53	19.20	35.10	8257.46	86752.59
	2001	57.58	50.46	7.12	1678.82	37.15	6.68	55.89	6.54	88.30	5.90	0.78	13.18	5.58	4.48	19.20	35.50	10359.94	86818.23
	2002	57.56	50.46	7.11	1634.36	41.46	7.69	55.89	7.07	87.35	6.72	0.97	13.16	5.62	4.42	19.10	35.90	84384.82	86886.54
	2003	57.56	50.45	7.11	1628.50	43.55	6.79	55.89	6.31	88.70	6.02	0.77	13.15	5.65	4.37	19.10	36.30	10217.69	86964.11
	2004	57.56	50.45	7.11	1631.87	59.01	8.39	55.89	5.27	90.57	7.60	0.79	13.13	5.67	4.32	19.10	36.70	19650.16	87075.86
	2005	57.61	50.48	7.13	1693.31	51.29	6.42	55.89	8.23	85.27	5.47	0.95	13.11	5.69	4.26	19.10	37.10	9453.03	87169.99
	2006	57.71	50.57	7.14	1690.52	52.47	5.92	55.89	8.12	85.47	5.06	0.86	13.09	5.74	4.21	19.10	37.50	14195.87	87244.45
	2007	57.87	50.71	7.15	1833.46	40.08	4.04	55.89	77.38	77.38	3.12	0.91	13.07	5.78	4.16	19.00	37.90	9773.24	87318.73
	2008	58.17	50.96	7.21	1785.17	49.61	4.06	55.89	13.51	75.82	3.08	0.98	13.06	5.82	4.10	19.00	38.30	9516.47	87390.68
	2009	58.39	51.14	7.25	1862.56	52.76	4.36	55.89	14.58	73.91	3.22	1.14	13.04	5.85	4.04	19.00	38.70	11298.73	87493.63
	2010	58.66	51.35	7.31	2004.82	59.25	4.18	55.89	13.28	76.24	3.18	0.99	13.02	5.87	3.99	19.00	39.10	10667.60	87576.19
	2011	59.00	51.64	7.36	1982.09	90.89	4.94	55.89	10.19	81.76	4.04	0.90	13.00	5.92	3.93	18.90	39.50	10888.09	87637.79
	2012	59.25	51.86	7.39	2029.31	97.88	4.55	55.89	11.02	80.29	3.65	0.90	12.98	5.96	3.87	18.90	39.90	11087.46	87692.71
	2013	59.70	52.23	7.47	2061.96	100.77	4.78	55.89	10.21	81.74	3.91	0.87	12.98	5.99	3.81	18.90	39.90	-	87754.97
	2014	60.08	52.54	7.54	2271.48	92.36	4.26	55.89	10.46	81.28	3.47	0.80	12.99	6.04	3.76	18.90	40.00	-	87804.96
	2015	60.44	52.84	7.60	2428.78	-	-	-	-	-	-	-	13.01	6.10	3.71	18.90	40.00	-	87865.32
	2016	60.79	53.13	7.67	2436.18	-	-	-	-	-	-	-	13.04	6.21	-	-	-	-	87916.05
	2017	58.50	50.83	7.66	-	-	-	-	-	-	-	-	-	-	-	-	-	-	-
Paraguay	1995	74.46	65.04	9.42	2937.58	112.74	5.92	90.37	57.87	35.96	2.13	3.79	52.13	7.81	4.11	60.60	63.60	60568.50	89474.93
	1996	74.44	65.02	9.41	2916.52	119.32	5.94	89.20	53.21	40.35	2.40	3.54	52.77	7.82	4.01	62.20	65.60	62323.21	89523.79
	1997	74.47	65.04	9.43	2973.31	123.88	6.19	85.32	51.86	39.22	2.43	3.76	53.41	7.80	3.91	63.80	67.60	63388.76	89584.27
	1998	74.45	65.02	9.44	2911.63	111.45	6.29	86.47	47.66	44.88	2.82	3.46	54.05	7.76	3.80	65.40	69.60	48081.51	89655.59

	1999	74.44	64.99	9.45	2812.38	109.72	6.79	83.70	46.36	44.61	3.03	3.76	54.69	7.74	3.68	67.00	71.50	136601.13	89743.69
	2000	74.37	64.92	9.46	2692.50	125.20	8.10	86.61	52.07	39.88	3.23	4.87	55.33	7.74	3.55	68.50	73.40	39439.47	89839.77
	2001	74.30	64.85	9.45	2618.73	107.91	7.61	84.94	55.33	34.86	2.65	4.96	55.97	7.79	3.43	70.10	75.30	86905.95	89934.00
	2002	74.25	64.79	9.45	2569.69	77.75	6.77	85.60	58.29	31.90	2.16	4.61	56.60	7.85	3.32	71.60	77.10	83003.03	90034.83
	2003	74.18	64.73	9.45	2633.24	70.32	5.99	84.90	57.24	32.57	1.95	4.04	56.89	7.92	3.21	73.00	78.80	83847.27	90143.84
	2004	73.93	64.51	9.41	2694.06	81.37	5.78	85.24	55.27	35.17	2.03	3.75	57.12	8.00	3.12	74.40	80.50	69049.06	90257.62
	2005	74.08	64.64	9.44	2707.97	91.54	6.07	85.55	52.38	38.78	2.36	3.72	57.35	8.07	3.04	75.80	82.10	90747.01	90352.93
	2006	74.06	64.63	9.43	2796.03	116.32	6.43	86.02	51.09	40.61	2.61	3.82	57.58	8.17	2.97	77.20	83.80	67108.72	90430.19
	2007	74.07	64.63	9.44	2906.44	148.25	6.41	86.04	41.99	41.99	2.69	3.72	57.81	8.27	2.91	78.60	85.40	138575.70	90515.53
	2008	74.05	64.61	9.44	3049.87	216.15	7.06	88.90	54.75	38.41	2.71	4.35	58.03	8.37	2.85	80.00	87.00	54092.03	90578.91
	2009	73.97	64.55	9.42	2890.34	237.66	9.14	90.58	55.91	38.28	3.50	5.64	58.26	8.49	2.79	81.30	88.70	46875.60	90662.94
	2010	73.99	64.56	9.43	3225.59	293.83	9.10	91.52	57.72	36.93	3.36	5.74	58.49	8.61	2.73	82.70	90.30	50027.50	90738.37
	2011	74.03	64.59	9.44	3320.79	375.81	9.42	91.38	54.86	39.96	3.77	5.66	58.71	8.74	2.68	84.10	91.90	50445.12	90804.70
	2012	74.02	64.59	9.44	3235.72	398.44	10.33	91.38	50.72	44.50	4.60	5.74	58.94	8.88	2.63	85.40	93.40	50843.95	90865.50
	2013	74.06	64.62	9.44	3640.52	469.01	10.49	91.74	49.94	45.56	4.78	5.71	59.17	9.03	2.58	86.80	95.00	-	90926.78
	2014	74.13	64.68	9.45	3761.91	464.09	9.81	91.31	49.42	45.87	4.50	5.31	59.42	9.22	2.54	87.80	96.60	-	90984.74
	2015	74.32	64.82	9.49	3822.86	-	-	-	-	-	-	-	59.67	9.43	2.51	88.60	98.00	-	91049.19
	2016	74.48	64.96	9.52	3925.56	-	-	-	-	-	-	-	59.92	9.67	-	-	-	-	91104.61
	2017	76.12	66.05	10.07	-	-	-	-	-	-	-	-	-	-	-	-	-	-	-
Peru	1995	71.20	62.59	8.62	3140.77	96.63	4.46	85.92	39.72	53.77	2.40	2.06	70.95	7.33	3.32	58.00	77.10	63406.51	90984.14
	1996	71.87	63.16	8.72	3175.54	99.52	4.50	86.05	39.45	54.15	2.44	2.07	71.38	7.47	3.23	59.00	77.70	66878.35	91016.74
	1997	72.55	63.73	8.82	3328.60	100.34	4.38	85.72	41.40	51.70	2.27	2.12	71.80	7.62	3.14	60.00	78.20	67172.28	91061.55
	1998	73.28	64.34	8.94	3266.57	98.62	4.56	83.84	36.53	56.42	2.57	1.99	72.22	7.76	3.06	61.00	78.80	70299.96	91114.60
	1999	74.12	65.04	9.08	3268.51	95.64	4.95	81.20	34.81	57.13	2.83	2.12	72.63	7.90	2.99	62.00	79.30	64302.17	91170.14
	2000	74.81	65.64	9.17	3310.78	94.97	4.83	83.43	36.41	56.36	2.72	2.11	73.04	8.04	2.93	62.90	79.80	66574.04	91226.45
	2001	75.39	66.09	9.30	3287.29	97.97	4.98	82.60	35.68	56.80	2.83	2.15	73.45	8.25	2.87	63.90	80.30	49674.78	91299.02
	2002	75.90	66.52	9.38	3422.24	101.10	4.94	81.83	35.01	57.22	2.83	2.11	73.85	8.46	2.82	64.80	80.90	51029.15	91377.73
	2003	76.29	66.86	9.43	3520.28	100.80	4.60	78.04	32.38	58.51	2.69	1.91	74.25	8.66	2.77	65.80	81.40	53092.70	91441.21
	2004	76.70	67.21	9.50	3649.37	111.58	4.56	79.83	35.32	55.76	2.54	2.02	74.64	8.86	2.73	66.70	81.80	56438.52	91507.22

	2005	77.11	67.60	9.51	3831.36	128.10	4.72	77.65	33.32	57.10	2.69	2.02	75.03	9.04	2.68	67.60	82.30	68557.46	91559.73
	2006	77.52	67.95	9.57	4069.78	140.48	4.47	85.24	39.61	53.54	2.39	2.08	75.42	9.15	2.65	68.50	82.80	58736.85	91578.70
	2007	77.72	68.06	9.66	4362.95	166.15	4.60	86.18	52.50	52.50	2.41	2.18	75.80	9.25	2.62	69.40	83.30	70911.51	91586.98
	2008	77.93	68.24	9.68	4703.08	200.87	4.73	86.01	41.42	51.84	2.45	2.28	76.18	9.36	2.59	70.30	83.70	63708.12	91579.00
	2009	77.87	68.22	9.65	4695.68	224.10	5.36	84.46	37.46	55.65	2.98	2.38	76.55	9.48	2.57	71.20	84.20	64753.41	91570.32
	2010	77.94	68.37	9.57	5022.49	254.48	5.03	83.48	37.12	55.53	2.79	2.24	76.92	9.62	2.55	72.00	84.60	71614.29	91549.19
	2011	78.17	68.47	9.69	5270.95	282.45	4.93	79.58	37.20	53.26	2.62	2.30	77.27	9.75	2.53	72.90	85.00	73289.70	91513.02
	2012	78.50	68.74	9.76	5520.56	330.75	5.18	79.16	35.62	55.00	2.85	2.33	77.62	9.90	2.50	73.70	85.50	74806.96	91479.39
	2013	78.91	69.06	9.85	5765.89	346.49	5.23	76.27	31.88	58.20	3.05	2.19	77.95	10.06	2.48	74.60	85.90	-	91454.52
	2014	79.37	69.42	9.95	5823.96	358.58	5.47	72.71	28.62	60.64	3.32	2.15	78.29	10.25	2.46	75.40	86.30	-	91421.57
	2015	79.56	69.58	9.98	5936.02	-	-	-	-	-	-	-	78.61	10.46	2.43	76.20	86.70	-	91412.18
	2016	79.74	69.84	9.90	6089.40	-	-	-	-	-	-	-	78.92	10.68	-	-	-	-	91412.32
	2017	80.31	70.28	10.03	-	-	-	-	-	-	-	-	-	-	-	-	-	-	-
Philippines	1995	67.98	59.54	8.45	1506.58	36.61	3.45	82.72	50.01	39.53	1.36	2.09	48.29	5.40	4.01	60.50	85.50	125623.45	90284.87
	1996	68.16	59.68	8.48	1558.71	41.08	3.54	81.75	48.25	40.98	1.45	2.09	48.23	5.43	3.96	61.10	85.80	134296.65	90461.54
	1997	68.23	59.73	8.50	1603.22	40.80	3.62	81.62	46.48	43.05	1.56	2.06	48.17	5.47	3.92	61.80	86.10	140736.42	90653.52
	1998	68.20	59.69	8.51	1559.21	30.94	3.20	81.13	46.30	42.93	1.37	1.83	48.11	5.52	3.89	62.50	86.40	163224.11	90859.66
	1999	68.16	59.64	8.52	1572.69	35.15	3.23	77.64	43.32	44.20	1.43	1.80	48.05	5.56	3.85	63.20	86.70	140842.11	91082.88
	2000	68.05	59.52	8.53	1607.20	33.37	3.21	77.25	40.50	47.57	1.53	1.68	47.96	5.59	3.81	63.80	87.10	150078.87	91314.83
	2001	68.07	59.58	8.49	1618.97	28.72	3.00	78.55	43.85	44.17	1.32	1.67	47.68	5.65	3.77	64.50	87.40	142869.33	91312.54
	2002	68.05	59.59	8.46	1643.20	27.93	2.79	77.97	46.78	40.01	1.12	1.67	47.41	5.71	3.71	65.10	87.70	138051.04	91322.88
	2003	68.03	59.59	8.44	1689.98	32.84	3.25	78.43	46.88	40.23	1.31	1.94	47.14	5.76	3.65	65.80	88.00	140443.04	91327.21
	2004	68.03	59.60	8.43	1768.11	34.87	3.23	78.63	46.89	40.37	1.30	1.93	46.87	5.80	3.58	66.40	88.30	142978.77	91344.05
	2005	68.15	59.70	8.46	1818.32	46.83	3.91	84.25	51.93	38.37	1.50	2.41	46.60	5.85	3.50	67.10	88.60	146433.72	91350.85
	2006	68.16	59.77	8.38	1880.19	55.13	3.95	85.30	53.96	36.75	1.45	2.50	46.33	6.02	3.43	67.80	88.90	201211.26	91132.91
	2007	68.38	60.02	8.37	1971.28	66.15	3.94	85.27	34.71	34.71	1.37	2.57	46.06	6.19	3.35	68.40	89.20	149106.27	90953.75
	2008	68.50	60.15	8.35	2020.15	78.08	4.05	85.24	57.17	32.93	1.33	2.71	45.79	6.34	3.28	69.10	89.50	152332.05	90795.88
	2009	68.66	60.31	8.35	2010.80	81.01	4.41	84.55	54.86	35.11	1.55	2.86	45.52	6.50	3.21	69.80	89.80	223801.40	90661.52
	2010	68.87	60.51	8.36	2129.50	93.83	4.37	84.47	54.08	35.98	1.57	2.80	45.26	6.68	3.16	70.50	90.10	159940.45	90573.56

	2011	69.01	60.65	8.36	2171.49	101.75	4.29	83.02	57.74	30.45	1.31	2.98	45.02	6.77	3.11	71.10	90.50	163797.79	90558.17
	2012	69.17	60.81	8.37	2278.64	116.19	4.46	83.00	57.19	31.09	1.39	3.07	44.81	6.87	3.07	71.80	90.80	167297.55	90537.54
	2013	69.24	60.88	8.36	2399.61	127.09	4.56	82.61	56.34	31.80	1.45	3.11	44.63	6.99	3.03	72.50	91.10	-	90515.20
	2014	69.67	61.25	8.42	2505.82	135.20	4.71	81.69	53.69	34.28	1.61	3.10	44.49	7.11	2.99	73.20	91.50	-	90486.81
	2015	69.92	61.47	8.45	2615.66	-	-	-	-	-	-	-	44.37	7.23	2.96	73.90	91.80	-	90467.54
	2016	70.10	61.64	8.46	2753.35	-	-	-	-	-	-	-	44.29	7.40	-	-	-	-	90447.45
	2017	69.73	60.99	8.74	-	-	-	-	-	-	-	-	-	-	-	-	-	-	-
Poland	1995	71.82	62.76	9.06	6539.05	197.38	5.36	100.00	27.11	72.89	3.91	1.45	61.49	16.59	1.62		95.00	458557.08	91796.45
	1996	72.28	63.12	9.15	6929.82	238.48	5.76	100.00	26.61	73.39	4.22	1.53	61.54	16.87	1.59	87.00	95.20	435648.57	91848.19
	1997	72.60	63.40	9.20	7372.62	227.99	5.54	100.00	28.04	71.96	3.98	1.55	61.58	17.10	1.51	87.00	95.40	433538.61	91905.79
	1998	72.98	63.70	9.28	7710.09	264.14	5.86	100.00	34.62	65.38	3.83	2.03	61.63	17.29	1.44	87.00	95.60	388905.68	91979.68
	1999	73.11	63.82	9.30	8068.67	248.86	5.67	100.00	28.87	71.13	4.03	1.64	61.67	17.44	1.38	87.00	95.80	380841.87	92044.79
	2000	73.77	64.38	9.39	8525.14	247.14	5.50	100.00	29.97	70.03	3.85	1.65	61.72	17.58	1.37	87.00	95.90	392642.04	92106.61
	2001	74.25	64.69	9.56	8633.92	291.66	5.86	100.00	28.10	71.90	4.21	1.65	61.76	17.86	1.31	87.80	96.10	385653.89	92162.89
	2002	74.63	64.98	9.65	8814.27	328.38	6.32	88.21	25.44	71.16	4.50	1.82	61.79	18.14	1.25	88.50	96.30	375643.18	92228.87
	2003	74.92	65.19	9.72	9134.44	354.11	6.22	87.96	26.43	69.91	4.35	1.87	61.68	18.38	1.22	89.20	96.50	387619.14	92289.03
	2004	75.05	65.28	9.77	9609.17	410.62	6.19	89.61	28.11	68.58	4.24	1.94	61.56	18.54	1.23	89.90	96.60	411840.44	92342.18
	2005	75.18	65.45	9.73	9949.25	494.70	6.20	85.35	26.12	69.30	4.30	1.90	61.45	18.60	1.24	90.60	96.80	411811.67	92386.78
	2006	75.29	65.55	9.74	10570.77	555.12	6.17	85.36	25.59	69.90	4.31	1.85	61.34	18.66	1.27	91.30	97.00	423290.67	92397.50
	2007	75.38	65.50	9.88	11320.55	706.20	6.28	83.25	70.39	70.39	4.42	1.86	61.23	18.63	1.31	92.10	97.10	421174.65	92403.56
	2008	75.60	65.66	9.95	11800.03	956.52	6.88	80.96	22.80	71.77	4.94	1.94	61.12	18.58	1.39	92.80	97.30	420981.53	92396.10
	2009	75.86	65.84	10.02	12124.61	815.01	7.12	80.57	22.70	71.58	5.10	2.01	61.00	18.65	1.40	93.50	97.50	405421.16	92401.94
	2010	76.31	66.27	10.04	12597.86	867.75	6.88	78.25	22.11	71.22	4.90	1.95	60.89	18.89	1.41	94.30	97.60	426485.87	92445.44
	2011	76.57	66.37	10.20	13222.81	931.05	6.70	76.20	22.28	70.28	4.71	1.96	60.78	19.36	1.33	95.00	97.80	422791.47	92436.30
	2012	76.71	66.51	10.21	13435.46	869.70	6.62	75.00	22.74	69.17	4.58	2.01	60.69	19.98	1.33	95.70	98.00	414606.89	92460.87
	2013	77.05	66.77	10.28	13630.69	881.77	6.40	80.65	23.55	70.80	4.53	1.87	60.62	20.73	1.29	96.50	98.20	-	92466.08
	2014	77.57	67.17	10.40	14088.75	910.28	6.35	80.85	23.46	70.98	4.51	1.84	60.57	21.57	1.32	97.20	98.30	-	92470.50
	2015	77.75	67.32	10.43	14640.15	-	-	-	-	-	-	-	60.54	22.46	1.32	97.20	98.30	-	92492.93
	2016	77.93	67.60	10.33	15074.73	-	-	-	-	-	-	-	60.53	23.46	-	-	-	-	92526.53

	2017	77.99	66.81	11.18	-	-	-	-	-	-	-	-	-	-	-	-	-	-	-
Portugal	1995	75.31	65.76	9.55	18080.55	875.76	7.42	63.99	23.92	62.62	4.65	2.77	51.11	22.36	1.41	94.50	97.10	69204.41	91283.68
	1996	75.44	65.89	9.55	18642.54	940.32	7.70	67.16	23.33	65.25	5.02	2.68	51.77	22.68	1.44	94.90	97.20	67633.10	91318.35
	1997	75.79	66.20	9.59	19380.98	892.43	7.69	66.67	22.87	65.69	5.05	2.64	52.43	23.01	1.47	95.20	97.40	70751.83	91319.68
	1998	76.06	66.44	9.61	20207.28	933.01	7.63	69.82	22.96	67.11	5.12	2.51	53.09	23.37	1.47	95.50	97.60	83126.73	91315.70
	1999	76.29	66.66	9.63	20874.58	984.93	7.87	73.02	23.68	67.56	5.32	2.55	53.74	23.73	1.50	95.80	97.80	84722.77	91297.75
	2000	76.70	67.00	9.69	21513.46	1055.67	9.14	70.03	22.58	67.75	6.19	2.95	54.40	24.11	1.55	96.20	98.00	88594.19	91306.80
	2001	77.04	67.26	9.78	21777.41	1071.10	9.10	70.91	22.36	68.47	6.23	2.87	55.04	24.44	1.45	96.50	98.10	85398.97	91381.85
	2002	77.34	67.52	9.82	21824.98	1182.29	9.15	71.01	20.88	70.59	6.46	2.69	55.67	24.76	1.46	96.80	98.30	88319.25	91459.58
	2003	77.64	67.77	9.87	21540.07	1506.96	9.54	69.06	21.55	68.80	6.56	2.98	56.29	25.07	1.44	97.10	98.50	89900.20	91548.67
	2004	78.09	68.12	9.97	21877.91	1777.19	9.84	72.38	21.66	70.07	6.89	2.94	56.91	25.36	1.40	97.30	98.60	84930.56	91653.27
	2005	78.37	68.38	9.99	22004.80	1875.70	9.98	73.55	22.02	70.06	6.99	2.99	57.52	25.63	1.41	97.60	98.80	101594.26	91748.63
	2006	78.79	68.72	10.07	22306.28	1919.22	9.67	74.12	23.67	68.06	6.58	3.09	58.14	26.10	1.37	97.90	99.00	81336.95	91835.27
	2007	79.07	68.91	10.16	22817.32	2193.50	9.62	74.64	67.64	67.64	6.51	3.11	58.75	26.56	1.35	98.20	99.10	79399.30	91923.86
	2008	79.35	69.13	10.22	22829.85	2457.72	9.90	74.81	24.37	67.42	6.67	3.22	59.36	27.03	1.39	98.50	99.30	74433.77	92015.43
	2009	79.63	69.34	10.29	22128.85	2403.96	10.42	74.38	23.26	68.73	7.16	3.26	59.96	27.56	1.34	98.70	99.50	75492.50	92109.80
	2010	79.91	69.59	10.32	22538.65	2352.32	10.44	74.49	23.30	68.71	7.17	3.27	60.57	28.17	1.39	99.00	99.60	71681.47	92223.61
	2011	80.21	69.80	10.40	22159.48	2332.35	10.07	75.22	25.16	66.55	6.70	3.37	61.17	28.82	1.35	99.20	99.80	71977.89	92306.17
	2012	80.38	69.96	10.42	21353.23	1999.82	9.74	76.16	27.40	64.03	6.24	3.50	61.76	29.52	1.28	99.50	99.90	72524.22	92411.60
	2013	80.61	70.15	10.46	21228.09	2059.46	9.55	75.89	26.38	65.24	6.23	3.32	62.34	30.26	1.21	99.60	100.00	-	92526.88
	2014	80.83	70.33	10.50	21533.49	2096.82	9.50	76.29	26.84	64.82	6.16	3.34	62.91	31.03	1.23	99.70	100.00	-	92646.68
	2015	80.90	70.40	10.50	22016.84	-	-	-	-	-	-	-	63.47	31.82	1.23	99.70	100.00	-	92761.64
	2016	80.99	70.51	10.47	22428.11	-	-	-	-	-	-	-	64.02	32.48	-	-	-	-	92869.03
	2017	81.44	70.13	11.31	-	-	-	-	-	-	-	-	-	-	-	-	-	-	-
Puerto Rico	1995	73.75	64.57	9.18	21966.86	-	-	-	-	-	-	-	93.73	16.29	2.09	99.30	93.60	2888.35	91663.68
	1996	74.05	64.81	9.24	22350.30	-	-	-	-	-	-	-	93.88	16.45	1.94	99.30	93.60	2947.39	91694.00
	1997	75.02	65.56	9.46	22842.31	-	-	-	-	-	-	-	94.02	16.63	1.90	99.30	93.60	2996.52	91748.19
	1998	75.60	66.00	9.60	24243.43	-	-	-	-	-	-	-	94.16	16.84	1.90	99.30	93.60	2864.89	91804.23
	1999	75.69	66.05	9.64	24620.79	-	-	-	-	-	-	-	94.30	17.09	1.97	99.30	93.60	2912.55	91869.56

	2000	76.06	66.32	9.74	24924.15	-	-	-	-	-	-	-	94.39	17.38	2.05	99.30	93.60	2963.27	91954.27
	2001	76.26	66.48	9.78	27320.47	-	-	-	-	-	-	-	94.33	17.63	1.93	99.30	93.60	3010.80	91981.47
	2002	76.60	66.74	9.86	27769.11	-	-	-	-	-	-	-	94.28	17.87	1.83	99.30	-	3031.48	92031.46
	2003	76.88	66.96	9.92	28082.79	-	-	-	-	-	-	-	94.22	18.10	1.76	99.30	-	3095.29	92073.41
	2004	76.92	66.99	9.94	28571.45	-	-	-	-	-	-	-	94.17	18.31	1.78	99.30	-	3098.94	92135.25
	2005	77.07	67.11	9.96	28775.39	-	-	-	-	-	-	-	94.11	18.52	1.77	99.30	-	3118.67	92228.06
	2006	77.33	67.33	10.01	28640.44	-	-	-	-	-	-	-	94.06	18.77	1.71	99.30	-	3105.72	92277.07
	2007	77.49	67.43	10.06	27850.56	-	-	-	-	-	-	-	94.00	18.98	1.65	99.30	-	3064.31	92323.84
	2008	77.66	67.57	10.09	27510.51	-	-	-	-	-	-	-	93.94	19.15	1.65	99.30	-	3067.97	92374.30
	2009	77.90	67.75	10.15	27007.86	-	-	-	-	-	-	-	93.88	19.33	1.65	99.30	-	3119.07	92427.12
	2010	77.98	67.86	10.12	26435.74	-	-	-	-	-	-	-	93.83	19.54	1.62	99.30	-	3199.25	92485.78
	2011	78.22	68.03	10.18	26237.68	-	-	-	-	-	-	-	93.77	19.96	1.60	99.30	-	3247.52	92508.43
	2012	78.31	68.14	10.16	25820.79	-	-	-	-	-	-	-	93.72	20.41	1.54	99.30	-	3293.25	92533.96
	2013	78.41	68.25	10.16	25967.59	-	-	-	-	-	-	-	93.68	20.87	1.47	99.30	-	-	92563.96
	2014	78.37	68.25	10.12	-	-	-	-	-	-	-	-	93.64	21.30	1.43	99.30	-	-	92604.45
	2015	78.55	68.41	10.15	-	-	-	-	-	-	-	-	93.60	21.70	1.43	99.30	-	-	92644.69
	2016	78.77	68.62	10.15	-	-	-	-	-	-	-	-	93.57	22.20	-	-	-	-	92693.99
	2017	81.44	70.13	11.31	-	-	-	-	-	-	-	-	-	-	-	-	-	-	-
Qatar	1995	74.64	63.81	10.83	-	601.71	3.70	92.68	34.63	62.64	2.32	1.38	95.00	1.95	3.59	99.90	98.70	-	89445.98
	1996	74.69	63.85	10.84	-	653.45	3.69	91.14	29.57	67.56	2.49	1.20	95.36	2.02	3.53	99.90	98.70	-	89524.07
	1997	74.75	63.91	10.85	-	672.57	3.14	90.24	28.61	68.29	2.15	1.00	95.66	2.09	3.47	99.80	98.80	-	89664.98
	1998	74.81	63.95	10.86	-	635.70	3.40	89.41	29.93	66.52	2.26	1.14	95.89	2.16	3.41	99.60	98.90	38744.69	89815.08
	1999	74.84	63.97	10.86	-	630.87	2.90	88.37	29.00	67.19	1.95	0.95	96.11	2.24	3.33	99.50	99.00	43695.70	89974.63
	2000	75.01	64.10	10.91	60858.19	651.80	2.18	100.00	27.70	72.30	1.57	0.60	96.31	2.32	3.24	99.40	99.10	44439.70	90116.90
	2001	75.25	64.30	10.95	60707.12	730.57	2.56	100.00	24.82	75.18	1.92	0.63	96.51	2.16	3.12	99.30	99.20	45516.27	90149.94
	2002	75.31	64.37	10.94	62167.56	827.74	2.71	100.00	24.92	75.08	2.04	0.68	96.69	2.01	3.00	99.20	99.40	46983.92	90151.62
	2003	75.55	64.57	10.98	60460.42	1458.93	4.14	100.00	15.79	84.21	3.49	0.65	96.87	1.87	2.86	99.10	99.50	51782.17	90144.54
	2004	75.98	64.92	11.06	65405.72	1563.70	3.61	100.00	15.81	84.19	3.04	0.57	97.11	1.73	2.71	98.90	99.60	58827.54	90177.39
	2005	76.37	65.27	11.10	61688.81	1603.30	3.01	100.00	15.87	84.13	2.54	0.48	97.45	1.61	2.57	98.80	99.70	61388.82	90264.52

	2006	76.78	65.62	11.16	66623.14	1591.21	2.58	100.00	15.92	84.08	2.17	0.41	97.75	1.47	2.43	98.70	99.80	68073.79	90281.99
	2007	77.27	66.01	11.26	66761.62	1563.75	2.31	100.00	84.02	84.02	1.94	0.37	98.02	1.39	2.31	98.60	99.90	77135.76	90369.29
	2008	77.77	66.40	11.37	67262.45	1555.81	1.87	100.00	16.03	83.97	1.57	0.30	98.26	1.32	2.21	98.50	100.00	83149.37	90486.63
	2009	78.34	66.82	11.51	65769.01	1618.84	2.63	73.31	15.82	78.42	2.07	0.57	98.47	1.27	2.13	98.40	100.00	86227.81	90638.35
	2010	79.04	67.35	11.69	70306.23	1483.09	2.09	71.01	16.01	77.45	1.62	0.47	98.66	1.22	2.07	98.30	100.00	98391.53	90803.26
	2011	79.55	67.74	11.81	72670.96	1716.89	1.93	63.46	13.86	78.17	1.51	0.42	98.81	1.20	2.03	98.10	100.00	100891.32	90679.70
	2012	79.82	67.97	11.84	70396.82	2031.81	2.16	57.65	8.68	84.94	1.83	0.32	98.95	1.20	2.00	98.00	100.00	103155.12	90595.89
	2013	80.03	68.15	11.87	68899.49	2067.51	2.15	48.15	6.53	86.43	1.85	0.29	99.06	1.20	1.97	98.00	100.00	-	90541.17
	2014	80.01	68.16	11.84	67901.22	2106.36	2.19	48.15	6.86	85.75	1.88	0.31	99.16	1.23	1.95	98.00	100.00	-	90514.04
	2015	79.95	68.16	11.79	67277.24	-	-	-	-	-	-	-	99.24	1.28	1.93	98.00	100.00	-	90512.89
	2016	79.87	68.15	11.71	66410.76	-	-	-	-	-	-	-	99.32	1.40	-	-	-	-	90579.55
	2017	80.21	68.35	11.85	-	-	-	-	-	-	-	-	-	-	-	-	-	-	-
Republic of Congo	1995	53.13	45.31	7.82	2418.43	24.60	3.16	98.41	40.04	59.31	1.88	1.29	56.41	6.84	5.14	-	-	57957.63	91427.38
	1996	51.08	43.69	7.39	2452.14	26.02	2.86	98.19	39.14	60.13	1.72	1.14	56.83	6.79	5.13	-	-	51566.39	91440.65
	1997	52.44	44.78	7.66	2368.34	25.05	3.10	98.55	37.75	61.70	1.91	1.19	57.30	6.73	5.12	12.50	68.30	49792.18	91465.81
	1998	52.61	44.93	7.68	2387.56	24.68	3.74	98.76	40.15	59.34	2.22	1.52	57.77	6.66	5.11	12.60	68.60	32353.10	91498.86
	1999	53.04	45.29	7.75	2260.73	23.80	3.07	98.83	42.31	57.19	1.75	1.31	58.23	6.58	5.11	12.60	68.90	27868.58	91518.28
	2000	53.35	45.55	7.80	2364.61	22.01	2.13	98.92	42.01	57.53	1.22	0.90	58.70	6.50	5.10	12.70	69.20	28435.40	91557.81
	2001	53.58	45.80	7.78	2387.85	21.38	2.44	98.73	44.65	54.78	1.33	1.10	59.16	6.47	5.09	12.80	69.50	35332.08	91570.82
	2002	53.74	45.96	7.78	2430.29	22.74	2.45	98.94	46.99	52.51	1.29	1.16	59.62	6.42	5.08	12.90	70.10	24853.87	91587.05
	2003	54.01	46.23	7.78	2383.36	27.39	2.61	99.20	49.79	49.81	1.30	1.31	60.08	6.37	5.07	13.10	70.60	34876.59	91611.79
	2004	54.85	46.96	7.89	2395.82	34.81	2.56	98.76	45.82	53.60	1.37	1.19	60.53	6.30	5.05	13.20	71.20	34296.33	91636.12
	2005	55.34	47.37	7.97	2503.31	42.08	2.42	98.78	40.26	59.24	1.43	0.99	60.99	6.24	5.04	13.40	71.70	38914.47	91656.07
	2006	55.87	47.87	8.00	2573.51	50.89	2.37	98.75	36.57	62.97	1.49	0.88	61.44	6.21	5.03	13.60	72.20	37981.72	91649.75
	2007	56.13	48.15	7.98	2447.51	57.33	2.54	98.48	60.91	60.91	1.55	0.99	61.89	6.18	5.01	13.70	72.80	34160.31	91661.45
	2008	56.45	48.48	7.97	2496.50	64.52	2.09	97.96	39.77	59.40	1.24	0.85	62.34	6.14	5.00	13.90	73.30	31049.21	91665.15
	2009	56.88	48.88	8.00	2595.75	55.01	2.27	96.87	48.08	50.37	1.14	1.12	62.79	6.11	4.97	14.10	73.80	34611.28	91674.70
	2010	57.50	49.44	8.07	2737.34	67.71	2.29	94.22	37.24	60.47	1.39	0.91	63.23	6.06	4.95	14.30	74.30	35247.16	91695.87
	2011	58.01	49.92	8.09	2751.91	96.35	2.79	96.02	27.97	70.87	1.98	0.81	63.67	6.11	4.92	14.40	74.80	35504.46	91633.23

	2012	58.51	50.40	8.11	2782.11	126.89	3.98	96.02	19.97	79.20	3.15	0.83	64.10	6.14	4.88	14.60	75.30	35743.92	91555.11
	2013	59.31	51.11	8.20	2806.35	163.31	5.09	96.02	19.55	79.64	4.06	1.04	64.53	6.17	4.83	14.80	75.80	-	91485.87
	2014	60.10	51.81	8.29	2922.97	161.64	5.15	96.02	17.51	81.76	4.21	0.94	64.96	6.19	4.78	14.90	76.30	-	91420.40
	2015	60.91	52.52	8.38	2925.53	-	-	-	-	-	-	-	65.38	6.21	4.72	15.00	76.50	-	91348.67
	2016	61.56	53.11	8.45	2798.07	-	-	-	-	-	-	-	65.80	6.24	-	-	-	-	91280.50
	2017	62.37	53.31	9.06	-	-	-	-	-	-	-	-	-	-	-	-	-	-	-
Republic of Guinea-Bissau	1995	50.84	44.30	6.54	639.36	24.31	6.43	58.68	44.16	24.75	1.59	4.84	32.63	5.88	6.29	10.60	43.80	1701.07	88988.13
	1996	51.11	44.55	6.56	700.03	19.29	6.95	58.71	40.91	30.32	2.11	4.84	33.42	5.87	6.20	10.80	45.40	1693.06	88972.32
	1997	50.51	44.05	6.46	732.47	15.22	6.72	55.26	36.76	33.47	2.25	4.47	34.22	5.83	6.11	11.00	47.10	1709.15	88983.76
	1998	50.65	44.23	6.42	517.78	11.57	7.06	58.80	40.42	31.25	2.21	4.85	35.02	5.77	6.01	11.20	48.70	6512.36	88997.75
	1999	51.13	44.63	6.50	514.15	10.03	5.76	53.45	39.90	25.34	1.46	4.30	35.83	5.69	5.91	11.80	50.40	8396.17	88998.78
	2000	52.21	45.55	6.66	532.27	15.69	4.94	54.75	49.02	10.45	0.52	4.42	36.65	5.60	5.82	12.40	52.10	6791.44	89008.23
	2001	52.52	45.87	6.65	533.50	14.63	5.04	56.09	50.71	9.60	0.48	4.56	37.48	5.53	5.73	13.00	53.80	7464.87	89010.16
	2002	52.81	46.12	6.68	517.62	16.85	5.84	56.03	43.64	22.10	1.29	4.55	38.32	5.44	5.64	13.70	55.50	5926.64	89000.25
	2003	53.08	46.36	6.72	509.66	19.10	5.62	54.80	42.16	23.07	1.30	4.32	39.16	5.35	5.56	14.30	57.20	10897.16	89009.36
	2004	53.13	46.42	6.72	512.43	20.08	5.41	56.82	47.84	15.80	0.86	4.56	40.01	5.25	5.47	15.00	59.00	7123.66	89035.99
	2005	53.14	46.40	6.75	522.49	22.93	5.72	56.83	46.06	18.96	1.08	4.63	40.87	5.17	5.39	15.60	60.70	5582.04	89051.45
	2006	53.75	46.96	6.79	522.51	23.26	5.87	58.39	47.77	18.19	1.07	4.81	41.73	5.17	5.31	16.30	62.50	10458.94	89048.07
	2007	54.21	47.36	6.84	526.84	27.80	6.10	57.81	22.33	22.33	1.36	4.74	42.60	5.17	5.24	16.90	64.30	6842.50	89053.41
	2008	54.46	47.59	6.87	530.93	33.48	6.05	59.12	47.80	19.14	1.16	4.89	43.47	5.16	5.17	17.60	66.10	5808.09	89061.65
	2009	55.00	48.07	6.93	535.28	35.20	6.81	59.04	42.41	28.16	1.92	4.89	44.34	5.14	5.11	18.20	68.00	5046.76	89077.64
	2010	55.60	48.57	7.03	546.72	34.74	6.70	58.63	42.44	27.61	1.85	4.85	45.22	5.11	5.05	18.90	69.80	7536.36	89104.11
	2011	56.14	49.06	7.07	576.01	35.98	5.46	59.49	48.07	19.19	1.05	4.41	46.08	5.13	4.99	19.60	71.70	7571.18	89118.73
	2012	56.60	49.49	7.11	551.64	37.26	5.96	63.96	46.88	26.69	1.59	4.37	46.92	5.13	4.92	20.20	73.60	7604.73	89140.54
	2013	57.11	49.95	7.16	554.91	40.46	6.14	62.23	45.76	26.47	1.63	4.51	47.75	5.13	4.86	20.50	75.50	-	89176.87
	2014	57.53	50.33	7.20	545.90	37.28	5.59	62.23	49.49	20.47	1.15	4.45	48.55	5.15	4.78	20.70	77.40	-	89227.45
	2015	58.10	50.83	7.27	564.72	-	-	-	-	-	-	-	49.33	5.19	4.71	20.80	79.30	-	89262.35
	2016	58.75	51.39	7.37	582.37	-	-	-	-	-	-	-	50.09	5.29	-	-	-	-	89314.10
	2017	59.99	52.00	7.99	-	-	-	-	-	-	-	-	-	-	-	-	-	-	-

Republic of	1995	74.21	65.27	8.94	12055.23	452.96	3.67	85.05	53.13	37.53	1.38	2.29	78.24	8.42	1.63	100.00	90.60	453629.67	87366.13
	1996	74.67	65.62	9.05	12847.77	509.24	3.88	84.81	50.52	40.43	1.57	2.31	78.66	8.67	1.57	100.00	91.20	487344.75	87516.53
	1997	75.10	65.97	9.13	13481.63	469.23	3.87	83.43	47.59	42.96	1.66	2.21	78.91	8.94	1.52	100.00	91.80	513934.87	87692.59
	1998	75.39	66.22	9.18	12652.35	320.70	3.97	81.48	42.51	47.83	1.90	2.07	79.15	9.24	1.45	100.00	92.30	450493.85	87860.91
	1999	75.72	66.49	9.23	13983.41	437.41	4.20	82.53	42.54	48.45	2.04	2.17	79.38	9.58	1.41	100.00	92.90	489986.32	87996.70
	2000	76.05	66.81	9.24	15104.52	504.90	4.23	80.60	41.13	48.97	2.07	2.16	79.62	9.95	1.47	100.00	93.40	512819.94	88135.01
	2001	76.37	67.00	9.38	15667.38	535.74	4.76	80.19	35.90	55.23	2.63	2.13	79.94	10.37	1.30	100.00	94.00	522374.16	88309.77
	2002	76.79	67.31	9.48	16734.85	589.89	4.61	80.44	36.91	54.12	2.50	2.12	80.30	10.84	1.17	100.00	94.50	534839.28	88516.24
	2003	77.32	67.71	9.61	17136.66	700.36	4.93	80.09	38.33	52.14	2.57	2.36	80.65	11.33	1.18	100.00	95.10	546211.11	88730.98
	2004	77.91	68.16	9.75	17905.23	789.63	4.96	80.77	38.35	52.52	2.60	2.35	81.00	11.80	1.15	100.00	95.60	562450.96	88956.84
	2005	78.44	68.63	9.81	18568.36	994.17	5.33	80.50	37.92	52.89	2.82	2.51	81.35	12.26	1.08	100.00	96.10	561372.54	89193.95
	2006	78.90	68.99	9.90	19427.19	1204.22	5.76	80.47	36.50	54.64	3.15	2.61	81.53	12.73	1.12	100.00	96.60	565697.20	89306.37
	2007	79.30	69.23	10.07	20385.32	1383.03	5.99	79.40	54.78	54.78	3.28	2.71	81.63	13.19	1.25	100.00	97.10	575558.38	89393.71
	2008	79.66	69.51	10.15	20803.50	1260.85	6.16	78.71	35.71	54.63	3.36	2.79	81.73	13.63	1.19	100.00	97.60	594982.20	89521.13
	2009	79.99	69.76	10.23	20843.13	1220.65	6.66	78.92	34.39	56.43	3.76	2.90	81.84	14.11	1.15	100.00	97.60	594493.20	89682.83
	2010	80.25	70.03	10.21	22086.95	1505.02	6.79	78.42	33.92	56.74	3.86	2.94	81.94	14.63	1.23	100.00	97.60	628838.72	89838.46
	2011	80.53	70.20	10.33	22724.71	1650.46	6.83	78.06	34.40	55.93	3.82	3.01	82.04	15.15	1.24	100.00	97.60	650085.53	89937.35
	2012	80.74	70.39	10.35	23123.76	1714.86	7.01	77.33	34.85	54.93	3.85	3.16	82.14	15.73	1.30	100.00	97.60	668989.65	90074.91
	2013	80.96	70.57	10.39	23685.41	1870.13	7.20	77.09	35.21	54.33	3.91	3.29	82.25	16.36	1.19	100.00	-	-	90212.30
	2014	80.99	70.63	10.36	24323.57	2060.25	7.37	78.54	36.09	54.05	3.99	3.39	82.36	17.02	1.21	100.00	-	-	90351.86
	2015	81.02	70.68	10.34	24870.77	-	-	-	-	-	-	-	82.47	17.73	1.24	100.00	-	-	90490.89
	2016	81.00	70.76	10.24	25458.89	-	-	-	-	-	-	-	82.59	18.43	-	-	-	-	90637.28
	2017	82.60	71.68	10.92	-	-	-	-	-	-	-	-	-	-	-	-	-	-	-
Republic of	1995	60.46	50.38	10.09	1032.52	39.66	4.20	95.62	64.72	32.32	1.36	2.85	23.76	6.53	7.53	31.60	63.30	17884.47	89232.81
Yemen	1996	60.80	50.69	10.11	1041.70	23.33	4.11	95.30	57.21	39.97	1.64	2.47	24.25	6.41	7.25	33.20	62.60	18386.30	89187.58
	1997	61.26	51.09	10.17	1061.49	20.31	4.26	95.19	52.77	44.56	1.90	2.36	24.74	6.27	6.99	34.70	61.90	19676.02	89175.99
	1998	61.67	51.45	10.22	1092.80	21.44	4.98	93.16	49.66	46.70	2.32	2.65	25.24	6.13	6.74	36.30	61.30	20869.79	89166.70
	1999	62.15	51.86	10.29	1102.61	21.51	4.28	94.94	47.77	49.69	2.13	2.15	25.75	5.97	6.51	37.80	60.60	22895.70	89173.72
	2000	62.61	52.26	10.35	1138.25	25.27	4.14	94.57	43.49	54.01	2.24	1.90	26.27	5.82	6.31	39.40	59.90	24225.18	89176.10

	2001	63.07	52.66	10.40	1148.43	26.62	4.34	94.79	44.63	52.92	2.29	2.04	26.79	5.72	6.13	41.00	59.20	25210.42	89175.88
	2002	63.53	53.08	10.45	1160.24	27.44	4.22	94.84	46.68	50.78	2.14	2.08	27.32	5.61	5.95	42.50	58.50	26580.10	89181.60
	2003	63.99	53.48	10.51	1170.14	35.01	5.00	96.18	54.25	43.60	2.18	2.82	27.85	5.49	5.78	44.10	57.90	28983.22	89207.25
	2004	64.50	53.93	10.57	1182.90	38.38	4.90	97.28	60.46	37.85	1.85	3.04	28.39	5.38	5.60	45.70	57.20	29514.28	89242.34
	2005	64.96	54.35	10.61	1214.70	42.54	4.58	97.95	64.78	33.86	1.55	3.03	28.94	5.26	5.43	47.30	56.50	32666.19	89282.97
	2006	65.51	54.80	10.71	1219.01	52.10	4.82	92.47	63.26	31.59	1.52	3.30	29.49	5.18	5.25	48.80	55.70	34959.45	89336.66
	2007	65.83	55.04	10.79	1225.47	58.09	4.92	98.44	29.75	29.75	1.46	3.45	30.04	5.10	5.09	50.40	55.00	36225.63	89355.69
	2008	66.39	55.48	10.90	1235.81	69.69	5.12	98.53	68.40	30.58	1.57	3.55	30.60	5.02	4.94	52.00	54.30	36800.23	89349.36
	2009	67.02	55.99	11.03	1249.03	65.91	5.32	98.58	73.79	25.14	1.34	3.98	31.17	4.96	4.80	52.30	54.50	37027.20	89316.41
	2010	67.49	56.39	11.09	1309.23	67.75	5.17	98.62	73.84	25.12	1.30	3.87	31.73	4.92	4.67	52.70	54.60	38865.29	89278.49
	2011	67.66	56.55	11.11	1112.35	64.65	5.04	98.63	72.21	26.79	1.35	3.69	32.30	4.94	4.55	53.00	54.70	39949.08	89227.49
	2012	67.88	56.76	11.12	1108.90	73.91	5.73	98.66	71.61	27.41	1.57	4.16	32.87	4.97	4.44	53.30	54.90	40924.63	89210.34
	2013	68.33	57.15	11.18	1132.10	78.52	5.78	98.67	74.58	24.41	1.41	4.37	33.45	5.01	4.33	-	-	-	89253.55
	2014	68.42	57.26	11.16	1101.12	79.94	5.64	98.68	76.42	22.56	1.27	4.37	34.03	5.03	4.22	-	-	-	89348.21
	2015	67.15	56.29	10.85	772.03	-	-	-	-	-	-	-	34.61	5.05	4.10	-	-	-	89472.89
	2016	66.68	56.00	10.68	679.67	-	-	-	-	-	-	-	35.19	5.09	-	-	-	-	89597.65
	2017	68.06	56.73	11.34	-	-	-	-	-	-	-	-	-	-	-	-	-	-	-
Romania	1995	69.28	60.56	8.72	4911.07	53.29	3.22	100.00	25.46	74.54	2.40	0.82	53.77	17.95	1.33	72.30	79.80	177197.36	92821.78
	1996	69.05	60.40	8.66	5115.83	52.66	3.21	100.00	26.78	73.22	2.35	0.86	53.62	18.44	1.30	72.60	80.90	181831.13	92826.65
	1997	69.25	60.57	8.68	4883.49	66.33	4.17	100.00	20.62	79.38	3.31	0.86	53.46	18.88	1.32	72.90	82.00	167308.77	92826.55
	1998	69.96	61.14	8.81	4791.46	69.67	3.75	100.00	24.42	75.58	2.84	0.92	53.31	19.29	1.32	73.20	83.10	147443.81	92831.76
	1999	70.78	61.81	8.98	4779.72	67.53	4.20	100.00	20.34	79.66	3.34	0.85	53.16	19.68	1.30	73.50	84.20	129620.60	92852.48
	2000	71.12	62.12	9.00	4900.54	72.61	4.33	100.00	18.80	81.20	3.51	0.81	53.00	20.06	1.31	73.80	85.30	132627.54	92887.08
	2001	71.05	62.06	8.99	5247.29	79.16	4.36	100.00	18.88	81.12	3.53	0.82	52.85	20.59	1.27	74.10	86.50	137737.59	92926.67
	2002	71.21	62.22	9.00	5621.29	96.66	4.57	100.00	17.81	82.19	3.76	0.81	52.78	21.11	1.27	74.40	87.60	135160.39	92955.47
	2003	71.53	62.48	9.05	5974.77	146.70	5.30	97.43	14.81	84.80	4.49	0.81	52.91	21.57	1.30	74.80	88.80	141128.16	92982.70
	2004	72.00	62.85	9.16	6511.17	192.79	5.43	96.81	24.13	74.60	4.05	1.35	53.04	21.91	1.33	75.20	90.00	139881.57	92993.97
	2005	72.36	63.20	9.16	6824.82	255.29	5.47	96.41	18.46	80.43	4.40	1.05	53.17	22.10	1.40	75.60	91.10	139578.17	93011.69
	2006	72.79	63.54	9.25	7418.42	294.14	5.06	98.16	19.79	79.66	4.03	1.02	53.31	22.37	1.42	75.90	92.30	144382.55	93021.92

	2007	73.18	63.78	9.40	8045.58	423.85	5.21	97.40	82.11	82.11	4.28	0.92	53.44	22.51	1.45	76.30	93.50	141043.06	93035.00
	2008	73.10	63.72	9.38	8872.78	541.47	5.33	98.09	17.62	81.99	4.37	0.96	53.57	22.58	1.60	76.70	94.60	136834.94	93038.14
	2009	73.26	63.84	9.42	8314.74	456.81	5.56	97.86	20.52	79.00	4.39	1.17	53.70	22.69	1.66	77.10	95.80	119648.21	93056.47
	2010	73.59	64.18	9.41	8297.48	482.95	5.83	98.16	19.21	80.35	4.69	1.14	53.83	22.89	1.59	77.50	96.90	117930.70	93123.06
	2011	74.43	64.72	9.71	8426.47	507.68	5.53	97.79	20.32	79.22	4.38	1.15	53.96	23.19	1.47	77.90	98.00	126194.42	93171.91
	2012	74.54	64.83	9.72	8518.32	467.64	5.48	96.99	19.14	80.27	4.40	1.08	54.09	23.56	1.52	78.30	99.00	121762.23	93207.23
	2013	74.74	64.99	9.75	8851.96	535.48	5.60	97.08	18.66	80.78	4.52	1.08	54.24	24.02	1.41	78.60	100.00	-	93226.76
	2014	74.75	65.02	9.73	9158.52	556.81	5.57	96.30	18.87	80.40	4.47	1.09	54.39	24.55	1.52	79.00	100.00	-	93228.62
	2015	75.09	65.28	9.81	9567.39	-	-	-	-	-	-	-	54.56	25.15	1.52	79.10	100.00	-	93236.02
	2016	75.23	65.54	9.69	10062.43	-	-	-	-	-	-	-	54.75	25.89	-	-	-	-	93255.43
	2017	75.18	64.71	10.47	-	-	-	-	-	-	-	-	-	-	-	-	-	-	-
Rwanda	1995	44.58	39.16	5.42	280.50	9.34	4.27	41.25	25.40	38.43	1.64	2.63	9.84	3.95	6.17	40.20	62.40	3231.68	86862.14
	1996	45.94	40.38	5.56	306.58	10.05	4.44	41.20	23.77	42.29	1.88	2.56	11.37	4.37	6.05	41.60	63.40	3420.40	87017.52
	1997	45.31	39.88	5.42	327.24	12.57	4.42	41.20	23.91	41.96	1.85	2.56	12.47	4.82	5.94	43.00	64.20	3682.84	87176.88
	1998	46.63	41.01	5.62	329.12	14.34	5.08	41.16	20.42	50.38	2.56	2.52	13.24	5.32	5.84	44.20	64.90	5454.04	87307.40
	1999	48.00	42.17	5.84	319.06	10.96	4.57	40.97	22.31	45.56	2.08	2.49	14.06	5.78	5.74	45.40	65.60	4837.27	87406.99
	2000	49.44	43.33	6.11	327.10	9.13	4.22	40.73	24.78	39.17	1.65	2.57	14.93	6.15	5.64	46.60	66.30	4900.33	87453.37
	2001	50.66	44.41	6.25	341.99	8.81	4.38	45.98	23.53	48.83	2.14	2.24	15.83	5.95	5.54	47.80	67.00	3924.02	87403.72
	2002	52.62	46.10	6.52	377.71	8.20	4.18	51.72	24.69	52.27	2.18	1.99	16.78	5.65	5.45	48.90	67.70	4806.29	87320.57
	2003	54.17	47.44	6.73	379.64	13.48	6.34	36.81	17.40	52.74	3.35	3.00	17.60	5.33	5.35	50.00	68.40	4793.81	87223.92
	2004	55.95	48.97	6.98	401.41	14.78	6.24	40.90	22.06	46.05	2.88	3.37	18.43	5.06	5.24	51.10	69.10	5635.40	87137.44
	2005	57.80	50.60	7.20	430.63	19.58	6.83	43.72	23.25	46.82	3.20	3.63	19.28	4.86	5.14	52.20	69.80	4963.96	87085.07
	2006	59.60	52.23	7.36	459.41	33.75	10.02	44.32	22.28	49.72	4.98	5.04	20.17	4.79	5.02	53.30	70.40	5021.66	87037.24
	2007	61.20	53.57	7.64	482.14	33.81	8.49	44.57	44.52	44.52	3.78	4.71	21.09	4.77	4.90	54.30	71.10	4904.62	86996.00
	2008	62.60	54.79	7.80	521.55	37.69	7.66	45.46	28.67	36.94	2.83	4.83	22.03	4.78	4.77	55.30	71.80	5183.74	86983.78
	2009	63.43	55.53	7.90	539.38	38.14	7.20	45.73	30.48	33.35	2.40	4.80	22.99	4.79	4.64	56.30	72.40	5967.06	87013.22
	2010	64.72	56.75	7.96	563.49	43.79	7.91	44.59	28.09	37.02	2.93	4.98	23.95	4.79	4.52	57.20	73.10	6547.47	87039.62
	2011	65.48	57.34	8.14	591.82	46.79	7.71	45.47	28.89	36.46	2.81	4.90	24.92	4.82	4.39	58.20	73.70	6620.25	87009.96
	2012	66.13	57.91	8.21	627.85	51.24	7.68	45.74	28.84	36.94	2.84	4.84	25.89	4.84	4.27	59.10	74.30	6689.95	87014.66

	2013	66.62	58.35	8.27	640.95	52.20	7.69	44.57	27.61	38.05	2.93	4.76	26.87	4.86	4.16	60.00	74.90	-	87029.25
	2014	67.06	58.73	8.32	672.75	52.48	7.53	45.44	28.13	38.10	2.87	4.66	27.84	4.91	4.06	60.80	75.50	-	87053.08
	2015	67.43	59.06	8.37	714.54	-	-	-	-	-	-	-	28.81	4.98	3.97	61.60	76.10	-	87084.28
	2016	67.77	59.47	8.30	738.64	-	-	-	-	-	-	-	29.78	5.10	-	-	-	-	87115.85
	2017	68.46	59.65	8.81	-	-	-	-	-	-	-	-	-	-	-	-	-	-	-
Saint Lucia	1995	73.14	63.78	9.36	6857.67	232.15	6.09	98.27	49.12	50.02	3.04	3.04	28.77	13.38	2.89	80.60	93.40	334.15	90646.74
	1996	73.47	64.09	9.39	6963.56	268.64	7.07	98.48	49.13	50.11	3.54	3.53	28.57	13.23	2.77	81.10	93.50	347.70	90737.09
	1997	73.79	64.37	9.42	6810.47	256.02	6.40	98.48	52.11	47.09	3.01	3.38	28.37	13.08	2.64	81.60	93.70	330.53	90844.89
	1998	73.97	64.52	9.44	7145.33	220.79	5.14	97.58	38.91	60.12	3.09	2.05	28.17	12.93	2.50	82.10	93.90	-	90950.88
	1999	74.13	64.66	9.46	7242.27	247.23	5.54	97.95	43.35	55.74	3.09	2.45	27.97	12.77	2.35	82.60	94.10	-	91050.36
	2000	74.17	64.71	9.45	7144.88	275.06	5.53	98.00	47.03	52.01	2.88	2.65	27.77	12.58	2.20	83.10	94.20	404.94	91167.63
	2001	74.07	64.66	9.41	6808.90	283.97	6.10	98.08	45.91	53.19	3.24	2.85	27.47	12.34	2.06	83.60	94.40	408.26	91273.42
	2002	74.08	64.70	9.38	6774.62	292.94	6.33	98.11	47.43	51.66	3.27	3.06	26.33	12.08	1.93	84.10	94.50	412.31	91379.37
	2003	73.92	64.60	9.32	7039.88	304.04	6.10	98.11	50.79	48.23	2.94	3.16	25.22	11.82	1.83	84.60	94.60	427.33	91501.58
	2004	73.90	64.60	9.29	7518.65	335.74	6.20	98.42	51.62	47.55	2.95	3.25	24.13	11.55	1.74	85.10	94.80	470.33	91625.78
	2005	74.13	64.80	9.33	7415.02	353.27	6.25	98.47	52.83	46.35	2.90	3.35	23.09	11.28	1.68	85.70	94.90	473.07	91749.58
	2006	74.42	65.05	9.38	7840.60	402.78	6.37	98.23	50.21	48.88	3.11	3.26	22.07	11.45	1.63	86.20	95.10	493.09	91896.52
	2007	74.76	65.29	9.47	7906.70	457.89	6.81	98.30	43.74	43.74	2.98	3.83	21.08	11.68	1.60	86.80	95.20	512.25	92044.78
	2008	75.13	65.58	9.56	8221.77	504.18	7.43	98.42	51.11	48.07	3.57	3.86	20.13	11.97	1.57	87.30	95.30	533.67	92193.59
	2009	75.54	65.88	9.66	8070.11	545.27	8.12	98.62	45.15	54.22	4.40	3.72	19.21	12.33	1.56	87.90	95.50	534.36	92343.37
	2010	75.41	65.76	9.65	8007.70	567.88	8.10	98.62	49.48	49.83	4.03	4.06	18.45	12.79	1.54	88.50	95.60	563.92	92488.50
	2011	75.90	66.13	9.77	8220.79	545.53	7.58	98.62	56.19	43.02	3.26	4.32	18.45	12.84	1.52	89.00	95.80	582.34	92558.79
	2012	76.03	66.24	9.79	8119.68	601.16	8.35	98.69	48.95	50.40	4.21	4.14	18.45	12.92	1.51	89.50	96.00	598.77	92629.43
	2013	76.14	66.34	9.81	8095.14	575.50	7.85	98.69	54.26	45.02	3.54	4.32	18.46	13.02	1.50	90.00	96.20	-	92697.16
	2014	76.04	66.27	9.77	7987.53	499.57	6.72	98.14	45.57	53.57	3.60	3.12	18.48	13.13	1.48	90.50	96.30	-	92756.72
	2015	76.09	66.31	9.78	8113.53	-	-	-	-	-	-	-	18.50	13.26	1.47	90.50	96.30	-	92826.72
	2016	76.13	66.38	9.75	8151.63	-	-	-	-	-	-	-	18.54	13.43	-	-	-	-	92892.06
	2017	75.54	66.33	9.21	-	-	-	-	-	-	-	-	-	-	-	-	-	-	-
Saint	1995	70.42	61.74	8.68	4159.20	165.70	6.72	100.00	43.77	56.23	3.78	2.94	43.38	11.31	2.70	68.20	90.80	188.11	91354.31

Vincent and	1996	70.32	61.67	8.65	4214.57	175.31	6.73	100.00	44.09	55.91	3.76	2.97	43.74	11.28	2.64	69.20	91.30	187.61	91292.43
	1997	70.53	61.83	8.70	4365.19	202.92	7.48	100.00	47.78	52.22	3.91	3.57	44.10	11.23	2.58	70.20	91.90	172.73	91246.85
	1998	70.54	61.83	8.71	4547.16	200.42	6.81	100.00	50.34	49.66	3.38	3.43	44.46	11.19	2.51	71.20	92.40	213.68	91213.23
	1999	70.35	61.67	8.68	4672.25	198.97	6.47	100.00	46.51	53.49	3.46	3.01	44.82	11.19	2.44	72.20	92.90	226.86	91205.44
	2000	70.15	61.49	8.66	4747.96	136.94	3.73	100.00	17.73	82.27	3.07	0.66	45.19	11.24	2.38	73.20	93.50	222.54	91207.07
	2001	70.48	61.76	8.72	4825.10	145.15	3.64	100.00	19.05	80.95	2.95	0.69	45.55	11.22	2.32	74.20	94.00	223.72	91307.62
	2002	70.43	61.73	8.71	5122.71	167.90	3.93	100.00	17.80	82.20	3.23	0.70	45.91	11.23	2.27	75.10	94.50	234.31	91414.35
	2003	70.77	62.00	8.77	5506.25	173.67	3.91	100.00	17.92	82.08	3.21	0.70	46.27	11.26	2.23	76.10	95.10	240.08	91513.54
	2004	70.88	62.09	8.79	5724.06	178.98	3.72	100.00	18.80	81.20	3.02	0.70	46.63	11.25	2.20	76.10	95.10	259.91	91621.16
	2005	71.08	62.25	8.83	5856.49	185.30	3.66	100.00	19.13	80.87	2.96	0.70	46.99	11.17	2.17	76.10	95.10	261.00	91709.96
	2006	71.18	62.35	8.83	6297.32	217.80	3.88	100.00	18.02	81.98	3.18	0.70	47.35	11.02	2.15	76.10	95.10	267.91	91773.43
	2007	71.33	62.46	8.86	6189.77	245.91	3.92	100.00	82.14	82.14	3.22	0.70	47.71	10.79	2.13	76.10	95.10	276.97	91835.49
	2008	71.50	62.60	8.90	6595.31	299.19	4.70	100.00	15.97	84.03	3.95	0.75	48.07	10.53	2.11	-	95.10	284.79	91889.41
	2009	71.57	62.65	8.92	6451.66	316.93	5.13	100.00	15.59	84.41	4.33	0.80	48.43	10.29	2.09	-	95.10	-	91946.67
	2010	71.52	62.60	8.92	6231.77	293.45	4.71	100.00	18.05	81.95	3.86	0.85	48.79	10.09	2.07	-	95.10	301.56	92000.41
	2011	71.54	62.61	8.93	6204.16	309.10	4.99	100.00	18.01	81.99	4.09	0.90	49.14	10.14	2.05	-	95.10	310.88	92033.34
	2012	71.48	62.57	8.92	6290.57	538.44	8.48	100.00	48.06	51.94	4.40	4.07	49.50	10.24	2.02	-	95.10	319.22	92060.78
	2013	71.12	62.27	8.85	6406.30	342.49	5.21	100.00	17.28	82.72	4.31	0.90	49.85	10.37	2.00	-	95.10	-	92104.47
	2014	71.32	62.44	8.88	6483.32	575.06	8.63	100.00	49.19	50.81	4.39	4.25	50.20	10.54	1.98	-	95.10	-	92142.18
	2015	71.47	62.56	8.91	6566.09	-	-	-	-	-	-	-	50.55	10.75	1.95	-	95.10	-	92204.07
	2016	71.64	62.70	8.94	6676.62	-	-	-	-	-	-	-	50.90	11.00	-	-	-	-	92276.37
	2017	72.29	63.46	8.83	-	-	-	-	-	-	-	-	-	-	-	-	-	-	-
Samoa	1995	69.60	60.97	8.63	2357.15	60.91	4.61	84.60	22.36	73.57	3.39	1.22	21.53	7.56	4.75	92.50	91.10	279.86	84879.81
	1996	69.77	61.11	8.65	2509.76	61.72	4.17	84.06	23.44	72.11	3.01	1.16	21.62	7.74	4.69	92.40	91.60	288.65	84884.79
	1997	69.92	61.25	8.68	2512.49	73.93	4.64	85.35	23.97	71.92	3.34	1.30	21.71	7.90	4.64	92.40	92.00	306.32	84886.07
	1998	70.10	61.39	8.71	2556.01	75.16	5.17	84.80	20.35	76.00	3.93	1.24	21.80	8.02	4.59	92.30	92.40	299.83	84879.96
	1999	70.18	61.45	8.72	2600.24	83.02	5.60	85.53	20.28	76.29	4.27	1.33	21.89	8.11	4.54	92.30	92.90	305.65	84857.89
	2000	70.30	61.55	8.74	2766.53	79.08	5.31	85.13	19.16	77.49	4.12	1.20	21.98	8.16	4.50	92.20	93.30	318.53	84895.24
	2001	70.45	61.68	8.77	2942.41	79.07	5.16	82.41	20.94	74.59	3.85	1.31	22.07	8.27	4.48	92.20	93.80	324.44	84942.86

	2002	70.62	61.83	8.80	3052.56	84.10	5.01	80.19	17.15	78.62	3.94	1.07	21.94	8.38	4.46	92.10	94.20	314.98	84984.81
	2003	70.77	61.95	8.82	3171.01	95.59	4.71	81.37	18.28	77.53	3.65	1.06	21.70	8.50	4.45	92.10	94.60	314.08	85029.47
	2004	70.91	62.06	8.85	3296.90	107.44	4.45	63.20	12.08	80.88	3.60	0.85	21.46	8.61	4.46	92.00	95.00	312.54	85093.88
	2005	71.06	62.18	8.87	3412.03	115.60	4.47	64.06	12.61	80.31	3.59	0.88	21.22	8.71	4.46	92.00	95.50	307.21	85148.23
	2006	71.20	62.30	8.90	3456.82	130.65	4.68	58.01	9.32	83.94	3.93	0.75	20.98	8.76	4.46	91.90	95.90	326.16	85177.99
	2007	71.24	62.32	8.92	3651.35	164.37	5.25	63.72	85.89	85.89	4.51	0.74	20.75	8.81	4.45	91.80	96.30	326.94	85222.13
	2008	71.34	62.40	8.95	3663.28	152.58	4.52	62.38	9.59	84.63	3.82	0.69	20.52	8.87	4.43	91.80	96.80	330.24	85260.89
	2009	69.27	60.66	8.60	3462.61	160.04	5.04	62.60	8.71	86.08	4.34	0.70	20.30	8.92	4.39	91.70	97.20	330.71	85324.05
	2010	71.39	62.43	8.96	3453.43	201.42	5.70	62.60	7.90	87.39	4.99	0.72	20.08	8.97	4.34	91.70	97.60	340.98	85404.94
	2011	71.39	62.43	8.96	3624.57	235.76	5.80	62.60	7.52	87.99	5.10	0.70	19.86	9.04	4.28	91.60	98.10	348.88	85472.67
	2012	71.30	62.36	8.94	3609.74	213.57	5.02	62.60	8.46	86.48	4.34	0.68	19.64	9.09	4.21	91.60	98.50	356.09	85604.60
	2013	71.52	62.56	8.97	3510.92	288.79	6.91	62.60	6.26	89.99	6.22	0.69	19.44	9.12	4.15	91.50	99.00	-	85698.02
	2014	71.58	62.61	8.97	3524.60	301.10	7.22	62.60	5.88	90.60	6.54	0.68	19.26	9.19	4.09	91.50	99.00	-	85823.14
	2015	71.66	62.67	8.99	3555.06	-	-	-	-	-	-	-	19.10	9.29	4.03	91.50	99.00	-	85966.62
	2016	71.89	62.87	9.02	3782.40	-	-	-	-	-	-	-	18.96	9.47	-	-	-	-	86137.04
	2017	72.88	63.26	9.62	-	-	-	-	-	-	-	-	-	-	-	-	-	-	-
Sao Tome and Principe	1995	63.77	55.60	8.17	-	59.96	7.64	71.80	45.45	36.70	2.81	4.84	48.63	9.40	5.55	18.20	74.00	107.21	88279.61
	1996	63.61	55.52	8.10	-	68.40	6.81	74.16	52.73	28.89	1.97	4.84	49.59	9.36	5.49	18.30	74.20	107.84	88261.37
	1997	63.64	55.58	8.07	-	55.87	8.32	65.18	42.15	35.33	2.94	5.38	50.55	9.33	5.44	18.40	74.40	111.49	88237.93
	1998	63.78	55.72	8.07	-	38.23	7.36	75.84	45.85	39.54	2.91	4.45	51.51	9.30	5.38	18.60	74.50	104.97	88215.48
	1999	64.16	56.04	8.12	-	46.37	8.49	71.15	38.83	45.42	3.86	4.63	52.47	9.28	5.33	19.70	76.40	109.36	88213.43
	2000	64.54	56.36	8.18	854.42	46.80	8.86	76.13	43.27	43.16	3.82	5.04	53.42	9.22	5.28	20.90	78.20	206.90	88232.63
	2001	64.97	56.75	8.23	862.90	53.02	10.28	64.26	40.55	36.90	3.79	6.49	54.38	9.16	5.23	22.10	80.00	187.96	88204.83
	2002	65.30	57.05	8.26	863.49	57.84	10.21	61.65	38.43	37.67	3.85	6.36	55.33	9.06	5.17	23.30	81.80	190.50	88212.65
	2003	65.74	57.42	8.32	898.65	74.33	11.12	70.35	32.14	54.31	6.04	5.08	56.25	8.93	5.12	24.50	83.50	210.28	88206.01
	2004	66.03	57.68	8.35	911.01	71.79	9.94	76.16	36.78	51.71	5.14	4.80	57.14	8.74	5.07	25.70	85.30	170.01	88207.91
	2005	66.13	57.75	8.38	952.68	81.79	10.04	73.06	34.53	52.73	5.29	4.74	58.01	8.50	5.02	26.90	87.00	177.25	88223.79
	2006	66.84	58.38	8.46	1015.40	69.14	7.84	77.88	52.64	32.41	2.54	5.30	58.85	8.24	4.97	28.10	88.70	155.28	88223.73
	2007	67.31	58.78	8.54	1024.18	70.14	7.44	73.80	28.74	28.74	2.14	5.30	59.66	7.95	4.93	29.40	90.40	169.71	88219.95

	2008	67.70	59.10	8.60	1082.71	69.40	5.66	62.56	44.97	28.12	1.59	4.07	60.43	7.62	4.88	30.60	92.10	175.96	88211.69
	2009	68.44	59.73	8.72	1083.66	86.35	6.65	48.10	29.97	37.69	2.51	4.15	61.19	7.29	4.84	31.80	93.70	177.02	88203.91
	2010	68.91	60.12	8.78	1129.75	66.50	5.24	57.40	29.62	48.41	2.54	2.70	61.91	6.96	4.79	33.00	95.30	185.45	88193.69
	2011	69.29	60.45	8.84	1152.90	81.22	5.44	46.32	24.15	47.87	2.61	2.84	62.60	6.66	4.74	34.30	97.00	190.74	88191.97
	2012	69.29	60.50	8.79	1162.54	133.62	8.60	17.14	12.20	28.85	2.48	6.12	63.27	6.37	4.69	34.40	97.00	195.49	88204.08
	2013	69.73	60.87	8.86	1191.44	182.78	9.76	16.25	10.28	36.75	3.59	6.17	63.90	6.10	4.64	34.50	97.00	-	88228.87
	2014	70.03	61.13	8.90	1241.00	165.55	8.35	19.72	11.19	43.24	3.61	4.74	64.51	5.84	4.58	34.60	97.10	-	88257.86
	2015	70.32	61.38	8.93	1260.32	-	-	-	-	-	-	-	65.09	5.61	4.52	34.70	97.10	-	88306.12
	2016	70.60	61.65	8.95	1283.89	-	-	-	-	-	-	-	65.65	5.45	-	-	-	-	88369.89
	2017	69.93	60.93	9.00	-	-	-	-	-	-	-	-	-	-	-	-	-	-	-
Saudi Arabia	1995	72.69	62.31	10.39	18648.86	221.29	2.93	72.28	34.25	52.62	1.54	1.39	78.67	5.26	4.98	93.90	93.30	262037.70	89818.10
	1996	72.73	62.38	10.34	18744.78	239.01	2.93	71.47	32.06	55.14	1.62	1.32	78.91	5.21	4.75	94.40	93.70	276181.97	89913.23
	1997	72.67	62.39	10.28	18588.30	251.94	3.03	70.97	29.90	57.87	1.75	1.28	79.15	5.16	4.53	95.00	94.00	280673.77	89994.40
	1998	72.63	62.41	10.22	18763.59	237.86	3.31	72.03	29.72	58.74	1.95	1.37	79.38	5.13	4.33	95.60	94.30	292876.65	90072.92
	1999	72.63	62.46	10.17	17690.92	316.80	4.10	69.04	21.82	68.39	2.80	1.30	79.62	5.11	4.14	96.20	94.70	295772.02	90145.67
	2000	72.85	62.68	10.17	18263.23	373.09	4.24	66.07	18.46	72.06	3.05	1.18	79.85	5.10	3.97	96.80	95.00	310713.50	90218.53
	2001	72.98	62.81	10.17	17585.39	373.30	4.49	65.68	18.04	72.54	3.26	1.23	80.08	5.01	3.83	97.30	95.40	321603.52	90300.50
	2002	73.14	62.97	10.17	16619.43	356.18	4.28	66.91	18.63	72.16	3.09	1.19	80.30	4.91	3.71	97.90	95.70	336300.49	90418.72
	2003	73.31	63.14	10.17	17954.95	369.17	4.02	65.51	18.29	72.07	2.90	1.12	80.53	4.83	3.60	98.50	96.00	355029.43	90554.94
	2004	73.50	63.32	10.18	18822.73	385.49	3.58	63.65	18.15	71.48	2.56	1.02	80.75	4.75	3.51	99.10	96.40	-	90707.74
	2005	73.74	63.57	10.17	19309.31	453.80	3.42	59.96	16.49	72.49	2.48	0.94	80.98	4.69	3.42	99.70	96.70	-	90863.40
	2006	73.98	63.79	10.19	19304.55	526.31	3.55	61.34	15.70	74.41	2.64	0.91	81.20	4.62	3.33	100.00	97.00	-	90990.95
	2007	74.26	64.00	10.26	19136.16	556.16	3.49	60.84	71.90	71.90	2.51	0.98	81.43	4.57	3.24	100.00	97.00	419853.00	91113.90
	2008	74.53	64.23	10.30	19792.72	557.87	2.87	58.67	20.05	65.83	1.89	0.98	81.65	4.52	3.15	100.00	97.00	446765.12	91244.30
	2009	74.72	64.40	10.32	18861.11	639.74	4.09	60.65	19.27	68.23	2.79	1.30	81.87	4.48	3.06	100.00	97.00	471266.33	91377.34
	2010	74.96	64.64	10.32	19259.59	655.07	3.49	56.44	19.84	64.85	2.27	1.23	82.08	4.43	2.96	100.00	97.00		91519.42
	2011	75.18	64.80	10.38	20575.50	829.25	3.57	54.34	16.30	70.00	2.50	1.07	82.30	4.36	2.87	100.00	97.00	514967.26	91667.47
	2012	75.42	65.01	10.41	21056.35	961.34	3.86	54.98	15.81	71.24	2.75	1.11	82.51	4.29	2.78	100.00	97.00	-	91819.10
	2013	75.90	65.40	10.50	21005.01	1052.10	4.25	55.51	14.97	73.03	3.10	1.14	82.72	4.24	2.70	100.00	97.00	-	91981.48

	2014	76.37	65.77	10.61	21183.46	1147.45	4.68	56.17	14.31	74.52	3.49	1.19	82.93	4.24	2.64	100.00	97.00	-	92138.38
	2015	76.52	65.88	10.65	21507.96	-	-	-	-	-	-	-	83.13	4.30	2.58	100.00	97.00	-	92296.79
	2016	77.10	66.38	10.72	21395.36	-	-	-	-	-	-	-	83.33	4.44	-	-	-	-	92434.20
	2017	76.86	66.32	10.54	-	-	-	-	-	-	-	-	-	-	-	-	-	-	-
Senegal	1995	58.36	50.85	7.51	810.57	22.04	3.93	95.14	64.19	32.53	1.28	2.65	39.62	6.07	6.00	37.90	63.70	12144.98	88284.62
	1996	58.56	51.06	7.51	805.92	23.14	4.08	93.69	59.64	36.33	1.48	2.60	39.77	6.11	5.88	38.40	64.40	12591.43	88299.89
	1997	58.81	51.30	7.51	811.00	20.09	3.94	93.21	62.83	32.60	1.28	2.66	39.91	6.13	5.77	38.90	65.20	13138.66	88316.38
	1998	58.97	51.46	7.51	838.61	21.25	3.97	92.65	62.02	33.06	1.31	2.65	40.06	6.13	5.67	39.30	65.90	44963.52	88363.50
	1999	59.10	51.60	7.50	870.79	24.01	4.49	92.47	56.61	38.78	1.74	2.75	40.20	6.12	5.57	39.80	66.70	56357.77	88404.75
	2000	59.66	52.08	7.57	877.00	21.98	4.63	91.60	54.11	40.93	1.90	2.74	40.35	6.11	5.47	40.30	67.40	48632.88	88439.73
	2001	60.01	52.40	7.61	894.51	24.00	4.98	91.07	52.01	42.89	2.14	2.84	40.49	6.10	5.39	40.70	68.20	58818.34	88437.26
	2002	60.22	52.58	7.64	877.65	26.00	5.06	90.42	55.59	38.52	1.95	3.11	40.64	6.09	5.31	41.20	68.90	55038.56	88435.28
	2003	60.98	53.22	7.76	912.25	34.70	5.40	89.73	54.97	38.74	2.09	3.31	40.78	6.06	5.25	41.70	69.60	34578.80	88434.52
	2004	61.39	53.57	7.82	940.69	41.60	5.68	88.50	55.49	37.30	2.12	3.56	40.94	6.02	5.20	42.10	70.40	49186.65	88411.45
	2005	61.74	53.87	7.87	967.50	41.37	5.35	77.37	34.27	55.70	2.98	2.37	41.12	5.97	5.16	42.60	71.10	50288.84	88402.96
	2006	62.17	54.26	7.91	965.11	44.00	5.44	76.72	32.32	57.86	3.15	2.29	41.31	5.96	5.13	43.10	71.80	58446.31	88369.89
	2007	62.69	54.69	7.99	985.75	44.79	4.72	78.37	49.40	49.40	2.33	2.39	41.52	5.93	5.11	43.60	72.60	47429.89	88331.73
	2008	63.20	55.13	8.07	994.38	54.02	4.94	76.03	37.09	51.21	2.53	2.41	41.74	5.89	5.10	44.10	73.30	51876.51	88316.50
	2009	63.64	55.50	8.14	990.32	48.96	4.81	77.41	37.37	51.72	2.49	2.32	41.98	5.84	5.08	44.60	74.10	47324.76	88292.69
	2010	64.04	55.85	8.19	1002.53	46.13	4.62	77.41	37.73	51.25	2.37	2.25	42.23	5.78	5.06	45.10	74.80	53407.57	88303.16
	2011	64.44	56.22	8.21	990.68	47.75	4.42	77.41	37.45	51.62	2.28	2.14	42.50	5.74	5.04	45.60	75.60	53810.05	88356.56
	2012	64.81	56.57	8.23	1003.99	43.94	4.31	77.41	38.27	50.56	2.18	2.13	42.78	5.69	5.00	46.10	76.30	54185.37	88434.47
	2013	65.17	56.92	8.26	1008.02	46.92	4.51	77.41	38.99	49.62	2.24	2.27	43.08	5.63	4.96	46.60	77.00	-	88521.62
	2014	65.55	57.25	8.29	1018.39	49.53	4.66	77.41	37.28	51.83	2.42	2.25	43.39	5.59	4.90	47.10	77.80	-	88620.14
	2015	65.90	57.57	8.33	1052.96	-	-	-	-	-	-	-	43.72	5.55	4.84	47.60	78.50	-	88719.55
	2016	66.24	57.89	8.35	1092.25	-	-	-	-	-	-	-	44.07	5.55	-	-	-	-	88815.93
	2017	68.05	58.85	9.20	-	-	-	-	-	-	-	-	-	-	-	-	-	-	-
Serbia	1995	70.67	61.92	8.74	3153.38	82.09	6.51	85.60	29.45	65.59	4.27	2.24	51.84	17.21	1.70	96.90	99.40	-	92120.14
	1996	71.87	62.95	8.92	3233.10	149.82	6.51	85.59	29.44	65.60	4.27	2.24	52.11	17.93	-	96.90	99.40	-	92182.56

	1997	72.02	63.08	8.95	3475.10	166.93	6.51	85.64	29.52	65.53	4.27	2.24	52.38	18.64	1.60	96.90	99.40	-	92263.86
	1998	71.41	62.57	8.83	3573.09	112.52	6.57	85.98	30.10	64.99	4.27	2.30	52.65	19.31	-	96.90	99.40	-	92366.76
	1999	70.91	62.16	8.75	3150.46	64.96	6.61	86.22	30.52	64.60	4.27	2.34	52.92	19.94	-	96.90	99.40	-	92470.55
	2000	72.13	63.18	8.96	3405.78	65.52	6.53	85.29	29.51	65.40	4.27	2.26	53.19	20.52	1.48	96.90	99.40	-	92573.86
	2001	72.37	63.37	9.01	3581.97	111.31	6.89	85.58	28.40	66.81	4.60	2.28	53.46	20.97	1.58	96.90	99.40	-	92627.95
	2002	72.48	63.46	9.02	3840.40	174.49	8.09	85.33	23.76	72.16	5.84	2.25	53.72	21.36	1.57	96.80	99.40	-	92663.55
	2003	72.67	63.62	9.05	4018.51	230.83	8.13	86.04	25.00	70.95	5.77	2.36	53.99	21.64	1.59	96.80	99.40	-	92717.21
	2004	72.89	63.79	9.10	4392.28	273.00	8.24	87.70	27.32	68.85	5.67	2.57	54.26	21.81	1.57	96.80	99.40	-	92768.72
	2005	73.21	64.06	9.15	4649.58	304.75	8.70	88.03	29.93	66.00	5.74	2.96	54.53	21.84	1.45	96.70	99.30	-	92824.27
	2006	73.54	64.32	9.23	4896.82	373.16	8.99	88.98	32.92	63.00	5.66	3.33	54.79	21.77	1.43	96.70	99.30	-	92855.16
	2007	73.88	64.53	9.36	5206.25	549.47	10.02	90.16	61.37	61.37	6.15	3.87	55.06	21.58	1.38	96.70	99.30	-	92888.46
	2008	74.22	64.77	9.46	5509.06	672.11	10.05	92.45	35.14	61.98	6.23	3.82	55.11	21.36	1.40	96.60	99.30	-	92936.19
	2009	74.44	64.92	9.52	5358.84	576.43	9.90	92.22	35.17	61.86	6.13	3.78	55.16	21.25	1.44	96.60	99.30	-	92976.90
	2010	74.64	65.11	9.53	5411.88	541.02	10.09	95.55	36.43	61.87	6.24	3.85	55.21	21.32	1.40	96.60	99.30	-	93034.57
	2011	74.84	65.21	9.64	5531.22	627.95	9.72	95.48	36.17	62.12	6.04	3.68	55.26	21.70	1.40	96.50	99.20	-	93012.29
	2012	75.09	65.39	9.69	5501.70	559.65	9.89	95.60	37.13	61.16	6.05	3.84	55.31	22.21	1.45	96.50	99.20	-	93003.49
	2013	75.34	65.58	9.76	5670.71	644.55	10.12	95.95	37.06	59.16	5.98	3.91	55.37	22.85	1.43	96.50	99.20	-	92986.78
	2014	75.53	65.73	9.80	5593.06	632.92	10.37	95.99	36.59	61.88	6.42	3.95	55.46	23.56	1.46	96.50	99.20	-	92984.94
	2015	75.74	65.87	9.86	5663.39	-	-	-	-	-	-	-	55.55	24.33	1.46	96.40	99.20	-	92993.80
	2016	75.90	66.07	9.84	5853.14	-	-	-	-	-	-	-	55.67	25.26	-	-	-	-	92999.13
	2017	75.71	65.28	10.44	-	-	-	-	-	-	-	-	-	-	-	-	-	-	-
Seychelles	1996	70.01	62.03	7.98	8281.28	328.44	5.07	98.73	12.07	87.78	4.45	0.62	49.61	11.56	-	98.40	95.70	396.06	88209.67
	1997	70.22	62.19	8.03	9163.69	399.74	5.57	98.73	13.32	86.51	4.82	0.75	49.72	11.74	2.10	98.40	95.70	447.49	88316.29
	1998	70.44	62.35	8.09	9741.36	379.79	4.95	98.73	15.16	84.64	4.19	0.76	49.84	11.91	2.04	98.40	95.70	-	88447.56
	1999	70.70	62.55	8.16	9730.70	385.74	4.96	98.73	14.36	85.45	4.24	0.72	49.97	12.07	2.04	98.40	95.70	-	88578.64
	2000	70.91	62.71	8.20	9790.27	349.83	4.62	99.17	17.86	81.99	3.79	0.83	50.12	12.22	2.08	98.40	95.70	-	88708.38
	2001	71.11	62.86	8.25	9559.55	335.86	4.45	98.91	15.44	84.39	3.76	0.70	50.28	11.87	1.98	98.40	95.70	-	88825.67
	2002	71.29	63.00	8.29	9384.15	349.10	4.21	99.11	13.48	86.40	3.64	0.57	50.45	11.51	2.04	98.40	95.70	680.72	88929.13
	2003	71.45	63.13	8.32	8932.20	382.15	4.64	98.70	10.60	89.26	4.15	0.50	50.64	11.14	2.10	98.40	95.70	697.09	89002.38

	2004	71.68	63.31	8.37	8709.78	400.98	4.17	97.45	8.42	91.36	3.81	0.36	50.84	10.77	2.00	98.40	95.70	-	89073.74
	2005	71.95	63.55	8.41	9450.28	404.69	3.91	96.62	7.11	92.64	3.62	0.29	51.06	10.39	2.20	98.40	95.70	-	89119.36
	2006	72.31	63.85	8.46	10126.29	421.60	3.73	79.92	5.81	92.73	3.46	0.27	51.28	10.39	2.10	98.40	95.70	-	89108.27
	2007	72.61	64.06	8.55	11124.66	375.67	3.30	78.62	92.70	92.70	3.06	0.24	51.52	10.46	2.24	98.40	95.70	786.52	89111.46
	2008	72.91	64.30	8.61	10645.09	278.32	2.64	71.30	6.26	91.21	2.41	0.23	51.78	10.62	2.30	98.40	95.70	806.42	89129.94
	2009	73.07	64.43	8.64	10486.19	241.85	2.64	67.70	5.72	91.55	2.41	0.22	52.04	10.83	2.40	98.40	95.70	805.94	89200.10
	2010	73.20	64.57	8.64	10804.68	374.52	3.59	38.14	4.09	89.28	3.21	0.39	52.32	11.06	2.10	98.40	95.70	854.07	89301.13
	2011	73.33	64.63	8.70	11967.36	384.30	3.38	88.21	4.79	94.57	3.20	0.18	52.61	11.04	2.40	98.40	95.70	883.83	89263.24
	2012	73.47	64.75	8.73	12633.89	489.97	4.08	35.43	2.48	93.01	3.80	0.29	52.92	11.03	2.40	98.40	95.70	910.29	89244.86
	2013	73.52	64.79	8.74	13153.04	530.31	3.58	29.44	2.00	93.22	3.34	0.24	53.23	11.09	2.40	98.40	95.70	-	89241.13
	2014	73.48	64.75	8.72	13379.59	494.13	3.37	30.01	2.34	92.21	3.11	0.26	53.56	11.31	2.30	98.40	95.70	-	89249.67
	2015	73.56	64.81	8.74	13542.23	-	-	-	-	-	-	-	53.89	11.71	2.30	98.40	95.70	-	89285.66
	2016	73.68	64.95	8.73	13963.59	-	-	-	-	-	-	-	54.21	12.04	-	-	-	-	89344.83
	2017	73.62	64.96	8.66	-	-	-	-	-	-	-	-	-	-	-	-	-	-	-
Sierra Leone	1995	49.84	43.12	6.72	318.28	25.76	11.35	93.02	73.45	21.03	2.39	8.97	34.43	4.95	6.61	10.70	42.00	8960.42	90363.95
	1996	51.20	44.26	6.94	323.29	27.23	11.08	92.83	74.71	19.52	2.16	8.92	34.67	4.94	6.56	10.80	43.00	8920.96	90287.81
	1997	50.70	43.87	6.83	302.66	25.65	11.59	93.62	73.33	21.68	2.51	9.08	34.91	4.88	6.51	11.00	44.00	9166.18	90243.30
	1998	48.96	42.42	6.55	304.65	18.93	10.92	93.46	75.94	18.74	2.05	8.87	35.14	4.78	6.46	11.10	45.10	18981.62	90205.11
	1999	49.04	42.46	6.58	292.96	20.61	12.16	92.49	69.18	25.21	3.06	9.09	35.38	4.63	6.39	11.20	46.10	25037.14	90200.92
	2000	50.89	43.98	6.91	303.79	21.35	13.63	93.13	69.26	25.63	3.49	10.14	35.63	4.45	6.32	11.30	47.20	14763.85	90195.90
	2001	51.29	44.36	6.93	271.69	30.27	11.83	94.87	72.23	23.86	2.82	9.01	35.87	4.47	6.24	11.50	48.20	32393.59	90214.35
	2002	51.35	44.46	6.89	327.97	33.51	11.96	94.86	71.34	24.79	2.96	9.00	36.11	4.47	6.16	11.60	49.20	22463.63	90240.83
	2003	51.70	44.78	6.92	342.02	34.50	11.69	95.10	72.46	23.81	2.78	8.91	36.35	4.46	6.07	11.70	50.20	20892.19	90244.06
	2004	52.09	45.13	6.96	347.90	34.27	11.66	91.28	70.27	23.01	2.68	8.98	36.60	4.44	5.97	11.80	51.30	21206.33	90241.50
	2005	52.44	45.42	7.02	348.94	39.31	12.25	84.28	65.86	21.85	2.68	9.57	36.84	4.42	5.86	11.90	52.30	14869.18	90227.12
	2006	52.87	45.86	7.01	356.24	38.39	10.68	88.55	72.29	18.37	1.96	8.72	37.10	4.48	5.75	12.10	53.30	26983.21	90153.17
	2007	53.21	46.21	6.99	374.28	40.51	10.12	94.30	16.26	16.26	1.65	8.47	37.36	4.52	5.62	12.20	54.30	28565.22	90071.97
	2008	53.82	46.79	7.03	384.90	46.70	10.29	96.48	79.72	17.37	1.79	8.50	37.65	4.54	5.49	12.30	55.40	33083.70	90014.09
	2009	54.31	47.24	7.07	393.55	57.07	13.13	76.70	63.81	16.81	2.21	10.93	37.94	4.54	5.35	12.50	56.40	17509.10	89966.77

	2010	55.04	47.89	7.15	405.13	46.06	10.32	85.81	65.88	23.23	2.40	7.92	38.24	4.53	5.20	12.60	57.40	11647.49	89957.37
	2011	55.70	48.52	7.19	414.79	59.47	11.98	83.62	65.15	22.09	2.65	9.34	38.56	4.58	5.06	12.70	58.50	11731.17	89939.14
	2012	56.44	49.18	7.26	466.86	70.45	11.24	78.33	64.52	17.63	1.98	9.25	38.89	4.61	4.92	12.80	59.50	11810.89	89933.54
	2013	57.38	50.02	7.36	550.88	92.40	11.59	73.45	62.70	14.63	1.69	9.89	39.23	4.62	4.79	13.00	60.60	-	89945.20
	2014	53.87	47.17	6.70	563.17	85.91	11.09	73.43	60.96	16.99	1.88	9.21	39.58	4.60	4.67	13.10	61.60	-	89946.94
	2015	57.62	50.31	7.30	438.00	-	-	-	-	-	-	-	39.94	4.57	4.56	13.30	62.60	-	89966.48
	2016	59.15	51.63	7.52	455.59	-	-	-	-	-	-	-	40.32	4.59	-	-	-	-	89987.36
	2017	60.42	52.09	8.33	-	-	-	-	-	-	-	-	-	-	-	-	-	-	-
Singapore	1995	77.36	68.39	8.98	29008.50	740.80	2.94	96.91	48.83	49.61	1.46	1.48	100.00	8.89	1.67	99.40	100.00	-	86377.64
	1996	77.70	68.66	9.04	29951.02	771.75	2.86	96.54	49.52	48.70	1.39	1.47	100.00	9.14	1.66	99.50	100.00	-	86247.01
	1997	78.17	69.05	9.12	31363.41	766.39	2.80	96.34	49.99	48.11	1.34	1.45	100.00	9.40	1.61	99.50	100.00	-	86182.56
	1998	78.53	69.35	9.18	29641.23	712.30	3.10	95.60	44.35	53.60	1.66	1.44	100.00	9.68	1.48	99.60	100.00	-	86180.31
	1999	78.93	69.69	9.24	31197.61	635.50	2.82	95.35	45.28	52.51	1.48	1.34	100.00	9.98	1.47	99.60	100.00	-	86158.72
	2000	79.38	70.08	9.30	33390.06	661.80	2.71	95.73	52.64	45.01	1.22	1.49	100.00	10.32	1.60	99.70	100.00	-	86184.85
	2001	79.82	70.43	9.39	32191.94	525.71	2.37	94.90	59.36	37.45	0.89	1.48	100.00	10.52	1.41	99.80	100.00	49851.52	86339.81
	2002	80.15	70.72	9.43	33242.99	632.06	2.84	95.64	60.02	37.24	1.06	1.78	100.00	10.71	1.37	99.80	100.00	-	86528.56
	2003	80.37	70.94	9.43	35233.13	826.63	3.63	93.33	58.68	37.13	1.35	2.28	100.00	10.87	1.27	99.90	100.00	43165.65	86742.74
	2004	80.67	71.22	9.46	38117.41	828.28	3.17	92.94	62.77	32.46	1.03	2.14	100.00	11.05	1.26	99.90	100.00	44836.63	87005.56
	2005	80.97	71.51	9.46	40020.26	1061.10	3.74	94.72	69.32	26.81	1.00	2.74	100.00	11.33	1.26	100.00	100.00	47597.81	87179.63
	2006	81.16	71.68	9.48	42223.89	1170.89	3.66	94.89	68.90	27.38	1.00	2.65	100.00	11.64	1.28	100.00	100.00	48418.80	87305.97
	2007	81.64	72.00	9.64	44191.24	1315.15	3.46	94.35	27.63	27.63	0.96	2.50	100.00	11.89	1.29	100.00	100.00	49828.82	87364.61
	2008	82.11	72.36	9.75	42650.10	1550.51	3.91	94.50	65.49	30.69	1.20	2.71	100.00	12.04	1.28	100.00	100.00	50059.95	87491.69
	2009	82.44	72.61	9.83	41133.30	1655.89	4.27	94.13	60.50	35.73	1.53	2.75	100.00	12.13	1.22	100.00	100.00	49828.22	87620.85
	2010	82.76	72.90	9.86	46569.68	1841.59	3.96	93.56	61.13	34.66	1.37	2.58	100.00	12.25	1.15	100.00	100.00	52732.00	87752.20
	2011	82.97	73.02	9.95	48447.68	2086.14	3.93	93.25	61.32	34.24	1.35	2.59	100.00	12.79	1.20	100.00	100.00	54413.55	87978.10
	2012	83.22	73.20	10.02	49103.71	2310.36	4.22	93.25	60.10	35.55	1.50	2.72	100.00	13.47	1.29	100.00	100.00	55910.28	88115.32
	2013	83.30	73.26	10.04	50731.30	2531.50	4.53	93.95	57.93	38.35	1.74	2.79	100.00	14.26	1.19	100.00	100.00	-	88282.24
	2014	83.45	73.37	10.08	51865.72	2752.32	4.92	94.11	54.83	41.74	2.05	2.87	100.00	15.13	1.25	100.00	100.00	-	88458.68
	2015	83.65	73.52	10.13	52244.59	-	-	-	-	-	-	-	100.00	16.05	1.24	100.00	100.00	-	88627.89

	2016	83.73	73.62	10.11	52600.64	-	-	-	-	-	-	-	100.00	16.96	-	-	-	-	88839.75
	2017	84.79	74.22	10.57	-	-	-	-	-	-	-	-	-	-	-	-	-	-	-
Slovak	1995	72.36	63.19	9.17	8698.87	221.76	6.06	100.00	11.48	88.52	5.37	0.70	56.54	16.13	1.52	98.90	99.80	53824.71	91114.74
	1996	72.71	63.47	9.25	9267.03	253.75	6.44	100.00	11.32	88.68	5.71	0.73	56.48	16.25	1.47	98.90	99.80	54863.26	91211.38
	1997	72.74	63.51	9.24	9811.18	230.05	5.78	100.00	8.32	91.68	5.30	0.48	56.42	16.34	1.43	98.90	99.80	54885.14	91294.93
	1998	72.76	63.53	9.23	10191.11	234.92	5.66	100.00	8.41	91.59	5.18	0.48	56.36	16.41	1.37	98.90	99.80	53026.07	91381.84
	1999	73.00	63.72	9.28	10159.83	218.43	5.75	100.00	10.36	89.64	5.16	0.60	56.29	16.42	1.33	98.90	99.80	52301.81	91456.72
	2000	73.25	63.94	9.31	10296.71	207.84	5.50	100.00	10.61	89.39	4.92	0.58	56.23	16.37	1.30	98.90	99.80	50421.40	91568.00
	2001	73.56	64.19	9.37	10657.69	216.06	5.50	100.00	10.71	89.29	4.91	0.59	56.16	16.39	1.20	98.90	99.80	50059.68	91644.08
	2002	73.79	64.37	9.42	11143.76	256.06	5.63	100.00	10.94	89.06	5.01	0.62	56.01	16.37	1.19	98.90	99.90	49587.28	91716.10
	2003	73.92	64.48	9.44	11755.35	360.23	5.82	100.00	11.68	88.32	5.14	0.68	55.86	16.32	1.20	98.90	99.90	49336.11	91771.74
	2004	74.16	64.68	9.48	12376.06	566.27	7.21	73.15	19.18	73.77	5.32	1.89	55.71	16.28	1.25	98.90	99.90	48357.39	91824.91
	2005	74.23	64.77	9.46	13210.27	628.10	7.04	88.15	22.57	74.40	5.24	1.80	55.56	16.27	1.27	98.90	99.90	48958.79	91879.02
	2006	74.42	64.93	9.50	14326.26	763.16	7.35	81.92	25.95	68.32	5.02	2.33	55.41	16.40	1.25	98.80	99.90	48387.52	91874.15
	2007	74.56	64.98	9.58	15868.80	1082.05	7.76	79.10	66.85	66.85	5.18	2.57	55.26	16.56	1.27	98.80	99.90	48141.60	91874.50
	2008	74.89	65.21	9.68	16747.81	1406.45	8.02	78.29	25.24	67.76	5.44	2.59	55.11	16.75	1.34	98.80	100.00	49160.99	91867.02
	2009	75.15	65.40	9.75	15818.56	1483.39	9.15	74.48	25.55	65.69	6.01	3.14	54.96	16.97	1.44	98.80	100.00	46722.86	91833.81
	2010	75.56	65.76	9.80	16600.61	1377.73	8.51	67.98	21.66	68.13	5.80	2.71	54.69	17.24	1.43	98.80	100.00	49973.28	91814.73
	2011	75.91	65.98	9.94	17046.61	1414.97	7.96	77.91	22.65	70.93	5.64	2.31	54.41	17.66	1.45	98.80	100.00	48551.75	91789.18
	2012	76.23	66.22	10.00	17299.63	1376.86	8.15	73.88	22.37	69.72	5.68	2.47	54.16	18.11	1.34	98.80	100.00	46301.27	91803.42
	2013	76.53	66.46	10.07	17538.65	1417.02	8.00	81.96	22.69	72.31	5.79	2.22	53.95	18.61	1.34	98.80	100.00	-	91822.84
	2014	76.80	66.67	10.14	18003.54	1454.81	8.05	81.96	22.54	72.51	5.84	2.21	53.76	19.20	1.37	98.80	100.00	-	91848.38
	2015	76.90	66.75	10.15	18678.93	-	-	-	-	-	-	-	53.60	19.89	1.37	98.80	100.00	-	91901.76
	2016	76.97	66.88	10.09	19282.52	-	-	-	-	-	-	-	53.47	20.74	-	-	-	-	91978.56
	2017	77.41	66.33	11.08	-	-	-	-	-	-	-	-	-	-	-	-	-	-	-
Slovenia	1995	74.57	64.62	9.95	15062.82	784.95	7.46	50.30	11.22	77.70	5.79	1.66	50.62	17.69	1.29	99.10	99.60	18869.70	91385.95
	1996	74.88	64.86	10.02	15602.22	811.82	7.64	49.50	11.79	76.18	5.82	1.82	50.64	18.21	1.28	99.10	99.60	20088.20	91498.63
	1997	75.09	65.03	10.06	16422.25	797.51	7.76	46.75	11.67	75.03	5.82	1.94	50.67	18.74	1.25	99.10	99.60	21798.46	91623.42
	1998	75.32	65.20	10.11	16998.86	856.07	7.81	48.39	11.85	75.52	5.90	1.91	50.70	19.25	1.23	99.10	99.60	21456.26	91751.40

	1999	75.61	65.43	10.18	17882.66	879.65	7.80	50.83	12.37	75.66	5.90	1.90	50.73	19.71	1.21	99.10	99.60	21132.26	91869.48
	2000	76.03	65.75	10.27	18570.70	831.07	8.26	44.10	11.46	74.01	6.12	2.15	50.75	20.12	1.26	99.10	99.60	20927.09	91999.48
	2001	76.26	65.88	10.38	19088.33	882.70	8.57	39.18	10.46	73.29	6.28	2.29	50.78	20.55	1.21	99.10	99.60	21694.41	92042.08
	2002	76.60	66.11	10.48	19796.07	1000.17	8.62	43.20	11.50	73.37	6.32	2.30	50.78	20.93	1.21	99.10	99.60	22342.26	92102.01
	2003	76.78	66.23	10.55	20346.42	1281.13	8.77	41.85	11.71	72.02	6.31	2.45	50.69	21.28	1.20	99.10	99.60	21936.49	92154.90
	2004	77.18	66.49	10.69	21218.25	1435.09	8.47	43.76	11.60	73.50	6.22	2.24	50.59	21.62	1.25	99.10	99.60	22064.48	92201.29
	2005	77.57	66.79	10.79	22029.43	1519.55	8.50	46.22	12.42	73.13	6.21	2.28	50.50	21.98	1.26	99.10	99.60	22366.88	92267.19
	2006	77.93	67.05	10.89	23201.26	1636.73	8.42	42.40	11.56	72.73	6.12	2.30	50.41	22.39	1.31	99.10	99.60	22781.65	92246.08
	2007	78.29	67.26	11.02	24673.44	1880.51	7.98	46.99	72.33	72.33	5.77	2.21	50.32	22.82	1.38	99.10	99.60	22932.54	92191.96
	2008	78.78	67.63	11.15	25447.43	2297.77	8.47	46.50	11.94	74.32	6.29	2.17	50.22	23.25	1.53	99.10	99.60	23252.26	92138.96
	2009	79.13	67.88	11.25	23252.10	2271.08	9.38	45.53	11.91	73.83	6.92	2.45	50.13	23.67	1.53	99.10	99.60	21453.16	92098.16
	2010	79.50	68.19	11.31	23437.47	2083.06	9.07	47.41	12.23	74.20	6.73	2.34	50.04	24.07	1.57	99.10	99.50	21893.66	92090.17
	2011	79.86	68.44	11.42	23540.71	2225.15	9.08	44.41	11.76	73.52	6.67	2.40	49.95	24.53	1.56	99.10	99.50	21513.48	92053.26
	2012	80.05	68.61	11.44	22864.21	2068.52	9.37	43.48	11.90	72.63	6.80	2.56	49.86	24.96	1.58	99.10	99.50	21074.75	92061.51
	2013	80.30	68.81	11.49	22574.71	2114.88	9.29	42.86	12.11	71.76	6.67	2.62	49.76	25.43	1.55	99.10	99.50	-	92083.19
	2014	80.79	69.19	11.60	23224.40	2160.75	9.23	42.69	12.07	71.73	6.62	2.61	49.70	26.03	1.58	99.10	99.50	-	92101.57
	2015	80.73	69.17	11.56	23731.17	-	-	-	-	-	-	-	49.65	26.82	1.58	99.10	99.50	-	92131.90
	2016	80.84	69.32	11.51	24462.73	-	-	-	-	-	-	-	49.63	27.77	-	-	-	-	92177.32
	2017	81.10	68.74	12.36	-	-	-	-	-	-	-	-	-	-	-	-	-	-	-
Solomon Islands	1995	60.94	53.66	7.29	1630.24	46.94	3.25	55.88	4.02	92.81	3.01	0.23	14.66	4.95	5.20	-	-	3885.28	86227.23
	1996	61.13	53.81	7.32	1610.48	46.19	3.02	55.88	4.35	92.21	2.78	0.24	14.87	4.98	5.08	-	-	4053.19	86198.99
	1997	61.27	53.92	7.35	1543.52	49.09	3.28	55.88	4.02	92.80	3.05	0.24	15.08	5.03	4.97	-	-	4059.36	86181.15
	1998	61.38	54.01	7.37	1528.04	42.57	3.53	55.88	3.98	92.88	3.28	0.25	15.29	5.08	4.87	-	-	3887.43	86170.80
	1999	61.45	54.06	7.39	1479.40	45.72	3.80	55.88	3.82	93.17	3.54	0.26	15.50	5.11	4.79	-	-	3784.05	86171.83
	2000	61.40	54.02	7.39	1234.30	48.12	4.56	56.70	3.24	94.28	4.30	0.26	15.81	5.11	4.72	25.50	79.70	4086.06	86193.46
	2001	61.30	53.91	7.38	1105.69	60.92	6.44	56.70	2.47	95.64	6.16	0.28	16.20	5.18	4.67	25.70	79.80	4147.14	86266.17
	2002	61.16	53.79	7.37	1046.56	47.85	6.09	56.70	2.51	95.58	5.82	0.27	16.60	5.23	4.62	26.00	79.80	4329.77	86344.26
	2003	61.06	53.70	7.36	1085.88	44.76	6.00	56.70	4.40	92.24	5.54	0.47	17.00	5.26	4.58	26.20	79.90	4401.76	86452.82
	2004	61.00	53.63	7.37	1110.35	46.21	5.64	56.70	4.62	91.85	5.18	0.46	17.41	5.31	4.54	26.50	80.00	4422.74	86551.81

	2005	61.00	53.61	7.39	1141.74	69.10	7.83	56.70	3.36	94.08	7.37	0.46	17.83	5.37	4.50	26.80	80.00	4323.80	86678.19
	2006	61.07	53.70	7.37	1191.80	63.31	6.66	56.70	3.90	93.12	6.21	0.46	18.26	5.48	4.46	27.10	80.10	4336.37	86751.94
	2007	60.97	53.62	7.35	1249.15	67.35	6.42	56.70	92.72	92.72	5.95	0.47	18.69	5.60	4.41	27.40	80.20	4406.11	86822.51
	2008	61.36	53.95	7.41	1307.18	72.15	5.97	56.70	4.26	92.49	5.52	0.45	19.14	5.72	4.36	27.70	80.30	4411.73	86903.67
	2009	61.40	53.98	7.42	1217.38	91.99	7.92	56.70	3.35	94.09	7.45	0.47	19.59	5.83	4.30	28.00	80.30	4419.52	86993.75
	2010	61.68	54.21	7.47	1272.45	95.32	7.47	56.70	3.38	94.04	7.02	0.44	20.05	5.92	4.24	28.30	80.40	4479.83	87087.08
	2011	61.92	54.41	7.52	1405.47	83.81	5.08	56.70	4.32	92.39	4.70	0.39	20.51	5.97	4.17	28.60	80.50	4536.58	87187.18
	2012	62.16	54.60	7.56	1439.19	102.30	5.48	56.70	3.99	92.96	5.09	0.39	20.96	6.01	4.10	28.90	80.50	4591.46	87309.90
	2013	62.35	54.75	7.60	1451.05	102.47	5.42	56.70	4.51	92.05	4.99	0.43	21.42	6.03	4.03	29.20	80.60	-	87448.11
	2014	62.39	54.77	7.62	1442.23	102.24	5.05	56.70	4.61	91.87	4.64	0.41	21.88	6.04	3.97	29.50	80.70	-	87609.49
	2015	62.77	55.08	7.69	1465.59	-	-	-	-	-	-	-	22.33	6.04	3.91	29.80	80.80	-	87776.83
	2016	63.04	55.30	7.74	1479.34	-	-	-	-	-	-	-	22.78	6.07	-	-	-	-	87943.20
	2017	65.73	57.15	8.58	-	-	-	-	-	-	-	-	-	-	-	-	-	-	-
Somalia	1995	51.34	45.22	6.12	-	-	-	-	-	-	-	-	31.43	5.37	7.65	21.40	21.00	18762.63	89444.04
	1996	51.21	45.12	6.09	-	-	-	-	-	-	-	-	31.79	5.37	7.68	21.50	21.00	19206.20	89487.16
	1997	50.89	44.85	6.04	-	-	-	-	-	-	-	-	32.15	5.35	7.69	21.60	21.10	19652.85	89554.37
	1998	51.60	45.45	6.15	-	-	-	-	-	-	-	-	32.51	5.32	7.68	21.70	21.80	20919.89	89621.11
	1999	51.75	45.56	6.19	-	-	-	-	-	-	-	-	32.88	5.29	7.66	21.80	22.60	21185.95	89681.73
	2000	51.99	45.75	6.24	-	-	-	-	-	-	-	-	33.25	5.27	7.62	21.80	23.50	20491.62	89741.19
	2001	52.39	46.13	6.26	-	-	-	-	-	-	-	-	33.62	5.30	7.57	21.90	24.40	20649.51	89714.82
	2002	52.66	46.39	6.27	-	-	-	-	-	-	-	-	33.99	5.33	7.51	22.00	25.30	20911.00	89702.51
	2003	53.07	46.77	6.30	-	-	-	-	-	-	-	-	34.37	5.35	7.45	22.10	26.30	21516.58	89680.99
	2004	53.32	47.02	6.30	-	-	-	-	-	-	-	-	34.76	5.36	7.38	22.30	27.30	21192.85	89673.91
	2005	53.83	47.45	6.38	-	-	-	-	-	-	-	-	35.16	5.35	7.31	22.40	28.40	21219.93	89672.85
	2006	53.98	47.59	6.39	-	-	-	-	-	-	-	-	35.56	5.37	7.23	22.60	29.50	21176.81	89651.23
	2007	54.08	47.66	6.42	-	-	-	-	-	-	-	-	35.97	5.37	7.14	22.70	30.70	21272.60	89630.02
	2008	54.70	48.19	6.51	-	-	-	-	-	-	-	-	36.39	5.35	7.06	22.90	30.90	21450.95	89625.91
	2009	54.96	48.39	6.57	-	-	-	-	-	-	-	-	36.82	5.33	6.96	23.10	31.20	21130.62	89634.16
	2010	53.08	46.74	6.34	-	-	-	-	-	-	-	-	37.26	5.30	6.87	23.30	31.40	21461.81	89673.13

	2011	53.35	47.00	6.35	-	-	-	-	-	-	-	-	37.70	5.32	6.77	23.50	31.70	21690.27	89600.76
	2012	55.79	49.15	6.64	-	-	-	-	-	-	-	-	38.15	5.33	6.67	-	-	21916.32	89536.70
	2013	56.55	49.82	6.73	-	-	-	-	-	-	-	-	38.61	5.33	6.56	-	-	-	89476.81
	2014	56.88	50.13	6.75	-	-	-	-	-	-	-	-	39.08	5.32	6.46	-	-	-	89418.62
	2015	57.19	50.41	6.78	-	-	-	-	-	-	-	-	39.55	5.31	6.37	-	-	-	89361.67
	2016	57.13	50.39	6.75	-	-	-	-	-	-	-	-	40.03	5.34	-	-	-	-	89307.63
	2017	58.52	51.20	7.32	-	-	-	-	-	-	-	-	-	-	-	-	-	-	-
South Africa	1995	64.15	55.55	8.61	5616.42	310.86	8.28	29.88	17.52	41.38	3.43	4.86	54.49	6.22	3.11	53.80	84.00	371350.21	88140.20
	1996	62.82	54.48	8.34	5749.58	296.25	8.47	29.23	15.98	45.33	3.84	4.63	54.97	6.30	3.04	54.50	84.50	383673.27	88236.81
	1997	60.88	52.92	7.97	5803.96	305.34	8.59	26.54	14.94	43.72	3.75	4.83	55.45	6.35	2.97	55.10	85.00	403030.55	88343.02
	1998	59.34	51.61	7.73	5745.30	268.23	8.49	25.38	14.66	42.24	3.58	4.90	55.93	6.40	2.92	55.80	85.50	457895.90	88444.47
	1999	57.55	50.07	7.48	5794.41	262.73	8.51	23.42	14.01	40.19	3.42	5.09	56.41	6.43	2.87	56.50	86.00	430853.84	88542.36
	2000	55.40	48.13	7.27	5946.00	245.06	8.07	23.10	13.68	40.79	3.29	4.78	56.89	6.45	2.83	57.20	86.50	420482.75	88641.33
	2001	54.32	47.25	7.07	6052.56	221.67	8.31	21.03	12.61	40.06	3.33	4.98	57.37	6.54	2.79	57.80	87.00	416031.24	88695.31
	2002	53.14	46.27	6.87	6200.32	201.80	8.09	19.49	11.70	39.97	3.23	4.85	57.90	6.61	2.76	58.50	87.40	434071.08	88737.39
	2003	52.14	45.44	6.71	6305.79	308.09	8.26	18.54	11.04	40.42	3.34	4.92	58.45	6.67	2.73	59.20	87.90	456334.13	88779.40
	2004	51.48	44.86	6.62	6511.14	380.13	7.93	18.26	10.84	40.60	3.22	4.71	58.99	6.73	2.70	59.80	88.40	472713.79	88819.93
	2005	51.13	44.47	6.65	6767.63	414.15	7.77	18.26	10.47	42.68	3.32	4.45	59.54	6.78	2.68	60.40	88.90	498312.89	88874.72
	2006	51.05	44.46	6.59	7053.94	419.29	7.57	18.08	10.05	44.41	3.36	4.21	60.08	6.89	2.66	61.10	89.40	480054.73	88887.95
	2007	51.50	44.86	6.65	7333.24	453.89	7.53	16.85	45.11	45.11	3.40	4.13	60.62	6.99	2.64	61.70	89.80	502130.26	88905.41
	2008	52.21	45.45	6.76	7464.37	441.24	7.75	15.69	8.41	46.41	3.60	4.15	61.15	7.09	2.62	62.30	90.30	-	88929.73
	2009	53.14	46.21	6.93	7247.45	486.71	8.39	14.55	7.70	47.07	3.95	4.44	61.69	7.16	2.60	62.90	90.70	-	88967.00
	2010	54.35	47.15	7.19	7361.76	618.35	8.50	13.84	7.35	46.92	3.99	4.51	62.22	7.22	2.59	63.50	91.10	-	89012.30
	2011	55.83	48.43	7.40	7493.30	686.94	8.61	13.43	6.99	47.99	4.13	4.48	62.75	7.32	2.57	64.10	91.60	-	89051.31
	2012	57.57	49.88	7.68	7545.78	661.43	8.79	13.09	6.72	48.62	4.28	4.52	63.27	7.41	2.55	64.70	92.00	-	89096.61
	2013	59.31	51.31	7.99	7616.78	601.37	8.78	12.66	6.59	47.92	4.20	4.57	63.79	7.50	2.53	65.30	92.40	-	89134.24
	2014	60.69	52.44	8.25	7626.81	570.21	8.80	12.54	6.49	48.24	4.24	4.55	64.30	7.61	2.51	65.80	92.80	-	89163.14
	2015	61.83	53.35	8.48	7604.36	-	-	-	-	-	-	-	64.80	7.75	2.49	66.40	93.20	-	89181.79
	2016	62.44	53.83	8.61	7503.27	-	-	-	-	-	-	-	65.30	7.94	-	-	-	-	89183.53

	2017	66.31	57.05	9.26	-	-	-	-	-	-	-	-	-	-	-	-	-	-	-
South Sudan	1995	53.34	45.08	8.26	-	-	-	-	-	-	-	-	15.87	5.45	6.55	-	-	-	88452.12
	1996	53.11	44.97	8.14	-	-	-	-	-	-	-	-	16.00	5.52	6.50	-	-	-	88488.84
	1997	53.08	45.03	8.05	-	-	-	-	-	-	-	-	16.12	5.60	6.44	-	-	-	88528.84
	1998	53.30	45.24	8.06	-	-	-	-	-	-	-	-	16.25	5.67	6.38	-	-	-	88565.19
	1999	54.57	46.37	8.20	-	-	-	-	-	-	-	-	16.38	5.74	6.30	-	-	-	88581.75
	2000	54.50	46.36	8.14	-	-	-	-	-	-	-	-	16.50	5.80	6.22	-	-	-	88589.29
	2001	54.62	46.56	8.06	-	-	-	-	-	-	-	-	16.63	5.87	6.14	-	-	-	88566.21
	2002	54.97	46.90	8.07	-	-	-	-	-	-	-	-	16.76	5.93	6.05	-	-	-	88554.73
	2003	56.01	47.81	8.20	-	-	-	-	-	-	-	-	16.89	5.98	5.97	-	-	-	88553.51
	2004	56.02	47.06	8.96	-	-	-	-	-	-	-	-	17.02	6.03	5.89	-	-	-	88557.36
	2005	56.59	47.56	9.03	-	-	-	-	-	-	-	-	17.15	6.08	5.80	-	-	-	88573.25
	2006	56.76	48.63	8.12	-	-	-	-	-	-	-	-	17.29	6.13	5.72	-	-	-	88543.64
	2007	57.12	49.02	8.10	-	-	-	-	-	-	-	-	17.42	6.17	5.64	-	-	-	88518.72
	2008	57.52	49.43	8.09	1532.18	-	-	-	-	-	-	-	17.56	6.21	5.55	-	-	-	88495.84
	2009	57.47	49.47	8.01	1541.61	-	-	-	-	-	-	-	17.70	6.26	5.47	-	-	-	88478.60
	2010	58.12	50.07	8.05	1562.24	-	-	-	-	-	-	-	17.86	6.31	5.38	-	-	-	88467.63
	2011	58.07	50.08	7.98	1435.33	-	-	-	-	-	-	-	18.02	6.35	5.29	6.60	58.60	-	88369.76
	2012	58.40	50.41	7.98	747.47	26.19	2.77	92.80	62.12	33.06	0.92	1.86	18.20	6.38	5.20	6.60	58.70	-	88275.42
	2013	58.56	50.60	7.96	818.44	26.99	2.62	93.01	60.28	35.19	0.92	1.70	18.39	6.40	5.11	6.70	58.70	-	88205.49
	2014	58.73	50.78	7.94	820.11	30.05	2.74	92.59	54.15	41.52	1.14	1.60	18.59	6.40	5.02	6.70	58.70	-	88118.11
	2015	58.88	50.95	7.93	745.34	-	-	-	-	-	-	-	18.80	6.37	4.94	6.70	58.70	-	88044.89
	2016	59.70	51.67	8.03	-	-	-	-	-	-	-	-	19.03	6.33	-	-	-	-	87957.30
	2017	59.25	50.60	8.64	-	-	-	-	-	-	-	-	-	-	-	-	-	-	-
Spain	1995	78.15	68.51	9.63	23686.84	1129.19	7.44	84.61	23.54	72.18	5.37	2.07	75.86	22.19	1.16	99.90	99.90	319459.33	91483.16
	1996	78.36	68.72	9.63	24219.33	1178.14	7.46	83.90	23.19	72.36	5.40	2.06	75.94	22.63	1.14	99.90	99.90	309786.21	91562.88
	1997	78.70	69.04	9.66	25007.89	1062.57	7.33	83.89	23.10	72.47	5.31	2.02	76.02	23.10	1.15	99.90	99.90	329912.39	91648.64
	1998	78.92	69.26	9.66	25977.00	1109.46	7.32	83.61	23.25	72.20	5.28	2.03	76.10	23.58	1.13	99.90	99.90	343785.34	91714.17
	1999	79.11	69.45	9.66	27032.22	1135.74	7.32	83.31	23.30	72.03	5.27	2.05	76.18	24.02	1.17	99.90	99.90	363569.60	91747.15

	2000	79.40	69.68	9.72	28335.00	1045.48	7.21	83.08	23.58	71.62	5.17	2.05	76.26	24.37	1.22	99.90	99.90	381031.15	91783.75
	2001	79.67	69.90	9.77	29264.88	1089.30	7.24	83.09	23.93	71.20	5.16	2.09	76.34	24.39	1.23	99.90	99.90	377629.97	91812.96
	2002	79.83	70.03	9.79	29685.36	1212.66	7.25	82.61	23.72	71.29	5.17	2.08	76.53	24.34	1.25	99.90	99.90	397417.36	91834.20
	2003	79.98	70.16	9.82	30082.63	1687.90	7.99	77.17	21.84	71.70	5.73	2.26	76.78	24.26	1.30	99.90	100.00	407869.66	91834.31
	2004	80.25	70.38	9.87	30504.36	1975.38	8.05	77.50	21.65	72.06	5.80	2.25	77.02	24.21	1.31	99.90	100.00	421107.43	91830.86
	2005	80.48	70.55	9.93	31110.01	2120.50	8.12	76.91	21.25	72.37	5.88	2.24	77.26	24.22	1.33	99.90	100.00	437016.74	91827.80
	2006	80.83	70.83	10.00	31865.37	2312.79	8.23	75.74	20.49	72.95	6.01	2.23	77.50	24.34	1.36	99.90	100.00	430218.33	91819.66
	2007	81.07	71.02	10.05	32459.92	2690.00	8.36	74.86	72.97	72.97	6.10	2.26	77.74	24.49	1.38	99.90	100.00	439904.17	91802.15
	2008	81.36	71.25	10.11	32303.24	3072.17	8.80	77.98	20.30	73.97	6.51	2.29	77.98	24.69	1.45	99.90	100.00	404869.43	91794.29
	2009	81.68	71.49	10.19	30874.13	2994.73	9.52	78.13	18.97	75.72	7.21	2.31	78.21	24.93	1.38	99.90	100.00	372822.52	91814.41
	2010	81.97	71.71	10.26	30736.63	2846.71	9.56	81.17	20.24	75.07	7.17	2.38	78.44	25.23	1.37	99.90	100.00	354618.02	91871.95
	2011	82.18	71.90	10.28	30321.70	2954.25	9.48	79.23	20.71	73.86	7.00	2.48	78.67	25.82	1.34	99.90	100.00	353857.39	91918.93
	2012	82.32	72.04	10.28	29414.86	2651.38	9.39	79.74	22.54	71.73	6.73	2.65	78.90	26.45	1.32	99.90	100.00	348257.29	92000.48
	2013	82.63	72.30	10.33	29008.02	2643.95	9.10	82.34	23.47	71.49	6.50	2.59	79.13	27.13	1.27	99.90	100.00	-	92105.54
	2014	82.84	72.49	10.36	29496.38	2658.27	9.03	82.39	23.99	70.88	6.40	2.63	79.36	27.82	1.32	99.90	100.00	-	92202.08
	2015	82.91	72.55	10.35	30530.57	-	-	-	-	-	-	-	79.58	28.51	1.32	99.90	100.00	-	92293.57
	2016	82.97	72.62	10.35	31532.82	-	-	-	-	-	-	-	79.80	29.02	-	-	-	-	92381.24
	2017	83.06	72.08	10.98	-	-	-	-	-	-	-	-	-	-	-	-	-	-	-
Sri Lanka	1995	71.99	63.07	8.92	1483.16	24.56	3.44	85.86	45.54	46.96	1.62	1.82	18.50	9.38	2.29	76.00	74.00	20611.10	88863.29
	1996	71.88	63.00	8.88	1528.03	26.17	3.46	87.61	46.81	46.57	1.61	1.85	18.49	9.40	2.27	77.00	75.10	21681.63	88923.60
	1997	71.89	63.03	8.86	1615.87	27.31	3.34	85.89	46.84	45.47	1.52	1.82	18.48	9.39	2.25	78.00	76.30	21386.16	88998.37
	1998	71.98	63.13	8.85	1682.43	31.58	3.71	85.89	42.45	50.58	1.88	1.83	18.47	9.35	2.24	79.10	77.40	20556.13	89082.01
	1999	72.43	63.52	8.91	1744.65	30.65	3.65	84.51	44.11	47.80	1.75	1.91	18.46	9.31	2.24	80.10	78.60	22816.59	89138.63
	2000	72.51	63.60	8.92	1837.14	32.82	3.77	80.83	41.30	48.91	1.85	1.93	18.44	9.28	2.24	81.20	79.70	23282.57	89186.39
	2001	73.06	64.07	8.99	1795.08	31.69	3.81	82.31	44.30	46.18	1.76	2.05	18.43	9.44	2.25	82.20	80.80	22429.20	89207.65
	2002	73.32	64.30	9.02	1852.26	34.88	3.89	81.08	45.97	43.30	1.68	2.20	18.42	9.61	2.26	83.30	82.00	23464.89	89214.30
	2003	73.92	64.81	9.12	1947.48	38.85	3.95	81.40	46.86	42.43	1.68	2.28	18.41	9.77	2.27	84.30	83.10	24580.38	89211.71
	2004	69.33	60.96	8.37	2038.04	45.67	4.28	82.71	43.98	46.83	2.01	2.28	18.39	9.94	2.28	85.40	84.20	25393.93	89226.76
	2005	73.93	64.81	9.11	2149.04	50.76	4.06	82.53	44.93	45.56	1.85	2.21	18.38	10.13	2.28	86.40	85.40	27223.29	89264.88

	2006	74.25	65.11	9.15	2296.41	58.40	4.06	82.23	42.76	48.01	1.95	2.11	18.37	10.24	2.28	87.50	86.50	26000.04	89247.76
	2007	74.63	65.39	9.24	2434.04	61.37	3.76	81.09	48.58	48.58	1.83	1.93	18.36	10.35	2.27	88.50	87.60	27768.84	89230.25
	2008	73.95	64.81	9.14	2559.47	70.30	3.44	81.08	43.86	45.91	1.58	1.86	18.35	10.47	2.26	89.60	88.80	27955.00	89238.10
	2009	73.86	64.71	9.15	2630.01	70.58	3.37	80.81	43.98	45.57	1.54	1.83	18.33	10.64	2.23	90.60	89.90	27826.56	89257.78
	2010	74.38	65.16	9.22	2819.51	84.22	3.43	81.90	44.83	45.27	1.55	1.88	18.32	10.88	2.20	91.70	91.10	29131.29	89298.54
	2011	75.95	66.43	9.52	3033.56	95.59	3.28	82.99	48.06	42.08	1.38	1.90	18.31	11.44	2.17	92.70	92.20	29822.78	89337.33
	2012	76.30	66.73	9.57	3286.01	93.34	3.21	78.47	48.65	38.00	1.22	1.99	18.30	12.07	2.14	93.80	93.30	30451.83	89413.63
	2013	76.61	66.99	9.62	3371.18	120.42	3.68	95.83	41.03	57.18	2.10	1.57	18.30	12.75	2.11	94.80	94.50	-	89502.63
	2014	76.88	67.23	9.65	3506.73	127.33	3.50	95.79	42.09	56.06	1.96	1.54	18.32	13.42	2.08	95.10	95.60	-	89614.00
	2015	77.18	67.48	9.71	3642.21	-	-	-	-	-	-	-	18.36	14.06	2.06	95.10	95.60	-	89731.80
	2016	77.41	67.75	9.66	3759.23	-	-	-	-	-	-	-	18.41	14.69	-	-	-	-	89847.03
	2017	77.47	67.87	9.60	-	-	-	-	-	-	-	-	-	-	-	-	-	-	-
Sudan	1995	60.39	51.39	9.00	860.00	20.73	3.98	92.67	74.40	19.72	0.79	3.20	32.23	5.71	5.83	26.90	65.50	101975.76	88883.33
	1996	60.68	51.66	9.02	886.48	14.59	4.44	92.85	69.54	25.10	1.11	3.32	32.28	5.73	5.76	26.60	64.80	103020.19	88877.42
	1997	61.06	51.99	9.07	953.55	16.57	4.23	92.87	73.31	21.07	0.89	3.34	32.34	5.75	5.69	26.40	64.10	107153.41	88880.40
	1998	61.45	52.33	9.12	967.41	16.78	4.11	92.62	71.29	23.03	0.95	3.17	32.39	5.76	5.61	26.00	63.40	519528.30	88904.52
	1999	61.84	52.67	9.17	969.96	14.94	3.82	92.29	73.85	19.98	0.76	3.06	32.44	5.76	5.54	25.70	62.70	511594.67	88942.64
	2000	62.57	53.29	9.28	1003.00	15.04	3.23	89.94	65.20	27.51	0.89	2.34	32.50	5.76	5.47	25.40	62.00	529253.88	89035.52
	2001	63.16	53.79	9.37	1038.58	16.16	2.96	89.54	66.72	25.49	0.76	2.21	32.55	5.79	5.40	25.10	61.30	423305.60	88966.33
	2002	63.69	54.25	9.44	1074.64	18.08	2.95	89.42	65.65	26.58	0.78	2.16	32.60	5.82	5.33	24.80	60.60	516953.93	88921.47
	2003	63.74	54.31	9.42	1125.76	22.34	3.18	89.96	64.95	27.80	0.88	2.29	32.65	5.83	5.27	24.50	59.90	499185.49	88914.91
	2004	64.08	54.62	9.46	1137.56	29.00	3.39	90.11	61.41	31.85	1.08	2.31	32.71	5.84	5.20	24.30	59.20	485680.68	88924.22
	2005	64.86	55.29	9.58	1189.96	34.94	3.18	88.97	58.40	34.36	1.09	2.09	32.76	5.85	5.14	24.00	58.50	536024.45	88941.19
	2006	65.17	55.55	9.62	1275.26	54.17	3.93	91.35	61.79	32.36	1.27	2.66	32.81	5.91	5.09	23.70	57.70	453816.36	88963.94
	2007	65.57	55.87	9.71	1385.41	83.47	4.72	92.96	32.01	32.01	1.51	3.21	32.87	5.96	5.03	23.40	57.00	634374.73	88991.74
	2008	65.87	56.11	9.76	1455.32	114.68	8.17	95.81	64.95	32.21	2.63	5.54	32.92	6.01	4.98	23.10	56.30	572341.41	89051.01
	2009	66.11	56.30	9.81	1464.25	109.97	8.04	95.81	67.72	29.32	2.36	5.68	32.99	6.05	4.93	22.80	55.60	559206.07	89103.86
	2010	66.27	56.44	9.83	1476.48	118.97	7.97	95.81	63.35	33.88	2.70	5.27	33.08	6.10	4.88	22.50	54.90	487562.73	89164.66
	2011	66.45	56.57	9.88	1592.96	120.34	8.03	95.87	65.34	31.85	2.56	5.47	33.19	6.14	4.83	23.50	55.40	489798.21	89245.29

	2012	66.80	56.86	9.94	1797.11	112.65	8.20	95.87	73.99	22.83	1.87	6.33	33.32	6.18	4.77	23.50	55.50	491982.27	89315.72
	2013	67.01	57.05	9.96	1832.31	119.38	8.42	96.06	74.85	22.08	1.86	6.56	33.46	6.21	4.72	23.60	55.50	-	89429.60
	2014	67.54	57.49	10.05	1837.14	129.84	8.43	96.06	75.52	21.38	1.80	6.63	33.62	6.24	4.66	23.60	55.50	-	89535.88
	2015	67.98	57.86	10.11	1881.90	-	-	-	-	-	-	-	33.81	6.28	4.60	-	-	-	89645.77
	2016	68.30	58.17	10.13	1923.42	-	-	-	-	-	-	-	34.01	6.33	-	-	-	-	89745.58
	2017	70.30	60.01	10.29	-	-	-	-	-	-	-	-	-	-	-	-	-	-	-
Suriname	1995	70.24	61.42	8.82	5617.99	108.29	6.97	19.58	7.02	64.17	4.47	2.50	66.08	8.36	3.08	81.10	88.30	9187.75	91098.13
	1996	70.36	61.52	8.83	5609.78	140.61	7.41	13.89	8.00	42.43	3.15	4.27	66.15	8.52	3.03	81.10	88.30	9411.23	91111.21
	1997	70.34	61.50	8.83	5854.60	201.80	10.06	30.51	13.37	56.18	5.65	4.41	66.22	8.64	2.99	81.10	88.40	9293.19	91144.46
	1998	70.16	61.36	8.80	5873.95	223.29	11.13	46.14	16.64	63.94	7.12	4.01	66.30	8.72	2.94	81.20	88.40	3183.83	91178.19
	1999	69.88	61.13	8.75	5754.92	171.03	8.91	49.50	20.63	58.32	5.20	3.71	66.37	8.77	2.90	81.00	88.90	2515.96	91221.34
	2000	69.64	60.92	8.73	5685.39	178.76	9.65	44.42	18.56	58.21	5.62	4.03	66.44	8.81	2.86	80.90	89.30	2495.76	91265.00
	2001	69.36	60.70	8.66	5877.68	132.21	8.38	53.44	26.75	49.94	4.18	4.19	66.52	8.94	2.82	80.80	89.80	-	91389.65
	2002	69.24	60.60	8.64	6063.10	158.61	7.14	43.96	22.10	49.72	3.55	3.59	66.59	9.07	2.78	80.70	90.30	3139.59	91525.94
	2003	69.19	60.56	8.63	6357.90	172.64	6.62	39.81	19.63	50.68	3.36	3.27	66.66	9.20	2.75	80.60	90.80	2773.23	91659.09
	2004	69.25	60.59	8.66	6871.84	208.59	6.88	31.94	16.49	48.39	3.33	3.55	66.74	9.29	2.71	80.50	91.20	3272.31	91797.54
	2005	69.39	60.68	8.71	7109.37	247.03	6.78	30.25	14.76	51.20	3.47	3.31	66.68	9.36	2.67	80.30	91.70	2577.08	91955.14
	2006	69.51	60.80	8.72	7304.04	329.23	6.22	23.60	11.54	51.11	3.18	3.04	66.62	9.42	2.64	80.20	92.10	2536.25	91997.50
	2007	69.67	60.92	8.75	7596.08	347.26	5.92	27.15	50.26	50.26	2.98	2.95	66.55	9.46	2.61	80.00	92.60	2466.90	92046.68
	2008	69.76	60.98	8.78	7827.23	412.97	5.92	27.15	14.73	45.75	2.71	3.21	66.48	9.47	2.58	79.90	93.00	3262.84	92093.47
	2009	70.08	61.23	8.85	7978.39	463.95	6.14	27.15	13.00	52.10	3.20	2.94	66.41	9.50	2.55	79.70	93.50	3125.75	92144.08
	2010	70.24	61.35	8.89	8303.31	489.42	5.81	27.15	13.44	50.48	2.93	2.87	66.34	9.54	2.52	79.60	93.90	-	92224.25
	2011	70.45	61.52	8.93	8698.30	501.12	5.93	27.15	13.77	49.26	2.92	3.01	66.28	9.63	2.50	79.40	94.40	2598.26	92139.80
	2012	70.67	61.69	8.97	8841.04	577.35	6.09	27.16	12.81	52.83	3.22	2.87	66.21	9.72	2.47	79.30	94.80	2657.88	92085.65
	2013	70.77	61.78	8.99	9008.76	592.48	5.96	23.60	10.93	53.69	3.20	2.76	66.14	9.83	2.45	79.20	94.80	-	92042.07
	2014	70.91	61.89	9.02	8942.96	588.63	5.69	23.60	11.40	51.70	2.94	2.75	66.09	9.94	2.42	79.20	94.80	-	92010.69
	2015	71.12	62.06	9.06	8627.53	-	-	-	-	-	-	-	66.04	10.06	2.40	79.20	94.80	-	91981.54
	2016	71.34	62.24	9.10	8108.24	-	-	-	-	-	-	-	66.02	10.23	-	-	-	-	91960.61
	2017	72.04	63.15	8.90	-	-	-	-	-	-	-	-	-	-	-	-	-	-	-

Swaziland	1995	59.31	51.66	7.66	2835.04	87.47	4.96	31.37	13.46	57.10	2.83	2.13	23.05	5.63	4.80	49.50	42.70	2292.55	89191.40
	1996	56.87	49.61	7.25	2881.08	87.97	5.40	33.80	14.67	56.59	3.06	2.35	23.08	5.64	4.65	50.00	44.60	2314.19	89228.47
	1997	54.82	47.88	6.94	2904.70	87.27	5.12	31.47	14.18	54.95	2.81	2.31	23.08	5.64	4.52	50.40	46.50	2385.25	89281.69
	1998	52.16	45.63	6.52	2916.39	84.40	5.51	34.93	16.67	52.29	2.88	2.63	22.95	5.63	4.39	50.90	48.30	3994.46	89321.51
	1999	49.83	43.62	6.20	2946.11	78.70	5.33	40.91	17.86	56.35	3.00	2.33	22.82	5.61	4.28	51.40	50.10	3251.86	89359.80
	2000	47.40	41.36	6.04	2953.23	75.32	5.26	42.39	18.53	56.29	2.96	2.30	22.69	5.59	4.19	51.80	51.90	2814.74	89388.03
	2001	45.46	39.75	5.71	2952.51	64.21	5.11	42.08	17.94	57.36	2.93	2.18	22.56	5.63	4.11	52.30	53.70	2694.99	89391.22
	2002	44.19	38.70	5.50	3059.01	58.32	5.16	41.86	17.09	59.17	3.05	2.11	22.43	5.65	4.04	52.70	55.60	2760.55	89387.87
	2003	43.24	37.86	5.38	3158.83	97.24	5.71	41.49	14.45	65.18	3.72	1.99	22.30	5.64	3.98	53.20	57.40	2632.37	89390.66
	2004	42.62	37.30	5.32	3250.40	130.09	5.88	42.03	15.44	63.27	3.72	2.16	22.17	5.59	3.93	53.70	59.20	3058.63	89387.15
	2005	42.32	36.93	5.39	3411.67	159.02	6.80	42.15	13.68	67.55	4.59	2.21	22.04	5.51	3.87	54.10	61.10	2940.41	89394.44
	2006	42.93	37.47	5.46	3568.86	179.62	6.81	42.28	13.99	66.92	4.56	2.25	21.91	5.49	3.82	54.60	62.90	2646.74	89408.35
	2007	43.80	38.20	5.60	3668.48	189.69	7.05	42.22	68.34	68.34	4.82	2.23	21.78	5.45	3.76	55.10	64.80	3715.27	89404.13
	2008	44.46	38.74	5.73	3633.32	214.49	8.19	42.84	13.04	69.56	5.70	2.49	21.67	5.40	3.69	55.60	66.60	3255.84	89373.68
	2009	45.51	39.59	5.92	3622.11	226.80	8.46	43.24	13.77	68.16	5.77	2.70	21.57	5.34	3.61	56.00	68.50	3032.49	89320.25
	2010	46.91	40.68	6.23	3690.24	250.43	8.47	42.86	12.68	70.43	5.97	2.50	21.49	5.29	3.53	56.50	70.40	3378.34	89285.05
	2011	48.80	42.45	6.34	3704.14	270.44	8.61	43.14	13.23	69.32	5.97	2.64	21.43	5.29	3.44	57.00	72.30	3430.02	89209.58
	2012	50.34	43.85	6.49	3807.74	267.41	8.76	42.77	12.03	71.88	6.30	2.46	21.37	5.28	3.36	57.50	74.20	3478.03	89166.55
	2013	51.80	45.11	6.69	3977.98	272.63	9.66	42.35	10.02	76.35	7.37	2.28	21.34	5.27	3.28	57.50	74.10	-	89161.88
	2014	53.59	46.59	7.00	3980.77	247.90	9.25	42.35	10.29	75.71	7.00	2.25	21.32	5.26	3.20	57.50	74.10	-	89153.80
	2015	56.11	48.61	7.50	3923.89	-	-	-	-	-	-	-	21.31	5.25	3.14	57.50	74.10	-	89160.05
	2016	57.44	49.62	7.82	3906.26	-	-	-	-	-	-	-	21.32	5.28	-	-	-	-	89168.55
	2017	59.89	51.33	8.56	-	-	-	-	-	-	-	-	-	-	-	-	-	-	-
Sweden	1995	78.86	68.40	10.46	37686.83	2291.81	7.96	99.89	13.34	86.65	6.90	1.06	83.82	27.51	1.73	99.30	100.00	82229.84	90010.53
	1996	79.10	68.61	10.49	38198.02	2565.39	8.20	99.88	13.06	86.93	7.13	1.07	83.91	27.45	1.60	99.30	100.00	87890.35	90157.68
	1997	79.31	68.79	10.52	39283.45	2297.72	8.03	99.77	14.15	85.82	6.89	1.14	83.94	27.35	1.52	99.30	100.00	81623.78	90326.33
	1998	79.44	68.91	10.53	40921.15	2336.59	8.12	95.72	13.60	85.79	6.96	1.15	83.97	27.22	1.50	99.30	100.00	83307.06	90488.97
	1999	79.54	68.99	10.55	42741.62	2396.26	8.20	93.80	13.39	85.73	7.03	1.17	84.00	27.08	1.50	99.30	100.00	81508.42	90655.50
	2000	79.70	69.11	10.59	44693.74	2282.46	8.18	91.14	13.77	84.89	6.94	1.24	84.03	26.92	1.54	99.30	100.00	77926.24	90805.83

	2001	79.84	69.24	10.60	45270.78	2267.83	8.86	86.86	16.39	81.14	7.19	1.67	84.07	26.78	1.57	99.30	100.00	77118.80	90860.11
	2002	80.00	69.38	10.62	46059.37	2599.51	9.23	86.37	16.04	81.42	7.51	1.71	84.13	26.63	1.65	99.30	100.00	78855.44	90913.76
	2003	80.15	69.50	10.65	46983.08	3277.40	9.31	87.08	15.67	82.01	7.63	1.68	84.20	26.50	1.71	99.30	100.00	79443.95	90935.88
	2004	80.26	69.59	10.67	48820.61	3666.47	9.09	86.17	16.05	81.37	7.40	1.69	84.26	26.45	1.75	99.30	100.00	77672.93	90963.52
	2005	80.54	69.80	10.74	49996.18	3726.57	9.06	87.10	16.41	81.16	7.35	1.71	84.32	26.51	1.77	99.30	100.00	74562.61	90979.88
	2006	80.77	69.98	10.79	52046.48	3947.09	8.95	86.66	16.35	81.13	7.26	1.69	84.43	26.66	1.85	99.30	100.00	74171.14	91008.54
	2007	80.93	70.11	10.82	53421.02	4525.77	8.92	86.79	81.36	81.36	7.25	1.66	84.59	26.86	1.88	99.30	100.00	71293.17	91021.68
	2008	81.09	70.23	10.86	52711.20	4886.13	9.23	87.13	16.11	81.50	7.52	1.71	84.75	27.13	1.91	99.30	100.00	69993.28	91043.95
	2009	81.29	70.38	10.92	49554.34	4357.24	9.94	87.09	16.11	81.50	8.10	1.84	84.90	27.47	1.94	99.30	100.00	65034.07	91049.68
	2010	81.54	70.56	10.99	52076.26	4694.46	9.47	86.62	16.01	81.51	7.72	1.75	85.06	27.91	1.98	99.30	100.00	71434.71	91068.09
	2011	81.70	70.67	11.03	53061.57	6662.30	11.70	87.46	13.36	84.72	9.91	1.79	85.21	28.47	1.90	99.30	100.00	68780.33	91092.80
	2012	81.78	70.73	11.06	52519.68	6521.58	11.80	88.00	13.77	84.35	9.96	1.85	85.36	29.13	1.91	99.30	100.00	65767.79	91144.43
	2013	81.92	70.82	11.10	52722.91	7000.16	11.97	88.07	14.09	84.00	10.05	1.91	85.51	29.84	1.89	99.30	100.00	-	91200.37
	2014	82.09	70.93	11.16	53561.89	6807.72	11.93	88.07	14.06	84.03	10.02	1.91	85.67	30.51	1.88	99.30	100.00	-	91270.31
	2015	82.07	70.92	11.16	55395.06	-	-	-	-	-	-	-	85.82	31.06	1.88	99.30	100.00	-	91345.81
	2016	82.05	70.88	11.16	56586.86	-	-	-	-	-	-	-	85.96	31.60	-	-	-	-	91434.04
	2017	82.50	70.93	11.57	-	-	-	-	-	-	-	-	-	-	-	-	-	-	-
Switzerland	1995	78.53	67.88	10.65	61773.10	4308.48	9.33	71.27	33.10	53.56	5.00	4.33	73.58	21.69	1.48	99.90	100.00	54577.65	92017.06
	1996	78.98	68.21	10.77	61833.01	4292.36	9.72	69.18	31.50	54.47	5.29	4.42	73.53	21.94	1.50	99.90	100.00	54921.84	92053.22
	1997	79.25	68.40	10.85	63127.74	3717.92	9.67	71.94	32.41	54.95	5.31	4.36	73.48	22.17	1.48	99.90	100.00	53612.51	92104.61
	1998	79.51	68.58	10.93	64822.29	3869.85	9.85	72.50	32.87	54.66	5.38	4.46	73.42	22.39	1.47	99.90	100.00	54369.68	92170.90
	1999	79.78	68.75	11.03	65607.21	3841.31	10.00	74.40	33.38	55.14	5.52	4.49	73.37	22.58	1.48	99.90	100.00	54250.62	92236.09
	2000	79.97	68.85	11.12	67807.93	3540.86	9.91	73.99	32.98	55.43	5.49	4.42	73.32	22.75	1.50	99.90	100.00	53063.22	92329.21
	2001	80.21	69.07	11.14	68264.53	3751.84	10.28	73.80	31.79	56.93	5.85	4.43	73.32	22.87	1.38	99.90	100.00	54075.37	92412.69
	2002	80.40	69.25	11.15	67860.24	4190.45	10.61	74.68	31.56	57.74	6.12	4.48	73.36	22.96	1.39	99.90	100.00	52997.42	92492.50
	2003	80.62	69.45	11.17	67385.30	5001.95	10.93	75.93	31.62	58.35	6.38	4.55	73.40	23.04	1.39	99.90	100.00	54552.75	92566.86
	2004	81.03	69.77	11.25	68781.61	5570.16	10.96	76.57	31.86	58.39	6.40	4.56	73.44	23.14	1.42	99.90	100.00	55222.56	92626.71
	2005	81.28	69.98	11.30	70471.44	5636.55	10.86	75.56	30.63	59.46	6.46	4.40	73.47	23.29	1.42	99.90	100.00	55882.85	92693.63
	2006	81.48	70.16	11.31	72823.84	5642.67	10.39	75.33	30.79	59.12	6.14	4.25	73.51	23.50	1.44	99.90	100.00	55762.01	92727.99

	2007	81.69	70.36	11.34	75143.70	6125.97	10.21	75.10	59.08	59.08	6.03	4.18	73.55	23.76	1.46	99.90	100.00	54112.49	92755.87
	2008	81.92	70.55	11.37	75793.63	7103.96	10.29	71.27	24.84	65.15	6.70	3.59	73.59	24.06	1.48	99.90	100.00	56634.89	92769.53
	2009	82.06	70.68	11.38	73189.19	7276.70	11.00	71.58	24.67	65.54	7.21	3.79	73.63	24.42	1.50	99.90	100.00	55245.97	92780.39
	2010	82.30	70.88	11.41	74605.72	7807.62	11.07	75.57	26.38	64.11	7.10	3.86	73.66	24.84	1.52	99.90	100.00	57154.18	92783.23
	2011	82.54	71.14	11.41	75029.76	9386.51	11.21	75.78	26.41	64.22	7.20	3.91	73.70	25.18	1.52	99.90	100.00	53066.87	92761.52
	2012	82.66	71.30	11.36	74984.14	9195.75	11.59	79.38	27.24	64.70	7.50	3.98	73.74	25.57	1.52	99.90	100.00	54108.10	92753.37
	2013	82.80	71.47	11.34	75499.71	9471.54	11.71	78.21	25.77	66.06	7.73	3.86	73.79	25.99	1.52	99.90	100.00	-	92745.80
	2014	83.09	71.74	11.35	76410.86	9673.52	11.66	78.82	26.80	66.00	7.70	3.96	73.84	26.40	1.54	99.90	100.00	-	92734.82
	2015	83.16	71.86	11.30	76472.46	-	-	-	-	-	-	-	73.91	26.77	1.54	99.90	100.00	-	92728.00
	2016	83.18	71.93	11.24	76694.00	-	-	-	-	-	-	-	73.99	27.23	-	-	-	-	92738.64
	2017	83.96	71.98	11.98	-	-	-	-	-	-	-	-	-	-	-	-	-	-	-
Syrian Arab	1995	72.07	61.94	10.13	-	51.11	5.52	100.00	60.32	39.68	2.19	3.33	50.10	6.01	4.51	85.40	86.10	60581.46	87449.28
	1996	72.26	62.15	10.10	-	58.61	5.50	100.00	64.47	35.53	1.95	3.55	50.47	6.03	4.41	86.00	86.40	62105.01	87469.08
	1997	72.41	62.33	10.08	-	57.93	5.30	100.00	64.56	35.44	1.88	3.42	50.84	6.04	4.32	86.70	86.70	62190.79	87514.25
	1998	72.59	62.53	10.07	-	55.35	5.12	100.00	61.73	38.27	1.96	3.16	51.21	6.04	4.23	87.40	87.00	66648.61	87570.66
	1999	72.90	62.80	10.10	-	57.28	5.25	100.00	61.63	38.37	2.01	3.24	51.58	6.03	4.14	88.00	87.20	67571.17	87627.17
	2000	73.24	63.12	10.12	-	57.89	4.92	100.00	59.55	40.45	1.99	2.93	51.95	6.00	4.04	88.60	87.50	68163.27	87701.04
	2001	73.62	63.43	10.19	-	56.91	4.92	100.00	55.79	44.21	2.17	2.74	52.31	5.97	3.94	89.30	87.80	65587.33	87723.23
	2002	73.90	63.68	10.22	-	57.17	4.94	100.00	54.23	45.77	2.26	2.68	52.68	5.93	3.84	89.90	88.00	66236.36	87782.94
	2003	74.19	63.93	10.26	-	61.07	5.12	100.00	51.75	48.25	2.47	2.65	53.05	5.89	3.74	90.50	88.20	66146.20	87866.33
	2004	74.44	64.14	10.30	-	61.74	4.48	100.00	52.03	47.97	2.15	2.33	53.42	5.86	3.65	91.10	88.50	66910.84	87949.58
	2005	74.79	64.44	10.35	-	63.78	4.11	100.00	49.50	50.50	2.08	2.03	53.78	5.85	3.56	91.70	88.70	67605.91	88092.69
	2006	75.02	64.62	10.40	-	66.96	3.78	100.00	51.46	48.54	1.83	1.94	54.15	5.82	3.47	92.30	88.90	71044.97	88249.16
	2007	75.23	64.74	10.49	-	77.48	3.72	100.00	48.08	48.08	1.79	1.93	54.53	5.80	3.39	92.90	89.10	73073.07	88403.46
	2008	75.38	64.85	10.53	-	88.85	3.40	100.00	53.01	46.99	1.60	1.80	54.91	5.79	3.33	93.50	89.30	70097.54	88549.15
	2009	75.64	65.05	10.60	-	93.06	3.55	100.00	54.00	46.00	1.63	1.92	55.29	5.78	3.26	94.00	89.50	69995.66	88666.30
	2010	76.04	65.37	10.67	-	95.14	3.28	100.00	54.00	46.00	1.51	1.77	55.68	5.77	3.21	94.60	89.70	73021.04	88768.07
	2011	74.86	64.36	10.49	-	85.53	3.23	100.00	53.38	46.62	1.51	1.73	56.07	5.99	3.16	95.20	89.90	75181.26	88727.39
	2012	68.02	58.86	9.17	-	70.98	3.25	100.00	53.69	46.31	1.51	1.75	56.46	6.21	3.11	95.70	90.10	77118.71	88734.21

	2013	68.79	59.43	9.36	-	68.83	3.25	100.00	53.69	46.31	1.51	1.75	56.86	6.43	3.07	95.70	90.10	-	88789.54
	2014	66.72	57.79	8.93	-	66.45	3.25	100.00	53.69	46.31	1.51	1.75	57.26	6.69	3.02	95.70	90.10	-	88925.75
	2015	68.01	58.80	9.21	-	-	-	-	-	-	-	-	57.66	6.98	2.97	95.70	90.10	-	89147.17
	2016	67.99	58.84	9.15	-	-	-	-	-	-	-	-	58.06	7.15	-	-	-	-	89505.75
	2017	69.79	59.79	9.99	-	-	-	-	-	-	-	-	-	-	-	-	-	-	-
Tajikistan	1995	63.81	56.18	7.63	445.16	3.01	3.07	99.19	57.48	42.06	1.29	1.78	28.88	7.44	4.59	89.50	57.70	12715.77	88187.50
	1996	62.92	55.47	7.45	365.44	5.53	3.10	97.85	56.98	41.77	1.30	1.81	28.34	7.41	4.46	89.50	57.40	12376.24	88365.47
	1997	64.30	56.65	7.65	366.34	5.64	3.63	97.15	54.34	44.07	1.60	2.03	27.81	7.29	4.33	89.50	57.20	11524.87	88539.34
	1998	64.35	56.72	7.63	380.16	7.81	3.56	97.58	66.29	32.07	1.14	2.42	27.28	7.10	4.19	89.80	57.90	11512.48	88732.81
	1999	64.84	57.17	7.67	388.22	6.91	3.88	96.99	70.95	26.84	1.04	2.84	26.76	6.89	4.05	90.10	58.70	11332.11	88954.01
	2000	65.16	57.45	7.71	413.59	7.23	4.64	99.00	78.81	20.39	0.95	3.69	26.49	6.68	3.91	90.40	59.60	10059.40	89196.33
	2001	65.45	57.75	7.71	447.78	7.81	4.59	99.17	78.63	20.71	0.95	3.64	26.46	6.72	3.78	90.70	60.60	10365.75	89287.23
	2002	65.83	58.10	7.73	486.87	8.48	4.48	98.94	78.92	20.24	0.91	3.57	26.43	6.78	3.68	91.10	61.50	10320.59	89382.49
	2003	66.26	58.49	7.77	529.81	10.63	4.46	97.55	77.64	20.41	0.91	3.55	26.40	6.81	3.59	91.40	62.50	10832.78	89454.28
	2004	66.75	58.93	7.82	572.54	15.78	5.07	97.68	79.51	18.61	0.94	4.13	26.42	6.77	3.53	91.70	63.60	11882.39	89527.90
	2005	67.25	59.37	7.88	598.30	19.96	5.89	97.25	78.35	19.43	1.14	4.74	26.43	6.64	3.50	92.10	64.60	12218.02	89583.17
	2006	67.93	59.93	8.00	626.80	23.16	5.70	97.49	78.19	19.80	1.13	4.57	26.45	6.48	3.49	92.40	65.60	13240.00	89561.15
	2007	68.44	60.35	8.10	661.34	28.00	5.35	93.83	22.16	22.16	1.18	4.16	26.47	6.28	3.49	92.70	66.60	14531.79	89527.41
	2008	68.75	60.62	8.13	698.23	39.74	5.58	95.84	72.30	24.56	1.37	4.21	26.48	6.05	3.49	93.00	67.60	14274.60	89486.43
	2009	69.24	61.01	8.23	708.95	39.44	5.93	90.31	67.79	24.94	1.48	4.45	26.50	5.84	3.50	93.40	68.60	14128.88	89438.61
	2010	69.51	61.24	8.27	738.35	44.96	6.04	90.81	66.85	26.39	1.59	4.45	26.52	5.66	3.51	93.70	69.70	14727.21	89378.58
	2011	69.72	61.43	8.29	775.30	50.16	5.98	86.01	61.42	28.59	1.71	4.27	26.53	5.54	3.50	94.00	70.70	15285.82	89306.61
	2012	70.36	61.93	8.43	814.78	61.19	6.39	85.08	60.09	29.37	1.88	4.51	26.57	5.45	3.49	94.40	71.70	15364.58	89224.95
	2013	70.94	62.38	8.56	855.51	70.80	6.75	86.65	60.12	30.62	2.07	4.68	26.62	5.38	3.47	94.70	72.70	-	89150.39
	2014	71.25	62.62	8.62	892.64	76.39	6.88	86.69	61.69	28.83	1.98	4.90	26.69	5.36	3.44	95.00	73.70	-	89094.11
	2015	71.51	62.84	8.67	925.62	-	-	-	-	-	-	-	26.78	5.40	3.40	95.00	73.80	-	89044.07
	2016	71.76	63.09	8.67	968.39	-	-	-	-	-	-	-	26.89	5.51	-	-	-	-	89000.88
	2017	70.32	61.32	9.01	-	-	-	-	-	-	-	-	-	-	-	-	-	-	-
Tanzania	1995	52.89	46.18	6.71	459.17	6.77	3.01	83.47	45.60	45.37	1.37	1.65	20.54	5.33	5.88	7.80	54.20	90916.00	88602.50

	1996	52.64	46.01	6.64	466.81	7.50	2.78	83.47	48.17	42.30	1.17	1.60	20.89	5.37	5.83	8.10	54.20	79397.97	88601.95
	1997	52.48	45.89	6.59	470.72	8.64	2.78	83.47	49.58	40.60	1.13	1.65	21.24	5.40	5.78	8.40	54.30	76244.39	88607.16
	1998	52.47	45.90	6.57	475.93	9.40	2.54	83.47	50.34	39.69	1.01	1.53	21.59	5.41	5.74	8.70	54.30	286147.05	88625.37
	1999	52.53	45.96	6.57	486.41	9.99	2.67	83.47	48.47	41.93	1.12	1.55	21.95	5.42	5.71	9.00	54.30	157566.04	88652.54
	2000	52.85	46.23	6.63	497.20	10.12	2.64	83.47	47.26	43.38	1.15	1.50	22.31	5.41	5.69	9.30	54.40	215939.36	88673.47
	2001	53.36	46.68	6.67	512.97	12.46	3.28	85.69	39.41	54.00	1.77	1.51	22.67	5.48	5.68	9.60	54.40	196701.20	88438.79
	2002	53.83	47.12	6.71	534.70	13.82	3.59	87.59	36.19	58.69	2.11	1.48	23.04	5.54	5.67	9.90	54.40	183662.08	88234.59
	2003	54.30	47.55	6.75	555.50	16.39	4.06	87.31	31.90	63.46	2.57	1.48	23.61	5.60	5.66	10.20	54.50	259727.88	88042.22
	2004	54.79	47.99	6.80	581.77	17.70	4.10	80.11	29.93	62.65	2.57	1.53	24.22	5.65	5.65	10.60	54.60	221348.89	87871.77
	2005	55.30	48.45	6.85	610.82	21.57	4.66	69.07	25.15	63.59	2.97	1.70	24.85	5.69	5.64	11.00	54.70	269558.99	87719.94
	2006	56.00	49.10	6.91	620.06	31.22	6.86	54.31	16.62	69.40	4.76	2.10	25.48	5.76	5.62	11.40	54.80	167500.80	87620.36
	2007	56.98	49.94	7.04	651.90	24.43	4.72	39.63	64.40	64.40	3.04	1.68	26.12	5.82	5.58	11.80	54.90	202224.85	87532.65
	2008	57.97	50.82	7.16	666.81	26.94	4.21	38.98	16.45	57.80	2.44	1.78	26.78	5.87	5.54	12.20	55.00	201404.52	87460.20
	2009	58.58	51.36	7.22	680.81	25.68	3.97	38.51	18.06	53.09	2.11	1.86	27.44	5.91	5.49	12.60	55.10	231456.25	87396.73
	2010	59.29	51.99	7.30	701.60	36.09	5.30	52.43	31.91	39.14	2.07	3.22	28.11	5.96	5.43	13.10	55.20	234147.16	87346.06
	2011	59.93	52.64	7.29	733.67	40.73	5.72	52.45	29.64	43.49	2.49	3.23	28.80	5.98	5.36	13.60	55.30	234762.03	87372.34
	2012	60.66	53.34	7.33	747.66	45.64	5.72	42.88	21.97	48.77	2.79	2.93	29.49	6.00	5.29	14.00	55.40	235353.12	87411.74
	2013	61.72	54.28	7.44	777.40	49.17	5.57	43.32	23.37	46.06	2.57	3.00	30.20	6.02	5.22	14.50	55.40	-	87444.54
	2014	62.60	55.05	7.55	806.15	51.72	5.58	43.32	23.21	46.41	2.59	2.99	30.90	6.03	5.15	15.00	55.50	-	87525.39
	2015	63.45	55.79	7.66	835.97	-	-	-	-	-	-	-	31.61	6.02	5.08	15.60	55.60	-	87615.85
	2016	64.29	56.53	7.76	866.95	-	-	-	-	-	-	-	32.32	6.00	-	-	-	-	87703.12
	2017	66.72	58.23	8.49	-	-	-	-	-	-	-	-	-	-	-	-	-	-	-
Thailand	1995	70.77	62.08	8.69	3530.29	100.12	3.53	80.36	42.59	47.01	1.66	1.87	30.28	8.11	1.87	89.10	89.60	281623.81	89391.45
	1996	70.93	62.21	8.72	3688.93	116.71	3.84	80.44	42.49	47.18	1.81	2.03	30.45	8.35	1.82	89.60	90.10	295588.19	89480.72
	1997	71.06	62.32	8.74	3545.39	99.60	4.00	80.07	36.88	53.95	2.16	1.84	30.62	8.60	1.78	90.00	90.50	300754.97	89588.42
	1998	71.23	62.46	8.77	3235.73	68.22	3.74	78.29	35.37	54.82	2.05	1.69	30.80	8.85	1.74	90.50	91.00	400464.25	89726.36
	1999	71.49	62.66	8.83	3345.16	69.18	3.50	76.50	34.51	54.89	1.92	1.58	30.97	9.12	1.71	90.90	91.40	316589.12	89869.12
	2000	71.89	62.98	8.91	3458.05	66.47	3.40	76.89	33.72	56.15	1.91	1.49	31.39	9.41	1.67	91.30	91.90	283870.04	90027.31
	2001	72.33	63.36	8.97	3544.21	60.41	3.32	75.82	33.07	56.38	1.87	1.45	32.57	9.71	1.64	91.70	92.40	287255.60	90131.12

	2002	72.77	63.75	9.02	3731.03	73.20	3.70	74.55	27.20	63.52	2.35	1.35	33.78	10.02	1.62	92.10	92.90	322454.08	90257.45
	2003	73.23	64.16	9.07	3969.42	78.83	3.58	74.15	26.85	63.79	2.28	1.30	35.01	10.34	1.60	92.50	93.30	324192.72	90374.69
	2004	73.34	64.28	9.06	4190.03	86.68	3.51	74.34	26.09	64.91	2.28	1.23	36.26	10.65	1.58	92.80	93.80	380171.27	90495.18
	2005	74.11	64.98	9.13	4337.26	95.01	3.55	76.49	27.23	64.39	2.29	1.26	37.52	10.94	1.57	93.20	94.30	364315.70	90621.19
	2006	74.60	65.41	9.19	4525.16	109.32	3.49	63.67	17.38	72.70	2.54	0.95	38.80	11.21	1.56	93.50	94.70	349980.87	90726.56
	2007	75.05	65.75	9.31	4744.33	132.66	3.56	61.25	76.33	76.33	2.72	0.84	40.10	11.48	1.56	93.50	95.20	388568.00	90830.23
	2008	75.55	66.15	9.40	4800.81	160.94	3.92	60.93	14.69	75.89	2.98	0.95	41.42	11.75	1.55	93.50	95.60	357708.28	90940.29
	2009	76.05	66.54	9.50	4743.69	162.97	4.11	59.59	15.37	74.21	3.05	1.06	42.74	12.05	1.55	93.40	96.00	363782.11	91062.60
	2010	76.53	66.97	9.56	5075.30	182.14	3.81	55.86	14.19	74.59	2.84	0.97	44.08	12.39	1.55	93.30	96.40	421227.83	91179.68
	2011	76.86	67.17	9.69	5093.58	212.86	4.12	55.76	12.43	77.70	3.20	0.92	45.39	12.80	1.54	93.30	96.80	431312.31	91277.71
	2012	77.13	67.39	9.74	5437.24	227.19	4.17	56.86	12.57	77.90	3.25	0.92	46.68	13.23	1.53	93.20	97.10	440411.68	91375.00
	2013	77.28	67.52	9.76	5561.29	229.88	4.00	53.76	12.21	77.30	3.09	0.91	47.94	13.70	1.52	93.10	97.50	-	91473.78
	2014	77.45	67.65	9.79	5589.70	227.52	4.12	53.79	11.92	77.83	3.21	0.91	49.17	14.22	1.51	93.00	97.80	-	91575.18
	2015	77.58	67.76	9.82	5733.92	-	-	-	-	-	-	-	50.37	14.79	1.50	93.00	97.80	-	91684.66
	2016	77.70	67.94	9.77	5901.88	-	-	-	-	-	-	-	51.54	15.34	-	-	-	-	91796.26
	2017	78.11	68.46	9.65	-	-	-	-	-	-	-	-	-	-	-	-	-	-	-
The Russian	1995	64.34	56.38	7.96	5919.34	113.31	5.36	64.66	16.89	73.88	3.96	1.40	73.37	18.25	1.34	72.60	94.20	2645144.69	92013.48
	1996	65.63	57.42	8.21	5714.55	146.98	5.55	63.21	18.06	71.44	3.97	1.59	73.37	18.40	1.27	72.60	94.30	2718564.86	92207.05
	1997	66.80	58.35	8.46	5804.14	194.57	7.10	61.90	18.08	70.79	5.03	2.07	73.36	18.30	1.22	72.50	94.40	2457479.79	92374.55
	1998	67.09	58.56	8.53	5505.63	121.70	6.62	65.92	23.01	65.09	4.31	2.31	73.36	18.08	1.23	72.50	94.60	3459685.66	92551.41
	1999	65.87	57.55	8.32	5876.15	77.28	5.80	72.09	27.49	61.86	3.59	2.21	73.35	17.93	1.16	72.50	94.70	2512656.97	92714.78
	2000	65.28	57.03	8.25	6491.00	96.19	5.42	74.70	29.97	59.88	3.25	2.18	73.35	17.95	1.20	72.50	94.90	2771222.00	92864.62
	2001	65.28	57.17	8.11	6850.52	119.11	5.67	73.74	30.48	58.66	3.33	2.35	73.35	18.31	1.22	72.40	95.00	2649244.38	92873.63
	2002	65.13	57.10	8.03	7208.57	142.17	5.99	75.19	30.86	58.95	3.53	2.46	73.34	18.80	1.29	72.40	95.20	2984844.66	92857.89
	2003	65.18	57.16	8.02	7769.67	167.37	5.61	79.71	32.83	58.81	3.30	2.31	73.37	19.29	1.32	72.40	95.30	3542027.45	92819.55
	2004	65.46	57.44	8.03	8360.81	213.48	5.19	82.24	33.24	59.58	3.09	2.10	73.42	19.55	1.34	72.40	95.50	2413520.92	92793.83
	2005	65.61	57.58	8.03	8927.91	276.91	5.21	82.39	31.32	61.98	3.23	1.98	73.46	19.46	1.29	72.40	95.60	2527172.17	92789.00
	2006	66.85	58.58	8.27	9687.49	367.68	5.30	81.50	29.97	63.23	3.35	1.95	73.51	19.38	1.31	72.30	95.80	2748951.04	92718.23
	2007	67.56	59.12	8.43	10532.33	490.02	5.38	83.02	64.18	64.18	3.45	1.93	73.55	19.06	1.42	72.30	95.90	2604102.33	92634.14

	2008	67.87	59.37	8.50	11089.93	718.67	6.22	91.22	39.63	56.55	3.52	2.70	73.60	18.64	1.50	72.30	96.10	2995286.60	92531.84
	2009	68.85	60.12	8.73	10219.52	631.99	7.44	93.86	41.41	55.88	4.16	3.28	73.64	18.32	1.54	72.30	96.20	2562920.98	92433.49
	2010	69.22	60.45	8.77	10674.99	727.37	6.83	94.39	43.30	54.12	3.70	3.13	73.69	18.17	1.57	72.30	96.30	2603289.63	92359.53
	2011	69.86	60.91	8.96	11121.51	879.60	6.61	94.63	43.36	54.18	3.58	3.03	73.73	18.27	1.58	72.30	96.50	2777724.31	92261.17
	2012	70.45	61.37	9.08	11493.40	963.49	6.88	94.92	42.55	55.18	3.80	3.08	73.79	18.45	1.69	72.20	96.60	2803398.49	92181.59
	2013	70.76	61.63	9.13	11615.70	1023.90	7.09	95.20	45.43	52.28	3.71	3.38	73.85	18.68	1.71	72.20	96.80	-	92110.31
	2014	70.86	61.73	9.13	11493.73	892.85	7.07	95.92	45.85	52.20	3.69	3.38	73.92	18.97	1.75	72.20	96.90	-	92048.21
	2015	70.83	61.76	9.08	11144.60	-	-	-	-	-	-	-	74.01	19.36	1.75	72.20	96.90	-	92030.83
	2016	70.87	61.91	8.96	11099.17	-	-	-	-	-	-	-	74.10	20.01	-	-	-	-	92048.77
	2017	72.08	62.16	9.92	-	-	-	-	-	-	-	-	-	-	-	-	-	-	-
Togo	1995	57.17	49.63	7.55	484.94	13.45	3.99	83.46	51.78	37.96	1.51	2.48	30.71	5.78	5.76	11.50	50.50	12779.71	89650.90
	1996	56.95	49.46	7.49	512.88	14.40	4.01	83.57	52.78	36.84	1.48	2.53	31.14	5.70	5.67	11.50	51.10	13188.63	89684.95
	1997	56.47	49.08	7.39	568.98	13.83	3.88	83.41	56.22	32.59	1.26	2.61	31.58	5.60	5.59	11.40	51.70	13225.65	89737.67
	1998	56.22	48.88	7.34	538.73	12.88	3.92	82.47	50.40	38.89	1.52	2.39	32.02	5.50	5.52	11.40	52.30	29197.54	89776.95
	1999	56.44	49.04	7.40	535.32	12.86	4.01	83.23	53.71	35.47	1.42	2.58	32.46	5.39	5.46	11.40	53.00	19898.01	89822.10
	2000	56.33	48.92	7.41	515.67	11.52	4.35	84.70	57.01	32.69	1.42	2.93	32.91	5.29	5.41	11.40	53.60	22054.71	89861.34
	2001	56.19	48.86	7.33	493.25	11.40	4.29	84.05	59.49	29.22	1.25	3.03	33.36	5.26	5.36	11.40	54.20	18097.70	89867.67
	2002	56.24	48.94	7.30	475.70	11.65	4.07	80.25	62.37	22.28	0.91	3.16	33.81	5.21	5.32	11.40	54.80	20990.36	89884.72
	2003	56.02	48.79	7.23	486.31	15.82	5.00	80.55	57.32	28.85	1.44	3.56	34.27	5.15	5.28	11.40	55.40	20323.80	89884.07
	2004	56.40	49.11	7.29	483.77	18.04	5.06	79.43	53.00	33.28	1.68	3.38	34.72	5.10	5.23	11.40	56.00	14388.00	89907.78
	2005	56.41	49.12	7.29	476.67	19.59	5.17	79.25	50.16	36.71	1.90	3.27	35.19	5.03	5.18	11.40	56.70	32080.96	89929.43
	2006	56.99	49.66	7.33	482.86	21.34	5.56	78.49	46.60	40.64	2.26	3.30	35.65	5.04	5.12	11.40	57.30	17748.18	89931.86
	2007	57.14	49.85	7.29	480.78	23.73	5.55	78.15	38.90	38.90	2.16	3.39	36.12	5.03	5.06	11.40	57.90	19389.96	89939.03
	2008	57.39	50.10	7.29	478.36	29.92	5.73	78.68	45.51	42.16	2.42	3.32	36.59	5.01	5.00	11.50	58.60	27174.08	89962.78
	2009	58.04	50.68	7.37	481.96	33.75	6.64	77.11	39.02	49.40	3.28	3.36	37.06	4.99	4.94	11.50	59.20	23856.09	89977.70
	2010	58.74	51.28	7.46	487.92	26.65	5.37	74.70	47.72	36.12	1.94	3.43	37.53	4.96	4.87	11.50	59.80	22578.12	90011.02
	2011	59.63	52.15	7.48	498.24	29.80	5.21	75.65	51.61	31.78	1.66	3.55	38.01	4.99	4.80	11.50	60.50	22760.64	90028.29
	2012	60.35	52.84	7.52	508.54	29.67	5.11	75.01	46.42	38.11	1.95	3.16	38.49	5.02	4.73	11.50	61.10	22931.86	90067.56
	2013	60.92	53.37	7.55	514.94	32.14	5.12	74.64	43.32	41.97	2.15	2.97	38.98	5.04	4.66	11.60	61.80	-	90096.01

	2014	61.47	53.87	7.60	531.16	33.89	5.25	75.13	46.24	38.45	2.02	3.23	39.47	5.05	4.59	11.60	62.40	-	90144.35
	2015	62.06	54.40	7.66	544.90	-	-	-	-	-	-	-	39.96	5.05	4.52	11.60	63.10	-	90186.79
	2016	62.59	54.87	7.72	558.12	-	-	-	-	-	-	-	40.46	5.09	-	-	-	-	90228.81
	2017	64.37	55.84	8.53	-	-	-	-	-	-	-	-	-	-	-	-	-	-	-
Tonga	1995	68.36	59.75	8.61	3035.55	87.25	4.13	77.47	27.38	64.66	2.67	1.46	22.86	9.73	4.45	93.70	98.60	150.45	85258.00
	1996	68.45	59.82	8.63	3026.63	96.22	4.21	77.47	26.06	66.36	2.80	1.42	22.90	9.92	4.39	93.50	98.60	154.37	85317.99
	1997	68.59	59.92	8.66	3011.53	98.44	4.48	77.47	24.88	67.88	3.04	1.44	22.93	10.02	4.34	93.40	98.60	152.25	85413.31
	1998	68.67	59.98	8.69	3080.35	92.01	4.73	77.47	24.82	67.96	3.21	1.51	22.96	10.11	4.30	93.20	98.60	142.26	85483.22
	1999	68.66	59.97	8.70	3184.97	102.82	5.14	77.47	20.79	73.17	3.76	1.38	22.98	10.18	4.27	93.10	98.60	140.79	85554.74
	2000	68.72	59.99	8.73	3275.81	91.61	4.75	77.47	22.88	70.47	3.35	1.40	23.01	10.24	4.25	93.00	98.60	175.12	85605.57
	2001	68.86	60.11	8.75	3375.59	96.03	5.66	77.47	18.82	75.71	4.28	1.37	23.04	10.38	4.24	92.80	98.60	174.84	85696.78
	2002	68.97	60.20	8.78	3470.83	92.36	5.03	77.47	18.82	75.71	3.81	1.22	23.07	10.53	4.22	92.70	98.60	150.11	85743.63
	2003	69.07	60.27	8.80	3522.53	106.14	5.09	62.94	10.59	83.17	4.23	0.86	23.10	10.67	4.20	92.50	98.60	158.06	85782.55
	2004	69.20	60.36	8.84	3488.58	114.81	4.87	62.94	10.59	83.17	4.05	0.82	23.13	10.76	4.18	92.40	98.60	152.49	85830.72
	2005	69.43	60.53	8.90	3521.51	167.36	6.52	62.26	8.05	87.07	5.68	0.84	23.16	10.81	4.14	92.30	98.70	153.79	85863.00
	2006	69.61	60.67	8.94	3470.65	159.05	5.61	62.26	8.07	87.04	4.88	0.73	23.18	10.80	4.10	92.10	98.80	152.77	85873.12
	2007	69.65	60.69	8.96	3304.36	170.63	5.82	67.82	81.87	81.87	4.76	1.05	23.23	10.75	4.06	92.00	98.90	153.47	85899.79
	2008	69.68	60.72	8.97	3385.35	169.65	5.13	67.82	12.47	81.61	4.19	0.94	23.28	10.63	4.01	91.80	99.00	147.59	85942.93
	2009	67.86	59.23	8.63	3442.87	145.05	4.67	67.82	13.99	79.36	3.71	0.96	23.34	10.49	3.96	91.70	99.10	147.55	86014.78
	2010	69.82	60.81	9.01	3548.07	163.44	4.59	67.82	13.27	80.43	3.69	0.90	23.39	10.32	3.91	91.60	99.20	151.93	86091.40
	2011	69.91	60.88	9.04	3631.54	171.63	4.06	67.82	13.98	79.39	3.22	0.84	23.44	10.32	3.86	91.40	99.30	155.29	86207.20
	2012	70.00	60.94	9.06	3650.82	196.87	4.51	67.82	13.09	80.70	3.64	0.87	23.50	10.32	3.82	91.30	99.40	-	86351.22
	2013	70.08	61.00	9.09	3524.23	205.21	4.98	67.82	12.63	81.37	4.06	0.93	23.56	10.30	3.77	91.10	99.50	-	86517.08
	2014	70.17	61.06	9.11	3581.84	212.98	5.18	67.82	11.94	82.40	4.27	0.91	23.63	10.27	3.72	91.00	99.60	-	86693.69
	2015	70.27	61.13	9.14	3694.46	-	-	-	-	-	-	-	23.71	10.20	3.68	91.00	99.60	-	86901.93
	2016	70.39	61.22	9.17	3792.29	-	-	-	-	-	-	-	23.80	10.11	-	-	-	-	87122.26
	2017	71.77	62.30	9.47	-	-	-	-	-	-	-	-	-	-	-	-	-	-	-
Trinidad and Tobago	1995	69.23	60.66	8.57	6869.50	216.49	5.10	87.21	42.08	51.75	2.64	2.46	9.62	9.62	1.96	90.30	92.50	-	91628.62
	1996	69.21	60.66	8.55	7339.93	216.61	4.73	86.28	42.77	50.43	2.39	2.35	9.85	9.65	1.90	90.40	92.60	-	91681.76

	1997	69.34	60.75	8.59	7877.62	208.33	4.58	86.34	42.12	51.22	2.34	2.23	10.09	9.64	1.84	90.50	92.80	-	91757.69
	1998	69.46	60.83	8.63	8505.07	200.08	4.18	86.35	46.01	46.72	1.95	2.23	10.33	9.62	1.80	90.50	93.00	21180.01	91821.38
	1999	69.24	60.64	8.59	9171.36	253.42	4.71	86.91	43.62	49.81	2.34	2.36	10.58	9.61	1.77	90.60	93.10	22878.23	91915.43
	2000	69.41	60.76	8.65	9779.50	268.01	4.17	86.32	45.81	46.92	1.96	2.21	10.78	9.62	1.75	90.70	93.30	24111.81	92007.37
	2001	69.66	60.95	8.71	10151.96	305.05	4.40	86.31	42.61	50.63	2.23	2.17	10.60	9.69	1.74	90.80	93.50	25251.93	92068.43
	2002	69.92	61.16	8.77	10910.90	352.32	5.00	86.91	40.88	52.96	2.65	2.35	10.42	9.77	1.74	90.80	93.60	-	92143.85
	2003	70.40	61.51	8.89	12426.15	448.51	5.13	90.04	44.36	50.73	2.60	2.53	10.24	9.88	1.75	90.90	93.80	31439.72	92225.88
	2004	70.97	61.94	9.03	13346.59	521.58	5.22	90.14	45.28	49.76	2.60	2.62	10.07	10.03	1.76	91.00	94.00	38415.06	92298.44
	2005	71.25	62.14	9.11	14105.34	654.40	5.31	82.75	41.58	49.75	2.64	2.67	9.90	10.20	1.77	91.10	94.10	43922.04	92363.77
	2006	71.56	62.40	9.16	15892.29	632.88	4.49	82.58	41.53	49.71	2.23	2.26	9.74	10.44	1.78	91.10	94.30	52004.72	92382.15
	2007	71.97	62.70	9.27	16570.07	797.26	4.82	81.67	49.25	49.25	2.38	2.45	9.57	10.71	1.79	91.20	94.50	51308.10	92414.62
	2008	72.21	62.88	9.33	17052.26	931.88	4.40	81.32	41.30	49.21	2.16	2.23	9.41	11.01	1.80	91.30	94.60	54553.47	92462.03
	2009	72.39	63.00	9.39	16226.51	938.84	6.47	83.38	41.11	50.70	3.28	3.19	9.25	11.33	1.80	91.40	94.80	54882.34	92532.25
	2010	72.43	63.04	9.40	16683.95	818.94	5.29	81.26	34.86	57.11	3.02	2.27	9.09	11.68	1.81	91.40	95.00	58068.76	92663.39
	2011	72.53	63.11	9.42	16551.49	891.80	5.06	79.66	37.44	53.01	2.68	2.38	8.94	12.01	1.80	91.50	95.10	59776.58	92701.48
	2012	72.66	63.21	9.45	16680.68	1000.98	5.78	81.32	40.61	50.07	2.89	2.89	8.80	12.35	1.80	91.50	95.10	61308.53	92773.67
	2013	72.77	63.30	9.47	17038.79	1084.36	5.98	81.70	38.76	52.55	3.14	2.84	8.67	12.71	1.79	91.50	95.10	-	92844.03
	2014	72.86	63.37	9.49	16862.55	1136.31	5.93	81.70	37.98	53.51	3.17	2.76	8.55	13.08	1.78	91.50	95.10	-	92919.19
	2015	72.94	63.43	9.51	16695.98	-	-	-	-	-	-	-	8.45	13.48	1.77	91.50	95.10	-	93002.75
	2016	73.02	63.51	9.51	16259.04	-	-	-	-	-	-	-	8.35	13.96	-	-	-	-	93091.60
	2017	74.24	64.99	9.26	-	-	-	-	-	-	-	-	-	-	-	-	-	-	-
Tunisia	1995	72.80	62.69	10.11	2432.31	127.26	5.86	80.12	40.54	49.40	2.89	2.96	61.47	9.17	2.61	77.80	86.50	24746.42	90228.32
	1996	73.34	63.13	10.21	2566.13	126.17	5.43	85.05	38.27	55.01	2.99	2.44	61.87	9.44	2.49	78.60	87.20	26068.71	90372.95
	1997	73.85	63.53	10.31	2668.81	113.60	5.14	85.26	40.46	52.54	2.70	2.44	62.26	9.74	2.38	79.40	87.90	26847.33	90517.88
	1998	74.26	63.86	10.39	2762.55	123.90	5.40	84.98	39.02	54.08	2.92	2.48	62.65	10.05	2.29	80.30	88.60	28144.02	90685.58
	1999	74.60	64.14	10.46	2897.98	129.61	5.43	83.48	38.88	53.43	2.90	2.53	63.04	10.33	2.21	81.10	89.20	29463.86	90851.87
	2000	74.87	64.37	10.50	3004.61	119.46	5.40	80.11	36.14	54.89	2.96	2.43	63.43	10.56	2.14	81.90	89.90	30799.83	91014.09
	2001	75.13	64.58	10.55	3091.10	119.30	5.29	80.13	37.20	53.57	2.83	2.46	63.82	10.71	2.09	82.70	90.50	31281.86	91134.50
	2002	75.33	64.75	10.58	3107.02	124.79	5.32	80.17	37.95	52.66	2.80	2.52	64.20	10.83	2.05	83.40	91.10	31568.35	91274.49

	2003	75.50	64.90	10.60	3228.46	147.99	5.36	80.17	37.77	52.88	2.83	2.52	64.59	10.91	2.02	84.20	91.80	31530.91	91386.03
	2004	75.76	65.13	10.63	3403.11	175.12	5.63	83.49	40.25	51.80	2.91	2.71	64.93	10.97	2.00	85.00	92.40	32529.08	91486.12
	2005	75.98	65.32	10.66	3492.17	177.84	5.57	84.16	40.83	51.48	2.87	2.70	65.10	11.01	1.99	85.60	92.90	32172.82	91583.10
	2006	76.17	65.48	10.69	3641.54	190.30	5.64	85.54	40.76	52.35	2.96	2.69	65.27	10.97	2.00	86.30	93.50	33238.04	91618.67
	2007	76.35	65.59	10.76	3847.41	213.16	5.64	86.81	52.33	52.33	2.95	2.69	65.43	10.92	2.02	87.00	94.10	33570.52	91648.86
	2008	76.54	65.73	10.81	3968.35	242.57	5.63	86.88	39.93	54.04	3.04	2.59	65.60	10.88	2.06	87.70	94.70	35101.99	91677.73
	2009	76.68	65.84	10.84	4044.63	255.40	6.18	86.68	37.52	56.72	3.51	2.68	65.77	10.83	2.10	88.30	95.20	35556.61	91714.81
	2010	76.84	65.99	10.85	4140.15	270.94	6.54	86.52	35.89	58.52	3.83	2.71	65.93	10.80	2.14	89.00	95.80	37649.97	91754.38
	2011	76.91	66.02	10.88	4014.92	305.08	7.15	87.25	36.43	58.25	4.17	2.99	66.10	10.79	2.18	89.60	96.30	38741.14	91768.16
	2012	77.07	66.17	10.90	4127.40	297.74	7.18	87.28	36.73	57.92	4.16	3.02	66.27	10.77	2.21	90.30	96.90	39721.01	91806.82
	2013	77.18	66.28	10.90	4196.75	309.47	7.26	87.06	36.37	58.22	4.23	3.04	66.46	10.79	2.23	90.90	97.30	-	91867.31
	2014	77.27	66.37	10.90	4265.15	305.31	7.00	87.06	37.73	56.67	3.97	3.04	66.65	10.88	2.23	91.60	97.70	-	91950.97
	2015	77.33	66.43	10.90	4264.52	-	-	-	-	-	-	-	66.84	11.08	2.22	91.60	97.70	-	92052.50
	2016	77.46	66.61	10.85	4265.37	-	-	-	-	-	-	-	67.05	11.38	-	-	-	-	92155.51
	2017	78.33	67.31	11.02	-	-	-	-	-	-	-	-	-	-	-	-	-	-	-
Turkey	1995	70.82	60.55	10.28	7315.18	96.22	2.51	100.00	29.68	70.32	1.76	0.74	62.12	8.44	2.76	85.50	89.20	261203.42	89791.53
	1996	71.34	61.00	10.34	7731.17	117.55	2.90	100.00	30.84	69.16	2.01	0.90	62.65	8.67	2.71	86.00	89.90	278000.49	89859.61
	1997	71.85	61.44	10.41	8186.24	130.79	3.13	99.56	28.26	71.61	2.24	0.89	63.18	8.93	2.66	86.60	90.60	289681.10	89949.30
	1998	72.56	62.02	10.54	8244.50	155.89	3.59	99.59	27.96	71.93	2.58	1.01	63.70	9.18	2.61	87.10	91.20	295884.83	90045.88
	1999	71.91	61.54	10.36	7842.59	189.19	4.77	74.81	29.08	61.13	2.91	1.85	64.22	9.42	2.56	87.60	91.90	295553.27	90160.09
	2000	74.05	63.25	10.80	8237.34	206.75	4.95	74.57	27.65	62.93	3.11	1.83	64.74	9.62	2.50	88.10	92.60	316464.84	90293.87
	2001	74.74	63.78	10.97	7631.40	156.34	5.16	71.55	22.85	68.07	3.51	1.65	65.33	9.80	2.45	88.60	93.20	297564.11	90343.33
	2002	75.35	64.28	11.08	8003.48	189.96	5.36	67.68	19.84	70.68	3.79	1.57	65.95	9.95	2.40	89.10	93.80	310061.15	90416.51
	2003	75.44	64.39	11.05	8331.76	243.47	5.34	65.75	18.46	71.92	3.84	1.50	66.57	10.07	2.35	89.70	94.50	323781.17	90488.77
	2004	76.25	65.03	11.23	9009.59	312.93	5.37	66.91	19.24	71.25	3.83	1.54	67.18	10.20	2.31	90.10	95.10	333497.62	90556.36
	2005	76.66	65.42	11.24	9691.82	386.19	5.45	70.78	22.76	67.84	3.70	1.75	67.78	10.32	2.27	90.60	95.70	347595.11	90630.88
	2006	77.25	65.91	11.34	10251.05	447.24	5.81	69.40	21.97	68.34	3.97	1.84	68.38	10.44	2.24	91.10	96.30	377156.06	90623.23
	2007	78.17	66.54	11.63	10637.72	559.69	6.04	67.82	67.83	67.83	4.10	1.94	68.98	10.56	2.21	91.60	96.90	406298.25	90620.45
	2008	78.82	67.04	11.78	10599.29	627.89	6.07	64.41	17.38	73.02	4.44	1.64	69.56	10.68	2.19	92.00	97.50	399181.90	90618.25

	2009	78.19	66.61	11.58	9973.34	522.03	6.08	73.88	14.06	80.97	4.92	1.16	70.14	10.81	2.17	92.50	98.00	404636.72	90662.62
	2010	78.27	66.77	11.50	10672.05	565.87	5.61	76.25	16.31	78.61	4.41	1.20	70.72	10.95	2.16	92.90	98.60	422721.87	90697.39
	2011	78.30	66.74	11.56	11683.23	557.47	5.29	75.49	15.44	79.55	4.21	1.08	71.28	11.06	2.14	93.30	99.10	434798.46	90768.69
	2012	78.66	67.03	11.63	12052.33	555.06	5.24	76.28	15.84	79.24	4.15	1.09	71.83	11.19	2.12	93.70	99.50	445640.08	90865.40
	2013	78.51	66.96	11.55	12865.68	588.20	5.38	77.94	16.77	78.48	4.23	1.16	72.37	11.33	2.11	94.10	99.80	-	90967.47
	2014	78.83	67.23	11.61	13312.02	567.63	5.41	78.71	17.75	77.45	4.19	1.22	72.89	11.50	2.09	94.50	100.00	-	91083.25
	2015	79.02	67.39	11.63	13898.30	-	-	-	-	-	-	-	73.40	11.71	2.07	94.90	100.00	-	91216.77
	2016	79.16	67.60	11.56	14116.98	-	-	-	-	-	-	-	73.89	11.93	-	-	-	-	91388.54
	2017	78.94	67.88	11.06	-	-	-	-	-	-	-	-	-	-	-	-	-	-	-
Turkmenista	1995	63.10	55.86	7.23	2054.28	43.02	3.07	100.00	39.50	60.50	1.86	1.21	44.79	7.27	3.51	62.00	59.00	55244.04	87842.52
	1996	63.03	55.85	7.18	2151.27	17.47	3.13	100.00	27.83	72.17	2.26	0.87	45.02	7.31	3.32	62.10	59.10	51096.43	88018.07
	1997	62.98	55.83	7.15	1876.37	30.67	4.96	100.00	29.85	70.15	3.48	1.48	45.24	7.29	3.15	62.10	59.30	45884.63	88216.96
	1998	63.06	55.92	7.15	1983.02	32.75	5.03	100.00	30.40	69.60	3.50	1.53	45.47	7.24	3.01	62.20	59.40	45828.87	88424.24
	1999	63.25	56.08	7.17	2282.98	33.65	3.88	100.00	38.54	61.46	2.39	1.50	45.69	7.20	2.90	62.30	59.50	55973.71	88625.78
	2000	63.62	56.38	7.24	2381.18	43.98	3.94	100.00	18.46	81.54	3.21	0.73	45.91	7.20	2.82	62.30	59.60	63236.16	88825.06
	2001	63.93	56.67	7.26	2458.51	58.98	3.87	100.00	27.22	72.78	2.82	1.05	46.14	7.28	2.77	62.40	59.70	66916.35	88921.30
	2002	64.21	56.91	7.30	2440.28	63.12	3.34	100.00	27.15	72.85	2.43	0.91	46.36	7.37	2.73	62.40	59.90	70833.49	89020.90
	2003	64.51	57.17	7.33	2495.29	94.71	3.85	100.00	29.71	70.29	2.71	1.14	46.59	7.45	2.70	62.50	60.00	80037.23	89109.67
	2004	64.83	57.45	7.38	2593.52	121.91	4.03	100.00	39.97	60.03	2.42	1.61	46.81	7.43	2.67	62.60	60.10	76966.67	89203.77
	2005	65.26	57.81	7.45	2900.12	126.98	3.51	100.00	42.42	57.58	2.02	1.49	47.05	7.31	2.65	62.60	60.20	79175.65	89271.96
	2006	65.76	58.25	7.51	3181.07	129.34	2.90	100.00	46.76	53.24	1.55	1.36	47.30	7.15	2.65	62.70	60.40	81028.32	89241.49
	2007	66.29	58.69	7.60	3489.33	115.35	2.16	100.00	64.31	64.31	1.39	0.77	47.56	6.91	2.67	-	-	86686.09	89212.05
	2008	66.81	59.13	7.68	3949.05	83.60	1.93	100.00	50.82	49.18	0.95	0.98	47.83	6.65	2.71	-	-	91468.98	89162.90
	2009	67.39	59.61	7.78	4129.54	76.40	1.88	100.00	38.19	61.81	1.16	0.72	48.11	6.42	2.77	-	-	80016.14	89114.10
	2010	67.91	60.06	7.85	4439.20	87.29	1.99	100.00	38.43	61.57	1.22	0.76	48.40	6.24	2.83	-	-	87379.67	89076.01
	2011	68.37	60.45	7.92	5006.30	113.41	1.98	100.00	35.99	64.01	1.27	0.71	48.71	6.15	2.89	-	-	88648.72	88998.06
	2012	68.83	60.84	7.99	5462.98	133.36	1.96	100.00	35.09	64.91	1.27	0.69	49.02	6.10	2.94	-	-	92178.07	88934.67
	2013	69.19	61.15	8.04	5909.77	165.98	2.12	100.00	32.76	67.24	1.43	0.69	49.35	6.10	2.96	-	-	-	88879.80
	2014	69.57	61.47	8.11	6399.27	186.72	2.07	100.00	34.77	65.23	1.35	0.72	49.69	6.14	2.96	-	-	-	88852.94

	2015	69.91	61.75	8.16	6693.94	-	-	-	-	-	-	-	50.04	6.22	2.93	-	-	-	88835.30
	2016	70.24	62.06	8.18	6986.86	-	-	-	-	-	-	-	50.40	6.40	-	-	-	-	88837.12
	2017	70.10	61.67	8.43	-	-	-	-	-	-	-	-	-	-	-	-	-	-	-
Uganda	1995	46.86	40.63	6.22	359.76	18.41	5.69	61.23	45.95	24.94	1.42	4.27	11.66	5.48	7.02	14.40	48.40	38761.04	88297.86
	1996	46.90	40.73	6.17	380.34	19.16	5.67	61.23	45.97	24.91	1.41	4.26	11.75	5.52	6.99	14.60	50.00	38939.18	88114.35
	1997	47.30	41.08	6.22	387.60	19.89	5.56	61.22	45.43	25.79	1.43	4.13	11.83	5.55	6.97	14.90	51.60	39732.04	87953.46
	1998	47.63	41.36	6.27	394.25	19.79	6.20	61.55	42.73	30.57	1.90	4.31	11.91	5.55	6.94	15.10	53.20	85877.49	87791.13
	1999	48.18	41.82	6.36	412.80	18.56	6.18	56.71	41.49	26.85	1.66	4.52	12.00	5.51	6.90	15.30	54.80	58912.74	87653.36
	2000	48.66	42.22	6.44	412.20	19.02	6.77	51.80	37.75	27.14	1.84	4.94	12.08	5.43	6.87	15.60	56.40	72771.09	87510.79
	2001	49.39	42.89	6.50	419.34	20.53	7.26	47.27	34.38	27.27	1.98	5.28	12.17	5.36	6.82	15.80	58.00	57992.59	87526.00
	2002	50.13	43.56	6.57	440.65	22.40	7.78	43.98	32.08	27.05	2.11	5.68	12.25	5.26	6.77	16.00	59.60	59822.89	87532.12
	2003	50.89	44.24	6.65	453.20	22.17	7.54	41.48	30.76	25.85	1.95	5.59	12.48	5.15	6.72	16.30	61.20	89594.61	87576.14
	2004	51.73	44.99	6.74	467.48	30.28	8.90	48.23	35.95	25.47	2.27	6.63	12.76	5.03	6.65	16.50	62.80	70139.65	87600.13
	2005	52.79	45.88	6.91	480.10	36.67	9.36	52.41	39.19	25.23	2.36	7.00	13.03	4.93	6.58	16.80	64.50	111346.72	87642.95
	2006	53.69	46.70	6.99	513.75	40.97	9.86	55.25	41.40	25.07	2.47	7.39	13.31	4.87	6.51	17.00	66.10	40779.02	87738.39
	2007	54.31	47.28	7.03	538.04	48.25	9.76	56.69	25.22	25.22	2.46	7.30	13.60	4.82	6.42	17.30	67.70	104200.61	87838.59
	2008	55.09	47.98	7.11	565.07	52.68	9.10	57.81	43.19	25.29	2.30	6.80	13.89	4.77	6.34	17.50	69.30	89206.67	87959.90
	2009	56.01	48.79	7.22	583.10	49.68	8.43	53.90	42.30	21.52	1.81	6.62	14.19	4.72	6.25	17.80	70.90	52830.35	88097.98
	2010	56.79	49.47	7.32	595.21	65.33	11.02	45.34	32.52	28.28	3.12	7.91	14.49	4.65	6.15	18.00	72.50	79906.21	88246.23
	2011	57.65	50.30	7.35	629.24	57.95	9.09	49.91	35.45	28.97	2.63	6.46	14.80	4.60	6.06	18.30	74.20	80318.97	88207.39
	2012	58.60	51.19	7.42	631.56	52.79	7.58	61.36	43.93	28.40	2.15	5.43	15.12	4.55	5.96	18.50	75.80	80725.08	88182.34
	2013	59.70	52.16	7.54	632.49	52.03	7.47	54.57	39.32	27.94	2.09	5.39	15.44	4.49	5.87	18.80	77.40	-	88169.55
	2014	60.69	53.02	7.66	642.88	52.29	7.22	54.57	40.96	24.94	1.80	5.42	15.77	4.44	5.78	19.00	78.90	-	88157.26
	2015	61.59	53.80	7.79	654.14	-	-	-	-	-	-	-	16.10	4.38	5.68	19.10	79.00	-	88165.14
	2016	62.20	54.35	7.86	662.43	-	-	-	-	-	-	-	16.44	4.36	-	-	-	-	88158.76
	2017	65.71	56.96	8.74	-	-	-	-	-	-	-	-	-	-	-	-	-	-	-
Ukraine	1995	66.53	58.36	8.16	1917.45	51.05	7.01	89.26	24.46	72.59	5.09	1.92	66.95	20.32	1.40	94.50	97.50	598434.25	91976.94
	1996	66.71	58.54	8.18	1741.09	60.47	6.85	89.19	26.85	69.90	4.79	2.06	66.99	20.47	1.33	94.50	97.50	533868.64	92119.56
	1997	67.32	59.02	8.30	1704.31	68.61	6.85	91.52	35.52	61.19	4.19	2.66	67.03	20.31	1.27	94.50	97.50	514547.65	92288.92

	1998	68.10	59.63	8.47	1686.94	55.71	6.60	92.60	42.96	53.60	3.54	3.06	67.07	20.03	1.21	94.50	97.50	485491.77	92459.34
	1999	67.70	59.33	8.37	1699.52	37.82	5.89	92.23	46.18	49.93	2.94	2.95	67.11	19.88	1.12	94.60	97.60	483347.22	92642.27
	2000	67.35	59.05	8.30	1818.00	35.84	5.59	91.41	44.05	51.81	2.90	2.69	67.15	19.98	1.11	94.70	97.60	470107.96	92824.33
	2001	67.57	59.25	8.32	2005.32	44.55	5.66	91.46	41.78	54.32	3.07	2.58	67.18	20.52	1.09	94.70	97.50	459965.89	92878.88
	2002	67.67	59.34	8.32	2130.66	55.33	6.25	92.19	40.37	56.21	3.51	2.74	67.28	21.28	1.13	94.80	97.40	462724.22	92923.62
	2003	67.67	59.37	8.31	2349.93	73.19	6.93	92.82	38.70	58.30	4.04	2.89	67.43	22.08	1.17	94.90	97.40	484337.02	92939.51
	2004	67.50	59.25	8.25	2654.33	91.01	6.61	93.05	38.57	58.55	3.87	2.74	67.60	22.66	1.22	95.00	97.30	457164.88	92933.22
	2005	67.10	58.96	8.14	2746.05	118.31	6.41	92.49	37.50	59.46	3.81	2.60	67.79	22.87	1.21	95.10	97.20	434668.76	92921.05
	2006	67.82	59.53	8.29	2966.50	148.12	6.39	92.47	36.28	60.77	3.88	2.51	67.97	23.06	1.31	95.20	97.10	432631.66	92870.37
	2007	67.60	59.34	8.26	3220.01	196.11	6.36	90.82	61.85	61.85	3.93	2.42	68.15	22.96	1.35	95.30	97.00	444201.66	92815.25
	2008	67.87	59.56	8.31	3311.96	253.62	6.63	93.15	39.44	57.66	3.83	2.81	68.33	22.73	1.46	95.40	96.90	436817.13	92749.29
	2009	69.61	60.91	8.70	2834.34	199.04	7.80	93.33	41.96	55.03	4.29	3.51	68.50	22.51	1.47	95.50	96.80	367349.71	92692.24
	2010	70.50	61.65	8.85	2965.14	233.45	7.81	93.39	40.50	56.63	4.42	3.39	68.69	22.39	1.44	95.60	96.70	393092.06	92665.37
	2011	71.21	62.16	9.04	3138.47	250.58	6.98	93.64	43.64	53.40	3.73	3.25	68.88	22.42	1.46	95.70	96.60	410067.31	92623.03
	2012	71.20	62.18	9.02	3153.74	289.71	7.47	93.77	41.98	55.23	4.13	3.34	69.07	22.48	1.53	95.80	96.50	404900.30	92594.47
	2013	71.36	62.32	9.04	3160.08	311.16	7.67	93.96	43.09	54.14	4.15	3.52	69.27	22.58	1.51	95.80	96.40	-	92574.95
	2014	71.82	62.67	9.15	3123.92	202.66	7.10	93.93	46.22	50.80	3.60	3.49	69.48	22.76	1.50	95.90	96.30	-	92599.44
	2015	72.03	62.87	9.17	2828.89	-	-	-	-	-	-	-	69.70	23.01	1.51	95.90	96.20	-	92635.14
	2016	72.10	63.03	9.07	2905.86	-	-	-	-	-	-	-	69.92	23.56	-	-	-	-	92687.66
	2017	70.55	60.99	9.56	-	-	-	-	-	-	-	-	-	-	-	-	-	-	-
United Arab Emirates	1995	73.15	62.79	10.36	61818.23	737.42	2.64	71.00	14.93	78.97	2.08	0.55	78.32	1.47	3.42	97.40	99.70	93904.01	88613.73
	1996	73.33	62.96	10.37	62294.13	733.12	2.46	68.94	14.30	79.25	1.95	0.51	78.49	1.46	3.23	97.40	99.70	103034.65	88584.24
	1997	73.51	63.13	10.38	64176.48	755.51	2.49	70.56	15.28	78.34	1.95	0.54	78.94	1.47	3.06	97.40	99.70	101901.38	88594.79
	1998	73.85	63.42	10.43	61231.23	801.21	2.89	74.82	21.65	71.06	2.06	0.84	79.38	1.49	2.91	97.40	99.70	104993.20	88623.00
	1999	74.04	63.59	10.46	59845.04	794.56	2.71	74.63	21.89	70.66	1.92	0.80	79.81	1.51	2.77	97.40	99.70	107207.93	88651.69
	2000	74.33	63.84	10.49	62833.25	814.14	2.38	74.27	21.94	70.46	1.68	0.70	80.24	1.50	2.64	97.40	99.70	115420.51	88739.41
	2001	74.56	64.02	10.54	60434.67	797.59	2.48	69.43	15.09	78.27	1.94	0.54	80.66	1.40	2.53	97.40	99.70	115018.82	88820.64
	2002	74.82	64.23	10.59	58707.01	879.29	2.72	75.94	28.04	63.07	1.71	1.00	81.07	1.31	2.43	97.40	99.70	126800.21	88931.85
	2003	75.21	64.53	10.68	59867.29	907.58	2.65	74.53	27.46	63.16	1.67	0.97	81.47	1.23	2.33	97.40	99.70	134091.69	89064.05

	2004	75.65	64.89	10.76	60042.60	915.42	2.46	74.41	30.41	59.14	1.46	1.01	81.87	1.18	2.24	97.50	99.70	139999.46	89238.88
	2005	75.96	65.19	10.77	56199.03	935.02	2.32	73.51	30.14	59.00	1.37	0.95	82.26	1.14	2.15	97.50	99.70	143367.66	89462.58
	2006	76.13	65.37	10.76	53926.60	998.79	2.33	73.28	29.71	59.45	1.38	0.94	82.65	1.02	2.07	97.50	99.70	151837.32	89590.26
	2007	76.11	65.32	10.79	48260.01	1101.01	2.57	73.33	60.62	60.62	1.56	1.01	83.02	0.95	2.01	97.50	99.60	167986.43	89773.60
	2008	75.97	65.24	10.73	43658.94	1338.93	2.93	73.11	25.14	65.62	1.92	1.01	83.38	0.90	1.95	97.50	99.60	181388.54	89947.00
	2009	75.79	65.13	10.66	37203.40	1332.63	4.05	64.19	16.10	74.92	3.03	1.02	83.72	0.88	1.90	97.50	99.60	182422.17	90100.16
	2010	75.64	65.07	10.56	35049.15	1349.45	3.93	64.38	17.95	72.13	2.83	1.10	84.06	0.87	1.87	97.50	99.60	193127.16	90242.48
	2011	75.53	64.97	10.56	35550.83	1473.34	3.70	64.38	16.88	73.77	2.73	0.97	84.38	0.95	1.84	97.50	99.60	199342.52	90250.74
	2012	75.47	64.94	10.53	36408.50	1433.11	3.45	64.38	16.74	74.00	2.55	0.90	84.68	1.02	1.82	97.50	99.60	204888.72	90313.91
	2013	75.44	64.93	10.51	38064.00	1551.35	3.49	64.38	17.15	73.37	2.56	0.93	84.98	1.10	1.80	97.50	99.60	-	90409.23
	2014	75.42	64.93	10.49	39034.38	1610.80	3.64	64.38	17.81	72.34	2.64	1.01	85.27	1.16	1.78	97.50	99.60	-	90531.98
	2015	75.44	64.96	10.49	40159.56	-	-	-	-	-	-	-	85.54	1.20	1.77	97.60	99.60	-	90725.02
	2016	75.51	65.08	10.43	40864.25	-	-	-	-	-	-	-	85.80	1.28	-	-	-	-	90901.52
	2017	73.32	63.10	10.22	-	-	-	-	-	-	-	-	-	-	-	-	-	-	-
United Kingdom	1995	76.69	66.66	10.03	30674.61	1364.12	6.69	72.81	10.91	83.86	5.61	1.00	78.35	24.60	1.71	99.20	100.00	728336.09	91868.82
	1996	76.91	66.85	10.06	31373.34	1436.87	6.72	68.10	10.99	82.94	5.57	1.08	78.41	24.63	1.73	99.20	100.00	744362.82	91899.20
	1997	77.14	67.05	10.09	32556.32	1542.68	6.49	59.81	11.75	80.36	5.22	1.28	78.47	24.61	1.72	99.20	100.00	716185.48	91935.15
	1998	77.34	67.23	10.11	33480.17	1660.74	6.56	59.83	11.75	80.35	5.27	1.29	78.53	24.56	1.71	99.20	100.00	705167.71	91978.39
	1999	77.53	67.40	10.14	34442.11	1769.18	6.83	58.90	11.41	80.63	5.50	1.32	78.59	24.49	1.68	99.20	100.00	680102.48	92028.34
	2000	77.84	67.64	10.20	35576.77	1763.46	6.94	53.81	10.99	79.58	5.52	1.42	78.65	24.42	1.64	99.20	100.00	673897.41	92096.09
	2001	78.10	67.84	10.25	36341.71	1840.44	7.31	51.17	10.81	78.87	5.77	1.54	78.75	24.42	1.63	99.20	100.00	683080.09	92036.88
	2002	78.31	68.01	10.30	37077.65	2070.69	7.57	53.56	10.81	79.81	6.04	1.53	79.05	24.40	1.63	99.20	100.00	661572.36	91979.28
	2003	78.45	68.15	10.30	38132.84	2462.06	7.81	53.59	10.99	79.49	6.21	1.60	79.34	24.37	1.70	99.20	100.00	670993.35	91910.75
	2004	78.82	68.39	10.43	38813.02	2963.51	7.98	53.09	9.96	81.24	6.48	1.50	79.63	24.34	1.75	99.20	100.00	662917.60	91835.22
	2005	79.10	68.59	10.51	39740.90	3176.57	8.24	49.55	9.49	80.85	6.66	1.58	79.92	24.32	1.76	99.20	100.00	659108.09	91764.83
	2006	79.38	68.82	10.56	40418.75	3425.61	8.36	53.63	9.85	81.63	6.83	1.54	80.20	24.41	1.82	99.20	100.00	658331.24	91704.29
	2007	79.64	69.03	10.61	41050.41	3937.19	8.42	51.96	80.73	80.73	6.79	1.62	80.48	24.50	1.86	99.20	100.00	646412.15	91635.04
	2008	79.88	69.23	10.65	40536.13	3863.74	8.85	48.73	8.98	81.58	7.22	1.63	80.76	24.63	1.91	99.20	100.00	635417.17	91572.83
	2009	80.17	69.45	10.72	38545.92	3491.25	9.81	52.74	8.85	83.21	8.16	1.65	81.03	24.85	1.89	99.20	100.00	584940.88	91518.45

	2010	80.39	69.62	10.76	38893.02	3491.22	9.51	58.39	9.61	83.53	7.94	1.57	81.30	25.19	1.92	99.20	100.00	609586.56	91472.82
	2011	80.58	69.78	10.81	39150.76	3648.95	9.34	55.21	9.30	83.15	7.77	1.57	81.57	25.71	1.91	99.20	100.00	568061.83	91457.18
	2012	80.68	69.87	10.82	39455.41	3648.68	9.41	55.38	9.46	82.91	7.80	1.61	81.83	26.33	1.92	99.20	100.00	585779.78	91461.84
	2013	80.77	69.94	10.83	39996.50	3684.75	9.34	57.19	9.55	83.31	7.78	1.56	82.09	27.00	1.83	99.20	100.00	-	91489.39
	2014	80.82	69.98	10.84	40908.75	3934.82	9.12	57.74	9.73	83.14	7.58	1.54	82.35	27.64	1.81	99.20	100.00	-	91553.40
	2015	80.86	70.01	10.84	41536.92	-	-	-	-	-	-	-	82.59	28.19	1.81	99.20	100.00	-	91614.94
	2016	80.90	70.05	10.85	41954.74	-	-	-	-	-	-	-	82.84	28.66	-	-	-	-	91672.80
	2017	80.97	69.30	11.67	-	-	-	-	-	-	-	-	-	-	-	-	-	-	-
United States	1995	75.96	65.63	10.33	38677.72	3788.31	13.09	26.33	14.44	45.16	5.91	7.18	77.26	19.37	1.98	99.60	98.60	6365296.60	88342.26
	1996	76.29	65.90	10.39	39681.52	3944.28	13.04	25.95	14.23	45.16	5.89	7.15	77.64	19.28	1.98	99.70	98.70	6577713.10	88357.64
	1997	76.67	66.20	10.47	40965.85	4116.25	12.96	26.29	14.47	44.98	5.83	7.13	78.01	19.15	1.97	99.70	98.70	6724414.40	88391.37
	1998	76.80	66.30	10.50	42292.89	4302.44	12.99	26.62	14.99	43.71	5.68	7.31	78.38	18.99	2.00	99.70	98.70	6749016.10	88432.26
	1999	76.84	66.33	10.52	43768.88	4521.69	12.99	26.34	14.96	43.22	5.61	7.38	78.74	18.82	2.01	99.70	98.80	6808137.80	88481.83
	2000	76.96	66.39	10.57	45055.82	4788.31	13.07	26.08	14.80	43.26	5.65	7.42	79.06	18.67	2.06	99.70	98.80	6969123.80	88553.19
	2001	77.04	66.45	10.59	45047.49	5140.23	13.73	25.37	14.15	44.24	6.07	7.65	79.23	18.59	2.03	99.80	98.80	6821235.60	88553.77
	2002	77.17	66.54	10.63	45428.65	5575.83	14.55	24.59	13.73	44.15	6.42	8.13	79.41	18.51	2.02	99.80	98.90	6981786.80	88574.09
	2003	77.32	66.65	10.68	46304.04	5995.08	15.06	24.26	13.60	43.95	6.62	8.44	79.58	18.44	2.05	99.80	98.90	6991255.20	88593.66
	2004	77.54	66.80	10.74	47614.28	6369.43	15.14	24.01	13.38	44.25	6.70	8.44	79.76	18.41	2.05	99.80	98.90	7244271.50	88625.36
	2005	77.70	66.89	10.80	48755.62	6741.03	15.15	23.92	13.31	44.36	6.72	8.43	79.93	18.40	2.06	99.80	99.00	7182808.40	88661.42
	2006	77.95	67.08	10.87	49575.40	7122.37	15.27	23.55	12.93	45.07	6.88	8.39	80.10	18.50	2.11	99.90	99.00	6994086.90	88657.74
	2007	78.17	67.23	10.94	49979.53	7511.96	15.57	23.47	45.07	45.07	7.02	8.55	80.27	18.63	2.12	99.90	99.00	7128951.70	88659.09
	2008	78.37	67.37	11.00	49364.64	7786.21	16.02	23.22	12.54	45.98	7.37	8.65	80.44	18.80	2.07	99.90	99.00	6648991.20	88684.16
	2009	78.60	67.53	11.07	47575.61	8023.00	17.00	22.74	12.02	47.15	8.01	8.98	80.61	19.06	2.00	99.90	99.10	6604068.95	88733.39
	2010	78.78	67.65	11.13	48373.88	8269.37	17.02	22.39	11.76	47.48	8.08	8.94	80.77	19.40	1.93	99.90	99.10	6713348.97	88809.49
	2011	78.87	67.72	11.15	48783.47	8523.83	17.06	22.22	11.70	47.34	8.07	8.98	80.94	19.83	1.89	100.00	99.10	6571653.98	88890.65
	2012	78.94	67.77	11.17	49497.59	8789.77	17.02	21.97	11.59	47.26	8.04	8.97	81.11	20.34	1.88	100.00	99.10	6343840.51	88984.00
	2013	78.96	67.78	11.18	49976.63	8987.90	16.90	21.93	11.49	47.61	8.05	8.85	81.28	20.90	1.86	100.00	99.20	-	89077.33
	2014	78.94	67.76	11.18	50782.52	9402.54	17.14	21.37	11.05	48.30	8.28	8.86	81.45	21.50	1.86	100.00	99.20	-	89164.28
	2015	78.87	67.71	11.17	51855.91	-	-	-	-	-	-	-	81.62	22.13	1.84	100.00	99.20	-	89250.07

	2016	78.86	67.69	11.16	52262.78	-	-	-	-	-	-	-	81.79	22.80	-	-	-	-	89337.35
	2017	78.59	66.57	12.03	-	-	-	-	-	-	-	-	-	-	-	-	-	-	-
Uruguay	1995	73.46	64.82	8.64	8044.64	755.83	12.63	23.32	16.16	30.72	3.88	8.75	90.54	19.90	2.40	92.30	95.40	30383.55	89306.26
	1996	73.65	64.99	8.67	8432.62	676.29	10.71	27.71	18.85	31.99	3.42	7.28	90.84	20.20	2.36	92.60	95.70	31694.52	89248.04
	1997	73.90	65.20	8.71	9089.12	615.46	8.40	30.95	21.33	31.10	2.61	5.79	91.15	20.46	2.33	92.80	96.00	31457.97	89224.76
	1998	74.16	65.42	8.73	9438.88	686.76	8.91	27.75	19.69	29.04	2.59	6.32	91.45	20.69	2.29	93.00	96.30	30975.10	89211.66
	1999	74.41	65.64	8.77	9207.79	561.73	7.75	33.18	20.69	37.65	2.92	4.83	91.74	20.87	2.27	93.20	96.50	32266.31	89236.22
	2000	74.82	65.99	8.84	8997.66	537.33	7.82	31.17	20.36	34.68	2.71	5.11	92.03	20.99	2.24	93.50	96.80	30375.78	89281.53
	2001	74.93	66.07	8.86	8636.55	468.52	7.46	30.87	21.29	31.03	2.31	5.14	92.30	21.20	2.22	93.70	97.00	29627.40	89354.60
	2002	75.06	66.17	8.89	7967.16	293.53	7.18	30.64	21.91	28.48	2.04	5.13	92.57	21.35	2.21	93.90	97.30	30061.09	89444.04
	2003	75.03	66.15	8.88	8036.48	235.98	6.52	32.63	23.85	26.91	1.75	4.76	92.83	21.45	2.19	94.10	97.50	30911.30	89544.09
	2004	75.30	66.38	8.93	8442.55	477.10	11.59	32.42	12.01	62.95	7.29	4.29	93.08	21.53	2.17	94.30	97.70	32686.69	89655.91
	2005	75.63	66.66	8.96	9068.24	582.12	11.15	32.08	11.74	63.41	7.07	4.08	93.32	21.59	2.16	94.50	97.90	32688.88	89776.85
	2006	75.84	66.83	9.01	9424.52	656.67	11.17	31.11	10.94	64.85	7.25	3.93	93.55	21.69	2.14	94.70	98.10	34035.50	89846.74
	2007	75.85	66.80	9.05	10014.87	576.77	8.23	29.88	55.82	55.82	4.59	3.63	93.78	21.77	2.13	94.90	98.40	33889.42	89909.10
	2008	76.15	67.01	9.14	10698.05	740.64	8.17	33.77	11.88	64.82	5.30	2.87	94.00	21.83	2.11	95.10	98.60	34052.25	89977.18
	2009	76.39	67.19	9.20	11112.46	827.09	8.78	46.73	18.25	60.93	5.35	3.43	94.21	21.88	2.09	95.30	98.70	33509.68	90026.76
	2010	76.42	67.22	9.20	11938.21	1030.06	8.63	46.92	17.71	62.27	5.37	3.26	94.41	21.94	2.08	95.50	98.90	33285.20	90079.23
	2011	76.49	67.24	9.25	12512.91	1211.82	8.55	48.70	17.27	64.54	5.52	3.03	94.61	22.05	2.06	95.70	99.10	33773.77	90095.49
	2012	76.58	67.31	9.27	12913.10	1322.25	8.74	52.74	16.86	68.04	5.95	2.79	94.80	22.15	2.05	95.90	99.30	34237.83	90108.16
	2013	76.81	67.48	9.32	13467.44	1464.75	8.68	54.90	16.47	70.00	6.07	2.60	94.98	22.26	2.03	96.10	99.50	-	90112.35
	2014	77.08	67.70	9.38	13856.70	1442.28	8.58	54.12	15.58	71.22	6.11	2.47	95.15	22.38	2.02	96.30	99.60	-	90111.58
	2015	77.21	67.80	9.41	13859.41	-	-	-	-	-	-	-	95.31	22.50	2.01	96.40	99.70	-	90122.75
	2016	77.33	67.93	9.39	14010.00	-	-	-	-	-	-	-	95.46	22.65	-	-	-	-	90128.16
	2017	77.05	67.20	9.84	-	-	-	-	-	-	-	-	-	-	-	-	-	-	-
US Virgin	1995	72.63	64.03	8.60	-	-	-	-	-	-	-	-	90.44	11.38	2.48	96.40	100.00	47.59	91166.12
	1996	72.88	64.25	8.64	-	-	-	-	-	-	-	-	90.92	11.66	2.37	96.40	100.00	48.03	91241.98
	1997	72.96	64.30	8.65	-	-	-	-	-	-	-	-	91.37	11.93	2.27	96.40	100.00	48.59	91333.97
	1998	73.08	64.40	8.68	-	-	-	-	-	-	-	-	91.81	12.28	2.18	96.40	100.00	42.02	91442.56

1999	73.19	64.49	8.71	-	-	-	-	-	-	-	-	92.23	12.71	2.13	96.40	100.00	42.62	91535.21
2000	73.35	64.61	8.74	-	-	-	-	-	-	-	-	92.59	13.23	2.06	96.40	100.00	43.27	91656.93
2001	73.49	64.72	8.77	-	-	-	-	-	-	-	-	92.81	13.60	1.90	96.40	100.00	43.89	91786.16
2002	73.59	64.78	8.80	36789.45	-	-	-	-	-	-	-	93.04	14.00	2.19	96.40	100.00	44.53	91891.22
2003	73.62	64.81	8.82	36708.26	-	-	-	-	-	-	-	93.25	14.48	2.05	96.40	100.00	45.14	92019.10
2004	73.67	64.83	8.84	37834.61	-	-	-	-	-	-	-	93.46	15.04	2.15	96.40	100.00	45.72	92144.42
2005	73.78	64.92	8.86	39240.49	-	-	-	-	-	-	-	93.66	15.73	2.23	96.40	100.00	48.06	92277.22
2006	73.83	64.97	8.86	40724.82	-	-	-	-	-	-	-	93.86	16.52	2.02	96.40	100.00	49.57	92369.55
2007	73.87	64.98	8.89	42477.50	-	-	-	-	-	-	-	94.05	17.39	1.97	96.40	100.00	51.75	92482.59
2008	73.94	65.03	8.91	43010.44	-	-	-	-	-	-	-	94.24	18.34	1.91	96.40	100.00	53.84	92602.44
2009	73.95	65.03	8.93	40298.41	-	-	-	-	-	-	-	94.42	19.38	1.86	96.40	100.00	56.64	92726.16
2010	73.93	65.02	8.91	40831.11	-	-	-	-	-	-	-	94.59	20.50	1.81	96.40	100.00	60.21	92860.49
2011	74.08	65.11	8.97	37671.15	-	-	-	-	-	-	-	94.76	21.88	1.80	96.40	100.00	60.86	92942.98
2012	74.15	65.16	8.98	32168.00	-	-	-	-	-	-	-	94.92	23.36	1.78	96.40	100.00	61.49	93007.22
2013	74.23	65.22	9.00	30457.72	-	-	-	-	-	-	-	95.06	24.92	1.77	96.40	100.00	-	93077.19
2014	74.31	65.28	9.03	30328.85	-	-	-	-	-	-	-	95.20	26.48	1.75	96.40	100.00	-	93139.13
2015	74.41	65.36	9.05	30562.65	-	-	-	-	-	-	-	95.34	28.00	1.74	96.40	100.00	-	93187.61
2016	74.62	65.54	9.08	-	-	-	-	-	-	-	-	95.46	29.23	-	-	-	-	93239.99
2017	73.93	65.38	8.55	-	-	-	-	-	-	-	-	-	-	-	-	-	-	-

Table S2 Projected global and regional Life Expectancy, 2017-2025 (95% confidence interval)

	2017	2018	2019	2020	2021	2022	2023	2024	2025
Afghanistan	58.30	58.66	59.01	59.37	59.74	60.10	60.47	60.84	61.21
Albania	77.59	77.80	78.00	78.20	78.40	78.60	78.80	79.00	79.20
Algeria	77.53	77.67	77.81	77.95	78.10	78.24	78.38	78.53	78.67
Andorra	82.52	82.52	82.52	82.52	82.51	82.51	82.51	82.51	82.51
Angola	65.30	65.94	66.57	67.17	67.75	68.31	68.83	69.33	69.79
Antigua and Barbuda	77.45	77.54	77.63	77.71	77.79	77.87	77.94	78.00	78.07
Argentina	76.88	77.05	77.22	77.40	77.57	77.74	77.92	78.09	78.26
Armenia	76.11	76.37	76.63	76.88	77.14	77.40	77.66	77.92	78.18
Australia	82.37	82.19	81.97	81.74	81.47	82.31	82.27	82.24	82.20
Austria	81.66	81.79	81.91	82.04	82.16	82.28	82.41	82.53	82.66
Azerbaijan	72.45	72.87	73.31	73.74	74.19	74.64	75.10	75.56	76.03
Bahamas	73.94	74.03	74.13	74.22	74.31	74.40	74.50	74.59	74.68
Bahrain	77.00	77.23	77.46	77.67	77.88	78.09	78.28	78.47	78.66
Bangladesh	73.02	73.44	73.90	74.38	74.91	75.49	76.11	76.79	77.52
Barbados	76.55	76.49	76.42	76.35	76.28	76.21	76.14	76.08	76.01
Belarus	73.93	74.24	74.54	74.85	75.16	75.46	75.77	76.07	76.38
Belgium	81.05	81.19	81.32	81.45	81.57	81.69	81.80	81.91	82.02
Benin	64.91	65.40	65.90	66.39	66.88	67.37	67.87	68.36	68.85
Bhutan	74.06	74.31	74.57	74.82	75.08	75.33	75.59	75.85	76.10
Bolivia	73.47	73.70	73.93	74.15	74.38	74.60	74.83	75.07	75.32
Bosnia and Herzegovina	77.75	77.95	78.15	78.35	78.55	78.75	78.95	79.15	79.35
Botswana	65.97	66.55	66.96	67.18	67.17	66.93	66.42	65.62	64.51
Brazil	75.35	75.45	75.55	75.65	75.76	75.86	75.96	76.07	76.17
Bulgaria	75.16	75.35	75.55	75.77	75.99	76.22	76.46	76.70	76.94
Burundi	60.71	61.08	61.45	61.81	62.18	62.54	62.91	63.27	63.64

(Continue to next page)

	2017	2018	2019	2020	2021	2022	2023	2024	2025
(Continued from previous page)									
Cameroon	60.65	61.16	61.67	62.19	62.70	63.21	63.72	64.23	64.74
Canada	81.93	82.01	82.08	82.16	82.24	82.31	82.39	82.47	82.54
Chad	60.37	60.95	61.54	62.12	62.70	63.28	63.86	64.44	65.02
Chile	80.47	80.62	80.77	80.92	81.07	81.22	81.37	81.52	81.67
Colombia	78.55	78.84	79.12	79.41	79.69	79.97	80.25	80.53	80.81
China	76.71	77.04	77.38	77.71	78.04	78.36	78.67	78.99	79.29
Comoros	68.00	68.33	68.76	69.11	69.52	69.89	70.29	70.67	71.06
Congogold	64.34	65.01	65.68	66.35	67.02	67.69	68.36	69.03	69.70
Costa Rica	81.08	81.17	81.27	81.36	81.46	81.55	81.65	81.74	81.84
Coted I voire	60.59	61.32	62.04	62.77	63.50	64.22	64.95	65.68	66.40
Croatia	77.37	77.38	77.39	77.40	77.41	77.42	77.43	77.44	77.45
Cuba	79.00	79.08	79.16	79.25	79.33	79.41	79.50	79.58	79.66
Cyprus	80.46	80.44	80.42	80.40	80.38	80.37	80.35	80.33	80.31
Czech Republic	79.25	79.40	79.54	79.69	79.84	79.98	80.13	80.27	80.42
Denmark	81.08	81.34	81.60	81.86	82.13	82.39	82.65	82.92	83.18
Djibouti	67.25	67.98	68.66	69.47	70.26	71.16	72.07	73.07	74.10
Timor-Leste	74.95	75.64	76.34	77.03	77.72	78.41	79.10	79.79	80.49
El Salvador	75.61	75.81	76.00	76.19	76.39	76.58	76.77	76.97	77.16
Equatorial Guinea	65.89	66.30	66.70	67.11	67.52	67.92	68.33	68.74	69.14
Eritrea	64.29	64.67	65.05	65.43	65.81	66.19	66.57	66.94	67.32
Estonia	77.79	77.98	78.15	78.29	78.40	78.50	78.56	78.61	78.62
Ethiopia	65.38	65.39	65.40	65.40	65.41	65.42	65.42	65.43	65.44
Fiji	65.38	65.39	65.40	65.40	65.41	65.42	65.42	65.43	65.44
Finland	82.02	82.31	82.60	82.89	83.19	83.49	83.80	84.11	84.42

France	82.49	82.64	82.78	82.92	83.05	83.18	83.30	83.42	83.53

(Continue to next page)

	2017	2018	2019	2020	2021	2022	2023	2024	2025
(Continued from previous page)									
Gabon	67.19	67.84	68.49	69.15	69.80	70.45	71.10	71.76	72.41
Gambia	67.68	68.04	68.41	68.79	69.18	69.58	69.99	70.41	70.85
Ghana	66.58	67.16	67.73	68.31	68.88	69.46	70.03	70.61	71.18
Greece	81.07	81.17	81.26	81.34	81.42	81.50	81.57	81.63	81.70
Guatemala	72.92	73.08	73.25	73.41	73.58	73.74	73.91	74.07	74.24
Guinea	61.15	61.63	62.12	62.62	63.14	63.66	64.19	64.74	65.29
Guyana	68.54	69.34	70.14	70.94	71.74	72.53	73.33	74.13	74.93
Honduras	73.23	73.48	73.73	73.98	74.23	74.48	74.73	74.99	75.24
Hungary	75.74	75.74	75.74	75.74	75.74	75.74	75.73	75.73	75.73
Iceland	82.30	82.32	82.35	82.38	82.40	82.43	82.46	82.48	82.51
India	68.93	69.36	69.75	70.16	70.52	70.91	71.26	71.62	71.95
Indonesia	71.94	72.22	72.51	72.79	73.07	73.35	73.63	73.92	74.20
Iraq	67.60	67.60	67.60	67.60	67.60	67.60	67.60	67.60	67.60
Ireland	81.17	81.19	81.21	81.23	81.26	81.28	81.30	81.32	81.35
Iran	76.34	76.76	77.19	77.63	78.09	78.56	79.04	79.54	80.05
Israel	82.29	82.44	82.58	82.72	82.84	82.96	83.07	83.17	83.27
Italy	82.45	82.47	82.49	82.51	82.53	82.55	82.57	82.59	82.61
Jamaica	74.96	75.04	75.12	75.22	75.32	75.43	75.54	75.66	75.78
Japan	84.08	84.21	84.33	84.44	84.55	84.66	84.76	84.85	84.93
Jordan	75.88	75.46	75.05	74.63	74.21	73.80	73.38	72.97	72.55
Kazakhstan	71.37	70.47	68.96	66.72	63.68	59.72	54.73	48.59	41.19
Kenya	67.38	67.92	68.46	69.00	69.54	70.08	70.62	71.16	71.70
Kiribati	62.02	62.25	62.49	62.72	62.96	63.19	63.43	63.66	63.89

Kuwait	79.61	79.53	79.45	79.37	79.29	79.21	79.12	79.04	78.96
Kyrgyzstan	71.42	71.61	71.79	71.98	72.16	72.35	72.53	72.72	72.90

(Continue to next page)

	2017	2018	2019	2020	2021	2022	2023	2024	2025
(Continued from previous page)									
Laos	67.73	68.21	68.69	69.18	69.66	70.14	70.62	71.10	71.59
Lebanon	80.19	80.23	80.26	80.30	80.33	80.37	80.40	80.44	80.47
Lesotho	51.11	51.92	52.72	53.53	54.33	55.14	55.94	56.75	57.55
Liberia	64.88	65.53	66.18	66.83	67.48	68.12	68.77	69.42	70.07
Lithuania	75.46	75.75	76.05	76.35	76.65	76.94	77.24	77.54	77.83
Luxembourg	82.20	82.29	82.38	82.46	82.55	82.63	82.72	82.80	82.89
Macedonia	74.97	75.12	75.27	75.41	75.55	75.69	75.83	75.97	76.10
Malawi	60.98	61.79	62.60	63.40	64.21	65.02	65.82	66.63	67.44
Malaysia	75.80	76.04	76.29	76.53	76.77	77.01	77.26	77.50	77.74
Malta	81.51	81.59	81.68	81.76	81.84	81.93	82.01	82.09	82.18
Mauritania	70.55	70.94	71.33	71.72	72.10	72.48	72.87	73.25	73.62
Mexico	76.41	76.42	76.42	76.41	76.39	76.35	76.30	76.23	76.16
Mongolia	68.24	68.36	68.48	68.60	68.73	68.85	68.97	69.09	69.21
Montenegro	77.14	77.25	77.35	77.45	77.56	77.66	77.77	77.87	77.98
Morocco	75.21	75.40	75.59	75.78	75.96	76.13	76.30	76.47	76.63
Mozambique	61.06	62.11	63.15	64.20	65.24	66.28	67.33	68.37	69.41
Namibia	65.41	66.13	66.94	67.82	68.79	69.83	70.96	72.17	73.46
new Zealand	81.98	82.13	82.28	82.44	82.59	82.74	82.90	83.05	83.20
Nicaragua	78.53	78.75	78.96	79.18	79.40	79.61	79.83	80.05	80.26
Niger	62.13	62.59	63.05	63.50	63.96	64.42	64.88	65.33	65.79
Nigeria	65.99	66.97	67.94	68.92	69.89	70.86	71.84	72.81	73.79
Norway	82.11	82.11	82.12	82.13	82.14	82.15	82.15	82.16	82.17

Pakistan	67.97	68.35	68.72	69.10	69.48	69.85	70.23	70.60	70.98
Panama	79.19	79.47	79.74	80.02	80.29	80.57	80.84	81.12	81.39
Papua New Guinea	61.13	61.48	61.83	62.17	62.52	62.87	63.22	63.57	63.91

(Continue to next page)

	2017	2018	2019	2020	2021	2022	2023	2024	2025
(Continued from previous page)									
Paraguay	74.59	74.72	74.85	74.98	75.11	75.23	75.36	75.49	75.62
Peru	79.92	80.10	80.27	80.45	80.63	80.81	80.98	81.16	81.34
Philippines	70.34	70.57	70.80	71.03	71.26	71.49	71.72	71.95	72.18
Poland	78.14	78.34	78.53	78.72	78.89	79.06	79.23	79.38	79.53
Portugal	81.12	81.20	81.27	81.35	81.43	81.50	81.58	81.66	81.74
Qatar	79.78	79.69	79.60	79.51	79.42	79.34	79.25	79.16	79.07
Republic of Congo	61.88	62.63	63.38	64.13	64.88	65.63	66.38	67.13	67.88
Republic of Guinea-Bissau	59.39	60.05	60.74	61.45	62.18	62.94	63.73	64.54	65.37
Republic of Korea	80.93	80.81	80.63	80.40	80.10	79.75	79.33	78.84	78.29
Romania	75.49	75.75	76.00	76.26	76.51	76.76	77.01	77.26	77.50
Rwanda	68.11	68.43	68.74	69.06	69.38	69.70	70.02	70.34	70.66
Saint Lucia	75.28	75.28	75.28	75.28	75.29	75.29	75.29	75.29	75.29
Saint Vincent	71.73	71.82	71.91	72.00	72.10	72.21	72.31	72.42	72.53
Sao Tome and Principe	70.89	71.17	71.46	71.75	72.04	72.33	72.62	72.91	73.20
Saudi Arabia	77.47	77.88	78.28	78.69	79.09	79.50	79.90	80.31	80.72
Senegal	66.64	67.04	67.45	67.85	68.26	68.68	69.09	69.51	69.94
Seychelles	73.80	73.92	74.04	74.16	74.29	74.41	74.53	74.65	74.77
Sierra Leone	57.87	58.27	58.68	59.08	59.49	59.89	60.29	60.70	61.10
Singapore	83.84	83.94	84.04	84.14	84.24	84.34	84.44	84.54	84.64
Slovak Republic	77.20	77.44	77.68	77.92	78.16	78.40	78.64	78.89	79.14
Slovenia	81.14	81.44	81.73	82.03	82.33	82.63	82.93	83.23	83.53

Solomon Islands	63.33	63.64	63.96	64.28	64.60	64.91	65.23	65.55	65.87
South Africa	63.04	63.65	64.25	64.86	65.47	66.07	66.68	67.29	67.89
Spain	83.16	83.34	83.52	83.69	83.86	84.03	84.19	84.35	84.51
Sri Lanka	77.40	77.68	77.95	78.23	78.51	78.78	79.06	79.33	79.61

(Continue to next page)

	2017	2018	2019	2020	2021	2022	2023	2024	2025
(Continued from previous page)									
Sudan	68.60	68.90	69.19	69.47	69.74	70.01	70.27	70.52	70.76
Suriname	71.56	71.78	72.00	72.22	72.44	72.66	72.88	73.10	73.32
Swaziland	58.77	60.11	61.44	62.77	64.11	65.44	66.77	68.10	69.44
Sweden	82.02	82.00	81.97	81.95	81.92	81.90	81.87	81.85	81.82
Switzerland	83.30	83.42	83.55	83.67	83.79	83.92	84.04	84.16	84.29
Tajikistan	72.41	72.83	73.25	73.67	74.10	74.52	74.94	75.36	75.79
Tanzania	65.13	65.97	66.80	67.64	68.48	69.32	70.16	70.99	71.83
Thailand	77.84	77.98	78.12	78.25	78.39	78.53	78.66	78.80	78.94
The Russian Federation	70.89	70.92	70.95	70.98	71.01	71.04	71.07	71.10	71.13
Togo	63.15	63.70	64.25	64.81	65.36	65.92	66.47	67.03	67.58
Tonga	70.39	70.48	70.57	70.66	70.75	70.84	70.94	71.03	71.12
Trinidad and Tobago	73.10	73.18	73.26	73.34	73.42	73.50	73.58	73.66	73.74
Tunisia	77.62	77.81	78.05	78.32	78.65	79.02	79.46	79.96	80.53
Turkey	79.22	79.26	79.26	79.23	79.18	79.09	78.98	78.84	78.66
Turkmenistan	70.58	70.91	71.25	71.58	71.92	72.25	72.58	72.92	73.25
Uganda	62.86	63.52	64.18	64.84	65.50	66.16	66.81	67.47	68.13
Ukraine	72.42	72.74	73.07	73.39	73.72	74.05	74.38	74.71	75.05
United Arab Emirates	75.59	75.66	75.73	75.80	75.87	75.94	76.02	76.09	76.16
United Kingdom	80.94	80.98	81.03	81.07	81.11	81.15	81.19	81.23	81.28
United States	78.84	78.82	78.81	78.79	78.77	78.75	78.74	78.72	78.70

Uruguay	77.46	77.58	77.70	77.82	77.92	78.03	78.13	78.22	78.31
US Virgin Islands	74.68	74.74	74.79	74.84	74.88	74.93	74.97	75.00	75.04
Uzbekistan	70.54	70.78	71.03	71.27	71.52	71.76	72.01	72.25	72.50
Vanuatu	64.04	64.23	64.42	64.61	64.80	64.99	65.18	65.37	65.56
Venezuela	75.87	76.00	76.12	76.25	76.38	76.50	76.63	76.75	76.88

(Continue to next page)

	2017	2018	2019	2020	2021	2022	2023	2024	2025
(Continued from previous page)									
Vietnam	74.79<br>(74.71-74.87)	75.02<br>(74.91-75.14)	75.26<br>(75.12-75.4)	75.50<br>(75.34-75.65)	75.73<br>(75.56-75.91)	75.97<br>(75.77-76.16)	76.20<br>(75.99-76.41)	76.44<br>(76.21-76.66)	76.67<br>(76.44-76.91)
Zambia	59.36<br>(58.76-59.96)	60.09<br>(58.81-61.37)	60.82<br>(58.73-62.92)	61.55<br>(58.52-64.58)	62.28<br>(58.21-66.35)	63.02<br>(57.81-68.22)	63.75<br>(57.31-70.18)	64.48<br>(56.74-72.22)	65.21<br>(56.08-74.34)

Table S3 Projected global and regional Healthy Life Expectancy, 2017-2025 (95% confidence interval)

	2017	2018	2019	2020	2021	2022	2023	2024	2025
Afghanistan	49.79	50.08	50.38	50.68	50.97	51.27	51.56	51.86	52.16
Albania	67.77	67.94	68.1	68.27	68.44	68.61	68.77	68.94	69.11
Algeria	66.14	66.28	66.41	66.54	66.68	66.81	66.95	67.08	67.22
Andorra	71.20	71.20	71.20	71.20	71.21	71.21	71.21	71.21	71.21
Angola	56.92	57.55	58.17	58.8	59.43	60.06	60.68	61.31	61.94
Antigua and Barbuda	67.61	67.68	67.76	67.82	67.89	67.95	68.01	68.07	68.12
Argentina	67.37	67.52	67.66	67.81	67.95	68.1	68.25	68.39	68.54
Armenia	9.43	9.49	9.54	9.60	9.65	9.70	9.76	9.81	9.87
Australia	71.53	71.52	71.51	71.51	71.50	71.49	71.49	71.48	71.47
Austria	71.04	71.14	71.24	71.34	71.44	71.54	71.64	71.74	71.84
Azerbaijan	63.72	64.05	64.38	64.71	65.04	65.38	65.71	66.06	66.40
Bahamas	64.96	65.05	65.14	65.23	65.32	65.41	65.51	65.6	65.69
Bahrain	65.55	65.74	65.91	66.09	66.25	66.42	66.57	66.72	66.86
Bangladesh	63.14	63.51	63.88	64.26	64.63	65	65.37	65.75	66.12
Barbados	66.87	66.83	66.78	66.73	66.69	66.64	66.6	66.55	66.50
Belarus	64.42	64.73	65.03	65.34	65.65	65.96	66.26	66.57	66.88
Belgium	70.25	70.38	70.51	70.63	70.76	70.88	71.01	71.13	71.25
Benin	56.34	56.77	57.19	57.62	58.04	58.47	58.89	59.32	59.74
Bhutan	63.40	63.60	63.79	63.95	64.09	64.22	64.33	64.41	64.48
Bolivia	64.27	64.56	64.86	65.17	65.5	65.84	66.21	66.6	67.01
Bosnia and Herzegovina	67.56	67.82	68.08	68.34	68.6	68.86	69.12	69.38	69.64
Botswana	57.14	57.57	57.86	57.98	57.94	57.69	57.24	56.55	55.61
Brazil	65.61	65.71	65.79	65.85	65.89	65.89	65.87	65.82	65.74
Bulgaria	65.76	65.92	66.07	66.23	66.39	66.55	66.7	66.86	67.01
Burkina Faso	53.35	53.76	54.18	54.59	55.01	55.42	55.84	56.25	56.67

(Continue to next page)

	2017	2018	2019	2020	2021	2022	2023	2024	2025
(Continued from previous page)									
Burundi	53.88	54.25	54.62	54.99	55.36	55.73	56.10	56.47	56.83
Cambodia	60.5	60.89	61.29	61.69	62.09	62.51	62.93	63.35	63.78
Cameroon	52.97	53.45	53.93	54.40	54.88	55.36	55.84	56.32	56.79
Canada	71.25	71.31	71.36	71.41	71.47	71.52	71.57	71.63	71.68
CapeVerde	64.50	64.74	64.97	65.21	65.44	65.68	65.92	66.15	66.39
Chad	52.18	52.68	53.17	53.67	54.16	54.66	55.15	55.65	56.14
Chile	70.10	70.30	70.49	70.69	70.89	71.09	71.29	71.50	71.70
Colombia	69.34	69.58	69.81	70.05	70.29	70.52	70.75	70.98	71.20
China	68.13	68.41	68.68	68.95	69.21	69.46	69.72	69.96	70.21
Comoros	59.68	59.99	60.36	60.69	61.05	61.38	61.74	62.08	62.43
Congo gold	55.75	56.33	56.9	57.48	58.05	58.63	59.20	59.78	60.36
Costa Rica	71.28	71.41	71.53	71.65	71.77	71.89	72.02	72.14	72.26
Coted I voire	52.77	53.38	54.00	54.62	55.24	55.85	56.47	57.09	57.71
Croatia	67.48	67.55	67.62	67.69	67.75	67.82	67.89	67.96	68.03
Cuba	69.29	69.38	69.48	69.58	69.68	69.78	69.87	69.97	70.07
Cyprus	69.77	69.3	68.77	68.19	67.55	66.87	66.13	65.33	64.49
Czech Republic	68.3	68.49	68.68	68.88	69.07	69.26	69.45	69.65	69.84
Denmark	69.93	69.99	70.04	70.09	70.15	70.20	70.25	70.31	70.36
Djibouti	59.19	59.92	60.71	61.62	62.62	63.73	64.96	66.31	67.79
DominicanR epublic	63.99	63.91	63.82	63.71	63.60	63.48	63.35	63.21	63.06
Timor-Leste	65.03	65.62	66.21	66.80	67.39	67.98	68.57	69.16	69.76
El Salvador	66.60	66.72	66.82	66.92	67.01	67.09	67.17	67.23	67.29
Equatorial Guinea	57.23	57.80	58.37	58.94	59.51	60.08	60.65	61.21	61.78
Eritrea	56.70	57.04	57.38	57.72	58.06	58.39	58.73	59.07	59.41

Estonia	67.92	68.29	68.67	69.04	69.41	69.78	70.15	70.52	70.89

(Continue to next page)

	2017	2018	2019	2020	2021	2022	2023	2024	2025
(Continued from previous page)									
Ethiopia	57.21	57.22	57.23	57.24	57.25	57.26	57.26	57.27	57.28
Fiji	57.21	57.22	57.23	57.24	57.25	57.26	57.26	57.27	57.28
Finland	70.76	71.00	71.25	71.51	71.77	72.03	72.30	72.58	72.85
France	71.81	71.92	72.01	72.11	72.19	72.28	72.35	72.43	72.49
Gabon	58.14	58.70	59.26	59.81	60.37	60.93	61.48	62.04	62.60
Gambia	58.58	58.9	59.24	59.58	59.93	60.30	60.67	61.06	61.45
Germany	70.31	70.38	70.45	70.52	70.59	70.67	70.74	70.81	70.88
Ghana	58.44	58.97	59.49	60.02	60.54	61.07	61.59	62.11	62.64
Greece	70.71	70.79	70.87	70.94	71.01	71.07	71.13	71.19	71.25
Guatemala	64.05	64.22	64.39	64.55	64.71	64.86	65.00	65.13	65.26
Guinea	53.11	53.50	53.88	54.26	54.65	55.03	55.41	55.79	56.18
Guyana	59.79	60.41	61.03	61.65	62.28	62.90	63.52	64.14	64.77
Honduras	64.75	64.96	65.18	65.40	65.61	65.83	66.04	66.26	66.48
Hungary	65.91	66.00	66.09	66.18	66.27	66.36	66.45	66.55	66.64
Iceland	71.51	71.54	71.56	71.59	71.62	71.65	71.68	71.70	71.73
India	59.31	59.74	60.20	60.69	61.19	61.72	62.28	62.87	63.48
Indonesia	63.16	63.37	63.58	63.79	64.00	64.20	64.40	64.60	64.80
Iraq	57.43	57.43	57.43	57.43	57.43	57.43	57.43	57.43	57.43
Ireland	70.47	70.49	70.51	70.53	70.55	70.57	70.59	70.61	70.63
Iran	64.99	65.21	65.44	65.66	65.89	66.11	66.34	66.56	66.79
Israel	71.64	71.84	72.05	72.25	72.45	72.66	72.86	73.07	73.28
Italy	71.80	71.80	71.80	71.81	71.81	71.81	71.81	71.81	71.81
Jamaica	65.42	65.51	65.60	65.69	65.79	65.89	66.00	66.12	66.24

Japan	73.25	73.34	73.42	73.50	73.57	73.63	73.69	73.75	73.79
Jordan	64.84	64.57	64.30	64.03	63.77	63.50	63.23	62.96	62.70

(Continue to next page)

	2017	2018	2019	2020	2021	2022	2023	2024	2025
(Continued from previous page)									
Kazakhstan	62.62	62.44	62.13	61.65	61.00	60.18	59.15	57.91	56.46
Kenya	59.03	59.48	59.92	60.37	60.81	61.25	61.70	62.14	62.59
Kiribati	54.21	54.40	54.59	54.78	54.98	55.17	55.36	55.55	55.74
Kuwait	68.24	68.23	68.23	68.23	68.23	68.22	68.22	68.22	68.22
	(67.87-68.61)	(67.41-69.06)	(66.85-69.62)	(66.20-70.26)	(65.48-70.97)	(64.69-71.75)	(63.84-72.60)	(62.93-73.50)	(61.97-74.46)
Kyrgyzstan	62.65	62.84	63.04	63.23	63.42	63.62	63.81	64.01	64.20
	(62.37-62.93)	(62.23-63.45)	(62.03-64.05)	(61.76-64.70)	(61.45-65.40)	(61.08-66.15)	(60.67-66.95)	(60.23-67.79)	(59.74-68.66)
Laos	59.52	59.96	60.40	60.84	61.28	61.72	62.16	62.60	63.04
	(59.36-59.68)	(59.61-60.31)	(59.82-60.98)	(60.00-61.68)	(60.15-62.41)	(60.27-63.17)	(60.36-63.95)	(60.43-64.76)	(60.48-65.59)
Lebanon	68.29	68.33	68.37	68.41	68.45	68.49	68.53	68.57	68.61
	(67.67-68.90)	(67.64-69.01)	(67.55-69.19)	(67.39-69.42)	(67.18-69.71)	(66.93-70.05)	(66.64-70.41)	(66.33-70.81)	(65.99-71.23)
Lesotho	44.45	45.09	45.72	46.35	46.99	47.62	48.26	48.89	49.52
	(43.76-45.15)	(43.54-46.64)	(43.13-48.31)	(42.56-50.15)	(41.85-52.13)	(41.01-54.23)	(40.06-56.46)	(38.99-58.79)	(37.82-61.22)
Liberia	55.67	56.25	56.84	57.42	58.00	58.58	59.16	59.74	60.33
	(53.76-57.58)	(54.33-58.17)	(54.91-58.76)	(55.48-59.35)	(56.05-59.95)	(56.62-60.54)	(57.2-61.13)	(57.77-61.72)	(58.34-62.31)
Lithuania	65.41	65.68	66.01	66.04	66.24	66.44	66.65	66.85	67.05
	(64.74-66.07)	(64.74-66.62)	(64.86-67.17)	(64.71-67.37)	(64.76-67.73)	(64.82-68.07)	(64.89-68.40)	(64.97-68.73)	(65.05-69.04)
Luxembourg	71.04	71.12	71.20	71.28	71.37	71.45	71.53	71.61	71.69
	(70.87-71.21)	(70.76-71.48)	(70.62-71.79)	(70.44-72.13)	(70.24-72.49)	(70.01-72.89)	(69.75-73.30)	(69.48-73.74)	(69.18-74.2)
Macedonia	65.68	65.77	65.86	65.95	66.03	66.10	66.17	66.24	66.31
	(65.49-65.87)	(65.51-66.04)	(65.54-66.19)	(65.57-66.32)	(65.60-66.45)	(65.64-66.56)	(65.68-66.67)	(65.71-66.78)	(65.74-66.87)
Malawi	53.58	54.61	55.82	57.23	58.82	60.63	62.65	64.89	67.37

	(53.27-53.89)	(53.92-55.3)	(54.67-56.98)	(55.54-58.91)	(56.54-61.11)	(57.69-63.57)	(59.00-66.29)	(60.49-69.29)	(62.16-72.57)
Malaysia	66.64	66.87	67.09	67.31	67.53	67.76	67.98	68.20	68.42
	(66.57-66.72)	(66.7-67.03)	(66.81-67.37)	(66.91-67.72)	(66.99-68.08)	(67.05-68.46)	(67.11-68.85)	(67.15-69.25)	(67.18-69.66)
Maldives	69.34	69.55	69.76	69.97	70.18	70.39	70.60	70.81	71.02
	(68.70-69.98)	(68.65-70.45)	(68.53-70.98)	(68.37-71.57)	(68.16-72.19)	(67.92-72.86)	(67.64-73.56)	(67.33-74.28)	(66.99-75.04)
Mali	54.50	54.99	55.49	55.98	56.48	56.97	57.46	57.96	58.45
	(53.69-55.31)	(54.18-55.81)	(54.67-56.31)	(55.16-56.80)	(55.65-57.30)	(56.14-57.80)	(56.63-58.30)	(57.12-58.80)	(57.61-59.30)
Malta	71.00	71.08	71.15	71.23	71.30	71.38	71.45	71.53	71.61
	(70.85-71.16)	(70.72-71.43)	(70.56-71.75)	(70.36-72.10)	(70.13-72.48)	(69.87-72.89)	(69.58-73.33)	(69.27-73.79)	(68.94-74.27)
Mauritania	61.35	61.71	62.06	62.42	62.78	63.14	63.50	63.86	64.22
	(61.15-61.54)	(61.43-61.98)	(61.73-62.40)	(62.03-62.81)	(62.35-63.22)	(62.66-63.62)	(62.98-64.02)	(63.31-64.41)	(63.64-64.81)
Mexico	67.24	67.42	67.60	67.78	67.95	68.13	68.31	68.49	68.67
	(66.95-67.53)	(66.81-68.03)	(66.59-68.60)	(66.33-69.23)	(66.01-69.90)	(65.65-70.62)	(65.24-71.38)	(64.79-72.19)	(64.31-73.02)
Mongolia	60.10	60.29	60.48	60.68	60.87	61.06	61.25	61.44	61.64
	(59.87-60.33)	(59.78-60.8)	(59.63-61.34)	(59.43-61.93)	(59.17-62.56)	(58.88-63.24)	(58.55-63.96)	(58.18-64.71)	(57.78-65.49)
Montenegro	67.24	67.36	67.47	67.59	67.70	67.82	67.93	68.04	68.16
	(67.13-67.35)	(67.11-67.61)	(67.05-67.89)	(66.98-68.20)	(66.87-68.53)	(66.75-68.88)	(66.61-69.25)	(66.45-69.64)	(66.28-70.04)
Morocco	64.17	64.38	64.59	64.80	65.00	65.21	65.42	65.63	65.84
	(64.08-64.26)	(64.25-64.5)	(64.41-64.77)	(64.54-65.05)	(64.67-65.34)	(64.79-65.63)	(64.90-65.94)	(65.00-66.26)	(65.10-66.58)
Mozambique	52.96	53.86	54.79	55.73	56.68	57.65	58.62	59.59	60.56
	(52.47-53.45)	(52.77-54.96)	(52.96-56.62)	(53.05-58.41)	(53.06-60.31)	(52.98-62.31)	(52.83-64.40)	(52.60-66.58)	(52.30-68.82)

(Continue to next page)

	2017	2018	2019	2020	2021	2022	2023	2024	2025
Namibia	57.02	57.62	58.28	59.00	59.79	60.63	61.55	62.54	63.60
	(56.46-57.57)	(56.38-58.86)	(56.21-60.36)	(55.96-62.04)	(55.67-63.90)	(55.34-65.93)	(54.98-68.12)	(54.61-70.47)	(54.23-72.97)
Netherlands	70.71	70.71	70.71	70.71	70.72	70.72	70.72	70.72	70.73
	(70.59-70.83)	(70.44-70.98)	(70.26-71.17)	(70.05-71.38)	(69.81-71.62)	(69.56-71.88)	(69.28-72.16)	(68.99-72.46)	(68.67-72.78)

(Continued from previous page)

new Zealand	70.82	70.84	70.86	70.88	70.90	70.92	70.94	70.96	70.98
	(70.65-70.99)	(70.55-71.13)	(70.43-71.29)	(70.31-71.45)	(70.17-71.63)	(70.02-71.82)	(69.86-72.02)	(69.69-72.23)	(69.51-72.45)
Nicaragua	69.13	69.32	69.51	69.70	69.89	70.08	70.26	70.45	70.64
	(68.09-70.17)	(68.2-70.44)	(68.31-70.70)	(68.43-70.96)	(68.56-71.22)	(68.68-71.47)	(68.81-71.72)	(68.94-71.97)	(69.07-72.21)
Niger	54.44	54.85	55.27	55.69	56.11	56.52	56.94	57.36	57.78
	(54.27-54.60)	(54.55-55.15)	(54.79-55.75)	(55.00-56.38)	(55.18-57.04)	(55.33-57.72)	(55.46-58.42)	(55.58-59.14)	(55.67-59.88)
Nigeria	57.16	58.04	58.92	59.79	60.67	61.55	62.43	63.31	64.19
	(56.81-57.51)	(57.36-58.72)	(57.85-59.99)	(58.28-61.31)	(58.67-62.68)	(59.02-64.09)	(59.32-65.54)	(59.59-67.03)	(59.82-68.55)
Norway	71.51	71.54	71.56	71.58	71.61	71.63	71.65	71.67	71.70
	(71.39-71.64)	(71.27-71.81)	(71.11-72.01)	(70.92-72.24)	(70.71-72.50)	(70.48-72.78)	(70.22-73.08)	(69.95-73.39)	(69.66-73.73)
Pakistan	58.93	59.26	59.58	59.91	60.23	60.56	60.88	61.20	61.53
	(58.28-59.59)	(58.41-60.1)	(58.52-60.65)	(58.60-61.21)	(58.66-61.80)	(58.71-62.40)	(58.73-63.03)	(58.75-63.66)	(58.74-64.31)
Panama	69.64	69.91	70.20	70.51	70.83	71.17	71.52	71.89	72.28
	(69.52-69.76)	(69.65-70.18)	(69.76-70.65)	(69.86-71.16)	(69.95-71.71)	(70.04-72.30)	(70.12-72.93)	(70.20-73.59)	(70.28-74.29)
Papua New Guinea	53.40	53.69	53.98	54.26	54.55	54.83	55.12	55.41	55.69
	(52.93-53.88)	(53.06-54.32)	(53.17-54.78)	(53.26-55.26)	(53.34-55.76)	(53.40-56.27)	(53.45-56.79)	(53.48-57.33)	(53.50-57.88)
Paraguay	65.06	65.16	65.27	65.38	65.49	65.59	65.70	65.81	65.92
	(64.90-65.21)	(64.92-65.41)	(64.92-65.62)	(64.91-65.85)	(64.89-66.08)	(64.86-66.33)	(64.82-66.58)	(64.77-66.85)	(64.72-67.12)
Peru	70.10	70.35	70.61	70.86	71.12	71.37	71.63	71.88	72.14
	(69.86-70.34)	(69.84-70.87)	(69.76-71.46)	(69.63-72.10)	(69.45-72.78)	(69.24-73.51)	(68.99-74.27)	(68.70-75.06)	(68.39-75.89)
Philippines	61.86	62.06	62.27	62.48	62.68	62.89	63.10	63.30	63.51
	(61.66-62.05)	(61.73-62.39)	(61.77-62.76)	(61.80-63.15)	(61.81-63.56)	(61.80-63.98)	(61.77-64.42)	(61.73-64.87)	(61.68-65.34)
Poland	67.78	67.96	68.14	68.31	68.48	68.65	68.81	68.96	69.12
	(67.48-68.08)	(67.54-68.38)	(67.62-68.65)	(67.72-68.90)	(67.82-69.14)	(67.92-69.37)	(68.02-69.59)	(68.13-69.80)	(68.23-70.01)
Portugal	70.63	70.72	70.82	70.92	71.01	71.11	71.20	71.30	71.40
	(70.50-70.76)	(70.53-70.92)	(70.54-71.10)	(70.52-71.31)	(70.50-71.53)	(70.46-71.76)	(70.41-72.00)	(70.34-72.26)	(70.27-72.52)
Qatar	68.14	68.13	68.12	68.11	68.09	68.08	68.07	68.06	68.05

	(67.95-68.33)	(67.71-68.54)	(67.42-68.81)	(67.09-69.13)	(66.71-69.48)	(66.31-69.86)	(65.87-70.28)	(65.40-70.72)	(64.90-71.19)
Republic of Congo	53.50	54.18	54.87	55.56	56.25	56.94	57.62	58.31	59.00
	(52.61-54.38)	(53.26-55.11)	(53.86-55.88)	(54.41-56.71)	(54.91-57.59)	(55.37-58.50)	(55.79-59.46)	(56.18-60.45)	(56.54-61.46)
Republic of Guinea-Bissau	51.94	52.52	53.12	53.73	54.37	55.03	55.71	56.41	57.13
	(51.35-52.53)	(51.68-53.35)	(52.09-54.14)	(52.55-54.92)	(53.05-55.69)	(53.58-56.48)	(54.15-57.27)	(54.74-58.08)	(55.35-58.90)
Republic of Korea	70.84	70.93	71.01	71.09	71.17	71.25	71.33	71.41	71.50
	(70.69-71.00)	(70.58-71.27)	(70.43-71.58)	(70.25-71.92)	(70.04-72.30)	(69.80-72.70)	(69.54-73.13)	(69.25-73.58)	(68.94-74.06)
Romania	65.91	66.17	66.37	66.53	66.76	67.06	67.38	67.63	67.83
	(65.64-66.17)	(65.74-66.6)	(65.87-66.87)	(66.03-67.04)	(66.26-67.27)	(66.55-67.58)	(66.80-67.95)	(66.99-68.28)	(67.16-68.50)
Rwanda	59.79	60.14	60.49	60.84	61.19	61.54	61.89	62.25	62.60
	(58.86-60.72)	(58.69-61.59)	(58.28-62.7)	(57.71-63.97)	(57.01-65.38)	(56.19-66.9)	(55.28-68.51)	(54.28-70.22)	(53.19-72.01)
Saint Lucia	66.45	66.51	66.56	66.61	66.66	66.70	66.73	66.76	66.78
	(66.17-66.73)	(66.11-66.9)	(66.08-67.05)	(66.06-67.17)	(66.03-67.29)	(66.01-67.38)	(65.99-67.47)	(65.97-67.55)	(65.94-67.62)

(Continue to next page)

	2017	2018	2019	2020	2021	2022	2023	2024	2025
(Continued from previous page)									
Saint Vincent	71.73	71.82	71.91	72.00	72.10	72.21	72.31	72.42	72.53
	(71.34-72.11)	(71.27-72.36)	(71.24-72.58)	(71.23-72.78)	(71.24-72.97)	(71.26-73.15)	(71.29-73.33)	(71.33-73.51)	(71.38-73.69)
Sao Tome and Principe	61.91	62.18	62.44	62.71	62.97	63.24	63.50	63.77	64.03
	(61.56-62.27)	(61.57-62.78)	(61.55-63.34)	(61.49-63.92)	(61.40-64.54)	(61.28-65.19)	(61.13-65.87)	(60.96-66.57)	(60.77-67.29)
Saudi Arabia	66.68	67.01	67.34	67.67	68.00	68.33	68.66	69.00	69.33
	(66.47-66.88)	(66.68-67.33)	(66.88-67.80)	(67.06-68.28)	(67.23-68.78)	(67.38-69.28)	(67.52-69.81)	(67.65-70.34)	(67.77-70.88)
Senegal	58.30	58.72	59.15	59.58	60.02	60.47	60.92	61.39	61.86
	(58.09-58.52)	(58.42-59.03)	(58.77-59.52)	(59.15-60.02)	(59.54-60.51)	(59.94-61.00)	(60.35-61.50)	(60.77-62.00)	(61.21-62.51)
Seychelles	65.08	65.20	65.33	65.46	65.59	65.72	65.84	65.97	66.10
	(64.95-65.20)	(64.94-65.47)	(64.90-65.77)	(64.83-66.09)	(64.74-66.43)	(64.63-66.80)	(64.50-67.18)	(64.36-67.58)	(64.20-68.00)

Sierra Leone	50.55 (48.54-52.57)	50.93 (48.9-52.95)	51.30 (49.27-53.34)	51.68 (49.64-53.72)	52.06 (50.00-54.11)	52.43 (50.37-54.50)	52.81 (50.74-54.88)	53.18 (51.10-55.27)	53.56 (51.47-55.65)
Singapore	73.74 (73.59-73.90)	73.86 (73.58-74.14)	73.98 (73.56-74.40)	74.10 (73.51-74.69)	74.22 (73.44-74.99)	74.33 (73.35-75.31)	74.45 (73.26-75.65)	74.57 (73.14-76.00)	74.69 (73.02-76.36)
Slovak Republic	67.07 (66.88-67.27)	67.27 (66.99-67.55)	67.46 (67.12-67.80)	67.66 (67.26-68.05)	67.86 (67.41-68.30)	68.05 (67.57-68.54)	68.25 (67.73-68.78)	68.46 (67.90-69.02)	68.66 (68.07-69.25)
Slovenia	69.56 (69.36-69.75)	69.79 (69.52-70.06)	70.02 (69.69-70.36)	70.26 (69.87-70.65)	70.49 (70.06-70.93)	70.73 (70.25-71.21)	70.97 (70.45-71.49)	71.21 (70.65-71.76)	71.44 (70.85-72.04)
Solomon Islands	55.54 (55.30-55.77)	55.8 (55.40-56.20)	56.06 (55.43-56.69)	56.32 (55.41-57.23)	56.58 (55.36-57.80)	56.84 (55.28-58.41)	57.1 (55.17-59.04)	57.37 (55.03-59.70)	57.63 (54.87-60.38)
South Africa	54.31 (53.57-55.04)	54.78 (53.13-56.43)	55.26 (52.49-58.02)	55.73 (51.69-59.78)	56.21 (50.73-61.69)	56.69 (49.64-63.73)	57.16 (48.42-65.90)	57.64 (47.09-68.19)	58.11 (45.65-70.58)
Spain	83.16 (82.99-83.32)	83.34 (83.10-83.58)	83.52 (83.22-83.81)	83.69 (83.35-84.03)	83.86 (83.48-84.24)	84.03 (83.61-84.44)	84.19 (83.74-84.64)	84.35 (83.87-84.83)	84.51 (84.00-85.01)
Sri Lanka	67.56 (65.60-69.52)	67.79 (65.82-69.77)	68.03 (66.04-70.01)	68.26 (66.27-70.25)	68.49 (66.49-70.49)	68.72 (66.71-70.73)	68.96 (66.94-70.98)	69.19 (67.16-71.22)	69.42 (67.38-71.46)
Sudan	58.43 (58.07-58.78)	58.67 (58.17-59.17)	58.91 (58.30-59.53)	59.14 (58.44-59.86)	59.37 (58.58-60.17)	59.59 (58.72-60.47)	59.80 (58.86-60.75)	60.00 (58.99-61.02)	60.20 (59.13-61.28)
Suriname	62.43 (62.27-62.60)	62.62 (62.26-62.99)	62.81 (62.20-63.42)	63.00 (62.10-63.90)	63.19 (61.97-64.40)	63.38 (61.82-64.94)	63.57 (61.63-65.50)	63.75 (61.42-66.09)	63.94 (61.18-66.71)
Swaziland	50.60 (49.83-51.38)	51.53 (49.79-53.27)	52.37 (49.46-55.28)	53.13 (48.87-57.38)	53.76 (48.00-59.53)	54.26 (46.85-61.68)	54.61 (45.41-63.81)	54.78 (43.68-65.89)	54.76 (41.63-67.89)
Sweden	70.92 (70.83-71.01)	70.95 (70.74-71.15)	70.92 (70.63-71.21)	70.89 (70.52-71.27)	70.90 (70.41-71.38)	70.90 (70.28-71.52)	70.87 (70.12-71.62)	70.85 (69.97-71.73)	70.84 (69.81-71.87)
Switzerland	72.10 (71.95-72.25)	72.27 (72.06-72.47)	72.43 (72.17-72.68)	72.59 (72.3-72.88)	72.75 (72.42-73.08)	72.90 (72.55-73.26)	73.06 (72.67-73.45)	73.21 (72.80-73.62)	73.36 (72.92-73.8)
Tajikistan	63.70	64.07	64.43	64.79	65.16	65.52	65.89	66.25	66.61

	(63.21-64.20)	(63.57-64.57)	(63.93-64.93)	(64.29-65.30)	(64.65-65.67)	(65.01-66.03)	(65.37-66.40)	(65.73-66.77)	(66.09-67.13)
Tanzania	57.26	57.96	58.64	59.28	59.89	60.45	60.96	61.42	61.82
	(56.95-57.56)	(57.27-58.64)	(57.49-59.78)	(57.60-60.96)	(57.62-62.16)	(57.53-63.37)	(57.34-64.59)	(57.05-65.80)	(56.65-66.99)
Thailand	68.22	68.51	68.79	69.08	69.37	69.66	69.96	70.25	70.55
	(67.98-68.46)	(68.17-68.84)	(68.38-69.20)	(68.61-69.55)	(68.84-69.90)	(69.09-70.24)	(69.33-70.58)	(69.58-70.92)	(69.84-71.25)
The Russian Federation	62.06	62.21	62.36	62.51	62.65	62.80	62.95	63.10	63.25
	(61.11-63.01)	(60.13-64.29)	(58.90-65.81)	(57.47-67.54)	(55.86-69.45)	(54.08-71.52)	(52.15-73.75)	(50.07-76.13)	(47.87-78.63)

(Continue to next page)

	2017	2018	2019	2020	2021	2022	2023	2024	2025
(Continued from previous page)									
Togo	55.37	55.87	56.36	56.86	57.35	57.85	58.35	58.84	59.34
	(54.92-55.82)	(55.05-56.68)	(55.13-57.59)	(55.15-58.57)	(55.12-59.59)	(55.05-60.65)	(54.94-61.75)	(54.79-62.90)	(54.6-64.08)
Tonga	61.22	61.28	61.35	61.41	61.48	61.54	61.61	61.67	61.74
	(60.51-61.92)	(60.58-61.98)	(60.64-62.05)	(60.71-62.11)	(60.77-62.18)	(60.84-62.24)	(60.90-62.31)	(60.97-62.37)	(61.04-62.44)
Trinidad and Tobago	63.59	63.66	63.74	63.81	63.89	63.97	64.04	64.12	64.19
	(63.35-63.82)	(63.21-64.12)	(63.02-64.46)	(62.80-64.83)	(62.55-65.23)	(62.27-65.66)	(61.97-66.11)	(61.64-66.60)	(61.28-67.1)
Tunisia	66.83	67.09	67.41	67.77	68.20	68.68	69.24	69.88	70.59
	(66.70-66.96)	(66.81-67.38)	(66.93-67.88)	(67.07-68.47)	(67.25-69.14)	(67.47-69.90)	(67.73-70.75)	(68.06-71.70)	(68.44-72.75)
Turkey	67.67	67.72	67.74	67.74	67.72	67.67	67.6	67.51	67.39
	(66.75-68.59)	(66.41-69.02)	(66.15-69.34)	(65.90-69.59)	(65.66-69.78)	(65.41-69.93)	(65.16-70.04)	(64.90-70.12)	(64.62-70.16)
Turkmenistan	62.37	62.69	63.00	63.31	63.63	63.94	64.25	64.57	64.88
	(62.25-62.49)	(62.42-62.96)	(62.55-63.45)	(62.65-63.98)	(62.73-64.53)	(62.78-65.10)	(62.82-65.69)	(62.83-66.30)	(62.83-66.93)
Uganda	54.93	55.52	56.1	56.68	57.27	57.85	58.44	59.02	59.60
	(54.64-55.23)	(54.90-56.13)	(55.10-57.10)	(55.24-58.13)	(55.34-59.20)	(55.39-60.32)	(55.39-61.48)	(55.36-62.68)	(55.29-63.92)
Ukraine	63.32	63.62	63.92	64.22	64.53	64.84	65.16	65.48	65.80
	(62.52-64.13)	(62.48-64.75)	(62.52-65.31)	(62.61-65.83)	(62.73-66.33)	(62.87-66.81)	(63.03-67.29)	(63.20-67.75)	(63.39-68.22)

United Arab Emirates	65.21 (65.03-65.39)	65.33 (64.93-65.74)	65.46 (64.78-66.13)	65.58 (64.60-66.57)	65.71 (64.37-67.05)	65.84 (64.12-67.56)	65.96 (63.83-68.10)	66.09 (63.51-68.66)	66.21 (63.17-69.26)
United Kingdom	70.08 (70.00-70.17)	70.12 (69.94-70.30)	70.15 (69.86-70.45)	70.19 (69.76-70.62)	70.23 (69.64-70.81)	70.26 (69.51-71.01)	70.30 (69.37-71.22)	70.33 (69.21-71.45)	70.37 (69.05-71.69)
United States	67.68 (67.56-67.80)	67.66 (67.39-67.93)	67.65 (67.20-68.10)	67.63 (66.97-68.29)	67.62 (66.72-68.51)	67.60 (66.45-68.75)	67.58 (66.16-69.01)	67.57 (65.85-69.29)	67.55 (65.52-69.59)
Uruguay	68.03 (67.81-68.25)	68.12 (67.81-68.43)	68.21 (67.83-68.59)	68.29 (67.85-68.73)	68.37 (67.88-68.86)	68.44 (67.91-68.98)	68.51 (67.93-69.09)	68.58 (67.96-69.20)	68.64 (67.98-69.29)
US Virgin Islands	65.58 (65.47-65.70)	65.62 (65.45-65.78)	65.65 (65.45-65.85)	65.68 (65.45-65.91)	65.71 (65.45-65.97)	65.73 (65.45-66.02)	65.75 (65.44-66.06)	65.77 (65.44-66.10)	65.78 (65.43-66.13)
Uzbekistan	62.05 (61.90-62.20)	62.30 (61.96-62.63)	62.55 (61.98-63.11)	62.79 (61.97-63.62)	63.04 (61.93-64.15)	63.29 (61.85-64.72)	63.53 (61.76-65.31)	63.78 (61.63-65.93)	64.03 (61.49-66.57)
Vanuatu	56.05 (55.52-56.58)	56.19 (55.65-56.74)	56.34 (55.78-56.91)	56.49 (55.88-57.10)	56.64 (55.97-57.31)	56.79 (56.04-57.53)	56.93 (56.09-57.78)	57.08 (56.13-58.04)	57.23 (56.15-58.31)
Venezuela	66.90 (65.49-68.31)	67.00 (65.59-68.41)	67.10 (65.68-68.52)	67.20 (65.78-68.63)	67.30 (65.87-68.73)	67.40 (65.97-68.84)	67.50 (66.06-68.95)	67.61 (66.16-69.06)	67.71 (66.25-69.16)
Vietnam	66.13 (66.06-66.20)	66.35 (66.25-66.44)	66.56 (66.45-66.68)	66.78 (66.64-66.92)	67.00 (66.85-67.15)	67.22 (67.05-67.39)	67.43 (67.25-67.62)	67.65 (67.46-67.85)	67.87 (67.66-68.08)
Zambia	51.96 (51.46-52.45)	52.36 (51.25-53.47)	52.66 (50.80-54.52)	52.85 (50.12-55.57)	52.91 (49.22-56.59)	52.83 (48.09-57.57)	52.60 (46.72-58.48)	52.20 (45.11-59.30)	51.64 (43.25-60.03)
Zimbabwe	52.89 (52.15-53.62)	53.69 (52.05-55.33)	54.50 (51.76-57.24)	55.31 (51.29-59.32)	56.11 (50.68-61.55)	56.92 (49.93-63.91)	57.73 (49.06-66.40)	58.53 (48.07-69.00)	59.34 (46.97-71.71)

Table S4 Projected global and regional GAP, 2017-2025 (95% confidence interval)

	2017	2018	2019	2020	2021	2022	2023	2024	2025
Afghanistan	8.49<br>(8.30-8.68)	8.52<br>(8.26-8.79)	8.55<br>(8.23-8.88)	8.59<br>(8.21-8.96)	8.62<br>(8.20-9.04)	8.65<br>(8.19-9.11)	8.68<br>(8.19-9.18)	8.72<br>(8.19-9.25)	8.75<br>(8.19-9.31)
Albania	9.93<br>(9.71-10.15)	9.98<br>(9.71-10.24)	10.02<br>(9.71-10.34)	10.07<br>(9.70-10.44)	10.12<br>(9.68-10.55)	10.16<br>(9.66-10.66)	10.21<br>(9.64-10.78)	10.25<br>(9.61-10.89)	10.30<br>(9.58-11.02)
Algeria	11.36<br>(11.30-11.42)	11.37<br>(11.29-11.45)	11.37<br>(11.25-11.48)	11.37<br>(11.21-11.53)	11.37<br>(11.16-11.58)	11.37<br>(11.11-11.64)	11.37<br>(11.05-11.70)	11.38<br>(10.98-11.77)	11.38<br>(10.91-11.84)
Andorra	11.32<br>(11.28-11.37)	11.32<br>(11.22-11.41)	11.31<br>(11.16-11.47)	11.31<br>(11.09-11.53)	11.30<br>(11.01-11.60)	11.30<br>(10.92-11.67)	11.29<br>(10.83-11.76)	11.29<br>(10.73-11.85)	11.28<br>(10.63-11.94)
Angola	8.42<br>(8.35-8.50)	8.50<br>(8.37-8.63)	8.57<br>(8.38-8.76)	8.64<br>(8.39-8.90)	8.72<br>(8.38-9.05)	8.79<br>(8.38-9.20)	8.86<br>(8.36-9.36)	8.93<br>(8.35-9.52)	9.01<br>(8.32-9.69)
Antigua and Barbuda	9.84<br>(9.75-9.93)	9.85<br>(9.73-9.98)	9.87<br>(9.72-10.01)	9.88<br>(9.70-10.05)	9.88<br>(9.69-10.08)	9.89<br>(9.68-10.10)	9.90<br>(9.67-10.13)	9.90<br>(9.66-10.15)	9.91<br>(9.65-10.17)
Argentina	9.54<br>(9.51-9.57)	9.57<br>(9.54-9.60)	9.60<br>(9.56-9.63)	9.63<br>(9.59-9.66)	9.65<br>(9.62-9.69)	9.68<br>(9.65-9.72)	9.71<br>(9.68-9.75)	9.74<br>(9.70-9.77)	9.77<br>(9.73-9.80)
Armenia	9.43<br>(9.28-9.59)	9.49<br>(9.28-9.70)	9.54<br>(9.28-9.81)	9.60<br>(9.29-9.91)	9.65<br>(9.31-10.00)	9.70<br>(9.33-10.09)	9.76<br>(9.35-10.18)	9.81<br>(9.38-10.26)	9.87<br>(9.41-10.35)
Australia	10.96<br>(10.91-11.00)	10.93<br>(10.83-11.02)	10.90<br>(10.74-11.06)	10.87<br>(10.64-11.10)	10.84<br>(10.54-11.15)	10.82<br>(10.42-11.21)	10.79<br>(10.30-11.28)	10.76<br>(10.17-11.35)	10.73<br>(10.03-11.43)
Austria	10.63<br>(10.60-10.67)	10.66<br>(10.61-10.71)	10.69<br>(10.63-10.75)	10.72<br>(10.65-10.78)	10.74<br>(10.67-10.82)	10.77<br>(10.68-10.85)	10.79<br>(10.70-10.88)	10.81<br>(10.72-10.91)	10.83<br>(10.73-10.93)
Azerbaijan	8.70<br>(8.58-8.82)	8.75<br>(8.55-8.96)	8.81<br>(8.52-9.11)	8.87<br>(8.47-9.27)	8.92<br>(8.41-9.44)	8.98<br>(8.34-9.62)	9.04<br>(8.27-9.81)	9.10<br>(8.19-10.01)	9.15<br>(8.09-10.21)

Bahamas	8.97 (8.90-9.04)	8.96 (8.84-9.09)	8.96 (8.76-9.15)	8.95 (8.67-9.23)	8.94 (8.57-9.31)	8.93 (8.47-9.40)	8.92 (8.36-9.49)	8.92 (8.24-9.59)	8.91 (8.11-9.70)
Bahrain	11.43 (11.31-11.54)	11.44 (11.27-11.61)	11.45 (11.24-11.65)	11.45 (11.22-11.69)	11.45 (11.19-11.72)	11.45 (11.16-11.74)	11.45 (11.13-11.76)	11.44 (11.10-11.77)	11.42 (11.07-11.77)
Bangladesh	9.90 (9.81-9.99)	9.93 (9.82-10.03)	9.95 (9.82-10.08)	9.97 (9.81-10.14)	9.99 (9.79-10.20)	10.01 (9.76-10.26)	10.03 (9.73-10.33)	10.05 (9.69-10.4)	10.06 (9.66-10.47)
Barbados	9.67 (9.61-9.74)	9.64 (9.51-9.77)	9.61 (9.41-9.81)	9.58 (9.30-9.86)	9.55 (9.18-9.92)	9.52 (9.05-9.98)	9.49 (8.92-10.06)	9.46 (8.77-10.14)	9.43 (8.62-10.23)
Belarus	9.49 (9.31-9.67)	9.47 (9.12-9.82)	9.45 (8.89-10.00)	9.43 (8.63-10.22)	9.40 (8.35-10.46)	9.38 (8.05-10.72)	9.36 (7.72-11.00)	9.34 (7.37-11.30)	9.32 (7.01-11.63)
Belgium	10.79 (10.73-10.86)	10.79 (10.64-10.93)	10.78 (10.54-11.02)	10.77 (10.42-11.12)	10.76 (10.29-11.23)	10.75 (10.16-11.35)	10.75 (10.01-11.49)	10.74 (9.85-11.63)	10.73 (9.68-11.79)
Benin	8.52 (8.44-8.59)	8.56 (8.46-8.66)	8.60 (8.46-8.75)	8.65 (8.46-8.83)	8.69 (8.46-8.92)	8.74 (8.46-9.01)	8.78 (8.45-9.11)	8.82 (8.44-9.21)	8.87 (8.43-9.31)
Bhutan	10.77 (10.63-10.91)	10.80 (10.66-10.94)	10.83 (10.69-10.98)	10.87 (10.71-11.02)	10.90 (10.73-11.06)	10.92 (10.75-11.10)	10.95 (10.76-11.14)	10.98 (10.77-11.19)	11.01 (10.78-11.24)
Bolivia	9.26 (9.21-9.31)	9.25 (9.17-9.33)	9.24 (9.12-9.37)	9.23 (9.06-9.41)	9.23 (8.99-9.46)	9.22 (8.92-9.52)	9.21 (8.84-9.58)	9.20 (8.75-9.65)	9.19 (8.66-9.72)
Bosnia and Herzegovina	10.34 (10.03-10.65)	10.42 (9.98-10.87)	10.51 (9.97-11.05)	10.60 (9.97-11.22)	10.68 (9.98-11.38)	10.77 (10.00-11.53)	10.85 (10.02-11.68)	10.94 (10.05-11.82)	11.02 (10.08-11.96)
Botswana	8.81 (8.58-9.04)	8.99 (8.47-9.51)	9.16 (8.30-10.03)	9.34 (8.07-10.61)	9.52 (7.80-11.24)	9.70 (7.48-11.91)	9.87 (7.12-12.62)	10.05 (6.73-13.37)	10.23 (6.31-14.15)

(Continue to next page)

	2017	2018	2019	2020	2021	2022	2023	2024	2025
(Continued from previous page)									
Brazil	9.74 (9.68-9.80)	9.71 (9.60-9.83)	9.69 (9.51-9.87)	9.66 (9.41-9.92)	9.64 (9.29-9.98)	9.61 (9.18-10.04)	9.58 (9.05-10.12)	9.56 (8.92-10.20)	9.53 (8.78-10.28)

Bulgaria	9.39<br>(9.29-9.49)	9.35<br>(9.14-9.56)	9.31<br>(8.97-9.65)	9.27<br>(8.79-9.75)	9.23<br>(8.58-9.88)	9.19<br>(8.37-10.01)	9.15<br>(8.13-10.17)	9.11<br>(7.89-10.33)	9.07<br>(7.63-10.51)
Burkina Faso	7.91<br>(7.80-8.02)	7.95<br>(7.79-8.11)	8.00<br>(7.81-8.19)	8.04<br>(7.82-8.26)	8.09<br>(7.85-8.34)	8.14<br>(7.87-8.41)	8.19<br>(7.90-8.48)	8.25<br>(7.94-8.56)	8.30<br>(7.97-8.63)
Burundi	6.86<br>(6.76-6.96)	6.87<br>(6.72-7.02)	6.88<br>(6.65-7.11)	6.90<br>(6.57-7.22)	6.91<br>(6.47-7.35)	6.92<br>(6.36-7.48)	6.93<br>(6.24-7.62)	6.94<br>(6.11-7.78)	6.96<br>(5.97-7.94)
Cambodia	8.63<br>(8.56-8.70)	8.62<br>(8.50-8.74)	8.62<br>(8.44-8.80)	8.61<br>(8.36-8.86)	8.61<br>(8.28-8.93)	8.60<br>(8.19-9.01)	8.59<br>(8.09-9.09)	8.59<br>(7.99-9.18)	8.58<br>(7.89-9.27)
Cameroon	7.68<br>(7.63-7.74)	7.71<br>(7.59-7.84)	7.75<br>(7.54-7.95)	7.78<br>(7.48-8.08)	7.81<br>(7.41-8.22)	7.84<br>(7.33-8.36)	7.88<br>(7.23-8.52)	7.91<br>(7.13-8.69)	7.94<br>(7.03-8.86)
Canada	10.67<br>(10.62-10.72)	10.69<br>(10.59-10.79)	10.71<br>(10.55-10.87)	10.73<br>(10.50-10.95)	10.74<br>(10.44-11.05)	10.76<br>(10.37-11.15)	10.78<br>(10.30-11.26)	10.80<br>(10.22-11.37)	10.81<br>(10.13-11.50)
CapeVerde	9.40<br>(9.32-9.48)	9.39<br>(9.27-9.51)	9.37<br>(9.23-9.52)	9.36<br>(9.19-9.53)	9.34<br>(9.15-9.53)	9.31<br>(9.11-9.52)	9.29<br>(9.06-9.51)	9.26<br>(9.02-9.50)	9.22<br>(8.97-9.48)
Chad	8.19<br>(8.10-8.28)	8.27<br>(8.15-8.40)	8.36<br>(8.17-8.54)	8.44<br>(8.19-8.70)	8.53<br>(8.19-8.87)	8.61<br>(8.18-9.05)	8.70<br>(8.16-9.23)	8.78<br>(8.14-9.43)	8.87<br>(8.10-9.63)
Chile	10.37<br>(10.27-10.47)	10.32<br>(10.12-10.51)	10.27<br>(9.95-10.58)	10.21<br>(9.77-10.66)	10.16<br>(9.58-10.75)	10.11<br>(9.37-10.86)	10.06<br>(9.14-10.98)	10.01<br>(8.91-11.11)	9.96<br>(8.67-11.25)
Colombia	9.26<br>(9.17-9.35)	9.31<br>(9.21-9.40)	9.35<br>(9.26-9.45)	9.40<br>(9.31-9.50)	9.45<br>(9.35-9.54)	9.50<br>(9.40-9.59)	9.54<br>(9.45-9.64)	9.59<br>(9.49-9.69)	9.64<br>(9.54-9.73)
China	8.55<br>(8.46-8.64)	8.58<br>(8.44-8.72)	8.62<br>(8.41-8.82)	8.65<br>(8.38-8.93)	8.69<br>(8.34-9.04)	8.72<br>(8.29-9.15)	8.76<br>(8.24-9.27)	8.79<br>(8.18-9.40)	8.82<br>(8.11-9.53)
Comoros	8.30<br>(8.16-8.45)	8.34<br>(8.17-8.52)	8.38<br>(8.19-8.57)	8.42<br>(8.21-8.63)	8.46<br>(8.23-8.69)	8.50<br>(8.25-8.74)	8.53<br>(8.27-8.80)	8.57<br>(8.30-8.85)	8.61<br>(8.32-8.90)
Congogold	8.59<br>(8.04-9.14)	8.69<br>(8.13-9.24)	8.78<br>(8.22-9.34)	8.87<br>(8.31-9.43)	8.97<br>(8.41-9.53)	9.06<br>(8.50-9.63)	9.16<br>(8.59-9.72)	9.25<br>(8.68-9.82)	9.34<br>(8.77-9.91)
Costa Rica	9.81	9.81	9.80	9.79	9.78	9.77	9.76	9.76	9.75

	(9.76-9.87)	(9.71-9.90)	(9.66-9.94)	(9.59-9.99)	(9.52-10.04)	(9.45-10.10)	(9.36-10.16)	(9.28-10.23)	(9.19-10.31)
Coted I voire	7.81	7.91	8.00	8.10	8.20	8.30	8.40	8.49	8.59
	(7.69-7.93)	(7.71-8.11)	(7.71-8.30)	(7.70-8.51)	(7.68-8.72)	(7.65-8.95)	(7.61-9.18)	(7.56-9.43)	(7.50-9.68)
Croatia	9.97	9.99	10.02	10.04	10.05	10.07	10.08	10.09	10.10
	(9.90-10.04)	(9.89-10.10)	(9.89-10.14)	(9.89-10.18)	(9.89-10.22)	(9.89-10.25)	(9.89-10.28)	(9.89-10.30)	(9.89-10.32)
Cuba	9.76	9.78	9.81	9.84	9.87	9.90	9.93	9.96	9.99
	(9.72-9.79)	(9.73-9.84)	(9.75-9.88)	(9.77-9.92)	(9.79-9.96)	(9.81-9.99)	(9.83-10.03)	(9.85-10.07)	(9.87-10.10)
Cyprus	10.25	10.22	10.19	10.16	10.13	10.10	10.07	10.04	10.01
	(10.16-10.34)	(10.05-10.39)	(9.93-10.45)	(9.79-10.53)	(9.64-10.62)	(9.48-10.72)	(9.31-10.83)	(9.14-10.94)	(8.95-11.07)
Czech Republic	11.07	11.14	11.21	11.27	11.34	11.41	11.48	11.54	11.61
	(10.97-11.16)	(11.00-11.27)	(11.04-11.37)	(11.08-11.46)	(11.13-11.55)	(11.18-11.64)	(11.22-11.73)	(11.28-11.81)	(11.33-11.90)
Denmark	10.97	10.99	11.02	11.04	11.06	11.09	11.11	11.13	11.16
	(10.92-11.02)	(10.90-11.08)	(10.88-11.15)	(10.85-11.23)	(10.81-11.32)	(10.77-11.41)	(10.72-11.50)	(10.67-11.60)	(10.61-11.70)
Djibouti	8.17	8.24	8.31	8.38	8.45	8.53	8.60	8.67	8.74
	(8.10-8.23)	(8.14-8.34)	(8.18-8.45)	(8.21-8.56)	(8.24-8.67)	(8.26-8.79)	(8.28-8.91)	(8.30-9.03)	(8.32-9.16)

(Continue to next page)

	2017	2018	2019	2020	2021	2022	2023	2024	2025
(Continued from previous page)									
DominicanRepublic	11.71	11.91	12.12	12.33	12.53	12.74	12.94	13.15	13.36
	(10.26-13.16)	(10.31-13.51)	(10.35-13.89)	(10.37-14.29)	(10.37-14.70)	(10.35-15.12)	(10.33-15.56)	(10.29-16.01)	(10.24-16.47)
Timor-Leste	9.92	10.02	10.12	10.23	10.33	10.43	10.53	10.63	10.73
	(9.21-10.63)	(9.31-10.74)	(9.41-10.84)	(9.50-10.95)	(9.60-11.05)	(9.70-11.15)	(9.80-11.26)	(9.90-11.36)	(9.99-11.47)
Ecuador	9.75	9.78	9.82	9.86	9.89	9.93	9.97	10.00	10.04
	(9.68-9.81)	(9.72-9.85)	(9.75-9.88)	(9.79-9.92)	(9.83-9.96)	(9.86-10.00)	(9.90-10.03)	(9.94-10.07)	(9.97-10.11)
El Salvador	9.01	9.01	9.01	9.01	9.01	9.01	9.01	9.01	9.01
	(8.93-9.09)	(8.92-9.10)	(8.89-9.13)	(8.86-9.16)	(8.82-9.20)	(8.77-9.25)	(8.72-9.30)	(8.66-9.35)	(8.60-9.41)

Equatorial Guinea	8.82	8.82	8.82	8.83	8.83	8.83	8.83	8.84	8.84
	(8.64-9.00)	(8.46-9.18)	(8.26-9.39)	(8.02-9.63)	(7.77-9.89)	(7.48-10.18)	(7.18-10.49)	(6.85-10.82)	(6.51-11.17)
Eritrea	7.57	7.61	7.65	7.69	7.73	7.77	7.82	7.86	7.90
	(6.95-8.19)	(6.99-8.23)	(7.03-8.28)	(7.07-8.32)	(7.11-8.36)	(7.15-8.40)	(7.19-8.44)	(7.23-8.48)	(7.27-8.52)
Estonia	10.46	10.50	10.54	10.57	10.60	10.62	10.65	10.66	10.68
	(10.19-10.72)	(10.12-10.87)	(10.08-10.99)	(10.04-11.10)	(10.01-11.19)	(9.98-11.27)	(9.95-11.35)	(9.91-11.41)	(9.88-11.47)
Ethiopia	8.17	8.17	8.17	8.17	8.17	8.17	8.17	8.17	8.17
	(8.11-8.23)	(8.09-8.25)	(8.07-8.27)	(8.06-8.29)	(8.04-8.30)	(8.03-8.31)	(8.02-8.32)	(8.01-8.33)	(8.00-8.34)
Fiji	8.17	8.17	8.17	8.17	8.17	8.17	8.17	8.17	8.17
	(8.11-8.23)	(8.09-8.25)	(8.07-8.27)	(8.06-8.29)	(8.04-8.30)	(8.03-8.31)	(8.02-8.32)	(8.01-8.33)	(8.00-8.34)
Finland	11.25	11.28	11.32	11.36	11.39	11.43	11.46	11.49	11.53
	(11.19-11.30)	(11.21-11.36)	(11.23-11.41)	(11.25-11.46)	(11.28-11.51)	(11.30-11.55)	(11.32-11.60)	(11.35-11.64)	(11.37-11.68)
France	10.65	10.66	10.67	10.68	10.69	10.70	10.71	10.72	10.73
	(10.59-10.70)	(10.56-10.75)	(10.52-10.81)	(10.48-10.88)	(10.43-10.95)	(10.37-11.02)	(10.31-11.11)	(10.25-11.19)	(10.18-11.28)
Gabon	9.05	9.15	9.25	9.34	9.44	9.54	9.64	9.74	9.83
	(8.91-9.19)	(8.94-9.35)	(8.96-9.53)	(8.97-9.72)	(8.97-9.91)	(8.96-10.12)	(8.95-10.32)	(8.93-10.54)	(8.91-10.76)
Gambia	9.10	9.14	9.18	9.22	9.26	9.30	9.35	9.39	9.44
	(9.04-9.17)	(9.05-9.23)	(9.07-9.29)	(9.09-9.35)	(9.12-9.41)	(9.14-9.46)	(9.18-9.52)	(9.21-9.58)	(9.24-9.63)
Germany	10.72	10.74	10.76	10.78	10.80	10.82	10.83	10.84	10.86
	(10.70-10.75)	(10.71-10.78)	(10.72-10.81)	(10.73-10.83)	(10.74-10.86)	(10.75-10.88)	(10.76-10.90)	(10.77-10.92)	(10.78-10.93)
Ghana	8.13	8.18	8.23	8.28	8.33	8.38	8.43	8.48	8.52
	(8.05-8.22)	(8.04-8.33)	(8.01-8.46)	(7.97-8.59)	(7.93-8.73)	(7.88-8.88)	(7.82-9.04)	(7.75-9.20)	(7.68-9.37)
Greece	10.33	10.32	10.30	10.29	10.27	10.26	10.24	10.23	10.21
	(10.29-10.37)	(10.23-10.40)	(10.17-10.43)	(10.11-10.46)	(10.04-10.50)	(9.97-10.55)	(9.89-10.60)	(9.80-10.66)	(9.71-10.71)
Guatemala	8.94	8.94	8.94	8.94	8.94	8.93	8.93	8.93	8.93
	(8.87-9.01)	(8.85-9.02)	(8.83-9.05)	(8.80-9.08)	(8.76-9.11)	(8.71-9.15)	(8.67-9.20)	(8.61-9.25)	(8.56-9.31)
Guinea	7.79	7.84	7.88	7.93	7.98	8.03	8.08	8.13	8.18

	(7.69-7.88)	(7.71-7.96)	(7.73-8.04)	(7.74-8.13)	(7.75-8.21)	(7.76-8.30)	(7.77-8.40)	(7.77-8.49)	(7.77-8.59)
Guyana	8.71	8.85	8.99	9.13	9.27	9.41	9.55	9.69	9.83
	(8.62-8.79)	(8.66-9.04)	(8.67-9.30)	(8.67-9.59)	(8.64-9.89)	(8.60-10.21)	(8.55-10.55)	(8.48-10.89)	(8.40-11.25)
Honduras	8.48	8.51	8.55	8.58	8.62	8.66	8.69	8.73	8.76
	(8.07-8.89)	(8.10-8.92)	(8.14-8.96)	(8.17-9.00)	(8.21-9.03)	(8.24-9.07)	(8.27-9.11)	(8.31-9.14)	(8.34-9.18)
Hungary	9.97	10.01	10.06	10.10	10.15	10.19	10.23	10.28	10.32
	(9.85-10.08)	(9.85-10.18)	(9.86-10.26)	(9.87-10.33)	(9.89-10.40)	(9.91-10.47)	(9.93-10.54)	(9.95-10.61)	(9.98-10.67)
Iceland	10.79	10.79	10.78	10.78	10.78	10.78	10.78	10.77	10.77
	(10.76-10.82)	(10.73-10.84)	(10.69-10.88)	(10.65-10.92)	(10.60-10.96)	(10.54-11.01)	(10.49-11.07)	(10.42-11.13)	(10.36-11.19)

(Continue to next page)

	2017	2018	2019	2020	2021	2022	2023	2024	2025
(Continued from previous page)									
India	9.73	9.80	9.87	9.95	10.02	10.10	10.18	10.26	10.34
	(9.69-9.77)	(9.74-9.86)	(9.80-9.95)	(9.86-10.03)	(9.93-10.12)	(9.99-10.21)	(10.06-10.29)	(10.13-10.38)	(10.21-10.47)
Indonesia	8.78	8.85	8.92	8.99	9.06	9.13	9.21	9.28	9.36
	(8.59-8.97)	(8.57-9.12)	(8.58-9.25)	(8.60-9.38)	(8.62-9.49)	(8.65-9.61)	(8.69-9.72)	(8.73-9.84)	(8.78-9.95)
Iraq	10.17	10.17	10.17	10.17	10.17	10.17	10.17	10.17	10.17
	(9.87-10.47)	(9.74-10.59)	(9.65-10.69)	(9.57-10.77)	(9.49-10.84)	(9.43-10.90)	(9.37-10.96)	(9.32-11.02)	(9.26-11.07)
Ireland	10.70	10.70	10.70	10.70	10.70	10.71	10.71	10.71	10.71
	(10.63-10.76)	(10.57-10.83)	(10.50-10.90)	(10.42-10.99)	(10.33-11.08)	(10.23-11.18)	(10.13-11.29)	(10.01-11.40)	(9.89-11.53)
Iran	11.23	11.29	11.34	11.40	11.46	11.51	11.57	11.62	11.68
	(10.99-11.47)	(10.96-11.62)	(10.91-11.77)	(10.86-11.94)	(10.80-12.11)	(10.73-12.29)	(10.65-12.48)	(10.57-12.68)	(10.48-12.88)
Israel	10.72	10.73	10.75	10.76	10.76	10.77	10.77	10.77	10.77
	(10.61-10.82)	(10.58-10.89)	(10.56-10.93)	(10.54-10.97)	(10.52-11.01)	(10.50-11.04)	(10.49-11.06)	(10.47-11.08)	(10.45-11.10)
Italy	10.61	10.63	10.64	10.66	10.67	10.69	10.70	10.71	10.72
	(10.56-10.66)	(10.56-10.69)	(10.56-10.73)	(10.56-10.76)	(10.56-10.78)	(10.57-10.81)	(10.57-10.83)	(10.57-10.85)	(10.58-10.87)

Jamaica	9.57 (9.50-9.64)	9.58 (9.48-9.68)	9.60 (9.48-9.71)	9.61 (9.47-9.75)	9.63 (9.47-9.78)	9.64 (9.47-9.81)	9.66 (9.48-9.84)	9.67 (9.48-9.87)	9.69 (9.48-9.90)
Japan	10.84 (10.79-10.89)	10.89 (10.84-10.95)	10.94 (10.88-11.00)	10.99 (10.93-11.06)	11.05 (10.98-11.11)	11.10 (11.02-11.17)	11.15 (11.07-11.22)	11.20 (11.12-11.28)	11.25 (11.17-11.33)
Jordan	11.04 (10.93-11.16)	10.89 (10.64-11.15)	10.74 (10.31-11.18)	10.60 (9.97-11.23)	10.45 (9.59-11.30)	10.30 (9.20-11.40)	10.15 (8.79-11.51)	10.00 (8.36-11.65)	9.85 (7.91-11.80)
Kazakhstan	9.03 (8.92-9.14)	8.97 (8.72-9.22)	8.91 (8.50-9.33)	8.86 (8.25-9.46)	8.80 (7.98-9.62)	8.74 (7.69-9.80)	8.69 (7.38-9.99)	8.63 (7.05-10.21)	8.57 (6.70-10.44)
Kenya	8.35 (8.27-8.42)	8.44 (8.28-8.61)	8.54 (8.26-8.82)	8.63 (8.23-9.04)	8.73 (8.18-9.28)	8.83 (8.12-9.53)	8.92 (8.05-9.80)	9.02 (7.96-10.07)	9.11 (7.86-10.36)
Kiribati	7.81 (7.79-7.83)	7.85 (7.80-7.90)	7.89 (7.81-7.98)	7.94 (7.82-8.06)	7.98 (7.82-8.14)	8.02 (7.81-8.23)	8.07 (7.81-8.33)	8.11 (7.79-8.42)	8.15 (7.78-8.52)
Kuwait	11.38 (11.27-11.49)	11.30 (11.06-11.54)	11.22 (10.81-11.63)	11.14 (10.54-11.74)	11.06 (10.25-11.87)	10.98 (9.94-12.02)	10.90 (9.61-12.19)	10.82 (9.26-12.38)	10.74 (8.90-12.59)
Kyrgyzstan	8.77 (8.68-8.86)	8.76 (8.57-8.96)	8.76 (8.42-9.09)	8.75 (8.26-9.23)	8.74 (8.08-9.40)	8.73 (7.88-9.58)	8.72 (7.67-9.77)	8.71 (7.44-9.98)	8.70 (7.20-10.21)
Laos	8.24 (8.19-8.30)	8.32 (8.24-8.39)	8.39 (8.29-8.49)	8.47 (8.35-8.58)	8.54 (8.42-8.66)	8.62 (8.48-8.75)	8.69 (8.54-8.84)	8.76 (8.61-8.92)	8.84 (8.67-9.01)
Lebanon	11.84 (11.60-12.09)	11.90 (11.55-12.24)	11.95 (11.53-12.37)	12.01 (11.52-12.49)	12.06 (11.51-12.60)	12.11 (11.52-12.71)	12.17 (11.52-12.81)	12.22 (11.53-12.91)	12.28 (11.55-13.01)
Lesotho	6.66 (6.49-6.83)	6.83 (6.45-7.21)	7.00 (6.37-7.63)	7.17 (6.25-8.10)	7.34 (6.09-8.60)	7.51 (5.90-9.13)	7.69 (5.68-9.69)	7.86 (5.44-10.27)	8.03 (5.17-10.88)
Liberia	9.21 (8.73-9.69)	9.28 (8.79-9.76)	9.35 (8.86-9.83)	9.41 (8.92-9.90)	9.48 (8.99-9.97)	9.54 (9.05-10.04)	9.61 (9.12-10.11)	9.68 (9.18-10.18)	9.74 (9.24-10.24)
Lithuania	9.93 (9.67-10.19)	9.89 (9.53-10.25)	9.84 (9.40-10.29)	9.79 (9.27-10.30)	9.73 (9.15-10.30)	9.66 (9.03-10.28)	9.58 (8.90-10.26)	9.49 (8.76-10.22)	9.40 (8.62-10.17)
Luxembourg	11.16	11.16	11.17	11.17	11.17	11.17	11.18	11.18	11.18

	(11.09-11.23)	(11.03-11.30)	(10.96-11.38)	(10.87-11.46)	(10.78-11.56)	(10.68-11.66)	(10.58-11.78)	(10.46-11.90)	(10.34-12.02)
Macedonia	9.28	9.33	9.38	9.44	9.49	9.55	9.60	9.66	9.72
	(9.21-9.35)	(9.23-9.43)	(9.26-9.51)	(9.29-9.58)	(9.33-9.65)	(9.37-9.72)	(9.41-9.79)	(9.46-9.86)	(9.50-9.93)

(Continue to next page)

	2017	2018	2019	2020	2021	2022	2023	2024	2025
(Continued from previous page)									
Malawi	7.57	7.67	7.78	7.88	7.99	8.09	8.20	8.30	8.41
	(7.47-7.67)	(7.47-7.88)	(7.44-8.12)	(7.39-8.37)	(7.33-8.65)	(7.25-8.93)	(7.16-9.24)	(7.05-9.55)	(6.94-9.88)
Malaysia	9.18	9.21	9.25	9.29	9.33	9.36	9.40	9.44	9.48
	(9.14-9.21)	(9.16-9.27)	(9.17-9.33)	(9.18-9.40)	(9.19-9.47)	(9.19-9.54)	(9.19-9.61)	(9.19-9.69)	(9.19-9.77)
Maldives	10.36	10.43	10.51	10.58	10.66	10.74	10.81	10.89	10.96
	(10.06-10.65)	(10.11-10.76)	(10.15-10.86)	(10.2-10.97)	(10.25-11.07)	(10.3-11.17)	(10.35-11.27)	(10.41-11.37)	(10.46-11.47)
Mali	8.18	8.21	8.23	8.25	8.27	8.29	8.31	8.32	8.33
	(7.96-8.40)	(7.90-8.51)	(7.85-8.61)	(7.82-8.69)	(7.78-8.76)	(7.76-8.82)	(7.73-8.88)	(7.70-8.94)	(7.68-8.99)
Malta	10.51	10.52	10.52	10.53	10.54	10.55	10.55	10.56	10.57
	(10.46-10.56)	(10.41-10.62)	(10.34-10.71)	(10.27-10.80)	(10.18-10.90)	(10.09-11.01)	(9.98-11.13)	(9.87-11.25)	(9.75-11.39)
Mauritania	9.20	9.23	9.25	9.27	9.28	9.30	9.31	9.32	9.32
	(9.16-9.25)	(9.16-9.29)	(9.17-9.32)	(9.18-9.36)	(9.18-9.38)	(9.19-9.41)	(9.19-9.43)	(9.19-9.44)	(9.19-9.46)
Mexico	9.33	9.35	9.37	9.39	9.41	9.42	9.44	9.46	9.48
	(9.24-9.43)	(9.22-9.48)	(9.21-9.53)	(9.21-9.57)	(9.20-9.61)	(9.20-9.65)	(9.20-9.69)	(9.20-9.72)	(9.20-9.75)
Mongolia	8.14	8.07	8.00	7.93	7.86	7.79	7.71	7.64	7.57
	(8.03-8.25)	(7.83-8.32)	(7.59-8.41)	(7.33-8.53)	(7.04-8.67)	(6.74-8.83)	(6.42-9.01)	(6.08-9.21)	(5.72-9.42)
Montenegro	9.91	9.90	9.90	9.90	9.89	9.89	9.88	9.88	9.88
	(9.84-9.97)	(9.76-10.04)	(9.67-10.12)	(9.57-10.22)	(9.46-10.33)	(9.33-10.45)	(9.2-10.57)	(9.05-10.71)	(8.9-10.85)
Morocco	11.02	10.99	10.95	10.89	10.83	10.75	10.66	10.55	10.43
	(10.98-11.07)	(10.91-11.06)	(10.82-11.07)	(10.72-11.06)	(10.60-11.06)	(10.46-11.04)	(10.30-11.01)	(10.12-10.97)	(9.93-10.93)

Mozambique	8.09	8.24	8.39	8.54	8.69	8.84	8.99	9.15	9.30
	(7.98-8.20)	(8.02-8.46)	(8.03-8.76)	(8.02-9.07)	(7.99-9.39)	(7.95-9.74)	(7.89-10.10)	(7.82-10.47)	(7.73-10.86)
Namibia	8.37	8.43	8.49	8.55	8.61	8.68	8.74	8.80	8.86
	(8.23-8.50)	(8.12-8.73)	(7.98-9.00)	(7.81-9.29)	(7.61-9.62)	(7.39-9.96)	(7.15-10.33)	(6.88-10.72)	(6.59-11.13)
Netherlands	10.93	10.93	10.92	10.91	10.91	10.90	10.89	10.89	10.88
	(10.89-10.98)	(10.83-11.02)	(10.77-11.07)	(10.70-11.13)	(10.62-11.20)	(10.53-11.27)	(10.43-11.35)	(10.34-11.43)	(10.23-11.53)
new Zealand	10.70	10.72	10.73	10.74	10.74	10.75	10.75	10.75	10.75
	(10.65-10.75)	(10.64-10.79)	(10.63-10.82)	(10.63-10.84)	(10.62-10.86)	(10.62-10.88)	(10.61-10.89)	(10.60-10.90)	(10.59-10.91)
Nicaragua	9.43	9.46	9.49	9.52	9.54	9.57	9.60	9.63	9.66
	(9.24-9.62)	(9.27-9.65)	(9.30-9.68)	(9.32-9.71)	(9.35-9.74)	(9.38-9.77)	(9.40-9.80)	(9.43-9.83)	(9.46-9.85)
Niger	7.70	7.75	7.79	7.84	7.88	7.92	7.97	8.01	8.06
	(7.66-7.75)	(7.68-7.81)	(7.71-7.88)	(7.73-7.95)	(7.74-8.02)	(7.76-8.09)	(7.77-8.17)	(7.78-8.24)	(7.79-8.32)
Nigeria	8.84	8.93	9.03	9.13	9.22	9.32	9.42	9.51	9.61
	(8.75-8.92)	(8.77-9.10)	(8.77-9.29)	(8.76-9.49)	(8.74-9.71)	(8.71-9.93)	(8.66-10.17)	(8.61-10.41)	(8.55-10.66)
Norway	10.64	10.67	10.70	10.73	10.76	10.80	10.83	10.86	10.89
	(10.59-10.69)	(10.60-10.74)	(10.61-10.79)	(10.63-10.84)	(10.65-10.88)	(10.67-10.92)	(10.69-10.97)	(10.71-11.01)	(10.74-11.05)
Pakistan	9.04	9.09	9.14	9.19	9.25	9.30	9.35	9.40	9.45
	(8.92-9.15)	(8.93-9.24)	(8.95-9.34)	(8.95-9.43)	(8.95-9.54)	(8.95-9.64)	(8.95-9.75)	(8.94-9.86)	(8.93-9.97)
Panama	9.58	9.61	9.65	9.69	9.72	9.76	9.80	9.83	9.87
	(9.53-9.62)	(9.53-9.69)	(9.52-9.78)	(9.50-9.87)	(9.47-9.97)	(9.44-10.08)	(9.40-10.19)	(9.36-10.31)	(9.31-10.43)
Papua New Guinea	7.73	7.79	7.85	7.91	7.97	8.04	8.10	8.16	8.22
	(7.64-7.82)	(7.66-7.91)	(7.69-8.01)	(7.70-8.12)	(7.72-8.23)	(7.73-8.34)	(7.74-8.46)	(7.74-8.58)	(7.75-8.70)
Paraguay	9.52	9.53	9.53	9.54	9.54	9.55	9.55	9.56	9.56
	(9.49-9.56)	(9.48-9.58)	(9.47-9.60)	(9.47-9.61)	(9.46-9.62)	(9.46-9.64)	(9.46-9.65)	(9.46-9.66)	(9.46-9.67)

(Continue to next page)

	2017	2018	2019	2020	2021	2022	2023	2024	2025

(Continued from previous page)

Peru	9.96<br>(9.84-10.08)	10.03<br>(9.86-10.19)	10.09<br>(9.88-10.29)	10.15<br>(9.92-10.39)	10.22<br>(9.95-10.48)	10.28<br>(9.99-10.57)	10.34<br>(10.03-10.66)	10.41<br>(10.07-10.74)	10.47<br>(10.12-10.83)
Philippines	8.46<br>(8.40-8.52)	8.46<br>(8.38-8.54)	8.46<br>(8.36-8.56)	8.46<br>(8.35-8.57)	8.46<br>(8.33-8.59)	8.46<br>(8.32-8.60)	8.46<br>(8.31-8.61)	8.46<br>(8.30-8.62)	8.46<br>(8.29-8.63)
Poland	10.37<br>(10.24-10.50)	10.41<br>(10.22-10.59)	10.44<br>(10.21-10.67)	10.47<br>(10.21-10.74)	10.50<br>(10.21-10.80)	10.53<br>(10.21-10.85)	10.56<br>(10.21-10.91)	10.58<br>(10.21-10.95)	10.60<br>(10.21-11.00)
Portugal	10.49<br>(10.43-10.55)	10.48<br>(10.40-10.57)	10.47<br>(10.35-10.59)	10.46<br>(10.29-10.63)	10.45<br>(10.22-10.68)	10.44<br>(10.15-10.73)	10.43<br>(10.07-10.79)	10.42<br>(9.99-10.85)	10.41<br>(9.90-10.92)
Qatar	11.64<br>(11.56-11.71)	11.56<br>(11.39-11.73)	11.48<br>(11.20-11.77)	11.41<br>(10.99-11.82)	11.33<br>(10.76-11.89)	11.25<br>(10.53-11.98)	11.18<br>(10.27-12.08)	11.10<br>(10.01-12.19)	11.02<br>(9.74-12.31)
Republic of Congo	8.37<br>(8.17-8.57)	8.40<br>(8.20-8.61)	8.44<br>(8.24-8.64)	8.48<br>(8.27-8.68)	8.51<br>(8.31-8.72)	8.55<br>(8.34-8.75)	8.58<br>(8.38-8.79)	8.62<br>(8.41-8.83)	8.66<br>(8.45-8.86)
Republic of Guinea-Bissau	7.44<br>(7.34-7.55)	7.52<br>(7.38-7.67)	7.61<br>(7.43-7.79)	7.70<br>(7.49-7.90)	7.79<br>(7.55-8.02)	7.88<br>(7.63-8.14)	7.98<br>(7.70-8.26)	8.08<br>(7.79-8.38)	8.19<br>(7.87-8.50)
Republic of Korea	10.17<br>(10.05-10.29)	10.10<br>(9.87-10.32)	10.03<br>(9.68-10.37)	9.96<br>(9.47-10.44)	9.89<br>(9.25-10.52)	9.82<br>(9.01-10.62)	9.75<br>(8.77-10.73)	9.68<br>(8.51-10.85)	9.61<br>(8.24-10.97)
Romania	9.90<br>(9.73-10.07)	9.96<br>(9.79-10.13)	10.01<br>(9.84-10.18)	10.07<br>(9.90-10.24)	10.13<br>(9.95-10.30)	10.18<br>(10.01-10.36)	10.24<br>(10.07-10.42)	10.30<br>(10.12-10.47)	10.35<br>(10.18-10.53)
Rwanda	8.39<br>(8.14-8.64)	8.52<br>(8.17-8.87)	8.64<br>(8.21-9.07)	8.76<br>(8.27-9.26)	8.89<br>(8.33-9.45)	9.01<br>(8.40-9.62)	9.14<br>(8.48-9.80)	9.26<br>(8.55-9.96)	9.38<br>(8.63-10.13)
Saint Lucia	9.73<br>(9.63-9.83)	9.71<br>(9.53-9.89)	9.69<br>(9.41-9.96)	9.67<br>(9.28-10.05)	9.65<br>(9.14-10.15)	9.63<br>(8.99-10.26)	9.61<br>(8.83-10.38)	9.59<br>(8.66-10.51)	9.57<br>(8.48-10.65)
Saint Vincent and the Grenadines	71.73<br>(71.34-72.11)	71.82<br>(71.27-72.36)	71.91<br>(71.24-72.58)	72.00<br>(71.23-72.78)	72.10<br>(71.24-72.97)	72.21<br>(71.26-73.15)	72.31<br>(71.29-73.33)	72.42<br>(71.33-73.51)	72.53<br>(71.38-73.69)
Sao Tome and Principe	8.97<br>(8.88-9.05)	8.99<br>(8.82-9.15)	9.01<br>(8.74-9.27)	9.03<br>(8.66-9.39)	9.05<br>(8.56-9.53)	9.07<br>(8.45-9.68)	9.09<br>(8.33-9.84)	9.11<br>(8.20-10.01)	9.13<br>(8.07-10.19)
Saudi Arabia	10.78	10.85	10.92	10.98	11.05	11.11	11.18	11.25	11.31

	(10.71-10.85)	(10.72-10.98)	(10.70-11.13)	(10.68-11.28)	(10.65-11.44)	(10.61-11.61)	(10.57-11.79)	(10.51-11.98)	(10.45-12.17)
Senegal	8.38	8.41	8.43	8.46	8.49	8.51	8.54	8.57	8.60
	(8.31-8.44)	(8.30-8.51)	(8.27-8.59)	(8.24-8.68)	(8.21-8.77)	(8.17-8.86)	(8.13-8.96)	(8.08-9.06)	(8.02-9.17)
Seychelles	8.77	8.80	8.84	8.88	8.92	8.95	8.99	9.03	9.07
	(8.72-8.81)	(8.74-8.87)	(8.76-8.92)	(8.79-8.97)	(8.81-9.02)	(8.84-9.07)	(8.87-9.11)	(8.90-9.16)	(8.93-9.20)
Sierra Leone	7.32	7.35	7.38	7.40	7.43	7.46	7.49	7.52	7.54
	(6.96-7.68)	(6.99-7.71)	(7.01-7.74)	(7.04-7.77)	(7.06-7.80)	(7.09-7.83)	(7.12-7.86)	(7.14-7.89)	(7.17-7.92)
Singapore	10.16	10.22	10.27	10.33	10.38	10.43	10.49	10.54	10.60
	(10.08-10.25)	(10.09-10.34)	(10.12-10.43)	(10.15-10.50)	(10.18-10.58)	(10.22-10.65)	(10.26-10.72)	(10.29-10.79)	(10.33-10.86)
Slovak Republic	10.13	10.17	10.22	10.26	10.30	10.35	10.39	10.43	10.48
	(10.03-10.22)	(10.04-10.30)	(10.05-10.38)	(10.07-10.45)	(10.09-10.51)	(10.12-10.58)	(10.14-10.64)	(10.17-10.70)	(10.19-10.76)
Slovenia	11.50	11.47	11.45	11.43	11.41	11.39	11.36	11.34	11.32
	(11.40-11.59)	(11.30-11.65)	(11.18-11.72)	(11.06-11.80)	(10.92-11.89)	(10.78-11.99)	(10.62-12.10)	(10.46-12.22)	(10.29-12.35)
Solomon Islands	7.79	7.84	7.89	7.94	7.99	8.04	8.09	8.14	8.19
	(7.74-7.84)	(7.75-7.93)	(7.76-8.02)	(7.76-8.12)	(7.76-8.22)	(7.75-8.33)	(7.74-8.44)	(7.72-8.56)	(7.70-8.68)
South Africa	8.74	8.88	9.02	9.15	9.29	9.43	9.57	9.70	9.84
	(8.59-8.90)	(8.54-9.22)	(8.46-9.57)	(8.35-9.96)	(8.20-10.38)	(8.03-10.83)	(7.83-11.30)	(7.61-11.79)	(7.37-12.30)

(Continue to next page)

	2017	2018	2019	2020	2021	2022	2023	2024	2025
(Continued from previous page)									
Spain	83.16	83.34	83.52	83.69	83.86	84.03	84.19	84.35	84.51
	(82.99-83.32)	(83.10-83.58)	(83.22-83.81)	(83.35-84.03)	(83.48-84.24)	(83.61-84.44)	(83.74-84.64)	(83.87-84.83)	(84.00-85.01)
Sri Lanka	9.66	9.71	9.75	9.79	9.84	9.88	9.92	9.97	10.01
	(9.26-10.06)	(9.30-10.11)	(9.35-10.15)	(9.39-10.20)	(9.43-10.24)	(9.48-10.28)	(9.52-10.33)	(9.56-10.37)	(9.61-10.41)
Sudan	10.17	10.22	10.27	10.31	10.36	10.40	10.44	10.49	10.53
	(10.10-10.24)	(10.12-10.32)	(10.14-10.39)	(10.17-10.46)	(10.19-10.52)	(10.22-10.58)	(10.25-10.64)	(10.28-10.69)	(10.31-10.75)

Suriname	9.13	9.16	9.20	9.23	9.27	9.30	9.34	9.37	9.41
	(9.08-9.18)	(9.06-9.27)	(9.04-9.36)	(9.00-9.47)	(8.96-9.58)	(8.91-9.69)	(8.86-9.82)	(8.79-9.95)	(8.73-10.09)
Swaziland	8.15	8.49	8.82	9.15	9.49	9.82	10.16	10.49	10.82
	(7.92-8.38)	(7.98-8.99)	(7.99-9.64)	(7.96-10.35)	(7.88-11.10)	(7.76-11.88)	(7.61-12.70)	(7.42-13.56)	(7.21-14.44)
Sweden	11.20	11.23	11.27	11.31	11.34	11.38	11.42	11.45	11.49
	(11.16-11.24)	(11.18-11.29)	(11.20-11.34)	(11.22-11.39)	(11.25-11.44)	(11.28-11.48)	(11.31-11.53)	(11.33-11.57)	(11.36-11.61)
Switzerland	11.19	11.13	11.08	11.02	10.97	10.91	10.86	10.80	10.75
	(11.12-11.26)	(10.99-11.28)	(10.84-11.32)	(10.67-11.38)	(10.49-11.45)	(10.30-11.53)	(10.10-11.62)	(9.89-11.72)	(9.67-11.83)
Tajikistan	8.76	8.82	8.89	8.95	9.01	9.07	9.13	9.20	9.26
	(8.61-8.91)	(8.63-9.02)	(8.64-9.13)	(8.64-9.25)	(8.65-9.37)	(8.64-9.50)	(8.63-9.63)	(8.62-9.77)	(8.61-9.91)
Tanzania	7.86	7.96	8.05	8.15	8.25	8.35	8.45	8.54	8.64
	(7.78-7.94)	(7.78-8.13)	(7.76-8.35)	(7.73-8.58)	(7.68-8.82)	(7.61-9.08)	(7.54-9.36)	(7.45-9.64)	(7.35-9.94)
Thailand	9.75	9.73	9.70	9.68	9.65	9.63	9.61	9.58	9.56
	(9.67-9.83)	(9.58-9.88)	(9.47-9.94)	(9.35-10.01)	(9.22-10.09)	(9.08-10.18)	(8.93-10.28)	(8.78-10.39)	(8.61-10.51)
The Russian Federation	8.85	8.74	8.63	8.52	8.41	8.30	8.19	8.08	7.97
	(8.59-9.10)	(8.21-9.26)	(7.78-9.48)	(7.29-9.74)	(6.77-10.05)	(6.20-10.39)	(5.60-10.77)	(4.97-11.18)	(4.31-11.63)
Togo	7.77	7.82	7.87	7.92	7.97	8.02	8.07	8.12	8.17
	(7.66-7.88)	(7.64-8.00)	(7.62-8.12)	(7.59-8.24)	(7.56-8.37)	(7.53-8.51)	(7.49-8.64)	(7.45-8.79)	(7.40-8.93)
Tonga	9.17	9.20	9.22	9.25	9.27	9.30	9.33	9.35	9.38
	(9.01-9.33)	(9.03-9.36)	(9.06-9.39)	(9.08-9.41)	(9.11-9.44)	(9.14-9.46)	(9.16-9.49)	(9.19-9.51)	(9.21-9.54)
Trinidad and Tobago	9.51	9.52	9.53	9.53	9.54	9.55	9.55	9.56	9.56
	(9.44-9.59)	(9.38-9.66)	(9.31-9.75)	(9.23-9.84)	(9.14-9.94)	(9.04-10.05)	(8.94-10.16)	(8.83-10.29)	(8.71-10.41)
Tunisia	10.80	10.74	10.58	10.52	10.45	10.39	10.32	10.25	10.19
	(10.76-10.84)	(10.65-10.84)	(10.42-10.74)	(10.28-10.75)	(10.13-10.77)	(9.98-10.79)	(9.82-10.82)	(9.65-10.86)	(9.47-10.91)
Turkey	11.54	11.52	11.49	11.45	11.40	11.35	11.29	11.22	11.15
	(11.24-11.84)	(11.10-11.94)	(10.97-12.00)	(10.85-12.04)	(10.74-12.07)	(10.62-12.08)	(10.50-12.08)	(10.38-12.07)	(10.25-12.04)
Turkmenistan	8.20	8.23	8.25	8.27	8.29	8.31	8.33	8.35	8.37

	(8.16-8.25)	(8.13-8.32)	(8.09-8.40)	(8.04-8.49)	(7.99-8.59)	(7.92-8.70)	(7.85-8.81)	(7.77-8.94)	(7.68-9.06)
Uganda	7.93	8.01	8.09	8.17	8.24	8.32	8.40	8.48	8.55
	(7.84-8.03)	(7.88-8.14)	(7.93-8.25)	(7.98-8.35)	(8.03-8.45)	(8.09-8.55)	(8.15-8.65)	(8.21-8.74)	(8.27-8.84)
Ukraine	9.14	9.21	9.28	9.35	9.42	9.50	9.58	9.66	9.74
	(8.88-9.40)	(8.84-9.57)	(8.83-9.72)	(8.83-9.87)	(8.85-10.00)	(8.87-10.13)	(8.90-10.26)	(8.93-10.39)	(8.97-10.52)
United Arab Emirates	10.39	10.36	10.32	10.28	10.24	10.20	10.16	10.12	10.09
	(10.32-10.47)	(10.21-10.51)	(10.08-10.55)	(9.95-10.61)	(9.81-10.67)	(9.66-10.75)	(9.50-10.83)	(9.33-10.92)	(9.15-11.02)
United Kingdom	10.43	10.43	10.43	10.43	10.43	10.43	10.43	10.43	10.43
	(10.36-10.50)	(10.34-10.53)	(10.31-10.55)	(10.30-10.57)	(10.28-10.59)	(10.26-10.60)	(10.25-10.61)	(10.24-10.63)	(10.23-10.64)
United States	11.16	11.16	11.16	11.16	11.15	11.15	11.15	11.15	11.14
	(11.12-11.21)	(11.07-11.25)	(11.01-11.31)	(10.94-11.37)	(10.86-11.44)	(10.78-11.52)	(10.69-11.61)	(10.59-11.70)	(10.49-11.80)

(Continue to next page)

	2017	2018	2019	2020	2021	2022	2023	2024	2025
(Continued from previous page)									
Uruguay	9.43	9.46	9.50	9.53	9.57	9.60	9.64	9.67	9.71
	(9.38-9.48)	(9.39-9.54)	(9.41-9.59)	(9.43-9.64)	(9.45-9.69)	(9.47-9.73)	(9.50-9.78)	(9.52-9.82)	(9.55-9.87)
US Virgin Islands	9.09	9.11	9.13	9.14	9.16	9.17	9.19	9.20	9.22
	(9.06-9.13)	(9.07-9.16)	(9.07-9.18)	(9.08-9.21)	(9.09-9.23)	(9.10-9.25)	(9.11-9.27)	(9.11-9.29)	(9.12-9.31)
Uzbekistan	8.49	8.48	8.48	8.48	8.48	8.48	8.47	8.47	8.47
	(8.43-8.54)	(8.36-8.61)	(8.27-8.69)	(8.17-8.79)	(8.06-8.89)	(7.94-9.01)	(7.81-9.14)	(7.67-9.27)	(7.52-9.42)
Vanuatu	7.93	7.96	7.99	8.01	8.04	8.07	8.10	8.12	8.15
	(7.79-8.07)	(7.80-8.12)	(7.79-8.18)	(7.79-8.24)	(7.78-8.30)	(7.77-8.37)	(7.76-8.44)	(7.74-8.50)	(7.73-8.58)
Venezuela	8.97	9.00	9.02	9.05	9.07	9.10	9.12	9.15	9.17
	(8.70-9.24)	(8.73-9.26)	(8.75-9.29)	(8.78-9.32)	(8.80-9.34)	(8.82-9.37)	(8.85-9.40)	(8.87-9.42)	(8.90-9.45)
Vietnam	8.66	8.68	8.70	8.73	8.75	8.77	8.79	8.81	8.83
	(8.63-8.69)	(8.63-8.73)	(8.64-8.76)	(8.66-8.79)	(8.67-8.82)	(8.69-8.85)	(8.70-8.88)	(8.72-8.91)	(8.73-8.94)

Zambia	7.29<br>(7.15-7.43)	7.42<br>(7.15-7.69)	7.55<br>(7.12-7.98)	7.68<br>(7.08-8.28)	7.81<br>(7.01-8.61)	7.94<br>(6.92-8.95)	8.07<br>(6.82-9.31)	8.19<br>(6.71-9.68)	8.32<br>(6.58-10.07)
Zimbabwe	7.33<br>(7.15-7.50)	7.53<br>(7.17-7.89)	7.73<br>(7.14-8.32)	7.93<br>(7.08-8.78)	8.13<br>(7.00-9.27)	8.34<br>(6.89-9.79)	8.54<br>(6.75-10.33)	8.74<br>(6.59-10.89)	8.94<br>(6.41-11.47)
